# Nickel-Catalyzed Enantioselective Synthesis of Pre-Differentiated Homoallylic *syn*- or *anti*-1,2-Diols from Aldehydes and Dienol Ethers

**DOI:** 10.1021/jacs.1c07042

**Published:** 2021-08-19

**Authors:** Thomas Q. Davies, John J. Murphy, Maxime Dousset, Alois Fürstner

**Affiliations:** Max-Planck-Institut für Kohlenforschung, 45470 Mülheim/Ruhr, Germany

## Abstract

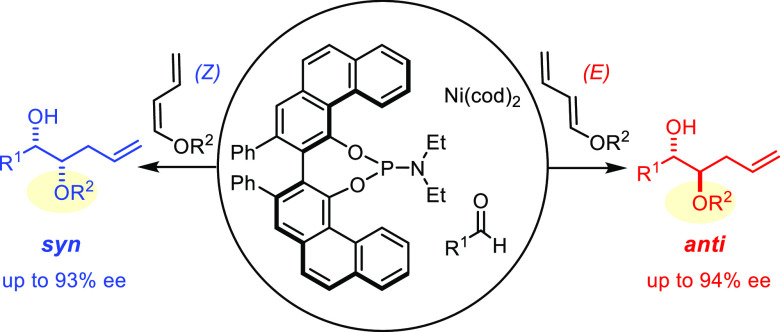

Nickel catalysis allied with cyclodiphosphazane or VAPOL-derived phosphoramidite ligands provides selective access to monoprotected vicinal diols by reductive coupling of dienol ethers and aldehydes. The observed regioselectivity is unprecedented, in that the diene reacts at the least nucleophilic and most hindered C atom that is attached to the oxygen substituent rather than at the terminal position. Notably, both *syn* and *anti* diastereomers of the products can be accessed depending on the configuration of the diene partner with usually excellent diastereo- and enantioselectivity.

In the 1990s, the groups of Tamaru and Mori pioneered the nickel-catalyzed reductive coupling of aldehydes with 1,3-dienes, notably isoprene.^1−5^ The benefits of this “isoprenylation,” or in its generalized format homoallylation reaction, include generally mild conditions and high levels of diastereoselectivity (Scheme 1A). Later, it was shown that different functional groups such as aryl substituents,^6^ boronic esters,^7,8^ silanes,^9^ or stannanes^10^ can be placed on the carbon chain, expanding the utility of the transformation. However, enantioselective variants remain relatively unexplored and were described in a recent review as a “largely unresolved challenge”.^11^ The first two intermolecular examples use a SPINOL-derived phosphoramidite (**3**, Scheme 1B) or a chiral N-heterocyclic carbene to couple symmetrical 1,4-diaryl dienes with aldehydes.^12,13^ Likewise, the asymmetric coupling of diene **4** with certain aldehydes in the presence of a silylborane to give products such as **5** is known (Scheme 1C), but the scope is again rather limited.^9^

**Scheme 1:**
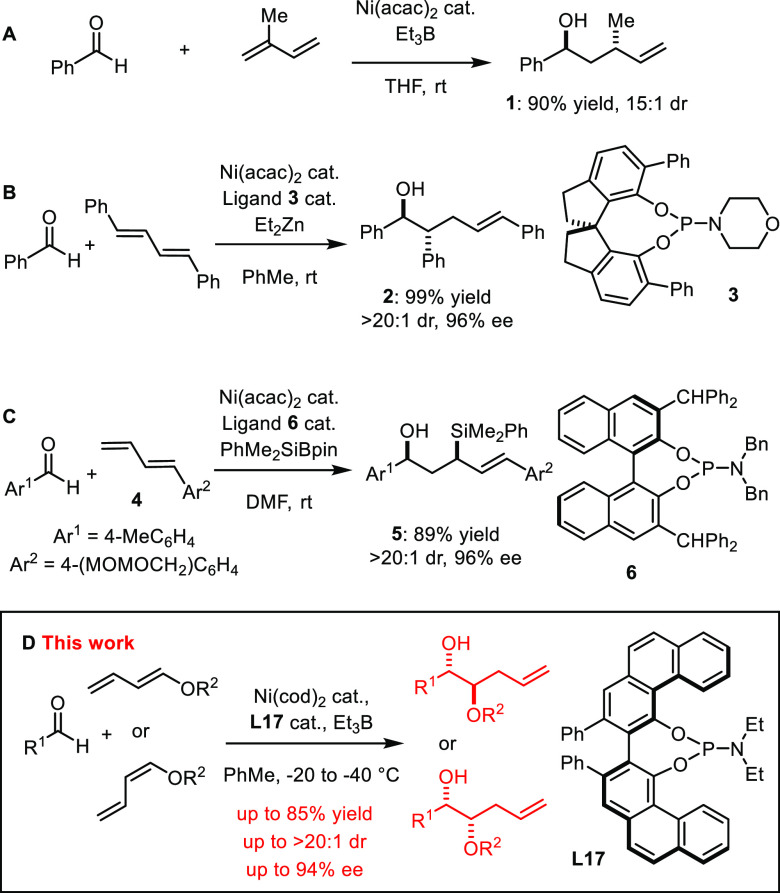
(A) The Original Intermolecular Nickel-Catalyzed “Tamaru Reaction;” (B) the First Enantioselective Variant (ref (12)); (C) a Silylative Asymmetric Variant (ref (13)); (D) This Work: Unprecedented Regioselectivity Enables Enantioselective Access to Monoprotected 1,2-Diols

The lack of catalyst control also surfaced during a recent total synthesis campaign, where we tried to take advantage of nickel-catalyzed isoprenylations of sugar-derived aldehydes; however, the inability to overwrite the stereochemical bias of some substrates with the aid of chiral nickel complexes marked an inherent limitation of this approach.^14,15^ Confronted with this impasse, we embarked into a more systematic investigation, during which an unexpected and, to the best of our knowledge, unprecedented reactivity mode was discovered.^16^ The preliminary results of this new diastereo- and enantioselective approach to monoprotected vicinal diols are summarized below (Scheme 1D).

Our studies began with the coupling of silyloxydiene **7** with hydrocinnamaldehyde using Ni(cod)_2_ as a catalyst and triethylborane as reductant. No conversion was observed in the absence of a ligand.^17^ With triphenylphosphine added, we observed not only the expected “Tamaru product” **8** but also the 1,2-diol derivative **9a** in a 1.2:1 ratio (Figure 1).

**Figure 1:**
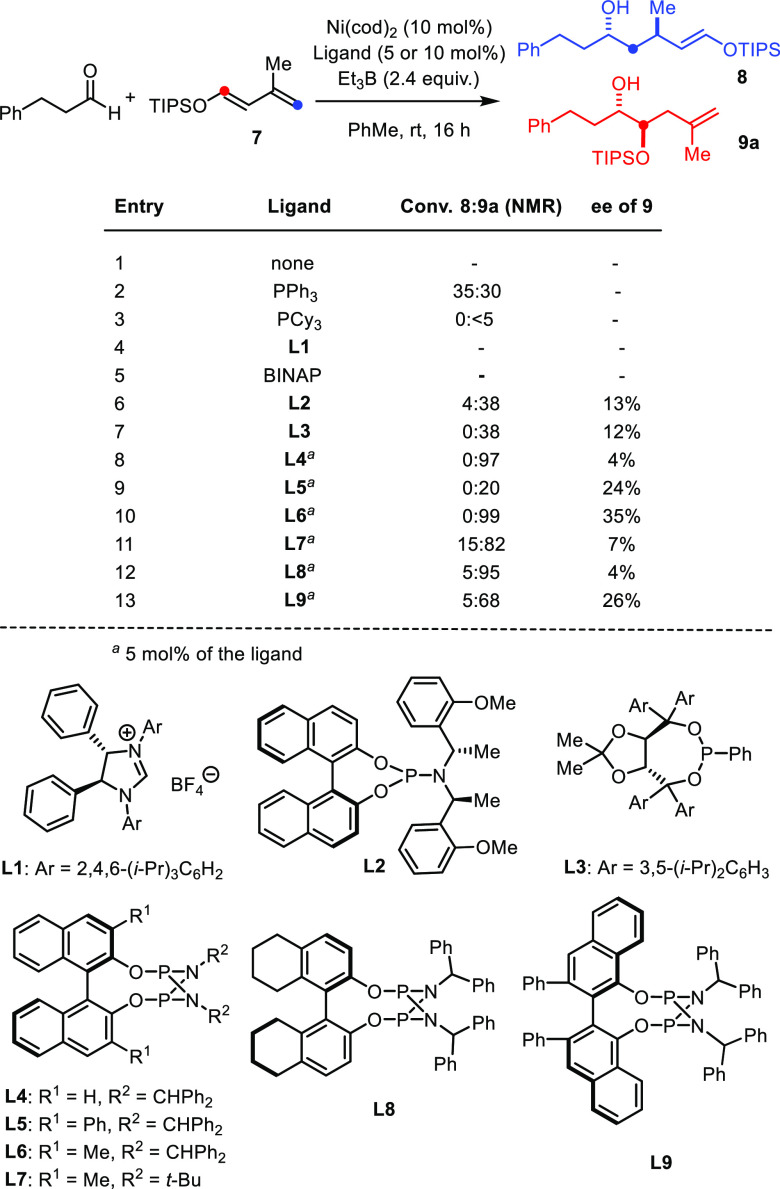
Initial ligand screening.

This outcome is striking as it implies attack of the silyloxy diene **7** at C1 rather than C4, which is the least nucleophilic and, at the same time, arguably most hindered site.^18^ If this reactivity pattern can be generalized, however, a new and potentially highly enabling entry into vicinal diols is gained; on top of the stereochemical virtues, it allows the two hydroxy groups to be rigorously discriminated in that one of them is delivered as the free alcohol, whereas the other one carries a protecting group. For this trait, such an approach nicely complements the traditional arsenal.^19−34^

Increasing the donor strength of the ligand (PCy_3_, NHC,^35^**L1**) or moving to a bidentate phosphine (BINAP) suppressed any conversion; more electron-deficient ligands, including phosphoramidite **L2** and bulky TADDOL-derived phosphonite **L3**,^36^ fared better, giving good or even complete selectivity in favor of diol **9a**, although the conversion and ee were low. Switching to the cyclodiphosphazane ligand **L4** proved key,^37,38^ with the diol product obtained with excellent regioselectivity, diastereoselectivity, and quantitative yield (NMR), though in virtually racemic form. Extensive efforts were made to improve the level of induction by (i) placing substituents at the 3,3′-positions of the ligand’s BINOL subunit (**L5** and **L6**), (ii) varying the amine part (**L7**), (iii) using octahydro-BINOL (**L8**), and (iv) replacing BINOL with VANOL (**L9**).^39^ Even though approximately 40 different chiral cyclodiphosphazanes were prepared and screened, many of which are synthetically quite challenging, ee’s were generally poor, and no result with both >40% conversion and >40% ee could be obtained (for full details, see the SI).^40^ Therefore, we first studied the racemic reaction using **L6** to see if there was any relevant scope.

Gratifyingly, linear (**9a**–**9d**) and branched (**9e**–**9f**) alkyl aldehydes performed well, and even sterically hindered pivaldehyde still gave an acceptable 40% yield (**9g**; Scheme 2). Likewise, aromatic and heteroaromatic aldehydes proved compliant and allowed the compatibility of the reaction with various polar substituents such as esters, nitriles, and ketones to be demonstrated (for additional functionality, see Scheme 5). Furfural also led to excellent results, whereas thiophene-2-carbaldehyde reacted sluggishly because of the thiophilicity of the nickel catalyst. Unsurprisingly, perhaps, pyridine-2-carbaldehyde failed to react under standard conditions, also likely because the heteroatom donor site functions as a competitive ligand for Ni(0). An α,β-unsaturated aldehyde was also transformed more slowly but did eventually give product **9k** in 46% yield and 20:1 dr.^41^ With regard to the reaction partner, excellent yields and remarkably high diastereoselectivities were maintained using dienyl ethers with −OTES and −OTBS groups; the latter proved particularly adequate when working with dienes lacking the methyl substituent at C3, where the −OTIPS derivative led to a slightly lower dr (compare **9i**/**9j**).

**Scheme 2:**
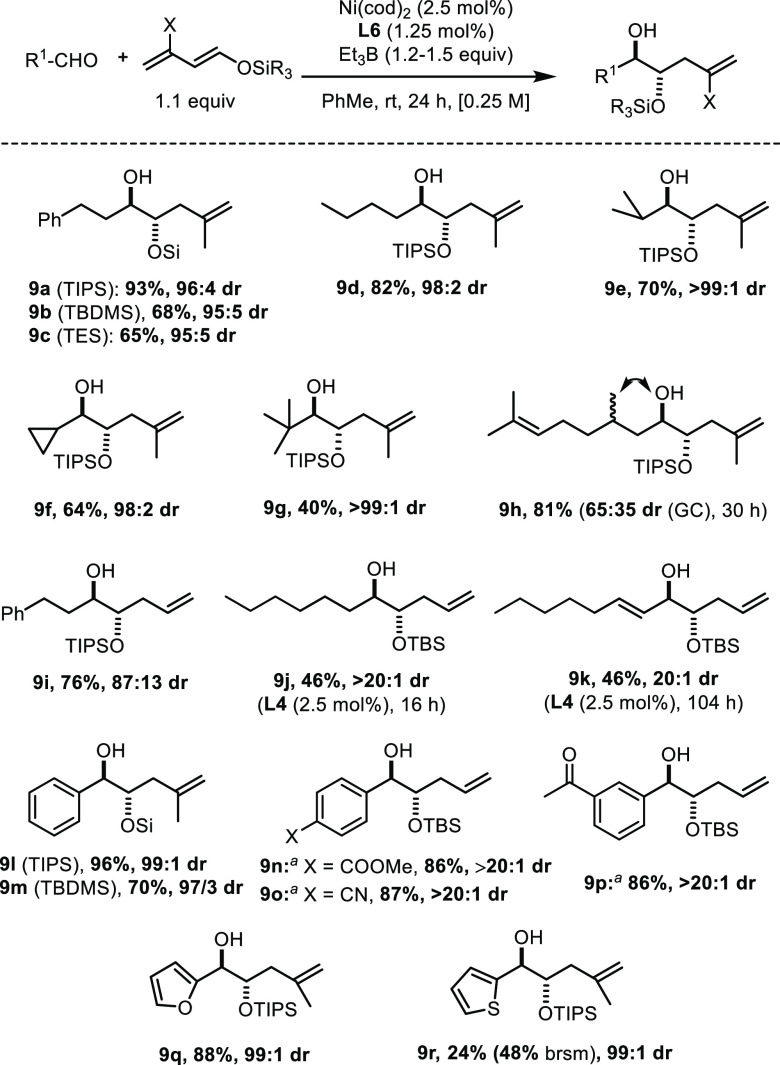
Scope of the (Virtually) Racemic Reaction^42^ Using Ni(cod)_2_ and **L4** (5 mol % each).

The major diastereomer formed was confirmed as the 1,2-*anti* diol by comparison of product **9j** derived from heptanal with authentic material prepared by a known literature route (see the SI).^43^

The use of (*Z*)-configured silyloxydienes (**10**) led to a stark reversal of diastereoselectivity as shown by the formation of the *syn*-configured diol derivatives **11a** and **11b** (Scheme 3). Furthermore, the trisubstituted diene **12** furnished product **13** in 91% yield as a single diastereomer (for the enantioselective version, see Scheme 5); this result proves that the high 1,3-*anti* selectivity characteristic of the original Tamaru isoprenylation^4^ is retained in the new diol synthesis even though C–C-bond formation now occurs at the head- rather than the tail-end of the dienolsilane partner.

**Scheme 3:**
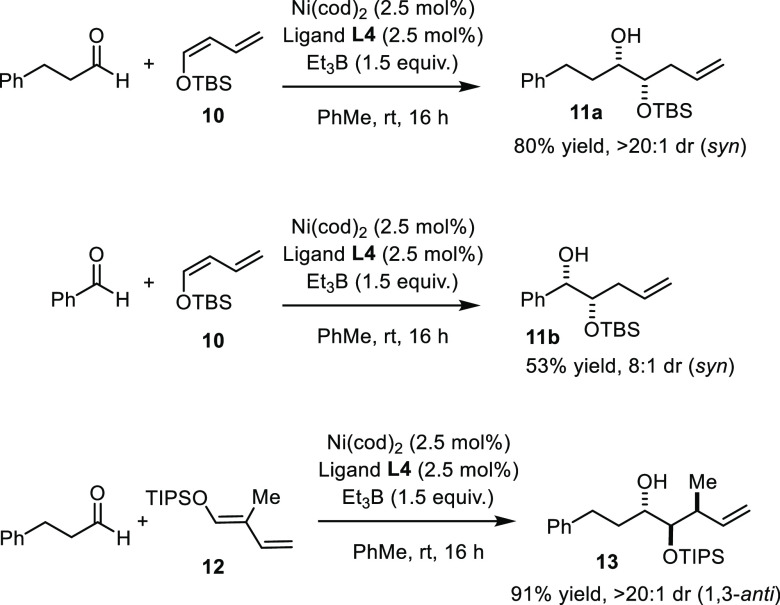
Stereochemical Effects of Alternative Dienes

In analogy to the mechanism of the Tamaru homoallylation,^4,44^ we propose that the new reaction proceeds through nickel-induced oxidative cyclization of the dienyl ether^45^ and the aldehyde to give nickelacycle **14** (Scheme 4). The bulky cyclodiphosphazane backbone dictates the relative orientation of the reaction partners on the loaded catalyst, such that a steric clash between the bulky ligand and the oxygen substituent on the dienyl ether is avoided; this array explains the formation of the 1,2-diol product. In line with this notion, sterically less demanding (but still reactive) ligands such as PPh_3_ lead to product mixtures. The diastereoselectivity results from the position of the R group of the aldehyde: when equatorially oriented, unfavorable 1,3-diaxial interactions across the metallacyclic ring are prevented; this likely includes transannular collisions with the ligand L on nickel, since the bulky cyclophosphazane **L6** entails a better dr than slimmer PPh_3_.

**Scheme 4:**
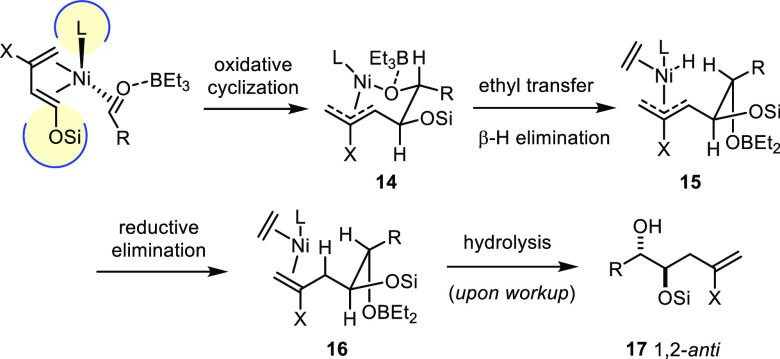
Proposed Mechanism

Coordination of Lewis-acidic triethylborane aids the cyclization by reducing the electron density of the carbonyl group; moreover, the subsequent ethyl transfer to nickel is rendered quasi-intramolecular and hence more facile. β-Hydride elimination then gives ethylene and the nickel hydride species **15**, which undergoes reductive elimination to release product **16** (leading to **17** upon hydrolysis of the B–O bond during workup) and regenerate the catalytically active nickel(0) species.

Convinced by these results of the utility of the reaction, we redoubled our efforts to develop an enantioselective variant (Figure 2). For the lack of any real hit, cyclodiphosphazanes were not pursued any further.^40^ Because of the literature precedent^12^ (see Scheme 1B), ligands **L10** and **L11** comprising a SPINOL backbone seemed promising; their use, however, was to no avail either. Likewise, BINOL-based phosphoramidites including **L12**–**L14** were rapidly ruled out, despite their excellent track record in asymmetric catalysis.^46^

**Figure 2:**
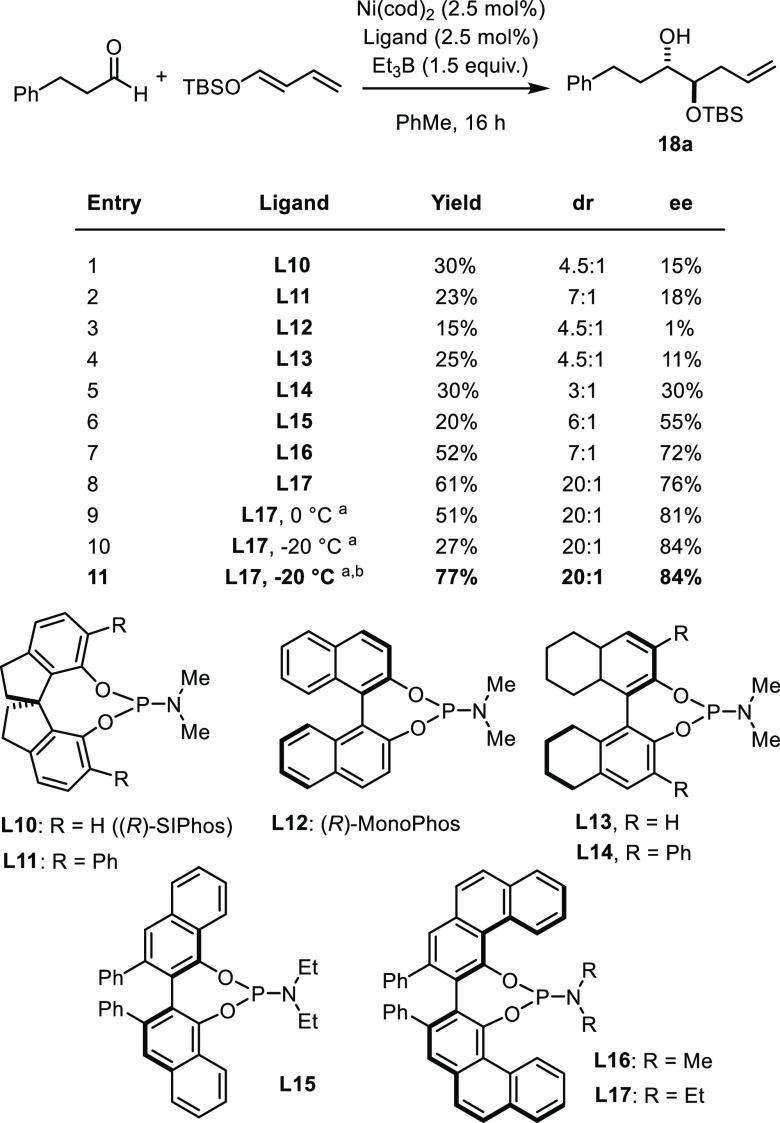
Optimization of the enantioselective reaction. ^*a*^72 h reaction time. ^*b*^3.0 equiv diene, 10 mol % Ni/**L17**.

For these systematic failures, we were prompted to revisit the design. Rather than forging a chiral cleft on the “backside” of the ligands as, e.g., in the case of BINOL-derived phosphoramidites, it seemed warranted to enlarge the “major groove” on the front side in the hope of crafting an effective (helically) chiral environment about the nickel center. Indeed, a first promising result was obtained with the VANOL-phosphoramidite derivative **L15**,^39^ which gave product **18a** with 55% ee. Extending the π-system further, as manifested in the VAPOL derivative **L16**,^39^ improved the outcome to 72% ee. The yield, dr, and ee were all boosted using the diethylamino analogue **L17**; further changes to the amine substituents, however, did not lead to any significant improvements (for details, see the SI). Varying the solvent had little effect, whereas lowering the temperature to 0 °C or −20 °C resulted in 81% and 84% ee, respectively; the decrease in conversion could be compensated by using an excess of the diene and a higher catalyst loading of 10 mol % (entry 11). Under these conditions, **18a** was obtained in 77% yield, 20:1 dr, and 84% ee.^47^ To the best of our knowledge, this represents the first use of a VAPOL-phosphoramidite in nickel catalysis.^48−50^

These conditions were then used to survey the scope of the enantioselective reaction. Hydrocinnamaldehyde, which had been chosen for the initial screening, actually turned out to be one of the more recalcitrant substrates, as evident from the results for compounds **13**, **18a**, and **18b**. Changing the O-protecting group on the dienyl ether hardly altered the attained ee’s (compare **18a**/**18b**, **18c**/**18d**, and **19a**/**19b**). In contrast, further lowering of the temperature to −40 °C had a notable effect for aryl aldehydes, though at the expense of a drop in yield (see **18e** and **19f**). Various aryl aldehydes with different steric and electronic properties were tested. Excellent ee’s were obtained for substrates bearing electron-donating, neutral, and weakly electron-withdrawing substituents. Even the presence of an *ortho*-methyl group is well tolerated (**18g**). The compatibility of the nickel-based catalyst system with an aryl chloride is also noteworthy (**18j**), as is the ability to run the reaction in the presence of an arylboronate group (**18l**), which opens numerous possibilities for downstream functionalization. The poorer result caused by the strongly electron-withdrawing 4-CF_3_ group (**18k**, 78% ee) reveals a limitation of the current catalyst system, which could not be ameliorated even by running the reaction at −60 °C (30% yield, 82% ee). In contrast, electron-rich furfural fared very well, furnishing product **18m** in excellent yield and selectivity.

Another interesting observation pertains to the *syn*-diol series. Whereas the results for aliphatic aldehydes were rather uniform, we were surprised to find that 4-phenylbenzyldehyde reacted less selectively with (*Z*)-configured dienes than with (*E*)-dienes (cf. **18e** versus **19e**, 74% versus 87/93% ee). Fortunately, recourse to a benzyl protecting group improved the outcome to a respectable 92% ee at −40 °C (**19f**); this result mandates further systematic survey. Finally, it is emphasized that the diastereoselectivity was invariably excellent in the *anti* as well as *syn* series; in many cases, the attained dr’s approach the limits of detection (NMR).

**Scheme 5:**
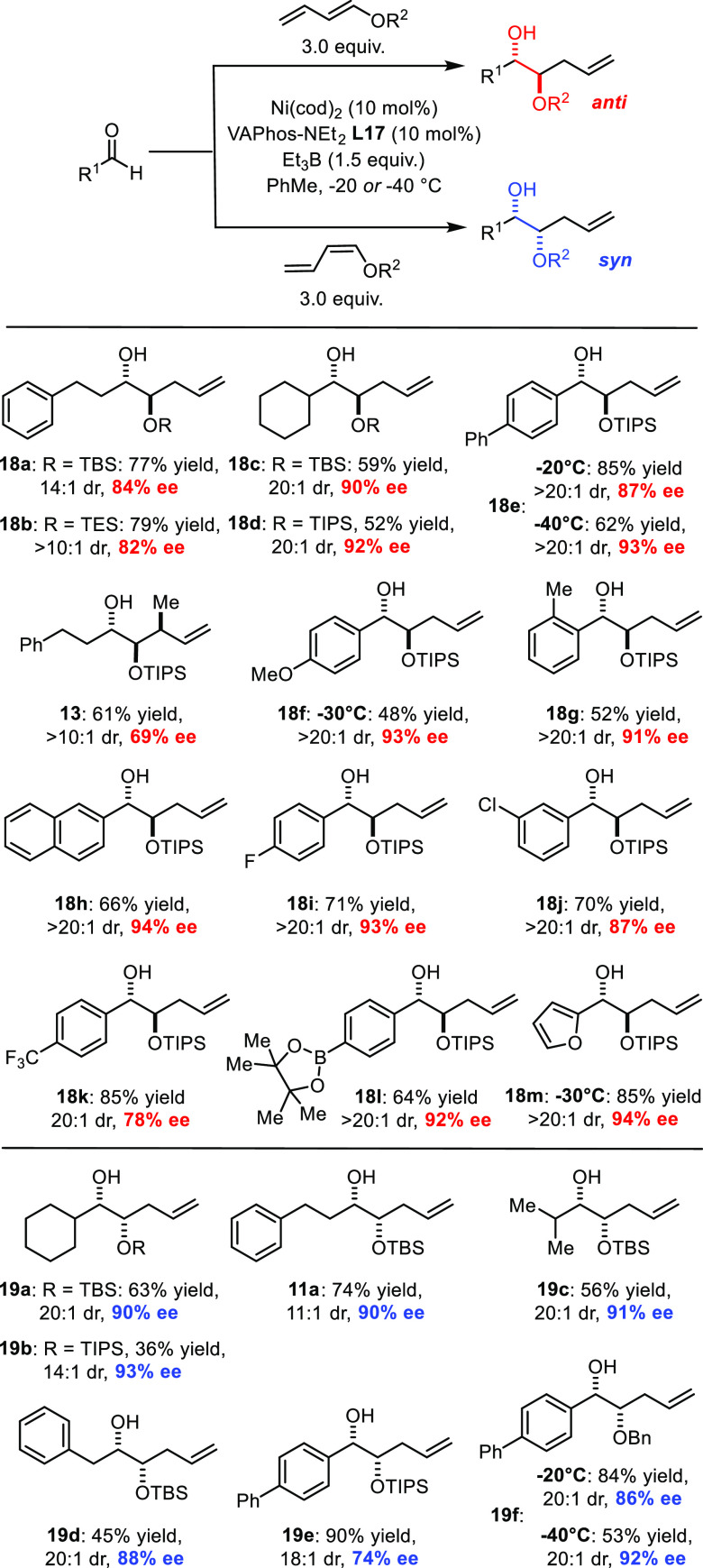
Scope of the Enantioselective Reaction

In conclusion, we have discovered a synthesis of monoprotected vicinal diols based on a nickel-catalyzed reductive coupling of dienol ethers and aldehydes that exhibits an unusual regioselective course and can selectively access either diastereomer of the product. The use of bulky, relatively electron-deficient phosphorus ligands including cyclodiphosphazane **L6** and VAPOL phosphoramidite **L17** proved key to unlocking this transformation. The presence of a silyl or benzyl group on one oxygen of the diol products should allow for selective functionalization; therefore, the new method nicely complements the traditional catalytic asymmetric toolbox which usually affords two unprotected vicinal hydroxy groups. Importantly, both *anti* and *syn* diol products can be obtained in invariably outstanding diastereoselectivity and often excellent enantioselectivity with a range of alkyl and aryl aldehydes. Work is underway to gain more mechanistic insights and increase the level of induction as well as the scope of the reaction even further.

## Supporting Information Available

Experimental procedures and supporting characterization data and spectra (PDF)
