# Supplementary material for: Nickel-Catalyzed Enantioselective Synthesis of Pre-Differentiated Homoallylic syn- or anti-1,2-Diols from Aldehydes and Dienol Ethers
Source: J Am Chem Soc. 2021 Aug 19;143(34):13489–94. doi: 10.1021/jacs.1c07042 (PMC8414482; doi:10.1021/jacs.1c07042)
Supplement: Supplementary file 1 — ja1c07042_si_001.pdf [file ja1c07042_si_001.pdf]

Supporting Information

Nickel-Catalyzed Enantioselective Synthesis of Pre-Differentiated Homoallylic *syn*- or *anti*-1,2-Diols from Aldehydes and Dienol Ethers

Thomas Q. Davies, John J. Murphy, Maxime Dousset, Alois Fürstner*

Max-Planck-Institut für Kohlenforschung, 45470 Mülheim/Ruhr, Germany

Email: fuerstner@kofo.mpg.de

1. Experimental S2
  1.1 General Considerations S2
  1.2 Reaction Optimisation Details S3
  1.3 Synthetic Procedures and Characterisation Data S11
    1.3.1 Synthesis of Ligands S11
    1.3.2 Ni-catalysed Reductive Coupling of Dienes and Aldehydes S35
      1.3.2.1 Racemic Scope S35
      1.3.2.2 Enantioselective Scope S45
    1.3.3 Synthesis of Dienyl Ethers S87
2. NMR Spectra S92
3. References S214

1. Experimental

1.1 General Considerations

Unless stated otherwise, all reactions were carried out under argon atmosphere in flame-dried Schlenk glassware. Solvents were purified by distillation over the indicated drying agents under argon: Toluene (CaH_2), THF (Mg/anthracene), Et_2O (Mg/anthacene), pentane (Na/K), CH_2Cl_2 (CaH_2). MeCN and Et_3N were dried by an absorption solvent purification system based on molecular sieves. Flash chromatography: VWR Chemicals silica gel 40 – 63 µm.

NMR spectra were recorded on Bruker DPX 300, AV 400, AV 500 or AV III 600 spectrometers in the solvents indicated; chemical shifts are given in ppm relative to TMS, coupling constants (*J*) in Hz. The solvent signals were used as references and the chemical shifts converted to the TMS scale ($CDCl_3$: δ_C = 77.16 ppm; δ_H = 7.26 ppm; C_6D_6: δ_C = 128.06 ppm; δ_H = 7.16 ppm; CD_2Cl_2: δ_C = 54.0 ppm; δ_H = 5.32 ppm). Proton and carbon assignments were established using HSQC, HMBC and NOESY experiments where necessary.

IR: Alpha Platinum ATR (Bruker), wavenumbers ($\tilde{\nu}$) in cm^{-1}.

MS (EI): Finnigan MAT 8200 (70 eV), ESI-MS: ESQ3000 (Bruker), Thermo Scientific LTQ-FT, or Thermo Scientific Exactive. HRMS: Bruker APEX III FT-MS (7T magnet), MAT 95 (Finnigan),Thermo Scientific LTQ-FT, or Thermo Scientific Exactive. GC-MS: Shimadzu GCMS-QP2010 Ultra instrument.

HPLC analyses for the determination of enantiomeric excesses were conducted on a Shimadzu LC 2020 instrument equipped with a Shimadzu SPD-M20A UV/VIS detector. Solvents (HPLC grade) were purchased and used as received. The exact conditions are stated separately for each compound.

Optical rotations were measured with an A-Krüss Otronic Model P8000-t polarimeter at a wavelength of 589 nm. The values are given as specific optical rotation with exact temperature, concentration (c/(10 mg/mL)) and solvent.

Aldehydes were purchased from commercial suppliers and distilled, except for solid aldehydes, which were typically used as received after checking purity by ^{1}H NMR spectroscopy. Unless stated otherwise, all other commercially available compounds (abcr, Acros, TCI, Aldrich, Alfa Aesar, Fluorochem) were used as received.

For compounds **L1,**[1] **L2,**[2] **L3,**[3] **L4,**[4] **L10,**[5] **L11,**[6] **L12,**[7] **L13,**[8] **L14,**[9] **SI-L11,**[4] **SI-L14,**[4] **SI-L15,**[4] **SI-L16,**[4] **SI-L17,**[4] and product **9j**,[10] see the cited literature

1.2 Reaction Optimisation Details

The initial optimisation revealed that cyclophosphazanes (particularly **L4** and **L6**) promoted the nickel-catalysed reductive coupling with excellent regio- and diastereoselectivity, but low enantioselectivity (see main paper). A number of these ligands were subsequently prepared and tested in the hope of obtaining useful levels of asymmetric induction. Their structures and results are shown below (note: for each ligand the R group on nitrogen is the same as the substituent on the other nitrogen). Despite varying the 3,3'-substituents on the BINOL scaffold and using amino R-groups with different levels of steric demand, in no case was even moderate enantioselectivity combined with good levels of conversion, and most changes hampered conversion relative to **L4** and **L6** (**Table S1**). Unfortunately, there was no clear trend to indicate which structural elements could be exploited to improve the results.

It must also be said here that the synthesis of some related ligands derived from other chiral diols was attempted but failed, likely due to the difficulty of closing the strained medium-sized ring of the cyclophosphazane with either very twisted (SPINOLs **SI-1a** and **SI-1b**) or extremely bulky precursors (BINOLs **SI-1c** and **SI-1d**, TADDOL **SI-1e**) shown below. Some of these limitations have previously been reported.[4] We cannot rule out that it is possible to prepare such structures, but under the standard conditions of refluxing in toluene with the dichlorocyclophosphazane intermediate, we did not observe appreciable amounts of the products.

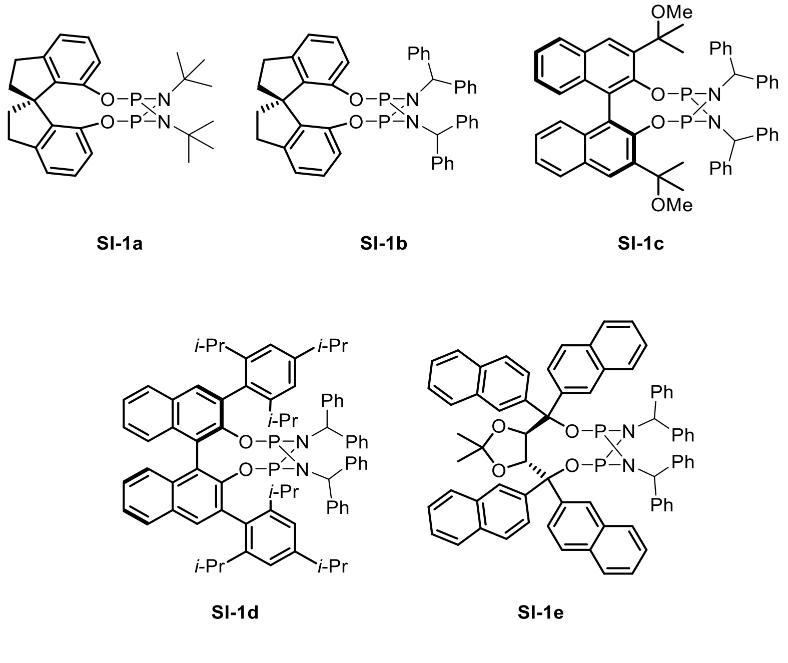

Scheme S1. Selection of ligand structures that could *not* be made.

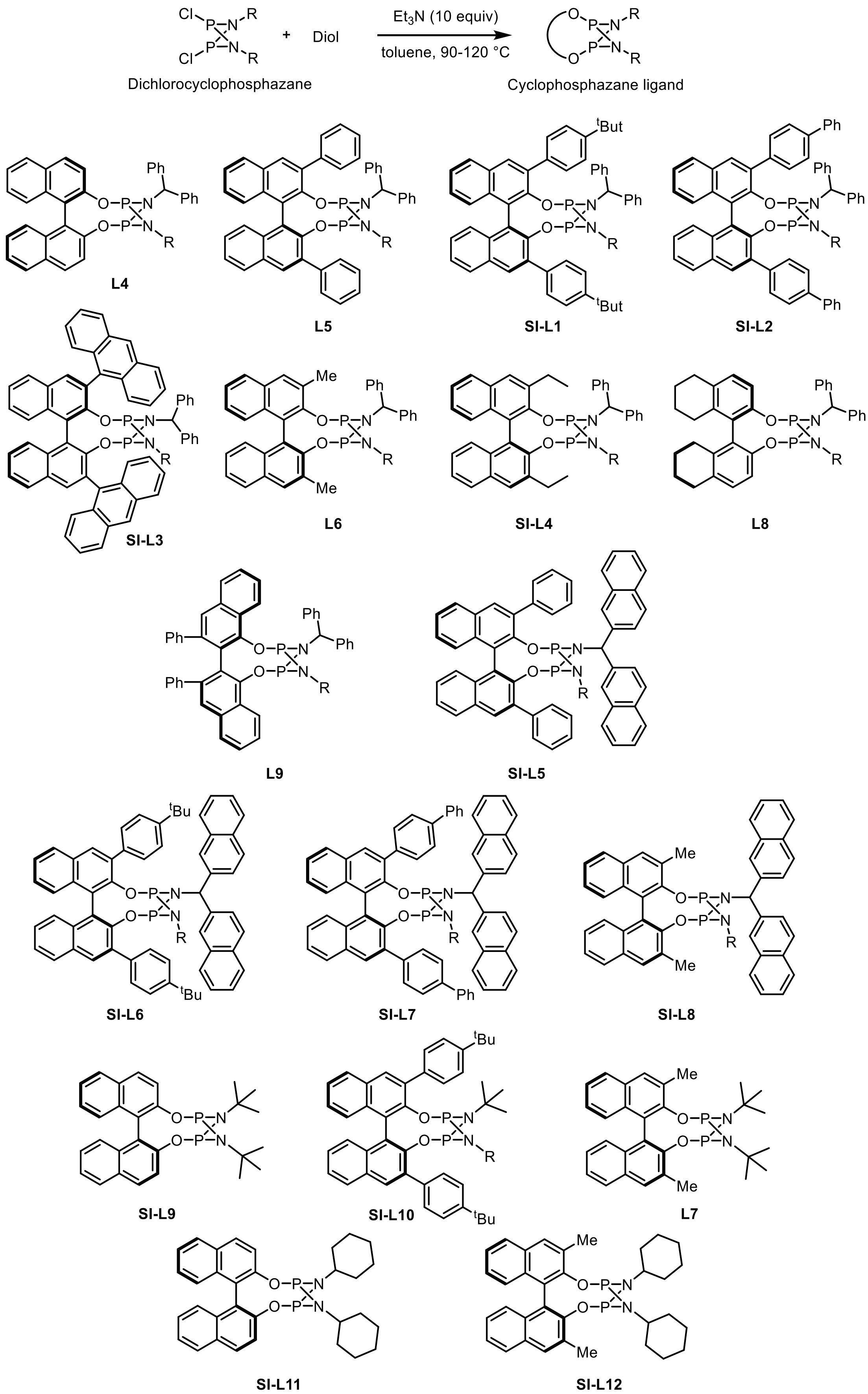

Scheme S2. Cyclophosphazane ligands prepared for the initial enantioselective screening.

Table S1. Initial round of ligand screening

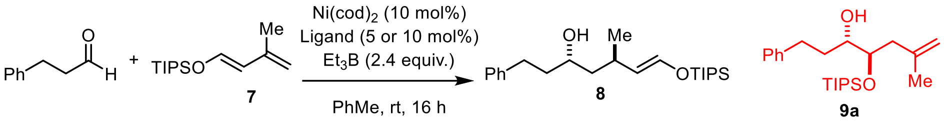

Entry	Ligand	Product (9a): GC ratio (%) (NMR) - (isolated) (%)	ee *anti* (%)	*anti* / *syn* dr
1	**L4**	96-(45)	4	98 / 2
2	**L5**	20 - (±5)	24	83 / 17
3	**SI-L1**	18 - (±5)	(-)47	69 / 31
4	**SI-L2**	6 - (±5)	(-)45	77 / 23
5	**SI-L3**	20 - (22) - (8)	40	92 / 8
6	**L6**	99 - (80) - (78)	35	93 / 7
7	**SI-L4**	99 – (70)	28	92 / 8
8	**L8**	95 - (90) - (63)	4	97 / 3
9	**L9**	68 - (67) - (40)	26	94 / 6
10	**SI-L5**	62 - (±5)	35	80 / 20
11	**SI-L6**	traces	31	55 / 45
12	**SI-L7**	12 - (±5)	31	57 / 43
13	**SI-L8**	97 - (42)	(-)10	93 / 7
14	**SI-L9**	0	-	-
15	**SI-L10**	45 - (16)	(-)15	94 / 6
16	**L7**	82 - (32)	(-)7	93 / 7
17	**SI-L11**	12 - (7)	12	86 / 14
18	**SI-L12**	0	-	-

We then prepared a selection of cyclophosphazanes mainly bearing chiral amine fragments and tested them under similar reaction conditions. Again, no clear trend could be interpreted and low levels of enantioselectivity were obtained in each case. However, it is notable that oxidising one phosphorus atom leads to a reversal of enantioselectivity (compare **L4** vs **SI-L13**). Desymmetrisation of the phosphorus atoms may be an interesting strategy to pursue for reactions which use cyclophosphazane ligands. The very high conversion observed with ligands **SI-L19** to **SI-L21** may make these useful ligands for the racemic reaction. Due to the lack of a promising hit at this stage, the racemic scope was performed using ligand **L6** (see manuscript).

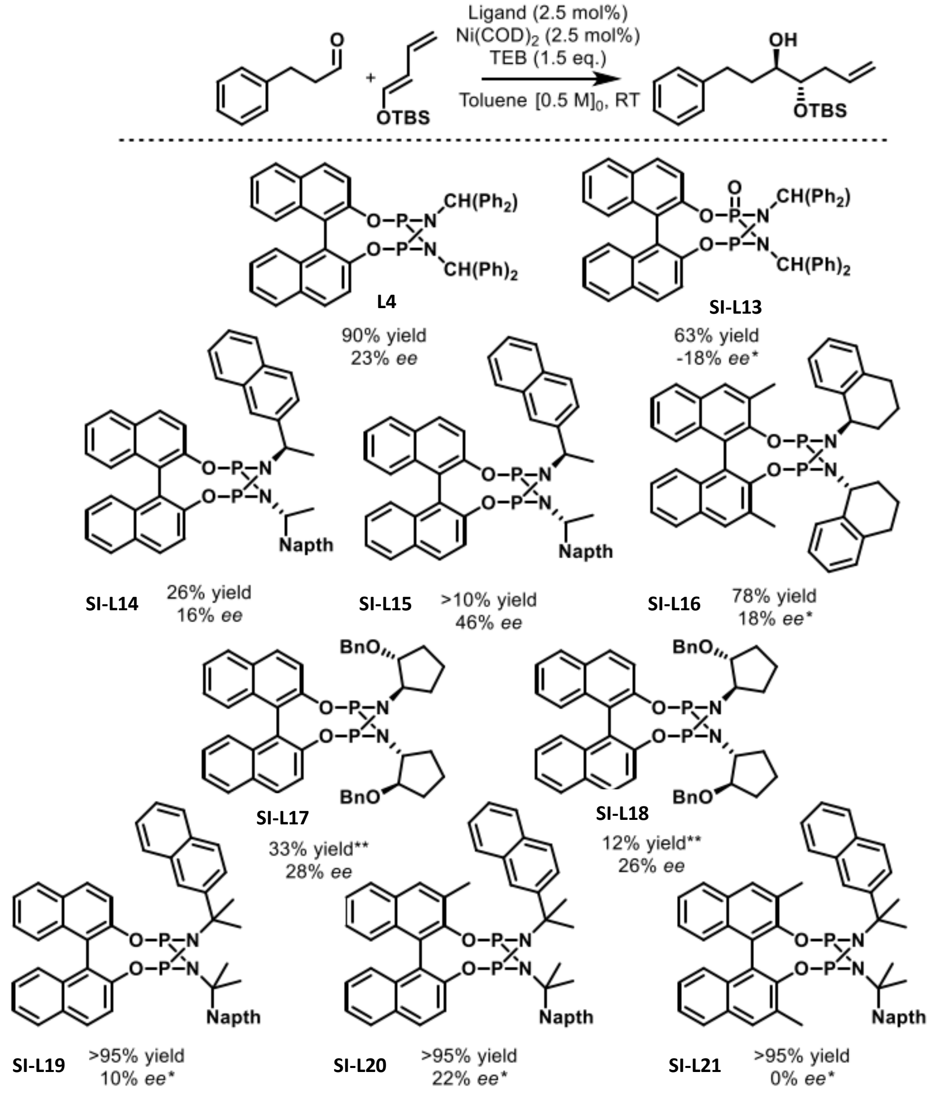

Scheme S3. Cyclophosphazane ligands used in the second round of screening [Note: compounds marked with an asterisk (*) were converted to the 2,4-dinitrobenzoate ester prior to chiral HPLC analysis]

After the scope of the reaction was found to be broad and the exciting discovery was made that the diastereomer of the diol product depended on the configuration of the diene, we resumed our efforts to render the reaction enantioselective. A number of cyclophosphazane ligands based on a rigid and sterically hindered *ortho*-substituted aniline scaffold were

prepared. Although these led to the highest ee's recorded for the reaction at this stage of the project (63% ee, **SI-L26**), the dr and conversion were generally poorer than the standard cyclophosphazane ligands **L4** and **L6**. Furthermore, increasing the steric bulk of the *ortho*-aryl group (**SI-L27**, **SI-L30**) or changing to an *ortho*-alkyl group (**SI-L28**, **SI-L29**) only led to worse results. Significantly lower ee's were obtained for the reaction with benzaldehyde instead of hydrocinnamaldehyde with **SI-L22** and **SI-L26**, suggesting that even if the selectivity could be optimised further, the substrate scope may be poor. Bearing in mind that as most of these *ortho*-aryl-substituted ligands were particularly susceptible to competing formation of the corresponding phosphoramidites[4] and sensitivity to silica gel, leading to tedious purifications and very low yields, it was clear that this was not a promising scaffold for further optimisation.

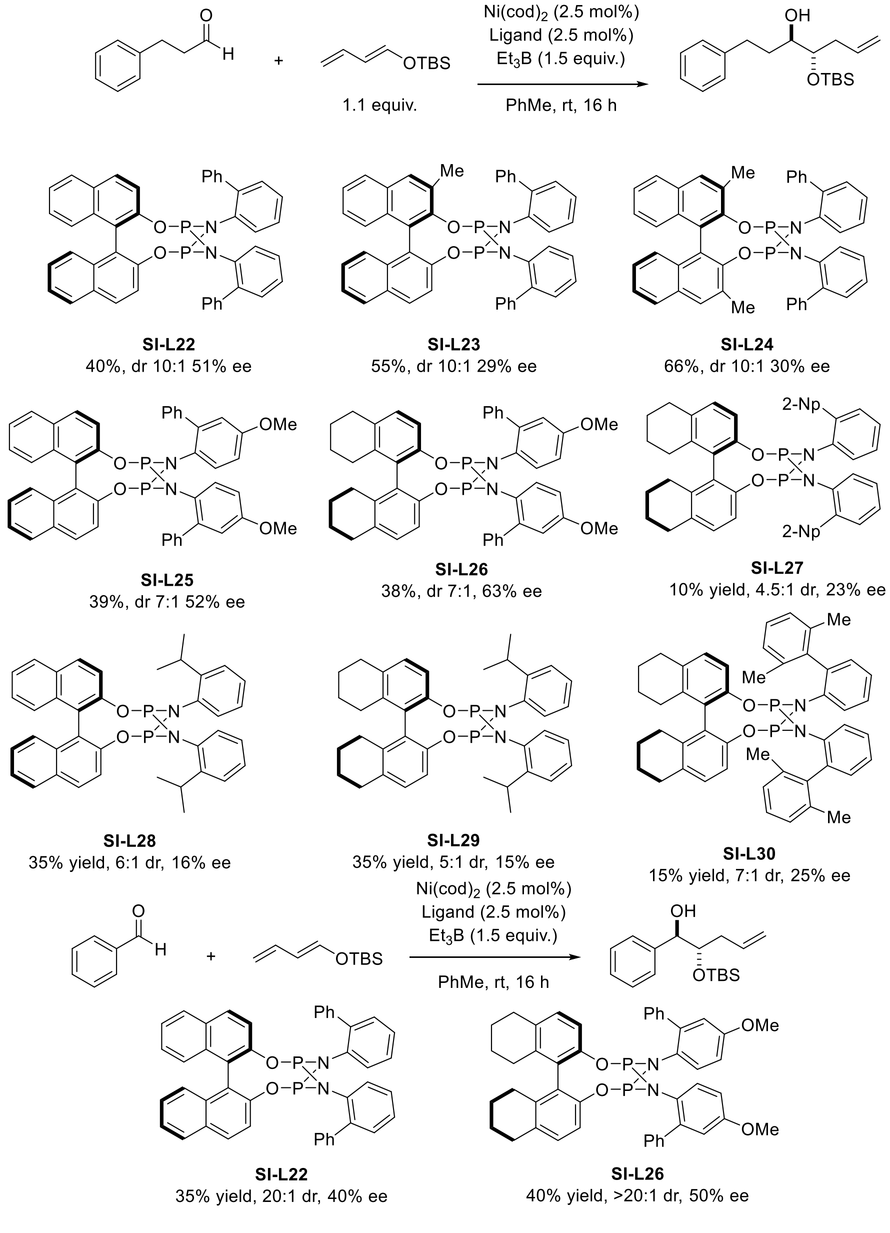

Scheme S4. *ortho*-Aryl-substituted cyclophosphazane ligands used in the third round of screening.

At this point, it seemed highly unlikely that further exploring cyclophosphazane ligands would lead to a useful enantioselective reaction. Considering the moderately promising result obtained with phosphoramidite **L2** (see manuscript) and the relative ease of preparing phosphoramidites with different chiral diol backbones (SPINOL, VANOL, VAPOL etc.), we decided to investigate these compounds instead. We focused on the NMe_2 derivatives as these can be conveniently synthesised from the diol and $P(NMe_2)_3$, allowing us to quickly test a range of diol scaffolds.

Phosphoramidites based on SPINOL, BINOL and H_8-BINOL, with and without substitution *ortho*- to the oxygen functionality, all showed poor reactivity and selectivity (**L10**-**L14**). However, we found that VAPOL-NMe_2-phosphoramidite **L16** promoted the reaction cleanly in 72% ee. Importantly, the reaction with benzaldehyde instead of hydrocinnamaldehyde using this ligand gave very similar results for this ligand.

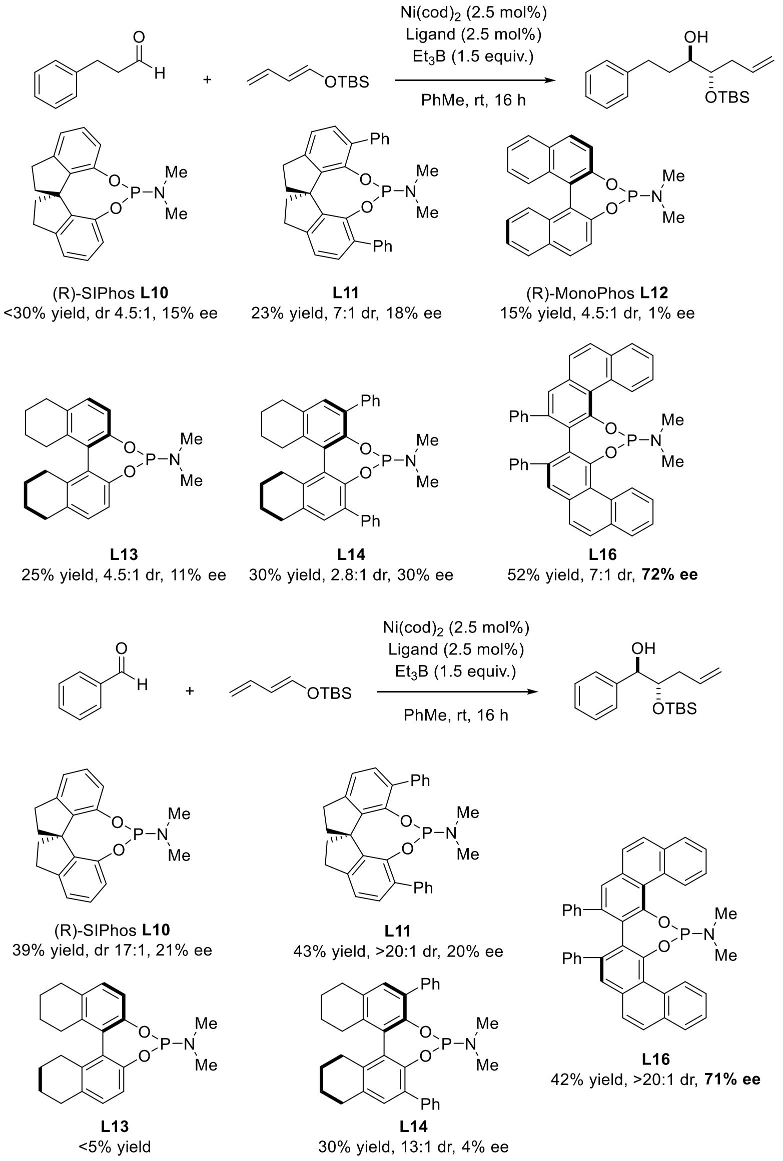

Scheme S5. Screening of phosphoramidite ligands.

Next, the effect of changing the substituent on nitrogen was explored. Moving to the *N*,*N*-diethyl analogue **L17** improved the dr and ee of the reaction. **L17** also proved to be more stable than the slightly air-sensitive dimethyl analogue **L16**. However, further increases in bulk (**SI-L32, SI-L34**), incorporation of a chiral amine (**SI-L35**) or unsymmetrical amine (**SI-L37**) did not lead to improved results, and either making the amine extremely bulky (**SI-L36**) or very flat (**SI-L38**) significantly eroded selectivity. Although the morpholine analogue **SI-L31** gave similar ee's, the diastereoselectivity was marginally worse, the ligand synthesis was lower yielding and it showed slight air instability similar to **L16**. For these reasons we opted to continue optimisation with VAPhos-NEt_2 **L17**.

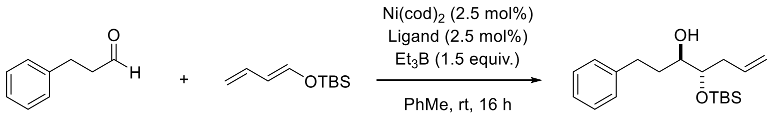

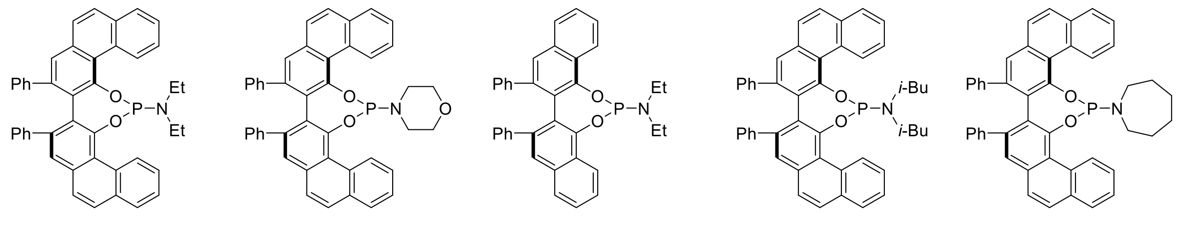

L17	SI-L31	L15	SI-L32	SI-L33
61% yield, 20:1 dr, 74% ee	59% yield, 16:1 dr, 75% ee	20% yield, 6:1 dr, 55% ee	62% yield, 20:1 dr, 71% ee	55% yield, 20:1 dr, 74% ee

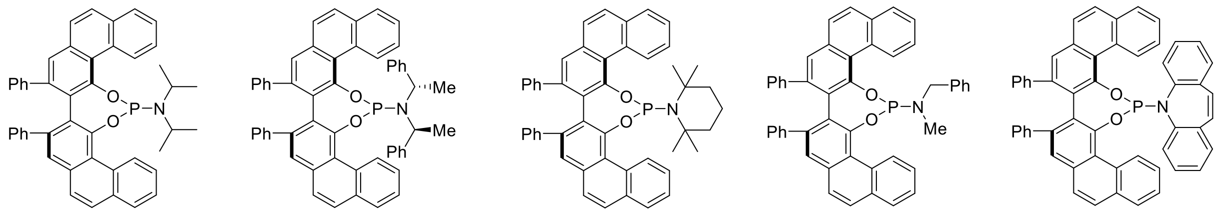

SI-L34	SI-L35	SI-L36	SI-L37	SI-L38
62% yield, >20:1 dr, 70% ee	60% yield, >20:1 dr, 73% ee	28% yield, >20:1 dr, 48% ee	63% yield, 18:1 dr, 74% ee	50% yield, 20:1 dr, 40% ee

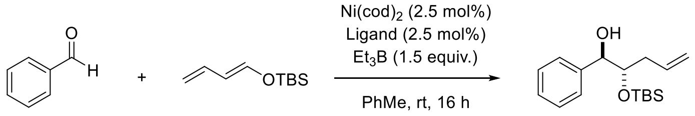

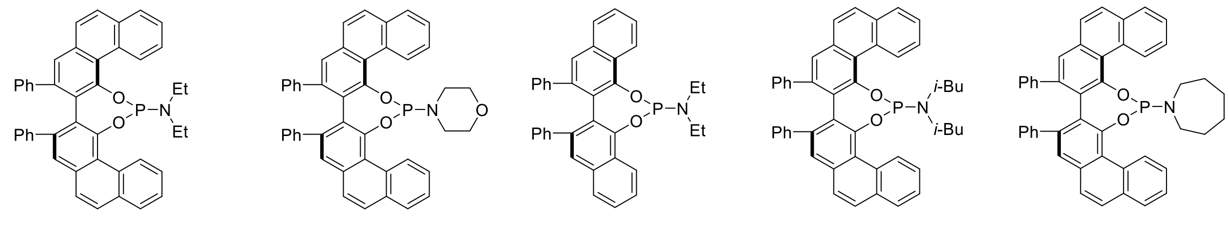

L17	SI-L31	L15	SI-L32	SI-L33
44% yield, >20:1 dr, 75% ee	51% yield, >20:1 dr, 78% ee	42% yield, >20:1 dr, 54% ee	37% yield, >20:1 dr, 53% ee	44% yield, >20:1 dr, 70% ee

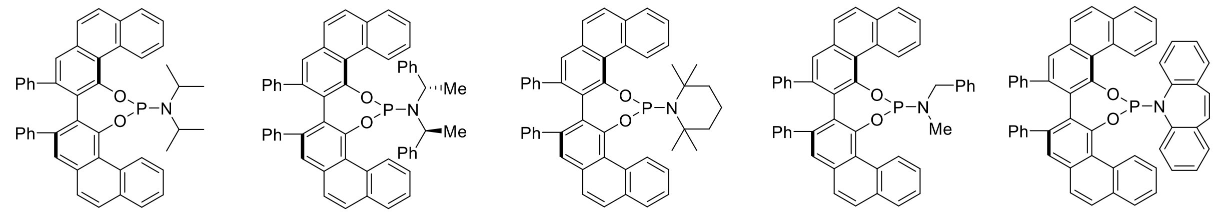

SI-L34	SI-L35	SI-L36	SI-L37	SI-L38
56% yield, >20:1 dr, 70% ee	66% yield, >20:1 dr, 67% ee	44% yield, >20:1 dr, 53% ee	60% yield, >20:1 dr, 65% ee	62% yield, >20:1 dr, 28% ee

Scheme S6. Screening of VAPOL phosphoramidite ligands with different amino substituents.

Non-polar solvents gave similar results as toluene, while more polar solvents like THF and DMF gave slightly lower enantioselectivity and poor reactivity respectively. Reducing the temperature gave a significant boost in ee but also lowered conversion, despite the use of longer reaction times in these experiments. The conversion could largely be recovered by using 3.0 equivalents of the diene instead of 1.1 equiv and increasing the catalyst loading to 10 mol% (see main manuscript). The excess diene seems to help stabilise the nickel catalyst over the course of the reaction.

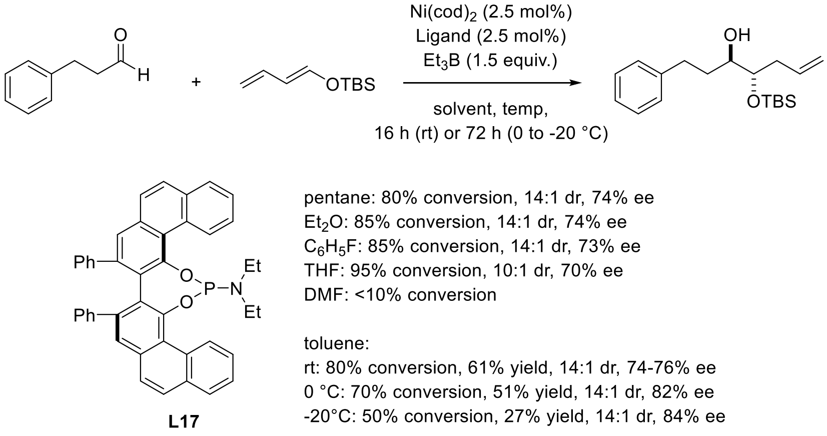

Scheme S7. Effect of solvent and temperature.

We were also encouraged that varying the temperature using a (*Z*)-configured diene also had a significant effect on enantioselectivity, even giving a high 90% ee with the standard hydrocinnamaldehyde substrate.

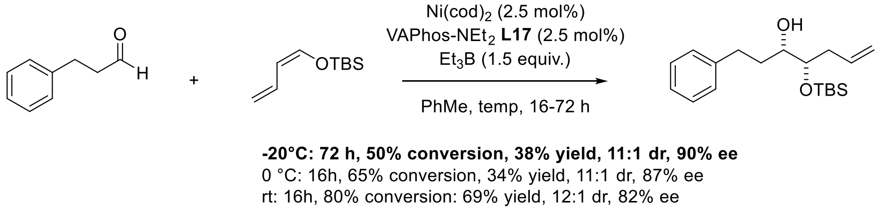

Scheme S8. Results with the (*Z*)-configured diene.

At this point we began to explore the scope using the excess of diene and 10 mol% catalyst loading. It is worth noting that raising the temperature to 0 °C allows for a lower catalyst loading to be used with only a small drop (2-3%) in ee, at least for the hydrocinnamaldehyde substrate.

1.3 Synthetic Procedures and Characterisation Data

1.3.1 Synthesis of Ligands

Most of the following cyclophosphazane ligands were prepared using a two-step procedure involving isolation of the intermediate dichlorocyclophosphazane after reaction of the amine with PCl_3 in the presence of triethylamine, followed by cyclisation with BINOL derivatives. However, difficulties involved in handling and purifying the air- and moisture-sensitive dichlorocyclophosphazane compounds led to often very low yields. The main problem is the necessity of distillation, sublimation or recrystallization to remove hydrochloride salts and side-products derived by over-addition of the amine to PCl_3 which can interfere with the BINOL cyclisation. An improved procedure which avoids isolation of the dichlorocyclophosphazane intermediate is detailed on page S24.

Representative procedure for synthesis of dichlorocyclophosphazanes.

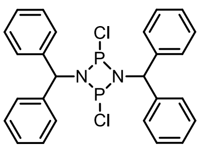

Dichlorocyclophosphazane SI-2. A solution of benzhydrylamine (4.25 g, 23.2 mmol) and triethylamine (32.0 mL, 232.0 mmol) in THF (20 mL) was added dropwise to a solution of phosphorus trichloride (2.0 mL, 23.2 mmol) in THF (15.0 mL) at −78 °C. The mixture was slowly warmed to room temperature overnight. The solvent was evaporated *in vacuo*. The solid material was dissolved in the minimum amount of boiling toluene and the solution gradually cooled to room temperature. The supernatant was removed by filtration, the residue was washed with a small amount of cold toluene and *n*-pentane and dried under high vacuum to yield the dichlorocyclophosphazane as a white solid (1.3 g, 23%). ^{1}H NMR (400 MHz, [D_8]-THF) δ 7.56-7.50 (m, 8H), 7.33-7.27 (m, 8H), 7.25-7.18 (m, H4), 5.71 (t, *J* = 6.20 Hz, 2H). ^{13}C NMR (100 MHz, [D_8]-THF) δ 140.2, 129.7, 129.3, 129.1, 64.4 (t, *J* = 6.9 Hz). ^{31}P NMR (161 MHz, [D_8]-THF) δ 223.8 (s). HRMS (ESI) m/z calcd for $C_{26}H_{22}N_2P_2Cl_2$: 494.0632 $[M]^+$, found 494.0635. Spectroscopic data matched those reported in the literature.[4]

Dichlorocyclophosphazane SI-3. Prepared according to the above procedure using

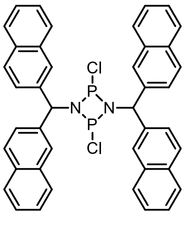

di(naphthalen-2-yl)methanamine (6.0 g, 21.0 mmol) and triethylamine (29.5 mL, 211.0 mmol) in THF (30.0 mL) and phosphorus trichloride (1.85 mL, 21.0 mmol) in THF (20.0 mL). White solid (0.7 g, 9.5%). ^{1}H NMR (300 MHz, CD_2Cl_2) δ 8.12 (br. s, 4H), 7.90-7.76 (m, 12H), 7.66 (dd, *J* = 8.5, 1.8 Hz, 4H), 7.54-7.43 (m, 8H), 6.07 (t, *J* = 6.3 Hz, 2H). ^{31}P NMR (121 MHz, CD_2Cl_2) δ 223.2 (s). HRMS (ESI) m/z calcd for $C_{42}H_{30}N_2P_2Cl_2$: 694.1279 $[M]^+$, found 694.1256. Spectroscopic data matched those reported in the literature.[4]

Cyclophosphazane L4. To a suspension of the dichlorocyclophosphazane **SI-2** (6.1 g, 12.34 mmol) in toluene (100 mL) was added (*S*)-BINOL (3.35 g, 12.36 mmol) and triethylamine (17.2 mL, 123.4 mmol). The milky suspension was heated to reflux for 16 hours. The reaction was then allowed to cool and filtered to remove the salts. The filtrate was concentrated to a pale yellow mass which was dissolved in toluene and filtered through a pad of silica gel, washing with 400 mL of toluene. The filtrate was concentrated to a colourless foam which was dissolved in minimal boiling ethyl acetate (40 mL). After cooling, the resulting colourless crystals were separated (4.91 g), and the mother liquor was concentrated and dissolved again in boiling ethyl acetate (20 mL) to crystallize a second crop (1.28 g). Colourless blocks (6.2 g, 71%). ^1H NMR (400 MHz, C_6D_6) δ 7.64 (dd, *J* = 8.3, 1.2 Hz, 2H), 7.52 (d, *J* = 8.8 Hz, 2H), 7.35 (d, *J* = 7.1 Hz, 2H), 7.26–7.09 (m, 14H), 7.04 (d, *J* = 8.8 Hz, 2H), 7.01–6.94 (m, 2H), 6.78–6.70 (m, 2H), 6.62 (t, *J* = 7.7 Hz, 4H), 6.49–6.42 (m, 4H), 5.13 (t, *J* = 5.7 Hz, 2H). ^{31}P NMR (162 MHz, C_6D_6) *δ* 174.4 ppm. Matches known data.[4]

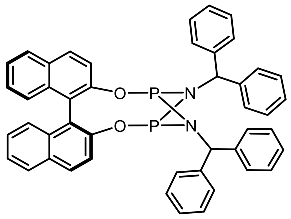

Representative procedure A for cyclophosphazane synthesis from isolated dichorophosphazanes. Cyclophosphazane L5. To a solution of the dichlorocyclophosphazane **SI-2** (63.5 mg, 0.13 mmol) in toluene (3.0 mL) was added a solution of the (*S*)-3,3-Ph_2BINOL (56.2 mg, 0.13 mmol) and triethylamine (0.18 mL, 1.3 mmol) in toluene (3.0 mL). The reaction mixture was stirred at 110 °C overnight. After cooling to room temperature, the mixture was filtered and the solvent was removed under reduced pressure. The residue was purified by flash chromatography over silica gel (hexane/CH_2Cl_2 = 90/10) to give **L5** as a white solid (58 mg, 53%). m.p. = 253-254 °C. *Rf* = 0.65 (hexane/CH_2Cl_2 = 60/40). ^1H NMR (400 MHz, CD_2Cl_2) δ 7.99-7.94 (m, 4H), 7.68-7.63 (m, 4H), 7.53 (ddd, *J* = 8.2, 6.8, 1.2 Hz, 2H), 7.41-7.29 (m, 8H), 7.22-7.12 (m, 8H), 6.97 (tt, *J* = 7.4, 1.3 Hz, 2H), 6.86-6.81 (m, 4H), 6.65-6.59 (m, 4H), 6.19-6.14 (m, 4H), 4.82 (t, *J* = 7.9 Hz, 2H). ^{13}C NMR (100 MHz, CD_2Cl_2) δ 150.9 (t, *J* = 7.3 Hz), 143.2, 139.3 (t, *J* = 4.2 Hz), 138.7, 135.5, 134.6, 131.4, 130.8, 130.5, 129.1, 128.7, 128.6, 128.5, 128.0 (br t, *J* = 1.4 Hz), 127.9, 127.7 (br s), 127.7, 127.5, 127.2, 126.2, 125.6, 63.4 (t, *J* = 14.8 Hz). ^{31}P NMR (161 MHz, CD_2Cl_2) δ 177.2 (s). IR (ATR): ṽ = 3058, 3026, 2964, 1620, 1599, 1493, 1451, 1415, 1354, 1331, 1297, 1262, 1243, 1188, 1184, 1147, 1136, 1091, 1070, 1028, 989, 962, 952, 935, 909, 894, 835, 807, 790, 739, 696, 647, 631, 615, 601, 568, 540, 521, 508, 457, 415. HRMS (ESI) m/z calcd for $C_{58}H_{43}N_2O_2P_2$: 861.2794 $[M+H]^+$, found 861.2800.

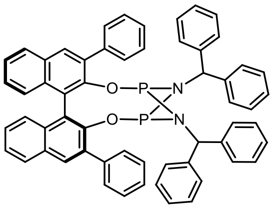

Cyclophosphazane L6. Prepared according to representative procedure A. Purified by flash

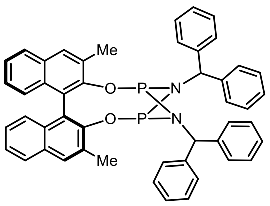

chromatography over silica gel (hexane/CH_2Cl_2 = 90/10) to give **L6** as a white solid (233 mg, 74%). m.p. = 271-277 °C. *Rf* = 0.60 (hexane/CH_2Cl_2 = 80/20). ^1H NMR (400 MHz, CD_2Cl_2) δ 7.89 (d, *J* = 8.2 Hz, 2H), 7.82 (s, 2H), 7.50-7.19 (m, 14H), 7.00-6.87 (m, 4H), 6.71 (t, *J* = 7.7 Hz, 4H), 6.20 (d, *J* = 7.7 Hz, 4H), 4.80 (t, *J* = 5.5 Hz, 2H), 2.34 (s, 6H). ^{13}C NMR (100 MHz, CD_2Cl_2) δ 152.6 (t, *J* = 6.8 Hz), 142.6, 138.7 (t, *J* = 4.3 Hz), 133.7, 131.22, 131.20, 129.8, 129.5, 129.0, 128.1, 127.9 (t, *J* = 3.5 Hz), 127.8, 127.6, 126.5 (br t, *J* = 1.7 Hz), 126.2, 126.0, 125.3, 63.8 (t, *J* = 13.2 Hz), 19.6. ^{31}P NMR (161 MHz, CD_2Cl_2) δ 174,3 (s). IR (ATR): ṽ = 3061, 3025, 1494, 1450, 1422, 1360, 1331, 1261, 1224, 1208, 1179, 1146, 1098, 1086, 1029, 991, 957, 934, 909, 886, 842, 807, 785, 757, 743, 733, 699, 630, 591, 555, 534, 510, 473, 451, 416. HRMS (ESI) m/z calcd for $C_{48}H_{39}N_2O_2P_2$: 737.2481 $[M+H]^+$, found 737.2483. $[\alpha]^{20}D$ = +437 ° (CH_2Cl_2, c =1.0).

Cyclophosphazane L8. Prepared according to representative procedure A. Purified by flash

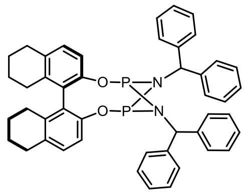

chromatography over silica gel (hexane/ethyl acetate = 90/10) to give **L8** as a white solid (90 mg, 64%). m.p. = 114-126 °C. *Rf* = 0.25 (hexane/ethyl acetate = 90/10). ^1H NMR (400 MHz, CD_2Cl_2) δ 7.45-7.25 (m, 10H), 7.16-7.08 (m, 6H), 7.06 (d, *J* = 8.2 Hz, 2H), 6.73 (d, *J* = 8.2 Hz, 2H), 6.58-6.50 (m, 4H), 4.89 (t, *J* = 4.9 Hz, 2H), 2.91-2.82 (m, 4H), 2.35-2.20 (m, 2H), 2.07-1.93 (m, 2H), 1.90-1.70 (m, 8H). ^{13}C NMR (100 MHz, CD_2Cl_2) δ 150.9 (t, *J* = 6.9 Hz), 142.7, 139.3 (t, *J* = 4.7 Hz), 137.4, 133.7, 132.2 (t, *J* = 1.7 Hz), 130.0, 129.9, 128.9 (br s), 128.2 (br s), 128.1 (t, *J* = 2.7 Hz), 128.0, 127.9, 121.1, 64.1 (t, *J* = 13.0 Hz), 30.1, 28.2, 23.9, 23.6. ^{31}P NMR (161 MHz, CD_2Cl_2) δ 169.3 (s). IR (ATR): ṽ = 3061, 3028, 2928, 2857, 2836, 1584, 1494, 1476, 1463, 1450, 1421, 1264, 1213, 1182, 1155, 1100, 1071, 1057, 1029, 1001, 959, 939, 909, 844, 832,816, 781, 740, 700, 670, 644, 542, 510. HRMS (ESI) m/z calcd for $C_{46}H_{43}N_2O_2P_2$: 717.2794 $[M+H]^+$, found 717.2797.

Cyclophosphazane L9. Prepared according to representative procedure A. Purified by flash

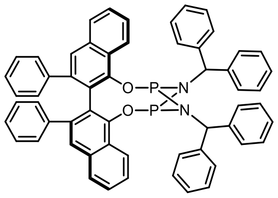

chromatography over silica gel (hexane/ethyl acetate = 90/10) to give **L9** as a white solid (88 mg, 36%). *Rf* = 0.60 (hexane/ethyl acetate = 70/30). m.p. = 238-239 °C. ^1H NMR (400 MHz, CD_2Cl_2) δ 7.99-7.94 (m, 2H), 7.78-7.73 (m, 2H), 7.54-7.45 (m, 4H), 7.42 (s, 2H), 7.40-7.31 (m, 6H), 7.12-6.93 (m, 20H), 6.33 (br d, *J* = 7.4 Hz, 4H), 4.24 (t, *J* = 5.5 Hz, 2H). ^{13}C NMR (100 MHz, CD_2Cl_2) δ 151.8, 141.97, 141.74, 141.0, 138.4 (t, *J* = 4.3 Hz),133.7, 129.4, 129.1, 128.5, 127.9, 127.8, 127.6, 127.4, 127.0, 126.9, 126.6, 126.5, 123.9, 122.9 (br s), 63.3 (t, *J* = 12.8 Hz), 53.8, 53.6, 53.4, 53.2, 53.0. ^{31}P NMR (161 MHz, CD_2Cl_2) δ 180.9 (s). IR (ATR): ṽ = 3059, 3027, 1591, 1563, 1488, 1450, 1357, 1329, 1288, 1268, 1188, 1107, 1074, 1055, 1028, 956,

899, 899, 884, 857, 838, 819, 792, 762, 753, 724, 696, 661, 637, 569, 507, 476, 419. HRMS (ESI) m/z calcd for $C_{58}H_{43}N_2O_2P_2$: 861.2793 $[M+H]^+$, found 861.2794. Spectroscopic data matched those reported in the literature.[4]

Dichlorophosphazane SI-4. A solution of *t*-butylamine (8.6 mL, 82.0 mmol, 1.5 equiv) in

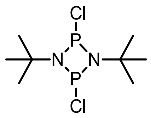

toluene (30.0 mL) was added dropwise to a well-stirred solution of phosphorus trichloride (4.8 mL, 55.0 mmol 1.0 equiv) and Et_3N (11.4 mL, 82.0 mmol, 1.5 equiv) in toluene (40.0 mL) at -78 °C. After the completion of the addition, the reaction mixture was allowed to r.t. and then stirred at reflux temperature for a further 4 h. The reaction mixture was then brought to room temperature and filtered to remove the amine hydrochloride. The filtrate was concentrated under reduced pressure to afford the product as a pasty white solid, which was then purified by vacuum distillation to give **SI-4** as a light yellow solid (5.3 g, 70.5%). ^1H NMR (400 MHz, C_6D_6) δ 1.18 (s, 18H). ^{13}C NMR (100 MHz, C_6D_6) δ 54.32 (t, J = 6.6 Hz), 30.24 (t, J = 6.3 Hz).^{31}P NMR (121 MHz, C_6D_6) δ 204.5 (s). HRMS (ESI) m/z calcd for $C_8H_{18}N_2P_2Cl_2$: 274.0324 $[M]^+$, found 274.0322. Spectroscopic data matched those reported in the literature.[4]

Cyclophosphazane L7. Prepared according to representative procedure A. Purified by flash

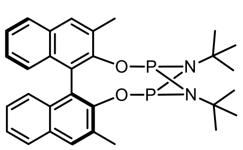

chromatographyover silica gel (hexane/CH_2Cl_2 = 90/10) to give **L7** as white solid (23 mg, 20%). m.p. = 266-267 °C. *Rf* = 0.85 (hexane/CH_2Cl_2 = 80/20). ^1H NMR (400 MHz, CD_2Cl_2) δ 7.77 (br. s, 2H), 7.75-7.72 (m, 2H), 7.27 (ddd, J = 8.1, 6.8, 1.2 Hz, 2H), 7.04 (ddd, J = 8.3, 6.8, 1.3 Hz, 2H), 6.53-6.49 (m, 2H), 2.55 (s, 6H), 0.86 (s, 18H). ^{13}C NMR (100 MHz, CD_2Cl_2) δ 151.7 (t, J = 6.9 Hz), 133.9, 131.7, 130.9, 129.3, 127.2, 126.9 (t, J = 2.7 Hz), 125.7, 124.7, 52.7 (t, J = 12.5 Hz), 30.7 (t, J = 6.5 Hz), 19.4. ^{31}P NMR (161 MHz, CD_2Cl_2) δ 169.5 (s). IR (ATR): ṽ = 3061, 2959, 2927, 2866, 1622, 1598, 1498, 1459, 1423, 1392, 1363, 1329, 1249, 1224, 1209, 1181, 1151, 1101, 1088, 1045, 1029, 1012, 989, 938, 927, 910, 894, 879, 782, 764, 751, 732, 629, 589, 475. HRMS (ESI) m/z calcd for $C_{30}H_{35}N_2O_2P_2$: 517.2168 $[M+H]^+$, found 517.2173. Spectroscopic data matched those reported in the literature.[4]

Cyclophosphazane SI-L1. Prepared according to representative procedure A. Purified by

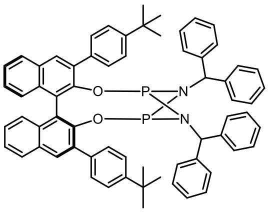

flash chromatography over silica gel (hexane/CH_2Cl_2 = 90/10) to give **SI-L1** as white solid (49 mg, 53%). m.p. = 208-209 °C. *Rf* = 0.68 (hexane/CH_2Cl_2 = 70/30). ^1H NMR (400 MHz, CD_2Cl_2) δ 8.02 (s, 2H), 7.98-7.93 (m, 2H), 7.67-7.62 (m, 4H), 7.52 (ddd, J = 8.1, 6.8, 1.2 Hz, 2H), 7.44-7.39 (m, 4H), 7.35 (ddd, J = 8.3, 6.8, 1.3 Hz, 2H), 7.22-7.12 (m, 8H), 6.98-6.92 (m, 2H), 6.85-6.80 (m, 4H), 6.66-6.60 (m, 4H), 6.18-6.12 (m, 4H), 4.85 (t, J = 7.3 Hz, 2H), 1.33 (s, 18H). ^{13}C NMR (100 MHz, CD_2Cl_2) δ 151.2 (t, J = 7.2 Hz), 151.2 , 143.1, 139.3 (t, J = 4.4 Hz), 135.7, 135.2, 134.4, 131.4, 130.5, 130.5, 129.2, 128.6, 128.4, 128.0 (br s), 127.9, 127.9 (t, J

= 2.9 Hz), 127.7, 127.5, 127.0, 126.2, 125.7, 125.5, 63.1 (t, *J* = 14.6 Hz), 34.9, 31.5. ^{31}P NMR (161 MHz, CD_2Cl_2) δ 175.9 (s). IR (ATR): ṽ = 3058, 3027, 2961, 2866, 1596, 1512, 1493, 1449, 1424, 1395, 1360, 1262, 1254, 1189, 1186, 1140, 1092, 1070, 1026, 992, 963, 952, 935, 909, 884, 849, 829, 806, 789, 746, 689, 660, 639, 622, 606, 593, 569, 531, 514, 408. HRMS (ESI) m/z calcd for $C_{66}H_{59}N_2O_2P_2$: 973.4057 [M+H]$^+$, found 973.4046.

Cyclophosphazane SI-L2. Prepared according to representative procedure A. Purified by

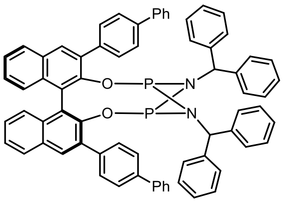

flash chromatography over silica gel (hexane/CH_2Cl_2 = 90/10) to give **SI-L2** as white solid (49 mg, 53%). m.p. = 183-184 °C. *Rf* = 0.75 (hexane/CH_2Cl_2 = 70/30). ^{1}H NMR (400 MHz, CD_2Cl_2) δ 8.02 (s, 2H), 8.00-7.96 (m, 2H), 7.78-7.73 (m, 4H), 7.66-7.61 (m, 8H), 7.54 (ddd, *J* = 8.1, 6.8, 1.2 Hz, 2H), 7.48-7.42 (m, 4H), 7.41-7.33 (m, 4H), 7.22-7.11 (m, 8H), 7.00-6.94 (m, 2H), 6.89-6.84 (m, 4H), 6.68-6.62 (m, 4H), 6.24-6.19 (m, 4H), 4.87 (t, *J* = 7.8 Hz, 2H). ^{13}C NMR (100 MHz, CD_2Cl_2) δ 151.0 (t, *J* = 7.1 Hz), 143.2, 140.8, 140.6, 139.3 (t, *J* = 4.0 Hz), 137.8, 135.1, 134.6, 131.4, 131.2, 130.5, 129.2, 129.2, 128.7, 128.6, 128.1 (br s), 127.9, 127.8, 127.8, 127.7, 127.5, 127.4, 127.2, 126.2, 125.6, 63.6 (t, *J* = 14.7 Hz). ^{31}P NMR (161 MHz, CD_2Cl_2) δ 177.1 (s). IR (ATR): ṽ = 3057, 3026, 2962, 2922, 1599, 1487, 1450, 1422, 1395, 1262, 1243, 1188, 1178, 1148, 1136, 1092, 1070, 1028, 1007, 962, 951, 935, 909, 884, 837, 809, 789, 762, 749, 738, 695, 677, 638, 624, 602, 573, 536, 515, 415. HRMS (ESI) m/z calcd for $C_{70}H_{51}N_2O_2P_2$: 1013.3411 [M+H]$^+$, found 1013.3420.

Cyclophosphazane SI-L3. Prepared according to representative procedure A. Purified by

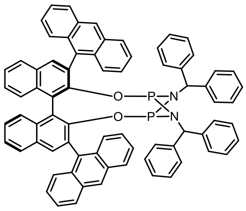

flash chromatography over silica gel (hexane/CH_2Cl_2 = 95/5) to give **SI-L3** as a light orange solid (130 mg, 52%). m.p. = 214-227 °C. *Rf* = 0.80 (hexane/CH_2Cl_2 = 60/40). ^{1}H NMR (400 MHz, CD_2Cl_2) δ δ 8.52 (s, 2H), 8.18 (s, 2H), 8.10-7.99 (m, 10H), 7.66-7.58 (m, 4H), 7.48-7.34 (m, 8H), 7.20 (ddd, *J* = 8.9, 6.6, 1.3 Hz, 2H), 6.98-6.93 (m, 2H), 6.91-6.86 (m, 2H), 6.84-6.78 (m, 4H), 6.51-6.45 (m, 4H), 6.27-6.21 (m, 8H), 4.85 (t, *J* = 10.0 Hz, 2H). ^{13}C NMR (100 MHz, CD_2Cl_2) δ 151.7 (t, *J* = 7.8 Hz), 143.2, 139.8 (t, *J* = 4.2 Hz), 135.9, 133.9, 132.9, 132.2, 131.9, 131.6, 131.2, 130.9, 130.1, 129.6, 128.8, 128.7, 128.5, 128.3, 128.2, 128.1, 128.0, 127.7, 127.51, 127.46, 127.4, 127.4, 127.3, 126.5, 126.3, 126.1, 125.7, 125.7, 125.6, 125.1, 63.1 (t, *J* = 15.7 Hz). ^{31}P NMR (161 MHz, CD_2Cl_2) δ 173.1 (s). IR (ATR): ṽ = 3051, 3025, 1492, 1444, 1425, 1402, 1316, 1203, 1181, 1145, 1099, 1086, 1068, 1026, 943, 905, 886, 864, 841, 785, 736, 729, 695, 641, 606, 516, 427. HRMS (ESI) m/z calcd for $C_{74}H_{51}N_2O_2P_2$: 1061.3420 [M+H]$^+$, found 1061.3428.

Cyclophosphazane SI-L4. Prepared according to representative procedure A. Purified by flash chromatography over silica gel (hexane/CH_2Cl_2 = 80/20) to give **SI-L4** as white solid (63 mg, 62%). m.p. = 231-232 °C. *Rf* = 0.70 (hexane/CH_2Cl_2 = 50/50). ^{1}H NMR (400 MHz, CD_2Cl_2) δ 7.93-7.88 (m, 2H), 7.78 (s, 2H), 7.47-7.31 (m, 8H), 7.27-7.21 (m, 6H), 6.97-6.88 (m, 4H), 6.71-6.64 (m, 4H), 6.20-6.15 (m, 4H), 4.73 (t, *J* = 5.5 Hz, 2H), 2.80 (dq, *J* = 15.0, 7.4 Hz, 2H), 2.51 (dq, *J* = 15.0, 7.4 Hz, 2H), 1.33 (t, *J* = 7.4 Hz, 6H). ^{13}C NMR (100 MHz, CD_2Cl_2) δ 152.4 (t, *J* = 6.7 Hz), 142.6, 138.7 (t, *J* = 4.3 Hz), 136.6, 133.6, 131.3, 129.5, 129.0, 128.0, 127.8, 127.8, 127.7, 127.3, 126.5 (br t, *J* = 1.50 Hz), 126.2, 125.9, 125.2, 63.9 (t, *J* = 13.1 Hz), 25.9 (br t, *J* = 1.9 Hz), 13.3. ^{31}P NMR (161 MHz, CD_2Cl_2) δ 174,0 (s). IR (ATR): ṽ = 3059, 3025, 2963, 2931, 2873, 1621, 1598, 1493, 1451, 1422, 1374, 1359, 1337, 1207, 1177, 1146, 1097, 1070, 1027, 953, 933, 910, 886, 865, 811, 797, 744, 701, 627, 595, 535, 511, 411. HRMS (ESI) m/z calcd for $C_{50}H_{42}N_2O_2P_2Na$: 787.2619 [M+Na]$^+$, found 787.2613.

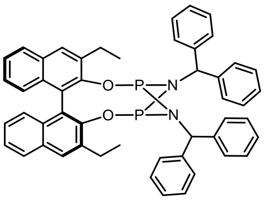

Cyclophosphazane SI-L5. Prepared according to representative procedure A. Purified by flash chromatography over silica gel (hexane/CH_2Cl_2 = 95/5) to give **SI-L5** as a white powder (66 mg, 55%). *Rf* = 0.60 (hexane/CH_2Cl_2 = 70/30). m.p. = 194-195 °C. ^{1}H NMR (400 MHz, CD_2Cl_2) δ 7.96 (s, 2H), 7.89-7.85 (m, 2H), 7.75-7.72 (m, 2H), 7.69-7.65 (m, 4H), 7.64-7.60 (m, 2H), 7.55 (ddd, *J* = 8.1, 6.8, 1.3 Hz, 2H), 7.49-7.44 (m, 4H), 7.42-7.26 (m, 16H), 7.24-7.16 (m, 6H), 6.94 (dd, *J* = 8.5, 1.9 Hz, 2H), 6.75 (br. s, 2H), 6.69-6.65 (m, 2H), 6.40 (dd, *J* = 8.6, 1.9 Hz, 2H), 5.15 (t, *J* = 8.2 Hz, 2H). ^{13}C NMR (100 MHz, CD_2Cl_2) δ 151.4 (t, *J* = 7.2 Hz), 140.8, 138.7, 136.82, 136.8 (t, *J* = 4.0 Hz), 135.6, 134.7, 133.4, 133.2, 133.1, 132.7, 131.5, 130.9, 130.6, 128.8, 128.7, 128.6, 128.5, 128.4, 128.3, 128.2, 128.1, 127.9, 127.5, 127.4, 127.0, 126.5, 126.6, 126.4, 126.2, 126.0, 126.0, 125.9, 125.8, 63.8 (t, *J* = 14.9 Hz). ^{31}P NMR (161 MHz, CD_2Cl_2) δ 179.2 (s). IR (ATR): ṽ = 3053, 2963, 2925, 1621, 1599, 1506, 1495, 1455, 1415, 1356, 1330, 1263, 1242, 1209, 1187, 1148, 1136, 1123, 1078, 1031, 990, 962, 944, 933, 893, 858, 835, 819, 789, 745, 740, 701, 648, 633, 614, 601, 569, 549, 520, 476, 458, 424. HRMS (ESI) m/z calcd for $C_{74}H_{51}N_2O_2P_2$: 1061.3420 [M+H]$^+$, found 1061.3439.

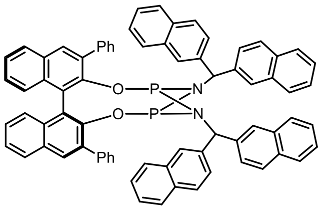

Cyclophosphazane SI-L6. Prepared according to representative procedure A. Purified by flash chromatography over silica gel (hexane/CH_2Cl_2 = 95/5) to give **SI-L6** as a yellow powder (77 mg, 72%). m.p. = 202-203 °C. *Rf* = 0.65 (hexane/CH_2Cl_2 = 70/30). ^{1}H NMR (400 MHz, CD_2Cl_2) δ 7.99 (s, 2H), 7.89 (d, *J* = 7.8 Hz, 2H), 7.74 (d, *J* = 8.0 Hz, 2H), 7.66-7.59 (m, 6H), 7.55-7.50 (m, 2H), 7.49-7.43 (m, 4H), 7.40-7.31 (m, 12H), 7.31-7.27 (m, 2H), 7.22-7.16 (m, 6H), 6.93 (dd, *J* = 8.5, 1.8 Hz, 2H), 6.73 (br. s, 2H), 6.67 (d, *J* = 8.2 Hz, 2H), 6.37 (dd, *J* = 8.6, 1.9 Hz, 2H), 5.16 (t, *J* = 7.8 Hz, 2H), 1.28 (s,

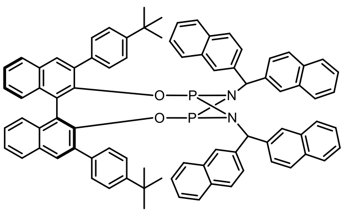

18H). ^{13}C NMR (100 MHz, CD_2Cl_2) δ 151.6 (t, *J* = 7.2 Hz), 151.2, 140.7, 136.9 (t, *J* = 4.2 Hz), 135.7, 135.4, 134.6, 133.4, 133.2, 133.1, 132.7, 131.5, 130.5, 128.7, 128.6, 128.4, 128.3, 128.2, 127.9, 127.5, 127.4, 127.3, 127.1, 126.7 (br t, *J* = 2.2 Hz), 126.5, 126.5, 126.3, 126.2, 125.9, 125.9, 125.8, 125.7, 63.6 (t, *J* = 14.8 Hz), 34.8, 31.5. ^{31}P NMR (161 MHz, CD_2Cl_2) δ 178.5 (s). IR (ATR): ṽ = 3053, 2961, 2902, 2866, 1599, 1508, 1449, 1423, 1395, 1360, 1329, 1263, 1243, 1187, 1140, 1122, 1080, 1019, 992, 963, 944, 933, 893, 886, 857, 829, 818, 789, 748, 742, 700, 660, 644, 620, 587, 570, 531, 476, 434. HRMS (ESI) m/z calcd for $C_{82}H_{67}N_2O_2P_2$: 1173.4672 $[M+H]^+$, found 1173.4669.

Cyclophosphazane SI-L7. Prepared according to representative procedure A. Purified by flash chromatography over silica gel (hexane/CH_2Cl_2 = 95/5) to give **SI-L7** as white solid (46 mg, 46%). m.p. = 207-208 °C. *Rf* = 0.70 (hexane/CH_2Cl_2 = 70/30). ^{1}H NMR (400 MHz, CD_2Cl_2) δ 8.02 (s, 2H), 7.90 (br d, *J* = 7.8 Hz, 2H), 7.79-7.75 (m, 4H), 7.72 (br d, *J* = 7.9 Hz, 2H), 7.65 (br d, *J* = 8.3 Hz, 2H), 7.61-7.17 (m, 36H), 6.97 (dd, *J* = 8.5, 1.9 Hz, 2H), 6.79 (br. s, 2H), 6.71 (dd, *J* = 8.3, 1.1 Hz, 2H), 6.44 (dd, *J* = 8.6, 1.9 Hz, 2H), 5.19 (t, *J* = 8.1 Hz, 2H). ^{13}C NMR (100 MHz, CD_2Cl_2) δ 151.5 (t, *J* = 7.2 Hz), 140.7, 140.7, 140.6, 137.7, 136.8 (t, *J* = 4.1 Hz), 135.1, 134.7, 133.4, 133.2, 133.13, 132.7, 131.5, 131.3, 130.5, 129.1, 128.8, 128.6, 128.5, 128.5, 128.2, 127.9, 127.8, 127.5, 127.5, 127.3, 127.3, 127.0, 126.6 (br t, *J* = 2.4 Hz), 126.5, 126.5, 126.3, 126.2, 126.0, 125.9 (t, *J* = 3.7 Hz), 25.8, 63.8 (t, *J* = 14.8 Hz). ^{31}P NMR (161 MHz, CD_2Cl_2) δ 179.4 (s). IR (ATR): ṽ = 3053, 3027, 1599, 1506, 1487, 1451, 1422, 1395, 1358, 1329, 1263, 1243, 1187, 1148, 1137, 1123, 1078, 1007, 990, 963, 944, 933, 886, 857, 837, 818, 789, 751, 736, 696, 676, 639, 622, 601, 573, 514, 476, 432, 426. HRMS (ESI) m/z calcd for $C_{86}H_{59}N_2O_2P_2$: 1213.4046 $[M+H]^+$, found 1213.4055.

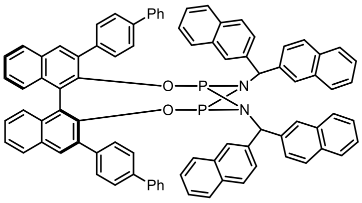

Cyclophosphazane SI-L8. Prepared according to representative procedure A. Purified by flash chromatography over silica gel (hexane/CH_2Cl_2 = 95/5) to give **SI-L8** as a white powder (78 mg, 83%). m.p. = 190-200 °C. *Rf* = 0.30 (hexane/CH_2Cl_2 = 70/30). ^{1}H NMR (400 MHz, CD_2Cl_2) δ 7.94-7.90 (m, 2H), 7.89-7.83 (m, 4H), 7.82-7.77 (m, 4H), 7.75-7.70 (m, 2H), 7.59-7.47 (m, 8H), 7.45 (dd, *J* = 8.5, 1.9 Hz, 2H), 7.34 (dddd, *J* = 8.1, 6.8, 2.7, 1.3 Hz, 4H), 7.23-7.16 (m, 4H), 7.00 (br d, *J* = 8.5 Hz, 2H), 6.78 (br d, *J* = 8.3 Hz, 2H), 6.60 (br. s, 2H), 6.45 (dd, *J* = 8.6, 1.8 Hz, 2H), 5.22 (t, *J* = 5.9 Hz, 2H), 2.37 (s, 3H), 2.37 (s, 3H). ^{13}C NMR (100 MHz, CD_2Cl_2) δ 152.8 (t, *J* = 6.8 Hz), 140.3, 136.0 (t, *J* = 4.1 Hz), 133.9, 133.8, 133.4, 133.1, 132.8, 131.4, 131.3, 129.8, 129.0, 128.5, 128.4, 128.1, 127.8, 127.57, 127.54, 127.1, 126.8, 126.7 (br t, *J* = 2.4 Hz), 126.6, 126.4, 126.3, 126.13, 126.09 (br s), 125.5, 64.1 (t, *J* = 13.4 Hz), 19.7. ^{31}P NMR (161 MHz, CD_2Cl_2) δ 175.7 (s). IR (ATR): ṽ = 3050, 3023, 1630, 1598, 1505, 1460, 1422, 1359, 1331, 1270, 1224, 1206, 1177, 1161, 1145, 1122, 1097, 1085, 1033, 977, 945, 931, 909, 893, 856, 816, 783, 747, 728, 649,

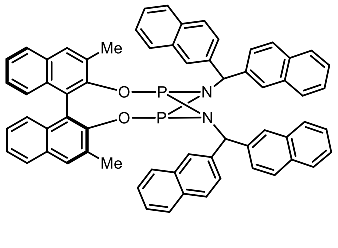

631, 621, 605, 592, 533, 474. HRMS (ESI) m/z calcd for $C_{64}H_{47}N_2O_2P_2$: 937.3115 $[M+H]^+$, found 937.3107.

Cyclophosphazane SI-L9. Prepared according to representative procedure A. Purified by

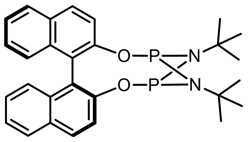

flash chromatography over silica gel (hexane/CH_2Cl_2 = 90/10) to give **SI-L9** as a yellow solid (101 mg, 57%). m.p. = 206-207 °C. *Rf* = 0.75 (hexane/CH_2Cl_2 = 80/20). ^{1}H NMR (400 MHz, CD_2Cl_2) δ 7.93-7.89 (m, 2H), 7.86-7.82 (m, 2H), 7.33 (ddd, *J* = 8.0, 6.7, 1.5 Hz, 2H), 7.33-7.29 (m, 2H), 7.13 (ddd, *J* = 8.3, 6.8, 1.4 Hz, 2H), 6.65-6.60 (m, 2H), 0.89 (s, 18H). ^{13}C NMR (100 MHz, CD_2Cl_2) δ 152.1 (t, *J* = 7.1 Hz), 134.9, 130.9, 129.3, 128.0, 126.7, 126.7 (t, *J* = 2.4 Hz), 126.1, 125.6, 124.9, 52.7 (t, *J* = 12.5 Hz), 30.9 (t, *J* = 6.5 Hz). ^{31}P NMR (161 MHz, CD_2Cl_2) δ 172.4 (s). IR (ATR): ṽ = 3061, 2960, 2926, 2901, 2864, 1618, 1595, 1504, 1470, 1426, 1392, 1364, 1323, 1261, 1247, 1202, 1153, 1142, 1071, 1045, 1013, 972, 950, 883, 868, 826, 783, 751, 680, 653, 627, 617, 590, 578, 536, 506, 471, 456. HRMS (ESI) m/z calcd for $C_{28}H_{31}N_2O_2P_2$: 489.1855 $[M+H]^+$, found 489.1856. Spectroscopic data matched those reported in the literature.[4]

Cyclophosphazane SI-L10. Prepared according to representative procedure A. Purified by

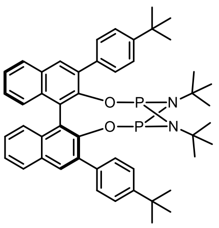

flash chromatography over silica gel (hexane/CH_2Cl_2 = 95/5) to give **SI-L10** as a yellow powder (38 mg, 31%). m.p. = 173-178 °C. *Rf* = 0.8 (hexane/CH_2Cl_2 = 80/20). ^{1}H NMR (400 MHz, CD_2Cl_2) δ 8.01 (br. s, 2H), 7.89-7.85 (m, 2H), 7.84-7.79 (m, 4H), 7.53-7.48 (m, 4H), 7.35 (ddd, *J* = 8.1, 6.8, 1.2 Hz, 2H), 7.17 (ddd, *J* = 8.3, 6.8, 1.3 Hz, 2H), 6.82-6.77 (m, 2H), 1.40 (s, 18H), 0.75 (s, 18H). ^{13}C NMR (100 MHz, CD_2Cl_2) δ 150.7, 149.7 (t, *J* = 7.1 Hz), 136.4, 135.9, 134.2, 131.1, 130.5, 130.0, 128.5 (t, *J* = 2.7 Hz), 128.2, 126.7, 125.6, 125.4, 125.1, 52.6 (t, *J* = 12.5 Hz), 34.9, 31.6, 30.9 (t, *J* = 6.6 Hz). ^{31}P NMR (161 MHz, CD_2Cl_2) δ 169.3 (s). IR (ATR): ṽ = 3052, 2962, 2903, 2866, 1619, 1594, 1514, 1461, 1449, 1424, 1393, 1365, 1261, 1193, 1139, 1089, 1075, 1042, 1042, 1013, 964, 937, 890, 849, 828, 799, 790, 761, 729, 700, 661, 634, 615, 589, 545, 526. HRMS (ESI) m/z calcd for $C_{48}H_{55}N_2O_2P_2$: 753.3733 $[M+H]^+$, found 753.3742.

Dichlorophosphazane SI-5. A solution of cyclohexylamine (11.8 mL, 103.0 mmol, 3.0 equiv)

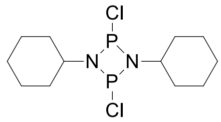

in toluene (10.0 mL) was added dropwise to a well-stirred solution of phosphorus trichloride (3.0 mL, 34.4 mmol 1.0 equiv) in toluene (20.0 mL) at -78 °C. After the completion of the addition, the reaction mixture was stirred at this temperature for 4 h and then heated under reflux for a further 4 h. The reaction mixture was then brought to room temperature and filtered to remove the amine hydrochloride. The filtrate was concentrated under reduced pressure to afford the product as a pasty white solid, which was then purified by vacuum distillation. The minor product, $PCl_2(NHCy)$, was distilled out at 70 °C (0.3 Torr), whereas the expected product distilled out at 128 °C (0.3 Torr)

to give **SI-5** as white solid (0.7 g, 9.5%). ^{1}H NMR (300 MHz, CD_2Cl_2) δ 1.10-2.16 (m, 22H). ^{13}C NMR (75 MHz, CD_2Cl_2) δ 53.5 (t, *J* = 5.5 Hz), 33.4 (t, *J* = 5.9 Hz), 25.6, 24.9. ^{31}P NMR (121 MHz, CD_2Cl_2) δ 219.9 (s). HRMS (ESI) m/z calcd for $C_{12}H_{22}N_2P_2Cl_2$: 326.0632 $[M]^+$, found 326.0635. Spectroscopic data matched those reported in the literature.[4]

Cyclophosphazane SI-L12. Prepared according to representative procedure A. Purified by

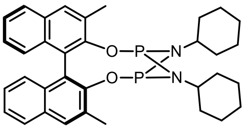

crystallization in 90/10 hexane/CH_2Cl_2 to give **SI-L12** as a white solid (68 mg, 61%). m.p. = 161-162 °C. *Rf* = 0.80 (hexane/CH_2Cl_2 = 80/20). ^{1}H NMR (400 MHz, CD_2Cl_2) δ 7.80-7.75 (m, 4H), 7.32 (ddd, *J* = 8.2, 6.8, 1.2 Hz, 2H), 7.12 (ddd, *J* = 8.2, 6.8, 1.3 Hz, 2H), 6.77-6.71 (m, 2H), 2.54 (s, 3H), 2.54 (s, 3H), 2.24 (ddt, *J* = 11.1, 7.4, 3.7 Hz, 2H), 1.93-1.82 (m, 2H), 1.68--1.59 (m, 2H), 1.48-1.39 (m, 2H), 1.38-1.01 (m, 8H), 1.00-0.70 (m, 6H). ^{13}C NMR (100 MHz, CD_2Cl_2) δ 152.3 (t, *J* = 6.5 Hz), 133.3, 131.1, 130.9, 129.1, 127.3, 126.6 (t, *J* = 2.3 Hz), 125.8, 125.7, 124.9, 54.6 (t, *J* = 11.1 Hz), 37.6 (t, *J* = 2.5 Hz), 32.9 (t, *J* = 7.3 Hz), 25.8, 25.7, 25.7, 19.5 (t, *J* = 1.9 Hz). ^{31}P NMR (161 MHz, CD_2Cl_2) δ 173.0 (s). IR (ATR): ṽ = 2923, 2852, 1623, 1599, 1486, 1461, 1447, 1424, 1372, 1361, 1332, 1261, 1222, 1208, 1178, 1135, 1097, 1085, 1033, 992, 939, 909, 888, 863, 843, 783, 750, 735, 703, 631, 607, 590, 536, 486, 432. HRMS (ES) m/z calcd for $C_{34}H_{38}N_2O_2P_2$: 568.2407 [M], found 568.2408.

Cyclophosphazane SI-L13. To a solution of cyclophosphazane **L4** (216 mg, 0.3 mmol) in THF

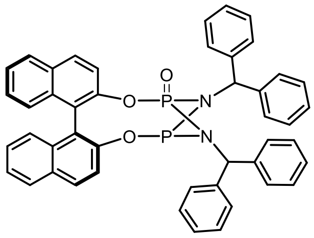

(2 mL) was added diisopropylazodicarboxylate (60 µL, 0.3 mmol). The reaction stirred at RT for 16 hours at which point the yellow colour of the DIAD had diminished. The reaction was concentrated to a yellow residue which was purified by flash chromatography (hexane/ethyl acetate 95:5 to 9:1) to give the product as a colourless foam (215 mg, 97%). $[\alpha]_D^{20}$ = +238.8 (c = 1.03, $CHCl_3$). ^{1}H NMR (400 MHz, C_6D_6) δ 7.64 (dd, *J* = 8.3, 1.2 Hz, 1H), 7.56–7.52 (m, 3H), 7.51–7.47 (m, 1H), 7.28–7.19 (m, 5H), 7.12–7.04 (m, 5H), 7.03–6.87 (m, 8H), 6.85–6.78 (m, 1H), 6.76 (d, *J* = 4.3 Hz, 4H), 6.69 (p, *J* = 4.2 Hz, 1H), 6.55 (d, *J* = 4.4 Hz, 4H), 5.55 (dd, *J* = 15.9, 4.1 Hz, 1H), 5.22 (dd, *J* = 9.9, 5.8 Hz, 1H). ^{13}C NMR (101 MHz, C6D6) δ 150.3, 150.2, 141.5, 141.4, 139.0, 134.8, 134.7, 131.2, 131.1, 130.5, 129.6, 128.7, 128.7, 128.4, 128.3, 128.3, 128.3, 128.0, 127.8, 127.6, 127.5, 127.3, 127.1, 127.0, 126.2, 125.3, 125.2, 124.7, 124.6, 124.1, 124.0, 122.6, 122.6, 63.8 (*J* = 12.4 Hz), 62.8 (d, *J* = 11.1 Hz). ^{31}P NMR (162 MHz, C6D6) *δ* 100.3 (d, *J* = 6.3 Hz), 6.8 (d, *J* = 6.0 Hz). IR (ATR): ṽ = 3062, 1620, 1596, 1495, 1471, 1453, 1428, 1359, 1325, 1279, 1210, 1153, 1097, 1073, 1029, 998, 944, 910, 845, 822, 749, 699, 634, 587, 518. HRMS (ESI$^+$) m/z calcd. for C46H35N2O2P2: 725.2117, found: 725.2115.

Dichlorophosphazane SI-6. To a solution of PCl_3 (2.52 mL, 28.9 mmol) in THF (50 mL) was added dropwise a solution of (*R*)-(+)-1-(2-naphthyl)ethylamine (4.94 g, 28.8 mmol) and triethylamine (40 mL, 287 mmol) in THF (50 mL) while maintaining an internal temp of –70 °C. After complete addition (ca. 2.5 hours) the reaction mixture was allowed to warm to rt overnight. The reaction mixture was filtered under argon, washing the solids with an additional portion of THF (2 x 50 mL). The filtrate was concentrated to a pale yellow foam. The foam was dissolved in hot toluene (20 mL) and when cooled was filtered again under argon to remove any residual salts, washing with additional toluene (2 x 10 mL). The toluene was removed *in vacuo* at rt until the product had fully precipitated from the mixture and around 10-15 mL of toluene remained. The suspension was then heated until complete dissolution occurred and was then allowed to cool back to rt. The precipitated crystals were isolated by filtration under argon and washed with a small amount of cold toluene and then pentane to give the product as white needles (2.15 g, 32%). ^1H NMR (400 MHz, THF) *δ* 7.92–7.86 (m, 3H), 7.86–7.78 (m, 5H), 7.56 (dd, *J* = 8.5, 1.8 Hz, 2H), 7.49–7.40 (m, 4H), 4.78 (h, *J* = 6.5 Hz, 2H), 1.69–1.59 (m, 6H). ^{13}C NMR (101 MHz, THF) δ 138.4, 133.9 (d, *J* = 5.0 Hz), 129.1, 128.4, 128.4, 128.0, 127.2, 126.6, 126.5, 125.2, 54.8, 21.5 ppm. ^{31}P NMR (162 MHz, THF) *δ* 221.2 ppm. Matches known data.[4]

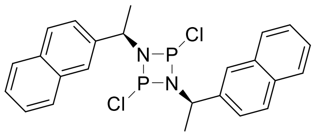

Cyclophosphazane SI-L14. Prepared according to representative procedure A. Purified by flash chromatography (hexane/ethyl acetate 99:1 to 95:5) to give the product as a colourless foam (479 mg, 40%). ^1H NMR (400 MHz, CD_2Cl_2) *δ* 7.89–7.76 (m, 4H), 7.72–7.63 (m, 2H), 7.49–7.19 (m, 14H), 6.97– 6.84 (m, 4H), 6.78 (dd, *J* = 8.5, 1.8 Hz, 2H), 3.76 (dtd, *J* = 11.8, 6.6, 5.0 Hz, 2H), 1.59 (d, *J* = 6.7 Hz, 6H). ^{13}C NMR (101 MHz, CD_2Cl_2) *δ* 151.8 (t, *J* = 7.0 Hz), 137.7 (d, *J* = 4.0 Hz) 137.6, 134.4, 132.8, 132.6, 130.5, 129.6, 127.9, 127.8, 127.4, 127.3, 126.8, 126.6, 125.8, 125.7, 125.5 (2C), 124.7, 123.8, 54.4 (t, *J* = 12.9 Hz), 23.1 (t, *J* = 4.4 Hz). ^{31}P NMR (162 MHz, CD_2Cl_2) *δ* 171.3 ppm. Matches known data.[4]

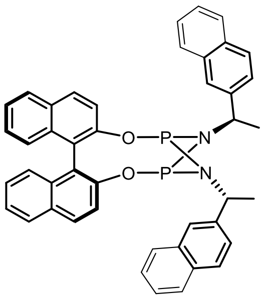

Cyclophosphazane SI-L15. Prepared according to representative procedure A. Purified by flash chromatography (hexane/ethyl acetate 99:1 to 95:5) to give the product as a colourless foam (724 mg, 41%). ^1H NMR (400 MHz, CD_2Cl_2) *δ* 8.03 (d, *J* = 8.8 Hz, 2H), 8.00–7.94 (m, 2H), 7.81–7.75 (m, 2H), 7.67 (d, *J* = 8.5 Hz, 2H), 7.49–7.39 (m, 10H), 7.36 (d, *J* = 8.9 Hz, 2H), 7.28 (ddd, *J* = 8.3, 6.8, 1.3 Hz, 2H), 7.23 (dd, *J* = 8.5, 1.9 Hz, 2H), 6.96 (dd, *J* = 8.5, 1.0 Hz, 2H), 4.06 (dtd, *J* = 11.7, 6.7, 4.9 Hz, 2H), 1.04 (d, *J* = 6.8 Hz, 6H). ^{13}C NMR (101 MHz, CD_2Cl_2) *δ* 151.6 (d, *J* = 12.9 Hz), 151.5 (d, *J* = 12.9 Hz), 142.0, 134.3, 133.2, 132.7, 130.6, 129.6, 128.1, 127.9, 127.8, 127.4, 126.7, 126.0, 125.9,

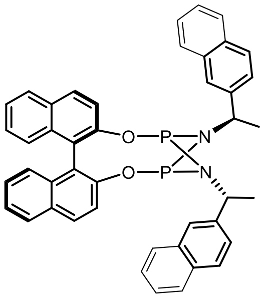

125.8, 125.7, 124.8, 124.8, 124.8, 124.6 (d, *J* = 2.3 Hz), 124.6 (d, *J* = 2.3 Hz), 124.3, 55.1 (d, *J* = 13.1 Hz), 55.0 (d, *J* = 13.1 Hz), 22.1 (d, *J* = 9.0 Hz), 22.0 (d, *J* = 9.0 Hz). ^{31}P NMR (162 MHz, CD_2Cl_2) δ 174.3. Matches known data.[4]

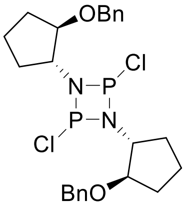

Dichlorophosphazane SI-7. To a solution of PCl_3 (2.3 mL, 26.4 mmol) in THF (50 mL) was added, dropwise, a solution of (1*R*,2*R*)-1-amino-2-benzyloxycyclopentane (5.02 g, 26.3 mmol) and triethylamine (37 mL, 266 mmol) in THF (50 mL) while maintaining an internal temp of –70 °C. After complete addition (ca. 2.5 hours) the thick beige reaction mixture was allowed to come to RT overnight. The reaction mixture was filtered under argon, washing the solids with an additional portion of THF (2 x 50 mL). The filtrate was concentrated to a brown solid. The solid was dissolved in hot toluene (20 mL) and when cooled was filtered again under argon to remove any residual salts and washing with additional toluene (2 x 10 mL). The toluene was removed at RT until the product had fully precipitated from the mixture and around 10 mL of toluene remained. Pentane (20 mL) was added to fully precipitate the product. The brown solid was isolated by filtration and washed with pentane. Light brown solid (733 mg, 11%). ^{1}H NMR (400 MHz, THF) δ 7.40–7.14 (m, 10H), 4.53 (d, *J* = 2.2 Hz, 4H), 4.06–3.93 (m, 2H), 3.70 (td, *J* = 6.8, 5.0 Hz, 2H), 2.11–1.98 (m, 3H), 1.86–1.71 (m, 7H), 1.70–1.58 (m, 2H). ^{31}P NMR (162 MHz, THF) δ 220.3 ppm. Matches known data.[4]

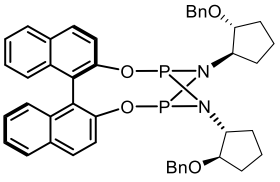

Cyclophosphazane SI-L17. Prepared according to representative procedure A. Purified by flash chromatography (hexane/ethyl acetate 99:1 to 95:5) to give the product as a colourless foam (71 mg, 17%). ^{1}H NMR (400 MHz, CD_2Cl_2) δ 7.94–7.86 (m, 4H), 7.36 (ddd, *J* = 8.1, 6.8, 1.2 Hz, 2H), 7.33–7.21 (m, 12H), 7.17 (ddd, *J* = 8.3, 6.8, 1.3 Hz, 2H), 6.84 (dd, *J* = 8.5, 1.1 Hz, 2H), 4.15–4.04 (m, 4H), 3.02 (dt, *J* = 15.8, 7.1 Hz, 4H), 1.72 (dq, *J* = 12.8, 6.9 Hz, 2H), 1.46 (ddd, *J* = 15.8, 7.9, 6.5 Hz, 4H), 1.41–1.30 (m, 2H), 1.14–1.07 (m, 4H). ^{13}C NMR (101 MHz, CD_2Cl_2) δ 151.8 (t, *J* = 6.5 Hz), 138.9, 134.1, 130.5, 129.3, 128.1, 127.7, 127.7, 127.3, 126.5, 125.7, 124.6, 124.2, 86.5, 71.5, 60.8 (t, *J* = 11.5 Hz), 49.0, 28.8, 28.1 (t, *J* = 7.3 Hz), 20.0 ppm. ^{31}P NMR (162 MHz, CD_2Cl_2) δ 174.4 ppm. Matches known data.[4]

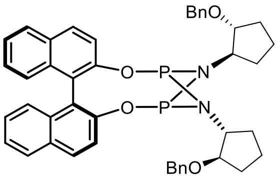

Cyclophosphazane SI-L18. Prepared according to representative procedure A. Purified by flash chromatography (hexane/ethyl acetate 99:1 to 95:5) to give the product as a colourless foam (109 mg, 19%). $[\alpha]_D^{20}$ = +182.8 (c = 0.99, CH_2Cl_2). ^{1}H NMR (400 MHz, CD_2Cl_2) δ 7.90 (dd, *J* = 9.0, 0.8 Hz, 2H), 7.83 (dt, *J* = 8.1, 0.9 Hz, 2H), 7.37–7.30 (m, 4H), 7.29–7.14 (m, 8H), 7.05–6.99 (m, 4H), 6.84 (dd, *J* = 8.5, 1.1 Hz, 2H), 3.96 (d, AB *J* = 11.6 Hz, 2H), 3.87 (d, AB *J* = 11.6 Hz, 2H), 3.66 (qd, *J* = 4.1, 2.0 Hz, 2H), 2.74– 2.66 (m, 2H), 1.89–1.73 (m, 4H), 1.68–1.41 (m, 8H). ^{13}C NMR (101 MHz, CD_2Cl_2) δ 151.8 (t, *J* = 7.0 Hz), 138.4, 134.3, 130.5,

129.6, 128.0 (2C), 127.8, 127.5, 127.2, 126.6, 125.6, 124.7, 123.9, 83.9 (t, J = 7.1 Hz), 70.5, 59.7 (t, J = 9.3 Hz), 31.4 (t, J = 5.2 Hz), 29.8, 21.2 ppm. ^{31}P NMR (162 MHz, CD_2Cl_2) δ 176.4. IR (ATR): ṽ = 3061, 2957, 2871, 1620, 1594, 1505, 1469, 1454, 1428, 1358, 1327, 1209, 1118, 1071, 1028, 966, 952, 886, 867, 821, 783, 750, 697, 640, 616, 584, 511. MS (ESIpos) m/z (%): 916 (3), 743.3 (100), 725.3 (42), 706.5 (2), 673.2 (2), 612.2 (7), 578.3 (8). HRMS (ESI$^+$) m/z calcd. for $C_{44}H_{43}N_2O_4P_2$: 725.2693, found: 725.2695.

Dichlorophosphazane SI-8. To a solution of 2-(naphthalen-2-yl)propan-2-amine (2.1 g, 11.4 mmol) in diethyl ether (50 mL) at –78 °C was added *n*-BuLi (8 mL, 1.51 M in hexanes, 12.1 mmol) dropwise. Once the addition was complete the yellow suspension was allowed to reach RT and stirred for 10 minutes. The reaction was then re-cooled to –78 °C and TMSCl (1.6 mL, 12.6 mmol) was added dropwise and the reaction mixture was allowed to come to rt. Once the consistency of the precipitate had lightened and the colour faded from yellow to off-white, the volatiles were removed and the resulting oily suspension was flash distilled under high vacuum to give the TMS protected amine as a colourless oil (557 mg, 19%).

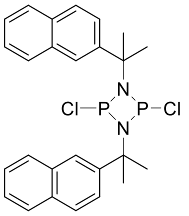

The oil was dissolved in diethyl ether (5 mL) and *n*BuLi (1.5 mL, 2.27 mmol) was added in a steady stream at 0 °C forming an orange solution. After stirring for 5 mins at rt the reaction mixture was cooled to –78 °C and transferred via cannula to a solution of PCl_3 (0.188 mL, 2.14 mmol) in diethyl ether (5 mL) at –78 °C. During the addition the colour of the solution remained very pale yellow and essentially homogenous. After addition was complete the solution was warmed to rt. During warming a colourless precipitate formed, and the solution remained pale yellow. The reaction was stirred at rt for 1 hour before the volatiles were removed and the residue was suspended in hexane (35 mL) and filtered, washing with hexane (2 x 5 mL). The filtrate was concentrated until crystallization began and was then cooled to –78 °C and the liquid was removed using a syringe. Pale yellow solid (157 mg, 29%). ^{1}H NMR (400 MHz, THF) δ 7.96 (d, J = 2.0 Hz, 2H), 7.90–7.80 (m, 6H), 7.71 (dd, J = 8.7, 2.0 Hz, 2H), 7.49–7.43 (m, 4H), 1.89 (d, J = 1.1 Hz, 12H). ^{13}C NMR (101 MHz, THF) δ 142.45 (t, J = 2.7 Hz), 133.8, 133.4, 128.8, 128.7, 127.7, 126.6, 126.5, 124.9 (t, J = 1.6 Hz), 124.6 (t, J = 2.1 Hz), 59.4 (t, J = 7.9 Hz), 29.2 (t, J = 6.3 Hz). ^{31}P NMR (162 MHz, THF) δ 206.7 ppm. MS (EI) m/z (%): 498 (17), 315 (4), 234 (5), 193 (4), 169 (100), 141 (12), 41 (3). HRMS (EI): m/z calcd. for $C_{26}H_{26}N_2P_2Cl_2$: 498.0948, found: 498.0947.

Cyclophosphazane SI-L19. Prepared according to representative procedure A. Purified by flash chromatography (hexane/ethyl acetate 99:1 to 95:5) to give the product as a colourless solid (93 mg, 47%). $[\alpha]_D^{20}$ = –313.7 (c = 1.05, CH_2Cl_2). ^{1}H NMR (400 MHz, CD_2Cl_2) δ 7.92 (d, J = 8.6 Hz, 4H), 7.70 (d, J = 8.1 Hz, 2H), 7.52–7.17 (m, 16H), 7.01–6.92 (m, 2H), 6.86 (d, J = 8.5 Hz, 2H), 1.75 (s, 6H), 1.13 (s, 6H). ^{13}C NMR (101 MHz, CD_2Cl_2) δ 151.8 (t, J = 7.5 Hz), 144.1 (t, J = 2.4 Hz), 134.9, 132.8, 132.2, 130.7, 129.2, 128.0, 127.9, 127.4, 127.1, 126.6, 126.3, 126.0, 125.7, 125.7, 125.4, 124.8, 124.7, 124.3, 57.9 (t, J = 14.8 Hz), 30.5 (t, J = 6.8 Hz), 29.2 (t, J = 5.2 Hz). ^{31}P NMR (162 MHz, CD_2Cl_2) δ 173.2 ppm. IR (ATR): ṽ = 3056, 2967, 1619, 1595, 1505, 1469, 1426, 1366, 1323, 1264, 1200, 1158, 1132, 1071, 999, 974, 952, 936, 879, 859, 821, 788, 748, 688, 680, 648, 632, 582, 509, 477. HRMS (ESI$^+$) m/z calcd. for $C_{46}H_{39}N_2O_2P_2$: 713.2481, found: 713.2482.

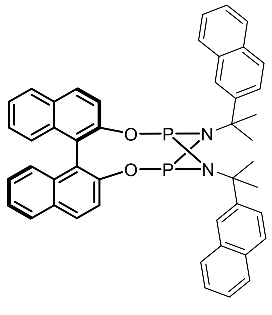

Cyclophosphazane **SI-L20.** Prepared according to representative procedure A. Purified by flash chromatography (hexane/ethyl acetate 99:1 to 95:5) to give the product as a colourless solid (81 mg, 37%).

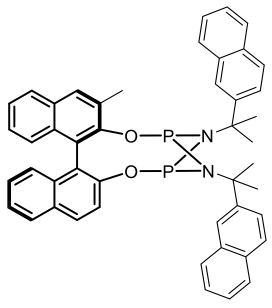

^{1}H NMR (400 MHz, CD_2Cl_2) δ 7.96–7.79 (m, 5H), 7.72 (t, J = 9.6 Hz, 2H), 7.54–7.15 (m, 14H), 7.06 (d, J = 8.8 Hz, 1H), 6.86 (dd, J = 13.5, 8.6 Hz, 2H), 6.74 (d, J = 8.6 Hz, 1H), 2.41 (s, 3H), 1.72 (s, 3H), 1.69 (s, 3H), 1.11 (s, 3H), 1.09 (s, 3H). ^{13}C NMR (101 MHz, CD_2Cl_2) δ 151.7 (d, J = 11.0 Hz), 151.6 (d, J = 11.5 Hz), 144.6, 143.7, 134.9, 133.8, 132.8, 132.8, 132.3, 132.2, 131.6, 130.8, 130.7, 129.3, 129.1, 128.1, 127.8, 127.5, 127.4, 127.2, 126.6, 125.9, 125.8, 125.8, 125.2, 125.0, 124.8, 124.7, 124.6, 124.3, 124.1, 57.9 (d, J = 11.9 Hz), 57.7 (d, J = 11.7 Hz), 30.2 (t, J = 6.9 Hz, 2C), 29.1, 28.8, 19.0 ppm. ^{31}P NMR (162 MHz, CD_2Cl_2) δ 172.5 (d, J = 24.6 Hz), 171.9 (d, J = 24.6 Hz). IR (ATR): ṽ = 3056, 2968, 1712, 1620, 1597, 1505, 1465, 1416, 1360, 1274, 1260, 1230, 1201, 1164, 1134, 1095, 1000, 959, 941, 889, 859, 820, 792, 747, 650, 477. HRMS (ESI$^+$) m/z calcd. for $C_{47}H_{41}N_2O_2P_2$: 727.2638, found: 727.2641.

Cyclophosphazane SI-L21. Prepared according to representative procedure A. Purified by flash chromatography (hexane/ethyl acetate 99:1 to 95:5) to give the product as a colourless solid (101 mg, 45%).

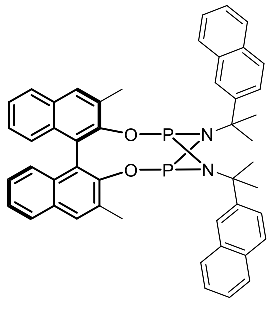

$[\alpha]_D^{20}$ = –247.9 (c = 1.0, CH_2Cl_2). ^{1}H NMR (400 MHz, CD_2Cl_2) δ 7.84 (d, J = 8.2 Hz, 2H), 7.79 (s, 2H), 7.72 (d, J = 7.8 Hz, 2H), 7.50–7.45 (m, 2H), 7.46– 7.35 (m, 8H), 7.32 (d, J = 8.7 Hz, 2H), 7.20 (t, J = 7.7 Hz, 2H), 6.97 (dd, J = 8.7, 2.0 Hz, 2H), 6.72 (d, J = 8.5 Hz, 2H), 2.40 (s, 6H), 1.63 (s, 6H), 1.09 (s, 6H). ^{13}C NMR (101 MHz, CD_2Cl_2) δ 151.5 (t, J = 7.1 Hz), 144.2 (t,

J = 2.5 Hz), 133.8, 132.8, 132.3, 131.4, 130.7, 129.2, 128.1, 127.4, 127.1, 127.1, 126.5 (t, J = 2.1 Hz), 125.8, 125.6, 124.8, 124.7, 124.0, 57.7 (t, J = 14.6 Hz), 30.0 (t, J = 6.7 Hz), 29.0 (t, J = 5.3 Hz), 18.9 ppm. ^{31}P NMR (162 MHz, CD_2Cl_2) δ 171.7 ppm. IR (ATR): ṽ = 3056, 2968, 1712, 1599, 1503, 1460, 1423, 1361, 1329, 1263, 1230, 1203, 1179, 1133, 1101, 1089, 1031, 999, 942, 911, 885, 859, 817, 768, 748, 703, 631, 592, 538, 477. HRMS (ESI$^+$) m/z calcd. for $C_{48}H_{43}N_2O_2P_2$: 741.2794, found: 741.2801.

Representative Procedure B for *ortho*-Substituted Aniline-derived Cyclodiphosphazanes

Note: *This procedure, based on a report by Schulz,[11] reacts a deprotonated silylamine with PCl_3 to form the intermediate dichlorocyclophosphazane, eliminating TMSCl (which can be removed in vacuo) and lithium chloride, which does not interfere in the subsequent cyclisation with the chiral diol. It is recommended over the procedure described on page S11, which requires purification of the sensitive dichlorocyclophosphazane. An isolated silylamine is used in the original synthesis. However, we found that the procedure also works well for these ortho-substituted anilines if the silylamine is simply made in situ by deprotonation with n-BuLi and reaction with TMSCl, avoiding purification of this often moisture-sensitive compound.*

Cyclophosphazane SI-L22. 2-Phenylaniline (1.75 g, 10.0 mmol) was dissolved in THF (50 mL) and the solution cooled to -78 °C. *n*-Butyllithium (7.0 mL, 10.2 mmol, 1.6 M in hexanes) was added and the solution was stirred at the same temperature for 20 min. TMSCl (1.3 mL, 10.5 mmol) was added and the solution was stirred for 30 min. *n*-Butyllithium (7.0 mL, 10.2 mmol, 1.6 M in hexanes) was added and the solution was stirred for a further 20 min. In a separate Schlenk flask, PCl_3 (0.87 mL, 10.0 mmol) was dissolved in THF (25 mL) and the solution was cooled to -78 °C. The cold (-78 °C) solution of lithium silylamide was transferred rapidly by cannula to the PCl_3 solution. The resulting orange solution was stirred at the same temperature for 5 min, then allowed to warm to rt and stirred for 1 hour, typically turning light yellow over time. The solvent and volatiles were removed *in vacuo* using a low vacuum pump attached to the Schlenk flask through a liquid nitrogen trap. Following evaporation to dryness, (*R*)-BINOL (1.43 g, 5.0 mmol) was added to the flask. Toluene (70 mL) and triethylamine (7.0 mL, 50 mmol) were added, the flask was transferred to a pre-heated oil bath at 130 °C and the mixture refluxed overnight. The reaction was allowed to cool to rt and filtered through SiO_2 (washed with toluene). The solvent was evaporated under reduced pressure to give a fluffy light yellow solid. Purification by flash chromatography (SiO_2, hexane/ethyl acetate 98:2 to 96:4) gave a fluffy off-white solid (1.39 g). Re-purification by flash chromatography (SiO_2, hexane/dichloromethane 9:2 to to 3:1) gave the product as a white solid (633 mg, 19% yield). $[\alpha]_D^{20}$ = -248 (c = 0.1, CH_2Cl_2); ^{1}H (400 MHz, CD_2Cl_2) δ = 7.77 (d,J = 8.0 Hz, 2H), 7.42-7.32 (m,

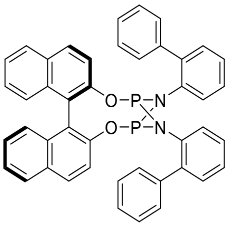

10H), 7.31-7.26 (m, 4H), 7.26-7.21 (m, 2H), 7.09 (dd, J = 7.5, 1.5 Hz, 2H), 6.96 (td, J = 7.5, 1.0 Hz, 2H), 6.93-6.90 (m, 2H), 6.73 (d, J = 9.0 Hz, 2H), 6.58 (td, J = 7.5, 1.5 Hz, 2H), 5.93 (d, J = 8.0 Hz, 2H); ^{13}C (101 MHz, CD_2Cl_2) δ = 151.9 (t, J = 6.5 Hz), 139.7, 138.3 (t, J = 8.5 Hz), 134.8, 134.4, 131.1, 130.8, 130.3 (t, J = 3.0 Hz), 129.2, 129.0, 128.4, 128.2, 128.1, 126.9, 126.0, 125.2, 125.09 (t, J = 2.0 Hz), 123.9, 123.7, 121.1 (t, J = 6.5 Hz); ^{31}P (162 MHz, CD_2Cl_2) δ = 175.0 (s); IR (ATR): ṽ = 3053, 3019, 1592, 1477, 1434, 1269, 1205, 1191, 1070, 950, 903, 818, 782, 749, 697, 685, 641, 598, 491; HRMS (ESI$^+$, m/z) calculated for $[C_{44}H_{31}N_2O_2P_2]^+$ 681.18602, found 681.18553.

Notes:

1. Cyclophosphazanes in general are acid-sensitive, often streaking on TLC plates and decomposing in $CDCl_3$. For this reason CD_2Cl_2 or C_6D_6 were usually used as NMR solvents.
2. None of the cyclophosphazanes prepared for this project were air-sensitive.
3. The *ortho*-substituted aniline-derived BINOL cyclodiphosphazanes proved sensitive to silica and this instability increased with larger *o*-aryl substituents such as 2-naphthyl and 2,6-dimethylphenyl, though the octahydro-BINOL analogues were more stable. This class of cyclophosphazanes typically required two flash columns to obtain pure and the low yields reflect loss of material and their propensity to form phosphoramidites as a side-product during the cyclisation step,[4] rather than the method of dichlorophosphazane synthesis, which typically achieved >95% purity as described below.
4. The purity of the intermediate dichlorocyclophosphazanes was checked by ^{31}P NMR spectroscopy in degassed, anhydrous d_8-THF. It is pivotal that this intermediate is obtained in high purity, otherwise the subsequent cyclisation will likely fail. Though the *ortho*-aryl-derived dichloro intermediates were fairly stable, likely due to steric shielding, others were found to be air-sensitive and ***in some cases pyrophoric. Care should therefore be taken when handling them***.

Cyclophosphazane SI-L23. Prepared according to representative procedure B. Purification by flash chromatography (SiO_2, hexane/dichloromethane 6:1 to 4:1) gave product **SI-L23** as a white solid (47 mg, 5% yield). $[\alpha]_D^{20}$ = −151 (c = 0.1, CH_2Cl_2); ^{1}H (400 MHz, C_6D_6) δ = 7.59 (d, J = 7.5 Hz, 1H), 7.50 (d, J = 7.5 Hz, 1H), 7.38 (t, J = 7.0 Hz, 4H), 7.26-7.16 (m, 8H), 7.15-7.07 (m, 4H), 7.01-6.96 (m, 2H), 6.94-6.89 (m, 2H), 6.85 (d, J = 8.5 Hz, 1H), 6.76-6.66 (m, 2H), 6.39 (d, J = 7.5 Hz, 1H), 6.33-6.26 (m, 2H), 6.03 (d, J = 8.0 Hz, 1H), 2.07 (s, 3H); ^{13}C (101 MHz, C_6D_6) Note: due to the complexity of the spectrum, a simple list of peaks without couplings is given. δ = 151.9, 151.8, 151.1, 150.9, 138.7(0), 138.6(7), 138.6(4), 138.6(1), 137.8, 137.7, 137.6,

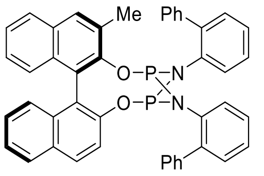

137.5, 136.4, 136.3, 136.2, 136.1, 135.7(9), 135.7(6), 133.5, 133.1(8), 133.1(5), 132.4, 130.1, 130.0, 129.8(7), 129.8(5), 129.5, 129.4, 129.2(4), 129.2(0), 128.7(9), 128.7(7), 128.4, 128.2, 127.8, 127.7, 127.5, 127.3, 126.9, 126.7, 126.2, 125.7, 125.3, 125.2, 125.1, 124.6, 124.5, 124.4(1), 124.3(8), 124.1, 123.8, 123.8, 123.3(7), 123.3(2), 123.2(8), 122.6, 122.2, 119.1(1), 119.0(7), 119.0(2), 118.9(9), 17.7(2), 17.6(8); ^{31}P (162 MHz, C_6D_6) δ = 179.9 (d, J = 36.0 Hz), 177.0 (d, J = 36.0 Hz); IR (ATR): ṽ = 3053, 3016, 1592, 1476, 1433, 1268, 1233, 1197, 1091, 957, 904, 746, 698, 643, 597, 492; HRMS (ESI$^+$, m/z) calculated for $[C_{45}H_{33}N_2O_2P_2]^+$ 695.20120, found 695.20118.

Cyclophosphazane SI-L24. Prepared according to representative procedure B. Purification

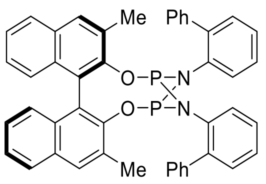

by flash chromatography (SiO_2, hexane/dichloromethane 8:1) then (SiO_2, hexane/ethyl acetate 96:4) gave product **SI-L24** as a white solid (31 mg, 3% yield). $[\alpha]_D^{20}$ = -246 (c = 0.1, CH_2Cl_2); ^{1}H (400 MHz, CD_2Cl_2) δ = 7.72-7.69 (m, 2H), 7.54-7.47 (m, 8H), 7.40-7.33 (m, 4H), 7.30 (app. br. s, 2H), 7.18-7.13 (m, 4H), 7.00 (td, J = 7.6, 1.2 Hz, 2H), 6.84-6.81 (m, 2H), 6.49 (td, J = 7.7, 1.6 Hz, 2H), 5.71 (d, J = 7.9 Hz, 2H), 2.03(0) (s, 3H,, Me_a), 2.02(8) (s, 3H, Me_b); ^{13}C (101 MHz, CD_2Cl_2) δ = 152.7 (t, J = 6.3 Hz), 139.8, 137.2 (t, J = 4.3 Hz), 136.1, 133.2, 131.3, 131.0, 130.7, 130.2, 129.1, 128.9, 128.3, 128.0, 127.4, 126.0, 125.8, 125.4 (t, J = 1.7 Hz), 125.2, 125.0, 123.54 (t, J = 4.3 Hz), 19.0; ^{31}P (162 MHz, CD_2Cl_2) δ = 180.9 (s); IR (ATR): ṽ = 3053, 3017, 2921, 1594, 1477, 1432, 1266, 1231, 1207, 1097, 942, 905, 765, 744, 698, 592, 493; HRMS (ESI$^+$, m/z) calculated for $[C_{46}H_{35}N_2O_2P_2]^+$ 709.21722, found 709.21683.

Cyclophosphazane SI-L25. Prepared according to representative procedure B. Purification

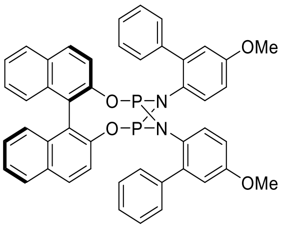

by flash chromatography (SiO_2, hexane/ethyl acetate 98:2 to 96:4 to 92:8) and subsequent trituration with ethyl acetate gave product **SI-L25** as a white solid (50 mg, 3% yield). $[\alpha]_D^{20}$ = -245 (c = 0.1, CH_2Cl_2); ^{1}H (400 MHz, C_6D_6) δ = 7.53 (d, J = 8.0 Hz, 2H), 7.41 (d, J = 7.0 Hz, 4H), 7.28-7.19 (m, 9H), 7.18-7.10 (m, 3H), 6.95-6.90 (m, 4H), 6.72 (d, J = 3.0 Hz, 2H), 6.07 (d, J = 8.5 Hz, 2H), 5.96 (dd, J = 8.5, 3.0 Hz, 2H), 3.19 (s, 6H); ^{13}C (101 MHz, C_6D_6) δ = 156.7, 152.6 (t, J = 6.5 Hz), 140.0, 136.7, 134.8, 131.6 (t, J = 9.0 Hz), 131.2, 130.2, 129.2, 128.9, 128.1, 127.1, 126.5, 125.6, 125.1, 124.3, 123.5 (t, J = 5.5 Hz), 116.1, 113.9, 55.0; ^{31}P (162 MHz, C_6D_6) δ = 177.6 (s); IR (ATR): ṽ = 3056, 3024, 2832, 1597, 1481, 1268, 1205, 1039, 949, 898, 850, 815, 781, 751, 679, 616; LRMS m/z (EI$^+$) $[M]^+$ 740; HRMS (EI$^+$, m/z) calculated for $[C_{46}H_{34}N_2O_4P_2]^+$ 740.19866, found 740.19939.

Cyclophosphazane SI-L26. Prepared according to representative procedure B. Purification by flash chromatography (SiO_2, hexane/ethyl acetate 98:2 to 96:4 to 92:8) gave product **SI-L26** as an off-white solid (250 mg, 16% yield). $[\alpha]_D^{20}$ = -377 (c = 0.1, CH_2Cl_2); ^1H (400 MHz, C_6D_6) δ = 7.54 (d, *J* = 8.0 Hz, 4H), 7.36 (t, *J* = 8.0 Hz, 4H), 7.23-7.17 (m, 2H), 6.74 (d, *J* = 3.0 Hz, 2H), 6.51 (d, *J* 8.0 Hz, 2H), 6.42 (dd, *J* = 8.5, 3.0 Hz, 4H), 6.32 (d, *J* = 8.5 Hz, 2H), 3.28 (s, 6H), 2.54-2.42 (m, 6H), 2.11-2.03 (m, 2H), 1.54-1.45 (m, 8H); ^{13}C (101 MHz, C_6D_6) δ = 156.7, 151.0 (t, *J* = 6.0 Hz), 140.3, 136.9, 136.5, 133.1, 132.0 (t, *J* = 8.5 Hz), 131.1, 130.2 (t, *J* = 2.5 Hz), 129.2, 129.0, 123.7 (t, *J* = 4.5 Hz), 121.4, 115.5, 113.9, 55.1, 30.0, 28.2, 23.7, 23.3; ^{31}P (162 MHz, C_6D_6) δ = 174.7 (s); IR (ATR): ṽ = 3056, 3025, 2931, 2832, 1597, 1481, 1466, 1268, 1205, 1038, 949, 897, 849, 815, 781, 756, 679, 616; LRMS m/z (EI^+) $[M]^+$ 748; HRMS (EI^+, m/z) calculated for $[C_{46}H_{42}N_2O_4P_2]^+$ 748.26127, found 748.26199.

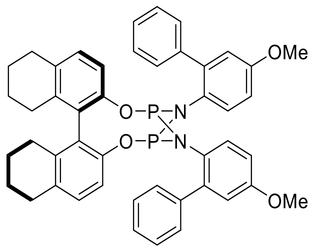

Cyclophosphazane SI-L27. Prepared according to representative procedure B. Purification by flash chromatography (SiO_2, hexane/ethyl acetate 95:5 to 9:1 to 4:1) then (SiO_2, hexane/toluene 4:1 to 3:1 to 2:1) gave product **SI-L27** as an off-white solid (67 mg, 7% yield). $[\alpha]_D^{20}$ = -353 (c = 0.1, CH_2Cl_2); ^1H (400 MHz, CD_2Cl_2) δ = 7.89-7.86 (m, 4H), 7.74 (dd, 2H, *J* = 7.5, 2.0 Hz), 7.69 (dd, 2H, *J* = 8.0, 1.5 Hz), 7.64 (dd, 2H, *J* = 8.5, 1.5 Hz), 7.33-7.25 (m, 4H), 7.07 (dd, 2H, *J* = 7.5, 1.5 Hz), 6.80 (td, 2H, *J* = 7.5, 1.5 Hz), 6.68 (td, 2H, *J* = 7.5, 1.5 Hz), 6.46 (d, 2H, *J* = 8.5 Hz), 6.42 (d, 2H, *J* = 8.0 Hz), 6.36 (d, 2H, *J* = 8.5 Hz), 2.50-2.38 (m, 6H), 2.10-2.00 (m, 2H), 1.53-1.42 (m, 8H); ^{13}C (101 MHz, $CDCl_3$) δ = 149.4 (t, *J* = 6.3 Hz), 137.9 (t, *J* = 8.0 Hz), 136.6, 135.2, 133.3, 133.2, 132.2, 131.9, 129.7, 129.6 (t, *J* = 2.0 Hz), 128.2, 127.7, 127.4, 127.2, 126.9, 126.7, 125.3, 125.2, 122.1, 119.8, 119.7(4), 119.6(8), 28.7, 26.9, 22.5, 22.1; ^{31}P (162 MHz, CD_2Cl_2) δ = 171.7 (s); IR (ATR): ṽ = 3052, 2962, 2918, 2855, 1589, 1489, 1464, 1442, 1261, 1214, 1054, 1024, 937, 892, 811, 783, 750; HRMS (ESI^+, m/z) calculated for $[C_{52}H_{43}N_2O_2P_2]^+$ 789.27943, found 789.27916.

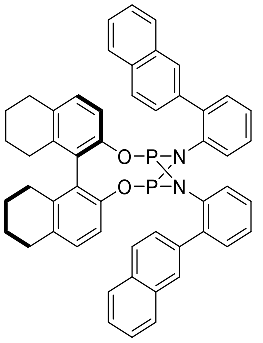

Note: synthesis of the related BINOL derivative was attempted but the product could not be isolated.

Cyclophosphazane SI-L28. Prepared according to representative procedure B. Purification by flash chromatography (SiO_2, hexane/dichloromethane 4:1) then (SiO_2, hexane/dichloromethane 6:1) gave product **SI-L28** as an off-white solid (82 mg, 6% yield). $[\alpha]_D^{20}$ = -307 (c = 0.1, CH_2Cl_2); ^1H (400 MHz, C_6D_6) δ = 7.50 (d, 2H, *J* = 8.0 Hz), 7.20-7.16 (m, 4H), 7.09 (t, 2H, *J* = 7.5 Hz), 7.02 (d, 2H, *J* = 8.0 Hz), 6.93-6.82 (m, 6H), 6.79 (d, 2H, *J* = 8.0 Hz), 6.65-6.59

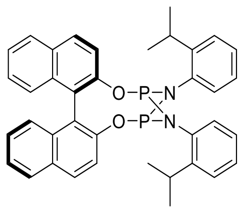

(m, 2H), 3.28-3.16 (m, 2H), 1.22 (d, 6H, J = 6.5 Hz), 0.71 (d, 2H, J = 6.5 Hz); ^{13}C (101 MHz, C_6D_6) δ = 152.3 (t, J = 7.0 Hz), 141.5, 138.7 (t, J = 9.5 Hz), 134.7, 131.3, 129.7, 128.2, 127.1, 126.7, 126.6, 126.5, 125.8, 125.2, 124.7, 124.0, 122.7 (t, J = 9.0 Hz), 28.1 (t, J = 5.5 Hz), 25.6 (t, J = 3.5 Hz), 23.2; ^{31}P (162 MHz, C_6D_6) δ = 175.3 (s); IR (ATR): ṽ = 3057, 2923, 2856, 1593, 1442, 1240, 1210, 949, 897, 815, 788, 747; LRMS m/z (EI^+) $[M]^+$ 612; HRMS (EI^+, m/z) calculated for $[C_{38}H_{34}N_2O_2P_2]^+$ 612.20913, found 612.20956.

Cyclophosphazane SI-L29. Prepared according to representative procedure B. Purification

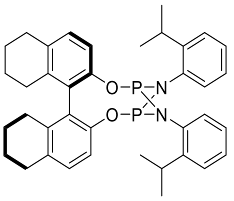

by flash chromatography (SiO_2, hexane/dichloromethane 9:1 to 6:1) gave product **SI-L29** as an off-white solid (165 mg, 13% yield). $[\alpha]_D^{20}$ = -181 (c = 0.1, CH_2Cl_2); ^{1}H (400 MHz, C_6D_6) δ = 7.09 (dd, 2H, J = 7.5, 1.5 Hz), 6.96-6.88 (m, 4H), 6.83 (td, 2H, J = 7.5, 1.5 Hz), 6.47 (d, 2H, J = 8.5 Hz), 6.36 (d, 2H, J = 8.5 Hz), 3.46-3.34 (m, 2H), 2.52-2.40 (m, 6H), 2.15-2.04 (m, 2H), 1.60-1.43 (m, 8H), 1.31 (d, 6H, J = 7.0 Hz), 1.09 (d, 6H, J = 7.0 Hz); ^{13}C (101 MHz, C_6D_6) δ = 150.9 (t, J = 7.0 Hz), 141.2, 139.2 (t, J = 9.0 Hz), 136.7, 133.4, 131.2, 129.6, 126.5, 126.4, 124.4, 122.5 (t, J = 9.0 Hz), 121.3, 29.8, 28.4 (t, J = 6.5 Hz), 28.3, 25.2 (t, J = 3.0 Hz), 23.9, 23.7, 23.3; ^{31}P (162 MHz, CD_2Cl_2) δ = 172.1 (s)*;* IR (ATR): ṽ = 3022, 2924, 2857, 1596, 1487, 1443, 1240, 1213, 938, 894, 785, 748, 657; HRMS (ESI^+, m/z) calculated for $[C_{38}H_{43}N_2O_2P_2]^+$ 621.2791, found 621.2794.

Cyclophosphazane SI-L30. Prepared according to representative procedure B. Purification

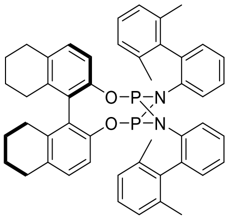

by flash chromatography (SiO_2, hexane/ethyl acetate 98:2 to 96:4) then (SiO_2, hexane/dichloromethane 6:1) gave product **SI-L30** as an off-white solid (60 mg, 7% yield). $[\alpha]_D^{20}$ = -304 (c = 0.1, CH_2Cl_2); ^{1}H (400 MHz, C_6D_6) δ = 7.13 (d, J = 7.5 Hz, 2H), 7.05-7.00 (m, 4H), 6.81 (app. pent. d, 7.5, 2.0 Hz, 4H), 6.73 (dd, J = 7.0, 2.0 Hz, 2H), 6.69 (d, J = 8.0 Hz, 2H), 6.63 (d, J = 8.5 Hz, 2H), 6.40 (d, J = 8.5 Hz, 2H), 2.52-2.39 (m, 6H), 2.13-2.02 (m, 8H), 1.78 (s, 6H), 1.56-1.44 (m, 8H); ^{13}C (101 MHz, C_6D_6) δ = 150.8 (t, J = 7.0 Hz), 139.7 (t, J = 9.0 Hz), 138.5, 137.8 (t, J = 3.0 Hz), 137.6, 136.5, 133.3, 132.4, 131.2, 130.6, 129.2, 127.7, 127.5, 123.5, 121.3, 120.4 (t, J = 7.3 Hz), 29.8, 28.2, 23.7, 23.3, 21.6 (t, J = 3.0 Hz), 21.0 (t, J = 2.5 Hz); ^{31}P (162 MHz, C_6D_6) δ = 172.2 (s); IR (ATR): ṽ = 3018, 2921, 2855, 1590, 1462, 1440, 1244, 1214, 1056, 937, 894, 749;LRMS m/z (EI^+) $[M]^+$ 744; HRMS (EI^+, m/z) calculated for $[C_{48}H_{46}N_2O_2P_2]^+$ 744.30325, found 744.30346.

Note: synthesis of the related BINOL derivative was attempted but the product could not be isolated.

Synthesis of Phosphoramidite Ligands

(*R*)-VAPhos-NEt$_2$ L17. (*R*)-VAPOL (470 mg, 0.873 mmol) was added to an oven-dried reaction tube under argon and dissolved in toluene (4.2 mL). Tris(diethylamino)phosphine (0.24 mL, 0.873 mmol) was added and the tube was sealed and stirred at 110 °C for 16 h. After cooling to room temperature, the solution was concentrated *in vacuo* to give an orange oil. Purification by flash chromatography (SiO_2, hexane/ethyl acetate 98:2) gave phosphoramidite **L17** as a white solid (559 mg, quant.). $[\alpha]_D^{20}$ = -580 (c = 0.1, CH_2Cl_2); ^{1}H (400 MHz, C_6D_6) δ = 10.24 (d, *J* = 8.5 Hz, 1H), 10.18 (d, *J* = 8.5 Hz, 1H), 7.74-7.69 (m, 2H), 7.65-7.60 (m, 1H), 7.56-7.38 (m, 9H), 6.93-6.87 (m, 2H), 6.84-6.80 (m, 2H), 6.79-6.73 (m, 6H), 2.89 (ddq, *J* = 14.0, 9.5, 7.0 Hz, 2H), 2.73-2.59 (m, 2H), 0.66 (t, *J* = 7.0 Hz, 6H); ^{13}C (101 MHz, C_6D_6) Note: due to the complexity of the spectrum, a simple list of peaks without couplings (except for the alkyl signals) is given. δ = 150.9, 150.8(1), 150.7(6), 142.0(2), 142.0(0), 141.9, 140.8, 140.7, 135.0, 134.8, 134.0, 133.6, 130.7, 130.5, 130.0(3), 130.0(2), 129.4(7), 129.4(2), 129.3(5), 128.8(9), 128.8(4), 128.7, 128.2, 127.9, 127.7, 127.5, 127.3, 127.2, 127.1, 126.9, 126.8, 126.7(2), 126.6(7), 126.2, 122.8(1), 122.7(9), 122.7, 39.6 (d, *J* = 22.3 Hz), 14.8 (d, *J* = 3.4 Hz); ^{31}P (162 MHz, C_6D_6) δ = 143.3 (s); IR (ATR): ṽ = 3051, 2967, 1594, 1556, 1369, 1327, 1173, 1124, 1016, 974, 874, 812, 741, 696, 497*;* LRMS m/z (EI$^+$) [M]$^+$ 639; HRMS (ESI$^+$, m/z) calculated for $[C_{44}H_{35}NO_2P]^+$ 640.23942, found 640.23999.

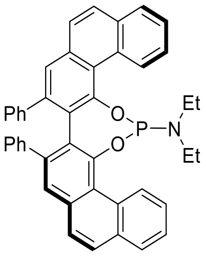

Notes:

1. **L17** has not shown evidence of air-instability but is stored in the freezer at -20 °C as a precaution since the related *N*,*N*-dimethyl derivative **L16** appears to be slightly air-sensitive. Furthermore, rotary evaporation was performed at 30 °C for this and similar VAPOL/VANOL phosphoramidites.
2. (R)-VAPOL (CAS 147702-16-7) was typically bought from Sigma-Aldrich and determined as 99.8% ee by chiral HPLC.
3. The reaction was performed in a crimp-capped vial as this was more convenient on small scale. However, very similar reactions on BINOL derivatives have been performed under reflux and it can be expected that this would also work well for this substrate.
4. Once it solidifies after purification and evaporation of the solvent, **L17** cannot readily be dissolved in hexane or ethyl acetate. If the isolated solid needs to be re-dissolved, toluene is more appropriate as it is well-soluble in this solvent.

Phosphoramidite L15. (*S*)-VANOL (30 mg, 0.068 mmol) was added to an oven-dried reaction tube under argon and dissolved in toluene (0.40 mL). Tris(diethylamino)phosphine (19 µL, 0.068 mmol) was added and the tube was sealed and stirred at 110 °C for 16 h. After cooling to room temperature, the solution was concentrated *in vacuo*. Purification by flash chromatography (SiO_2, hexane/ethyl acetate 97:3) gave phosphoramidite **L15** as a white solid (42 mg, quant.). $[\alpha]_D^{20}$ = 289 (c = 0.1, CH_2Cl_2); ^1H (400 MHz, C_6D_6) δ = 8.64 (d, *J* = 8.5 Hz, 1H), 8.56 (d, *J* = 8.0 Hz, 1H), 7.61 (d, *J* = 8.0 Hz, 1H), 7.56 (d, *J* = 8.0 Hz, 1H), 7.43 (s, 1H), 7.40-7.24 (m, 5H), 6.92-6.86 (m, 2H), 6.76-6.65 (m, 8H), 3.15 (ddt, *J* = 16.0, 14.0, 7.0 Hz, 2H), 2.66 (ddt, *J* = 18.5, 14.0, 7.0 Hz, 2H), 0.83 (t, *J* = 7.0 Hz, 6H); ^{13}C (101 MHz, C_6D_6) Note: due to the complexity of the spectrum, a simple list of peaks without couplings (except for the alkyl signals) is given δ = 149.0, 148.9, 148.5(7), 148.5(6), 141.4(2), 141.4(1), 141.2(4), 141.2(3), 141.1(2), 141.0(9), 134.9, 134.6, 129.7, 129.6, 128.4, 128.2, 127.9, 127.6(2), 127.6(0), 127.5, 127.2, 127.1, 126.7, 126.6, 126.5, 126.2, 126.1, 125.7(4), 125.7(0), 125.0, 124.3(3), 124.3(1), 123.7, 122.8, 39.1 (d, *J* = 22.1 Hz), 14.9 (d, *J* = 3.0 Hz); ^{31}P (162 MHz, CD_2Cl_2) δ = 152.0 (s); IR (ATR): ṽ = 3051, 2967, 2928, 2869, 1563, 1487, 1360, 1174, 1086, 1018, 884, 819, 748, 719, 696, 496; LRMS m/z (EI^+) $[M]^+$ 539; HRMS (ESI^+, m/z) calculated for $[C_{36}H_{31}NO_2P]^+$ 540.20855, found 540.20869.

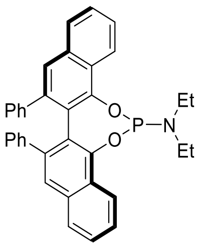

*Note: synthesis of the related dimethyl derivative was attempted, but it proved too air-sensitive to be isolated using column chromatography. It was not further pursued due to the moderate ee obtained in the nickel-catalysed reductive coupling using **L15**.*

Phosphoramidite L16. (*S*)-VAPOL (25 mg, 0.046 mmol) was added to an oven-dried reaction tube under argon and dissolved in toluene (0.27 mL). Tris(dimethylamino)phosphine (8.5 µL, 0.046 mmol) was added and the tube was sealed and stirred at 110 °C for 15 h. After cooling to room temperature, the solution was concentrated *in vacuo*. Purification by flash chromatography (SiO_2, hexane/ethyl acetate 97:3) gave phosphoramidite **L16** as a white solid (28 mg, 99% yield). $[\alpha]_D^{20}$ = 566 (c = 0.1, CH_2Cl_2); ^1H NMR (400 MHz, C_6D_6) δ = 10.18-10.14 (m, 2H), 7.69 (dd, 2H, *J* = 8.0, 1.5 Hz), 7.62-7.56 (m, 1H), 7.53-7.35 (m, 9H), 6.96-6.86 (m, 2H), 6.83-6.72 (m, 8H), 2.09 (d, 6H, *J* = 9.5 Hz); ^{13}C NMR (101 MHz, C_6D_6) Note: due to the complexity of the spectrum, a simple list of peaks without couplings (except for the Me signals) is given. δ = 150.6, 150.5(3), 150.5(0), 141.9(5), 141.9(3), 141.8, 140.8, 140.7, 135.1, 135.0, 134.0, 133.6, 130.5, 130.4, 130.0(3), 129.9(9), 129.5, 129.4, 129.3, 129.0, 128.9, 128.8, 128.7, 128.6, 127.6, 127.4, 127.1, 126.9, 126.8, 126.7(4), 126.6(8), 126.4, 122.8(9), 122.8(6), 122.6, 35.4 (d, *J* = 21.1 Hz); ^{31}P (162 MHz, C_6D_6) δ = 143.0 (s); IR (ATR): ṽ = 3051, 2924, 2844, 1595,

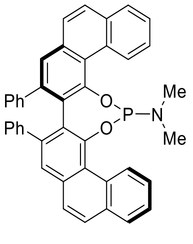

1556, 1485, 1383, 1327, 1231, 1126, 1018, 974, 875, 812, 741, 695, 496; LRMS m/z (EI$^+$) [M]$^+$ 611; HRMS (ESI$^+$, m/z) calculated for $[C_{42}H_{31}NO_2P]^+$ 612.20866, found 612.20869.

Representative Procedure C for Phosphoramidite Synthesis from PCl_3 and Amines

Phosphoramidite SI-L31. PCl_3 (8 µL, 0.093 mmol) in CH_2Cl_2 (0.75 mL) was added to a flame-dried Schlenk flask under argon. The resulting solution was cooled to 0 °C and triethylamine (65 µL, 0.464 mmol) was added. The cloudy solution was stirred for 30 min and then morpholine (8 µL, 0.093 mmol) was added. The resulting mixture was stirred at rt for 4 h. (*R*)-VAPOL (50 mg, 0.093 mmol) was then added and the resulting suspension was stirred for 15 h at rt, then filtered through silica (washed with CH_2Cl_2). The solvent was evaporated in vacuo to give a yellow solid. Purification by chromatography (SiO_2, hexane/ethyl acetate 95:5 to 9:1 gave phosphoramidite **SI-L31** as a yellow solid (44 mg, 73% yield). ^{1}H NMR (400 MHz, C_6D_6) δ = 10.18-10.11 (m, 2H), 7.74-7.67 (m, 2H), 7.66-7.60 (m, 1H), 7.54-7.39 (m, 9H), 6.93-6.86 (m, 2H), 6.79-6.73 (m, 8H), 3.11-3.03 (m, 2H), 2.96-2.82 (m, 4H), 2.54 (app. br. s, 2H); ^{13}C NMR (101 MHz, C_6D_6) Note: due to the complexity of the spectrum, a simple list of peaks without couplings (except for the alkyl signals) is given. δ = 150.4, 150.2, 150.1, 142.0(0), 141.9(9), 141.7, 140.7, 140.5, 135.1, 134.9, 134.0, 133.6, 130.5, 130.4, 130.0, 129.9, 129.4, 129.2, 129.0, 128.9, 128.1(8), 128.1(6), 127.9(1), 127.8(8), 127.6, 127.4(2), 127.3(8), 127.3(4), 127.2, 126.9(9), 126.9(6), 126.8, 126.7, 126.5, 122.8(0), 122.7(8), 122.5, 67.6 (d, *J* = 5.0 Hz), 44.9 (d, *J* = 18.0 Hz); ^{31}P (162 MHz, C_6D_6) δ = 143.4 (s); IR (ATR): ṽ = 3052, 2965, 2846, 1592, 1557, 1438, 1371, 1328, 1231, 1125, 1016, 961, 874, 812, 742, 696, 497; LRMS m/z (EI$^+$) [M]$^+$ 653; HRMS (ESI$^+$, m/z) calculated for $[C_{44}H_{33}NO_3P]^+$ 654.21945, found 654.21926.

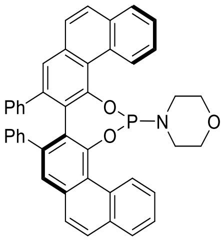

Phosphoramidite SI-L32. Prepared according to representative procedure C. Purification by flash chromatography (SiO_2, hexane/ethyl acetate 98:2) gave phosphoramidite **SI-L32** as an off-white solid (61 mg, 94% yield). ^{1}H NMR (400 MHz, C_6D_6) δ = 10.33 (d, *J* = 8.5 Hz, 1H), 10.22 (d, *J* = 8.5 Hz, 1H), 7.73-7.67 (m, 3H), 7.55-7.39 (m, 9H), 6.92-6.87 (m, 2H), 6.84-6.80 (m, 2H), 6.79-6.73 (m, 6H), 2.55 (ddd, *J* = 14.0, 9.5, 8.0 Hz, 2H), 2.39 (app. br. s, 2H), 1.60 (app. hept., *J* = 6.5 Hz, 2H), 0.86 (d, *J* = 6.5 Hz, 6H), 0.66 (d, *J* = 6.5 Hz, 6H); ^{13}C NMR (101 MHz, C_6D_6) Note: due to the complexity of the spectrum, a simple list of peaks without couplings is given for the aryl and methyl carbons. δ = 152.2(2), 151.1(8), 150.6, 141.9, 140.8, 140.6, 135.0(2), 134.9(9), 133.9, 133.6, 130.6, 130.5, 130.1, 130.0, 129.7, 129.6, 129.3, 129.0, 128.8, 128.6(4), 128.6(2), 127.9, 127.6, 127.4(0), 127.3(8), 127.1(1), 127.0(9), 126.9, 126.8(3), 126.7(5), 126.6(9), 126.0, 122.8(2), 122.8(1), 56.4 (d, *J* = 16.0 Hz), 28.1 (d, *J* = 5.0 Hz), 20.6(3), 20.6(2); ^{31}P (162 MHz, C_6D_6) δ = 147.4 (s); IR (ATR): ṽ = 3052, 2956, 2868, 1595,

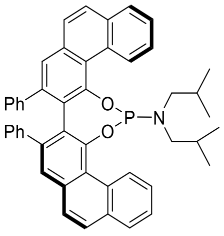

1556, 1371, 1233, 1011, 874, 813, 748, 696, 497; LRMS m/z (EI$^+$) [M]$^+$ 695; HRMS (ESI$^+$, m/z) calculated for [$C_{48}H_{43}NO_2P$]$^+$ 696.30223, found 696.30259.

Phosphoramidite SI-L33. Prepared according to representative procedure C. Purification by

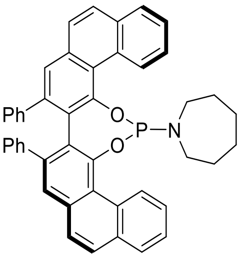

flash chromatography (SiO_2, hexane/ethyl acetate 98:2) gave phosphoramidite **SI-L33** as an off-white solid (30 mg, 49% yield). ^{1}H NMR (400 MHz, C_6D_6) δ = 10.09-10.02 (m, 2H), 7.55-7.51 (m, 2H), 7.49-7.44 (m, 1H), 7.37-7.20 (m, 9H), 6.73-6.67 (m, 2H), 6.62-6.53 (m, 8H), 2.73-2.61 (m, 2H), 2.53 (app. br. s, 2H), 1.24-1.14 (m, 2H), 1.14-1.07 (m, 4H), 1.04-0.93 (m, 2H); ^{13}C NMR (101 MHz, C_6D_6) Note: due to the complexity of the spectrum, a simple list of peaks without couplings is given except for alkyl carbons. δ = 149.9, 149.8, 149.7, 140.8(5), 140.8(3), 140.7, 139.7, 139.5, 133.8, 133.7, 132.8, 132.4, 129.5, 129.3, 128.8(2), 128.8(0), 128.3(1), 128.2(5), 128.1, 127.8, 127.6, 127.5, 127.3, 127.2, 127.0, 126.7, 126.5, 126.4, 126.1, 125.9, 125.7, 125.6, 125.5, 125.1, 121.6(4), 121.6(1), 121.5, 46.0 (d, *J* = 20.5 Hz), 29.9 (d, *J* = 3.5 Hz), 25.7; ^{31}P (162 MHz, C_6D_6) δ = 143.7 (s); IR (ATR): ṽ = 3052, 2955, 2866, 1595, 1556, 1371, 1327, 1232, 1047, 1012, 887, 812, 748, 696, 496; LRMS m/z (EI$^+$) [M]$^+$ 665; HRMS (ESI$^+$, m/z) calculated for [$C_{46}H_{36}NO_2PNa$]$^+$ 688.23759, found 688.23759.

Phosphoramidite SI-L34. Prepared according to representative procedure C. Purification by

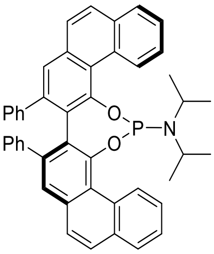

flash chromatography (SiO_2, hexane/ethyl acetate 98:2) gave phosphoramidite **SI-L34** as a light yellow solid (61 mg, 98% yield). $[\alpha]_D^{20}$ = –804 (c = 0.1, CH_2Cl_2); ^{1}H NMR (400 MHz, C_6D_6) Note: isopropyl signals were very broad, likely due to restricted rotation. δ = 10.28 (d, *J* = 8.5 Hz, 1H), 10.22 (d, *J* = 8.5 Hz, 1H), 7.74 (d, *J* = 8.0 Hz, 1H), 7.70 (d, *J* = 8.0 Hz, 1H), 7.62-7.57 (m, 1H), 7.56-7.38 (m, 9H), 6.92-6.81 (m, 4H), 6.78-6.71 (m, 6H), 3.26 (br. s, 2H), 1.24 (br. s, 12H); ^{13}C NMR (101 MHz, C_6D_6) Note: due to the complexity of the spectrum, a simple list of peaks without couplings is given. The isopropyl carbon peaks were unusually very broad. δ = 151.4, 151.2, 151.1, 141.9(9), 141.9(8), 140.9, 140.7, 135.0, 134.7, 133.9, 133.4, 131.0, 130.6, 130.1, 130.0, 129.6, 129.5, 129.4, 129.3, 128.7(3), 128.7(1), 128.6, 128.4(9), 128.4(7), 128.2, 127.9, 127.7, 127.6(2), 127.6(0), 127.5, 127.2, 127.1, 127.0, 126.9, 126.7, 126.6, 125.8, 122.7(3), 122.7(1), 122.6, 45.6 (br. s), 25.8 (br. s); ^{31}P (162 MHz, C_6D_6) δ = 146.3 (s); IR (ATR): ṽ = 3052, 2967, 2930, 1592, 1563, 1487, 1364, 1173, 1122, 1023, 975, 876, 748, 696, 496; LRMS m/z (EI$^+$) [M]$^+$ 667; HRMS (ESI$^+$, m/z) calculated for [$C_{46}H_{38}NO_2P$]$^+$ 668.27158, found 668.27129.

Phosphoramidite SI-L35. Prepared according to representative procedure C. Purification by

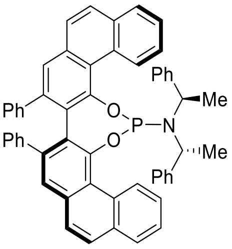

flash chromatography (SiO_2, hexane/ethyl acetate 98:2) gave phosphoramidite **SI-L35** as a white solid (70 mg, 95% yield). $[\alpha]_D^{20} = -281$ (c = 0.1, CH_2Cl_2); ^1H NMR (400 MHz, C_6D_6) Note: □-methylbenzylamine peaks were very broad, likely due to restricted rotation. δ = 10.18 (d, *J* = 8.5 Hz, 1H), 9.78 (d, *J* = 8.0 Hz, 1H), 7.77 (d, *J* = 8.0 Hz, 1H), 7.72-7.63 (m, 2H), 7.57-7.49 (m, 2H), 7.49-7.38 (m, 4H), 7.31 (t, *J* = 4.5 Hz, 2H), 7.29-7.23 (m, 1H), 7.00-6.61 (m, 20H), 4.44 (br. s, 2H), 1.68 (br. s, 6H); ^{13}C NMR (101 MHz, C_6D_6) Note: due to the complexity of the spectrum, a simple list of peaks without couplings is given. δ = 150.9, 150.8, 142.2, 142.1, 141.9(7), 141.9(6), 140.8, 140.6, 135.0, 134.6(9), 134.6(8), 133.9, 133.5, 130.9, 130.4, 130.3, 130.0, 129.4, 128.9, 128.8, 128.7, 128.6, 128.5(3), 128.5(0), 128.4(8), 127.8, 127.7, 127.5, 127.3, 127.1, 127.0, 126.9, 126.8, 126.7(0), 126.6(6), 126.6(3), 126.5, 125.6, 122.6(3), 122.6(1), 55.5, 55.4; ^{31}P (162 MHz, C_6D_6) δ = 148.7 (s); IR (ATR): ṽ = 3052, 2965, 2929, 1595, 1556, 1488, 1422, 1371, 1232, 1125, 1017, 875, 812, 742, 695, 497; LRMS m/z (EI^+) $[M]^+$ 791; HRMS (ESI^+, m/z) calculated for $[C_{56}H_{43}NO_2P]^+$ 792.3023, found 792.3026.

Note: use of the opposite (S,S)-amine enantiomer with (R)-VAPOL was attempted twice using this procedure but failed to give any product.

Phosphoramidite SI-L36. 2,2,6,6-Tetramethylpiperidine (19.5 µL, 0.116 mmol) was added to

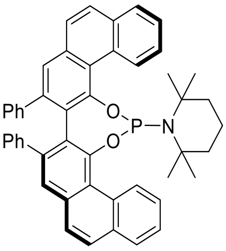

a Schlenk flask under argon. THF (0.58 mL) was added and the solution was cooled to 0 °C. *n*-Butyllithium (70 µL, 0.116 mmol, 1.6 M in hexanes) was added dropwise and stirred for 30 mins. PCl_3 (30 µL, 0.35 mmol) in THF (0.3 mL) was added in one portion and the reaction mixture was stirred at rt for 1 hour. Excess PCl_3 was removed *in vacuo* at room temperature using a low vacuum pump attached to the Schlenk flask through an intermediate liquid nitrogen trap. Dry THF (0.87 mL) was added to the residue and stirred for 10 min at rt. The mixture was cooled to 0 °C. A solution of (*R*)-VAPOL (50 mg, 0.093 mmol) and triethylamine (40 µL, 0.283 mmol) in dry THF (1 mL) was added. The reaction was then warmed to rt and stirred overnight. The reaction mixture was filtered through Celite (washed with toluene) and the solvent was evaporated. Purification by flash chromatography (SiO_2, hexane/ethyl acetate 98:2) gave phosphoramidite **SI-L36** as a white solid (60 mg, 91% yield). $[\alpha]_D^{20} = -618$ (c = 0.1, CH_2Cl_2); ^1H NMR (400 MHz, C_6D_6) δ = 10.48 (d, *J* = 8.5 Hz, 1H), 10.34 (d, *J* = 8.5 Hz, 1H), 7.77-7.72 (m, 2H), 7.68-7.61 (m, 2H), 7.57-7.39 (m, 8H), 6.91-6.84 (m, 4H), 6.79-6.71 (m, 6H), 1.91 (d, *J* = 5.5 Hz, 3H), 1.63 (d, *J* = 8.0 Hz, 6H), 1.53-1.34 (m, 3H), 1.13-1.05 (m, 1H), 0.96 (s, 3H), 0.91-0.84 (m, 2H); ^{13}C NMR (101 MHz, C_6D_6) Note: due to the complexity of the spectrum, a simple list of peaks without couplings is given. δ = 152.5, 152.4, 151.3, 151.2, 141.4(2), 141.4(0),

141.2(6), 141.2(5), 140.0, 139.6, 133.8(8), 133.8(7), 133.6(2), 133.6(1), 132.7, 132.5, 129.8, 129.6, 129.5, 129.2, 129.1, 129.0, 128.4, 128.1, 127.7, 127.6(1), 127.5(9), 127.5(0), 127.4(7), 126.6, 126.5, 125.8, 125.7, 125.5(3), 125.5(1), 125.4(9), 125.4(7), 124.0, 121.7(3), 121.7(0), 121.6(7), 56.4, 56.0, 55.8, 55.6, 41.2, 40.9, 40.8, 35.1, 34.6, 31.4, 31.3, 31.2, 28.7, 16.2; ^{31}P (162 MHz, C_6D_6) δ = 155.9 (s); IR (ATR): ṽ = 3051, 2962, 2927, 1595, 1565, 1422, 1369, 1328, 1231, 1126, 1018, 874, 811, 747, 695, 496; LRMS m/z (EI^+) $[M]^+$ 707; HRMS (ESI^+, m/z) calculated for $[C_{49}H_{43}NO_2P]^+$ 708.30294, found 708.30259.

Phosphoramidite SI-L37. Prepared according to representative procedure C. Purification by flash chromatography (SiO_2, hexane/ethyl acetate 98:2) gave phosphoramidite **SI-L37** as an off-white solid (31 mg, 49% yield). ^{1}H NMR (400 MHz, C_6D_6) δ =10.29 (d, *J* = 8.0 Hz, 1H), 10.25 (dd, *J* = 8.0, 1.5 Hz, 1H), 7.77-7.72 (m, 3H), 7.54 (dd, *J* = 9.0, 4.5 Hz, 2H), 7.50-7.42 (m, 7H), 7.14-7.09 (m, 3H), 7.07-7.02 (m, 1H), 6.93-6.87 (m, 2H), 6.80-6.73 (m, 8H), 3.92 (app. br. s, 1H), 3.73 (app. br. s, 1H), 2.05 (d, *J* = 7.5 Hz, 3H); ^{13}C NMR (101 MHz, C_6D_6) Note: due to the complexity of the spectrum, a simple list of peaks without couplings is given. δ = 150.6, 150.5, 150.4, 142.0(1), 142.0(0), 141.9, 140.7, 140.6, 138.5, 138.4, 135.1, 135.0, 134.0, 133.7, 130.5(3), 130.4(6), 130.0(3), 129.9(7), 129.4, 129.1, 128.9, 128.7, 128.5(1), 128.4(9), 128.2, 127.9, 127.6(4), 127.5(6), 127.5(1), 127.4, 127.3, 127.2, 127.0, 126.9, 126.7, 126.6, 122.9, 122.8, 122.6, 52.4, 31.8; ^{31}P (162 MHz, C_6D_6) δ = 141.1 (s); IR (ATR): ṽ = 3052, 2956, 2868, 1595, 1556, 1466, 1371, 1232, 1012, 874, 813, 748, 696, 498; LRMS m/z (EI^+) $[M]^+$ 687; HRMS (ESI^+, m/z) calculated for $[C_{48}H_{35}NO_2P]^+$ 688.24008, found 688.23999.

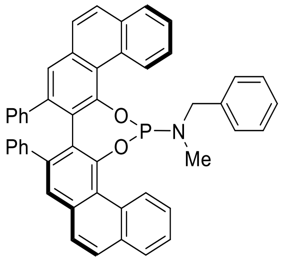

Phosphoramidite SI-L38. Prepared according to representative procedure C, except PCl_3 was added to a solution of the amine and triethylamine with subsequent stirring for 3 h at 0 °C. Purification by flash chromatography (SiO_2, hexane/ethyl acetate 97:3 to 95:5) gave phosphoramidite **SI-L38** as an off-white solid (69 mg, 96% yield). $[\alpha]_D^{20}$ = -600 (c = 0.1, CH_2Cl_2); ^{1}H NMR (400 MHz, C_6D_6) δ = 10.43 (d, *J* = 8.5 Hz, 1H), 10.15 (dd, *J* = 8.0, 1.5 Hz, 1H), 7.82-7.69 (m, 3H), 7.56-7.48 (m, 4H), 7.47-7.41 (m, 2H), 7.40-7.34 (m, 3H), 7.31 (s, 1H), 6.93-6.78 (m, 6H), 6.77-6.66 (m, 7H), 6.63-6.57 (m, 3H), 6.52-6.47 (m, 2H), 5.88 (d, *J* = 11.5 Hz, 1H); ^{13}C NMR (101 MHz, C_6D_6) Note: due to the complexity of the spectrum, a simple list of peaks without couplings is given. δ = 149.9, 149.8, 143.0, 142.9, 142.0, 141.9(7), 141.9(5), 141.9(3), 141.9(2), 141.9(1), 140.7, 140.5, 136.6(8), 136.6(5), 135.9(9), 135.9(8), 134.9, 134.6(7), 134.6(6), 133.9, 133.6, 131.4, 130.8, 130.7, 130.2, 130.0(9), 130.0(6), 130.0(1), 129.6, 129.5, 129.4(1), 129.3(7), 129.3, 129.1, 128.9, 128.8(1), 128.7(9), 128.7(5), 128.7(1),128.6(5), 128.5(9), 128.4, 128.2, 127.9, 127.7, 127.6, 127.4(2), 127.3(8), 127.2, 127.1, 127.0, 126.8(2), 126.7(7), 126.7, 126.6, 126.5, 125.9, 122.6, 122.5(6),

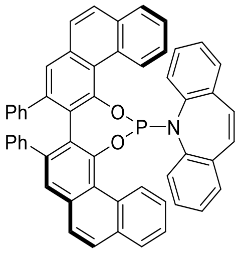

122.5(4); ^{31}P (162 MHz, C_6D_6) δ = 134.7 (s); IR (ATR): ṽ = 3051, 1595, 1555, 1485, 1372, 1232, 1125, 1107, 1017, 876, 742, 695, 496; LRMS m/z (EI$^+$) [M]$^+$ 759; HRMS (ESI$^+$, m/z) calculated for $[C_{54}H_{35}NO_2P]^+$ 760.2392, found 760.2400.

1.3.2 Ni-catalysed Reductive Coupling of Dienes and Aldehydes

1.3.2.1 Racemic Scope

Representative Procedure D for Virtually Racemic Diol Synthesis with L6. Ni(cod)$_2$ (6.9 mg, 0.025 mmol) and cyclodiphosphazane ligand **L6** (8.8 mg, 0.012 mmol) were added to a flame-dried Schlenk flask under argon and dissolved in toluene 1.0 mL). Diene **7** (265 mg, 1.10 mmol) was added and the mixture stirred for 5 mins. Hydrocinnamaldehyde (134 mg, 1.0 mmol) and triethylborane (1.2 mL, 1 M in hexanes, 1.2 mmol) were added, the flask was sealed and the reaction stirred for 24 h at rt. The reaction mixture was quenched with sat. aq. $NaHCO_3$ solution (0.5 mL) and stirred for 15 min at rt. The mixture was diluted with methyl *tert*-butyl ether, the organic phase was separated and the aqueous layer was extracted with methyl *tert*-butyl ether twice. The combined organic layers were dried over anhydrous $MgSO_4$, filtered and concentrated *in vacuo*. Purification by flash chromatography (SiO_2, hexane/*tert*-butyl methyl ether 100:0 to 95:5) gave the diol **9a** as a colourless oil (353 mg, 93% yield, 96:4 dr).

For detailed notes and troubleshooting, please see the procedure for the enantioselective reaction (page S44).

***anti*-6-Methyl-1-phenyl-4-((triisopropylsilyl)oxy)hept-6-en-3-ol 9a.** *Rf* = 0.60 (hexane/*tert*-butyl methyl ether = 95/5). ^{1}H NMR (400 MHz, $CDCl_3$) δ 7.30-7.26 (m, 2H), 7.22-7.15 (m, 3H), 4.76 (q, *J* = 1.7 Hz, 1H), 4.71 (dq, *J* = 2.0, 1.0 Hz, 1H), 4.07 (ddd, *J* = 7.4, 6.3, 2.8 Hz, 1H), 3.61 (dt, *J* = 9.2, 3.2 Hz, 1H), 2.93 (ddd, *J* = 14.3, 9.3, 5.4 Hz, 1H), 2.68 (ddd, *J* = 13.7, 9.1, 7.5 Hz, 1H), 2.23-2.22 (m, 2H), 1.83-1.74 (m, 2H), 1.72 (br. s, 3H), 1.07-1.04 (m, 21H). ^{13}C NMR (100 MHz, $CDCl_3$) δ 142.4, 142.2, 128.6, 128.5, 125.9, 113.4, 74.1, 73.8, 41.3, 32.9, 32.5, 22.9, 18.32, 18.30, 12.9. IR (ATR): ṽ = 3580, 3027, 2942, 2893, 2865, 1649, 1604, 1496, 1455, 1381, 1333, 1280, 1247, 1212, 1115, 1086, 1065, 1031, 1013, 998, 967, 920, 882, 805, 744, 698, 677, 583, 559, 535, 497, 467, 441, 412. HRMS (ESI) m/z calcd for $C_{23}H_{40}O_2SiNa$: 399.2690 [M+Na]$^+$, found 399.2689.

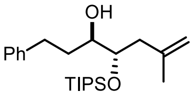

***trans*-4-((*tert*-Butyldimethylsilyl)oxy)-6-methyl-1-phenylhept-6-en-3-ol 9b.** Prepared according to representative procedure D. The residue was purified by flash chromatography over silica gel (hexane/*tert*-butyl methyl ether = 100:0 to 95:5) to give **9b** as a colorless oil (86 mg, 68%, 95:5 dr). *Rf* = 0.65 (hexane/ *tert* -butyl methyl ether = 95/5). ^{1}H NMR (400 MHz, $CDCl_3$) δ 7.31-7.26 (m, 2H), 7.23-7.16 (m, 3H), 4.78 (dq, *J* = 3.0, 1.5 Hz, 1H), 4.71 (dq, *J* = 2.0, 1.0 Hz, 1H), 3.82 (ddd, *J* = 7.7, 5.3, 3.3 Hz, 1H), 3.63-

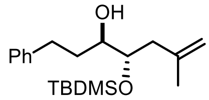

3.57 (m, 1H), 2.88 (ddd, *J* = 13.8, 9.0, 6.2 Hz, 1H), 2.69-2.59 (m, 1H), 2.26 (ddd, *J* = 13.7, 7.8, 1.0 Hz, 1H), 2.15 (ddd, *J* = 13.8, 5.4, 1.1 Hz, 1H), 1.78-1.72 (m, 2H), 1,72 (br. s, 3H), 1.88 (s, 9H), 0.04 (s, 6H). ^{13}C NMR (100 MHz, $CDCl_3$) δ 142.4, 142.3, 128.6, 128.5, 125.9, 113.6, 74.1, 73.9, 40.4, 33.4, 32.6, 26.0, 22.9, 18.2, -4.3, -4.5. IR (ATR): ṽ = 3575, 3476, 3064, 3027, 2952, 2929, 2894, 2856, 1649, 1604, 1471, 1455, 1376, 1361, 1253, 1070, 1032, 1004, 981, 931, 889, 933, 810, 774, 745, 698, 672, 536, 468, 450. HRMS (ESI) m/z calcd for $C_{20}H_{34}O_2SiNa$: 357.2220 [M+Na]$^+$, found 357.2220.

***trans*-6-Methyl-1-phenyl-4-((triethylsilyl)oxy)hept-6-en-3-ol 9c.** Prepared according to representative procedure D. The residue was purified by flash chromatography over silica gel (hexane/*tert*-butyl methyl ether = 100:0 to 95:5) to give **9c** as a colorless liquid (82 mg, 65%, 95:5 dr). *Rf* = 0.55 (hexane *tert*-butyl methyl ether = 95/5). ^{1}H NMR (400 MHz, $CDCl_3$) δ 7.31-7.26 (m, 2H), 7.23-7.16 (m, 3H), 4.78 (dt, *J* = 3.0, 1.5 Hz, 1H), 4.73-4.70 (m, 1H), 3.84 (ddd, *J* = 7.5, 5.6, 3.3 Hz, 1H), 3.61-3.56 (m, 1H), 2.92 (ddd, *J* = 14.5, 9.1, 5.9 Hz, 1H), 2.68 (ddd, *J* = 13.7, 9.1, 7.5 Hz, 1H), 2.25 (ddd, *J* = 13.8, 7.6, 1.0 Hz, 1H), 2.75 (ddd, *J* = 13.8, 5.7, 1.1 Hz, 1H), 1.78-1.73 (m, 2H), 1.73 (br. s, 3H), 0.96 (t, *J* = 7.9 Hz, 9H), 0.61 (q, *J* = 8.1 Hz, 6H). ^{13}C NMR (100 MHz, $CDCl_3$) δ 142.5, 142.3, 128.6, 128.5, 125.9, 113.3, 74.2, 73.9, 40.5, 33.4, 32.5, 23.0, 7.0, 5.2. IR (ATR): ṽ = 3569, 3472, 3064, 3027, 2953, 2912, 2876, 1649, 1604, 1585, 1496, 1455, 1414, 1377, 1333, 1292, 1238, 1113, 1071, 1031, 1004, 979, 926, 889, 850, 810, 766, 738, 725, 698, 589, 567, 536, 501, 468, 453, 437.HRMS (ESI) m/z calcd for $C_{20}H_{34}O_2SiNa$: 357.2220 [M+Na]$^+$, found 357.2220.

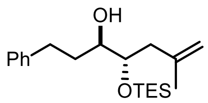

***trans*-2-Methyl-4-((triisopropylsilyl)oxy)non-1-en-5-ol 9d.** Prepared according to representative procedure D. The residue was purified by flash chromatography over silica gel (hexane/*tert*-butyl methyl ether = 100:0 to 95:5) to give **9d** as a colorless oil (102 mg, 82%, 98:2 dr). *Rf* = 0.55 (hexane/*tert*-butyl methyl ether = 95/5). ^{1}H NMR (400 MHz, $CDCl_3$) δ 4.80-4.77 (m, 1H), 4.77-4.75 (m, 1H), 4.07 (td, *J* = 6.7, 2.9 Hz, 1H), 4.65-3.99 (m, 1H), 2.28 (br d, *J* = 6.7 Hz, 2H), 1.76 (t, *J* = 1.1 Hz, 3H), 1.50-1.27 (m, 6H), 1.10-1.06 (m, 21H), 0.92 (t, *J* = 7.1 Hz, 3H). ^{13}C NMR (100 MHz, $CDCl_3$) δ 142.5, 113.3, 74.8, 74.1, 41.0, 30.9, 28.5, 23.0, 22.9, 18.3, 18.3, 14.20, 12.91. IR (ATR): ṽ = 3583, 3491, 3076, 2942, 2894, 2866, 1649, 1463, 1379, 1257, 1202, 1050, 1035, 1010, 997, 952, 919, 882, 791, 749, 676, 685, 554, 507, 463.HRMS (ESI) m/z calcd for $C_{19}H_{40}O_2SiNa$: 351.2689 [M+Na]$^+$, found 351.2689.

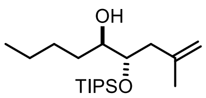

***trans*-2,6-Dimethyl-4-((triisopropylsilyl)oxy)hept-6-en-3-ol 9e.** Prepared according to representative procedure D. The residue was purified by flash chromatography over silica gel (hexane/*tert*-butyl methyl ether = 100:0 to 95:5) to give **9e** as a colorless oil (83 mg, 70%, 99:1 dr). *Rf* = 0.45 (hexane/*tert*-butyl methyl ether = 95/5). ^{1}H NMR (400 MHz, $CDCl_3$) δ 4.82-4.78 (m, 2H), 4.16 (ddd, *J* = 6.9, 5.5, 3.1 Hz, 1H), 3.33 (dd, *J* = 8.4,

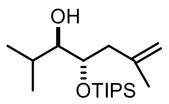

3.1 Hz, 1H), 2.41 (br. s, 1H), 2.26-2.25 (m, 1H), 2.25-2.23 (m, 1H), 1.79-1.78 (m, 3H), 1.78-1.69 (m, 1H), 1.09-1.07 (m, 21H), 1.04 (d, *J* = 6.6 Hz, 3H), 0.88 (d, *J* = 6.8 Hz, 3H). ^{13}C NMR (100 MHz, $CDCl_3$) δ 142.6, 113.6, 80.6, 72.4, 39.5, 29.8, 23.2, 19.7, 18.9, 18.3, 12.9. IR (ATR): ṽ = 3581, 3076, 2936, 2944, 2867, 1649, 1463, 1383, 1366, 1333, 1307, 1246, 1119, 1085, 1057, 982, 999, 950, 927, 882, 802, 745, 675, 651, 563, 533, 507, 462, 407. HRMS (ESI) m/z calcd for $C_{18}H_{38}O_2SiNa$: 337.2533 [M+Na]$^+$, found 337.2533.

***trans*-1-Cyclopropyl-4-methyl-2-((triisopropylsilyl)oxy)pent-4-en-1-ol 9f.** Prepared

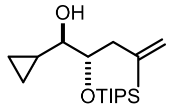

according to representative procedure D. The residue was purified by flash chromatography over silica gel (hexane/*tert*-butyl methyl ether = 100:0 to 95:5) to give **9f** as a colorless oil (75 mg, 64%, 99:1 dr). *Rf* = 0.45 (hexane/*tert*-butyl methyl ether = 90/10). ^{1}H NMR (400 MHz, $CDCl_3$) δ 4.80-4.77 (m, 2H), 4.20 (ddd, *J* = 8.0, 5.6, 2.6 Hz, 1H), 2.96 (dd, *J* = 8.8, 2.6 Hz, 1H), 2.57 (br dd, *J* = 14.5, 7.7 Hz, 1H), 2.32 (br dd, *J* = 14.5, 5.5 Hz, 1H), 2.22 (br. s, 1H), 1.78-1.76 (m, 3H), 1.10-1.06 (m, 21H), 1.06-0.97 (m, 1H), 0.58-0.45 (m, 2H), 0.37-0.30 (m, 1H), 0.28-0.21 (m, 1H). ^{13}C NMR (100 MHz, $CDCl_3$) δ 142.6, 113.0, 79.4, 73.9, 41.1, 23.1, 18.3, 18.3, 12.9, 12.0, 2.8, 2.7. IR (ATR): ṽ = 3569, 3482, 3079, 3006, 2943, 2893, 2866, 1648, 1463, 1381, 1309, 1246, 1195, 1108, 1061, 1013, 917, 882, 823, 786, 744, 675, 653, 564, 461. HRMS (ESI) m/z calcd for $C_{18}H_{36}O_2SiNa$: 335.2380 [M+Na]$^+$, found 335.2377.

***trans*-2,2,6-Trimethyl-4-((triisopropylsilyl)oxy)hept-6-en-3-ol 9g.** Prepared according to

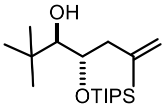

representative procedure D. The residue was purified by flash chromatography over silica gel (hexane/*tert*-butyl methyl ether = 100:0 to 95:5) to give **9g** as a colorless liquid (49 mg, 40%, 99:1 dr). *Rf* = 0.50 (hexane/*tert*-butyl methyl ether = 95/5). ^{1}H NMR (400 MHz, $CDCl_3$) δ 4.82-4.77 (m, 2H), 4.20 (ddd, *J* = 8.6, 3.3, 2.3 Hz, 1H), 3.50 (d, *J* = 2.3 Hz, 1H), 2.34 (br dd, *J* = 14.6, 3.3 Hz, 1H), 2.27 (br. s, 1H), 2.26 (ddd, *J* = 14.6, 8.6, 0.9 Hz, 1H), 1.77-1.76 (m, 3H), 1.08-1.06 (m, 21H), 0.99 (s, 9H). ^{13}C NMR (100 MHz, $CDCl_3$) δ 142.9, 113.1, 83.1, 72.7, 40.2, 33.8, 27.4, 23.5, 18.4, 13.1. IR (ATR): ṽ = 3584, 3077, 2944, 2893, 2867, 1650, 1464, 1378, 1364, 1326, 1294, 1259, 1239, 1206, 1177, 1143, 1080, 1058, 1025, 1014, 997, 958, 947, 932, 882, 845, 804, 727, 675, 651, 567, 548, 530, 517, 496, 465, 445. HRMS (ESI) m/z calcd for $C_{19}H_{40}O_2SiNa$: 351.2689 [M+Na]$^+$, found 351.2689.

***trans*-2,7,11-Trimethyl-4-((triisopropylsilyl)oxy)dodeca-1,10-dien-5-ol 9h.** Prepared

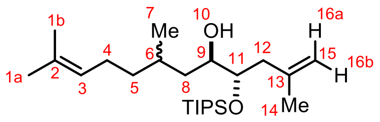

according to representative procedure D, except stirred for 30 h. The residue was purified by flash chromatography over silica gel (hexane/*tert*-butyl methyl ether = 100:0 to 95:5) to give **9h** as a colorless oil (121 mg, 81%, 65:35 dr). *Rf* = 0.60 (hexane/*tert*-butyl methyl ether = 95/5). Traces of pure both diastereoisomers have been isolated by preparative HPLC. ^{1}H NMR (400 MHz, $CDCl_3$) δ (*major*) 5.14 (tdt, *J* = 5.7, 2.8, 1.4 Hz, 1H, H3), 4.81-4.77 (m, 1H, H16b), 4.77-

4.73 (m, 1H, H16a), 4.07 (td, J = 6.9, 2.8 Hz, 1H, H11), 3.74 (dt, J = 10.4, 2.7 Hz, 1H, H9), 2.29 (br d, J = 6.8 Hz, 2H, H12), 2.06-1.89 (m, 3H, H10, H4), 1.76 (br. s, 3H, H14), 1.73-1.64 (m, 1H, H6), 1.68 (br d, J = 1.0 Hz, 3H, H1a), 1.60 (br. s, 3H, H1b), 1.57 (ddd, J = 14.0, 10.3, 3.7 Hz, 1H, H8a), 1.37-1.19 (m, 2H, H5), 1.15-1.08 (m, 1H, H8b), 1.10-1.06 (m, 21H), 0.89 (d, J = 6.6 Hz, 3H, H7). (*minor*) 5.12 (tdt, J = 7.0, 2.9, 1.4 Hz, 1H, H3), 4.81-4.77 (m, 1H, H16b), 4.77-4.74 (m, 1H, H16a), 4.05 (td, J = 6.7, 2.8 Hz, 1H, H11), 3.75 (ddd, J = 7.4, 5.6, 2.8 Hz, 1H, H9), 2.27 (br d, J = 6.6 Hz, 2H, H12), 2.07-1.87 (m, 3H, H10, H4), 1.76 (br. s, 3H, H14), 1.68 (br d, J = 1.0 Hz, 3H, H1a), 1.68-1.60 (m, 1H, H6), 1.60 (br. s, 3H, H1b), 1.45-1.38 (m, 1H, H5a), 1.38-1.33 (m, 2H, H8), 1.15-1.08 (m, 1H, H5b), 1.10-1.06 (m, 21H), 0.96 (d, J = 6.7 Hz, 3H, H7). ^{13}C NMR (100 MHz, $CDCl_3$) δ (*major*) 142.3 (C15), 131.2 (C2), 125.0 (C3), 113.3 (C15), 74.5 (C11), 72.3 (C9), 41.4 (C12), 38.4 (C5), 38.0 (C8), 29.0 (C6), 25.9 (C1a), 25.8 (C4), 22.9 (C14), 19.2 (C7), 18.35 (CH_3-TIPS), 18.33 (CH_3-TIPS), 17.8 (C1b), 12.9 (CH-TIPS). (*minor*) 142.4 (C15), 131.3 (C2), 125.0 (C3), 113.4 (C15), 74.2 (C11), 72.9 (C9), 40.9 (C12), 38.5 (C8), 36.6 (C5), 29.5 (C6), 25.9 (C1a), 25.5 (C4), 23.0 (C14), 20.8 (C7), 18.35 (CH_3-TIPS), 18.34 (CH_3-TIPS), 17.8 (C1b), 12.9 (CH-TIPS). IR (ATR): ṽ = 3583, 3076, 2943, 2948, 2866, 1649, 1462, 1377, 1291, 1256, 1247, 1083, 1075, 1064, 1013, 997, 920, 883, 770, 748, 677, 688, 549, 504, 463, 440. HRMS (ESI) m/z calcd for $C_{24}H_{48}O_2SiNa$: 419.3314 $[M+Na]^+$, found 419.3316.

***trans*-1-Phenyl-4-((triisopropylsilyl)oxy)hept-6-en-3-ol 9i.** Prepared according to

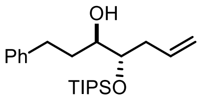

representative procedure D. Purified by flash chromatography over silica gel (hexane/*tert*-butyl methyl ether = 100/0 to 95/5) to give **9i** as a colorless oil (104 mg, 76%, 87:13 dr). *Rf* = 0.55 (hexane/*tert*-butyl methyl ether = 95/5). ^{1}H NMR (400 MHz, $CDCl_3$) δ (*major*) 7.30-7.26 (m, 2H), 7.23-7.16 (m, 3H), 5.88 (ddt, J = 17.2, 10.1, 7.1 Hz, 1H), 5.08 (br ddt, J = 17.1, 3.4, 1.5 Hz, 1H), 5.03 (ddt, J = 10.2, 2.2, 1.2 Hz, 1H), 3.88 (td, J = 6.1, 3.2 Hz, 1H), 3.68 (dt, J = 9.4, 3.3 Hz, 1H), 2.92 (ddd, J = 14.4, 9.6, 5.2 Hz, 1H), 2.69 (ddd, J = 13.8, 9.3, 7.2 Hz, 1H), 2.39-2.25 (m, 2H), 2.20 (br. s, 1H), 1.85-1.66 (m, 2H), 1.10-1.07 (m, 3H), 1.06-1.04 (m, 18H). ^{13}C NMR (100 MHz, $CDCl_3$) δ (*major*) 142.2, 135.2, 128.6, 128.5, 125.9, 117.1, 75.4, 73.9, 36.9, 33.4, 32.6, 18.3, 12.7. IR (ATR): ṽ = 3581, 3478, 3106, 3076, 3064, 3027, 2942, 2892, 2866, 1641, 1604, 1496, 1462, 1414, 1384, 1368, 1331, 1287, 1248, 1208, 1086, 1065, 1032, 1013, 996, 913, 881, 820, 744, 689, 677, 586, 566, 505, 469, 446. HRMS (ESI) m/z calcd for $C_{22}H_{38}O_2SiNa$: 385.2535 $[M+Na]^+$, found 385.2533.

***anti*-4-((*tert*-Butyldimethylsilyl)oxy)undec-1-en-5-ol 9j**. To a blood red solution of $Ni(COD)_2$

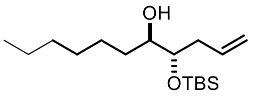

(7.4 mg, 0.027 mmol) and **L4** (19 mg, 0.027 mmol) in toluene (2.15 mL) was added the dienolsilane **115** (218 mg, 1.18 mmol) causing a darkening of the solution to a brown/red colour. Triethylborane (1.6 mL, 1 M in hexanes, 1.6 mmol) was added followed by heptanal (0.15 mL, 1.08 mmol). The red reaction mixture was stirred at rt for 16 hours. The reaction was quenched with the addition of $NaHCO_3$ solution and

stirred for 30 minutes before the organic phase was separated, and the aqueous phase was extracted with *tert*-butyl methyl ether (3 x 10 mL). The combined organic phases were washed with brine solution and dried over Na_2SO_4, filtered and concentrated to give a cloudy yellow oil. The residue was purified by flash chromatography (hexane/ethyl acetate 95:5 to 9:1) to give **9j** as a colourless oil (150 mg, 46%). ^{1}H NMR (400 MHz, $CDCl_3$) *δ* 5.83 (ddt, *J* = 17.2, 10.1, 7.2 Hz, 1H), 5.11–5.00 (m, 2H), 3.65 (ddd, *J* = 7.5, 4.6, 3.5 Hz, 1H), 3.61–3.53 (m, 1H), 2.35– 2.25 (m, 1H), 2.19 (dddt, *J* = 14.3, 7.3, 4.6, 1.3 Hz, 1H), 2.09 (d, *J* = 3.6 Hz, 1H), 1.41 (dd, *J* = 13.4, 6.5 Hz, 2H), 1.35–1.22 (m, 6H), 0.94–0.82 (m, 13H), 0.07 (s, 6H). ^{13}C NMR (101 MHz, $CDCl_3$) *δ* 135.6, 116.8, 75.1, 74.6, 35.9, 31.9, 31.8, 29.4, 26.1, 25.8 (2C), 22.6, 18.1, 14.1, –4.3, –4.6 ppm. Matches known data for *anti*-4-((*tert*-butyldimethylsilyl)oxy)undec-1-en-5-ol.[10]

Procedure for the reaction with octenal (**9k** and **iso-9k**). To a blood red solution of $Ni(COD)_2$ (8 mg, 0.03 mmol) and **L4** (21 mg, 0.03 mmol) in toluene (1.83 mL) was added the dienolsilane **S13** (430 mg, 2.33 mmol) causing a darkening of the solution to a brown/red colour. Triethylborane (1.75 mL, 1 M in hexanes, 1.75 mmol) was added followed by dropwise addition of *trans*-2-octenal (0.174 mL, 1.17 mmol) in toluene (0.5 mL) over 16 hours. Stirring was continued for a total of 106 hours at rt. The reaction was quenched with the addition of $NaHCO_3$ solution and the mixture stirred for 30 minutes before the organic phase was separated, and the aqueous phase was extracted with *tert*-butyl methyl ether (3 x 10 mL). The combined organic phases were washed with brine solution and dried over Na_2SO_4, filtered and concentrated to give a cloudy yellow oil. The residue was purified by flash chromatography (hexane/ethyl acetate 95:5 to 9:1) to give the product **9k** as a colourless oil (167 mg, 46%) and the regiosomer ***iso*-9k** as a colourless oil (40 mg, 11%).

(*anti*,*E*)-4-((*tert*-Butyldimethylsilyl)oxy)dodeca-1,6-dien-5-ol 9k. ^{1}H NMR (400 MHz,

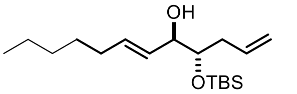

$CDCl_3$) δ 5.84–5.64 (m, 2H), 5.45 (ddt, *J* = 15.5, 7.1, 1.4 Hz, 1H), 5.08–4.98 (m, 2H), 4.03 (dt, *J* = 7.2, 3.6 Hz, 1H), 3.70 (ddd, *J* = 7.0, 5.5, 3.7 Hz, 1H), 2.34–2.17 (m, 2H), 2.15 (br d, *J* = 3.9 Hz, 1H), 2.10–1.98 (m, 2H), 1.42–1.20 (m, 6H), 0.94–0.80 (m, 12H), 0.07 (s, 3H), 0.07 (s, 3H). ^{13}C NMR (101 MHz, CDCl3) δ 135.2, 134.0, 128.0, 116.9, 75.4, 75.3, 36.9, 32.4, 31.4, 28.7, 25.8, 22.5, 18.1, 14.0, –4.3, –4.6 ppm. IR (ATR): ṽ = 3460, 2955, 2927, 2856, 1463, 1361, 1253, 1087, 1004, 972, 912, 825, 760, 676. HRMS (ESI$^-$): m/z calcd. for $C_{18}H_{35}O_2Si$: 311.2412, found: 311.2416.

Regioisomer *iso*-9k. ^{1}H NMR (400 MHz, $CDCl_3$) δ 6.24 (dt, *J* = 11.9, 1.3 Hz, 1H), 5.63 (dtd, *J*

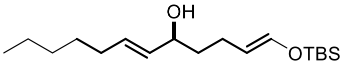

= 15.3, 6.7, 0.9 Hz, 1H), 5.44 (ddt, *J* = 15.4, 7.1, 1.4 Hz, 1H), 4.98 (dt, *J* = 11.9, 7.5 Hz, 1H), 4.06 (q, *J* = 6.8 Hz, 1H), 2.07–1.99 (m, 2H), 1.95 (qd, *J* = 7.6, 1.2 Hz, 2H), 1.65–1.48 (m, 2H), 1.47–1.44 (m, 1H), 1.42–1.21 (m, 6H), 0.95–0.81 (m, 12H), 0.12 (s, 6H). ^{13}C NMR (101 MHz, $CDCl_3$) δ 140.5, 132.8, 132.3, 110.8,

72.5, 37.9, 32.1, 31.4, 28.9, 25.7, 23.4, 22.5, 18.3, 14.0, -5.2. IR (ATR): ṽ = 3352, 2956, 2928, 2857, 1663, 1463, 1253, 1159, 1058, 1005, 970, 923, 825, 780, 671. HRMS (ESI^+) m/z calcd. for $C_{18}H_{36}O_2SiNa$: 335.2377, found: 335.2374.

***anti*-4-Methyl-1-phenyl-2-((triisopropylsilyl)oxy)pent-4-en-1-ol 9l.** To a solution of Ni(cod)$_2$ (2.6 mg, 0.0094 mmol, 2.5 mol%) and **L6** (3.33 mg, 0.0045 mmol, 1.25 mol%) in toluene (0.5 mL), was added the diene **7** (99.7 mg, 0.42 mmol, 1.1 equiv), benzaldehyde (39.9 mg, 0.38 mmol, 1.0 equiv) and Et_3B solution (0.45 mL, 1 M in hexanes, 0.45 mmol, 1.2 equiv). After work up as described above, the residue was purified by flash chromatography over silica gel (hexane/*tert*-butyl methyl ether = 100/0 to 95/5) to give **9l** as a colorless oil (131 mg, 96%, 99:1 dr). *Rf* = 0.40 (hexane/*tert*-butyl methyl ether = 95/5). ^{1}H NMR (400 MHz, $CDCl_3$) δ 7.39-7.29 (m, 4H), 7.28-.722 (m, 1H), 4.87 (d, *J* = 3.4 Hz, 1H), 4.72-.4.69 (m, 1H), 4.64-4.60 (m, 1H), 4.33 (ddd, *J* = 6.9, 5.7, 3.4 Hz, 1H), 2.74 (br. s, 1H), 2.24 (ddd, *J* = 14.3, 6.9, 1.1 Hz, 1H), 2.00 (ddd, *J* = 14.3, 5.7, 1.1 Hz, 1H), 1.61 (t, *J* = 1.1 Hz, 3H), 1.13-1.09 (m, 21H). ^{13}C NMR (100 MHz, $CDCl_3$) δ 142.3, 140.2, 128.2, 127.4, 126.7, 113.3, 76.8, 75.1, 39.9, 22.9, 18.38, 18.35, 12.9. IR (ATR): ṽ = 3565, 3480, 3082, 2943, 2892, 2866, 1650, 1495, 1462, 1453, 1385, 1324, 1279, 1256, 1246, 1193, 1174, 1088, 1063, 1028, 1013, 999, 939, 919, 882, 798, 756, 744, 699, 674, 653, 622, 560, 541, 402, 466, 441. HRMS (ESI) m/z calcd for $C_{21}H_{36}O_2SiNa$: 371.2378 $[M+Na]^+$, found 371.2376.

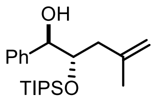

***anti*-2-((*tert*-Butyldimethylsilyl)oxy)-4-methyl-1-phenylpent-4-en-1-ol 9m.** [*Note: not run using the optimised conditions*]. To a solution of Ni(cod)$_2$ (3.0 mg, 0.01 mmol, 10 mol%) and **L6** (4.0 mg, 0.006 mmol, 5.0 mol%) in toluene (0.4 mL), was added the diene **S9** (24.0 mg, 0.12 mmol, 1.1 equiv), benzaldehyde (11.6 mg, 0.1 mmol, 1.0 equiv) and Et_3B solution (0.26 mL, 1 M in hexanes, 0.26 mmol, 2.4 equiv). After work up as described above, the residue was purified by flash chromatography over silica gel (hexane/*tert*-butyl methyl ether = 100/0 to 95/5) to give **9m** as a colorless oil (23 mg, 70%, 97:3 dr). *Rf* = 0.45 (hexane/*tert*-butyl methyl ether = 95/5). ^{1}H NMR (400 MHz, $CDCl_3$) δ 7.38-7.30 (m, 4H), 7.29-7.23 (m, 1H), 4.80-4.73 (m, 2H), 4.70-4.66 (m, 1H), 4.05 (dt, *J* = 8.2, 4.2 Hz, 1H), 2.62 (br. s, 1H), 2.24 (ddd, *J* = 14.0, 8.1, 0.9 Hz, 1H), 1.93 (dd, *J* = 13.9, 4.1 Hz, 1H), 1.65 (br. s, 3H), 0.91-0.89 (m, 9H), 0.06 (s, 3H), 0.02 (s, 3H). ^{13}C NMR (100 MHz, $CDCl_3$) δ 142.5, 140.5, 128.3, 127.5, 126.7, 113.5, 76.9, 75.0, 39.7, 26.0, 23.0, 18.2, -4.4, -4.6. IR (ATR): ṽ = 3458, 3073, 3030, 2952, 2928, 2886, 2856, 1469, 1494, 1471, 1453, 1389, 1361, 1327, 1254, 1193, 1083, 1047, 1026, 1001, 939, 890, 831, 808, 770, 755, 698, 674, 670, 616, 598, 578, 535, 444. HRMS (ESI) m/z calcd for $C_{18}H_{30}O_2SiNa$: 329.1909 $[M+Na]^+$, found 329.1907.

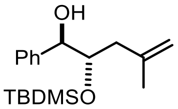

Methyl 4-(*anti*-2-((*tert*-butyldimethylsilyl)oxy)-1-hydroxypent-4-en-1-yl)benzoate 9n.

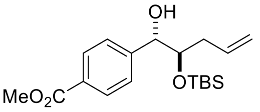

Prepared according to representative procedure D (using 5 mol% **L4** and 5 mol% Ni(cod)$_2$). Purified by flash chromatography over silica gel (hexane/ethyl acetate = 91:9) to give **9n** as a colourless oil (153 mg, 86%, >20:1 dr). ^{1}H (400 MHz, CDCl$_3$) δ = 8.02-7.98 (m, 2H), 7.44-7.40 (m, 2H), 5.73 (dddd, *J* = 17.0, 10.5, 7.5, 7.0 Hz, 1H), 5.02-4.93 (m, 2H), 4.77 (d, *J* = 4.5 Hz, 1H), 3.91 (s, 3H), 2.27 (app. dtt, *J* = 14.5, 7.0, 1.0 Hz, 1H), 1.96 (app. dddt, *J* = 14.5, 7.5, 4.5, 1.0 Hz, 1H), 0.90-0.87 (m, 9H), 0.06 (s, 3H), –0.04 (s, 3H); ^{13}C (101 MHz, CDCl$_3$) δ = 167.1, 145.9, 135.0, 129.6, 129.4, 126.7, 117.5, 76.3, 75.9, 52.2, 36.0, 26.0, 18.2, –4.3, –4.7; IR (ATR): ṽ = 3491, 3076, 2953, 2929, 2887, 2857, 1724, 1612, 1462, 1436, 1276, 1252, 1099, 912, 831, 772, 710; LRMS m/z (ESI$^+$) [M+Na]$^+$ 373; HRMS (ESI$^+$, m/z) calculated for [$C_{19}H_{30}O_4SiNa$]$^+$ 373.1810, found 373.1806.

4-(*anti*-2-((*tert*-Butyldimethylsilyl)oxy)-1-hydroxypent-4-en-1-yl)benzonitrile 9o.

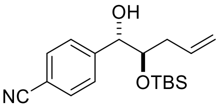

Prepared according to representative procedure D (using 5 mol% **L4** and 5 mol% Ni(cod)$_2$). Purified by flash chromatography over silica gel (hexane/ethyl acetate = 9:1) to give **9o** as a colourless oil (141 mg, 87%, >20:1 dr). ^{1}H (400 MHz, CDCl$_3$) δ = 7.64-7.61 (m, 2H), 7.48-7.45 (m, 2H), 5.78-5.67 (m, 1H), 5.04-4.95 (m, 2H), 4.75 (d, *J* = 4.5 Hz, 1H), 3.88 (dt, *J* = 7.0, 4.5 Hz, 1H), 2.49 (br. s, 1H, -OH), 2.34-2.22 (m, 1H), 1.96 (dddt, *J* = 11.5, 8.0, 4.5, 1.5 Hz, 1H), 0.89-0.87 (m, 9H), 0.05 (s, 3H), –0.06 (s, 3H); ^{13}C (101 MHz, CDCl$_3$) δ = 146.2, 134.6, 132.1, 127.6, 119.0, 117.7, 111.4, 76.0, 75.7, 36.2, 25.9, 18.2, –4.3, –4.8; IR (ATR): ṽ = 3489, 3076, 2954, 2929, 2866, 2857, 2229, 1610, 1471, 1411, 1252, 1091, 914, 827, 774, 567; LRMS m/z (ESI$^+$) [M+Na]$^+$ 340; HRMS (ESI$^+$, m/z) calculated for [$C_{18}H_{27}O_2NSiNa$]$^+$ 340.1707, found 340.1703.

1-(3-(*anti*-2-((*tert*-Butyldimethylsilyl)oxy)-1-hydroxypent-4-en-1-yl)phenyl)ethan-1-one

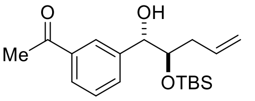

9p. Prepared according to representative procedure D (using 5 mol% **L4** and 5 mol% Ni(cod)$_2$). Purified by flash chromatography over silica gel (hexane/ethyl acetate = 9:1) to give **9p** as a colourless oil (146 mg, 86%, >20:1 dr). ^{1}H (400 MHz, CDCl$_3$) δ = 7.94-7.92 (m, 1H), 7.86 (dt, *J* = 8.0, 1.5 Hz, 1H), 7.59-7.55 (m, 1H), 7.43 (t, *J* = 8.0 Hz, 1H), 5.75 (dddd, *J* = 17.0, 10.5, 7.5, 7.0 Hz, 1H), 5.04-4.95 (m, 2H), 4.76 (d, *J* = 4.5 Hz, 1H), 3.91 (dt, *J* = 7.0, 4.5 Hz, 1H), 2.60 (s, 3H), 2.33-2.24 (m, 1H), 2.00 (dddt, *J* = 14.5, 7.0, 4.5, 1.5 Hz, 1H), 0.89-0.86 (m, 9H), 0.05 (s, 3H), -0.06 (s, 3H); ^{13}C (101 MHz, CDCl$_3$) δ = 198.3, 141.5, 137.1, 134.9, 131.6, 128.6, 127.7, 126.7, 117.5, 76.2, 75.9, 36.3, 26.8, 25.9, 18.2, –4.3, –4.7; IR (ATR): ṽ = 3429, 3075, 2953, 2929, 2887, 2856, 1681, 1602, 1586, 1471, 1434, 1359, 1255, 911, 830, 774, 697, 589; LRMS m/z (ESI$^+$) [M+Na]$^+$ 357; HRMS (ESI$^-$, m/z) calculated for [$C_{19}H_{29}O_3Si$]$^-$ 333.1892, found 333.1891.

***anti*-1-(Furan-2-yl)-4-methyl-2-((triisopropylsilyl)oxy)pent-4-en-1-ol 9q.** Prepared

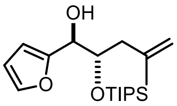

according to representative procedure D. Purified by flash chromatography over silica gel (hexane/*tert*-butyl methyl ether = 100/0 to 95/5) to give **9q** as a light yellow liquid (113 mg, 88.5%, 99:1 dr). *Rf* = 0.35 (hexane/*tert*-butyl methyl ether = 95/5). ^{1}H NMR (400 MHz, $CDCl_3$) δ 7.36-7.35 (m, 1H), 6.35-6.32 (m, 2H), 4.77-4.74 (m, 2H), 4.64 (dq, *J* = 2.1, 1.0 Hz, 1H), 4.41 (ddd, *J* = 8.1, 5.5, 3.3 Hz, 1H), 2.61 (br. s, 1H), 2.33 (br dd, *J* = 14.1, 5.5 Hz, 1H), 2.22 (ddd, *J* = 14.1, 8.1, 1.0 Hz, 1H), 1.71 (t, *J* = 1.1 Hz, 3H), 1.11-1.08 (m, 21H). ^{13}C NMR (100 MHz, $CDCl_3$) δ 153.8, 141.7, 141.6, 113.5, 110.3, 107.4, 73.6, 71.2, 41.7, 23.0, 18.3, 18.3, 12.9. IR (ATR): ṽ = 3569, 3481, 2943, 2893, 2866, 1649, 1504, 1463, 1382, 1370, 1334, 1317, 1245, 1208, 1167, 1145, 1107, 1066, 1035, 1000, 950, 932, 882, 804, 731, 676, 652, 598, 530, 503, 443. HRMS (ESI) m/z calcd for $C_{19}H_{34}O_3SiNa$: 361.2175 $[M+Na]^+$, found 361.2169.

***anti*-4-Methyl-1-(thiophen-2-yl)-2-((triisopropylsilyl)oxy)pent-4-en-1-ol 9r.** Prepared

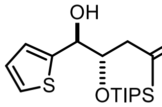

according to representative procedure D. Purified by flash chromatography over silica gel (hexane/*tert*-butyl methyl ether = 100/0 to 95/5) to give **9r** as a light yellow oil (32 mg, 24%, 99:1 dr). *Rf* = 0.50 (hexane/*tert*-butyl methyl ether = 90/10). ^{1}H NMR (400 MHz, $CDCl_3$) δ 7.28 (br dd, *J* = 5.1, 1.2 Hz, 1H), 7.01 (ddd, *J* = 3.4, 1.2, 0.6 Hz, 1H), 6.98 (dd, *J* = 5.1, 3.4 Hz, 1H), 4.96 (d, *J* = 2.9 Hz, 1H), 4.77 (br. s, 1H), 4.63-4.59 (m, 1H), 4.42 (ddd, *J* = 9.0, 5.2, 2.9 Hz, 1H), 2.62 (br. s, 1H), 2.31 (br dd, *J* = 14.0, 5.3 Hz, 1H), 2.10 (ddd, *J* = 14.0, 8.9, 0.9 Hz, 1H), 1.71 (br. s, 3H), 1.16-1.11 (m, 21H). ^{13}C NMR (100 MHz, $CDCl_3$) δ 142.4, 141.5, 126.4, 126.1, 125.9, 113.7, 74.3, 72.7, 41.8, 22.9, 18.39, 18.37, 12.9. IR (ATR): ṽ = 3569, 3444, 3075, 2942, 2893, 2866, 1650, 1462, 1382, 1329, 1244, 1201, 1164, 1102, 1066, 1043, 1011, 998, 967, 935, 918, 882, 857, 827, 799, 670, 676, 689, 566, 538, 505, 457, 443. HRMS (ESI) m/z calcd for $C_{19}H_{34}O_2SSiNa$: 377.1945 $[M+Na]^+$, found 377.1941.

***syn*-4-((*tert*-Butyldimethylsilyl)oxy)-1-phenylhept-6-en-3-ol 11a**. To a blood red solution of

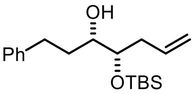

Ni(COD)$_2$ (8.5 mg, 0.031 mmol) and **L4** (21.9 mg, 0.031 mmol) in toluene (2.5 mL) was added the diene **S17** (251 mg, 1.36 mmol). Triethylborane (1.9 mL, 1 M in hexanes, 1.9 mmol) was added followed by hydrocinnamaldehyde (0.165 mL, 1.24 mmol). The red reaction mixture was stirred at rt for 16 hours. The reaction was quenched with the addition of $NaHCO_3$ solution and stirred for 30 minutes before organic phase was separated, and the aqueous phase was extracted with *tert*-butyl methyl ether (3 x 10 mL). The combined organic phases were washed with brine solution and dried over Na_2SO_4, filtered and concentrated to give a cloudy yellow oil. The residue was purified by flash chromatography (hexane/ethyl acetate 95:5 to 9:1) to give product **11a** as a colourless oil (318 mg, 80%). ^{1}H NMR (400 MHz, $CDCl_3$) δ 7.33–7.25 (m, 2H), 7.20 (dt, *J* = 8.1, 2.1 Hz, 3H), 5.77 (ddt, *J* = 17.3, 10.2, 7.2 Hz, 1H), 5.11–4.99 (m, 2H), 3.59 (ddd, *J* = 7.0, 4.7, 3.8 Hz, 1H), 3.49 (tdd, *J* = 8.4, 5.8, 4.2 Hz, 1H), 2.85 (ddd, *J* = 13.8, 8.8, 6.5 Hz, 1H), 2.74–2.60 (m, 1H), 2.48–2.37 (m, 1H),

2.27–2.13 (m, 2H), 1.81–1.70 (m, 2H), 0.91 (s, 8H), 0.10 (s, 3H), 0.08 (s, 2H). ^{13}C NMR (101 MHz, $CDCl_3$) *δ* 142.2, 134.2, 128.4, 128.3, 125.7, 117.6, 74.7, 71.9, 38.6, 35.7, 32.2, 25.9, 18.1, –4.1, – 4.6 ppm. IR (ATR): ṽ = 3461, 2929, 2857, 1641, 1604, 1496, 1471, 1390, 1361, 1254, 1074, 1004, 912, 833, 810, 774, 746, 698, 678. HRMS (ESI$^+$) m/z calcd. for $C_{19}H_{32}O_2SiNa$: 343.2063, found: 343.2065.

***syn*-2-((*tert*-Butyldimethylsilyl)oxy)-1-phenylpent-4-en-1-ol 11b**. To a blood red solution of

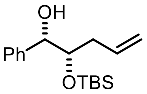

Ni(COD)$_2$ (6.9 mg, 0.025 mmol) and **L4** (18 mg, 0.025 mmol) in toluene (2 mL) was added the diene **S17** (185 mg, 1 mmol). Triethylborane (1.5 mL, 1 M in hexanes, 1.5 mmol) was added followed by benzaldehyde (0.1 mL, 1 mmol). The red reaction mixture was stirred at rt for 16 hours. The reaction was quenched with the addition of $NaHCO_3$ solution and stirred for 30 minutes before the organic phase was separated, and the aqueous phase was extracted with *tert*-butyl methyl ether (3 x 10 mL). The combined organic phases were washed with brine solution and dried over Na_2SO_4, filtered and concentrated to give a cloudy yellow oil. The residue was purified by flash chromatography (hexane/ethyl acetate 95:5 to 9:1) to give the product **11b** as a mixture of diastereomers (8:1 *syn/anti*) as a colourless oil (157 mg, 54%). ^{1}H NMR (400 MHz, $CDCl_3$) *δ* 7.37–7.24 (m, 5H), 5.86 (dddd, *J* = 17.1, 10.5, 7.5, 6.8 Hz, 1H), 5.15–5.05 (m, 2H), 4.60 (d, *J* = 4.7 Hz, 1H), 3.80 (dt, *J* = 7.0, 4.5 Hz, 1H), 2.42 (dtt, *J* = 14.0, 6.9, 1.4 Hz, 1H), 2.16 (dddt, *J* = 14.3, 7.5, 4.4, 1.2 Hz, 1H); 0.87 (s, 9H), –0.01 (s, 3H), –0.20 (s, 3H). ^{13}C NMR (101 MHz, $CDCl_3$) δ 142.0, 134.0, 128.1, 127.4, 126.5, 117.9, 76.8, 74.7, 38.4, 25.8, –4.4, –5.2 ppm. IR (ATR): ṽ = 3460, 2953, 2929, 2886, 2857, 1641, 1494, 1472, 1389, 1361, 1253, 1195, 1076, 1058, 1026, 1002, 913, 851, 807, 774, 746, 610, 568. HRMS (ESI$^+$) m/z calcd. for $C_{17}H_{28}O_2SiNa$: 315.1751, found: 315.1753.

***anti*-5-Methyl-1-phenyl-4-((triisopropylsilyl)oxy)hept-6-en-3-ol 13.** Prepared according to

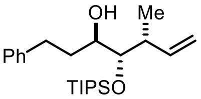

representative procedure D. Purified by flash chromatography over silica gel (hexane/*tert*-butyl methyl ether = 100/0 to 95/5) to give **13** as colorless oil (129 mg, 91%, 94:6 dr). *Rf* = 0.50 (hexane/*tert*-butyl methyl ether = 90/10). ^{1}H NMR (400 MHz, $CDCl_3$) δ 7.31-7.26 (m, 2H), 7.22-7.15 (m, 3H), 5.93 (ddd, *J* = 17.6, 10.3, 7.5 Hz, 1H), 5.01-4.93 (m, 2H), 3.76 (dd, *J* = 4.4, 3.5 Hz, 1H), 3.70 (dt, *J* = 9.4, 3.4 Hz, 1H), 2.91 (ddd, *J* = 14.2, 9.4, 5.2 Hz, 1H), 2.68 (ddd, *J* = 13.7, 9.2, 7.4 Hz, 1H), 2.49-2.40 (m, 1H), 2.07 (br. s, 1H), 1.89-1.72 (m, 2H), 1.10 (d, *J* = 6.9 Hz, 3H), 1.08-1.06 (m, 21H). ^{13}C NMR (100 MHz, $CDCl_3$) δ 142.3, 142.2, 128.6, 128.5, 125.9, 113.9, 79.0, 74.2, 40.6, 33.9, 32.7, 18.4, 15.8, 13.1. IR (ATR): ṽ = 3585, 3480, 3082, 3064, 3027, 2943, 2891, 2866, 1639, 1604, 1496, 1456, 1384, 1367, 1341, 1284, 1247, 1213, 1096, 1063, 1032, 1014, 997, 913, 882, 819, 791, 746, 698, 676, 660, 568, 504, 464, 446.HRMS (ESI) m/z calcd for $C_{23}H_{40}O_2SiNa$: 399.2689 $[M+Na]^+$, found 399.2689.

The stereochemistry of the above example **13** was further verified by synthesis of the known compound[12] **13b** below bearing the same groups with 1,2-*anti* and 1,3-*anti* relationships:

2,3-*anti*-3,4-*syn*-3-((Triisopropylsilyl)oxy)-4-methylhex-5-en-2-ol 13b. To a blood red

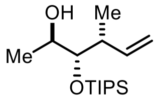

solution of Ni(COD)$_2$ (7.7 mg, 0.028 mmol) and **L4** (20 mg, 0.028 mmol) in toluene (2.22 mL) was added the dienolsilane 124 (270 mg, 1.12 mmol) causing a darkening of the solution to a brown/red colour. Triethylborane (1.7 mL, 1 M in hexanes, 1.7 mmol) was added followed by acetaldehyde (63 µL, 1.12 mmol). The red reaction mixture was stirred at RT for 16 hours. The reaction was quenched with the addition of $NaHCO_3$ solution and stirred for 30 minutes before organic phase was separated, and the aqueous phase was extracted with *tert*-butyl methyl ether (3 x 10 mL). The combined organic phases were washed with brine solution and dried over Na_2SO_4, filtered and concentrated to give a cloudy yellow oil. The residue was purified by flash chromatography (hexane/Ethyl acetate 95:5 to 9:1) to give product **13b** as a colourless oil. ^{1}H NMR (400 MHz, $CDCl_3$) δ 5.85 (ddd, *J* = 17.5, 10.4, 7.3 Hz, 1H), 4.99–4.88 (m, 2H), 3.80 (qdd, *J* = 6.4, 4.8, 3.4 Hz, 1H), 3.69 (dd, *J* = 4.9, 3.4 Hz, 1H), 2.44 – 2.31 (m, 1H), 1.88 (d, *J* = 4.7 Hz, 1H), 1.11 (d, *J* = 6.5 Hz, 3H), 1.05–1.02 (m, 24H). ^{13}C NMR (101 MHz, $CDCl_3$) *δ* 141.8, 113.7, 79.5, 70.4, 40.7, 18.3, 17.8, 15.8, 13.1 ppm. IR (ATR): $\tilde{\nu}$ = 3443, 2943, 2867, 1639, 1463, 1385, 1253, 1102, 1061, 1015, 997, 912, 851, 826, 790, 774, 464. HRMS (ESI$^+$) m/z calcd. for $C_{16}H_{34}O_2SiNa$: 309.2220, found: 309.2221.

Procedure for the Synthesis of Racemic Diol Derivatives with PPh_3

The following procedure was used to obtain racemates for chiral HPLC analysis. Products were generally obtained with lower diastereoselectivity than the enantioselective reaction with **L17**. The regioselectivity was also generally lower, with small amounts of the product of reaction at C4 (rather than C1) of the diene observed – however, this side-product is generally more polar and consequently easily separable by flash chromatography.

Ni(cod)$_2$ (4 mg, 0.00145 mmol) and PPh_3 (3.8 mg, 0.00145 mmol) were added to a flame-dried Schlenk flask under argon and dissolved in toluene (0.6 mL). The diene (0.319 mmol), triethylborane (0.44 mL, 1 M in hexanes, 0.44 mmol) and aldehyde (0.29 mmol) were added and the reaction was stirred at rt overnight. The reaction mixture was cooled to 0 °C and pH 7 phosphate buffer solution (~1.5 mL) and aq. H_2O_2 (30% in water, ~0.5 mL) were added. After stirring for 1 hour, the mixture was diluted with ethyl acetate (10 mL) and water (10 mL) and transferred to a separating funnel. The organic layer was separated and the aqueous layer was extracted with 2 × 5 mL ethyl acetate. The combined organic layers were washed with sat. aq. sodium thiosulfate solution (10 mL) and brine (10 mL), dried over anhydrous Na_2SO_4, filtered and concentrated *in vacuo*. Purification by flash chromatography (SiO_2, hexane/ethyl acetate) gave the desired diol.

1.3.2.2 Enantioselective Scope

Representative Procedure E for Enantioselective Vicinal Diol Synthesis with L17

***anti*-4-((*tert*-Butyldimethylsilyl)oxy)-1-phenylhept-6-en-3-ol 18a.** $Ni(cod)_2$ (8 mg, 0.029 mmol) and VAPhos-NEt_2 **L17** (18.6 mg, 0.029 mmol) were added to a flame-dried Schlenk flask under argon and dissolved in toluene (0.6 mL). Diene **S13** (110 mg, 0.582 mmol) and triethylborane (0.44 mL, 1M in hexanes, 0.44 mmol) were added and the resulting solution was cooled to –20 °C. Hydrocinnamaldehyde (38 µL, 0.291 mmol) was then added, the reaction was sealed under argon and the reaction was stirred for 64 h. The reaction mixture was warmed to 0 °C and pH 7 phosphate buffer solution (1 mL) and aq. H_2O_2 (30% in water, ~0.5 mL) were added. After stirring for 1 hour at 0 °C, the mixture was diluted with ethyl acetate (10 mL) and water (10 mL) and transferred to a separating funnel. The organic layer was separated and the aqueous layer was extracted with 2 × 5 mL ethyl acetate. The combined organic layers were washed with sat. aq. sodium thiosulfate solution (10 mL) and brine (10 mL), dried over anhydrous Na_2SO_4, filtered and concentrated *in vacuo* to give a light yellow oil. Purification by flash chromatography (SiO_2, hexane/ethyl acetate 97:3) gave vicinal diol **18a** as a colourless oil (72 mg, 77% yield, 20:1 dr, 84% ee).

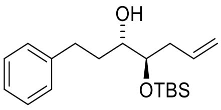

In our hands, this reaction is robust and reproducible. However, several of the components are oxygen-sensitive and care must be taken to ensure the reaction is air-free and that all reaction components are of high purity. The following notes are provided to prevent potential issues and help prospective users obtain the highest yields.

Notes on solvents and reagents:

1. Toluene used in the reaction was refluxed overnight over CaH_2 under argon and then distilled under argon, and was therefore rigorously oxygen-free.
2. $Ni(cod)_2$ was purchased from Strem Chemicals and transferred to a flame-dried Schlenk flask under argon immediately after opening, after which it is stored in the freezer at –20 °C under an over-pressure of argon. The colour should be bright yellow. When taken out of the freezer, the Schlenk flask was allowed to warm to room temperature before weighing the solid out directly into the reaction flask using the "Argon pants" apparatus shown in Figure S1 below. The same 2 gram batch of $Ni(cod)_2$ was taken out of the freezer and used in this manner multiple times a week for over a year with no issues.
3. Liquid aldehydes were typically distilled under vacuum and stored in the freezer (–20 °C) under argon in a crimp capped vial prior to use. Most aldehydes could be stored and re-used for weeks or months in this manner. The presence of carboxylic acid impurities may have a deleterious effect on the enantioselectivity of the reaction and

so the purity of aldehydes was checked by ^{1}H NMR spectroscopy after distillation, as well as before use in the reaction if stored for a long time.

4. Solid aldehydes such as 4-phenylbenzaldehyde and 4-formylphenylboronic acid, pinacol ester were typically used as received after checking purity by ^{1}H NMR spectroscopy. These compounds were stored on the bench at room temperature as they were found not to undergo appreciable air oxidation over days or weeks.
5. Silyloxydienes used were all liquids and were stored analogously to liquid aldehydes. These compounds are very stable at –20 °C: no evidence of decomposition or isomerisation was noted even after several months.
6. Triethylborane (1 M in hexane) was stored in the freezer at -20 °C under argon. Although triethylborane itself is very pyrophoric and any solution of it should be handled under oxygen-free conditions, we had no issues using the hexanes solution. Triethylborane in THF solution is also effective, though since the use of THF as reaction solvent gives marginally lower ees (see section 1.2), the enantioselectivity may drop slightly.

Notes on reaction set-up and purification:

7. Reactions with aliphatic aldehydes are typically run at -20 °C, while aryl aldehydes are run at -40 °C. Electron-rich aryl aldehydes (e.g. *p*-anisaldehyde, see **18f**) may be run at higher temperatures for better conversion.
8. The ligand should always be added before the solvent as it coordinates and helps to stabilise the nickel(0) catalyst if any oxygen is present in the solution. This is important as $Ni(cod)_2$ is relatively stable in the solid state but extremely O_2-sensitive in solution. The diene also seems to help stabilise the nickel, while triethylborane will react with any excess oxygen. The addition of these two components soon after the solvent is therefore recommended to minimise potential decomposition, though it is not necessary if oxygen is not present.
9. Non-polar aldehydes such as hydrocinnamaldehyde sometimes co-eluted with the products during column chromatography. Any remaining traces were removed by standing the product under high vacuum ($1*10^{-3}$ mbar) overnight.
10. The reaction is run in a sealed Schlenk flask rather than under a flow of argon due to the volatility of triethylborane (bp 95 °C). However, ethylene gas is given off (from reduction of the nickel catalyst with triethylborane) over time, and care should therefore be taken to use reaction vessels which can withstand the increase in pressure caused by this gas evolution, especially if scaling up.
11. The diethylborinate ester formed in the reaction can be surprisingly resistant to hydrolysis (to give the alcohol product). In the original (virtually racemic) reaction with cyclodiphosphazane **L6**, the reaction was quenched with sat. aq. $NaHCO_3$ solution.

However, traces of remaining (non-polar) borinate ester were often observed to elute at the start of the column. Complete hydrolysis could be achieved by adsorbing the crude reaction mixture onto silica gel and leaving it to stand overnight prior to purification. Nevertheless, when performing the enantioselective scope it was decided to switch to a neutral oxidative work-up with H_2O_2 and a pH 7 phosphate buffer, which quantitatively cleaves the B-O bond. Either procedure may be used, depending on the presence of base or oxidatively sensitive functionalities on the product.

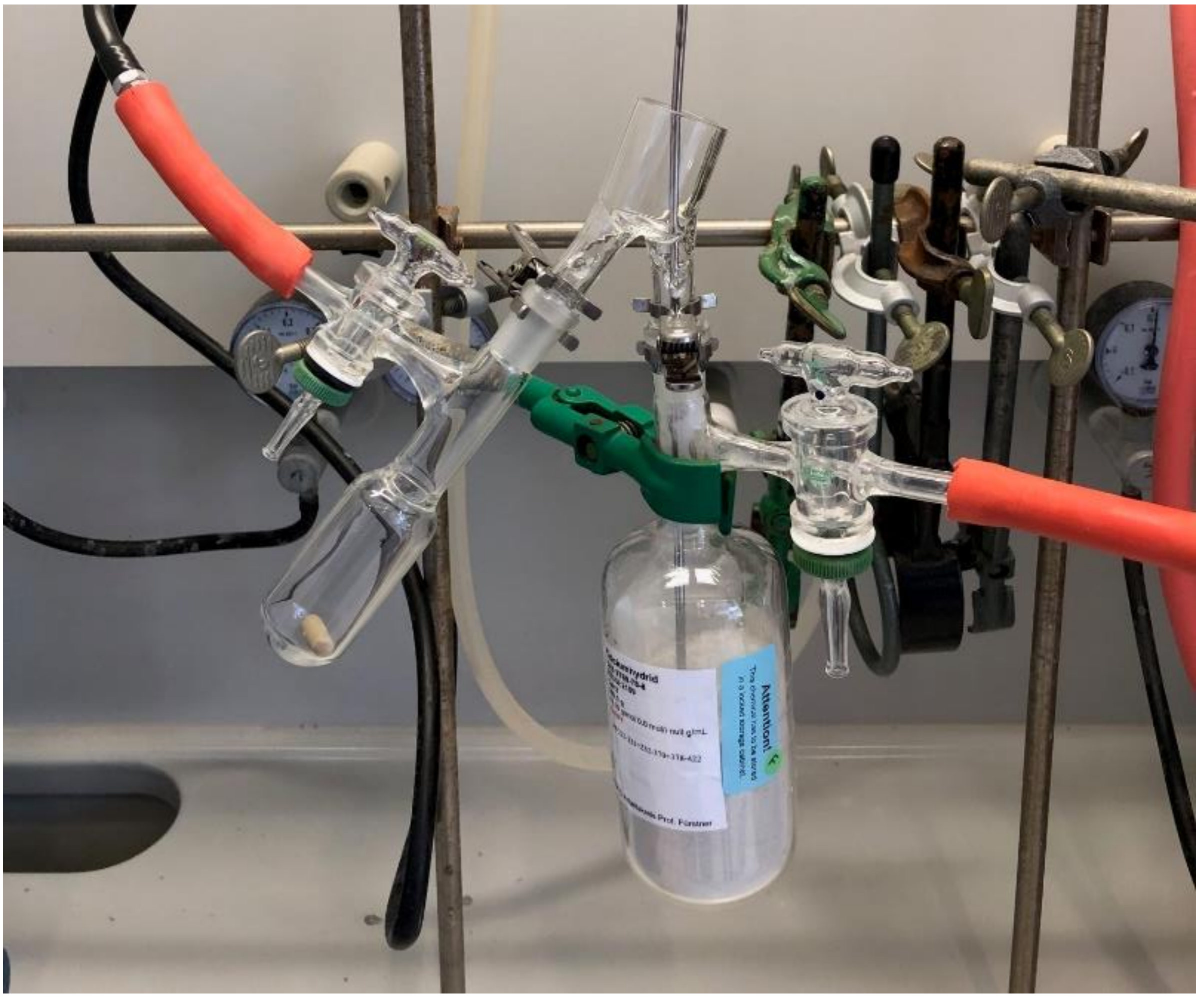

Figure S1. Use of “Argon pants”. The reaction flask and reagent flask are connected *via* a glassware with two legs and an open top. Argon is run through both Schlenk flasks continuously during operation, ensuring an inert atmosphere and allowing weighing of air-sensitive compounds like Ni(cod)$_2$ without use of a glovebox. Note: compound shown in picture is not Ni(cod)$_2$.

Notes on the dienes:

TBS dienes seem to be the most reactive with all aldehydes and give generally good enantioselectivity.

TIPS dienes often give the best enantioselectivity but slightly lower reactivity than TBS with alkyl aldehydes. The (*Z*)-TIPS diene tends to give lower diastereoselectivity for *syn* products than the less bulky silyl groups.

TES dienes do not have a particular advantage over TBS and TIPS in terms of reactivity or enantioselectivity, but the (Z)-TES diene may give better diastereoselectivity for *syn* products. These products are unsurprisingly more sensitive than those derived from the bulkier silyl groups and their use was generally avoided in favour of TBS and TIPS dienes.

Notes on HPLC analysis:

Although diastereoselectivity was typically very high for the enantioselective reaction with **L17**, the racemic reaction with PPh_3 generally gave a lower dr. This often meant the individual product diastereomers and their enantiomers were difficult to separate on chiral HPLC columns. For this reason, 2D HPLC analysis was often employed to ensure accurate ee determination. This involved first running the isolated product down an achiral column to separate the diastereomers, before subsequent chiral separation of the pure major diastereomer using a chiral column.

Assignment of Absolute Configuration:

The absolute and relative configuration of the *anti*-configured product **18l** was determined by a three-step sequence of ozonolysis, chromium oxidation and desilylation with TBAF to the corresponding lactone. The ^{1}H NMR spectrum matched the literature. The sign of the optical rotation was opposite to that of (4*S*,5*R*)-5-(4-fluorophenyl)-4-hydroxydihydrofuran-2(3*H*)-one reported in the literature.[13] Ligand **L17** comprising (*R*)-VAPOL therefore gives the **(4*R*,5*S*)-configured product**.

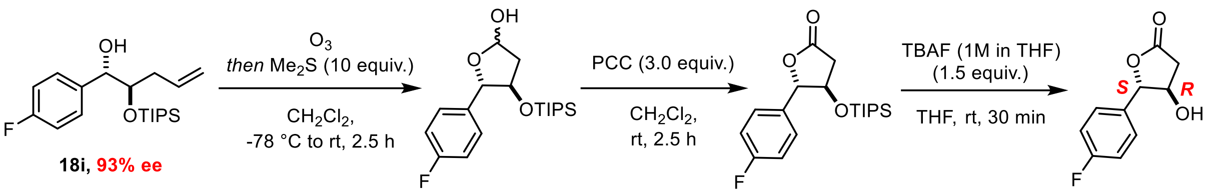

Data of the lactone: $[\alpha]_D^{20}$ = –20.0 (c = 0.7, MeOH); lit. (for (4*S*,5*R*)-5-(4-fluorophenyl)-4-hydroxydihydrofuran-2(3*H*)-one): $[\alpha]_D^{20}$ = +25 (c = 0.87, MeOH), 95% ee);[13] ^{1}H (400 MHz, $CDCl_3$) δ = 7.35-7.30 (m, 2H), 7.13-7.07 (m, 2H), 5.32 (d, *J* = 4.0 Hz, 1H), 4.46 (ddd, *J* = 6.5, 5.5, 4.0 Hz, 1H), 2.90 (dd, *J* = 17.5, 6.5 Hz, 1H), 2.64 (dd, *J* = 17.5, 5.5 Hz, 1H), 2.29 (br. s, 1H).

The absolute and relative configuration of the *syn*-configured product **19d** was determined by desilylation with TBAF to give the corresponding *syn*-diol **19d-OH**. The ^{1}H NMR spectrum matched the literature. The sign of the optical rotation was opposite to that of the (*R*,*R*)-configured product reported previously in the literature.[14] Ligand **L17** comprising (*R*)-VAPOL therefore gives the **(*S*,*S*)-configured product**.

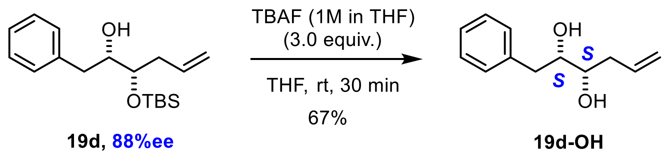

Data of diol **19d**-OH: $[\alpha]_D^{22}$ = –2.0 (c = 1.15, $CHCl_3$); lit. (for the *R*,*R*-isomer): $[\alpha]_D^{20}$ = +6.06 (c = 1.23, $CHCl_3$), 96% ee);[14] ^{1}H (400 MHz, $CDCl_3$) δ = 7.37-7.29 (m, 2H), 7.26-7.21 (m, 3H), 5.86 (dddd, *J* = 17.0, 10.0, 8.0, 6.5 Hz, 1H), 5.20-5.12 (m, 2H), 3.73 (dd, *J* = 8.5, 4.5 Hz, 1H), 3.58 (dd, *J* = 8.0, 4.0 Hz, 1H), 2.91 (dd, *J* = 13.5, 5.0 Hz, 1H), 2.79 (dd, *J* = 13.5, 8.5 Hz, 1H), 2.44-2.28 (m, 2H), 2.13 (br. d, *J* = 4.5 Hz, 1H), 2.00 (br. d, *J* = 4.5 Hz, 1H).

It is important to note that the chiral center derived from the aldehyde is *S*-configured in the *anti*- as well as in the *syn*-series. On this basis, all other products were assigned by analogy.

***anti*-5-Methyl-1-phenyl-4-((triisopropylsilyl)oxy)hept-6-en-3-ol 13.** Prepared according to representative procedure E (–20 °C). Purified by column chromatography over silica gel (hexane/ethyl acetate = 99:1 to 98:2) to give **13** as a colorless oil (67 mg, yield = 61%, >10:1 dr). For characterisation data, please see the entry for the racemate. The shown absolute configuration was assigned by analogy (see above).

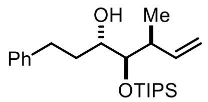

The ee of **13** was determined by 2D HPLC analysis.

Separation of diastereomers: 100 m RX-SiL, ∅ 4.6 mm, *n*-heptane/MTBE 98:2, v = 1.0 mL/min, λ = 220 nm, t(major) = 3.60 min, 308 K. (Note: minor diastereomer not identified for either sample)

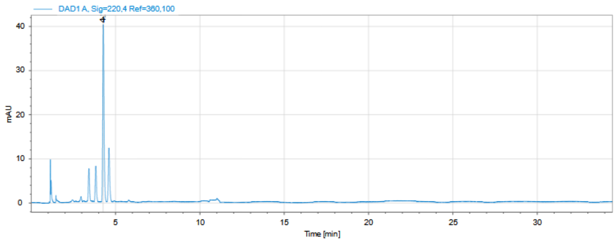

Sampling table (¹D)

Cut group	Cut #	¹D Cut start [min]	¹D Ret. time [min]	¹D Duration [min]	Trigger	²D Run start [min]
	1	4.24	4.275	0.04	Peak	4.30

13: separation of diastereomers on achiral column

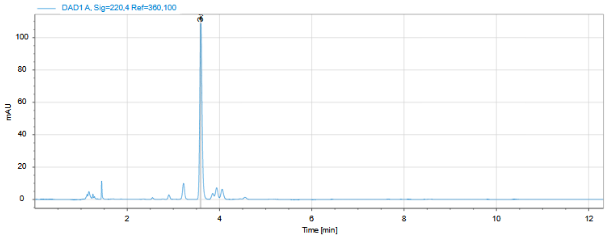

Sampling table (¹D)

Cut group	Cut #	¹D Cut start [min]	¹D Ret. time [min]	¹D Duration [min]	Trigger	²D Run start [min]
	1	3.57	3.595	0.04	Peak	3.62

rac-**13:** separation of diastereomers on achiral column

Chiral separation of major diastereomer: 150 mm Chiralcel OD-3R, 4.6 mm i.D., *n*-heptane/MTBE = 85:15, v = 1.0 mL/min, λ = 220 nm, t(major) = 3.00 min, t(minor) = 4.53 min. 298 K

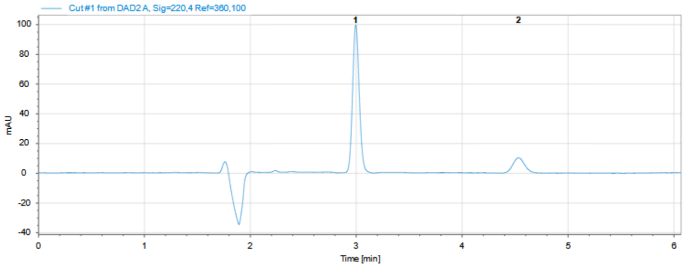

Signal: DAD2 A, Sig=220,4 Ref=360,100

Compound	Cut	Ret.Time	Area	Width	Height	Symmetry
1	1	2.995	437.824<br>**437.824**	0.069	99.508	0.871
2	1	4.532	79.729<br>**79.729**	0.109	10.297	0.910

Component	¹D Sampling range [min]	Ret.Time ²D [min]	Area	Area%	
1	4.24 - 4.28	2.995	437.824	84.595	1. enantiomer
2	4.24 - 4.28	4.532	79.729	15.405	2. enantiomer

ee = 69.2%

13: ee determination of pure major diastereomer

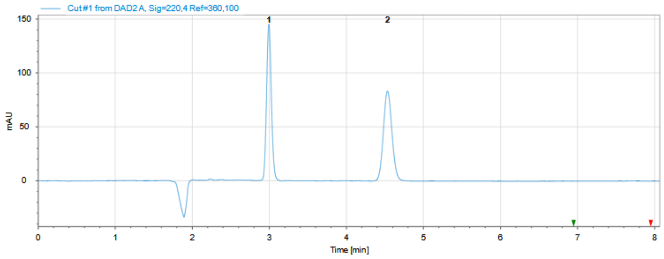

Signal: DAD2 A, Sig=220,4 Ref=360,100

Compound	Cut	Ret.Time	Area	Width	Height	Symmetry
1	1	2.993	637.012<br>**637.012**	0.073	145.098	0.864
2	1	4.533	647.818<br>**647.818**	0.129	83.438	0.923

rac-**13**: separation of enantiomers

***anti*-4-((*tert*-Butyldimethylsilyl)oxy)-1-phenylhept-6-en-3-ol 18a.** $[\alpha]_D^{20}$ = –11.2 (c = 0.51, CHCl3); ^{1}H NMR (400 MHz, $CDCl_3$) δ 7.33–7.25 (m, 2H), 7.25–7.15 (m, 3H), 5.79 (ddt, *J* = 17.3, 10.2, 7.2 Hz, 1H), 5.09 – 4.98 (m, 2H), 3.71 – 3.64 (m, 1H), 3.61 (dq, *J* = 8.1, 4.0 Hz, 1H), 2.89 (ddd, *J* = 14.6, 8.8, 6.5 Hz, 1H), 2.71–2.60 (m, 1H), 2.37–2.27 (m, 1H), 2.25–2.15 (m, 1H), 2.13 (dd, *J* = 4.1, 1.3 Hz, 1H), 1.75 (dddd, *J* = 9.3, 8.0, 5.2, 2.0 Hz, 2H), 0.90 (d, *J* = 0.7 Hz, 9H), 0.06 (s, 3H), 0.05 (s, 3H). ^{13}C NMR (101 MHz, $CDCl_3$) δ 142.1, 135.3, 128.4, 128.4, 125.8, 117.0, 75.1, 73.8, 36.2, 33.5, 32.4, 25.9, 18.1, –4.3, –4.6. IR (ATR): ṽ = 3460, 2956, 2926, 2857, 1472, 1361, 1253, 1074, 1031, 1004, 912, 825, 810, 780, 746, 698. HRMS (GC-CI): m/z calcd. for $C_{19}H_{33}O_2Si$: 321.2244, found: 321.2239. The shown absolute configuration was assigned by analogy (see above).

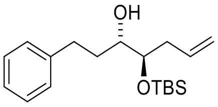

The ee of **18a** was determined by HPLC analysis: Daicel 150 mm Chiralpak IB-N-3, ∅ 4.6 mm, n-heptane/i-propanol = 99:1, v = 1.0 mL/min, λ = 210 nm, t(major) = 3.68 min, t(minor) = 7.61 min.

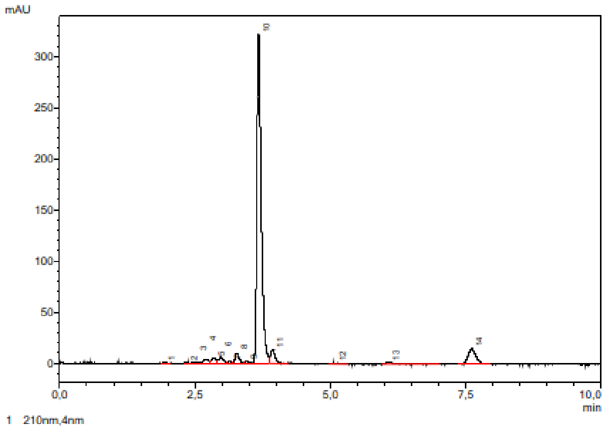

1 210nm,4nm

PDA Ch1 210nm

Peak #	Ret. Time	Area %	Name
1	1,93	0,29	
2	2,34	0,16	
3	2,51	0,26	
4	2,69	1,22	
5	2,85	1,57	
6	2,97	1,79	
7	3,14	0,62	
8	3,27	2,46	
9	3,45	0,72	
10	3,68	79,28	1. Enantiomer Diastereomer 1 84.2 % ee
11	3,93	3,78	1. u. 2. Enantiomer Diastereomer 2
12	5,09	0,33	
13	6,07	0,72	
14	7,61	6,79	2. Enantiomer Diastereomer 1
Total		100,00	

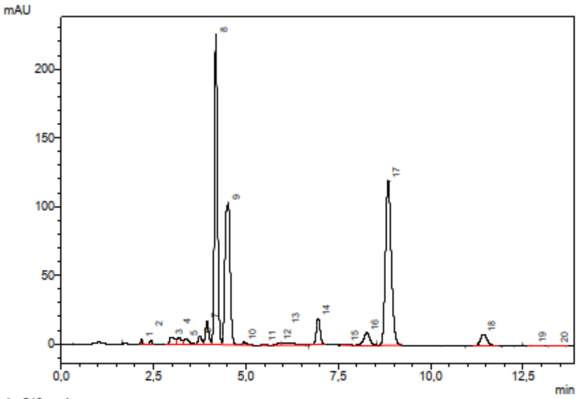

1 210nm,4nm

PDA Ch1 210nm

Peak #	Ret. Time	Area %	Name
1	2,17	0,30	
2	2,43	0,34	
3	2,97	1,33	
4	3,19	1,01	
5	3,38	1,19	
6	3,75	1,03	
7	3,95	2,45	
8	4,19	29,07	1. Enantiomer Diastereomer 1
9	4,52	23,05	1. u. 2. Enantiomer Diastereomer 2
10	4,96	0,33	
11	5,49	0,13	
12	5,91	0,37	
13	6,13	1,08	
14	6,96	3,26	
15	7,73	0,16	
16	8,27	2,42	
17	8,85	29,77	2. Enantiomer Diastereomer 1
18	11,43	2,44	
19	12,81	0,10	

Peak #	Ret. Time	Area %	Name
20	13,40	0,16	
Total		100,00	

***anti*-1-Phenyl-4-((triethylsilyl)oxy)hept-6-en-3-ol 18b.** Prepared according to representative procedure E (-20 °C). Purified by flash chromatography (SiO_2, hexane/ethyl acetate 97:3 to 95:5) to give diol **18b** as a light yellow oil (74 mg, 79% yield, >10:1 dr, 82% ee).

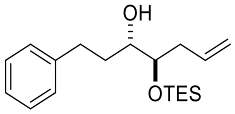

^{1}H NMR (400 MHz, C_6D_6) δ = 7.31-7.26 (m, 2H), 7.23-7.16 (m, 3H), 5.80 (ddt, J = 17.0, 10.0, 7.0 Hz, 1H), 5.08-5.00 (m, 2H), 3.68 (ddd, J = 7.5, 5.0, 3.5 Hz, 1H), 3.59 (dt, J = 8.0, 3.5 Hz, 1H), 2.88 (ddd, J = 14..5, 9.0, 6.0 Hz, 1H), 2.65 (ddd, J = 14.0, 9.0, 7.5 Hz, 1H), 2.30 (dtt, J = 14.5, 7.0, 1.5 Hz, 1H), 2.25-2.09 (br. s, 1H, O-H), 2.24-2.16 (m, 1H), 1.74 (ddt, J = 9.5, 7.5, 4.5 Hz, 1H), 0.94 (t, J = 8.0 Hz, 9H), 0.59 (q, J = 8.0 Hz, 6H); ^{13}C NMR (101 MHz, $CDCl_3$) δ = 142.3, 135.4, 128.6, 128.5, 126.0, 117.1, 75.2, 74.0, 36.4, 33.6, 32.6, 7.0, 5.2; IR (ATR): ṽ = 3455, 2953, 2876, 1641, 1455, 1239, 1074, 1003, 911, 724, 698; LRMS *m*/*z* (ESI$^+$) $[M+Na]^+$ 343; HRMS (ESI$^+$, *m*/*z*) calculated for $[C_{19}H_{32}O_2SiNa]^+$ 343.2064, found 343.2061. The shown absolute configuration was assigned by analogy (see above).

The ee of **18b** was determined by 2D HPLC analysis

Separation of diastereomers: 50 mm Zorbax Eclipse Plus C18, ∅ 4.6 mm, methanol/water gradient 70% to 90% over 5 minutes, v = 1.0 mL/min, λ = 220 nm, t(major) = 5.73 min, t(minor) = 5.83 min, 308 K.

¹D chromatogram(s)

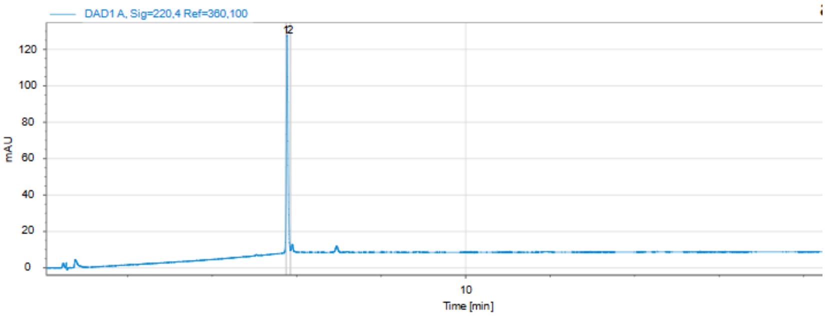

Sampling table (¹D)

Cut group	Cut #	¹D Cut start [min]	¹D Ret. time [min]	¹D Duration [min]	Trigger	²D Run start [min]	
	1	5.73	***	0.04	Time	5.77	1st Diastereomer
	2	5.83	***	0.04	Time	27.62	2nd Diastereomer

Component table

Signal: DAD2 A, Sig=220,4 Ref=360,100

Component	¹D Sampling range [min]	Ret.Time ²D [min]	Area	Area%
1	5.73 - 5.77	13.665	805.865	83.570
2	5.73 - 5.77	15.063	81.765	8.479
3	5.83 - 5.87	13.312	12.447	1.291
4	5.83 - 5.87	13.721	64.217	6.660

18b: separation of diastereomers on achiral column

¹D chromatogram(s)

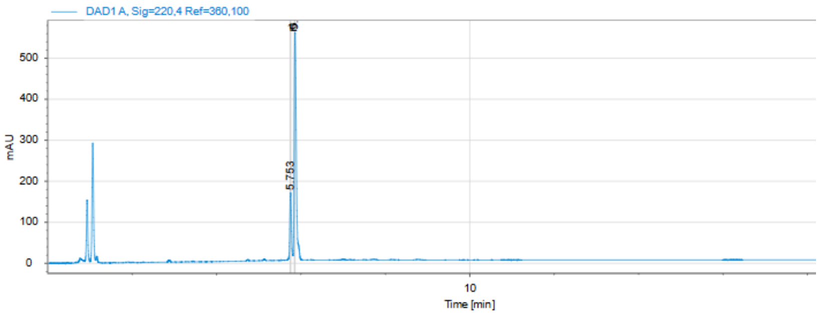

Sampling table (¹D)

Cut group	Cut #	¹D Cut start [min]	¹D Ret. time [min]	¹D Duration [min]	Trigger	²D Run start [min]	
	1	5.73	5.753	0.04	Time	5.77	1st Diastereomer
	2	5.83	5.859	0.04	Time	27.62	2nd Diastereomer

Component table

Signal: DAD2 A, Sig=220,4 Ref=360,100

Component	¹D Sampling range [min]	Ret.Time ²D [min]	Area	Area%
1	5.73 - 5.77	13.648	1065.157	12.577
2	5.73 - 5.77	14.962	1053.460	12.439
3	5.83 - 5.87	13.877	3151.375	37.210
4	5.83 - 5.87	14.359	3199.265	37.775

rac-**18b:** separation of diastereomers on achiral column

Chiral separation of major diastereomer: Daicel 150 mm Chiralpak IB-N-3, ∅ 4.6 mm, n-heptane/i-propanol = 99:1, v = 1.0 mL/min, λ = 210 nm, t(major) = 3.68 min, t(minor) = 7.61 min.

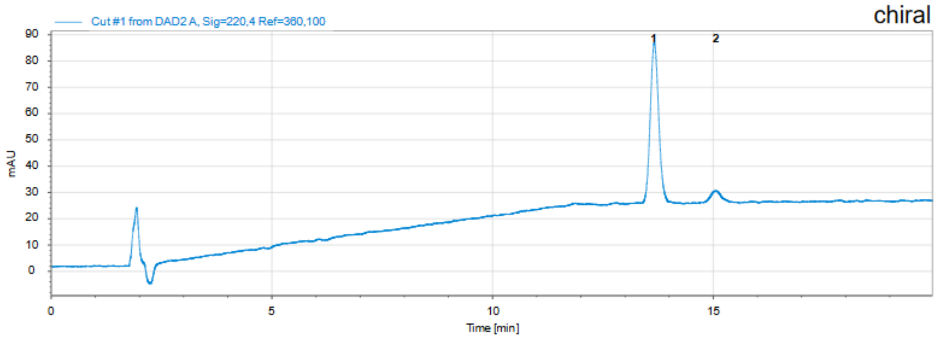

Signal: DAD2 A, Sig=220,4 Ref=360,100

Compound	Cut	Ret.Time	Area	Width	Height	Symmetry	
1	1	13.665	805.865<br>**805.865**	0.220	60.924	0.889	1st Enantiomer
2	1	15.063	81.765<br>**81.765**	0.296	4.607	1.081	2nd Enantiomer

ee = 81.6 %

18b: ee determination of pure major diastereomer

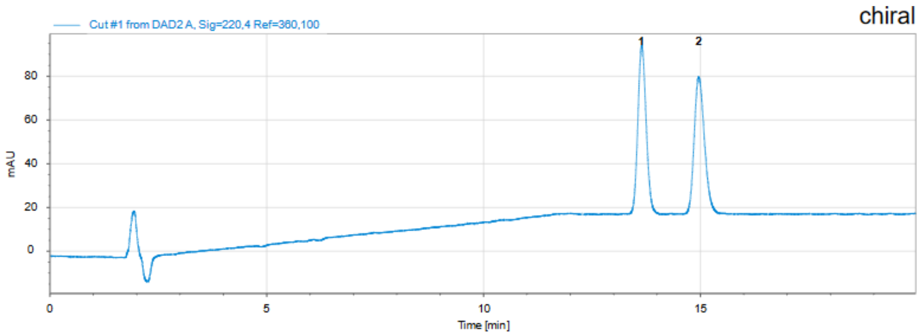

Signal: DAD2 A, Sig=220,4 Ref=360,100

Compound	Cut	Ret.Time	Area	Width	Height	Symmetry	
1	1	13.648	1065.157<br>**1065.157**	0.227	78.136	0.880	1st Enantiomer
2	1	14.962	1053.460<br>**1053.460**	0.278	63.108	0.782	2nd Enantiomer

rac-**18b**: separation of enantiomers of pure major diastereomer

***anti*-2-((*tert*-Butyldimethylsilyl)oxy)-1-cyclohexylpent-4-en-1-ol 18c.** Prepared according

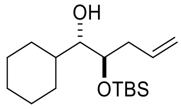

to representative procedure E (-20 °C). Purified by flash chromatography (SiO_2, hexane/ethyl acetate 99:1 to 98:2) to give diol **18c** as a light yellow oil (51 mg, 59% yield, 20:1 dr, 90% ee). $[\alpha]_D^{20}$ = 6.1 (c = 0.49, $CHCl_3$); ^{1}H NMR (400 MHz, $CDCl_3$) δ = 5.84 (ddt, *J* = 17.0, 10.0, 7.0 Hz, 1H), 5.10-5.01 (m, 2H), 3.79 (dt, *J* = 8.0, 3.5 Hz, 1H), 3.29 (dd, *J* = 8.5, 3.5 Hz, 1H), 2.30 (app. dddt, *J* = 14.5, 8.5, 7.0, 1.0 Hz, 1H), 2.23-2.15 (m, 1H), 2.10-2.02 (m, 1H), 1.78-1.62 (m, 3H), 1.59-1.51 (m, 1H), 1.47-1.36 (m, 1H), 1.31-1.13 (m, 3H), 1.06-0.96 (tt, *J* = 11.0, 3.0 Hz, 2H), 0.90 (s, 9H), 0.06(2) (s, 3H), 0.05(7) (s, 3H); ^{13}C NMR (101 MHz, $CDCl_3$) δ = 136.1, 116.9, 78.9, 73.0, 39.3, 35.0, 29.7, 28.8, 26.6, 26.1, 26.0, 25.9, 18.2, -4.2, -4.4; IR (ATR): ṽ = 3577, 2926, 2854, 1450, 1362, 1253, 1065, 911, 832, 773; LRMS *m*/*z* (ESI^+) $[M+Na]^+$ 321; HRMS (ESI^+, *m*/*z*) calculated for $[C_{17}H_{34}O_2SiNa]^+$ 321.2220, found 321.2218. The shown absolute configuration was assigned by analogy (see above).

The ee of **18c** was determined by chiral GC analysis

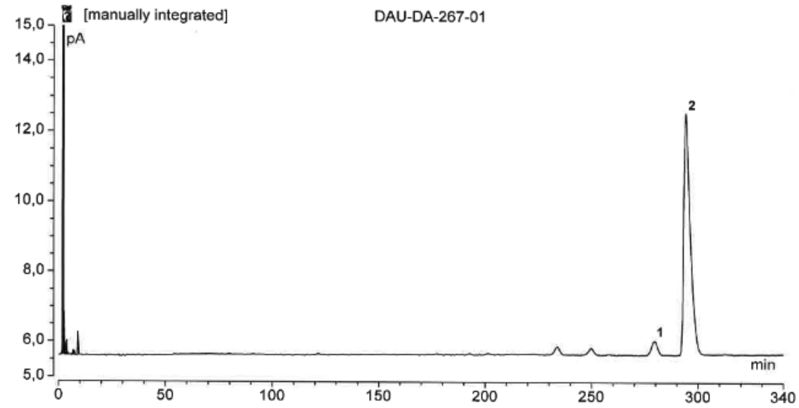

Sample:	**DAU-DA-267-01**	Instrument:	**GC_413**
Sequenz:	**8090 DAU-DA Kes**	Measured:	**04.03.21 15:46**
Sequenz date:	**04.03.21**	Processing M.:	ee DAU-DA
		Report-File:	ee 267-01

chirale Messung, Verhältnis der Enantiomere
Zuordnung siehe achirale GC-MS des Racemates 27800 DAU-DA-181-01 20/7709

No.	Ret.Time min	Rel.Area %	Peak Name
1	279,48	5,09 .	
2	294,11	94,91 .	

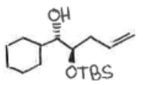

Instrument parameters:

Column:	25,0 m	Hydrodex-beta-TBDAc-CD 0,25/?df G/681
Temperature:	220/ 100 iso/ 350 Split 80	
Gas:	0,50 bar	Hydrogen
Sample size:	1,0 µL	

18c: 90% ee

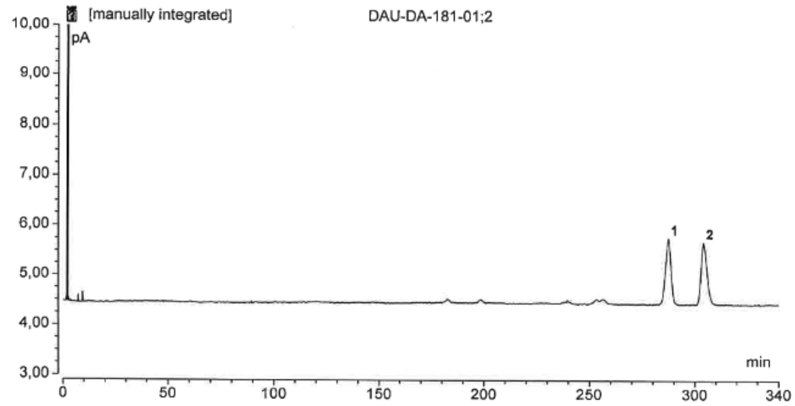

Sample:	**DAU-DA-181-01;2**	Instrument:	**GC_413**
Sequenz:	**7709 DAU-DA VD**	Measured:	**20.10.20 09:20**
Sequenz date:	**19.10.20**	Processing M.:	ee DAU-DA
		Report-File:	ee 181-01

chirale Messung des Racemates, Verhältnis der Enantiomere
Zuordnung siehe achirale GC-MS 27800 DAU-DA-181-01 20/7709

No.	Ret.Time min	Rel.Area %	Peak Name
1	287,46	50,04 .	
2	304,07	49,96 .	

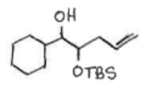

Instrument parameters:

Column:	25,0 m	Hydrodex-beta-TBDAc-CD 0,25/?df G/681
Temperature:	220/ 100 iso/ 350	
Gas:	0,50 bar	Hydrogen
Sample size:	0,2 µL	

rac-**18c:** separation of enantiomers

***anti*-1-Cyclohexyl-2-((triisopropylsilyl)oxy)pent-4-en-1-ol 18d.** Prepared according to representative procedure E (-20 °C). Purified by flash chromatography (SiO_2, hexane/ethyl acetate 99:1) to give diol **18d** as a colourless oil (52 mg, 52% yield, 20:1 dr, 92% ee). $[\alpha]_D^{20}$ = 7.8 (c = 0.45, $CHCl_3$); ^1H NMR (400 MHz, $CDCl_3$) δ = 5.93 (ddt, *J* = 17.0, 10.0, 7.0 Hz, 1H), 5.08 (app. dq, *J* = 17.0, 1.5 Hz, 1H), 5.02 (ddt, *J* = 10.0, 2.0, 1.0 Hz, 1H), 4.00 (td, *J* = 5.5, 3.0 Hz, 1H), 3.38 (dd, *J* = 9.0, 3.0 Hz, 1H), 2.40 (br. s, 1H), 2.34-2.28 (m, 2H), 2.15-2.07 (m, 1H), 1.78-1.62 (m, 3H), 1.58-1.52 (m, 1H), 1.48-1.37 (m, 1H), 1.27-1.16 (m, 3H), 1.09-1.06 (m, 21H), 1.00-0.90 (m, 2H); ^{13}C NMR (101 MHz, $CDCl_3$) δ = 136.1, 116.8, 79.3, 73.2, 39.5, 35.6, 30.1, 28.7, 26.6, 26.0, 25.9, 18.3, 12.8; IR (ATR): ṽ = 3576, 2923, 2853, 1707, 1641, 1463, 1450, 1385, 1366, 1296, 1256, 1079, 1061, 995, 912, 882, 750, 676; LRMS m/z (ESI$^+$) $[M+Na]^+$ 363; HRMS (ESI$^+$, m/z) calculated for $[C_{20}H_{40}O_2SiNa]^+$ 363.2690, found 363.2689. The shown absolute configuration was assigned by analogy (see above).

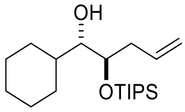

The ee of **18d** was determined by chiral GC analysis

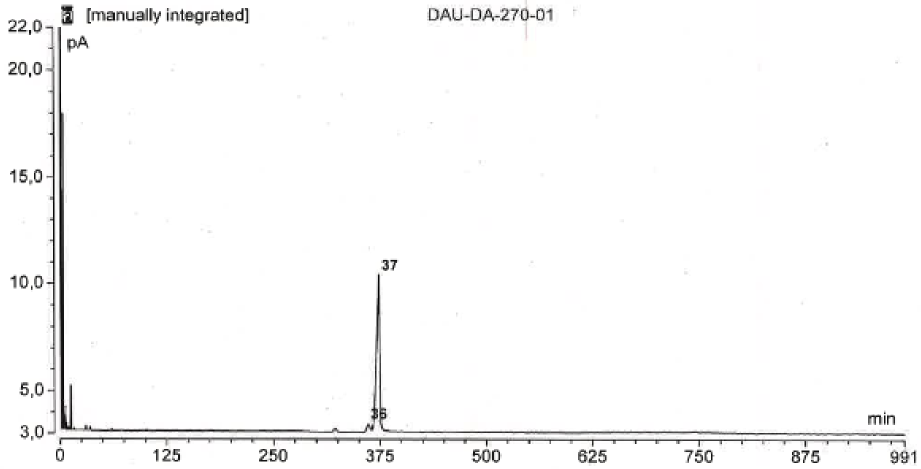

Sample: DAU-DA-270-01
Sequenz: 8093 DAU-DA SE
Sequenz date: 05.03.21
Instrument: GC_411
Measured: 08.03.21 14:16
Processing M.: DAU-DA
Report-File: Verhältnis 270-01

Verhältnis der Enantiomere
Zuordnung achiral nach Racemat GCMS 30077 DAU-DA-253-01 21/8093

No.	Ret.Time min	Rel.Area %	Peak Name
36	360,59	3,85	.
37	373,69	96,15	.

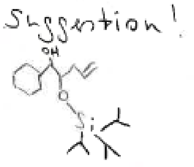

Instrument parameters:
Column: 24,5 m IVADEX-1 0,25/0,25df G/662
Temperature: 220/120 iso/350
Gas: 0,50 bar Hydrogen
Sample size: 0,2 µL

18d: 92% ee

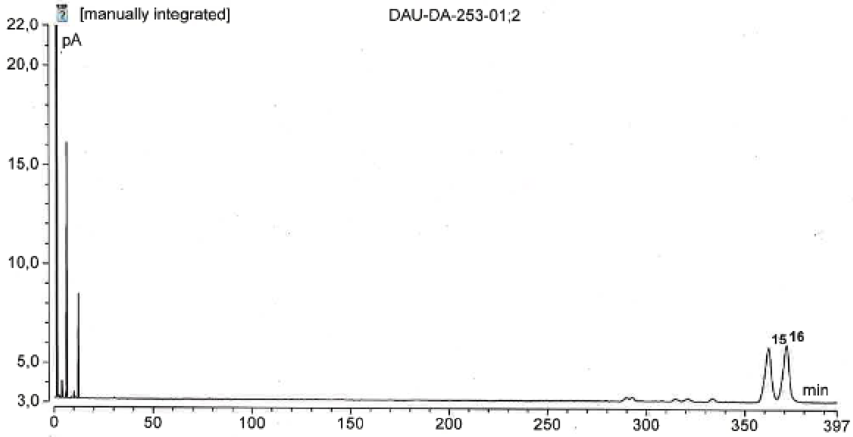

Sample:	**DAU-DA-253-01;2**	Instrument:	**GC_411**
Sequenz:	**8093 DAU-DA SE**	Measured:	**08.03.21 07:19**
Sequenz date:	**05.03.21**	Processing M.:	DAU-DA
		Report-File:	Verhältnis 253-01

Racemat
Verhältnis der Enantiomere
Zuordnung achiral nach GCMS 30077 DAU-DA-253-01 21/8093

No.	Ret.Time min	Rel.Area %	Peak Name
15	362,04	49,06	.
16	371,08	50,94	.

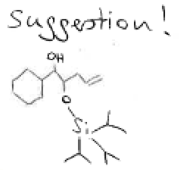

Instrument parameters:

Column:	24,5 m	IVADEX-1 0,25/0,25df G/662
Temperature:	220/120 iso/350	
Gas:	0,50 bar	Hydrogen
Sample size:	0,2 µL	

rac-**18d**: separation of enantiomers

***anti*-1-([1,1'-Biphenyl]-4-yl)-2-((triisopropylsilyl)oxy)pent-4-en-1-ol 18e.** Prepared according to representative procedure E (-40 °C). Purified by flash chromatography (SiO_2, hexane/ethyl acetate 98:2) to give diol **18e** as a light yellow oil (74 mg, 62% yield, >20:1 dr, 93% ee). $[\alpha]_D^{20}$ = -4.0 (c = 0.48, $CHCl_3$); ^{1}H NMR (400 MHz, $CDCl_3$) δ = 7.64 (m, 4H), 7.48-7.43 (m, 4H), 7.39-7.33 (m, 1H), 5.83-5.71 (m, 1H), 5.03-4.91 (m, 3H), 4.19 (td, J = 5.5, 4.0 Hz, 1H), 2.80 (br. s, 1H), 2.37-2.28 (m, 1H), 2.21-2.13 (m, 1H), 1.18-1.11 (m, 21H); ^{13}C NMR (101 MHz, $CDCl_3$) δ = 141.1, 140.3, 139.3, 135.3, 128.9, 127.3, 127.2, 127.0, 126.9, 117.1, 76.5(2), 76.4(8), 36.0, 18.3(3), 18.3(1), 12.8; IR (ATR): ṽ = 3572, 3030, 2942, 2865, 1642, 1600, 1487, 1462, 1387, 1322, 1244, 1181, 1093, 1063, 996, 912, 882, 828, 763, 677; HRMS (ESI$^+$, *m*/*z*) calculated for $[C_{26}H_{38}O_2SiNa]^+$ 433.2533, found 433.2534. The shown absolute configuration was assigned by analogy (see above). The ee of **18e** was determined by 2D HPLC analysis. Separation of diastereomers: 50 mm Eclipse Plus C18, ∅ 4.6 mm, methanol/water 85:15, v = 1.0 mL/min, λ = 220 nm, t(major) = 11.8 min, t(minor) = 12.6 min, 308 K.

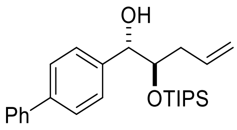

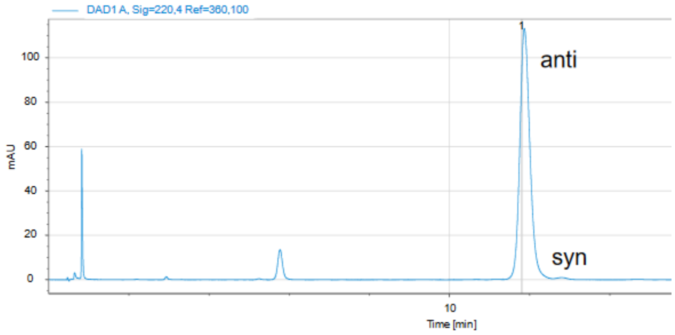

Sampling table (¹D)

Cut group	Cut #	¹D Cut start [min]	¹D Ret. time [min]	¹D Duration [min]	Trigger	²D Run start [min]
	1	11.78	***	0.04	Peak	11.83

18e: separation of diastereomers on achiral column

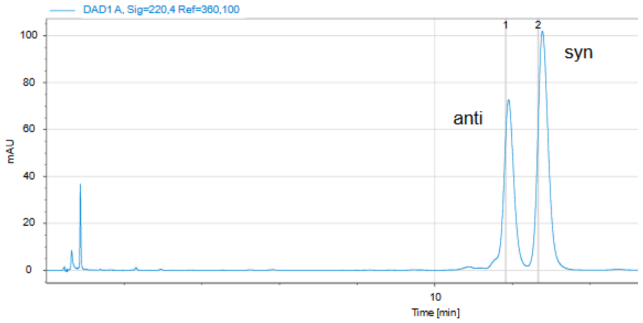

Sampling table (¹D)

Cut group	Cut #	¹D Cut start [min]	¹D Ret. time [min]	¹D Duration [min]	Trigger	²D Run start [min]
	1	11.81	***	0.04	Peak	11.85
	2	12.64	***	0.04	Peak	28.75

rac-**18e:** separation of diastereomers on achiral column

Chiral separation of major diastereomer: 150 mm Chiralpak IC-3, ∅ 4.6 mm, acetonitrile/water = 70:30, v = 1.0 mL/min, λ = 220 nm, t(minor) = 8.37 min, t(major) = 9.23 min. 318 K

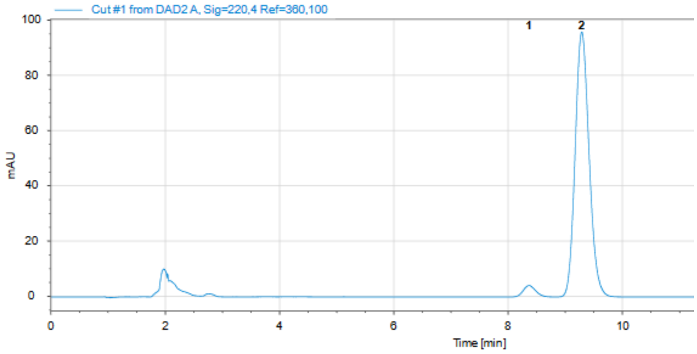

Signal: DAD2 A, Sig=220,4 Ref=360,100

Compound	Cut	Ret.Time	Area	Width	Height	Symmetry
1	1	8.367	62.280<br>**62.280**	0.179	4.104	0.868
2	1	9.289	1621.640<br>**1621.640**	0.250	95.886	0.863

Component	¹D Sampling range [min]	Ret.Time ²D [min]	Area	Area%	
1	11.78 - 11.82	8.367	62.280	3.699	
2	11.78 - 11.82	9.289	1621.640	96.301	= 92.6 % ee

18e: ee determination of pure major diastereomer

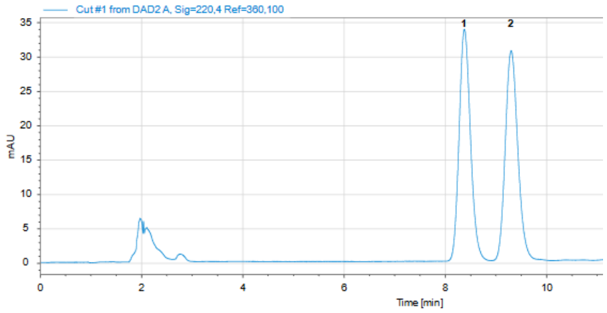

Signal: DAD2 A, Sig=220,4 Ref=360,100

Compound	Cut	Ret.Time	Area	Width	Height	Symmetry
1	1	8.375	524.938<br>**524.938**	0.211	33.699	0.823
2	1	9.299	523.481<br>**523.481**	0.226	30.535	0.840

rac-**18e**: separation of enantiomers

***anti*-1-(4-Methoxyphenyl)-2-((triisopropylsilyl)oxy)pent-4-en-1-ol 18f.** Prepared according to representative procedure E (-30 °C). Purified by flash chromatography (SiO_2, hexane/ethyl acetate 98:2 to 95.5:4:5) to give diol **18f** as a colourless oil (51 mg, 48% yield, >20:1 dr, 93% ee). $[\alpha]_D^{20}$ = 4.3 (c = 0.49, $CHCl_3$); ^1H NMR (400 MHz, $CDCl_3$) δ = 7.30-7.27 (m, 2H), 6.90-6.85 (m, 2H), 5.59 (ddt, *J* = 16.0, 11.5, 7.0 Hz, 1H), 4.96-4.90 (m, 2H), 4.81 (d, *J* = 3.5 Hz, 1H), 4.08 (td, *J* = 6.0, 3.5 Hz, 1H), 3.81 (s, 3H), 2.23 (app. dddt, *J* = 14.5, 7.5, 6.0, 1.5 Hz, 1H), 2.08 (app. dddt, *J* = 14.5, 7.5, 6.0, 1.5 Hz, 1H), 1.13-1.06 (m, 21H); ^{13}C NMR (101 MHz, $CDCl_3$) δ = 159.0, 135.4, 132.4, 127.7, 117.0, 113.7, 76.6, 76.3, 55.4, 36.0, 18.3(3), 18.3(2), 12.8; IR (ATR): ṽ = 3571, 3075, 2943, 2866, 1613, 1513, 1463, 1246, 1172, 1094, 1063, 1036, 997, 913, 882, 825, 676; LRMS *m/z* (EI$^+$) [M]$^+$ 364; HRMS (ESI$^+$, *m/z*) calculated for $[C_{21}H_{36}O_3SiNa]^+$ 387.2326, found 387.2326. The shown absolute configuration was assigned by analogy (see above).

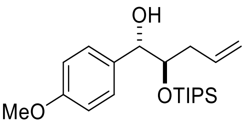

The ee of **18f** was determined by HPLC analysis: 150 mm Chiralpak IA-3, ∅ 4.6 mm, *n*-heptane/i-propanol = 99.5:0.5, v = 1.0 mL/min, λ = 225 nm, t(major) = 7.26 min, t(minor) = 10.25 min.

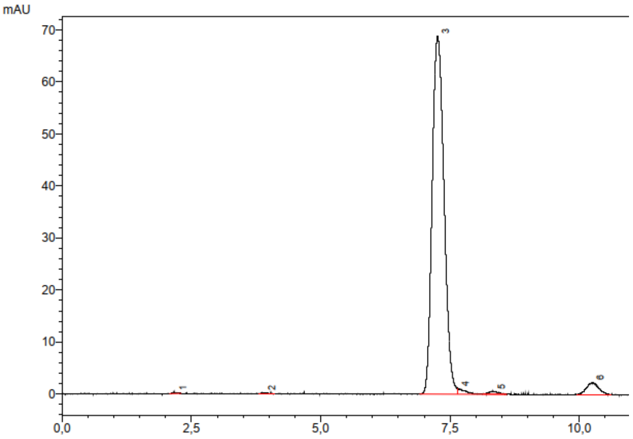

1 225nm,4nm

PDA Ch1 225nm

Peak #	Ret. Time	Area %	Name
1	2,18	0,13	
2	3,90	0,18	
3	7,26	94,77	1. Enantiomer Diastereomer 1 93.2 % ee
4	7,65	1,04	1. u. 2. Enantiomer Diastereomer 2
5	8,34	0,57	
6	10,25	3,31	2. Enantiomer Diastereomer 1
Total		100,00	

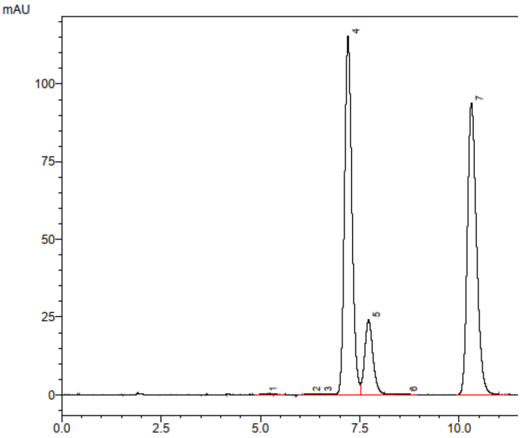

1 225nm,4nm

PDA Ch1 225nm

Peak #	Ret. Time	Area %	Name
1	5.11	0.25	
2	6.21	0.12	
3	6.50	0.21	
4	7.20	44.37	1. Enantiomer Diastereomer 1
5	7.72	10.54	1. u. 2. Enantiomer Diastereomer 2
6	8.65	0.14	
7	10.31	44.37	2. Enantiomer Diastereomer 1
Total		100.00	

***anti*-1-(2-Methylphenyl)-2-((triisopropylsilyl)oxy)pent-4-en-1-ol 18g.** Prepared according to representative procedure E (-40 °C). Purified by flash chromatography (SiO_2, hexane/ethyl acetate 99:1 to 98.5:1.5) with remaining aldehyde removed under high vacuum overnight to give diol **18g** as a light yellow oil (53 mg, 52% yield, >20:1 dr, 91% ee). $[\alpha]_D^{20}$ = -12.5 (c = 0.52, $CHCl_3$); ^{1}H NMR (400 MHz, $CDCl_3$) δ = 7.63 (dd, *J* = 7.5, 1.5 Hz, 1H), 7.22 (td, *J* = 7.5, 1.5 Hz, 1H), 7.16 (td, *J* = 7.5, 1.5 Hz, 1H), 7-10 (dd, *J* = 7.5, 1.5 Hz, 1H), 5.67-5.56 (m, 1H), 5.06 (d, *J* = 4.0 Hz, 1H), 4.94-4.88 (m, 2H), 4.14 (td, *J* = 5.5, 4.0 Hz, 1H), 2.80 (br. s, 1H), 2.31 (s, 3H), 2.30-2.15 (m, 2H), 1.12-1.07 (m, 21H); ^{13}C NMR (101 MHz, $CDCl_3$) δ = 138.3, 135.4, 134.6, 130.2, 127.3, 126.7, 126.6, 116.9, 74.1, 73.5, 36.1, 19.6, 18.3, 12.8; IR (ATR): ṽ = 3562, 3075, 2943, 2866, 1640, 1462, 1383, 1244, 1092, 1061, 996, 912, 881, 754, 676; LRMS *m*/*z* (ESI$^+$) [M+Na]$^+$ 371; HRMS (ESI$^+$, *m*/*z*) calculated for $[C_{21}H_{36}O_2SiNa]^+$ 371.2377, found 371.2380. The shown absolute configuration was assigned by analogy (see above). The ee of **18g** was determined by 2D HPLC analysis

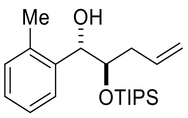

Separation of diastereomers: 150 mm Eclipse Plus C18 1.8 µm, ∅ 4.6 mm, methanol/water 85:15, v = 1.0 mL/min, λ = 220 nm, t(major) = 19.6 min, t(minor) = 20.3 min, 308 K.

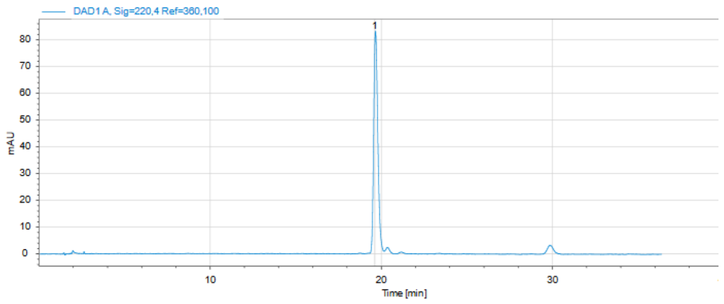

Sampling table (¹D)

Cut group	Cut #	¹D Cut start [min]	¹D Ret. time [min]	¹D Duration [min]	Trigger	²D Run start [min]	
	1	19.60	***	0.04	Time	19.65	1. diastereomer

18g: separation of diastereomers on achiral column

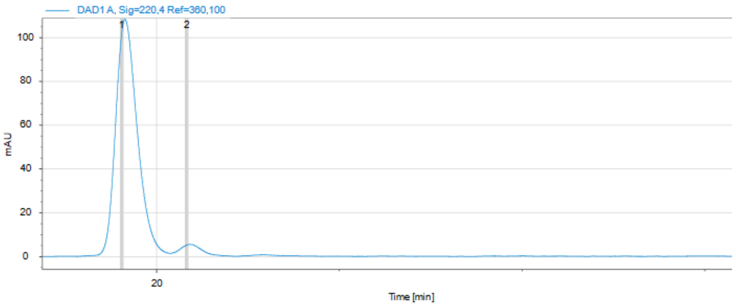

Sampling table (¹D)

Cut group	Cut #	¹D Cut start [min]	¹D Ret. time [min]	¹D Duration [min]	Trigger	²D Run start [min]	
	1	19.60	***	0.04	Time	19.65	1. diastereomer
	2	20.31	***	0.04	Time	39.75	2. diastereomer

rac-**18g:** separation of diastereomers on achiral column

Chiral separation of major diastereomer: 150 mm Chiralpak IBN-3, ∅ 4.6 mm, acetonitrile/water = 70:30, v = 1.0 mL/min, λ = 220 nm, t(minor) = 8.37 min, t(major) = 9.23 min. 298 K

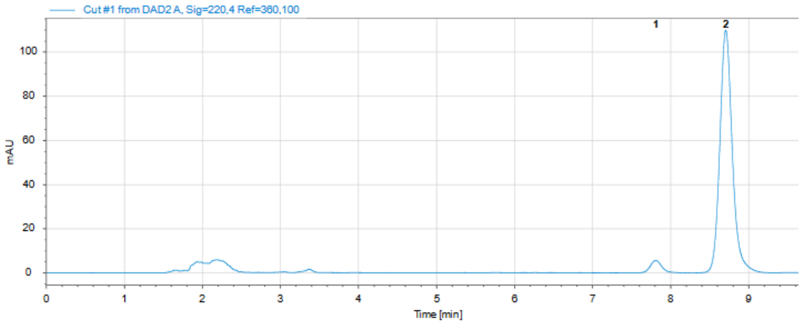

Signal: DAD2 A, Sig=220,4 Ref=360,100

Compound	Cut	Ret.Time	Area	Width	Height	Symmetry
1	1	7.810	58.036 **58.036**	0.170	5.572	0.850
2	1	8.707	1218.026 **1218.026**	0.178	109.022	0.868

Component	¹D Sampling range [min]	Ret.Time ²D [min]	Area	Area%	
1	19.60 - 19.64	7.810	58.036	4.548	1. diastereomer 1. enantiomer
2	19.60 - 19.64	8.707	1218.026	95.452	1. diastereomer 2. enantiomer

ee = 90.9%

18g: ee determination of pure major diastereomer

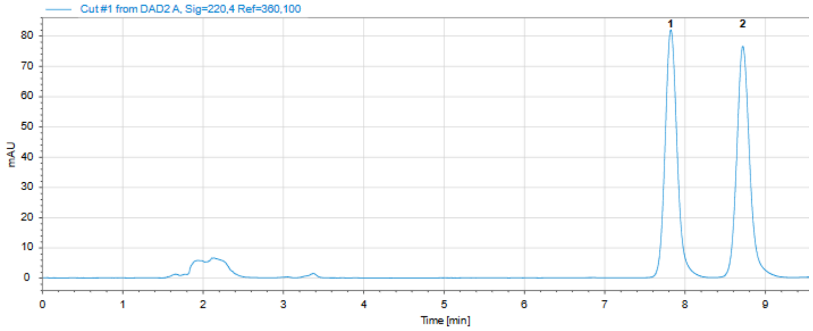

Signal: DAD2 A, Sig=220,4 Ref=360,100

Compound	Cut	Ret.Time	Area	Width	Height	Symmetry
1	1	7.824	856.087 **856.087**	0.171	81.019	0.876
2	1	8.720	859.189 **859.189**	0.180	75.882	0.843

rac-**18g**: separation of enantiomers

***anti*-1-(Naphthalen-2-yl)-2-((triisopropylsilyl)oxy)pent-4-en-1-ol 18h.** Prepared according to representative procedure E (-40 °C). Purified by flash chromatography (SiO_2, hexane/ethyl acetate 99:1 to 98.5:1.5) to give diol **18h** as a colourless oil (74 mg, 66% yield, >20:1 dr, 94% ee). $[\alpha]_D^{20}$ = -16.4 (c = 0.50, $CHCl_3$); ^1H NMR (400 MHz, $CDCl_3$) δ = 7.88-7.80 (m, 4H), 7.50-7.44 (m, 3H), 5.71 (ddt, *J* = 16.5, 11.0, 7.0 Hz, 1H), 5.05 (d, *J* = 3.5 Hz, 1H), 4.94-4.87 (m, 2H), 4.24 (ddd, *J* = 6.5, 5.5, 3.5 Hz, 1H), 2.87 (br. s, 1H), 2.27 (app. dddt, *J* = 15.0, 7.5, 6.5, 1.5 Hz, 1H), 2.09 (app. dddt, *J* = 15.0, 7.0, 5.5, 1.5 Hz, 1H), 1.16-1.10 (m, 21H); ^{13}C NMR (101 MHz, $CDCl_3$) δ = 137.7, 135.3, 133.4, 133.0, 128.1, 127.9, 127.8, 126.2, 125.9, 125.3, 124.6, 117.1, 76.8, 76.4, 35.9, 18.4, 18.3, 12.8; IR (ATR): ṽ = 3571, 3057, 2942, 2865, 1639, 1463, 1362, 1244, 1094, 1063, 996, 912, 882, 816, 745677, 476; HRMS (ESI^+, *m*/*z*) calculated for $[C_{24}H_{36}O_2SiNa]^+$ 407.2377, found 407.2380. The shown absolute configuration was assigned by analogy (see above).

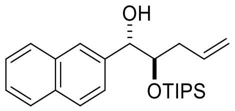

The ee of **18h** was determined by HPLC analysis: 150 mm Chiralpak IC-3, ∅ 4.6 mm, *n*-heptane/*i*-propanol = 99:1, v = 1.0 mL/min, λ = 225 nm, t(minor) = 3.59 min, t(major) = 3.92 min.

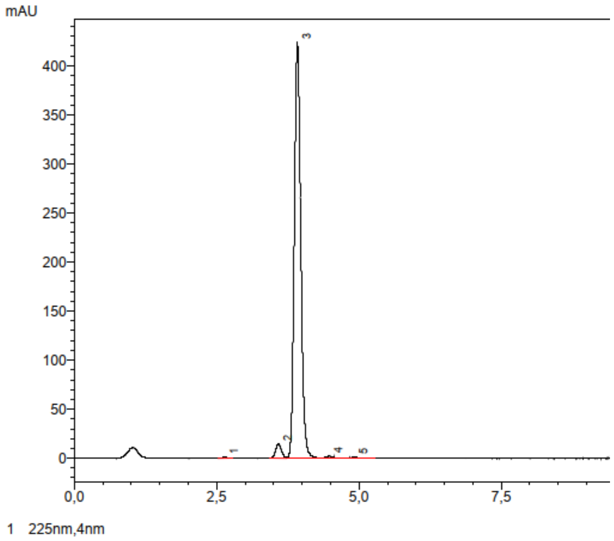

PDA Ch1 225nm

Peak #	Ret. Time	Area %	Name
1	2,64	0,24	
2	3,59	2,95	1. Enantiomer Diastereomer 1
3	3,92	95,08	2. Enantiomer Diastereomer 1 94.0 % ee
4	4,48	0,62	1. Enantiomer Diastereomer 2
5	4,91	0,42	2. Enantiomer Diastereomer 2 + impurity
6	11,19	0,69	
Total		100,00	

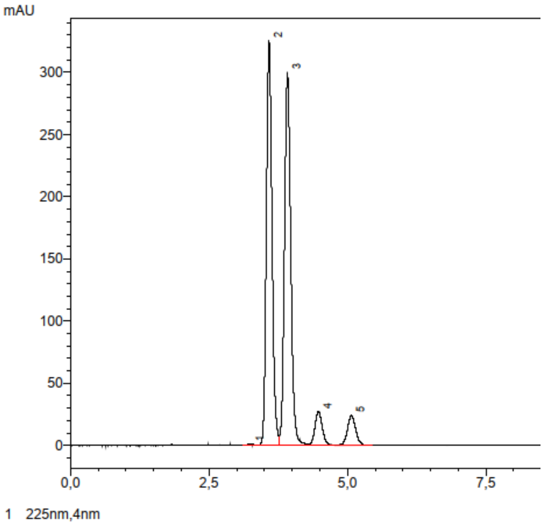

PDA Ch1 225nm

Peak #	Ret. Time	Area %	Name
1	3,25	0,18	
2	3,58	44,79	1. Enantiomer Diastereomer 1
3	3,92	45,40	2. Enantiomer Diastereomer 1
4	4,48	4,84	1. Enantiomer Diastereomer 2
5	5,07	4,79	2. Enantiomer Diastereomer 2
Total		100,00	

***anti*-1-(4-Fluorophenyl)-2-((triisopropylsilyl)oxy)pent-4-en-1-ol 18i.** Prepared according to representative procedure E (-40 °C). Purified by flash chromatography (SiO_2, hexane/ethyl acetate 99:1 to 98:2) to give diol **18i** as a light yellow oil (73 mg, 71% yield, >20:1 dr, 93% ee). $[\alpha]_D^{20}$ = -1.9 (c = 0.54, $CHCl_3$); ^1H NMR (400 MHz, $CDCl_3$) δ = 7.36-7.30 (m, 2H), 7.06-6.99 (m, 2H), 5.68 (ddt, *J* = 17.5, 10.5, 7.0 Hz, 1H), 4.97-4.90 (m, 2H), 4.83 (d, *J* = 4.0 Hz, 1H), 4.09 (td, *J* = 6.0, 4.0 Hz, 1H), 2.74 (br. s, 1H), 2.23 (app. dddt, *J* = 14.5, 7.0, 6.0, 1.5 Hz, 1H), 2.07 (app. dddt, *J* = 14.5, 7.0, 5.5, 1.5 Hz), 1.12-1.08 (m, 21H); ^{13}C NMR (101 MHz, $CDCl_3$) δ = 162.3 (d, $^1J_{CF}$ = 245.0 Hz), 136.0 (d, $^4J_{CF}$ = 3.0 Hz), 135.0, 128.2 (d, $^3J_{CF}$ = 8.0 Hz), 117.2, 115.1 (d, $^2J_{CF}$ = 21.0 Hz), 76.4, 76.1, 36.0, 18.3(1), 18.2(9), 12.8; IR (ATR): ṽ = 3562, 3075, 2943, 2866, 1640, 1462, 1383, 1244, 1092, 1061, 996, 912, 881, 754, 676; HRMS (ESI^+, *m/z*) calculated for $[C_{20}H_{33}F_3O_2SiNa]^+$ 375.2126, found 375.2129. The shown stereostructure was assigned by conversion into a compound of known absolute configuration (see above).

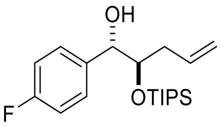

The ee of **18i** was determined by 2D HPLC analysis

Separation of diastereomers: 50 mm Phenyl Hexyl, ∅ 4.6 mm, methanol/water 80:20, v = 1.0 mL/min, λ = 220 nm, t(major) = 3.39 min, t(minor) = 3.66 min, 308 K.

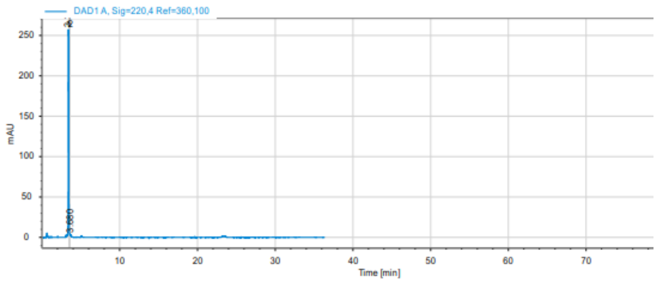

Sampling table (¹D)

Cut group	Cut #	¹D Cut start [min]	¹D Ret. time [min]	¹D Duration [min]	Trigger	²D Run start [min]	
	1	3.39	3.396	0.04	Time	3.44	1. diastereomer
	2	3.66	3.680	0.04	Time	57.44	2. diastereomer

18i: separation of diastereomers on achiral column

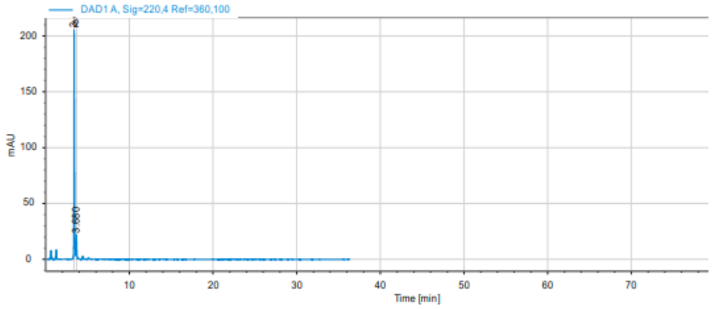

Sampling table (¹D)

Cut group	Cut #	¹D Cut start [min]	¹D Ret. time [min]	¹D Duration [min]	Trigger	²D Run start [min]	
	1	3.39	3.400	0.04	Time	3.44	1. diastereomer
	2	3.66	3.680	0.04	Time	57.44	2. diastereomer

rac-**18i:** separation of diastereomers on achiral column

Chiral separation of major diastereomer: 150 mm Chiralpak AS-3R, ∅ 4.6 mm, acetonitrile/water = 70:30, v = 1.0 mL/min, λ = 220 nm, t(major) = 12.94 min, t(minor) = 14.24 min. 298 K

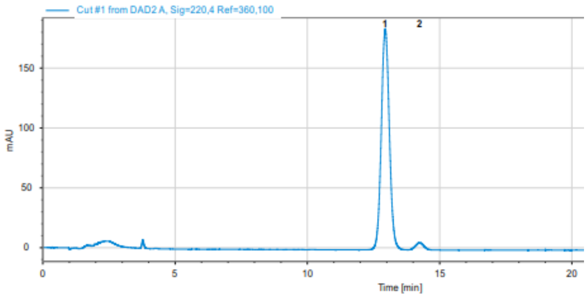

Signal: DAD2 A, Sig=220,4 Ref=360,100

Compound	Cut	Ret.Time	Area	Width	Height	Symmetry
1	1	12.944	3911.805<br>**3911.805**	0.335	184.522	0.914
2	1	14.244	136.702<br>**136.702**	0.347	6.153	0.962

Component	¹D Sampling range [min]	Ret.Time ²D [min]	Area	Area%	
1	3.39 - 3.43	12.944	3911.805	95.497	1. diastereomer 1. enantiomer
2	3.39 - 3.43	14.244	136.702	3.337	1. diastereomer 2. enantiomer
3	3.66 - 3.70	12.942	47.733	1.165	

ee = 93.2%

18i: ee determination of pure major diastereomer

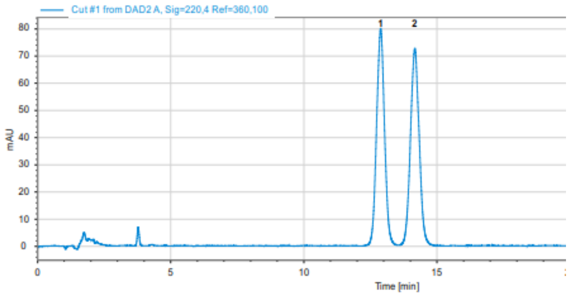

Signal: DAD2 A, Sig=220,4 Ref=360,100

Compound	Cut	Ret.Time	Area	Width	Height	Symmetry
1	1	12.882	1660.094<br>**1660.094**	0.331	79.734	0.976
2	1	14.165	1665.501<br>**1665.501**	0.364	72.507	0.942

rac-**18i**: separation of enantiomers

***anti*-1-(3-Chlorophenyl)-2-((triisopropylsilyl)oxy)pent-4-en-1-ol 18j.** Prepared according to representative procedure E (-40 °C). Purified by flash chromatography (SiO_2, hexane/ethyl acetate 98:2) to give diol **18j** as a colourless oil (75 mg, 70% yield, >20:1 dr, 87% ee). $[\alpha]_D^{20}$ = -23.8 (c = 0.52, $CHCl_3$); ^{1}H NMR (400 MHz, $CDCl_3$) δ = 7.39-7.37 (m, 1H), 7.27-7.20 (m, 3H), 5.70 (ddt, *J* = 17.5, 10.5, 7.0 Hz, 1H), 4.99-4.91 (m, 2H), 4.82 (d, *J* = 4.0 Hz, 1H), 4.10 (td, *J* = 6.0, 4.0 Hz, 1H), 2.75 (br. s, 1H), 2.24 (app. dddt, *J* = 14.5, 7.0, 6.0, 1.5 Hz, 1H), 2.07 (app. dddt, *J* = 14.5, 7.0, 6.0, 1.5 Hz, 1H), 1.12-1.07 (m, 21H); ^{13}C NMR (101 MHz, $CDCl_3$) δ = 142.4, 134.9, 134.4, 129.6, 127.7, 126.8, 124.7, 117.3, 76.2, 76.1, 36.1, 18.3(1), 18.2(8), 12.8; IR (ATR): ṽ = 3567, 2943, 2866, 1599, 1575, 1464, 1431, 1192, 1095, 1063, 997, 914, 881, 783, 678; LRMS *m/z* (ESI^+) $[M+Na]^+$ 391; HRMS (ESI^+, *m/z*) calculated for $[C_{20}H_{33}O_2{}^{35}ClSiNa]^+$ 391.1831, found 391.1833. The shown absolute configuration was assigned by analogy (see above).

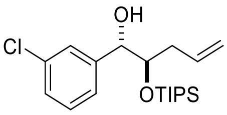

The ee of **18j** was determined by 2D HPLC analysis

Separation of diastereomers: 50 mm Phenyl Hexyl, ∅ 3.0 mm, methanol/water 75:25, v = 0.5 mL/min, λ = 220 nm, t(major) = 11.30 min, t(minor) = 12.08 min, 308 K.

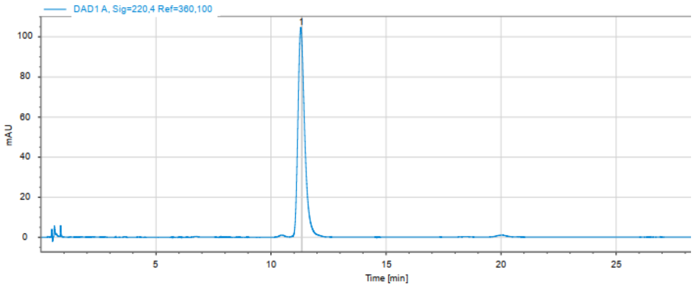

Sampling table (¹D)

Cut group	Cut #	¹D Cut start [min]	¹D Ret. time [min]	¹D Duration [min]	Trigger	²D Run start [min]	
	1	11.30	***	0.08	Time	11.39	**1st diastereomer**

18j: separation of diastereomers on achiral column

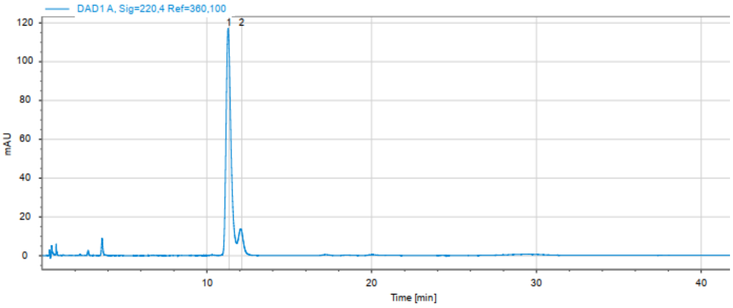

Sampling table (¹D)

Cut group	Cut #	¹D Cut start [min]	¹D Ret. time [min]	¹D Duration [min]	Trigger	²D Run start [min]	
	1	11.30	***	0.08	Time	11.39	1st diastereomer
	2	12.08	***	0.08	Time	28.69	2nd diastereomer

rac-**18j:** separation of diastereomers on achiral column

Chiral separation of major diastereomer: 150 mm Chiralpak IB-N3, ∅ 4.6 mm, methanol/water = 80:20, v = 1.0 mL/min, λ = 220 nm, t(major) = 11.52 min, t(minor) = 12.66 min. 298 K

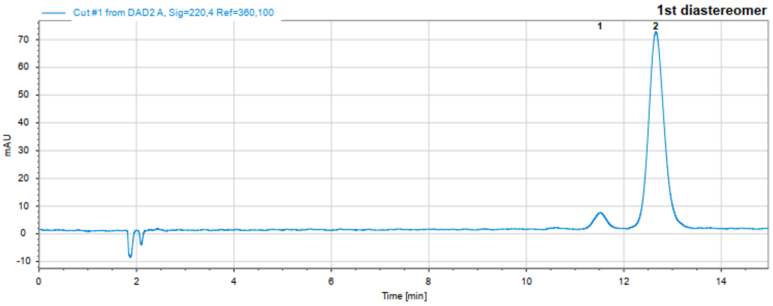

Signal: DAD2 A, Sig=220,4 Ref=360,100

Compound	Cut	Ret.Time	Area	Width	Height	Symmetry	
1	1	11.523	115.896<br>**115.896**	0.229	6.057	1.007	**1st enantiomer**
2	1	12.661	1603.361<br>**1603.361**	0.329	71.283	0.866	**2nd enantiomer**

Component	¹D Sampling range [min]	Ret.Time ²D [min]	Area	Area%		
1	11.30 - 11.38	11.523	115.896	6.741	E1/D1	**ee = 86.5%**
2	11.30 - 11.38	12.661	1603.361	93.259	E2/D1	

18j: ee determination of pure major diastereomer

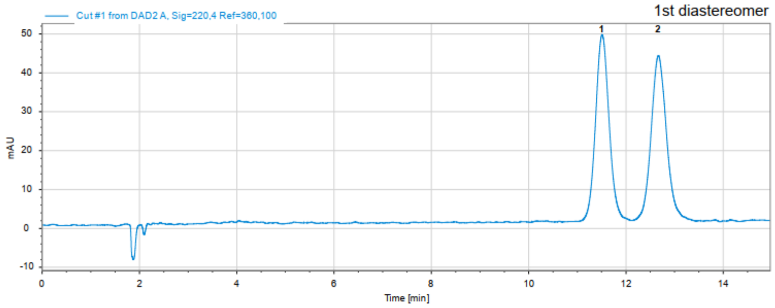

Signal: DAD2 A, Sig=220,4 Ref=360,100

Compound	Cut	Ret.Time	Area	Width	Height	Symmetry	
1	1	11.510	934.491<br>**934.491**	0.299	47.996	0.898	1st enantiomer
2	1	12.667	942.454<br>**942.454**	0.346	42.540	0.873	2st enantiomer

rac-**18j**: separation of enantiomers

***anti*-1-(4-(Trifluoromethyl)phenyl)-2-((triisopropylsilyl)oxy)pent-4-en-1-ol 18k.** Prepared according to representative procedure E (-40 °C). Purified by flash chromatography (SiO_2, hexane/ethyl acetate 98:2) to give diol **18k** as a colourless oil (99 mg, 75% yield, 20:1 dr, 78% ee). $[\alpha]_D^{20}$ = 2.0 (c = 0.51, $CHCl_3$); ^{1}H NMR (400 MHz, $CDCl_3$) δ = 7.60 (d, *J* = 8.0 Hz, 2H), 7.49 (d, *J* = 8.0 Hz, 2H), 5.69 (ddt, *J* = 17.5, 10.5, 7.0 Hz, 1H), 4.98-4.88 (m, 3H), 4.14 (td, *J* = 6.0, 4.0 Hz, 1H), 2.83 (br. s, 1H), 2.24 (app. dddt, *J* = 14.5, 7.0, 6.0, 1.5 Hz, 1H), 2.05 (dddt, *J* = 14.2, 7.0, 5.6, 1.4 Hz, 1H), 1.13-1.08 (m, 21H); ^{13}C NMR (101 MHz, $CDCl_3$) δ = 144.3, 134.8, 129.8 (q, $^2J_{CF}$ = 32.5 Hz), 126.9, 125.2 (q, $^3J_{CF}$ = 4.0 Hz), 124.3 (q, $^1J_{CF}$ = 273.0 Hz), 117.4, 76.2, 76.1, 18.2(9), 18.2(6), 12.8; IR (ATR): ṽ = 3457, 1621, 1464, 1416, 1323, 1164, 1125, 1066, 1017, 917, 882, 827, 679; LRMS m/z (ESI$^-$) [M-H]$^-$ 401; HRMS (ESI$^-$, *m*/*z*) calculated for $[C_{21}H_{32}O_2F_3Si]^-$ 401.2134, found 401.2130. The shown absolute configuration was assigned by analogy (see above).

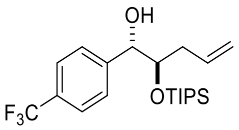

The ee of **18k** was determined by HPLC analysis: 150 mm Chiralpak IA-3, ∅ 4.6 mm, *n*-heptane/*i*-propanol = 98:2, v = 1.0 mL/min, λ = 220 nm, t(major) = 3.02 min, t(minor) = 3.67 min.

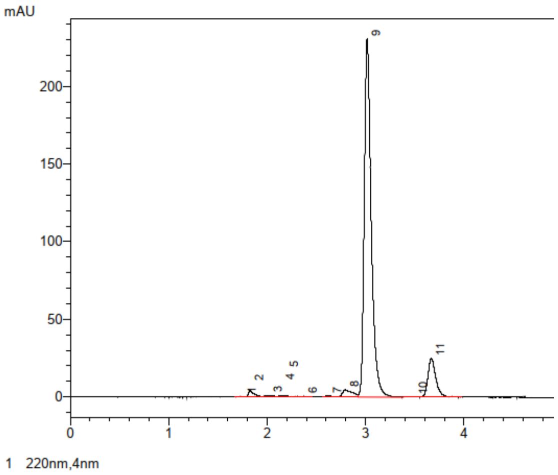

PDA Ch1 220nm

Peak #	Ret. Time	Area %	Name
1	1,75	0,09	
2	1,83	1,29	
3	2,02	0,45	
4	2,14	0,21	
5	2,18	0,34	
6	2,37	0,27	
7	2,62	0,34	
8	2,80	2,43	1. u. 2. Enantiomer Diastereomer 1
9	3,02	84,15	1. Enantiomer 2. Diastereomer 78.1 % ee
10	3,49	0,10	
11	3,67	10,33	2. Enantiomer 2. Diastereomer
Total		100,00	

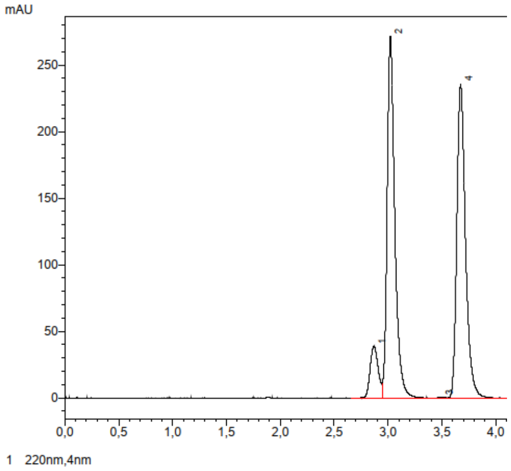

PDA Ch1 220nm

Peak #	Ret. Time	Area %	Name
1	2,87	7,19	1. u. 2. Enantiomer Diastereomer 1
2	3,02	46,77	1. Enantiomer Diastereomer 2
3	3,49	0,11	
4	3,67	45,94	2. Enantiomer Diastereomer 2
Total		100,00	

***anti*-1-(4-(4,4,5,5-Tetramethyl-1,3,2-dioxaborolan-2-yl)phenyl)-2-((triisopropylsilyl)oxy)-pent-4-en-1-ol 18l.** Prepared according to representative procedure E (-40 °C). Purified by flash chromatography (SiO_2, hexane/ethyl acetate 98:2) to give diol **18l** as a colourless oil (86 mg, 64% yield, >20:1 dr, 92% ee). $[\alpha]_D^{20}$ = 4.0 (c = 0.52, $CHCl_3$); ^1H NMR (400 MHz, $CDCl_3$) δ = 7.78 (d, J = 8.0 Hz, 2H), 7.36 (d, J = 8.0 Hz, 2H), 5.68 (ddt, J = 17.5, 10.5, 7.0 Hz, 1H), 4.95-4.87 (m, 3H), 4.15-4.09 (m, 1H), 2.76 (br. s, 1H), 2.20 (app. dddt, J = 15.0, 7.5, 6.5, 1.4 Hz, 1H), 2.06-1.97 (m, 1H), 1.35 (s, 12H), 1.13-1.08 (m, 21H); ^{13}C NMR (101 MHz, $CDCl_3$) Note: one aryl carbon missing due to overlap. δ = 143.3, 135.3, 134.8, 125.8, 117.1, 83.9, 76.7, 76.4, 35.8, 25.1, 25.0, 18.3(3), 18.3(1), 12.8; IR (ATR): ṽ = 3567, 2943, 2866, 1613, 1464, 1399, 1358, 1319, 1144, 1087, 1020, 882, 859, 676, 657; HRMS (ESI^+, *m*/*z*) calculated for $[C_{26}H_{45}O_4BSiNa]^+$ 483.3076, found 483.3072. The shown absolute configuration was assigned by analogy (see above).

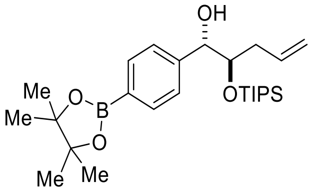

The ee of **18l** was determined by 2D HPLC analysis

Separation of diastereomers: 50 mm Phenyl Hexyl, ∅ 4.6 mm, methanol/water 80:20, v = 1.0 mL/min, λ = 220 nm, t(major) = 6.37 min, t(minor) = 7.07 min, 308 K.

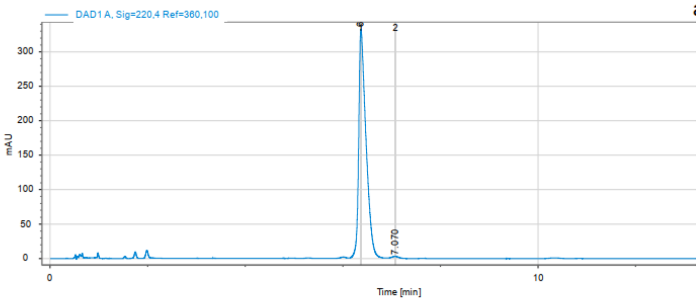

Sampling table (¹D)

Cut group	Cut #	¹D Cut start [min]	¹D Ret. time [min]	¹D Duration [min]	Trigger	²D Run start [min]	
	1	6.35	6.370	0.04	Time	6.39	1st diastereomer
	2	7.06	7.070	0.04	Time	33.24	2nd diastereomer

18l: separation of diastereomers on achiral column

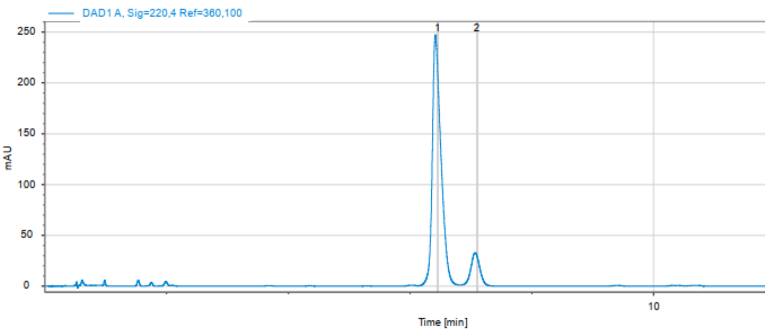

Sampling table (¹D)

Cut group	Cut #	¹D Cut start [min]	¹D Ret. time [min]	¹D Duration [min]	Trigger	²D Run start [min]	
	1	6.43	***	0.04	Time	6.47	1st diastereomer
	2	7.08	***	0.04	Time	33.32	2nd diastereomer

rac-**18l:** separation of diastereomers on achiral column

Chiral separation of major diastereomer: 150 mm Chiralcel OZ-3R, ∅ 4.6 mm, acetonitrile/water = 65:35, v = 1.0 mL/min, λ = 220 nm, t(major) = 12.56 min, t(minor) = 13.43 min. 298 K

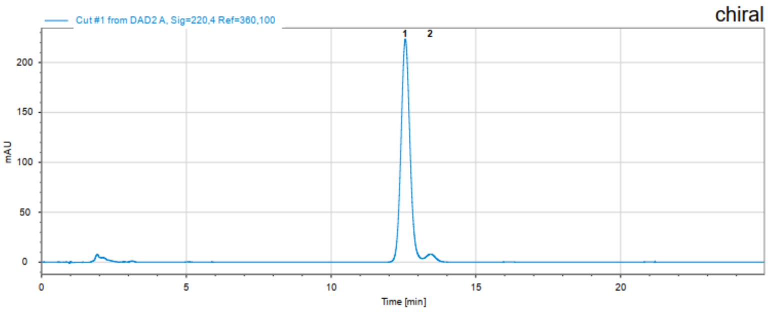

Signal: DAD2 A, Sig=220,4 Ref=360,100

Compound	Cut	Ret.Time	Area	Width	Height	Symmetry	
1	1	12.564	4794.488<br>**4794.488**	0.323	223.377	0.905	1st enantiomer
2	1	13.431	197.623<br>**197.623**	0.285	8.114	0.923	2nd enantiomer

ee = 92.1 %

18l: ee determination of pure major diastereomer

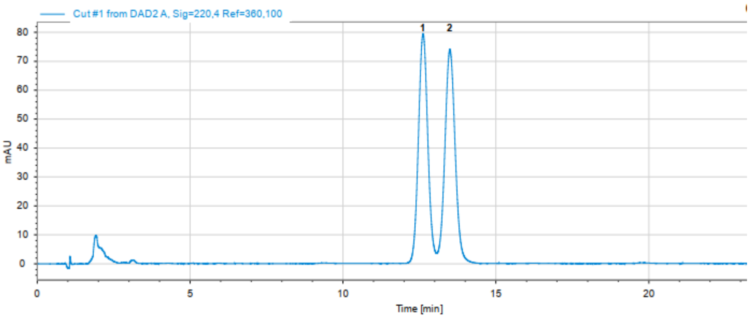

Signal: DAD2 A, Sig=220,4 Ref=360,100

Compound	Cut	Ret.Time	Area	Width	Height	Symmetry	
1	1	12.618	1700.665<br>**1700.665**	0.302	79.416	0.944	1st enantiomer
2	1	13.494	1729.389<br>**1729.389**	0.327	73.925	0.915	2nd enantiomer

rac-**18l**: separation of enantiomers

***anti*-1-(Furan-2-yl)-2-((triisopropylsilyl)oxy)pent-4-en-1-ol 18m.** Prepared according to representative procedure E (–30 °C). Purified by flash chromatography (SiO_2, hexane/ethyl acetate 99:1 to 97:3) to give diol **18m** as a colourless oil (75 mg, 70% yield, >20:1 dr, 94% ee). $[\alpha]_D^{20}$ = –17.5 (c = 0.55, $CHCl_3$); ^{1}H (400 MHz, $CDCl_3$) δ = 7.37-7.35 (m, 1H), 6.35-6.31 (m, 2H), 5.81-5.68 (m, 1H), 5.05-5.97 (m, 2H), 4.80-4.75 (m, 1H), 4.26-4.20 (m, 1H), 2.58 (dd, *J* = 5.0, 1.5 Hz, 1H), 2.35 (ddd, *J* = 14.5, 7.0, 5.5 Hz, 1H), 2.30-2.21 (m, 1H), 1.08 (s, 21H); ^{13}C (101 MHz, $CDCl_3$) δ = 153.2, 141.7, 134.4, 117.5, 110.4, 107.3, 74.7, 71.3, 37.5, 18.3, 18.2, 12.8; IR (ATR): ṽ = 3458, 2943, 2866, 1640, 1463, 1385, 1245, 1147, 1104, 1065, 1000, 916, 881, 803, 731, 676; LRMS *m*/*z* (ESI$^+$) [M+Na]$^+$ 347; HRMS (ESI$^+$, *m*/*z*) calculated for $[C_{18}H_{32}O_3SiNa]^+$ 347.2019, found 347.2013.

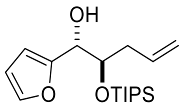

The ee of **18m** was determined by 2D HPLC analysis

Separation of diastereomers: 100 m RX-SiL, ∅ 4.6 mm, *n*-heptane/MTBE 97:3, v = 1.0 mL/min, λ = 220 nm, t(major) = 4.03 min, 308 K. (Note: minor diastereomer not identified for either sample)

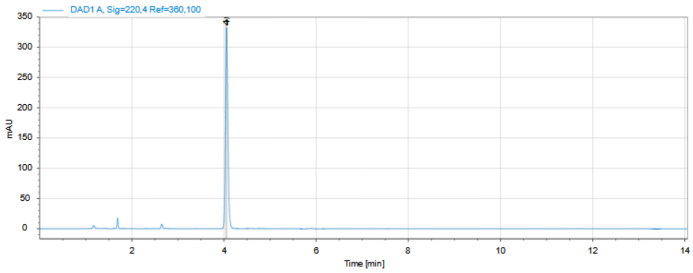

Sampling table (^{1}D)

Cut group	Cut #	^{1}D Cut start [min]	^{1}D Ret. time [min]	^{1}D Duration [min]	Trigger	^{2}D Run start [min]
	1	4.03	4.056	0.04	Peak	4.08

18m: separation of diastereomers on achiral column

^{1}D chromatogram(s)

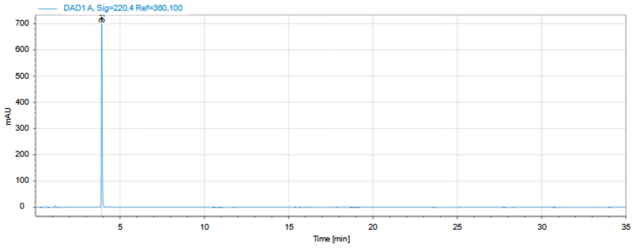

Sampling table (^{1}D)

Cut group	Cut #	^{1}D Cut start [min]	^{1}D Ret. time [min]	^{1}D Duration [min]	Trigger	^{2}D Run start [min]
	1	3.88	3.920	0.04	Peak	3.94

rac-**18m:** separation of diastereomers on achiral column

Chiral separation of major diastereomer: 150 mm Chiralcel OD-3R, ∅ 4.6 mm, *n*-heptane/MTBE = 95:5, v = 1.0 mL/min, λ = 220 nm, t(major) = 5.33 min, t(minor) = 6.05 min. 298 K

Cut# : 1

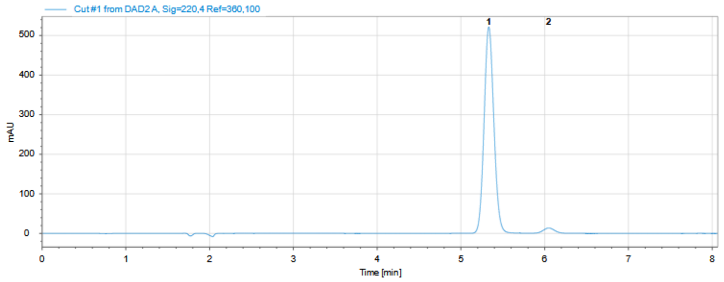

Signal: DAD2 A, Sig=220,4 Ref=360,100

Compound	Cut	Ret.Time	Area	Width	Height	Symmetry
1	1	5.333	4225.224<br>**4225.224**	0.135	521.356	0.894
2	1	6.049	123.017<br>**123.017**	0.153	13.382	0.946

Component	^{1}D Sampling range [min]	Ret.Time ^{2}D [min]	Area	Area%	
1	4.03 - 4.07	5.333	4225.224	97.171	1. enantiomer
2	4.03 - 4.07	6.049	123.017	2.829	2. enantiomer

ee = 94.3 %

18m: ee determination of pure major diastereomer

Cut# : 1

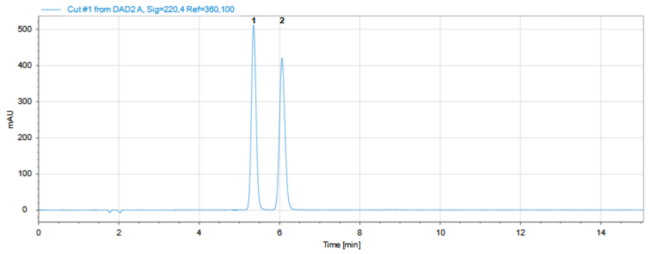

Signal: DAD2 A, Sig=220,4 Ref=360,100

Compound	Cut	Ret.Time	Area	Width	Height	Symmetry
1	1	5.354	4143.951<br>**4143.951**	0.127	510.341	0.889
2	1	6.062	3995.290<br>**3995.290**	0.159	418.970	0.866

rac-**18m**: separation of enantiomers

syn-2-((_tert_-Butyldimethylsilyl)oxy)-1-cyclohexylpent-4-en-1-ol 19a. Prepared according to representative procedure E (-20 °C). Purified by flash chromatography (SiO_2, hexane/ethyl acetate 99:1 to 98:2) to give diol **19a** as a colourless oil (55 mg, 63% yield, 20:1 dr, 90% ee). $[\alpha]_D^{20}$ = 10.5 (c = 0.55, $CHCl_3$); ^{1}H NMR (400 MHz, $CDCl_3$) δ = 5.67 (ddt, _J_ = 17.5, 10.0, 7.0 Hz, 1H), 5.03-4.94 (m, 2H), 3.74-3.69 (m, 1H), 3.01 (dd, _J_ = 7.5, 2.5 Hz, 1H), 2.41-2.32 (m, 1H), 2.17-2.09 (m, 1H), 1.96 (br. s, 1H), 1.90-1.83 (m, 1H), 1.71-1.61 (m, 2H), 1.59-1.48 (m, 2H), 1.32-1.22 (m, 1H), 1.18-1.03 (m, 3H), 1.00-0.87 (m, 2H), 0.82 (s, 9H), 0.02 (s, 3H), 0.00 (s, 3H); ^{13}C NMR (101 MHz, $CDCl_3$) δ = 134.3, 117.5, 76.1, 71.3, 40.3, 39.1, 29.7, 28.7, 26.5, 26.2, 26.1, 25.9, 18.1, -3.9, -4.7; IR (ATR): ṽ = 3562, 2926, 2854, 1641, 1449, 1390, 1361, 1253, 1102, 1049, 910, 834, 774, 678; LRMS m/z (EI^+) $[M]^+$ 298; HRMS (ESI^-, m/z) calculated for $[C_{17}H_{33}O_2Si]^-$ 297.22564, found 297.22553. The shown absolute configuration was assigned by analogy (see above).

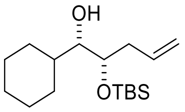

The ee of **19a** was determined by chiral GC

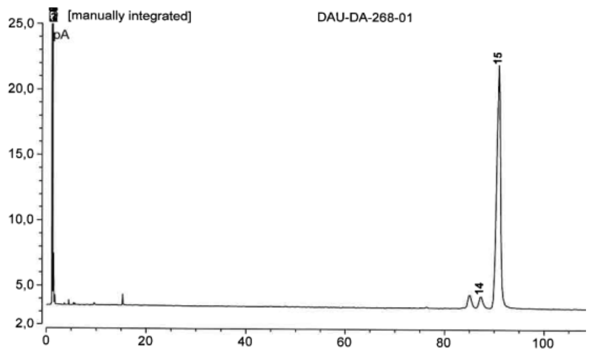

Sample: DAU-DA-268-01
Sequenz: 8091 DAU-DA Kes
Sequenz date: 04.03.21
Instrument: GC_411
Measured: 04.03.21 15:50
Processing M.: ee DAU-DA
Report-File: ee 268-01

chirale Messung, Verhältnis der Enantiomere
Zuordnung siehe Racemat DAU-DA-217-01 20/7826

No.	Ret.Time min	Rel.Area %	Peak Name
14	87,26	5,24	.
15	90,96	94,76	.

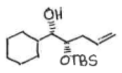

Instrument parameters:
Column: 24,5 m IVADEX-1 0,25/0,25df G/662
Temperature: 220/ 115 iso/ 350 Split 80
Gas: 0,50 bar Hydrogen
Sample size: 1,0 µL

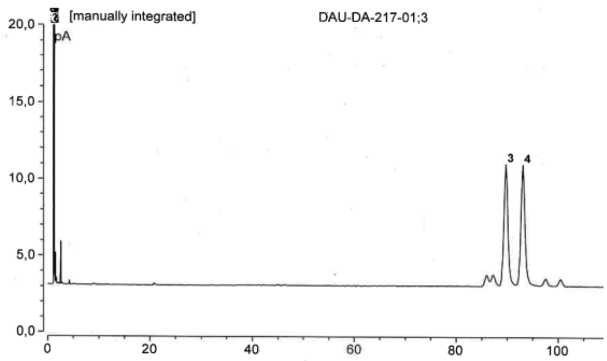

Sample: DAU-DA-217-01;3
Sequenz: 7826 DAU-DA VD
Sequenz date: 20.11.20
Instrument: GC_411
Measured: 24.11.20 13:58
Processing M.: ee DAU-DA
Report-File: ee 217-01

chirale Mesung des Racemates

No.	Ret.Time min	Rel.Area %	Peak Name
3	89,80	49,53	.
4	93,09	50,47	.

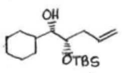

Instrument parameters:
Column: 24,5 m IVADEX-1 0,25/0,25df G/662
Temperature: 220/ 115 iso/ 350
Gas: 0,50 bar Hydrogen
Sample size: 0,2 µL

***syn*-1-Cyclohexyl-2-((triisopropylsilyl)oxy)pent-4-en-1-ol 19b.** Prepared according to representative procedure E (-20 °C). Purified by flash chromatography (SiO_2, hexane/ethyl acetate 99:1) to give diol **19b** as a colourless oil (55 mg, 36% yield, 14:1 dr, 93% ee). $[\alpha]_D^{20}$ = 10.9 (c = 0.47, $CHCl_3$); ^1H NMR (400 MHz, $CDCl_3$) δ = 5.75 (ddt, *J* = 17.0, 10.0, 7.0 Hz, 1H), 5.12-5.04 (m, 2H), 3.96 (dt, *J* = 9.0, 3.0 Hz, 1H), 3.12 (dd, *J* = 7.5, 3.0 Hz, 1H), 2.54 (app. dddt, *J* = 14.0, 8.4, 7.5, 1.0 Hz, 1H), 2.27 (app. dddd, *J* = 13.5, 6.5, 3.5, 1.5 Hz, 1H), 2.16 (br. s, 1H), 1.97-1.89 (m, 1H), 1.80-1.58 (m, 5H), 1.49-1.38 (m, 1H), 1.23-1.14 (m, 3H), 1.10-1.07 (m, 21H); ^{13}C NMR (101 MHz, $CDCl_3$) δ = 134.2, 117.8, 75.9, 71.8, 40.3, 39.1, 30.1, 28.7, 26.7, 26.3(2), 26.3(0), 18.4, 18.3, 13.0; IR (ATR): ṽ = 3550, 2924, 2866, 1705, 1641, 1463, 1450, 1386, 1249, 1107, 1058, 995, 916, 881, 677; LRMS *m*/*z* (ESI$^+$) [M+Na]$^+$ 363; HRMS (ESI$^+$, *m*/*z*) calculated for $[C_{20}H_{40}O_2SiNa]^+$ 363.2690, found 363.2688. The shown absolute configuration was assigned by analogy (see above).

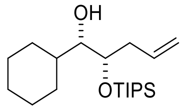

The ee of **19b** was determined by chiral GC

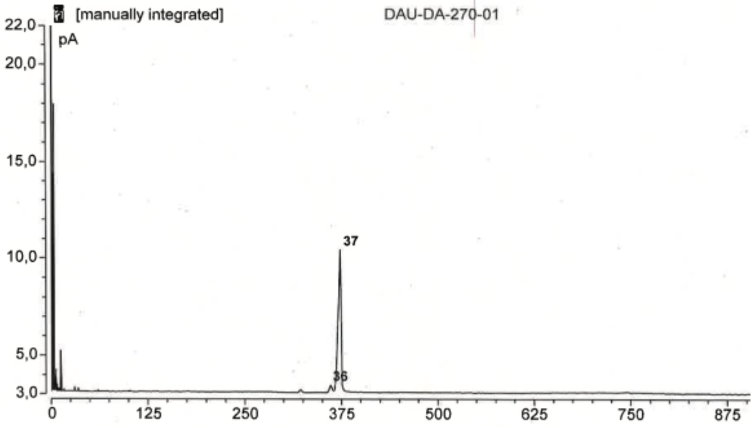

Sample:	**DAU-DA-270-01**	Instrument:	**GC_411**
Sequenz:	**8093 DAU-DA SE**	Measured:	**08.03.21 14:16**
Sequenz date:	**05.03.21**	Processing M.:	DAU-DA
		Report-File:	Verhältnis 270-01

Verhältnis der Enantiomere
Zuordnung achiral nach Racemat GCMS 30077 DAU-DA-253-01 21/8093

No.	Ret.Time min	Rel.Area %	Peak Name
36	360,59	3,85	.
37	373,69	96,15	.

Suggestion!

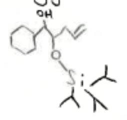

Instrument parameters:

Column:	24,5 m	IVADEX-1 0,25/0,25df G/662
Temperature:	220/120 iso/350	
Gas:	0,50 bar	Hydrogen
Sample size:	0,2 µL	

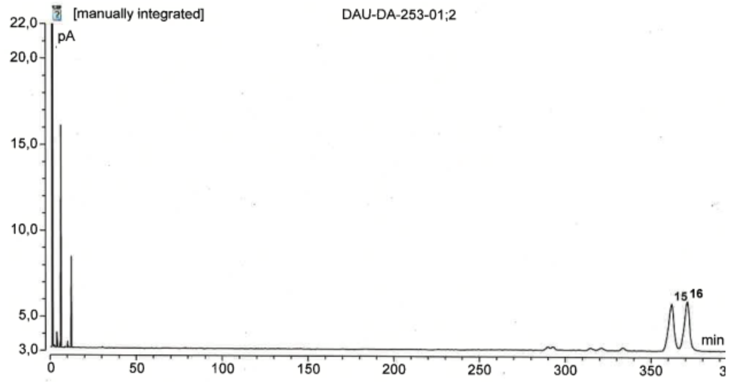

Sample:	**DAU-DA-253-01;2**	Instrument:	**GC_411**
Sequenz:	**8093 DAU-DA SE**	Measured:	**08.03.21 07:19**
Sequenz date:	**05.03.21**	Processing M.:	DAU-DA
		Report-File:	Verhältnis 253-01

Racemat
Verhältnis der Enantiomere
Zuordnung achiral nach GCMS 30077 DAU-DA-253-01 21/8093

No.	Ret.Time min	Rel.Area %	Peak Name
15	362,04	49,06	.
16	371,08	50,94	.

Suggestion!

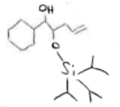

Instrument parameters:

Column:	24,5 m	IVADEX-1 0,25/0,25df G/662
Temperature:	220/120 iso/350	
Gas:	0,50 bar	Hydrogen
Sample size:	0,2 µL	

***syn*-4-((*tert*-Butyldimethylsilyl)oxy)-1-phenylhept-6-en-3-ol 11a.** Prepared according to representative procedure E (-20 °C). Purified by flash chromatography (SiO_2, hexane/ethyl acetate 97:3) to give diol **11a** as a colourless oil (69 mg, 74% yield, 11:1 dr, 90% ee). $[\alpha]^{20}_{D}$ = -4.6 (c = 0.52, $CHCl_3$); ^{1}H NMR (400 MHz, $CDCl_3$) δ 7.33–7.25 (m, 2H), 7.20 (dt, *J* = 8.1, 2.1 Hz, 3H), 5.77 (ddt, *J* = 17.3, 10.2, 7.2 Hz, 1H), 5.11–4.99 (m, 2H), 3.59 (ddd, *J* = 7.0, 4.7, 3.8 Hz, 1H), 3.49 (tdd, *J* = 8.4, 5.8, 4.2 Hz, 1H), 2.85 (ddd, *J* = 13.8, 8.8, 6.5 Hz, 1H), 2.74–2.60 (m, 1H), 2.48–2.37 (m, 1H), 2.27–2.13 (m, 2H), 1.81–1.70 (m, 2H), 0.91 (s, 8H), 0.10 (s, 3H), 0.08 (s, 3H). ^{13}C NMR (101 MHz, $CDCl_3$) δ 142.2, 134.2, 128.4, 128.3, 125.7, 117.6, 74.7, 71.9, 38.6, 35.7, 32.2, 25.9, 18.1, –4.1, – 4.6 ppm. IR (ATR): ṽ = 3461, 2929, 2857, 1641, 1604, 1496, 1471, 1390, 1361, 1254, 1074, 1004, 912, 833, 810, 774, 746, 698, 678. MS (EI) m/z (%): 263 (12), 245 (4), 207 (4), 185 (100), 171 (39), 129 (64), 91 (65), 75 (79). HRMS (ESI$^+$) m/z calcd. for $C_{19}H_{32}O_2SiNa$: 343.2063, found: 343.2065. The shown absolute configuration was assigned by analogy (see above).

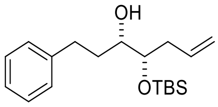

The ee of **11a** was determined by 2D HPLC analysis. Please note, in this case the *syn* diastereomer, which was the major diastereomer in the enantioselective reaction, was the minor (2nd) diastereomer of the racemic sample.

Separation of diastereomers: 50 mm Zorbax Eclipse Plus C18, ∅ 4.6 mm, acetonitrile/water 75:25, v = 1.0 mL/min, λ = 220 nm, t(major) = 5.37 min, t(minor) = 5.64 min, 308 K.

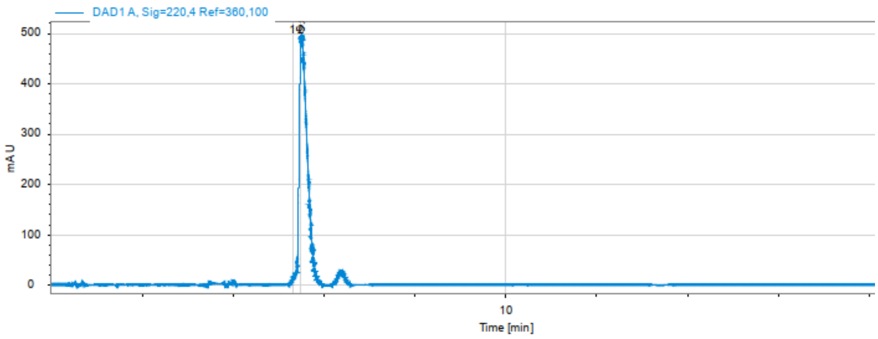

Sampling table (¹D)

Cut group	Cut #	¹D Cut start [min]	¹D Ret. time [min]	¹D Duration [min]	Trigger	²D Run start [min]	
	1	5.29	***	0.04	Time	5.33	1st Diastereomer
	2	5.47	5.508	0.04	Time	32.18	2nd Diastereomer

11a: separation of diastereomers on achiral column

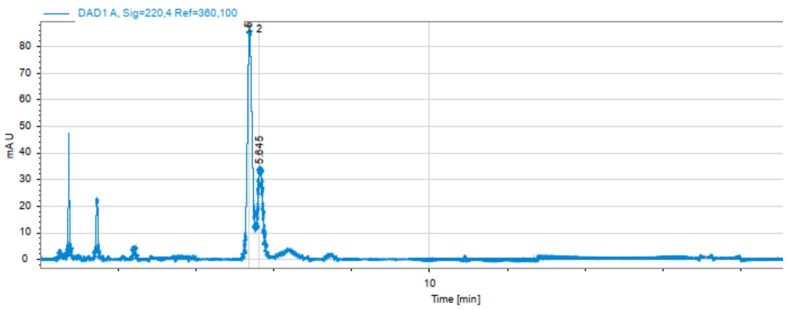

Sampling table (¹D)

Cut group	Cut #	¹D Cut start [min]	¹D Ret. time [min]	¹D Duration [min]	Trigger	²D Run start [min]	
	1	5.35	5.372	0.04	Time	5.39	1st Diastereomer
	2	5.61	5.645	0.04	Time	32.24	2nd Diastereomer

rac-**11a:** separation of diastereomers on achiral column

Chiral separation of *syn* diastereomer: 150 mm Chiralpak IB-N3, ∅ 4.6 mm, acetonitrile/water = 55:45, v = 1.0 mL/min, λ = 220 nm, t(minor) = 16.71 min, t(major) = 17.60 min. 298 K

Cut# : 2 2nd Diastereomer

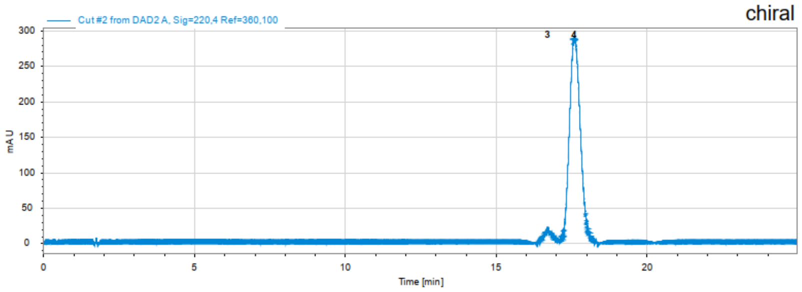

Signal: DAD2 A, Sig=220,4 Ref=360,100

Compound	Cut	Ret.Time	Area	Width	Height	Symmetry	
3	2	16.713	409.537 **409.537**	0.360	17.410	0.926	1st Enantiomer
4	2	17.597	7421.021 **7421.021**	0.401	289.337	0.853	2nd Enantiomer

ee = 89.5 %

11a: ee determination of pure *syn* diastereomer

Cut# : 2 2nd Diastereomer

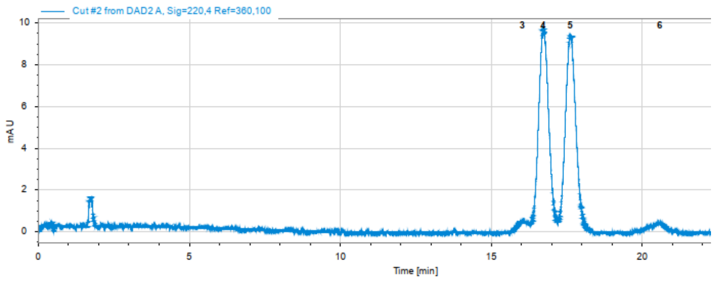

Signal: DAD2 A, Sig=220,4 Ref=360,100

Compound	Cut	Ret.Time	Area	Width	Height	Symmetry	
3	2	16.047	13.013 **13.013**	0.409	0.531	0.991	
4	2	16.734	233.371 **233.371**	0.399	9.744	0.931	1st Enantiomer
5	2	17.624	232.800 **232.800**	0.384	9.432	0.902	2nd Enantiomer
6	2	20.599	19.235 **19.235**	0.659	0.486	0.693	

rac-**11a**: separation of enantiomers of *syn* diastereomer

***syn*-4-((*tert*-Butyldimethylsilyl)oxy)-2-methylhept-6-en-3-ol 19c.** Prepared according to representative procedure E (-20 °C). Purified by flash chromatography (SiO_2, hexane/ethyl acetate 98.5:1.5) to give diol **19c** as a colourless oil (42 mg, 56% yield, 20:1 dr, 91% ee). Note: compound is likely volatile, should not be left under high vacuum. $[\alpha]_D^{20}$ = 21.0 (c = 0.49, $CHCl_3$); ^{1}H NMR (400 MHz, $CDCl_3$) δ = 5.77 (ddt, *J* = 17.5, 10.0, 7.0 Hz, 1H), 5.12-5.04 (m, 2H), 3.77 (ddd, *J* = 7.5, 4.5, 3.0 Hz, 1H), 3.05 (dd, *J* = 7.5, 3.0 Hz, 1H), 2.48-2.39 (m, 1H), 2.22 (app. dddt, *J* = 14.0, 7.0, 4.5, 1.5 Hz, 1H), 2.07 (br. s, 1H), 1.71-1.60 (m, 1H), 0.97 (d, *J* = 6.5 Hz, 3H), 0.91-0.89 (m, 9H), 0.88 (d, *J* = 6.5 Hz, 3H), 0.11 (s, 3H), 0.09 (s, 3H); ^{13}C NMR (101 MHz, $CDCl_3$) δ = 134.4, 117.7, 77.2, 72.0, 39.3, 30.6, 26.0, 19.6, 18.4,, 18.2, -3.8, -4.5; IR (ATR): ṽ = 3561, 2956, 2930, 2858, 1641, 1741, 1389, 1362, 1254, 1055, 1005, 913, 866, 834, 774, 679; LRMS m/z (EI$^+$) [M]$^+$ 258; HRMS (ESI$^+$, *m/z*) calculated for $[C_{14}H_{30}O_2SiNa]^+$ 281.1907, found 281.1903. The shown absolute configuration was assigned by analogy (see above).

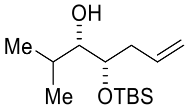

The ee of **19c** was determined by chiral GC

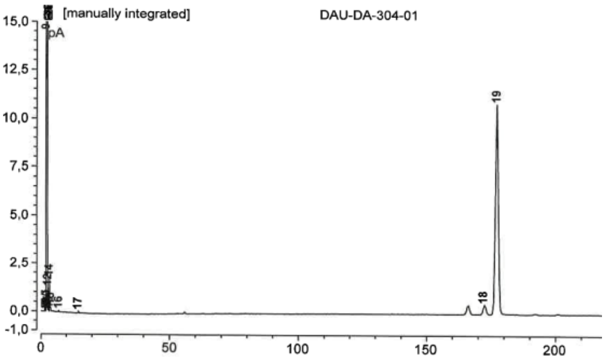

Sample:	**DAU-DA-304-01**		Instrument:	**GC_313**
Sequenz:	**8190 DAU-DA MM**		Measured:	**16.04.21 08:41**
Sequenz date:	**14.04.21**		Processing M.:	DAU 305
			Report-File:	Verhältnis

Enantiomerenverhältnis
Zuordnung nach achiral Messung

No.	Ret.Time min	Rel.Area %	Peak Name
18	172,45	4,41	.
19	177,39	95,59	.

Instrument parameters:

Column:	30,0 m	BGB-176/BGB-15 0,25/0,25df G/618
Temperature:	220 / 70, 225 min iso 8/min 240, 3 min iso / 350	
Gas:	0,60 bar	H2
Sample size:	0,2 µL	

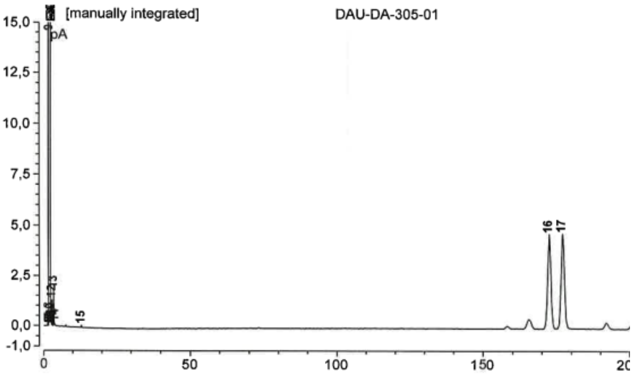

Sample:	**DAU-DA-305-01**		Instrument:	**GC_313**
Sequenz:	**8190 DAU-DA MM**		Measured:	**15.04.21 11:04**
Sequenz date:	**14.04.21**		Processing M.:	DAU 305
			Report-File:	Verhältnis

Razemat
Zuordnung nach achiral Messung

No.	Ret.Time min	Rel.Area %	Peak Name
16	172,70	49,32	.
17	177,22	50,68	.

Instrument parameters:

Column:	30,0 m	BGB-176/BGB-15 0,25/0,25df G/618
Temperature:	220/ 70 iso/ 350	
Gas:	0,60 bar	H2
Sample size:	0,2 µL	

***syn*-3-((*tert*-Butyldimethylsilyl)oxy)-1-phenylhex-5-en-2-ol 19d.** Prepared according to

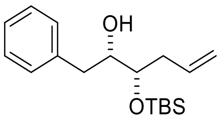

representative procedure E (-20 °C). Purified by flash chromatography (SiO_2, hexane/ethyl acetate 98:2) to give diol **19d** as a colourless oil (40 mg, 45% yield, 20:1 dr, 88% ee). $[\alpha]_D^{20}$ = -3.1 (c = 0.55, $CHCl_3$); ^1H NMR (400 MHz, $CDCl_3$) δ = 7.32-7.27 (m, 2H), 7.24-7.20 (m, 3H), 5.78 (ddt, *J* = 17.5, 10.5, 7.0 Hz, 1H), 5.12-5.04 (m, 2H), 3.76 (ddd, *J* = 8.5, 4.5, 3.5 Hz, 1H), 3.67 (ddd, *J* = *J* = 7.0, 5.0, 3.5 Hz, 1H), 2.80 (dd, *J* = 14.0, 4.5 Hz, 1H), 2.71 (dd, *J* = 14.0, 8.5 Hz, 1H), 2.47 (app. dtt, *J* = 14.0, 7.0, 1.5 Hz, 1H), 2.25 (app. dddt, *J* = 14.0, 7.5, 5.0, 1.5 Hz, 1H), 0.94 (s, 9H), 0.12 (s, 3H), 0.11 (s, 3H); ^{13}C NMR (101 MHz, $CDCl_3$) δ = 139.1, 134.3, 129.4, 128.5, 126.4, 117.7, 74.1, 73.7, 40.3, 38.6, 26.0, 18.3, -3.9, -4.4; IR (ATR): ṽ = 3560, 3028, 2953, 2929, 2857, 1641, 1471, 1389, 1361, 1253, 1076, 1004, 909, 832, 774, 74, 698; LRMS m/z (EI^+) $[M]^+$ 306; HRMS (ESI^+, m/z) calculated for $[C_{18}H_{30}O_2SiNa]^+$ 329.1907, found 329.1910. The absolute configuration was assigned after desilylation by comparison of the resulting diol with literature data (see above).

The ee of **19d** was determined by 2D HPLC analysis

Separation of diastereomers: 50 mm Eclipse PAH, ∅ 4.6 mm, acetonitrile/water 60:40, v = 1.0 mL/min, λ = 220 nm, t(minor) = 9.99 min, t(major) = 10.36 min, 308 K.

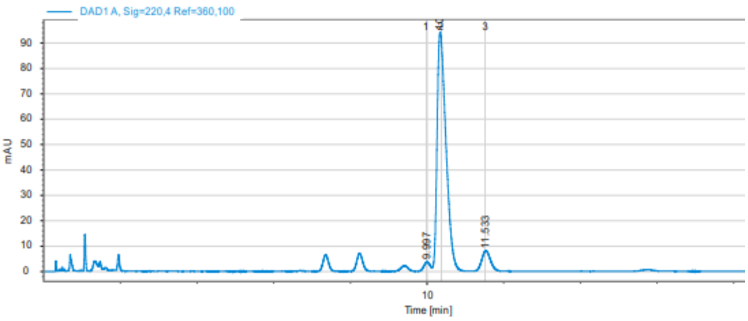

Sampling table (^1D)

Cut group	Cut #	^1D Cut start [min]	^1D Ret. time [min]	^1D Duration [min]	Trigger	^2D Run start [min]	
	1	9.96	9.997	0.04	Time	10.01	1st diastereomer
	2	10.34	10.341	0.04	Time	61.86	2nd diastereomer
	3	11.50	11.533	0.04	Time	36.86	isomer

19d: separation of diastereomers on achiral column

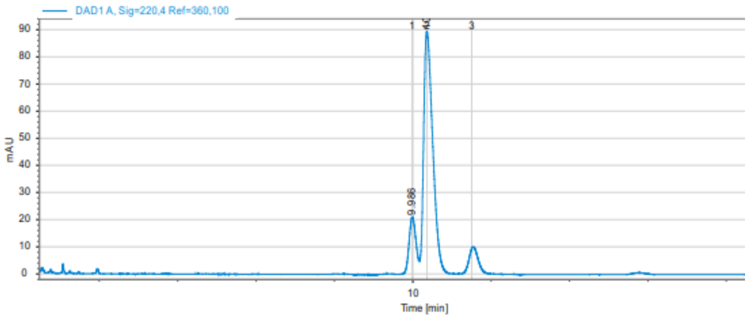

Sampling table (¹D)

Cut group	Cut #	¹D Cut start [min]	¹D Ret. time [min]	¹D Duration [min]	Trigger	²D Run start [min]	
	1	9.96	9.986	0.04	Time	10.01	1st diastereomer
	2	10.34	10.358	0.04	Time	61.86	2nd diastereomer
	3	11.50	***	0.04	Time	36.86	isomer

rac-**19d:** separation of diastereomers on achiral column

Chiral separation of major diastereomer: 150 mm Chiralpak OJ-3R, ∅ 4.6 mm, acetonitrile/water = 50:50, v = 1.0 mL/min, λ = 220 nm, t(major) = 18.73 min, t(minor) = 22.81 min. 298 K

Cut# : 2 2nd diastereomer

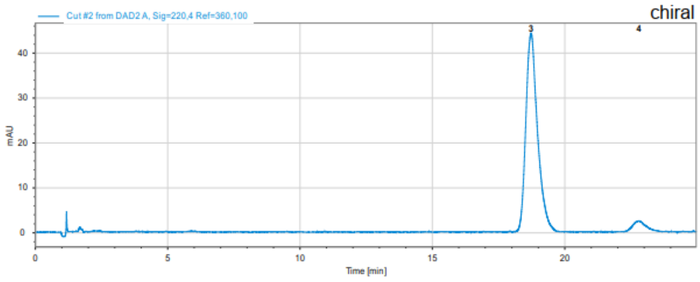

Signal: DAD2 A, Sig=220,4 Ref=360,100

Compound	Cut	Ret.Time	Area	Width	Height	Symmetry		
3	2	18.726	1372.961<br>**1372.961**	0.515	44.425	0.761	1st enantiomer	ee = 88.1 %
4	2	22.806	86.775<br>**86.775**	0.586	2.468	0.926	2nd enantiomer	

19d: ee determination of pure major diastereomer

Cut# : 2 2nd diastereomer

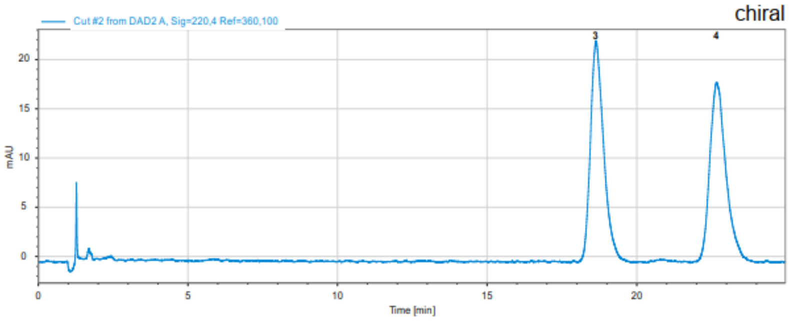

Signal: DAD2 A, Sig=220,4 Ref=360,100

Compound	Cut	Ret.Time	Area	Width	Height	Symmetry	
3	2	18.637	689.760<br>**689.760**	0.511	22.507	0.717	1st enantiomer
4	2	22.667	684.341<br>**684.341**	0.625	18.239	0.700	2nd enantiomer

rac-**19d**: separation of enantiomers

***syn*-1-([1,1'-Biphenyl]-4-yl)-2-((triisopropylsilyl)oxy)pent-4-en-1-ol 19e.** Prepared

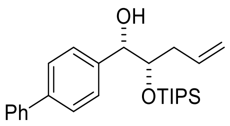

according to representative procedure E (-20 °C). Purified by flash chromatography (SiO_2, hexane/ethyl acetate 98:2) to give diol **19a** as a colourless oil (107 mg, 90% yield, 18:1 dr, 74% ee). $[\alpha]_D^{20}$ = -12.3 (c = 0.47, $CHCl_3$); ^1H NMR (400 MHz, C_6D_6) δ = 7.64-7.56 (m, 4H), 7.48-7.41 (m, 4H), 7.39-7.33 (m, 1H), 6.00-5.88 (m, 1H), 5.20-5.11 (m, 2H), 4.70 (d, *J* = 5.0 Hz, 1H), 4.08 (ddd, *J* = 8.0, 5.0, 3.5 Hz, 1H), 3.06 (br. s, 1H), 2.56 (dddt, *J* = 14.5, 8.0, 6.5, 1.5 Hz, 1H), 2.31-2.21 (m, 1H), 1.09-1.04 (m, 21H); ^{13}C NMR (101 MHz, $CDCl_3$) δ = 141.2, 140.5, 133.9, 128.9, 127.3, 127.2, 127.1, 127.0, 118.2, 77.1, 74.6, 38.5, 18.2, 18.1, 12.9; IR (ATR): ṽ = 3556, 3030, 2942, 2865, 1640, 1600, 1487, 1463, 1387, 1241, 1201, 1094, 1060, 997, 912, 882, 825, 762, 677, 510; LRMS *m*/*z* (ESI$^+$) $[M+Na]^+$ 433; HRMS (ESI$^+$, *m*/*z*) calculated for $[C_{26}H_{38}O_2SiNa]^+$ 433.2533, found 433.2534. The shown absolute configuration was assigned by analogy (see above).

The ee of **19e** was determined by 2D HPLC analysis

Separation of diastereomers: 50 mm Eclipse Plus C18, ∅ 4.6 mm, methanol/water 85:15, v = 1.0 mL/min, λ = 220 nm, t(minor) = 11.91 min, t(major) = 12.64 min, 308 K.

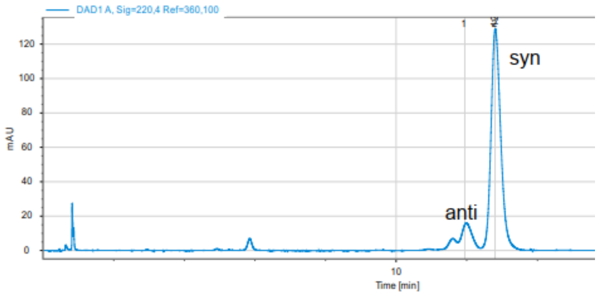

Sampling table (¹D)

Cut group	Cut #	¹D Cut start [min]	¹D Ret. time [min]	¹D Duration [min]	Trigger	²D Run start [min]
	1	11.91	***	0.04	Time	11.95
	2	12.77	12.808	0.04	Time	28.85

19e: separation of diastereomers on achiral column

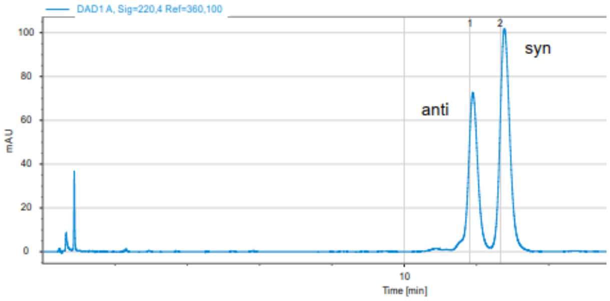

Sampling table (¹D)

Cut group	Cut #	¹D Cut start [min]	¹D Ret. time [min]	¹D Duration [min]	Trigger	²D Run start [min]
	1	11.81	***	0.04	Peak	11.85
	2	12.64	***	0.04	Peak	28.75

rac-**19e:** separation of diastereomers on achiral column

Chiral separation of major diastereomer: 150 mm Chiralcel IC-3, ∅ 4.6 mm, acetonitrile/water = 70:30, v = 1.0 mL/min, λ = 220 nm, t(major) = 10.19 min, t(minor) = 11.24 min. 298 K

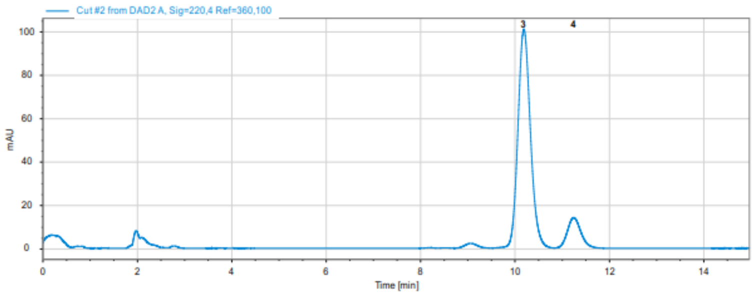

Signal: DAD2 A, Sig=220,4 Ref=360,100

Compound	Cut	Ret.Time	Area	Width	Height	Symmetry	
3	2	10.187	1854.377 **1854.377**	0.275	100.814	0.859	syn enantiomers
4	2	11.241	279.871 **279.871**	0.241	14.057	0.810	

Component	¹D Sampling range [min]	Ret.Time ²D [min]	Area	Area%	
1	11.91 - 11.95	8.413	111.014	4.692	anti
2	11.91 - 11.95	9.333	120.541	5.095	
3	12.77 - 12.81	10.187	1854.377	78.383	syn = 73.8 % ee
4	12.77 - 12.81	11.241	279.871	11.830	

19e: ee determination of pure major diastereomer

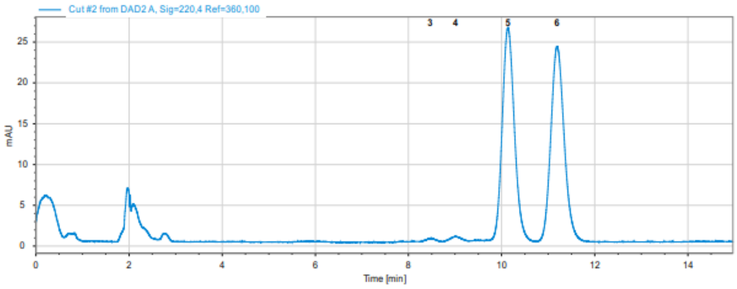

Signal: DAD2 A, Sig=220,4 Ref=360,100

Compound	Cut	Ret.Time	Area	Width	Height	Symmetry	
3	2	8.466	5.255 **5.255**	0.161	0.386	0.790	
4	2	9.011	9.419 **9.419**	0.194	0.570	0.928	
5	2	10.136	474.095 **474.095**	0.242	26.164	0.855	syn enantiomers
6	2	11.191	475.960 **475.960**	0.241	23.923	0.843	

rac-**19e**: separation of enantiomers

***syn*-1-([1,1'-Biphenyl]-4-yl)-2-(benzyloxy)pent-4-en-1-ol 19f.** Prepared according to representative procedure E (-40 °C). Purified by flash chromatography (SiO_2, hexane/ethyl acetate 95:5 to 92:8) to give diol **19f** as a light yellow oil (53 mg, 53% yield, 18:1 dr, 92% ee). $[\alpha]_D^{20}$ = 60.7 (c = 0.46, $CHCl_3$); ^1H NMR (400 MHz, $CDCl_3$) δ = 7.64-7.57 (m, 4H), 7.48-7.43 (m, 4H), 7.39-7.30 (m, 6H), 5.96-5.84 (m, 1H), 5.16-5.09 (m, 2H), 4.73-4.68 (m, 2H), 4.50 (d, *J* = 11.5 Hz, 1H), 3.71-3.65 (m, 1H), 2.90 (br. s, 1H), 2.45 (app. dddt, 14.8, 6.7, 5.3, 1.5 Hz, 1H), 2.24 (app. dddt, 14.5, 7.8, 5.5, 1.2 Hz, 1H); ^{13}C NMR (101 MHz, $CDCl_3$) δ = 141.0. 140.9, 140.1, 138.1, 134.1, 128.9, 128.6, 128.1, 128.0, 127.6, 127.4, 127.2, 118.0, 83.2, 75.3, 72.7, 34.9; IR (ATR): ṽ = 3436, 3030, 2871, 1640, 1600, 1486, 1453, 1405, 1270, 1205, 1064, 914, 839, 733, 695; LRMS *m*/*z* (EI^+) $[M]^+$ 344; HRMS (ESI^+, *m*/*z*) calculated for $[C_{24}H_{24}O_2Na]^+$ 367.1668, found 367.1671. The shown absolute configuration was assigned by analogy (see above).

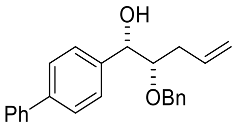

The ee of **19f** was determined by HPLC analysis: 150 mm Chiralpak IC-3, ∅ 4.6 mm, *n*-heptane/*i*-propanol = 90:10, v = 1.0 mL/min, λ = 220 nm, t(minor) = 4.59 min, t(major) = 5.01 min.

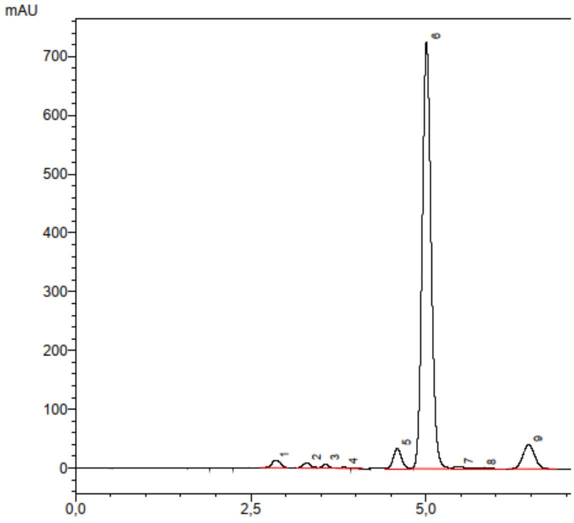

1 254nm,4nm

PDA Ch1 254nm

Peak #	Ret. Time	Area %	Name
1	2,84	1,49	
2	3,30	0,97	
3	3,57	0,63	
4	3,83	0,18	
5	4,59	3,77	1. Enantiomer
6	5,01	85,53	2. Enantiomer 91.6 % ee
7	5,47	0,55	
8	5,80	0,35	
9	6,47	6,53	
Total		100,00	

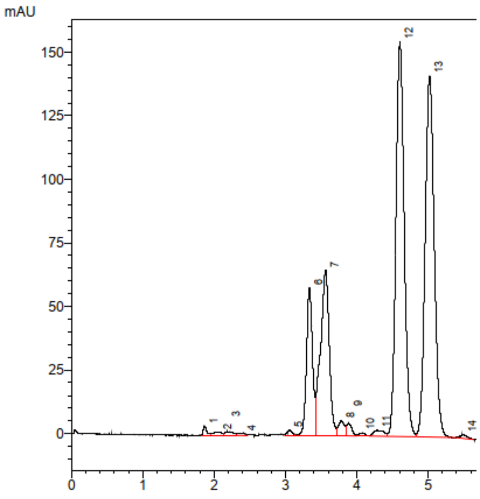

1 220nm,4nm

PDA Ch1 220nm

Peak #	Ret. Time	Area %	
1	1,86	0,36	
2	2,06	0,33	
3	2,18	0,38	
4	2,40	0,19	
5	3,05	0,33	
6	3,34	9,55	
7	3,56	15,71	
8	3,78	1,00	
9	3,89	0,74	
10	4,06	0,20	
11	4,29	0,65	
12	4,60	35,07	1. Enantiomer
13	5,02	35,00	2. Enantiomer
14	5,49	0,29	
15	6,47	0,22	
Total		100,00	

1.3.3 Synthesis of Dienyl Ethers

(E)-Configured dienes were prepared by reacting the appropriate aldehyde with a silyl chloride or triflate in the presence of triethylamine. (Z)-Configured dienes were made through a three-step sequence starting with methylation of commercially available cis-2-buten-1,4-diol using methyl iodide, substitution of the remaining hydroxy group, and (Z)-selective low temperature elimination of the methoxy group with n-BuLi. Both methods give the desired dienes as effectively a single isomer. These compounds are generally stable to silica gel chromatography and may be stored for months in the freezer under argon without decomposition or isomerisation as judged by ^{1}H NMR spectroscopy. However, they are acid-sensitive and for this reason either C_6D_6 or neutralised $CDCl_3$ were used to record NMR spectra.

General Procedure F for Preparation of (*E*)-DienylEthers. The aldehyde (1.0 or 1.7 equiv) and triethylamine (2.5 equiv) were dissolved in dichloromethane. To this solution was added the appropriate silyl triflate (1.0 or 1.1 equiv) dropwise at 0 °C, then the mixture was stirred at reflux temperature for 5 h. The mixture was diluted with ether and washed with cold sat. $NaHCO_3$ and brine. The organic phase was dried over $MgSO_4$, filtered and the solvent was removed *in vacuo*. The residue was purified by flash chromatography over silica gel (hexane 100%) to give the product.

(*E*)-Triisopropyl((3-methylbuta-1,3-dien-1-yl)oxy)silane 7. The desired product was

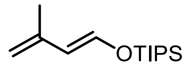

prepared according to the general procedure F, using 3-methylbut-2-enal (1.6 mL, 17.0 mmol, 1.7 equiv), triethylamine (3.5 mL, 25.0 mmol, 2.5 equiv) and TIPS triflate (2.7 mL, 10.0 mmol, 1.0 equiv) in dichloromethane (7.0 mL). The residue was purified by flash chromatography over silica gel (hexane 100%) to give **7** as colorless liquid (2.2 g, 92% yield). ^{1}H NMR (400 MHz, $CDCl_3$) δ 6.64 (br d, *J* = 12.0 Hz, 1H), 5.86 (dd, *J* = 12.0, 0.6 Hz, 1H), 4.76-4.72 (m, 1H), 4.68-4.65 (m, 1H), 1.80 (dd, *J* = 1.4, 0.7 Hz, 3H), 1.20-1.13 (m, 3H), 1.11-1.07 (m, 18H). HRMS (ESI) m/z calcd for $C_{14}H_{28}OSi$: 240.1901 $[M]^+$, found 240.1903. Matches known data.[15]

(*E*)-*tert*-Butyldimethyl((3-methylbuta-1,3-dien-1-yl)oxy)silane S9. The desired product was

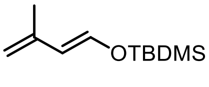

prepared according to the general procedure F, using 3-methylbut-2-enal (1.6 mL, 17.0 mmol, 1.7 equiv), triethylamine (3.5 mL, 25.0 mmol, 2.5 equiv) and TBDMS triflate (2.3 mL, 10.0 mmol, 1.0 equiv) in dichloromethane (7.0 mL). The residue was purified by flash chromatography over silica gel (hexane 100%) to give **S9** as colorless liquid (1.6 g, 81% yield). ^{1}H NMR (400 MHz, $CDCl_3$) δ 6.55 (br d, *J* = 12.2 Hz, 1H), 5.84 (br d, *J* = 12.2 Hz, 1H), 4.75-4.74 (m, 1H), 4.69-4.66 (m, 1H), 1.80 (dd, *J* = 1.4, 0.7 Hz, 3H), 0.93 (s, 9H), 0.16 (s, 6H). ^{13}C NMR (100 MHz, $CDCl_3$) δ 142.4, 139.9, 116.3, 111.9, 25.8, 19.2, 18.4, -5.1. HRMS (ESI) m/z calcd for $C_{11}H_{22}OSi$: 198.1432 $[M]^+$, found 198.1434. Matches known data.[16]

(*E*)-Triethyl((3-methylbuta-1,3-dien-1-yl)oxy)silane S10. The desired product was prepared

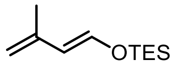

according to the general procedure F, using 3-methylbut-2-enal (1.4 mL, 14.1 mmol, 1.4 equiv), triethylamine (3.5 mL, 25.1 mmol, 2.5 equiv) and TES triflate (2.3 mL, 10.0 mmol, 1.0 equiv) in dichloromethane (7.0 mL). The residue was purified by flash chromatography over silica gel (hexane 100%) to give **S10** as colorless liquid (1.4 g, 99% yield). ^{1}H NMR (400 MHz, $CDCl_3$) δ 6.56 (d, *J* = 12.1 Hz, 1H), 5.84 (dd, *J* = 12.1, 0.7 Hz, 1H), 4.75-4.73 (m, 1H), 4.69-4.66 (m, 1H), 1.80 (dd, *J* = 1.4, 0.7 Hz, 3H), 1.02-0.96 (m, 9H), 0.73-0.66 (m, 6H). ^{13}C NMR (100 MHz, $CDCl_3$) δ 142.3, 140.0, 116.3, 111.9, 19.2, 6.2, 4.1. IR (ATR): ṽ = 3083, 3035, 2956, 2813, 2878, 1642, 1605, 1457, 1414, 1379, 1333, 1272, 1239, 1167, 1098, 1074, 1005, 974, 921, 867, 791, 765, 729, 680, 516 cm^{-1}. HRMS (ESI) m/z calcd for $C_{14}H_{28}OSi$: 240.1903 [M]$^+$, found 240.1903.

(*E*)-triisopropyl((2-methylbuta-1,3-dien-1-yl)oxy)silane S11. The desired product was

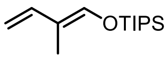

prepared according to the general procedure F, using 2-methylbut-2-enal (0.6 mL, 5.9 mmol, 1.0 equiv), triethylamine (1.7 mL, 11.9 mmol, 2.0 equiv) and TIPS triflate (1.8 mL, 6.5 mmol, 1.1 equiv) in dichloromethane (4.0 mL). The residue was purified by flash chromatography over silica gel (hexane 100%) to give **S11** as colorless liquid (1.1 g, 80% yield). ^{1}H NMR (400 MHz, $CDCl_3$) δ 6.52-6.50 (m, 1H), 6.32 (ddd, *J* = 17.2, 10.7, 0.6 Hz, 1H), 5.00 (ddd, *J* = 17.2, 1.5, 0.7 Hz, 1H), 4.83 (dd, *J* = 10.5, 1.4 Hz, 1H), 1.73 (d, *J* = 1.3 Hz, 3H), 1.23-1.13 (m, 3H), 1.10-1.06 (m, 18H). ^{13}C NMR (100 MHz, $CDCl_3$) δ 142.6, 137.3, 118.0, 107.9, 17.8, 12.0, 8.7. IR (ATR): ṽ = 2944, 2893, 2867, 1695, 1641, 1463, 1419, 1392, 1368, 1242, 1176, 1071, 1014, 995, 984, 919, 878, 829, 788, 684, 664, 592, 504, 463, 443 cm^{-1}. HRMS (ESI) m/z calcd for $C_{14}H_{28}OSi$: 240.1903 [M]$^+$, found 240.1903.

(*E*)-(Buta-1,3-dien-1-yloxy)triisopropylsilane S12. Prepared according to the general

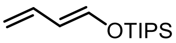

procedure F, using crotonaldehyde (1.4 mL, 17.0 mmol), triethylamine (3.5 mL, 25.0 mmol) and TIPS triflate (2.7 mL, 10.0 mmol) in dichloromethane (7.0 mL). The residue was purified by flash chromatography over silica gel (hexane 100%) to give **S12** as a colorless liquid (1.8 g, 80% yield). ^{1}H (400 MHz, $CDCl_3$) δ = 6.67 (dd, *J* = 11.8, 0.8 Hz, 1H), 6.28 (dddd, *J* = 16.9, 10.9, 10.2, 0.6 Hz, 1H), 5.78 (ddt, *J* = 11.8, 11.0, 0.8 Hz, 1H), 5.00 (ddt, *J* = 16.9, 1.7, 0.8 Hz, 1H), 4.82 (ddd, *J* = 10.3, 1.8, 0.8 Hz, 1H) 1.21-1.12 (m, 3H), 1.10-1.06 (m, 18H). ^{13}C (100 MHz, $CDCl_3$) δ = 145.9, 133.6, 114.1, 111.6, 17.8, 12.1. HRMS (ESI) m/z calcd for $C_{13}H_{26}OSi$: 226.1745 [M]$^+$, found 226.1747. Matches known data.[15]

(*E*)-(Buta-1,3-dien-1-yloxy)(tert-butyl)dimethylsilane S13. To a solution of crotonaldehyde

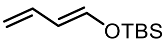

(5.5 mL, 66 mmol), triethylamine (10 mL, 72 mmol) and TBSCl (10 g, 66 mmol) in MeCN (10 ml) was added dropwise a solution of NaI (10.5 g, 70 mmol) in MeCN (50 ml). The reaction was heated to 50 °C for 16 hours and allowed to cool before being poured onto ice (150 g) and extracted with pentane (4 x 100 ml). The combined organic phases

were washed with brine solution and dried over Na_2SO_4, filtered and concentrated to give a pale yellow oil. The crude liquid was distilled under vacuum (20 mbar) discarding the forerun 68–75 !C collecting the between fraction 75–82 °C to give the product **S13** as a colorless liquid (7.9 g, 65%). ^{1}H NMR (400 MHz, $CDCl_3$) δ 6.57 (dd, J = 11.8, 0.7 Hz, 1H), 6.22 (dddd, J = 16.9, 10.9, 10.3, 0.7 Hz, 1H), 5.73 (ddt, J = 11.8, 10.9, 0.8 Hz, 1H), 5.04–4.93 (m, 1H), 4.81 (ddt, J = 10.3, 1.9, 0.7 Hz, 1H), 0.92 (s, 9H), 0.16 (s, 6H). ^{13}C NMR (101 MHz, $CDCl_3$) δ 145.5, 133.5, 114.3, 112.0, 25.7, 18.4, -5.1. Matches known data.[17]

(*E*)-(Buta-1,3-dien-1-yloxy)triethylsilane S14. The desired product was prepared according

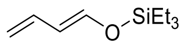

to general procedure # using crotonaldehyde (2.73 mL, 33.0 mmol), triethylamine (10.5 mL, 75.0 mmol), triethylsilyl triflate (6.78 mL, 30.0 mmol) and dichloromethane (21 mL) to give product **S14** as a colourless oil (4.09 g, 90% yield). ^{1}H (400 MHz, C_6D_6) δ = 6.57 (app. dq, J = 12.0, 0.5 Hz, 1H), 6.26 (dddd, J = 17.0, 11.0, 10.0, 0.5 Hz, 1H), 5.98 (app. ddt, J = 11.5, 11.0, 0.5 Hz, 1H), 5.03 (app. ddt, J = 17.0, 1.5, 0.5 Hz, 1H), 4.87 (app. ddt, J = 10.0, 1.5, 0.5 Hz, 1H), 0.91 (t, J = 8.0 Hz, 9H), 0.55 (q, J = 8.0 Hz, 6H). Matches known data.[18]

(*Z*)-4-Methoxybut-2-en-1-ol S15. To a suspension of NaH (900 mg, 37.7 mmol) in THF (120

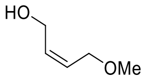

mL) at 0 °C was added *cis*-1,4-butendiol (10 mL, 121.7 mmol) dropwise. Once addition was complete the reaction was allowed to come to RT and stirred until H_2 evolution had ceased (30 minutes). At this point the beige suspension was again chilled to 0 °C and methyl iodide (1.8 mL, 28.9 mmol) was added in a single portion. After stirring for 10 mins at 0 °C the reaction was allowed to come to RT and stirred overnight. The reaction was then quenched with the addition of NH_4Cl solution and the organic phase was separated, and the aqueous phase was extracted with ethyl acetate (3 x 50 mL). The combined organic phases were washed with brine solution and dried over Na_2SO_4, filtered and concentrated to give a pale yellow oil. The residue was purified by flash chromatography (hexane/ethyl acetate 2:1) to give product **S15** as a colourless oil (2.38 g, 81%). ^{1}H NMR (400 MHz, $CDCl_3$) δ 5.77–5.68 (m, 1H), 5.59 (dtt, J = 11.2, 6.4, 1.4 Hz, 1H), 4.16–4.07 (m, 2H), 3.94 (ddd, J = 6.3, 1.6, 0.8 Hz, 2H), 3.28 (s, 3H), 3.08 (d, J = 8.6 Hz, 1H). ^{13}C NMR (101 MHz, $CDCl_3$) δ 132.4, 127.6, 68.0, 58.3, 58.0 ppm. Matches known data.[19]

(*Z*)-(*tert*-butyl)(dimethyl)(4-methoxybut-2-en-1-yl)oxy)silane S16. To a solution of alcohol

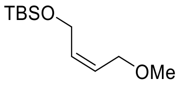

S15 (2.31 g, 22.59 mmol) in DMF (20 mL) at 0 °C was added imidazole (3 g, 44.1 mmol) followed by TBSCl (5.1 g, 33.8 mmol). The reaction was stirred with cooling for 10 minutes before being allowed to reach rt and being stirred overnight. The homogenous reaction mixture was then poured into NH_4Cl solution and extracted with ethyl acetate (3 x 100 mL). The combined organic extracts were washed twice with brine, dried and concentrated to a pale yellow oil. The residue was purified by flash

chromatography (hexane/*tert*-butyl methyl ether 95:5) to give the product as a colourless oil (4.45 g, 91%). ^{1}H NMR (400 MHz, $CDCl_3$) δ 5.74–5.64 (m, 1H), 5.57 (dtt, *J* = 11.3, 6.3, 1.6 Hz, 1H), 4.24 (ddt, *J* = 6.0, 1.9, 0.9 Hz, 2H), 3.98 (dd, *J* = 6.3, 1.3 Hz, 2H), 3.32 (s, 3H), 0.90 (s, 9H), 0.07 (s, 6H). ^{13}C NMR (101 MHz, $CDCl_3$) δ 132.7, 126.8, 68.2, 59.5, 58.0, 25.9, 18.3, -5.2 ppm. Matches known data.[20]

(*Z*)-(Buta-1,3-dien-1-yloxy)(*tert*-butyl)dimethylsilane S17. To a solution of TBS ether **S16** 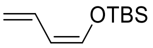

(4.15 g, 19.2 mmol) in diethyl ether (75 mL) at –30 °C was added *n*-BuLi (20 mL, 1.6 M in hexanes, 32 mmol) dropwise ensuring the internal temperature never exceeded –20 °C. The reaction was then stirred for two hours maintaining the temperature between –20 °C and –30 °C. The reaction was quenched with NH_4Cl solution and allowed to reach rt. The organic phase was separated, and the aqueous phase was extracted with pentane (3 x 50 mL). The combined organic phases were washed with brine solution and dried over Na_2SO_4, filtered and concentrated to give a pale yellow oil. The oil was distilled under vacuum, collecting all distillate between 65–70 °C at 17 mbar. Colourless liquid (2.65 g, 81%). ^{1}H NMR (400 MHz, $CDCl_3$) δ 6.76 (dtd, *J* = 17.3, 10.6, 1.0 Hz, 1H), 6.23–6.15 (m, 1H), 5.20 (dd, *J* = 10.8, 5.7 Hz, 1H), 5.07 (dd, *J* = 17.3, 2.2 Hz, 1H), 4.89 (d, *J* = 10.4 Hz, 1H), 0.95 (s, 9H), 0.16 (s, 6H). ^{13}C NMR (101 MHz, $CDCl_3$) δ 140.5, 129.9, 113.0, 111.2, 25.6, 18.3, –5.4 ppm. Matches known data.[20]

(*Z*)-Triisopropyl((4-methoxybut-2-en-1-yl)oxy)silane S18. Alcohol **S15** (1.0 g, 9.79 mmol) 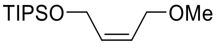

was dissolved in DMF (10 mL) in a flame-dried Schlenk flask. The solution was cooled to 0 °C and imidazole (1.33 g, 19.6 mmol) and TIPSCl (3.14 mL, 14.7 mmol) were added. The reaction was stirred at 0 °C for 10 mins, then allowed to warm to room temperature and stirred overnight. The reaction was quenched with sat. aq. NH_4Cl solution and extracted 3x with ethyl acetate. The combined organic layers were washed with brine (2 x), dried over Na_2SO_4, filtered and concentrated *in vacuo* to give a light yellow liquid. The crude product was purified by flash chromatography (SiO_2, hexane/ethyl acetate 98:2) to give the desired product **S18** as a colourless oil (2.30 g, 91% yield). ^{1}H (400 MHz, $CDCl_3$) δ = 5.72 (dtt, *J* = 11.5, 6.0, 1.5 Hz, 1H), 5.56 (dtt, *J* = 11.5, 6.5, 1.5 Hz, 1H), 4.31 (ddt, *J* = 7.0, 1.5, 1.0 Hz, 2H), 3.99 (ddt, *J* = 6.5, 1.5, 1.0 Hz, 3H), 3.32 (s, 3H), 1.09-1.05 (m, 21H); ^{13}C (101 MHz, $CDCl_3$) δ = 133.2, 126.6, 68.5, 59.9, 58.1, 18.1, 12.1; IR (ATR): ṽ = 2942, 2865, 1463, 1091, 1066, 995, 881, 803, 679, 657; HRMS (CI^+ (isobutane), m/z) calculated for $[C_{14}H_{31}O_2Si]^+$ 259.20855, found 259.20878. Matches known data.[20]

(*Z*)-(Buta-1,3-dien-1-yloxy)triisopropylsilane S19. Silyl ether **S18** (2.22 g, 8.59 mmol) was 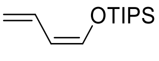

added to a flame-dried Schlenk flask under argon, dissolved in diethyl ether (32 mL) and cooled to -20 °C. *n*-BuLi (7.3 mL, 1.6 M in hexanes, 11.7 mmol) was added and the reaction mixture stirred at the same temperature for 2 h. The mixture was

quenched with sat. aq. NH_4Cl solution and extracted 3x with ethyl acetate. The combined organic extracts were washed with brine, dried over Na_2SO_4, filtered and evaporated under reduced pressure to give a colourless liquid. Purification by flash chromatography (SiO_2, hexane/ethyl acetate 98:2) to give the desired product as a colourless liquid (1.35 g, 70% yield). ^{1}H (400 MHz, C_6D_6) δ = 7.19-7.08 (m, 1H), 6.18 (app. ddt, *J* = 6.0, 2.0, 1.0 Hz, 1H), 5.26 (app. ddt, *J* = 11.0, 6.0, 1.0 Hz, 1H), 5.15 (app. ddt, *J* = 17.5, 2.0, 1.0 Hz), 4.98 (dddd, *J* = 10.5, 2.0, 1.5, 1.0 Hz), 1.04-1.00 (m, 21H); ^{13}C (101 MHz, C_6D_6) δ = 141.1, 130.4, 113.2, 111.7, 17.8, 12.2. Matches known data.[20]

(*Z*)-Triethyl((4-methoxybut-2-en-1-yl)oxy)silane S20. Alcohol **S15** (1.00 g, 9.79 mmol) was dissolved in DMF (10 mL) in a flame-dried Schlenk flask. The solution was cooled to 0 °C and imidazole (1.33 g, 19.6 mmol) and TESCl (2.47 mL, 14.7 mmol) were added. The reaction was stirred at 0 °C for 10 mins then allowed to warm to room temperature and stirred overnight. The reaction was quenched with water and extracted with ethyl acetate (3 x). The combined organic layers were washed with brine (2 x), dried over Na_2SO_4, filtered and concentrated *in vacuo* to give a colourless liquid. The crude product was purified by flash chromatography (SiO_2, hexane/ethyl acetate 98:2) to give the desired product as a colourless oil (1.88 g, 89% yield). ^{1}H (400 MHz, $CDCl_3$) δ = 5.70 (dtt, *J* = 11.0, 6.0, 1.5 Hz. 1H), 5.57 (dtt, *J* = 11.5, 6.5, 1.5 Hz, 1H), 4.23 (ddt, *J* = 6.0, 1.5, 1.0 Hz, 2H), 3.98 (ddt, *J* = 6.5, 1.5, 1.0 Hz, 3H), 3.32 (s, 3H), 0.96 (t, *J* = 8.0 Hz, 9H), 0.64-0.57 (m, 6H); ^{13}C (101 MHz, $CDCl_3$) δ = 132.8, 127.1, 68.3, 59.2, 58.1, 6.8, 4.6; IR (ATR): ṽ = 2955, 2876, 1459, 1413, 1293, 1190, 1080, 1004, 816, 724; HRMS (CI^+ (isobutane), m/z) calculated for $[C_{11}H_{25}O_2Si]^+$ 217.16154, found 217.16183. Matches known data.[20]

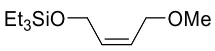

(*Z*)-(Buta-1,3-dien-1-yloxy)triethylsilane S21. Silyl ether **S20** (1.80 g, 8.32 mmol) was added to a flame-dried Schlenk flask under argon, dissolved in diethyl ether and cooled to -20 °C. *n*-BuLi (7.1 mL, 1.6 M in hexanes, 11.3 mmol) was added and the reaction mixture stirred at the same temperature for 2 h. The mixture was quenched with 10% aq. NH_4Cl solution and extracted 3x with ethyl acetate. The combined organic extracts were washed with brine, dried over Na_2SO_4, filtered and evaporated under reduced pressure to give a yellow oil. Purification by flash chromatography (SiO_2, hexane/TERT-BUTYL METHYL ETHER 99:1) to give the desired product **S21** as a colourless liquid (882 mg, 58% yield). ^{1}H (400 MHz, C_6D_6) δ = 7.16-7.06 (m, 1H), 6.11 (app. ddt, *J* = 6.0, 2.0, 1.0 Hz, 1H), 5.28 (app. ddt, *J* = 11.0, 6.0, 1.0 Hz, 1H), 5.15 (app. ddt, *J* = 17.5, 2.0, 1.0 Hz, 1H), 4.97 (dddd, *J* = 10.5, 2.0, 1.5, 1.0 Hz, 1H), 0.91 (t, *J* = 8.0 Hz, 9H), 0.53 (q, *J* = 8.0 Hz, 6H); ^{13}C (101 MHz, C_6D_6) δ = 140.5, 130.5, 113.3, 112.1, 6.7, 4.7. Matches known data.[20]

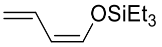

2. NMR Spectra of New or Previously Insufficiently Characterized Compounds

SI-2

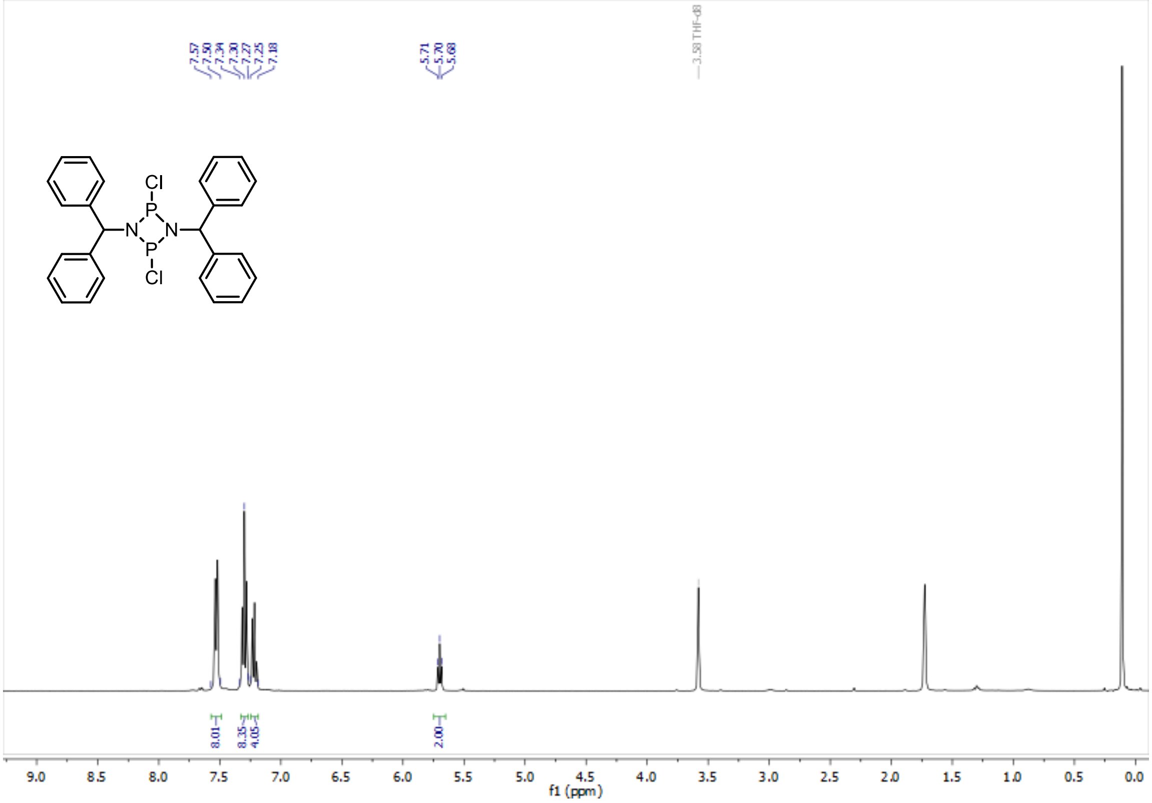

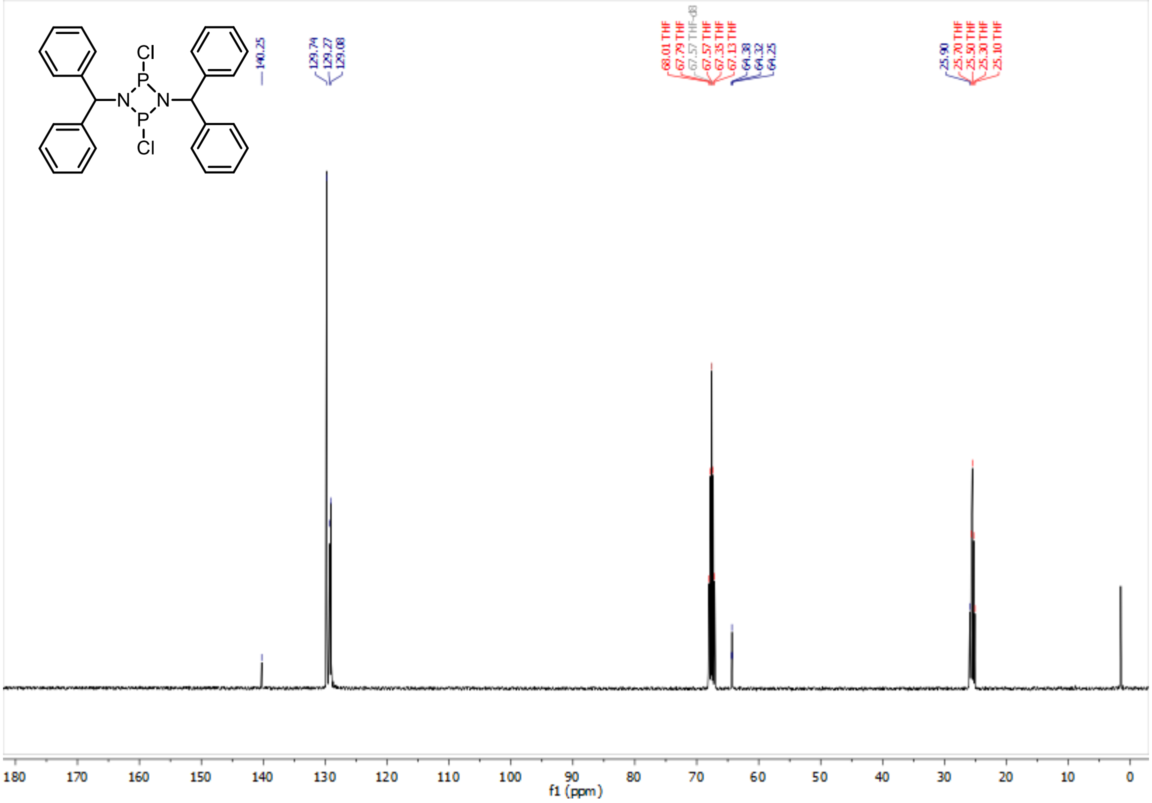

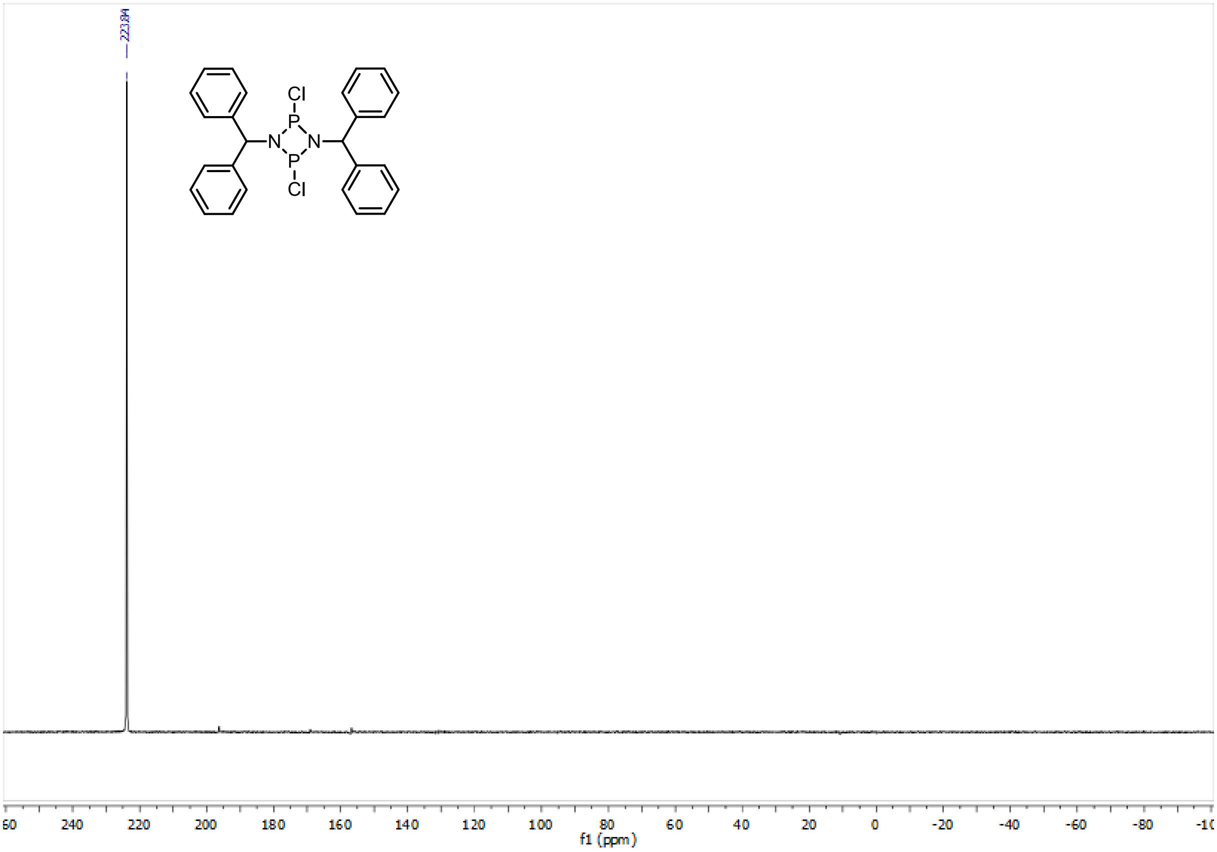
223.84
Cl
P
N
N
P
Cl
60
240
220
200
180
160
140
120
100
80
f1 (ppm)
60
40
20
0
-20
-40
-60
-80
-10

SI-3

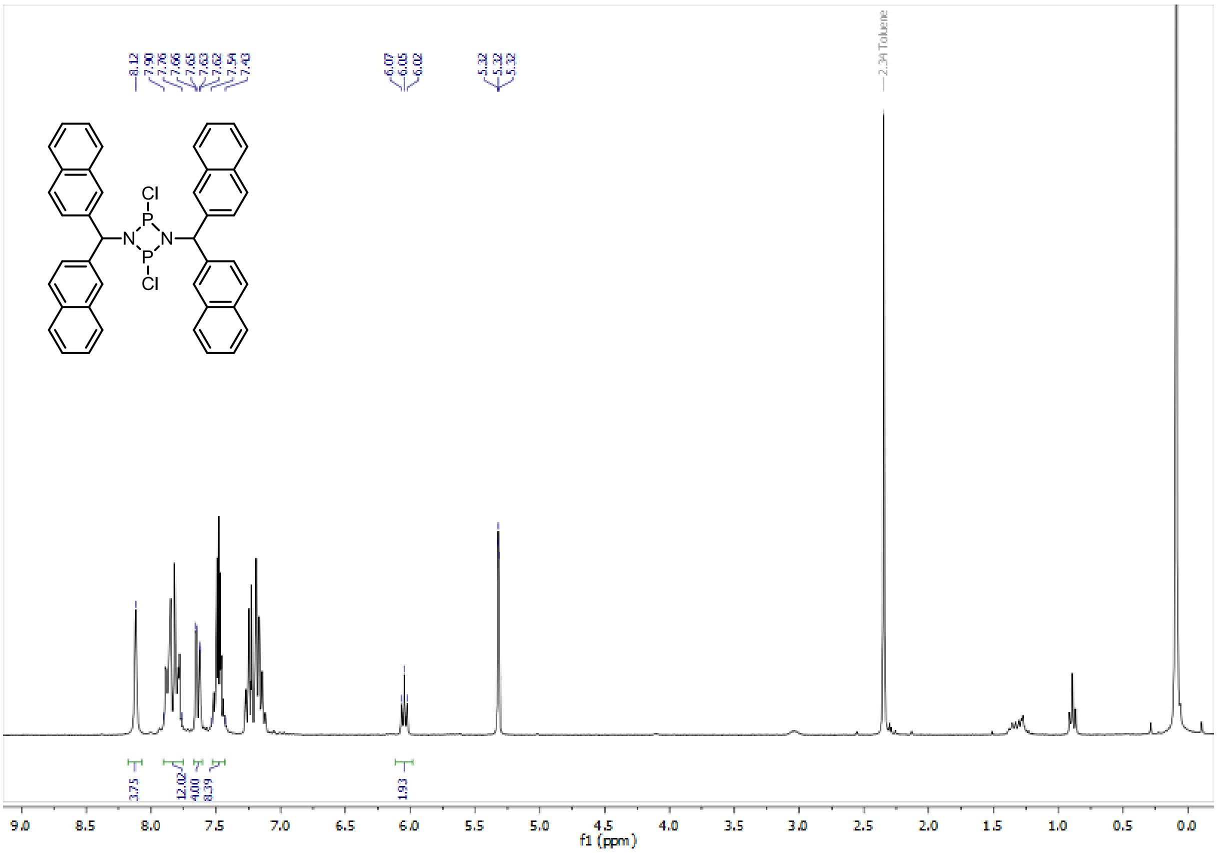

8.12
7.90
7.76
7.66
7.65
7.63
7.62
7.54
7.43
6.07
6.05
6.02
5.32
5.32
5.32
2.34 Toluene
Cl
P
N
N
P
Cl
3.75
12.02
4.00
8.39
1.93
f1 (ppm)

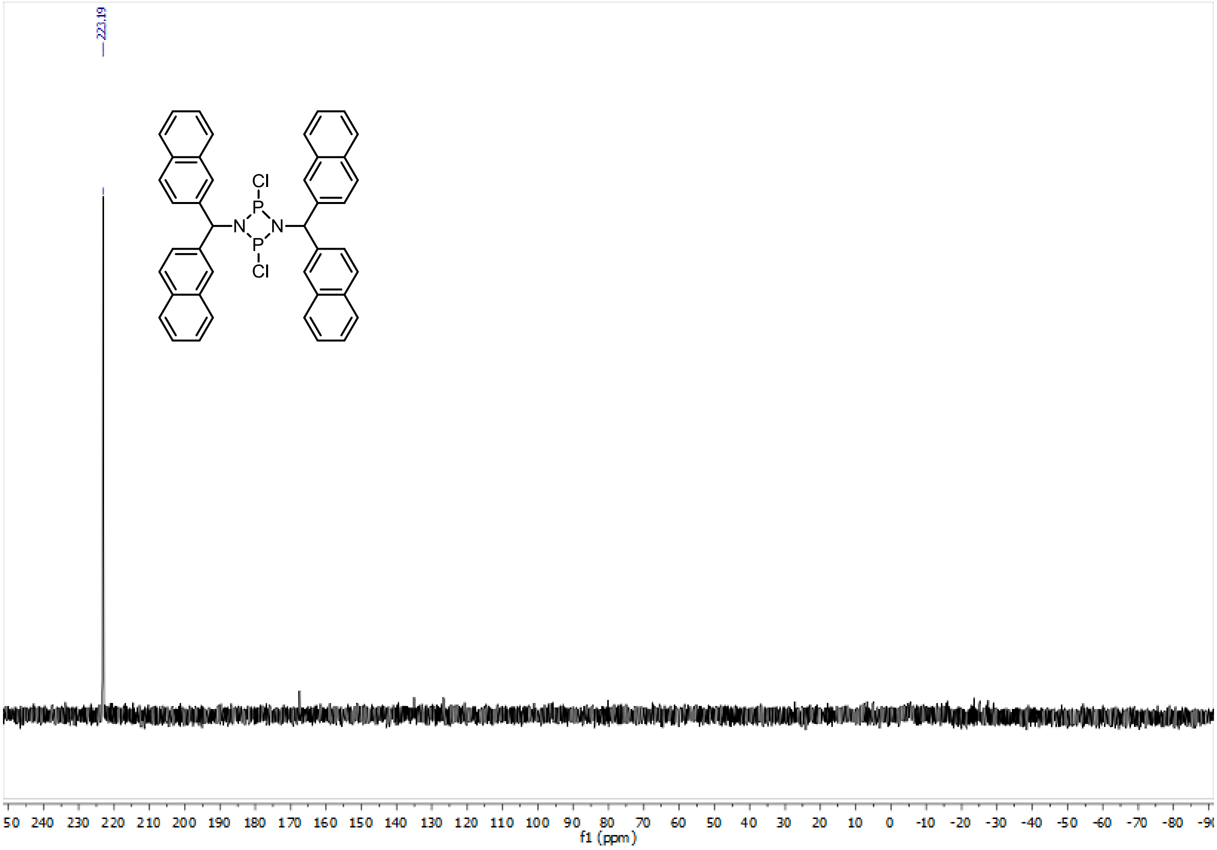

223.19
Cl
P
N
N
P
Cl
f1 (ppm)

SI-4

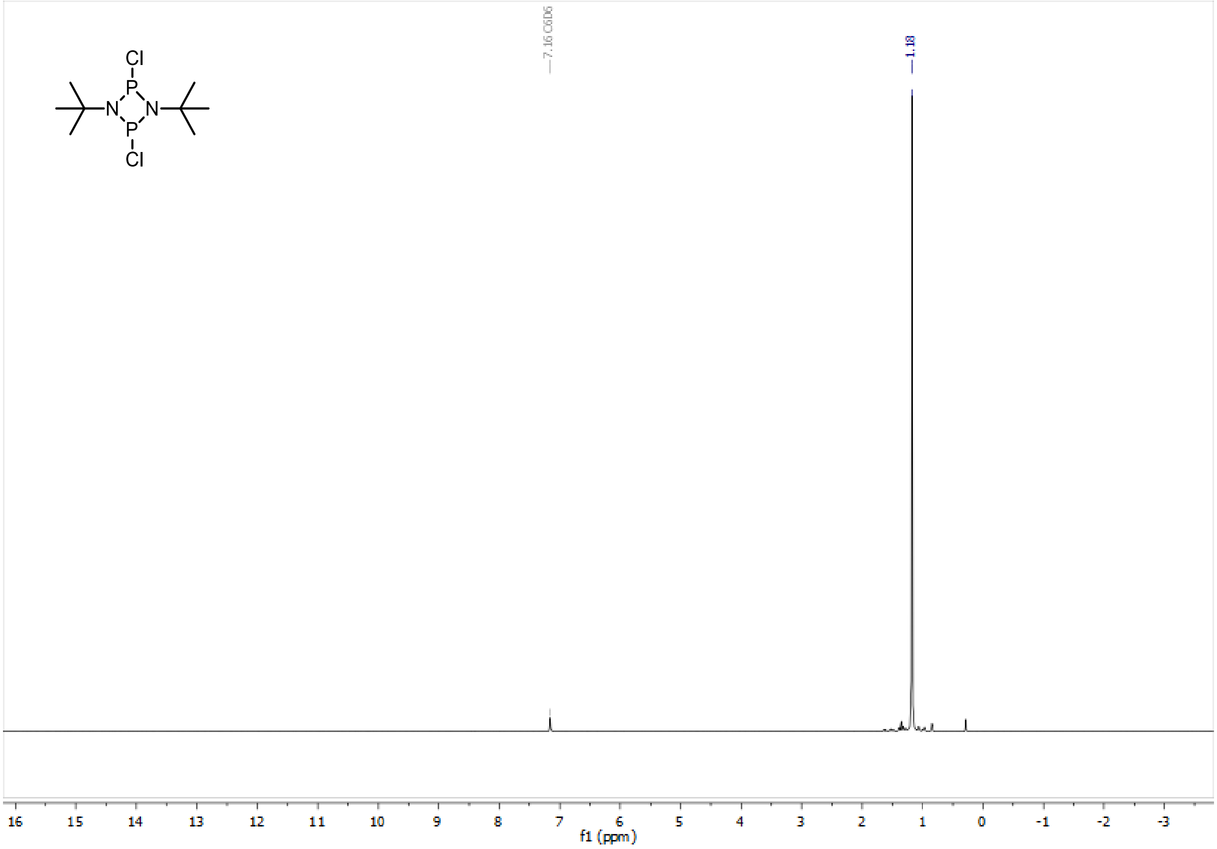

Cl
P
N
N
P
Cl
7.16 C6D6
1.18
16
15
14
13
12
11
10
9
8
7
6
5
4
3
2
1
0
-1
-2
-3
f1 (ppm)

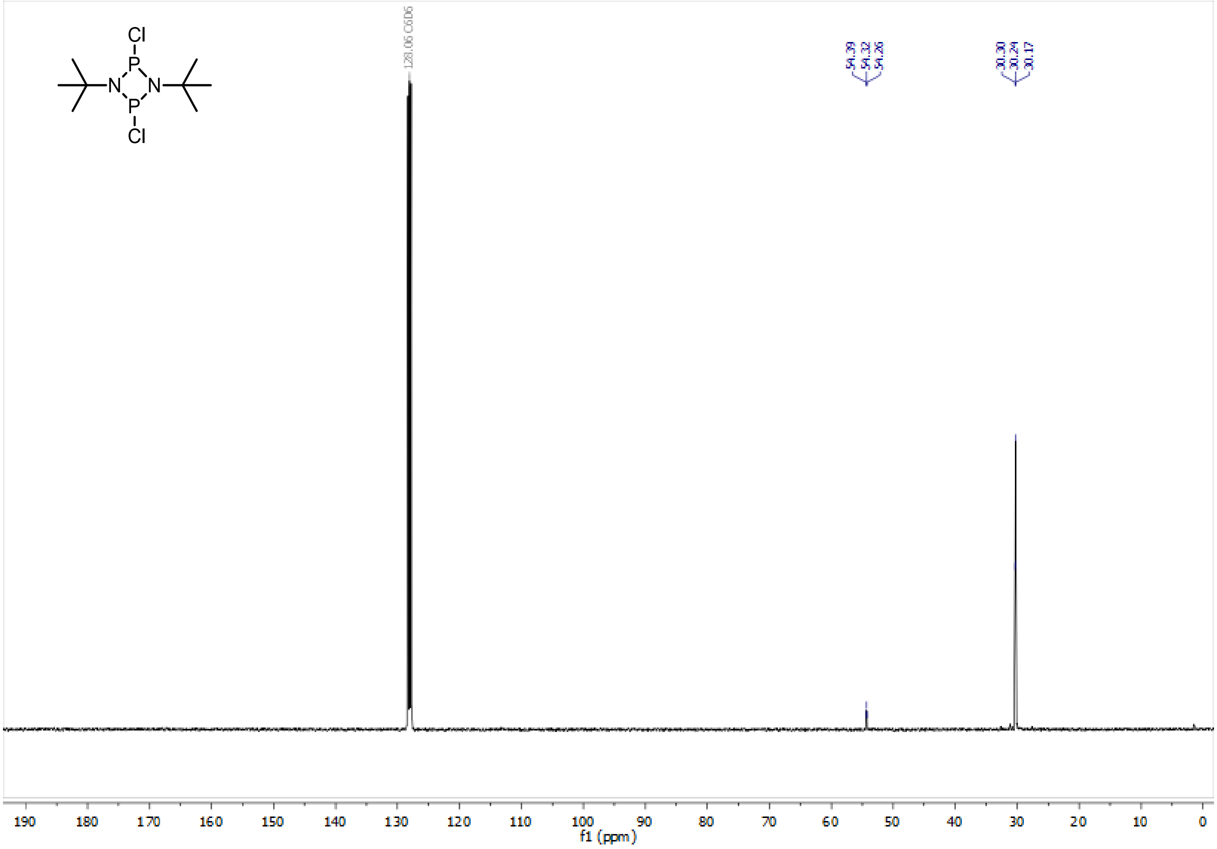

Cl
P
N
N
P
Cl
128.06 C6D6
54.39
54.32
54.26
30.30
30.24
30.17
190
180
170
160
150
140
130
120
110
100
90
80
70
60
50
40
30
20
10
0
f1 (ppm)

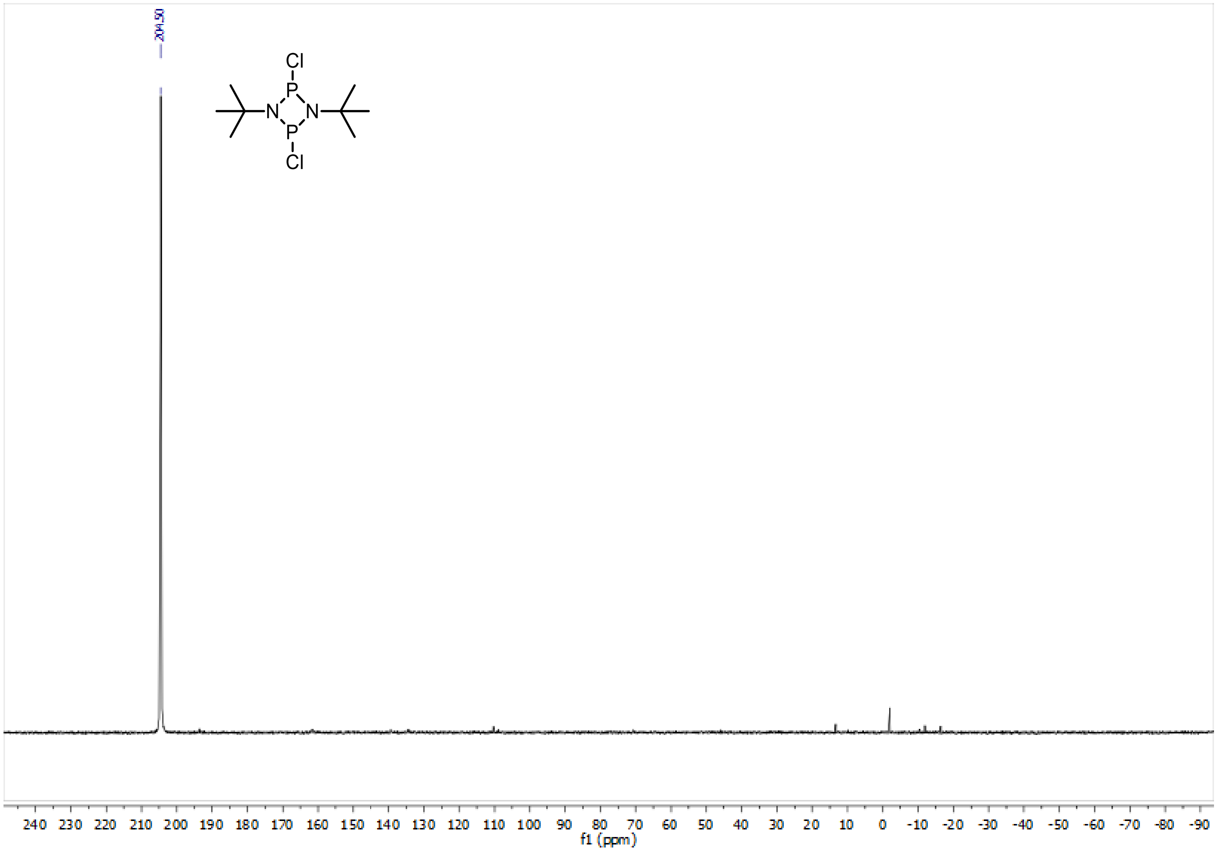

204.50
Cl
P
N
N
P
Cl
240 230 220 210 200 190 180 170 160 150 140 130 120 110 100 90 80 70 60 50 40 30 20 10 0 -10 -20 -30 -40 -50 -60 -70 -80 -90
f1 (ppm)

SI-5

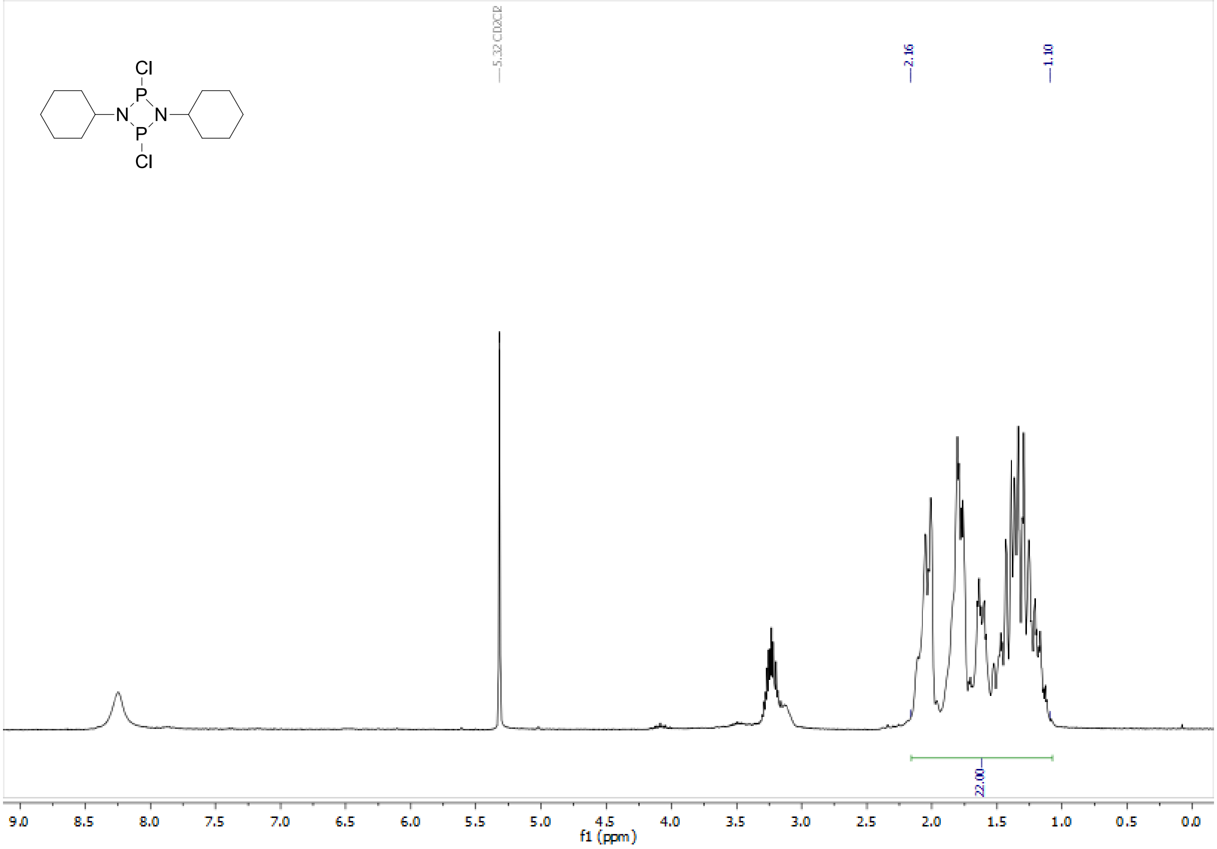

Cl
P
N
N
P
Cl
5.32 CD2Cl2
2.16
1.10
22.00
9.0
8.5
8.0
7.5
7.0
6.5
6.0
5.5
5.0
4.5
4.0
3.5
3.0
2.5
2.0
1.5
1.0
0.5
0.0
f1 (ppm)

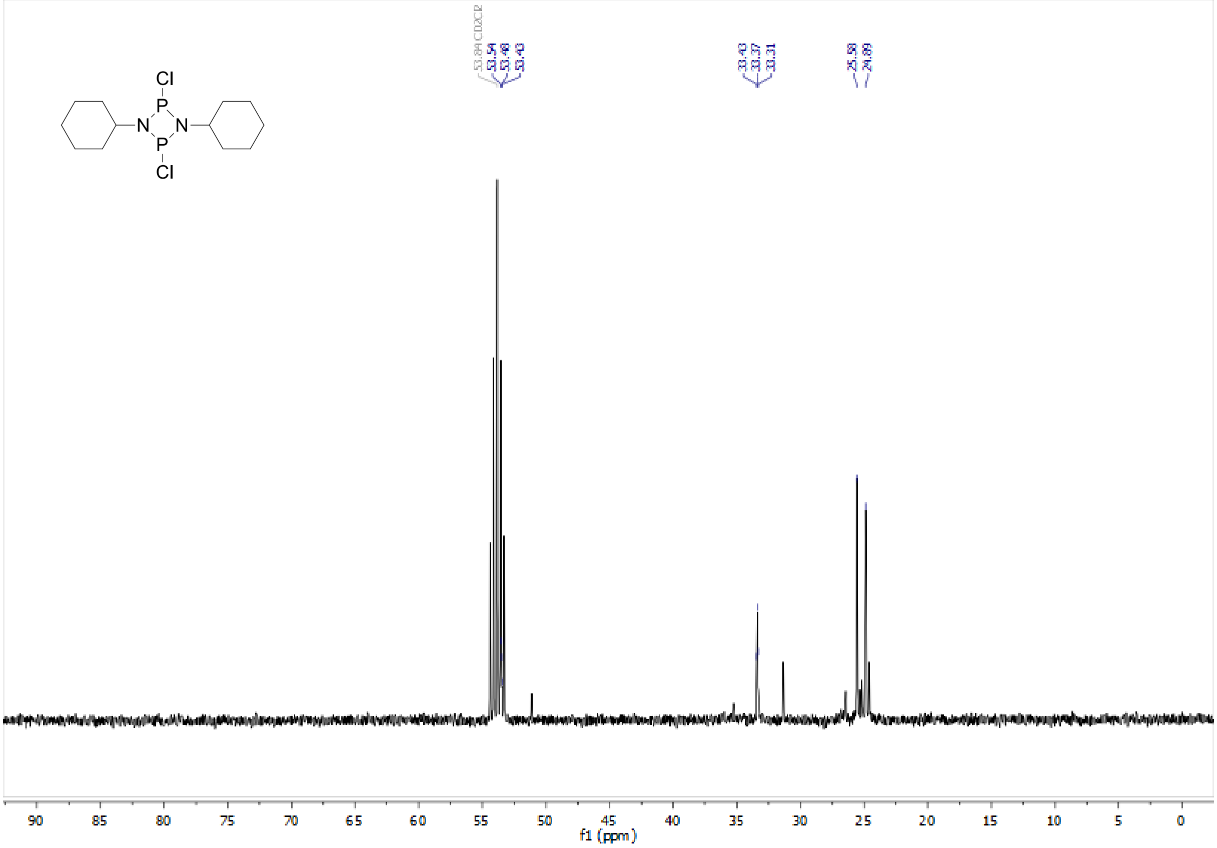

Cl
P
N
N
P
Cl
53.84 CD2Cl2
53.54
53.48
53.43
33.43
33.37
33.31
25.58
24.89
90
85
80
75
70
65
60
55
50
45
40
35
30
25
20
15
10
5
0
f1 (ppm)

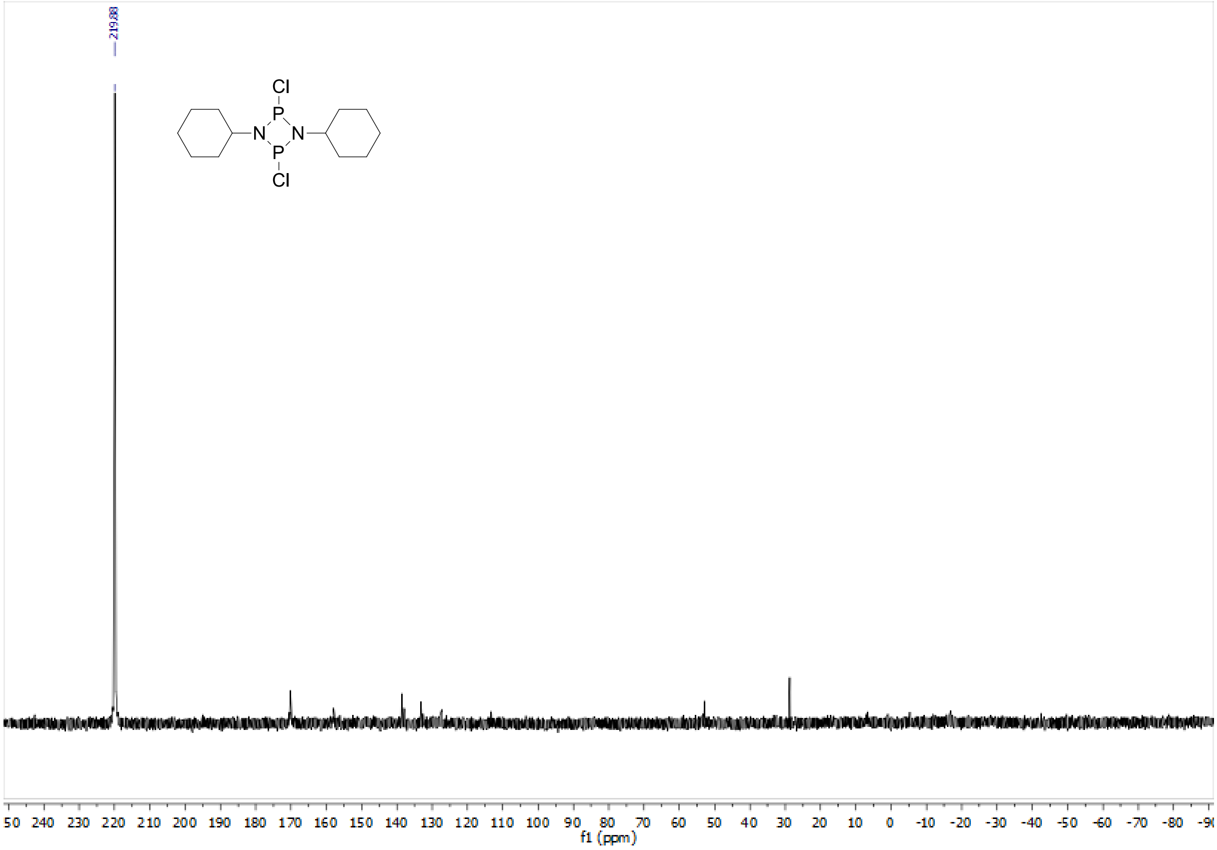

SI-8

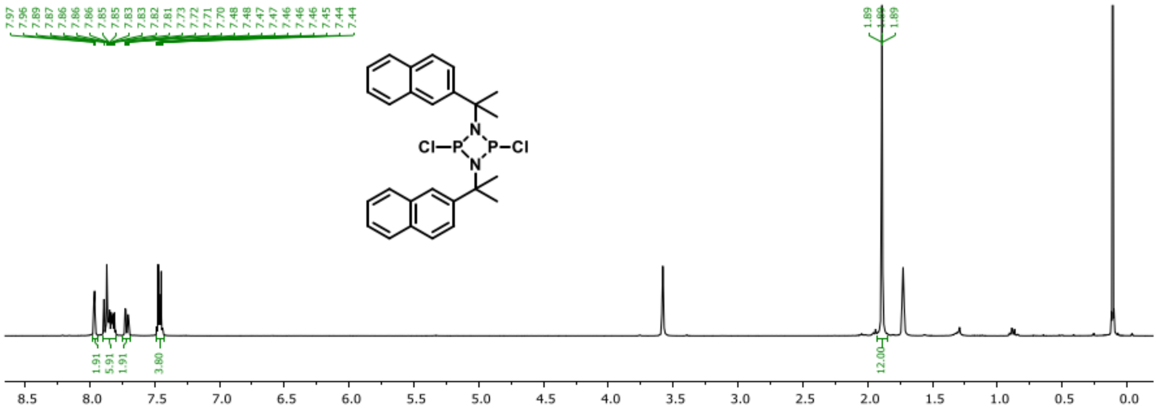

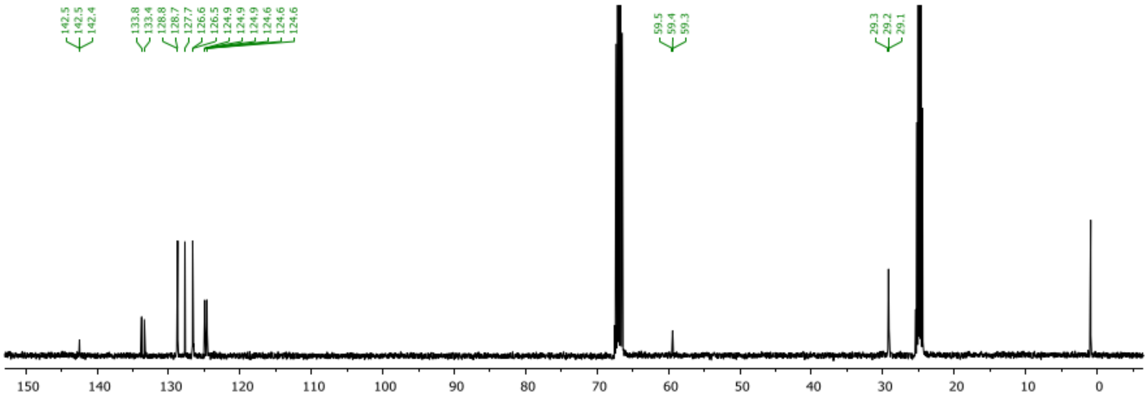

L5

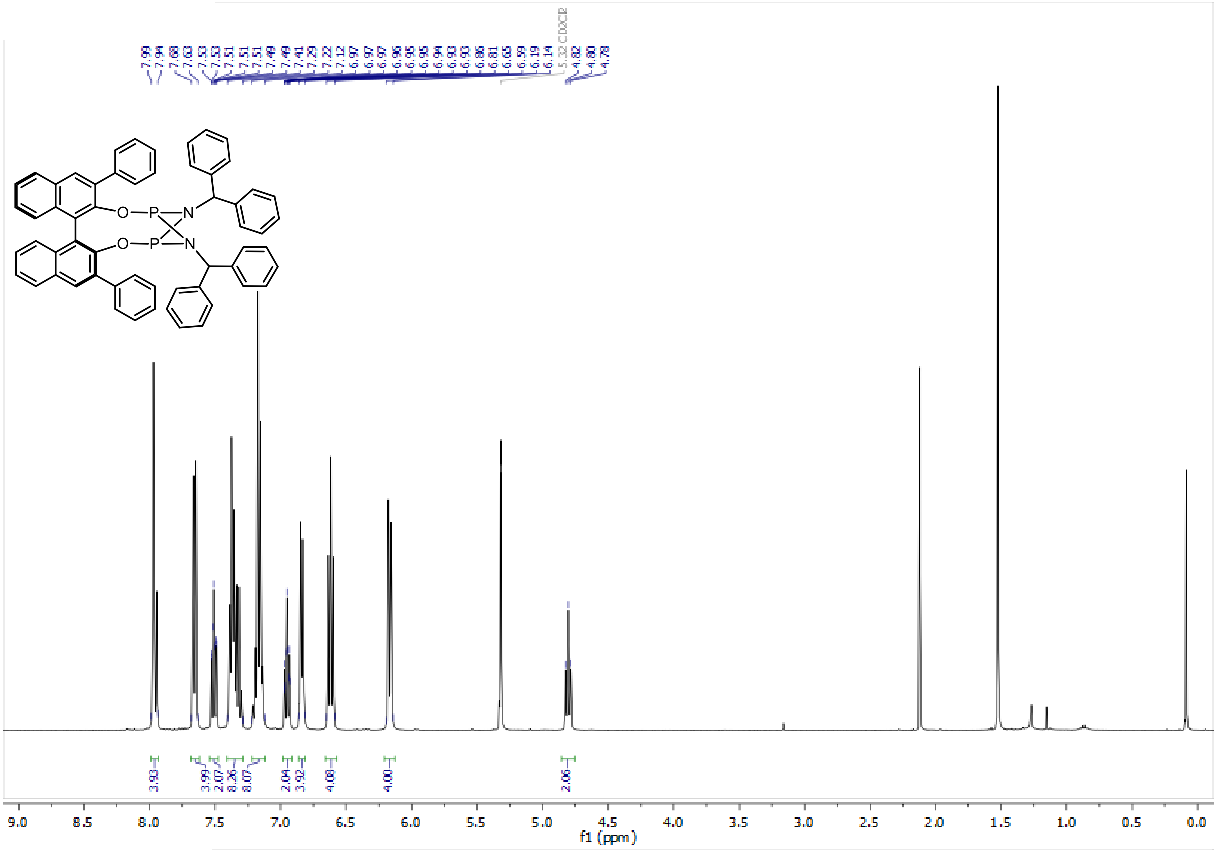

7.99
7.94
7.68
7.63
7.53
7.53
7.51
7.51
7.51
7.49
7.49
7.41
7.29
7.22
7.12
6.97
6.97
6.97
6.96
6.95
6.95
6.94
6.93
6.93
6.93
6.86
6.81
6.65
6.59
6.19
6.14
5.32 CD2Cl2
4.82
4.80
4.78
O
P
N
3.93
3.99
2.07
8.26
8.07
2.04
3.92
4.08
4.00
2.06
f1 (ppm)

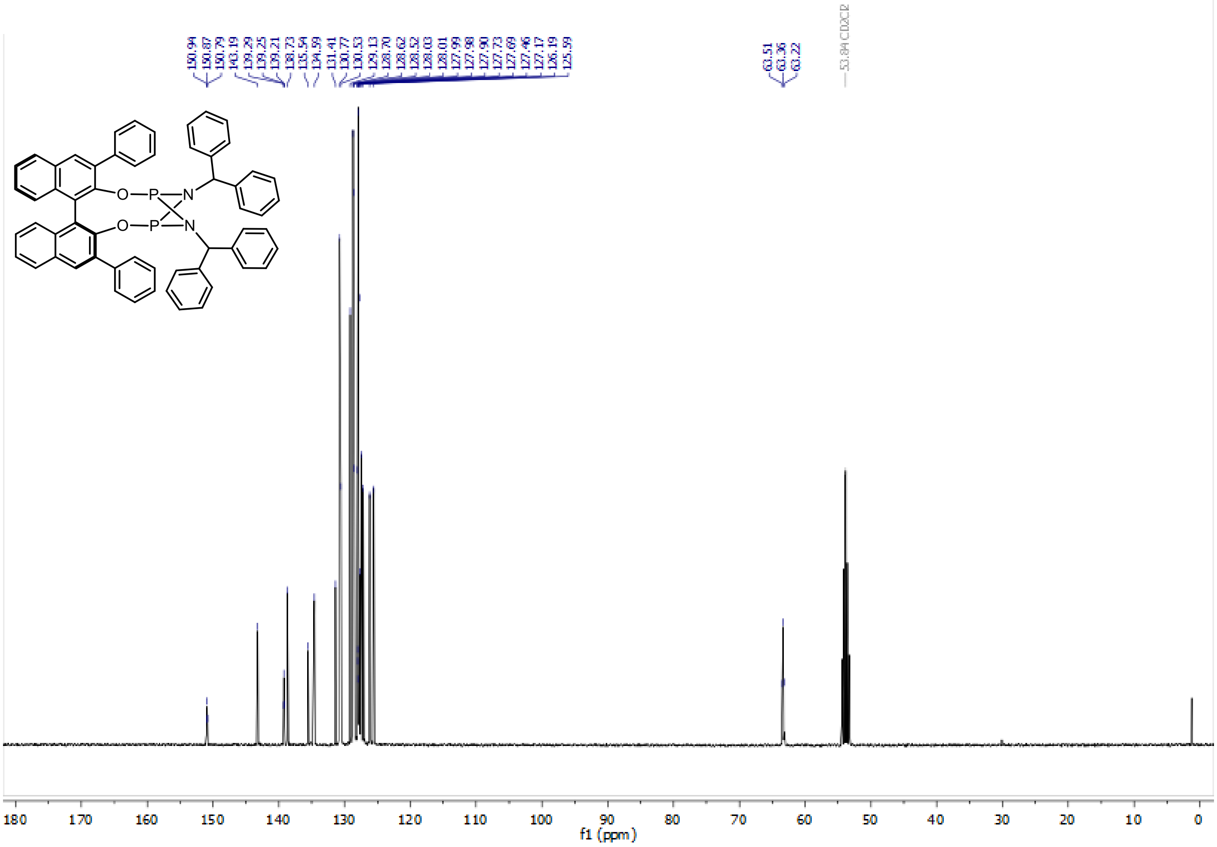

150.94
150.87
150.79
143.19
139.29
139.25
139.21
138.73
135.54
134.59
131.41
130.77
130.53
129.13
128.70
128.62
128.52
128.03
128.01
127.99
127.98
127.90
127.73
127.69
127.46
127.17
126.19
125.59
63.51
63.36
63.22
53.84 CD2Cl2
O
P
N
f1 (ppm)

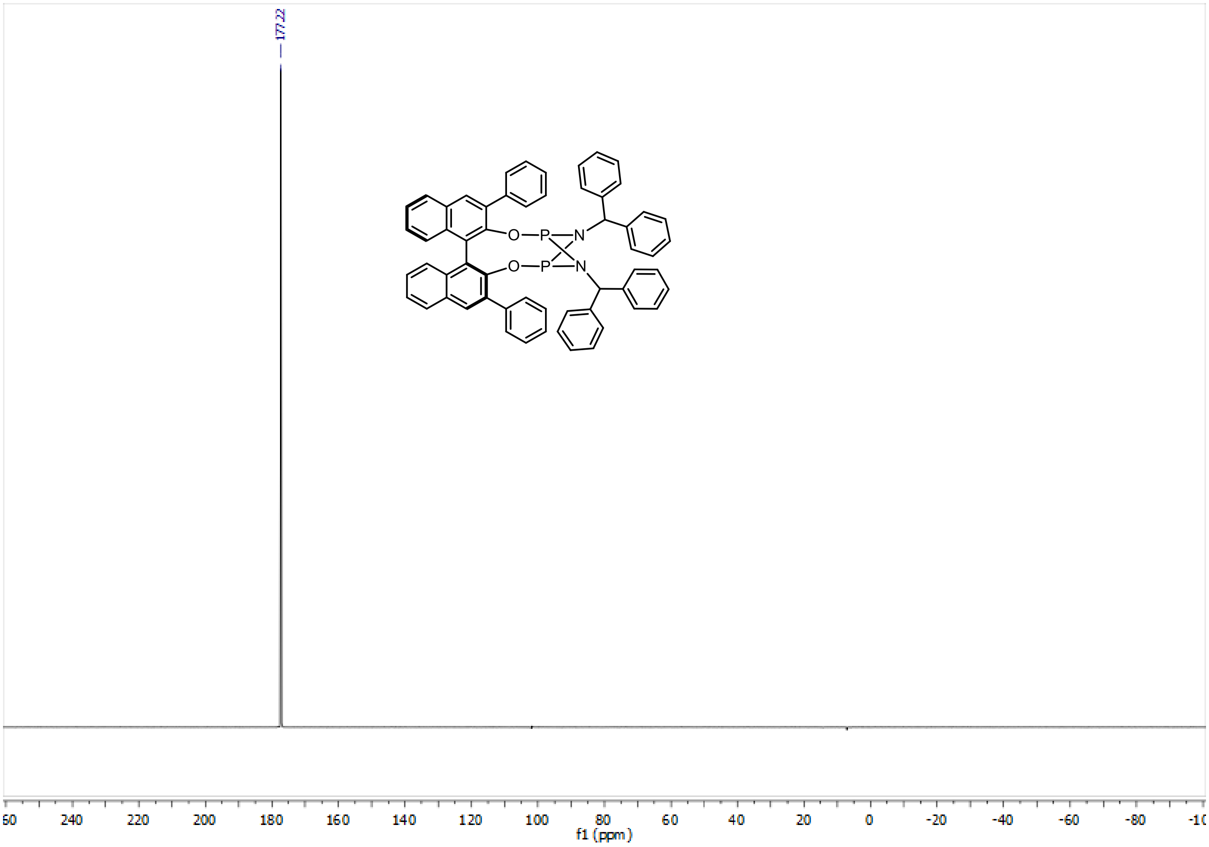

177.22
O
P
N
O
P
N
260
240
220
200
180
160
140
120
100
80
60
40
20
0
-20
-40
-60
-80
f1 (ppm)

L6

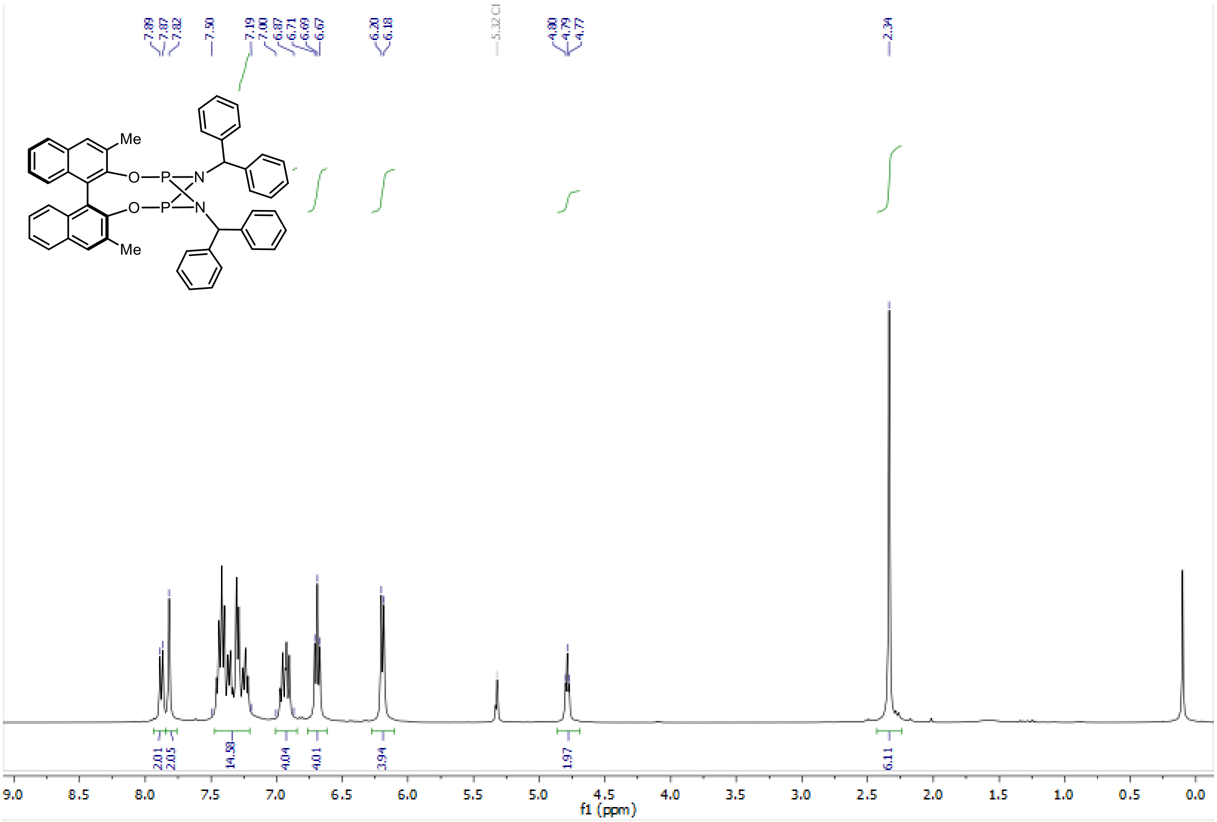

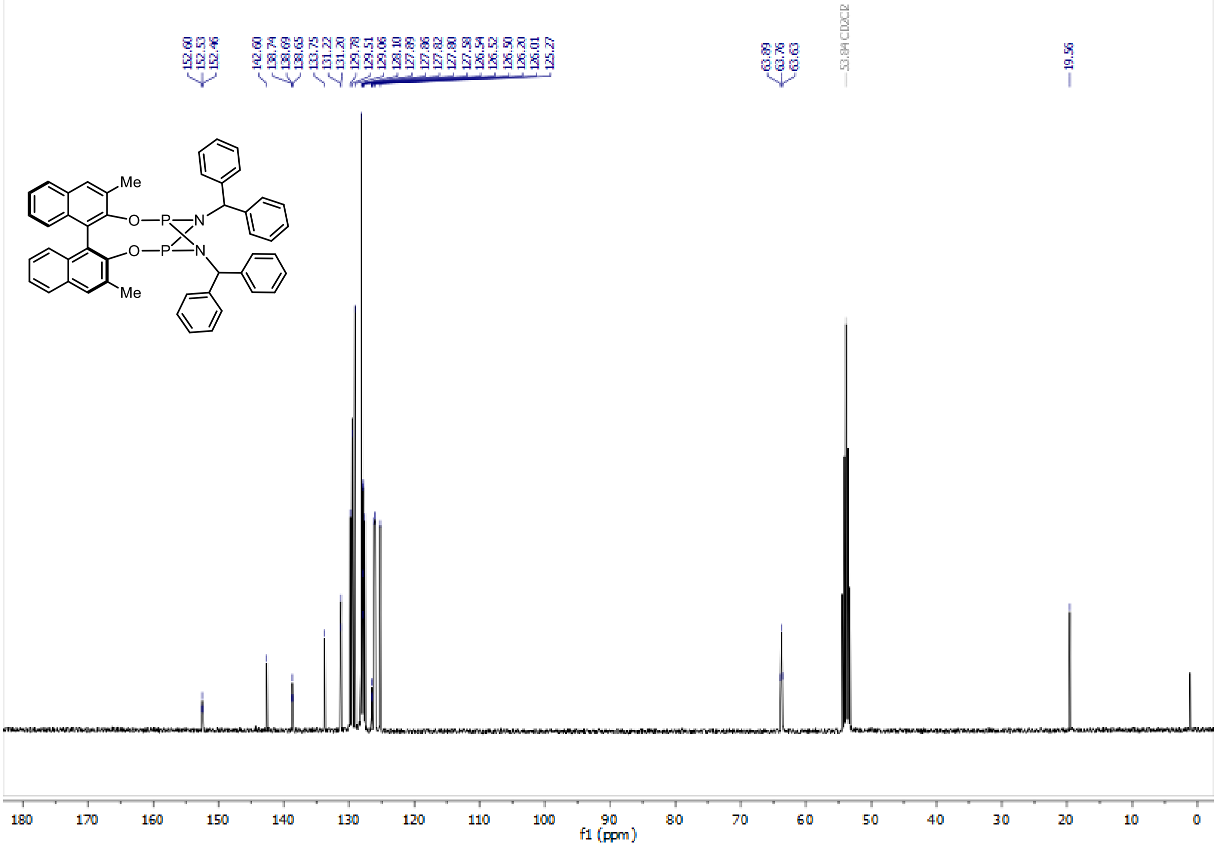

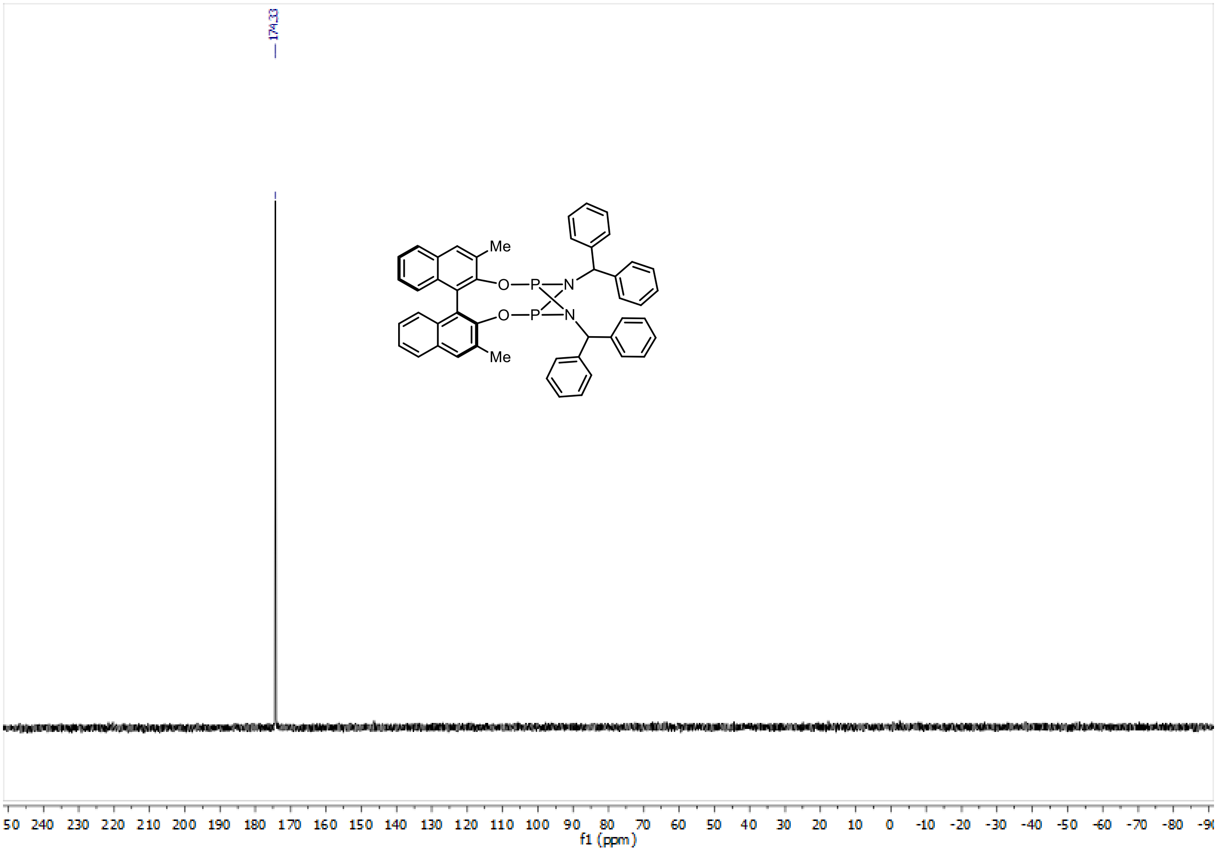

174.33
Me
O
P
N
Me
f1 (ppm)

L7

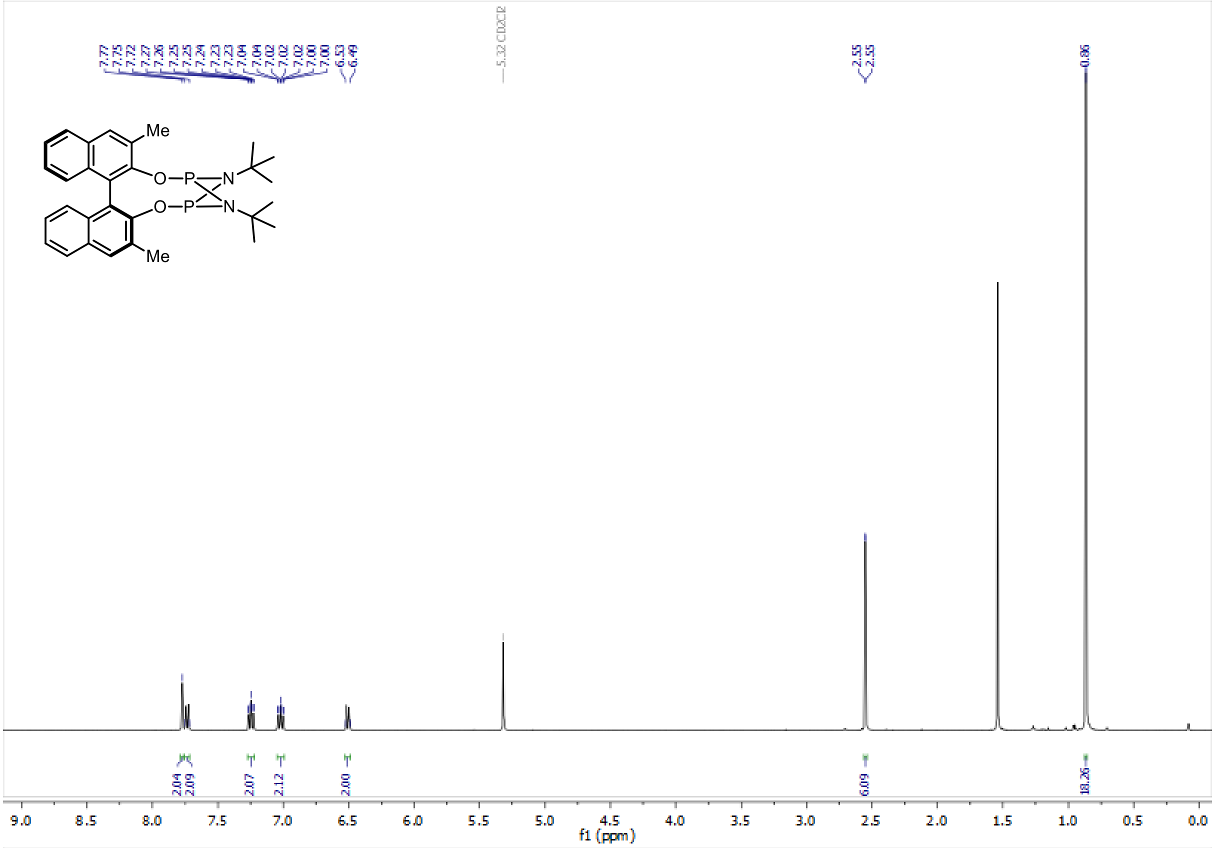

7.77
7.75
7.72
7.27
7.26
7.25
7.25
7.24
7.23
7.23
7.04
7.04
7.02
7.02
7.02
7.00
7.00
6.53
6.49
5.32 CD2Cl2
2.55
2.55
0.86
Me
O
P
N
Me
2.04
2.09
2.07
2.12
2.00
6.09
18.26
9.0
8.5
8.0
7.5
7.0
6.5
6.0
5.5
5.0
4.5
4.0
3.5
3.0
2.5
2.0
1.5
1.0
0.5
0.0
f1 (ppm)

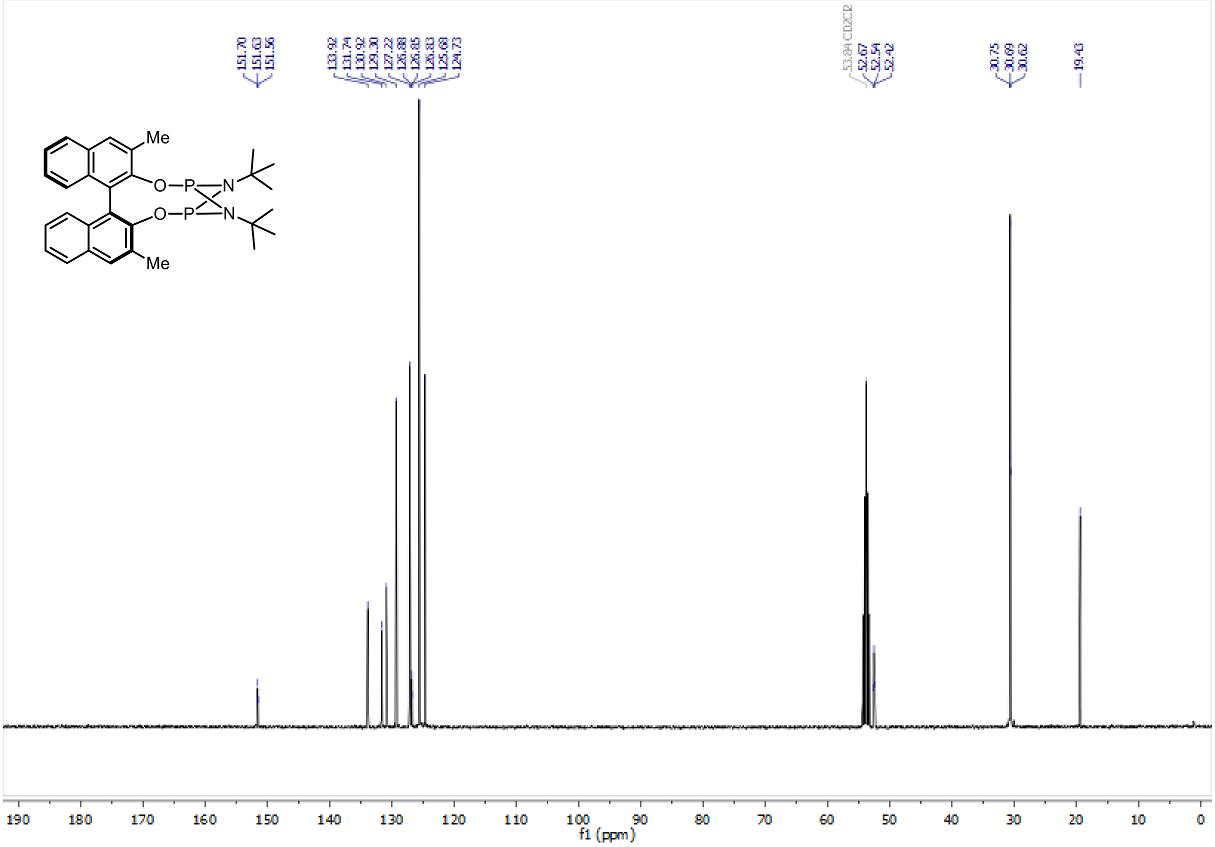

151.70
151.63
151.56
133.92
131.74
130.92
129.30
127.22
126.88
126.85
126.83
125.68
124.73
53.84 CD2Cl2
52.67
52.54
52.42
30.75
30.69
30.62
19.43
Me
O
P
N
Me
190
180
170
160
150
140
130
120
110
100
90
80
70
60
50
40
30
20
10
0
f1 (ppm)

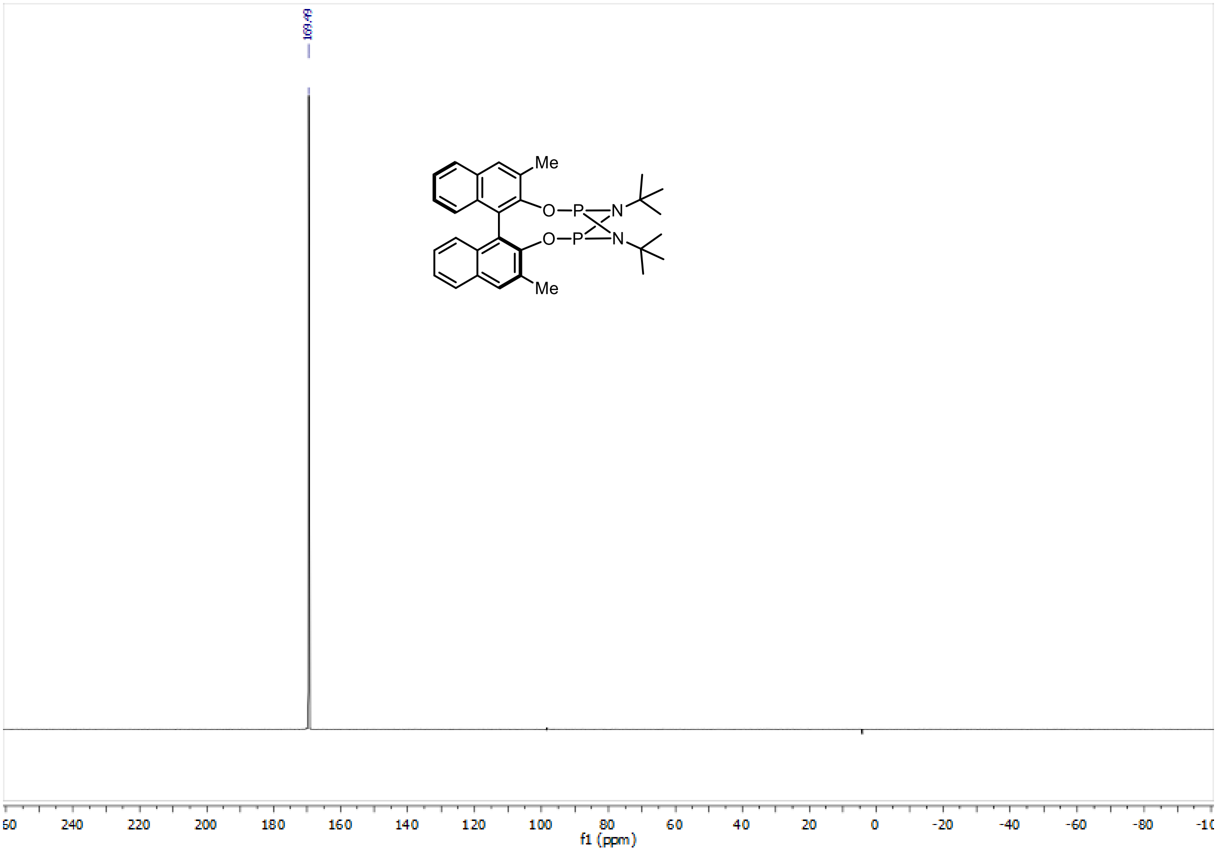

169.49
Me
O
P
N
O
P
N
Me
60
240
220
200
180
160
140
120
100
80
60
40
20
0
-20
-40
-60
-80
f1 (ppm)

L8

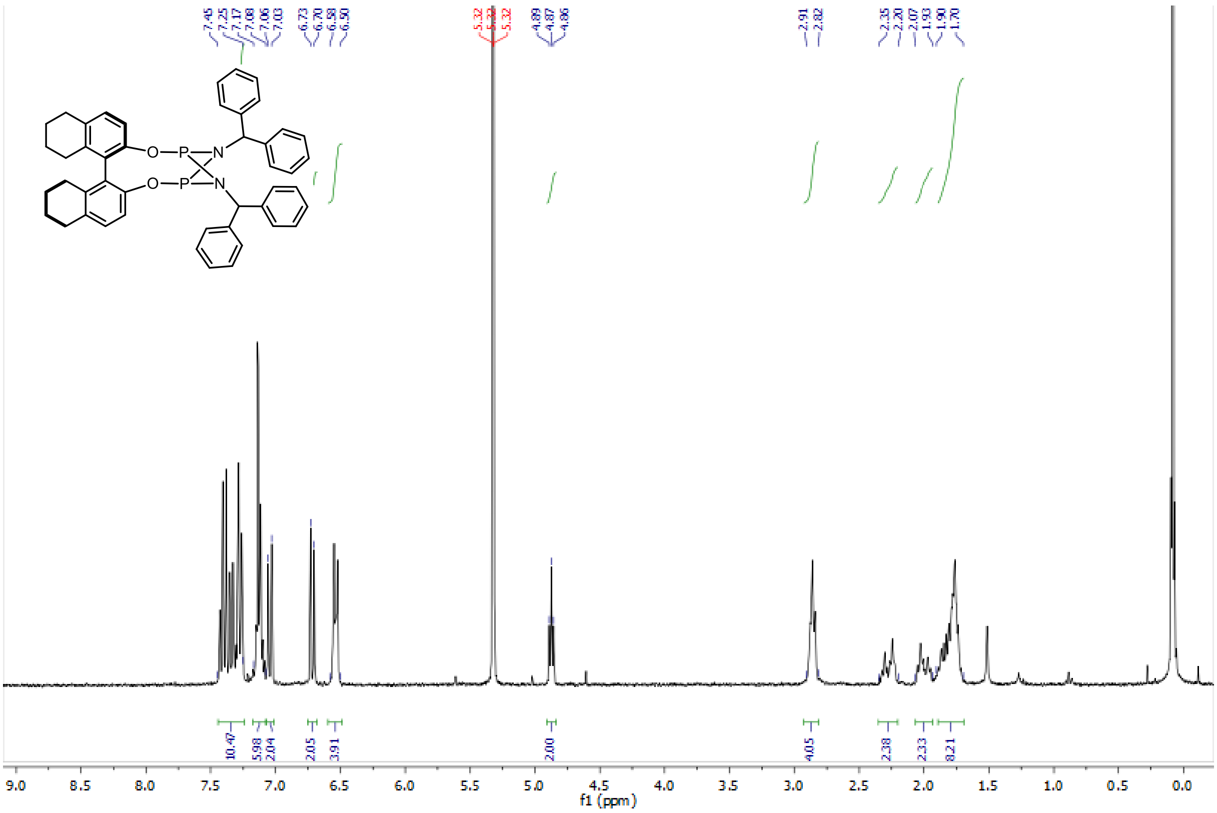

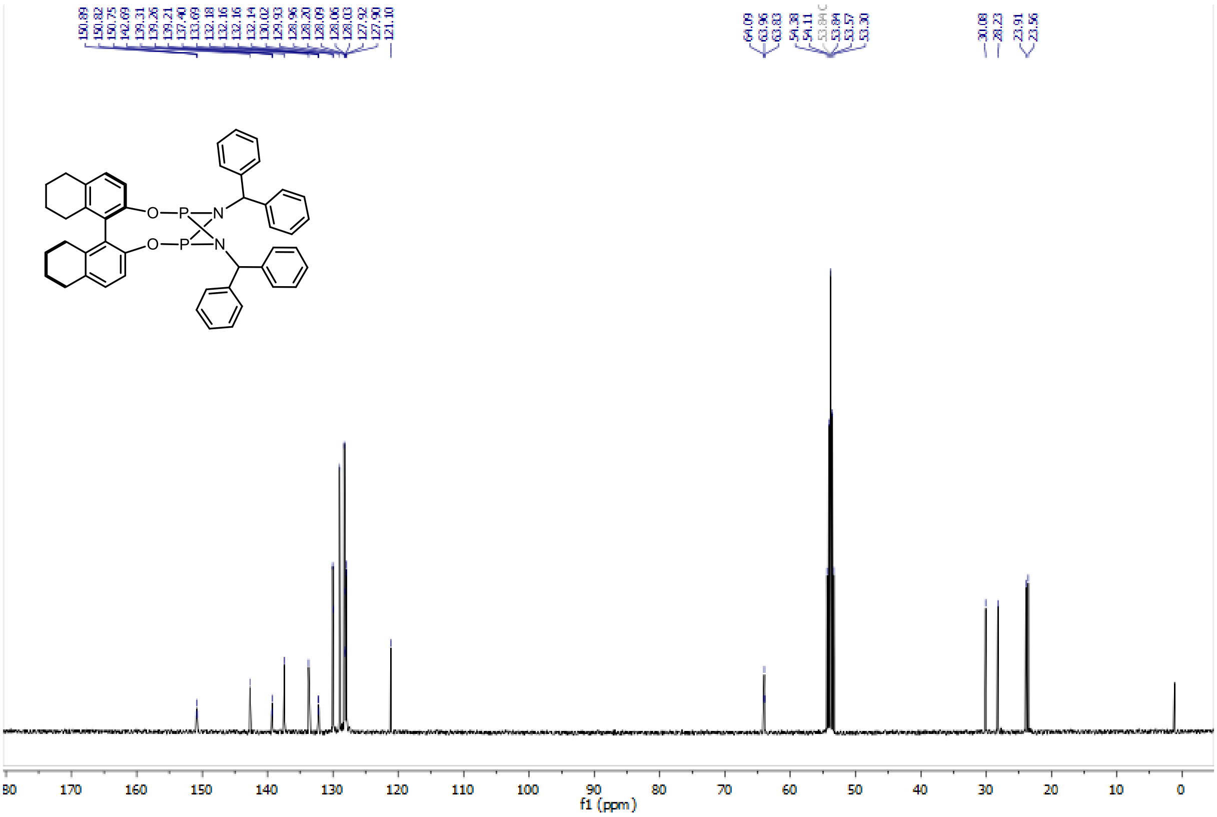

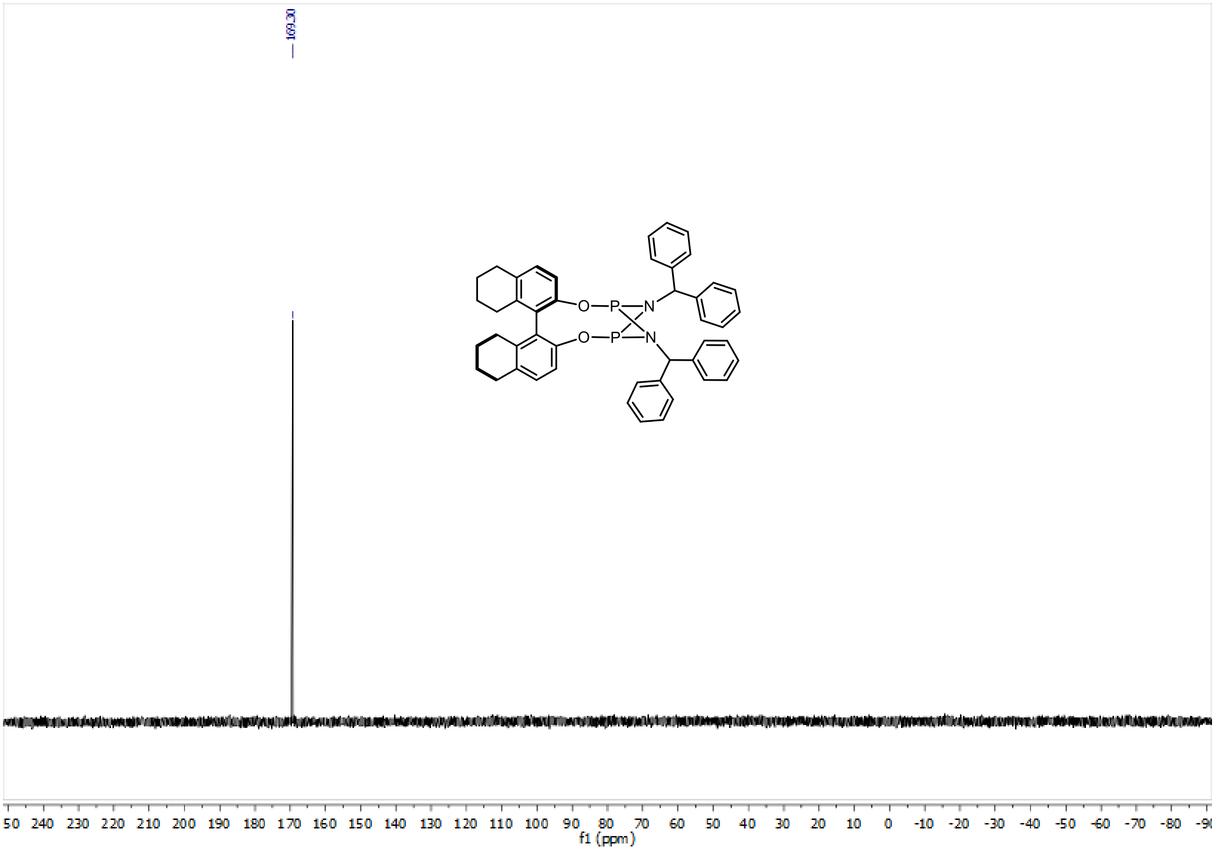
169.30
O
P
N
O
P
N
50 240 230 220 210 200 190 180 170 160 150 140 130 120 110 100 90 80 70 60 50 40 30 20 10 0 -10 -20 -30 -40 -50 -60 -70 -80
f1 (ppm)

L9

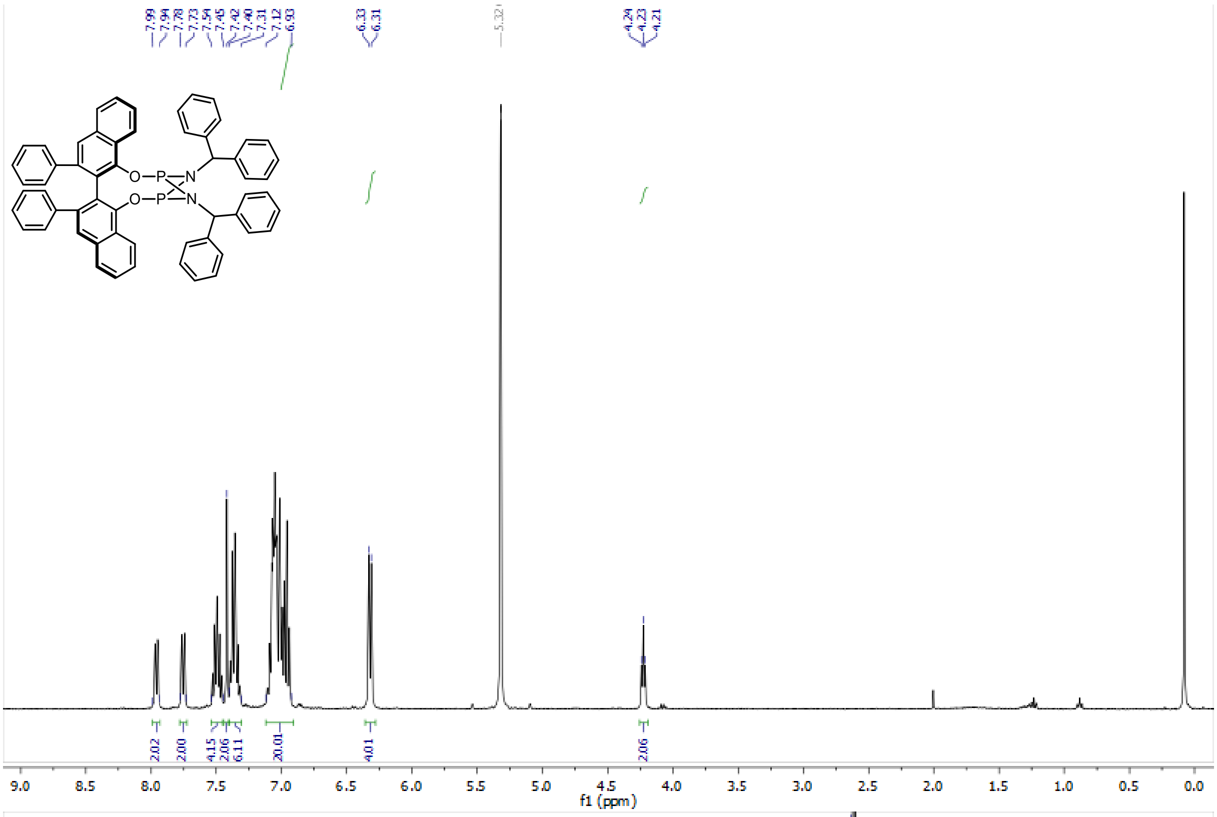

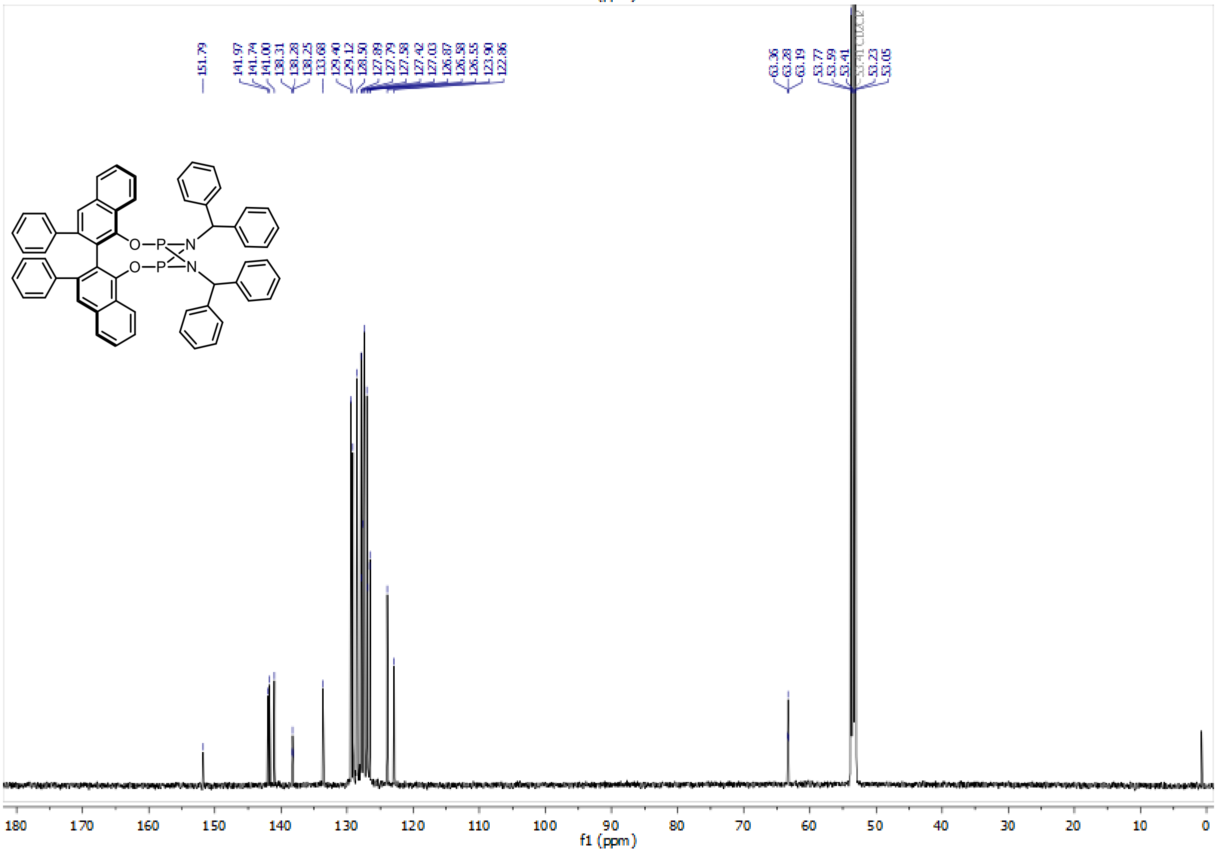

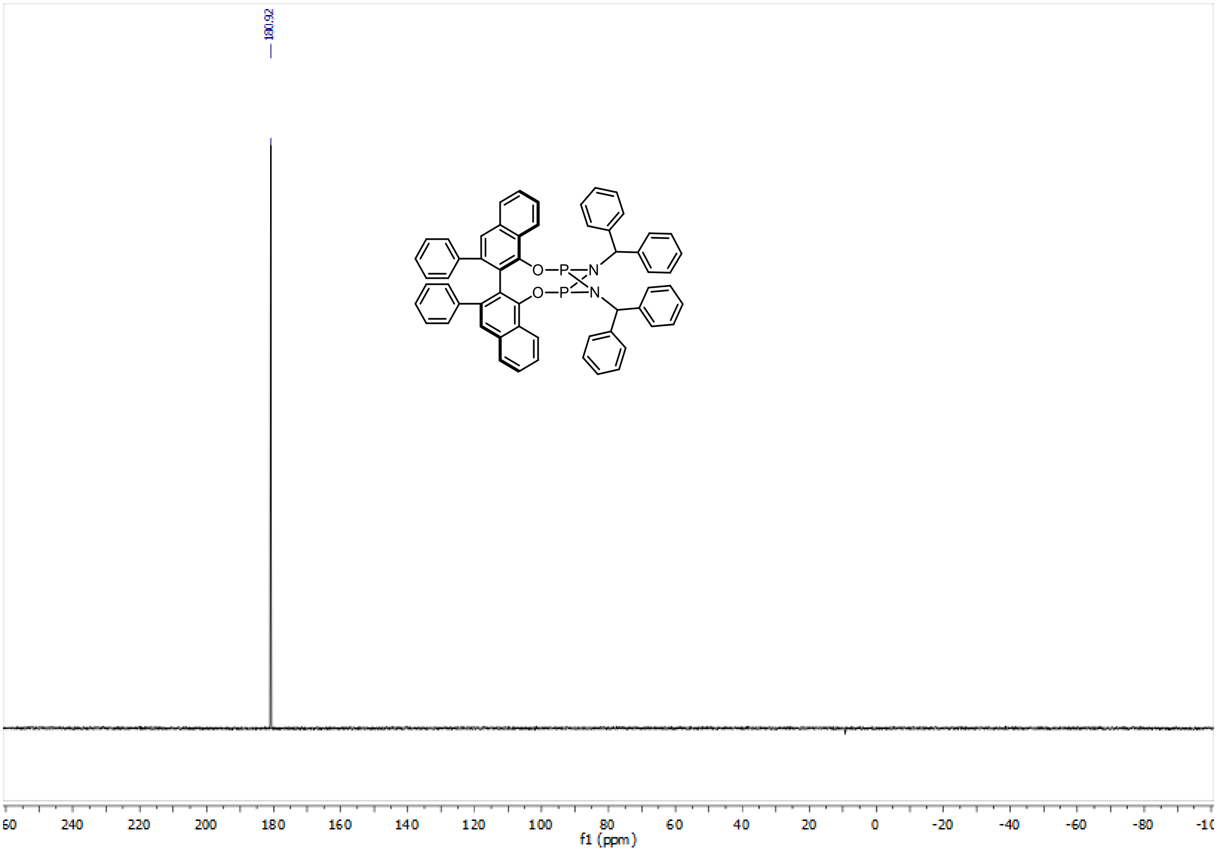

180.92
f1 (ppm)

L15

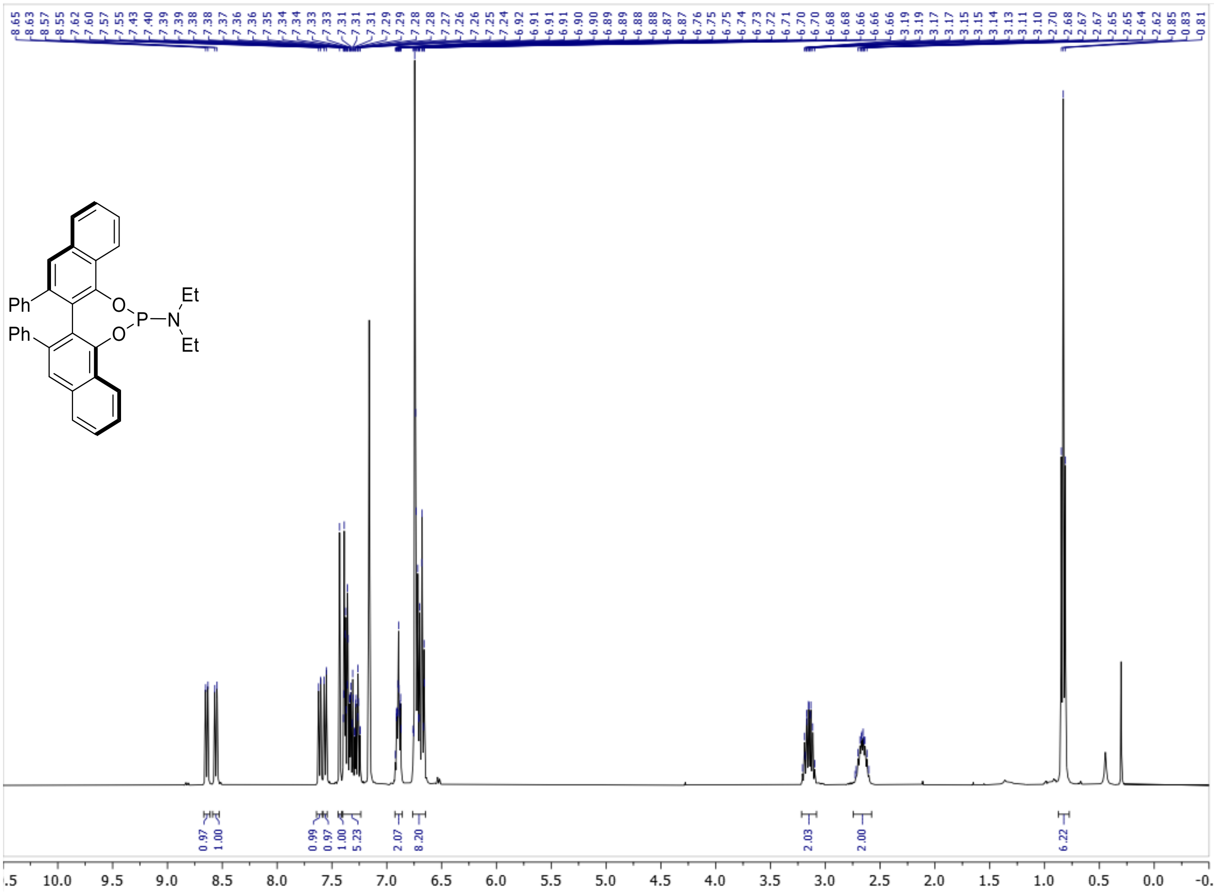
8.65 8.63 8.57 8.55 7.62 7.60 7.57 7.55 7.43 7.40 7.39 7.39 7.38 7.38 7.37 7.36 7.36 7.35 7.34 7.34 7.33 7.33 7.31 7.31 7.31 7.29 7.29 7.28 7.28 7.27 7.26 7.26 7.25 7.24 6.92 6.91 6.91 6.91 6.90 6.89 6.89 6.88 6.88 6.87 6.87 6.76 6.75 6.75 6.74 6.73 6.72 6.71 6.70 6.70 6.68 6.68 6.66 6.66 6.66 3.19 3.19 3.17 3.17 3.15 3.15 3.14 3.13 3.11 3.10 2.70 2.68 2.67 2.67 2.65 2.65 2.64 2.62 0.85 0.83 0.81
Ph
Ph
O
O
P
N
Et
Et
0.97 1.00 0.99 0.97 1.00 5.23 2.07 8.20 2.03 2.00 6.22
10.0 9.5 9.0 8.5 8.0 7.5 7.0 6.5 6.0 5.5 5.0 4.5 4.0 3.5 3.0 2.5 2.0 1.5 1.0 0.5 0.0

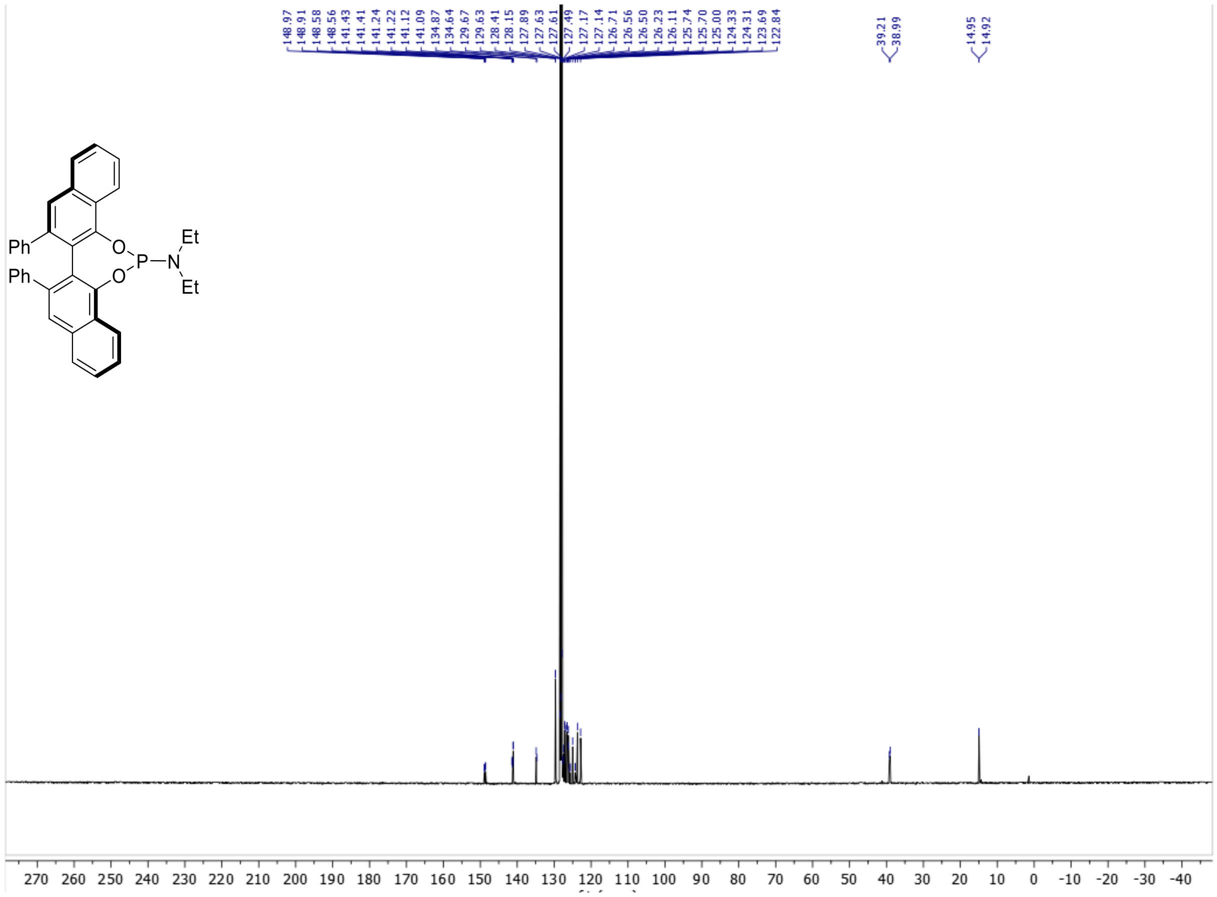
148.97 148.91 148.58 148.56 141.43 141.41 141.24 141.22 141.12 141.09 134.87 134.64 129.67 129.63 128.41 128.15 127.89 127.63 127.61 127.49 127.17 127.14 126.71 126.56 126.50 126.23 126.11 125.74 125.70 125.00 124.33 124.31 123.69 122.84
39.21 38.99
14.95 14.92
Ph
Ph
O
O
P
N
Et
Et
270 260 250 240 230 220 210 200 190 180 170 160 150 140 130 120 110 100 90 80 70 60 50 40 30 20 10 0 -10 -20 -30 -40

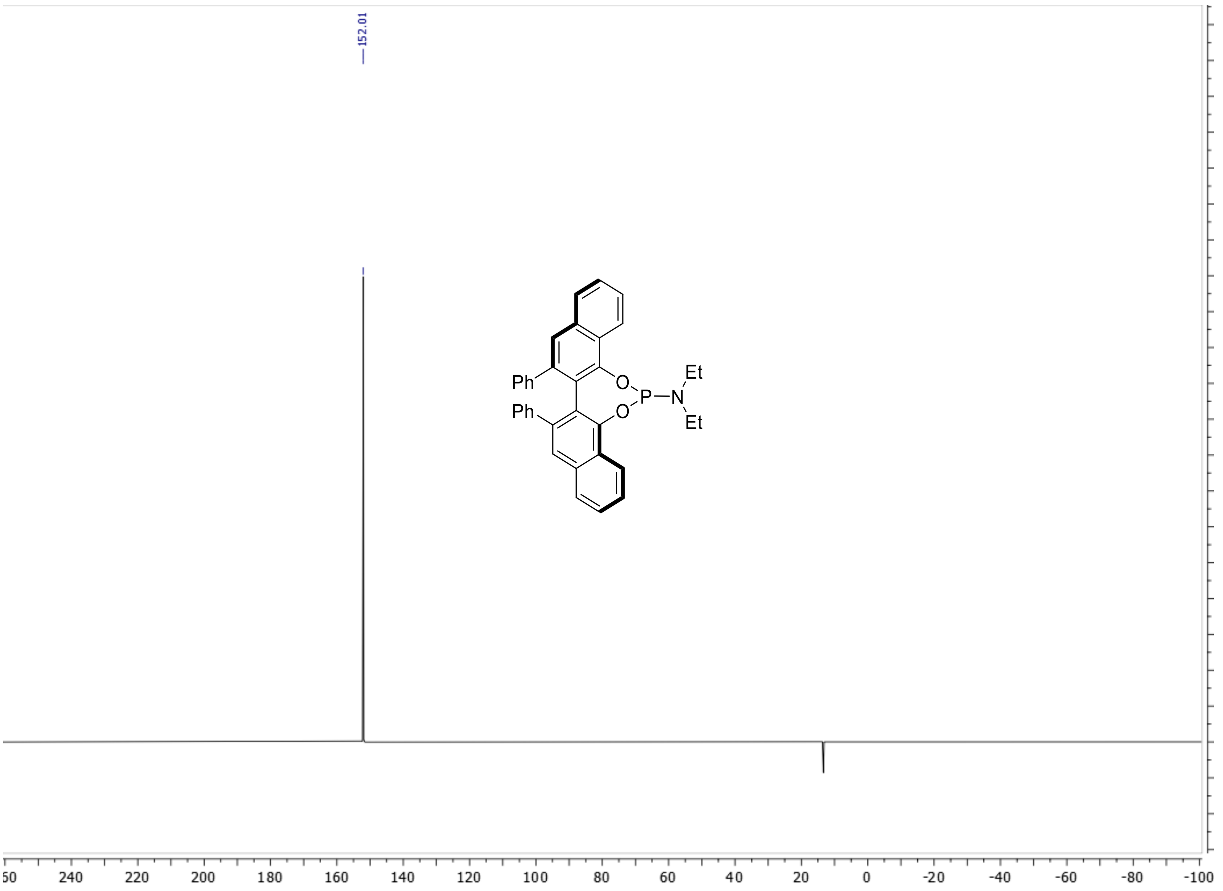
152.01
Ph
Ph
O
O
P
N
Et
Et
60
240
220
200
180
160
140
120
100
80
60
40
20
0
-20
-40
-60
-80
-100

L16

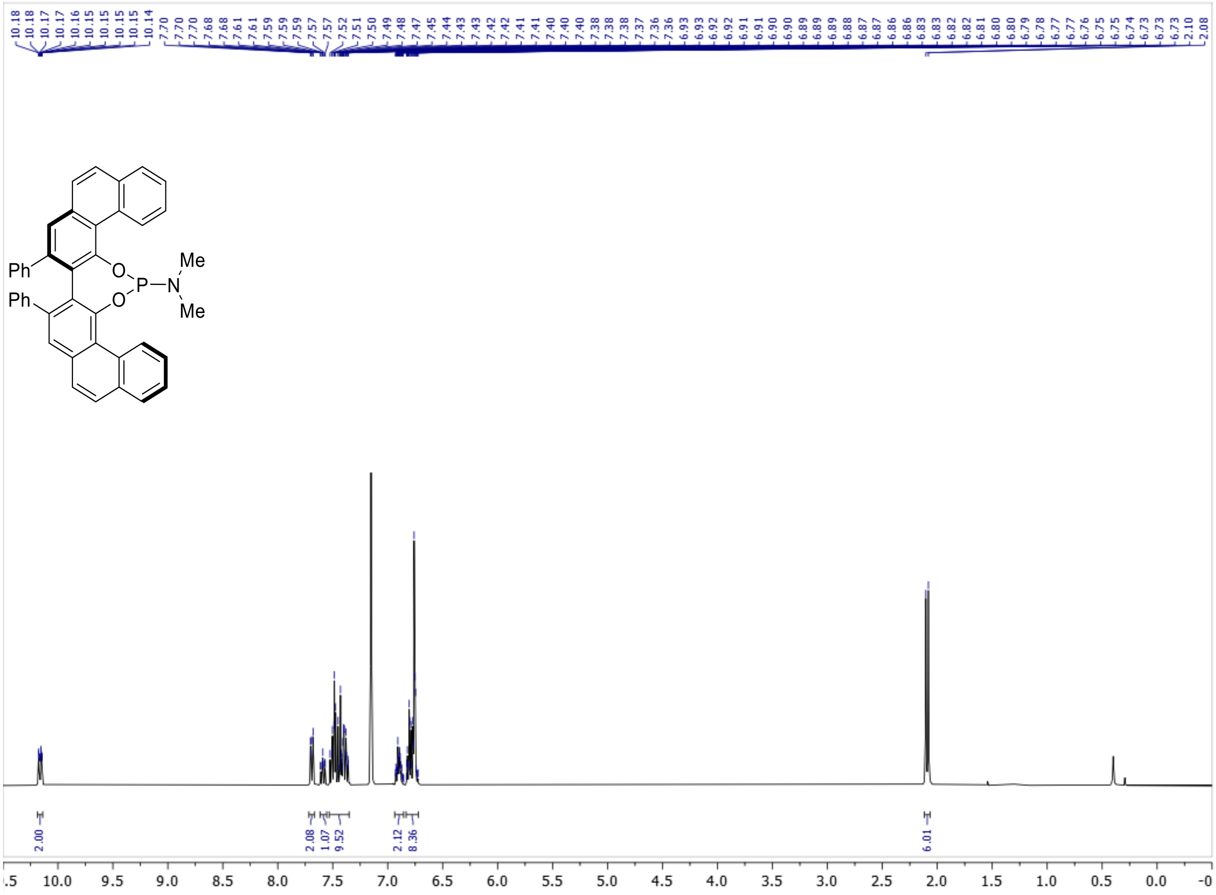
10.18
10.18
10.17
10.16
10.15
10.15
10.15
10.15
10.14
7.70
7.70
7.68
7.61
7.61
7.59
7.59
7.59
7.57
7.57
7.52
7.51
7.50
7.49
7.48
7.47
7.45
7.44
7.43
7.43
7.42
7.42
7.41
7.41
7.40
7.40
7.40
7.38
7.38
7.38
7.37
7.36
7.36
6.93
6.93
6.92
6.92
6.91
6.91
6.90
6.89
6.89
6.89
6.88
6.87
6.87
6.86
6.86
6.83
6.83
6.82
6.82
6.81
6.80
6.80
6.79
6.78
6.77
6.77
6.76
6.75
6.75
6.74
6.73
6.73
6.73
2.10
2.08
Ph
Ph
O
O
P
N
Me
Me
2.00
2.08
1.07
9.52
2.12
8.36
6.01
10.0 9.5 9.0 8.5 8.0 7.5 7.0 6.5 6.0 5.5 5.0 4.5 4.0 3.5 3.0 2.5 2.0 1.5 1.0 0.5 0.0

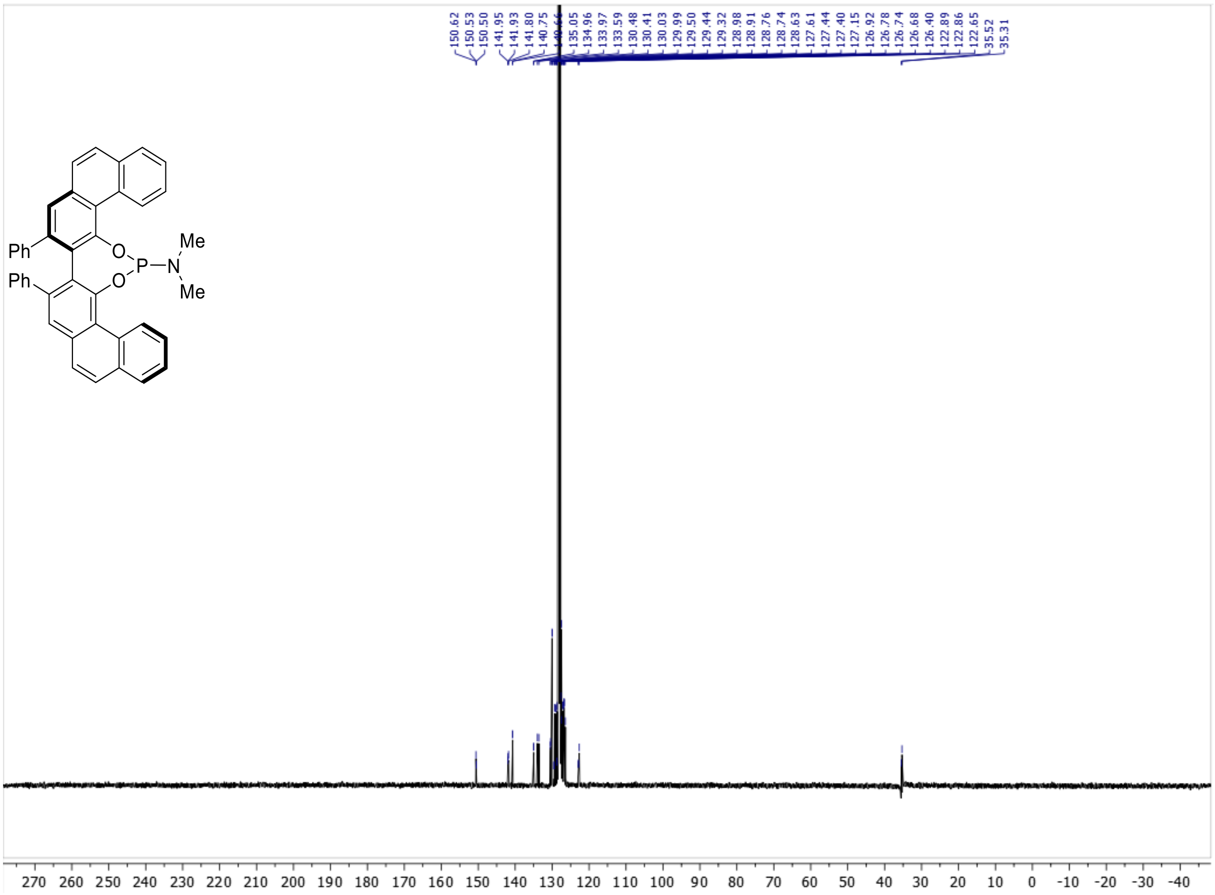
150.62
150.53
150.50
141.95
141.93
141.80
140.75
135.05
134.96
133.97
133.59
130.48
130.41
130.03
129.99
129.50
129.44
129.32
128.98
128.91
128.76
128.74
128.63
127.61
127.44
127.40
127.15
126.92
126.78
126.74
126.68
126.40
122.89
122.86
122.65
35.52
35.31
Ph
Ph
O
O
P
N
Me
Me
270 260 250 240 230 220 210 200 190 180 170 160 150 140 130 120 110 100 90 80 70 60 50 40 30 20 10 0 -10 -20 -30 -40

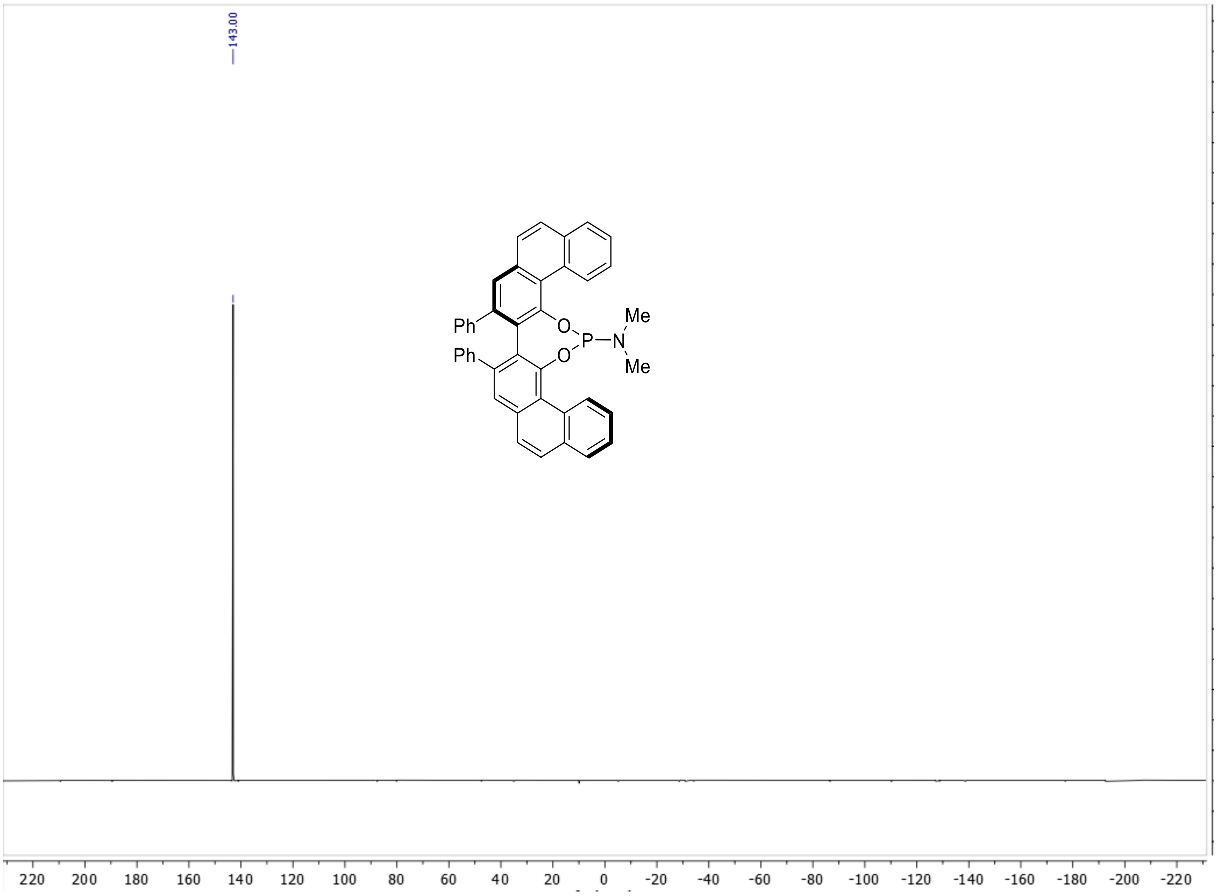

143.00
220
200
180
160
140
120
100
80
60
40
20
0
-20
-40
-60
-80
-100
-120
-140
-160
-180
-200
-220
Ph
Ph
O
O
P
N
Me
Me

L17

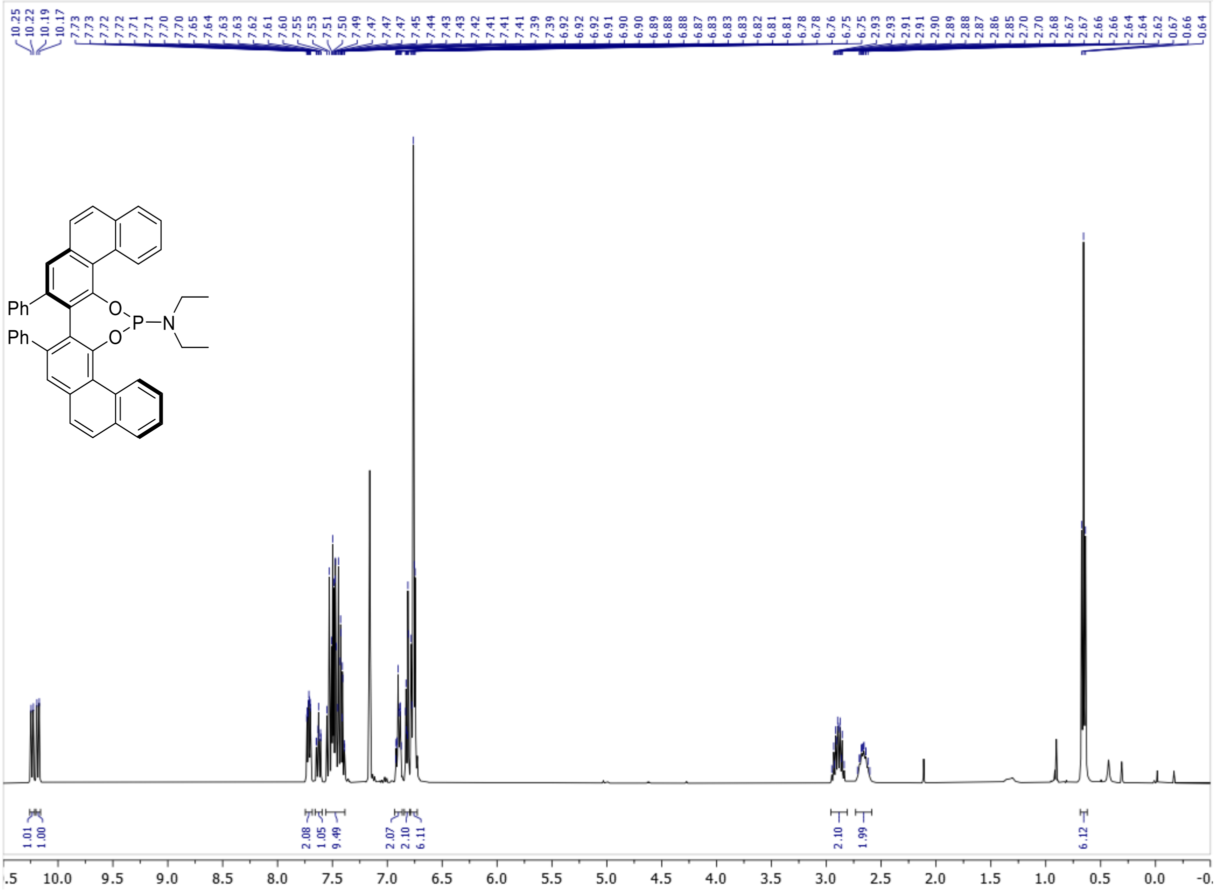

10.25
10.22
10.19
10.17
2.08
1.05
9.49
2.07
2.10
6.11
2.10
1.99
6.12
10.0 9.5 9.0 8.5 8.0 7.5 7.0 6.5 6.0 5.5 5.0 4.5 4.0 3.5 3.0 2.5 2.0 1.5 1.0 0.5 0.0
Ph
Ph
O
O
P
N

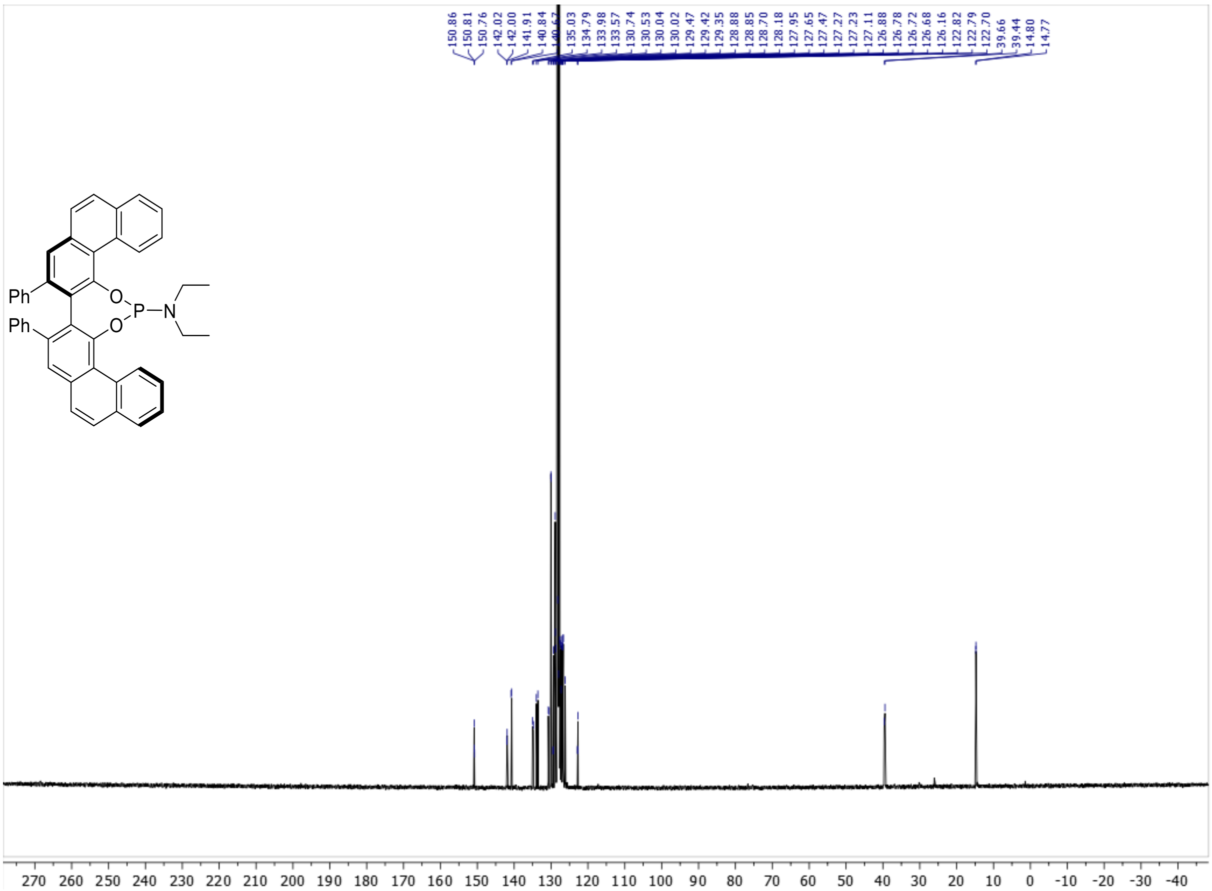

150.86
150.81
150.76
142.02
142.00
141.91
140.84
135.03
134.79
133.98
133.57
130.74
130.53
130.04
130.02
129.47
129.42
129.35
128.88
128.85
128.70
128.18
127.95
127.65
127.47
127.27
127.23
127.11
126.88
126.78
126.72
126.68
126.16
122.82
122.79
122.70
39.66
39.44
14.80
14.77
270 260 250 240 230 220 210 200 190 180 170 160 150 140 130 120 110 100 90 80 70 60 50 40 30 20 10 0 -10 -20 -30 -40
Ph
Ph
O
O
P
N

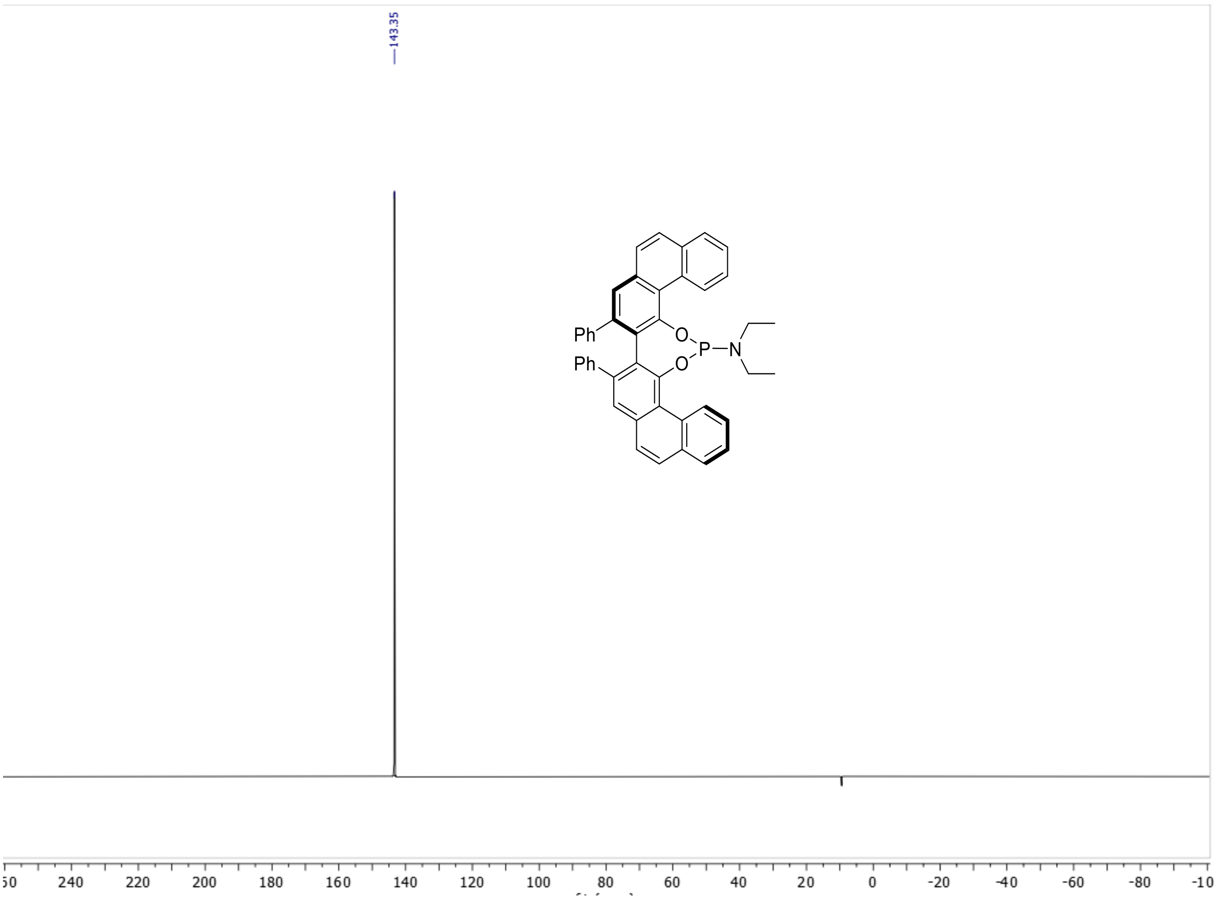
143.35
Ph
Ph
O
O
P
N
50
240
220
200
180
160
140
120
100
80
60
40
20
0
-20
-40
-60
-80
-10

SI-L1

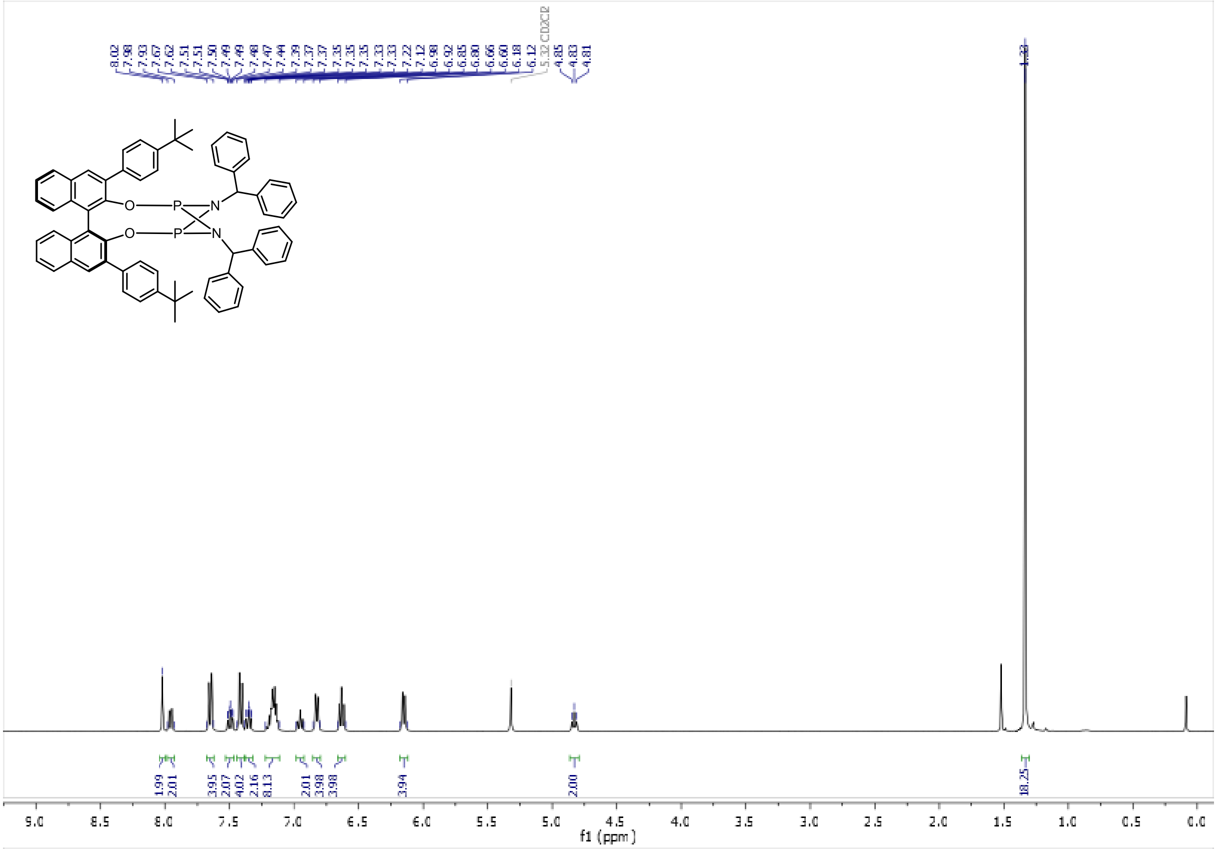

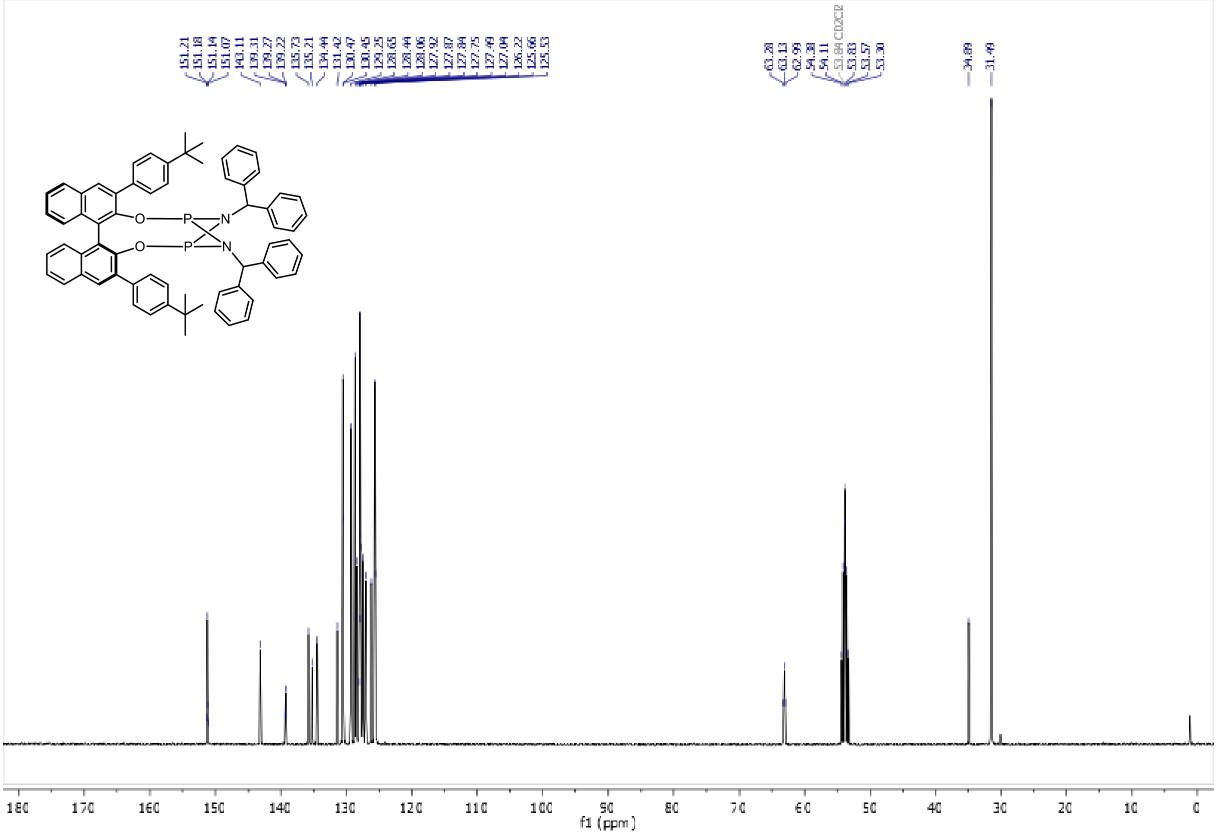

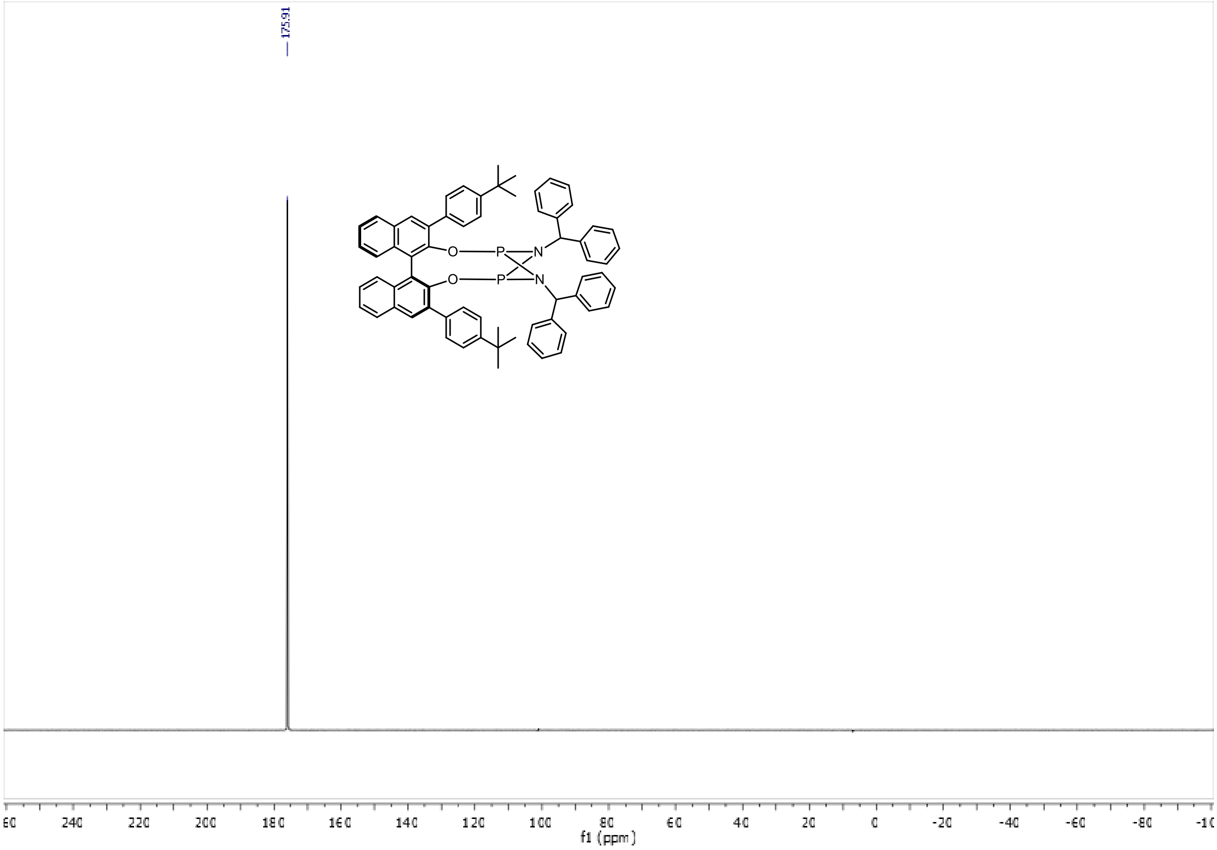

175.91
O
P
N
O
P
N
f1 (ppm)

SI-L2

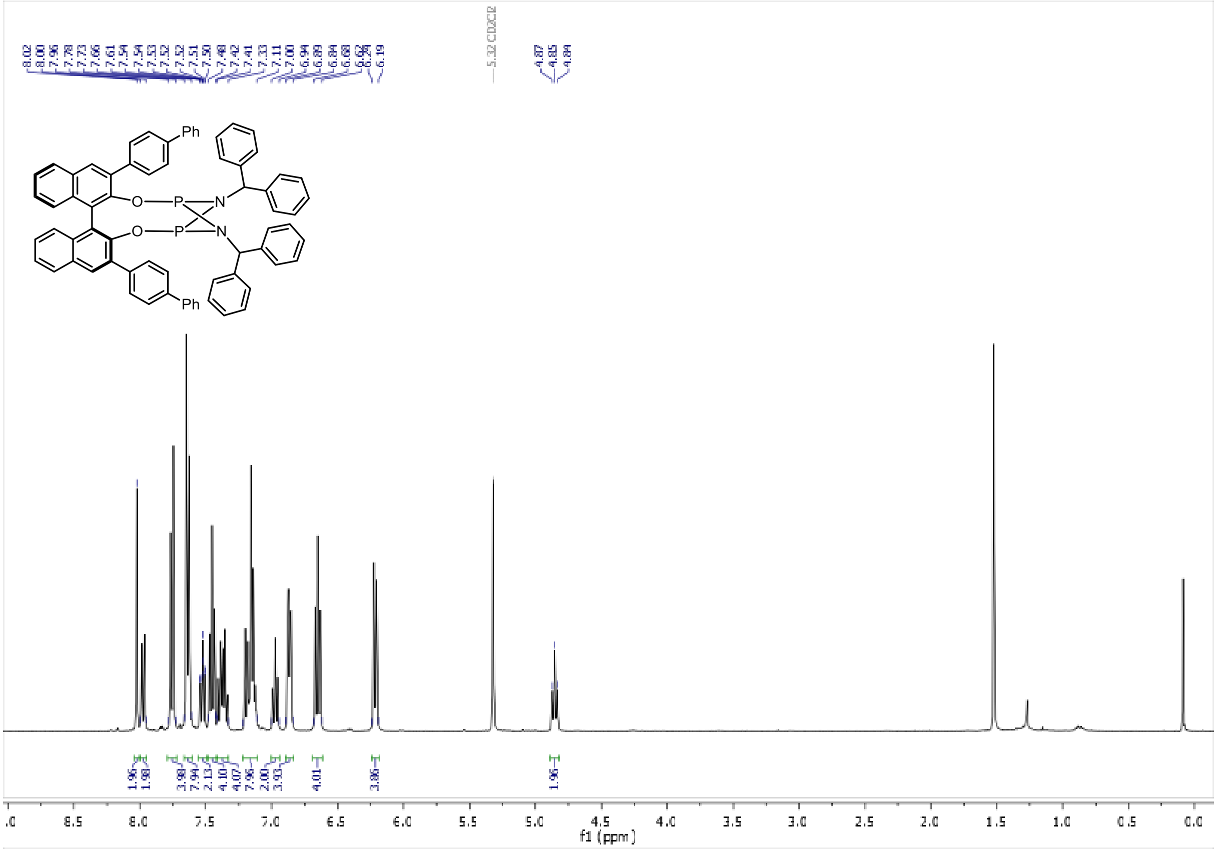

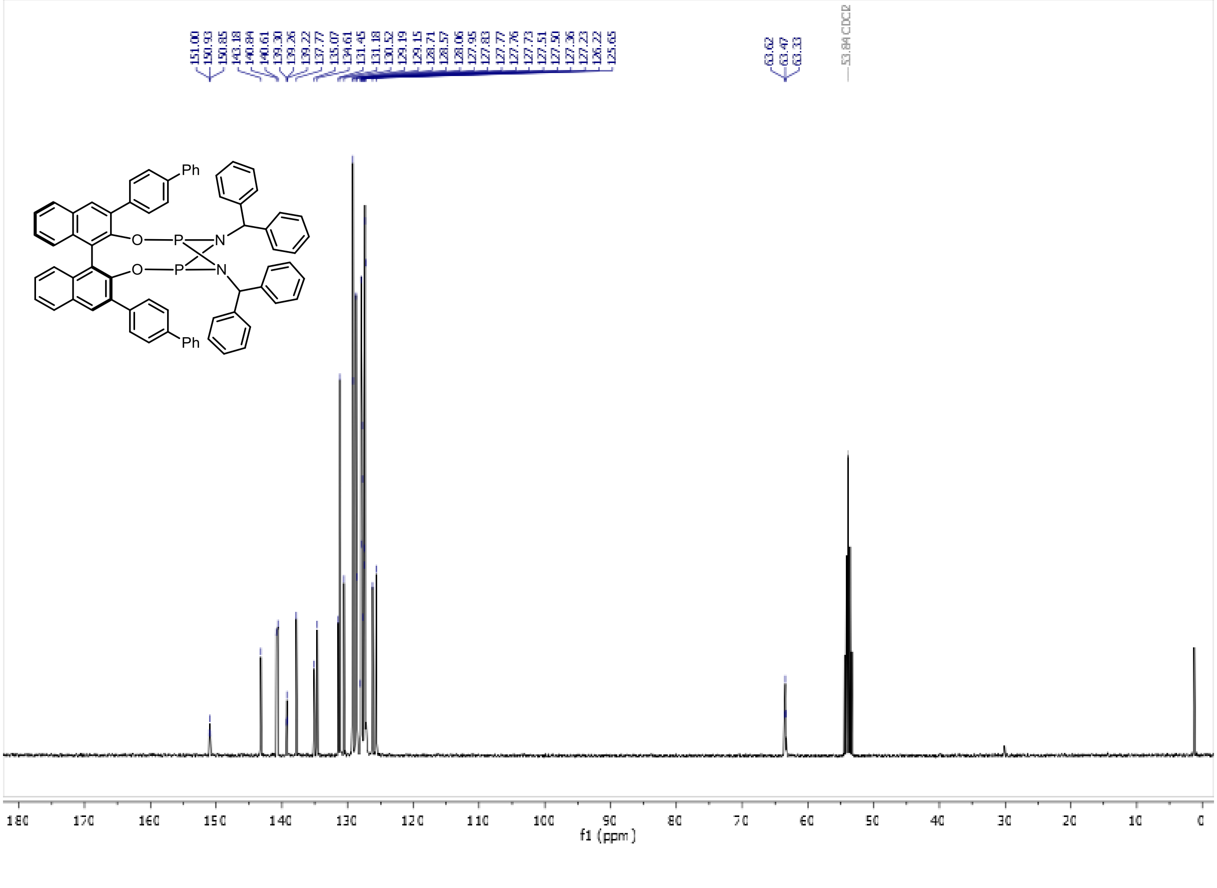

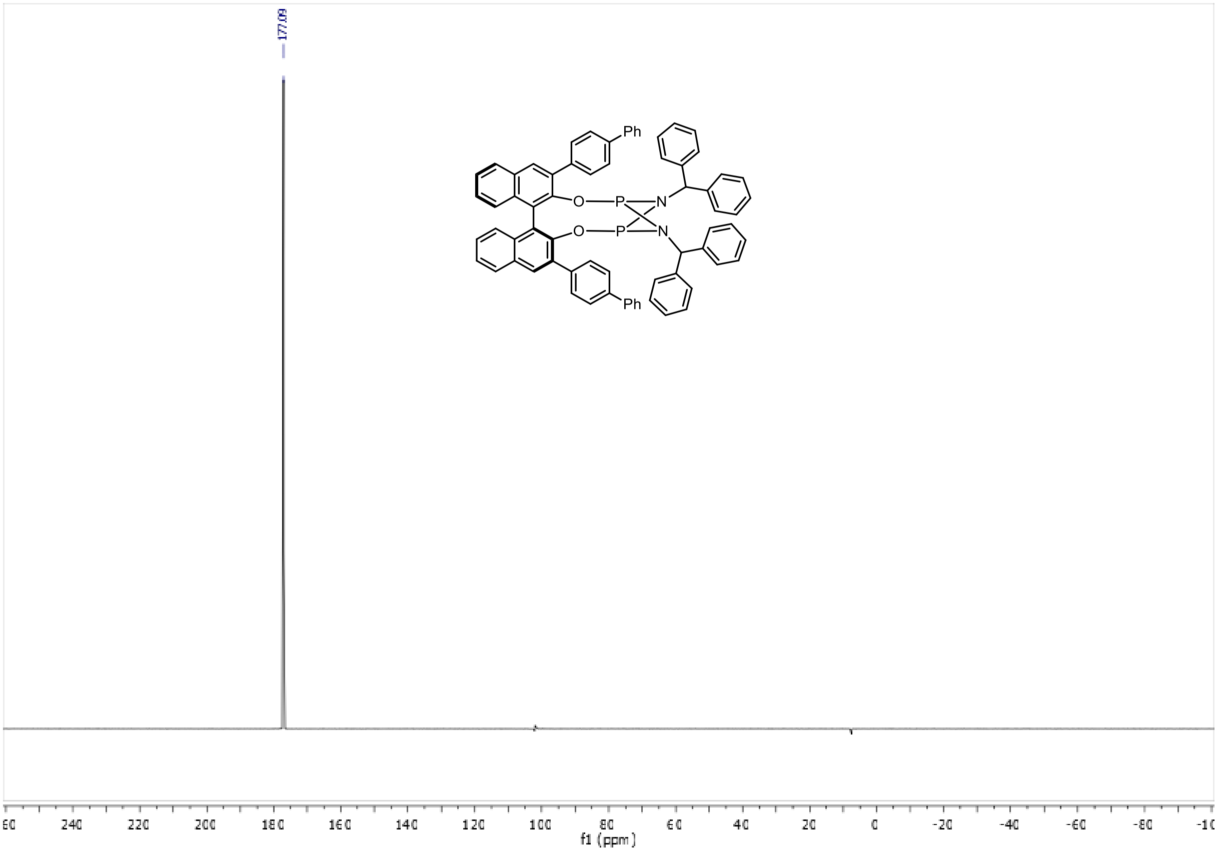
177.09
Ph
O
P
N
O
P
N
Ph
f1 (ppm)

SI-L3

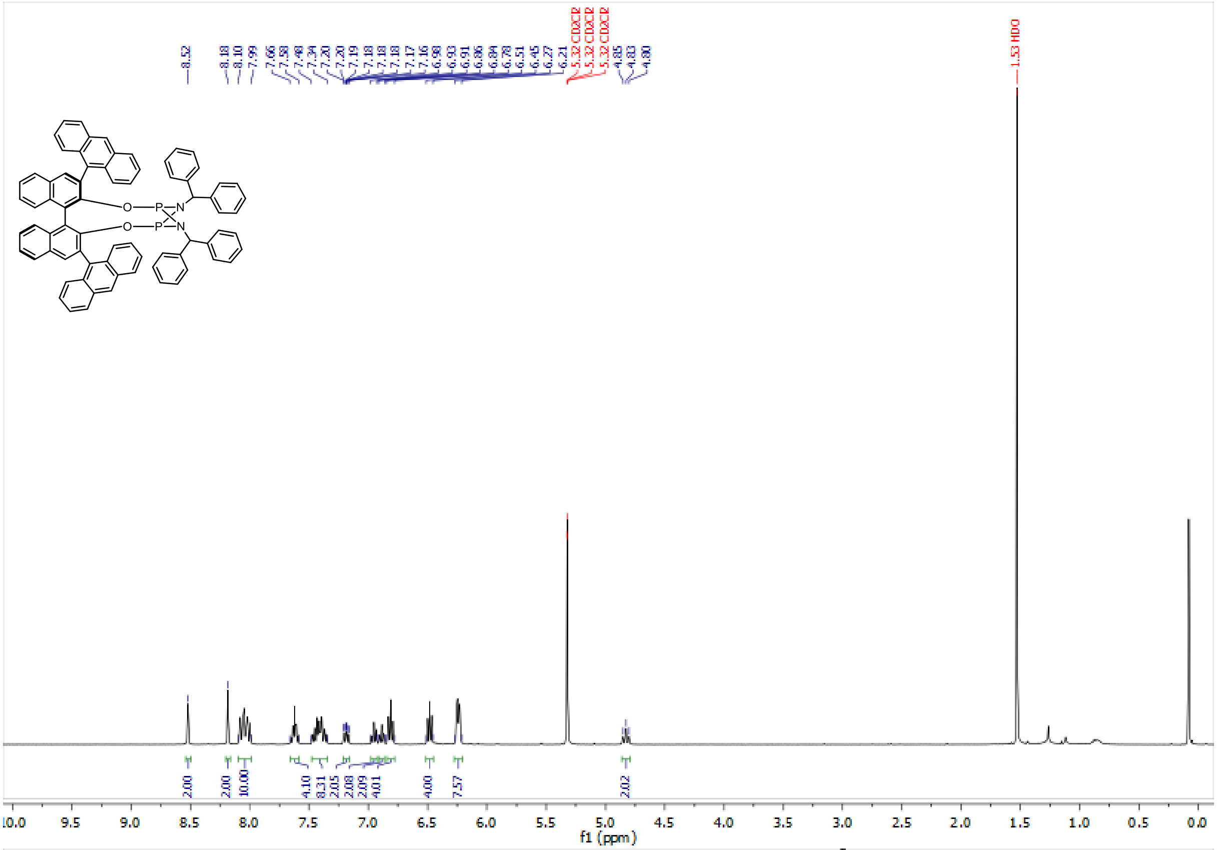

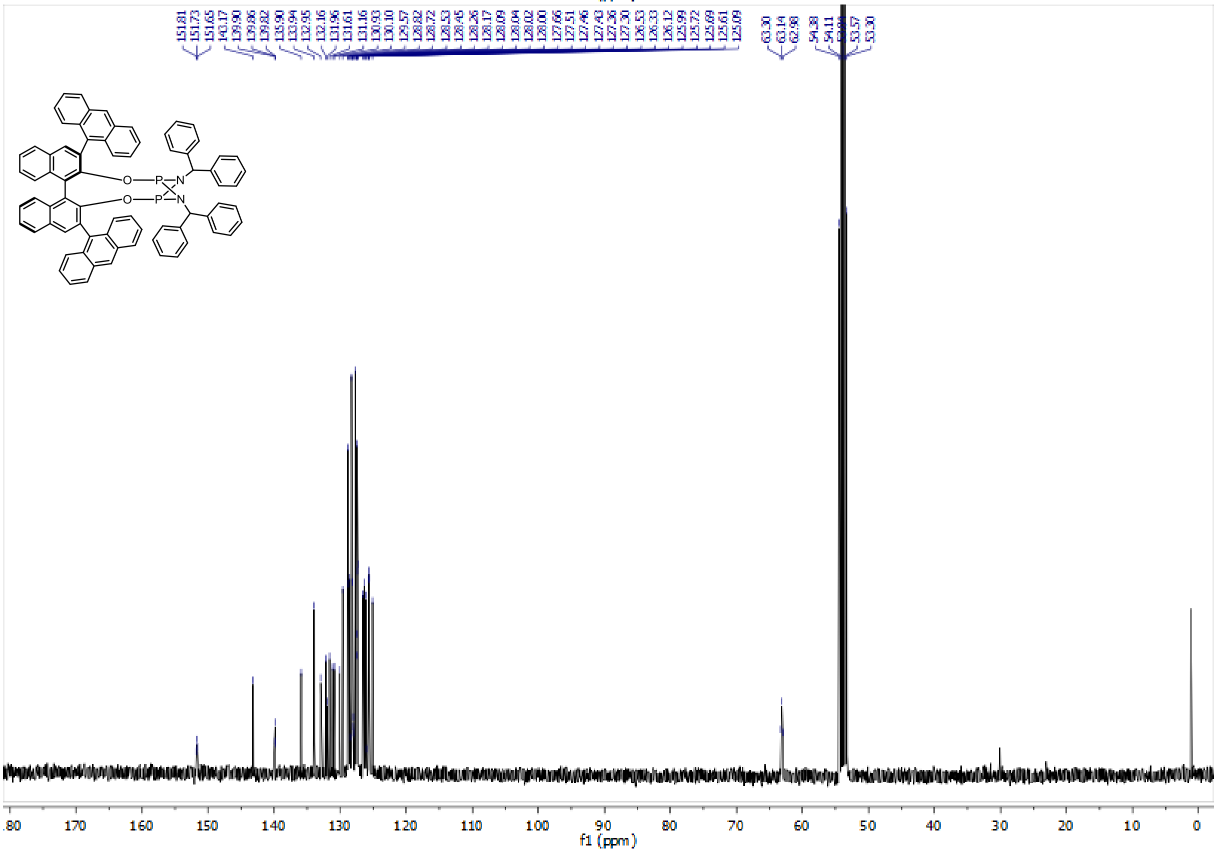

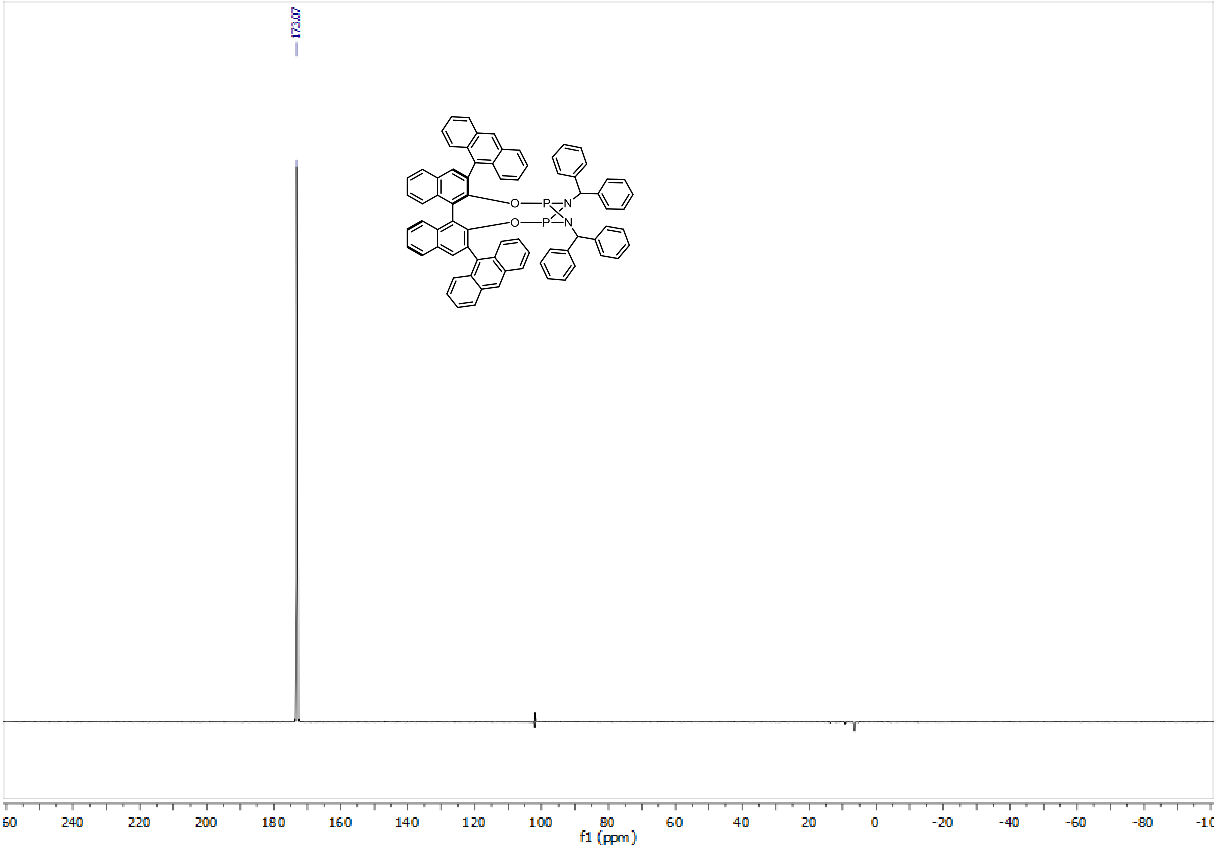
173.07
O
P
N
O
P
N
260
240
220
200
180
160
140
120
100
80
60
40
20
0
-20
-40
-60
-80
-10
f1 (ppm)

SI-L4

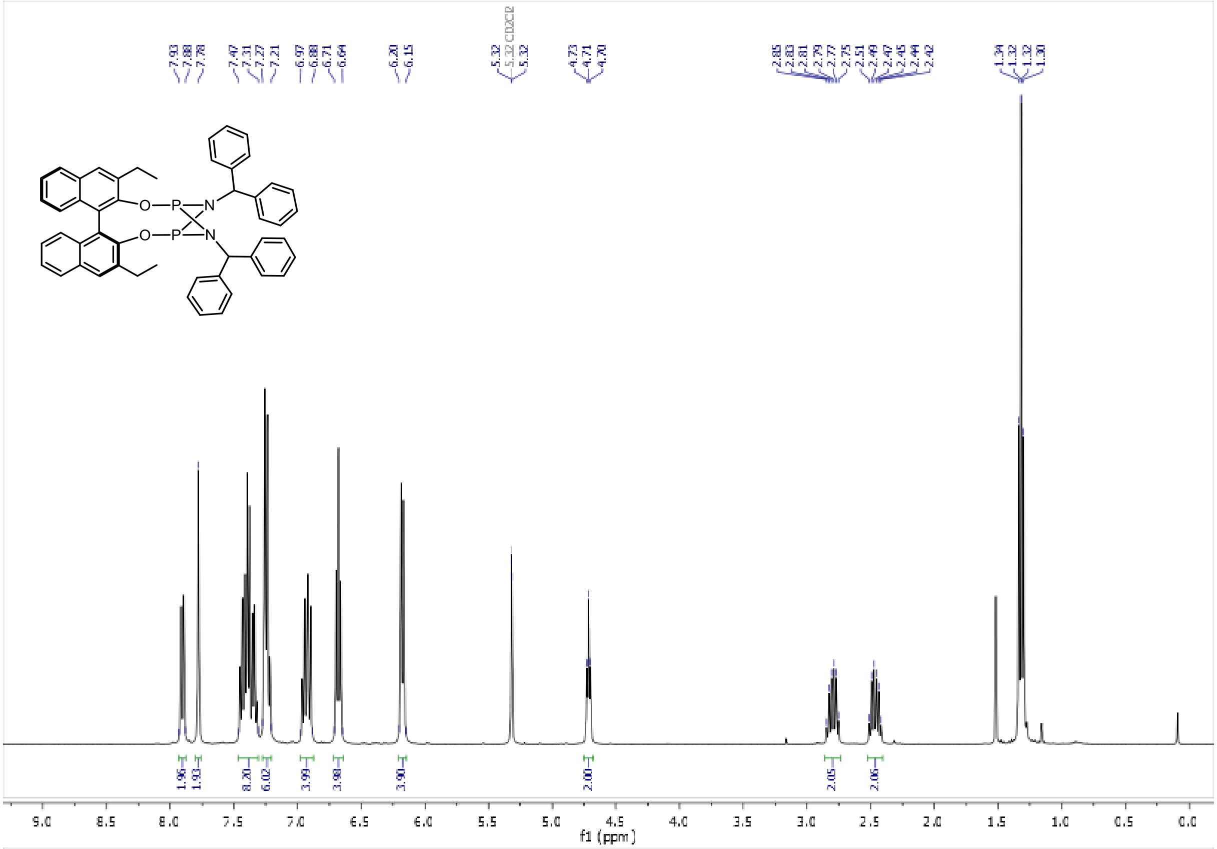
7.93
7.88
7.78
7.47
7.31
7.27
7.21
6.97
6.88
6.71
6.64
6.20
6.15
5.32
5.32 CD2Cl2
5.32
4.73
4.71
4.70
2.85
2.83
2.81
2.79
2.77
2.75
2.51
2.49
2.47
2.45
2.44
2.42
1.34
1.32
1.32
1.30
O
P
N
1.96
1.93
8.20
6.02
3.99
3.98
3.90
2.00
2.05
2.06
f1 (ppm)

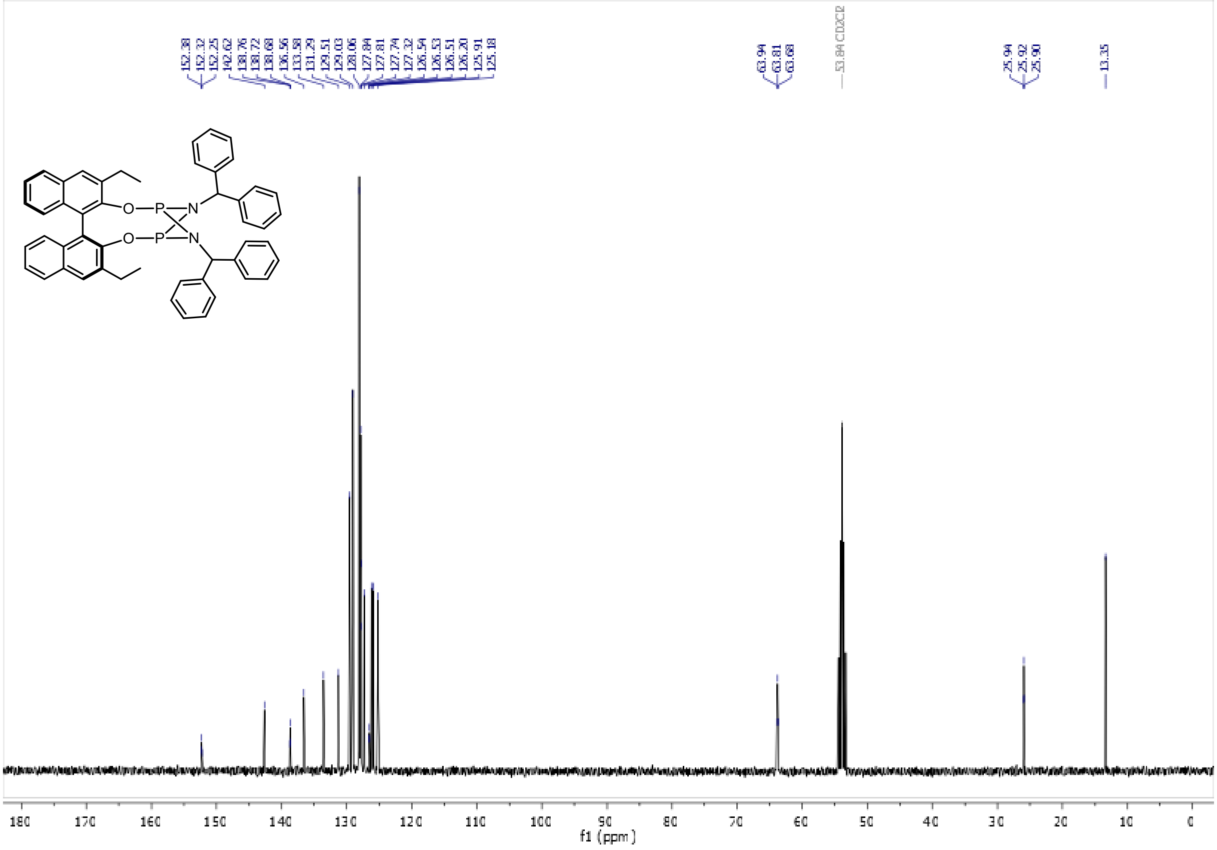
152.38
152.32
152.25
142.62
138.76
138.72
138.68
136.56
133.58
131.29
129.51
129.03
128.06
127.84
127.81
127.74
127.32
126.54
126.53
126.51
126.20
125.91
125.18
63.94
63.81
63.68
53.84 CD2Cl2
25.94
25.92
25.90
13.35
O
P
N
f1 (ppm)

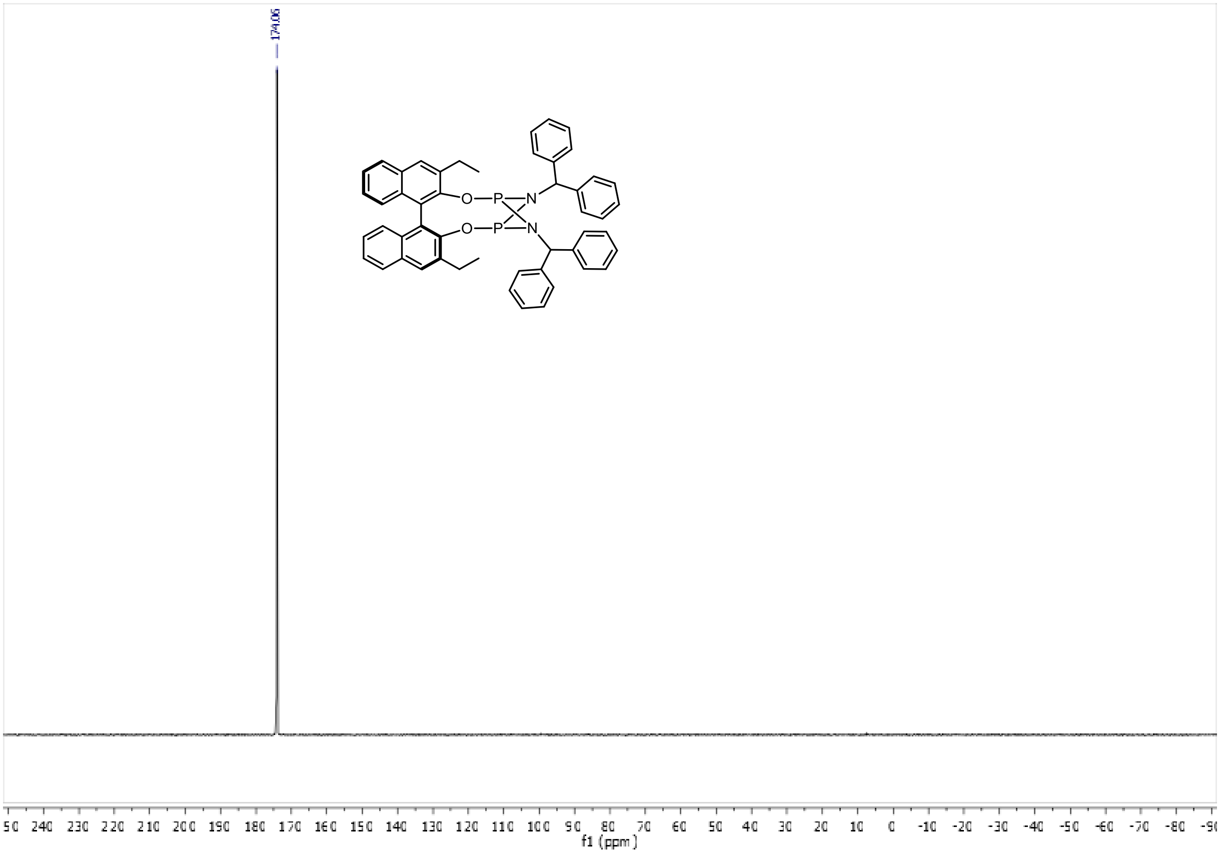
174.06
O
P
N
O
P
N
f1 (ppm)

SI-L5

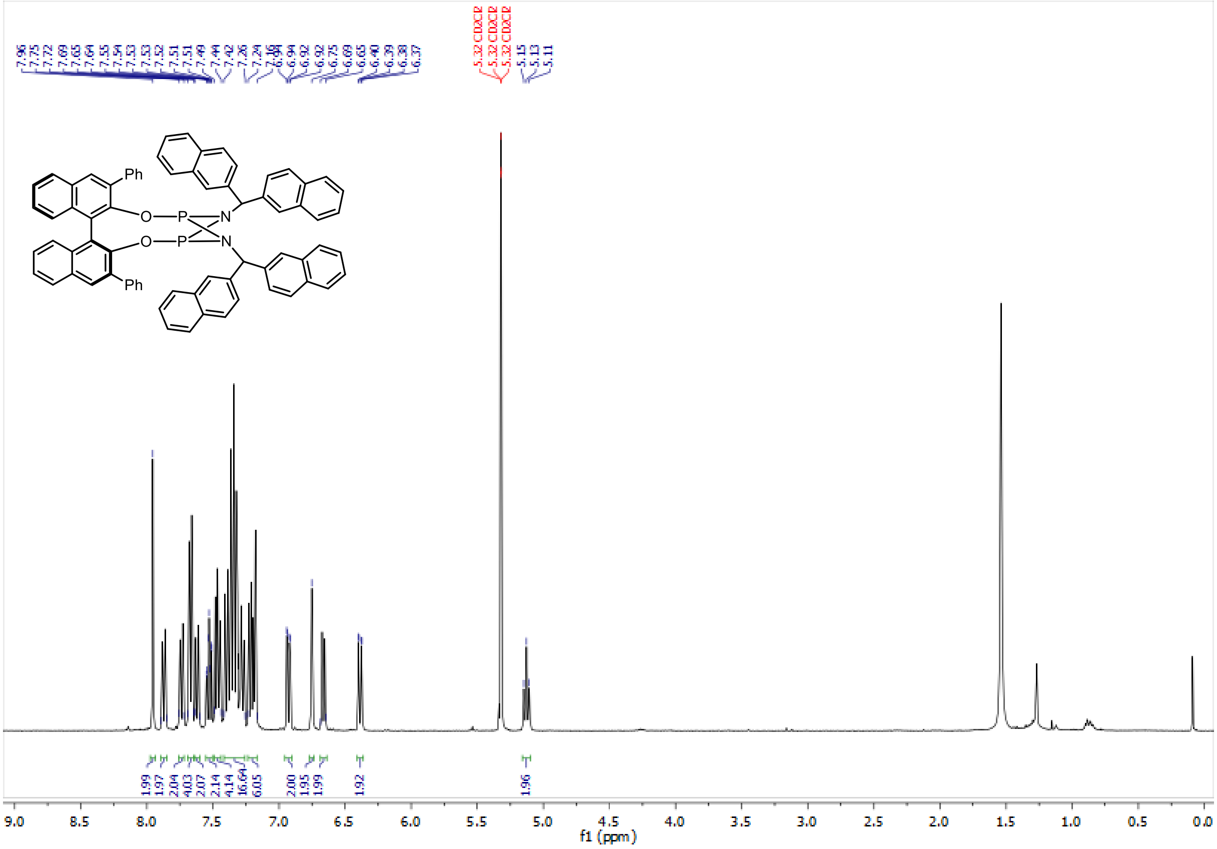

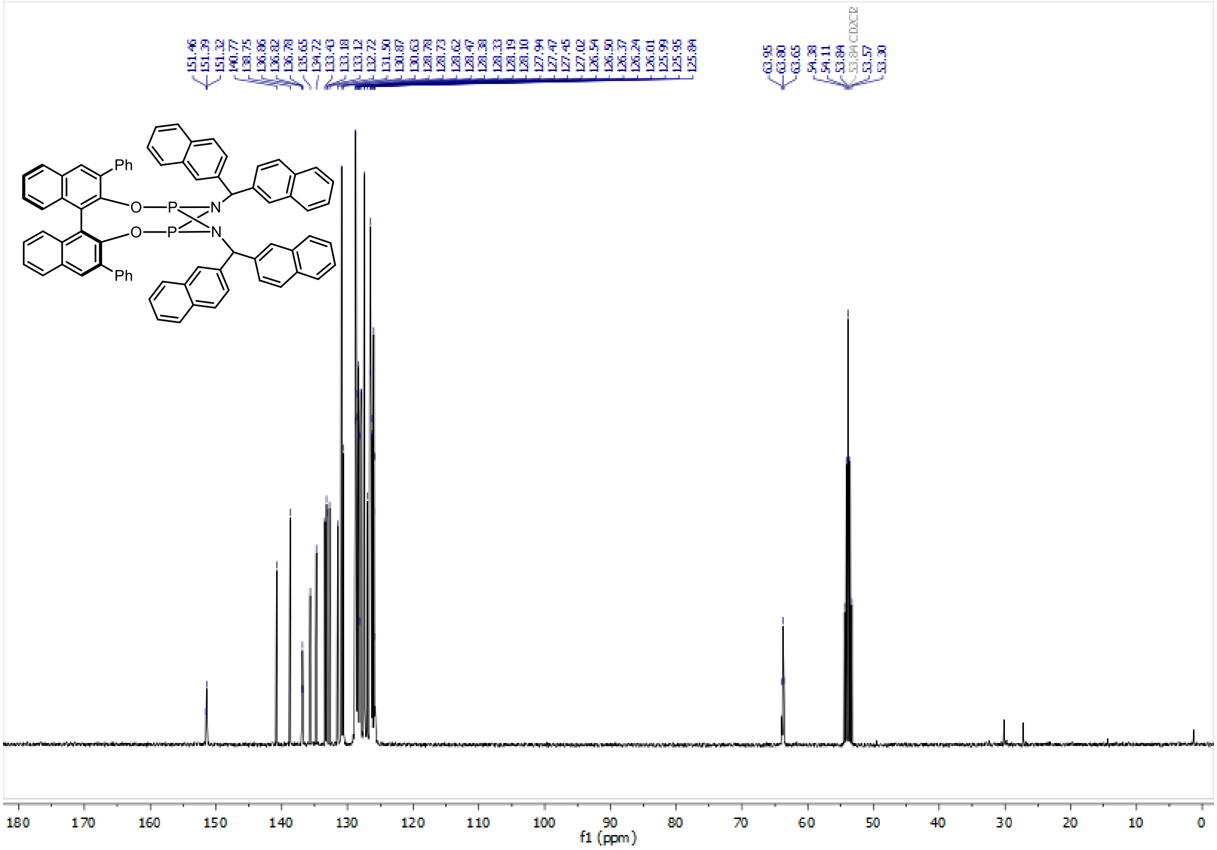

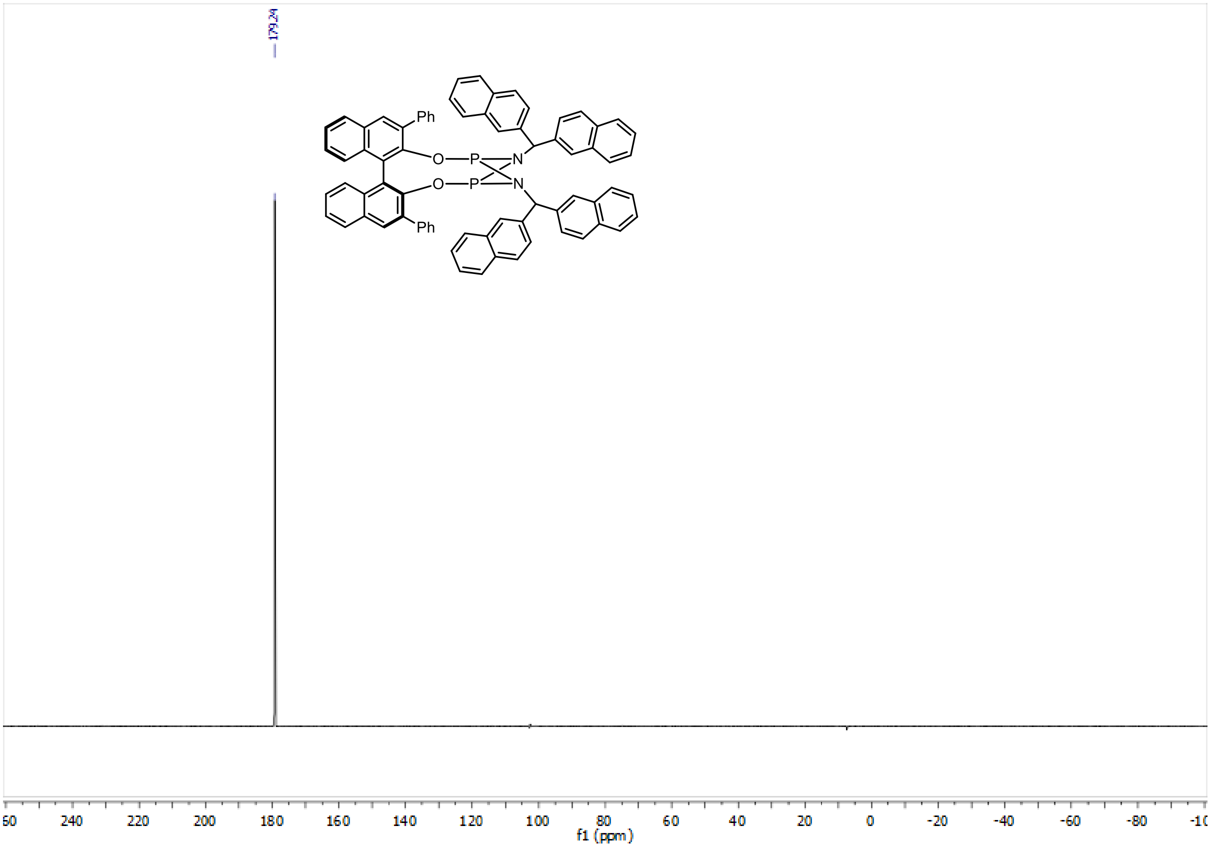
179.24
Ph
O
P
N
O
P
N
Ph
f1 (ppm)

SI-L6

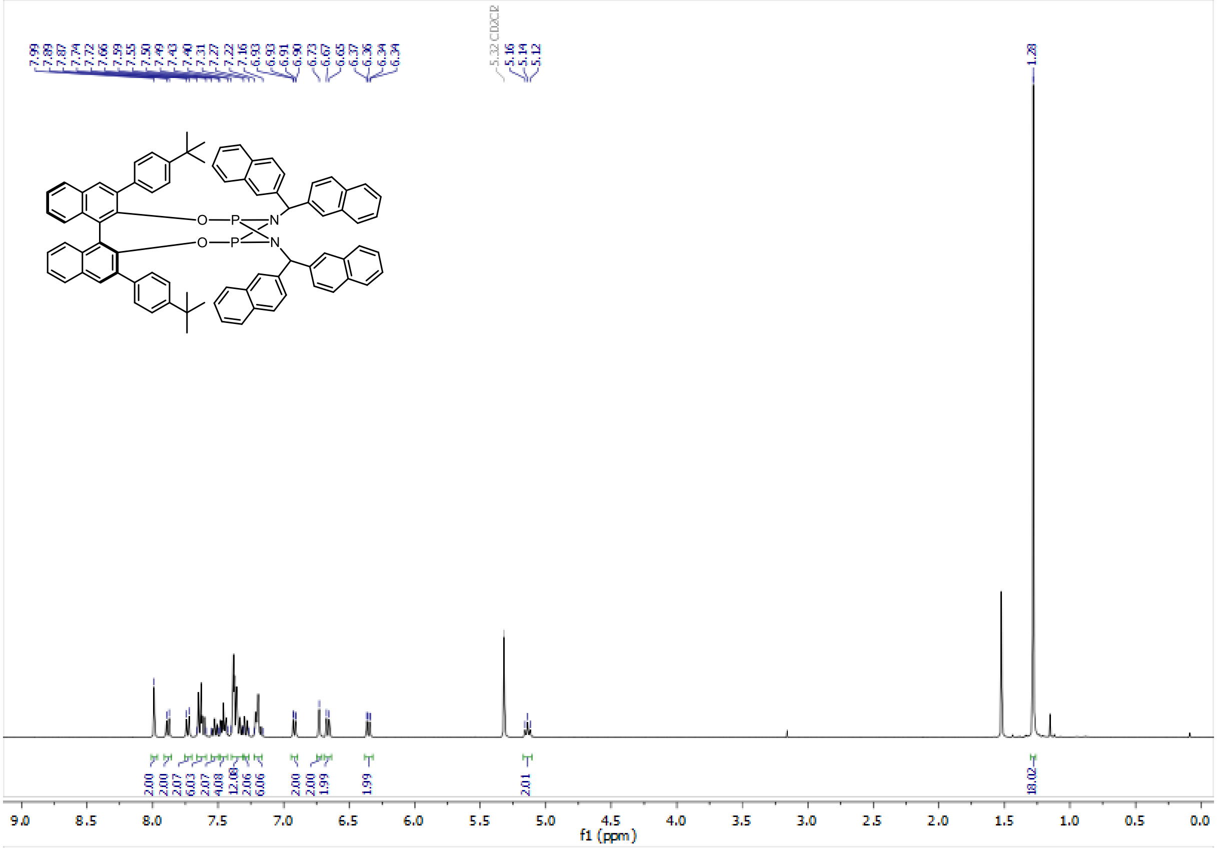

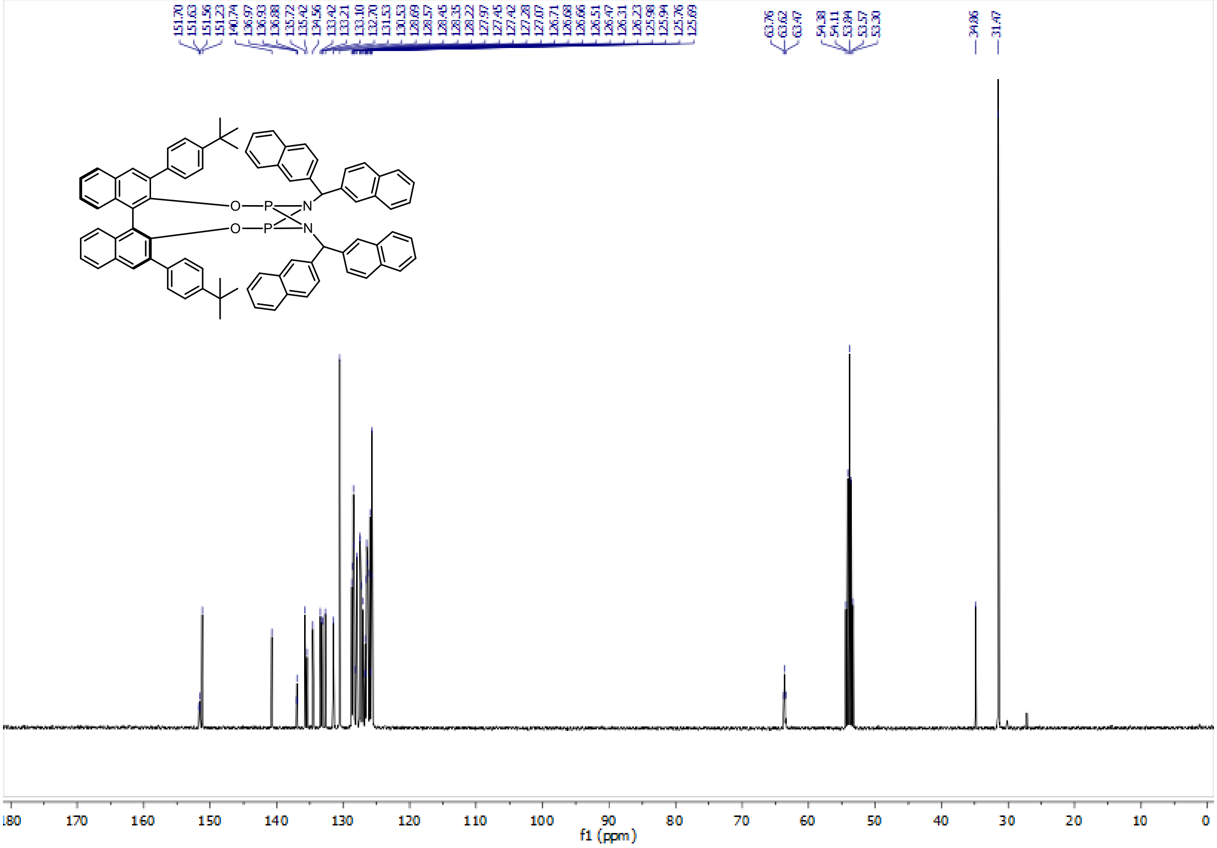

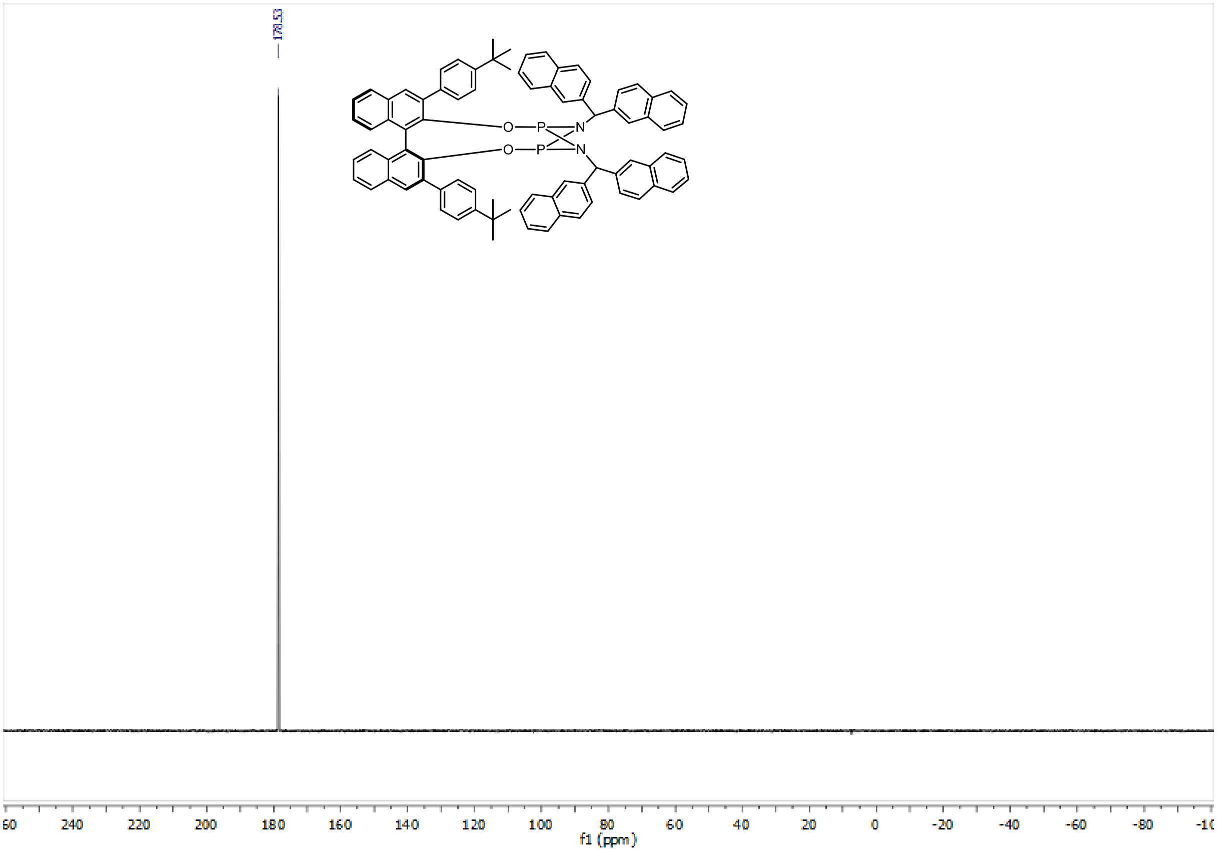

178.53
O
P
N
O
P
N
60
240
220
200
180
160
140
120
100
80
60
40
20
0
-20
-40
-60
-80
f1 (ppm)

SI-L7

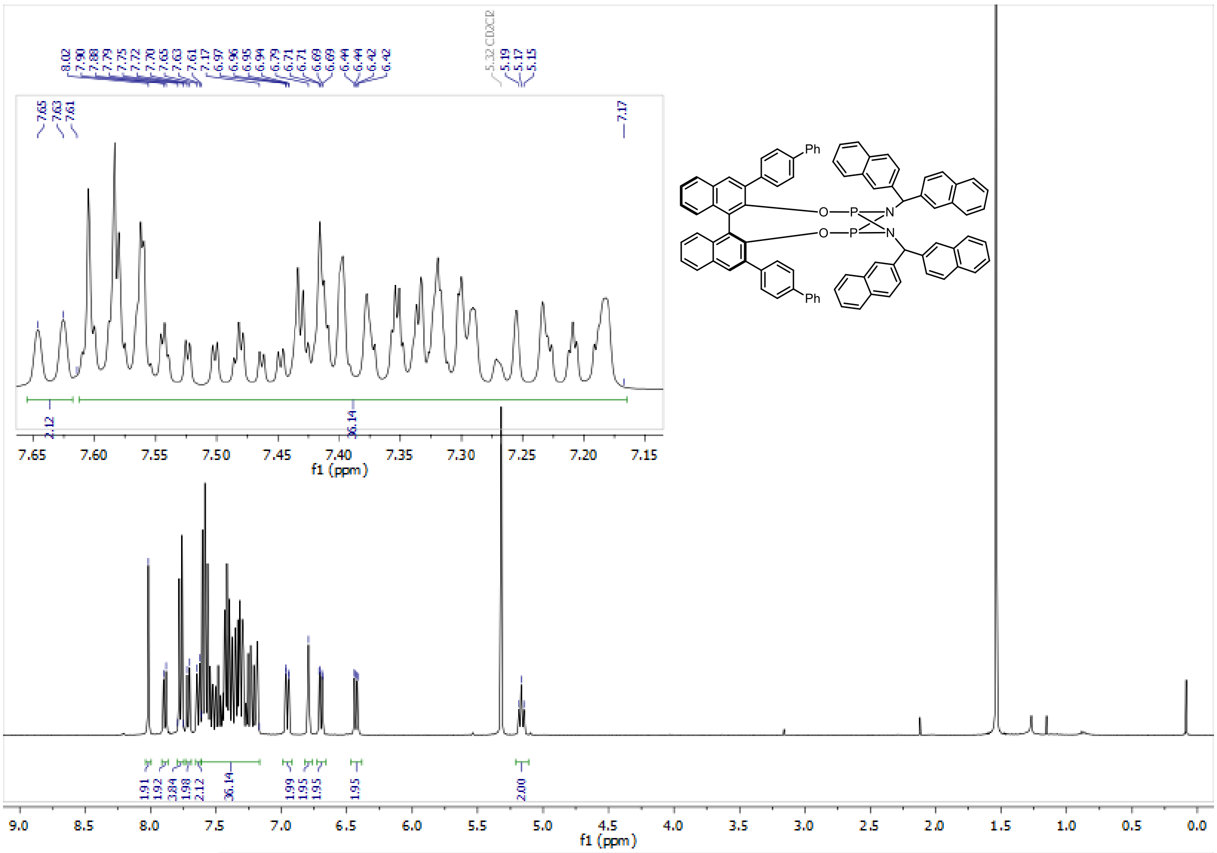

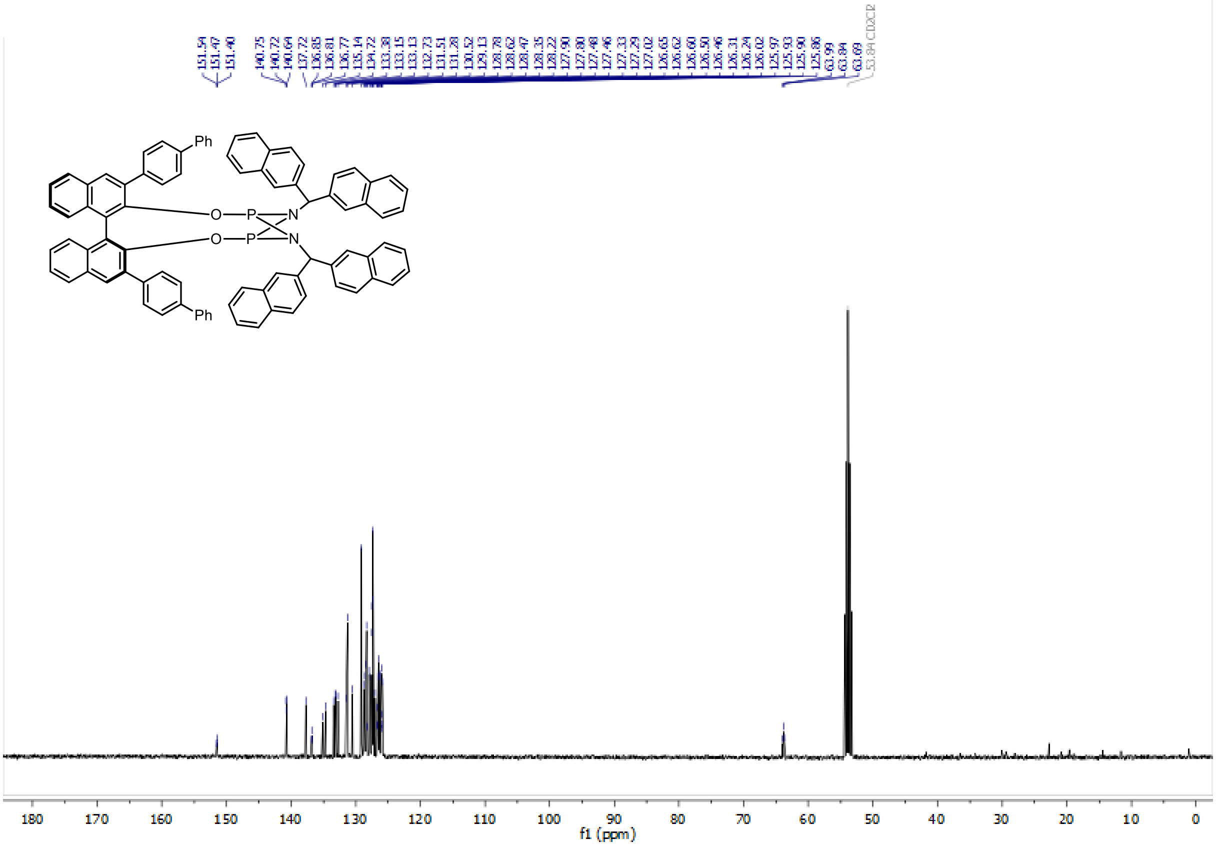

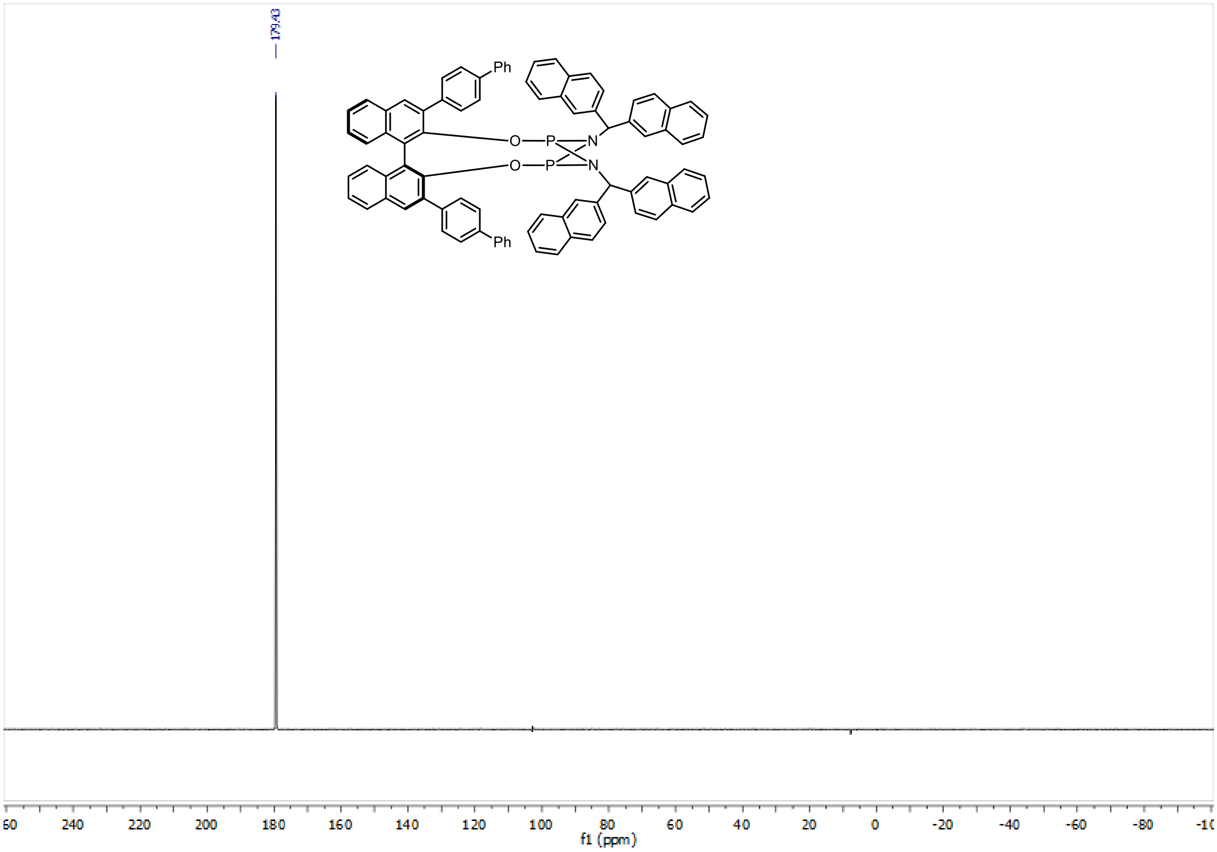
179.43
Ph
O
P
N
O
P
N
Ph
f1 (ppm)

SI-L8

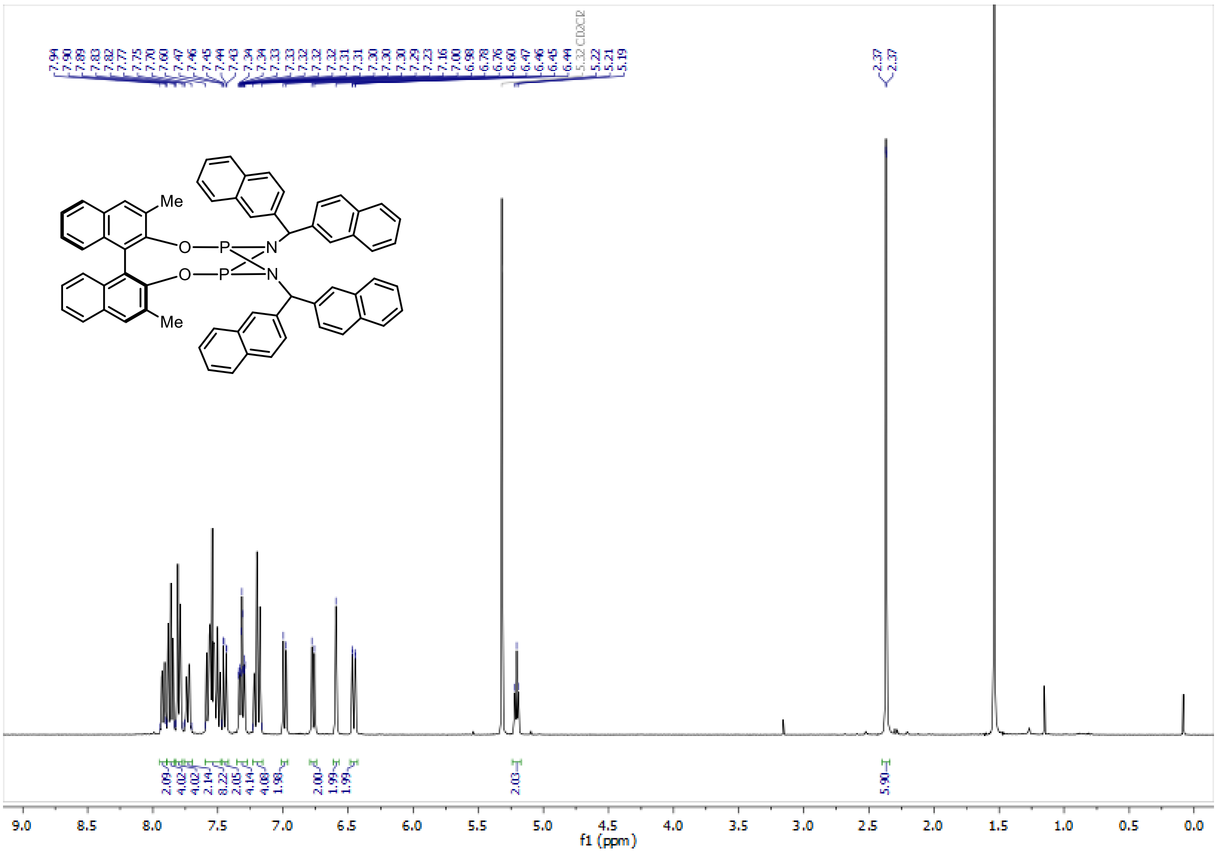

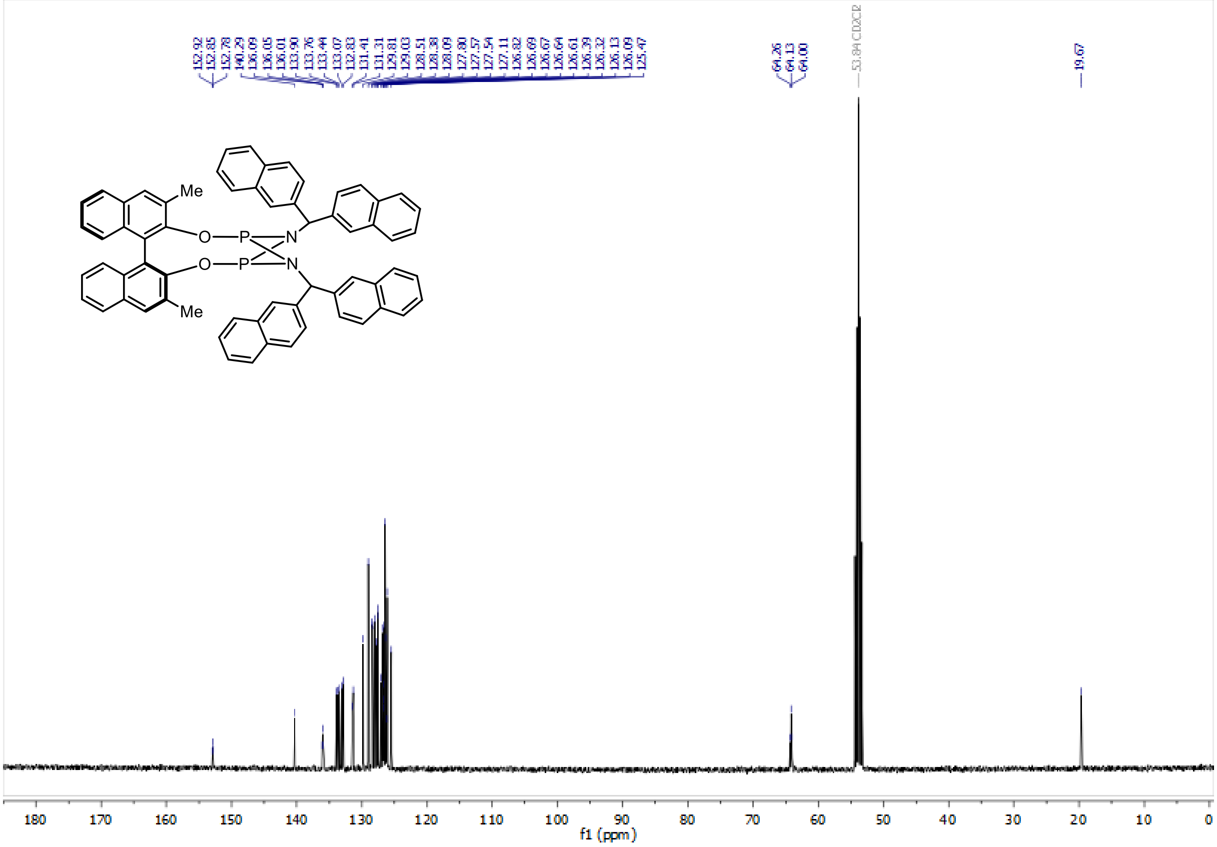

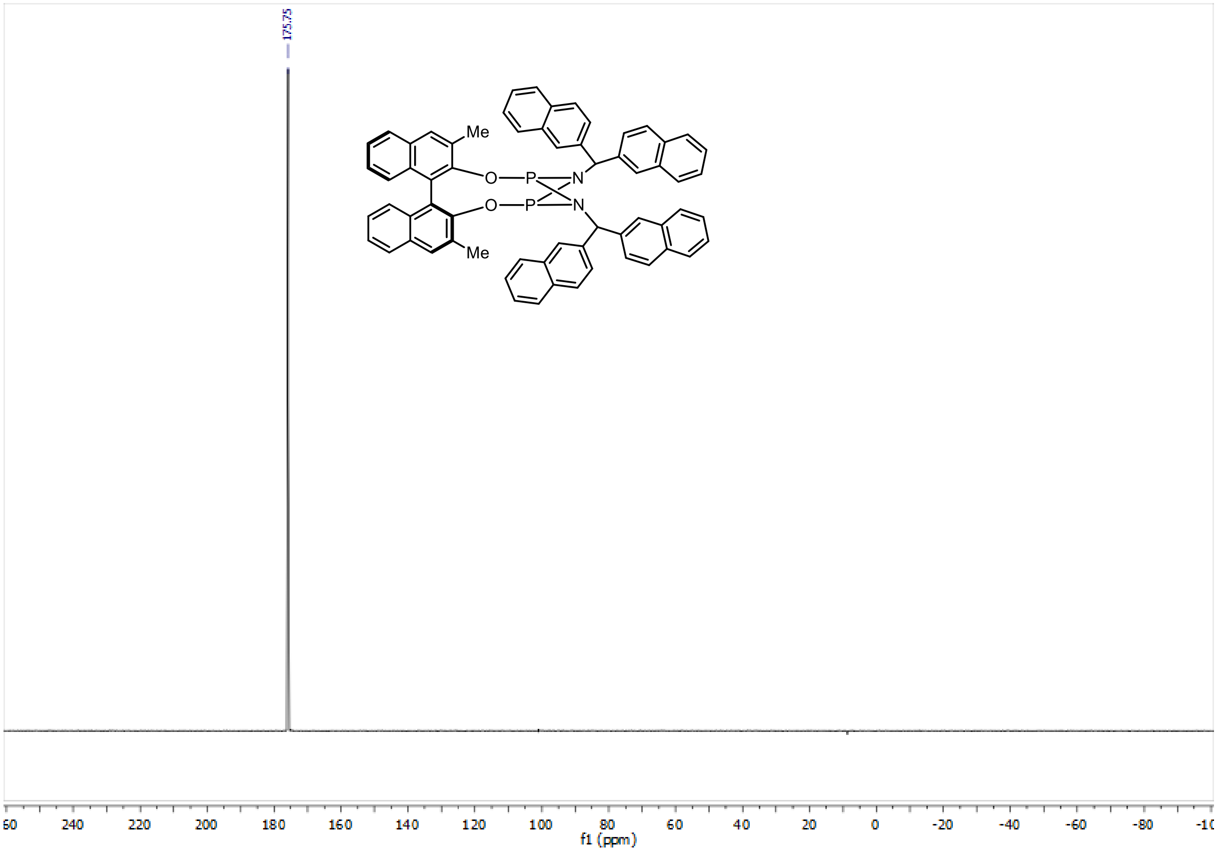
175.75
Me
O
P
N
O
P
N
Me
60
240
220
200
180
160
140
120
100
80
60
40
20
0
-20
-40
-60
-80
-10
f1 (ppm)

SI-L9

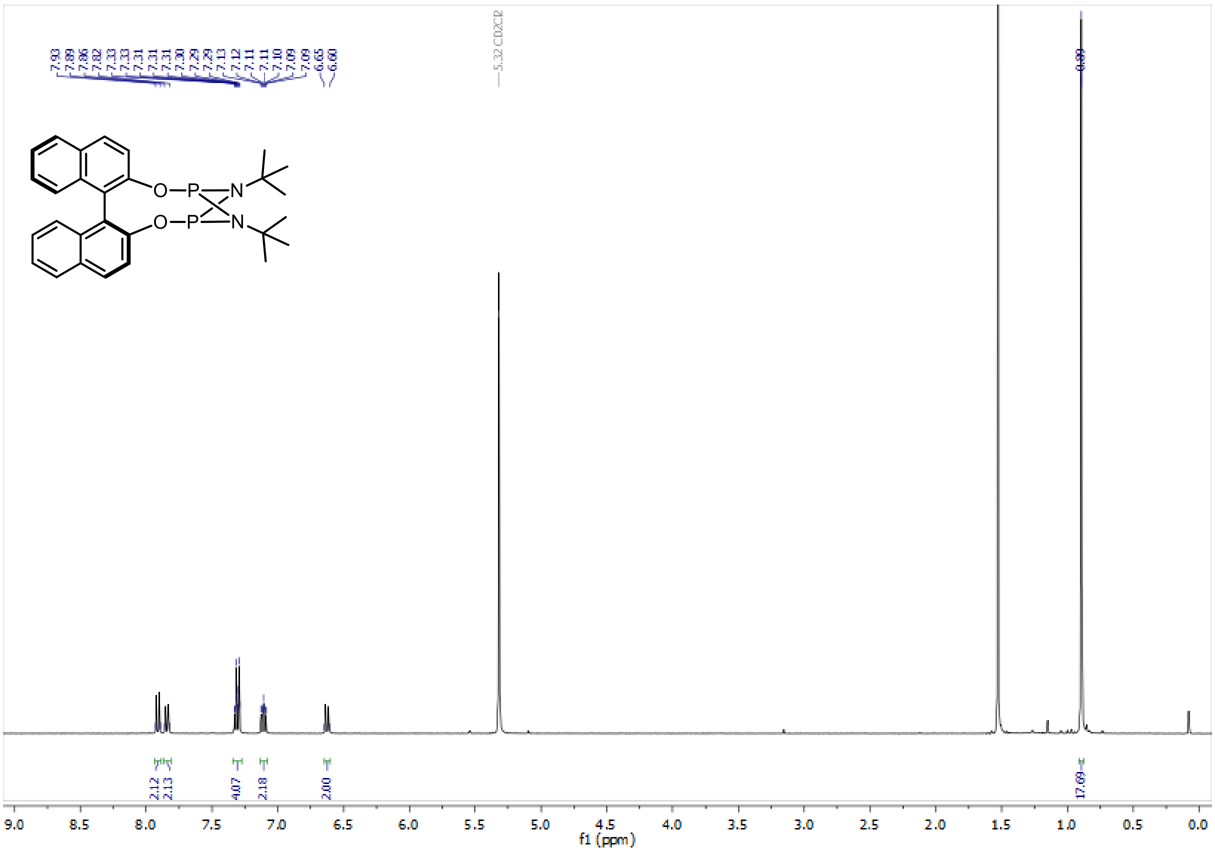
7.93
7.89
7.86
7.82
7.33
7.33
7.31
7.31
7.31
7.30
7.29
7.29
7.13
7.12
7.11
7.11
7.10
7.09
7.09
6.65
6.60
5.32 CD2Cl2
0.89
O
P
N
O
P
N
2.12
2.13
4.07
2.18
2.00
17.69
9.0
8.5
8.0
7.5
7.0
6.5
6.0
5.5
5.0
4.5
4.0
3.5
3.0
2.5
2.0
1.5
1.0
0.5
0.0
f1 (ppm)

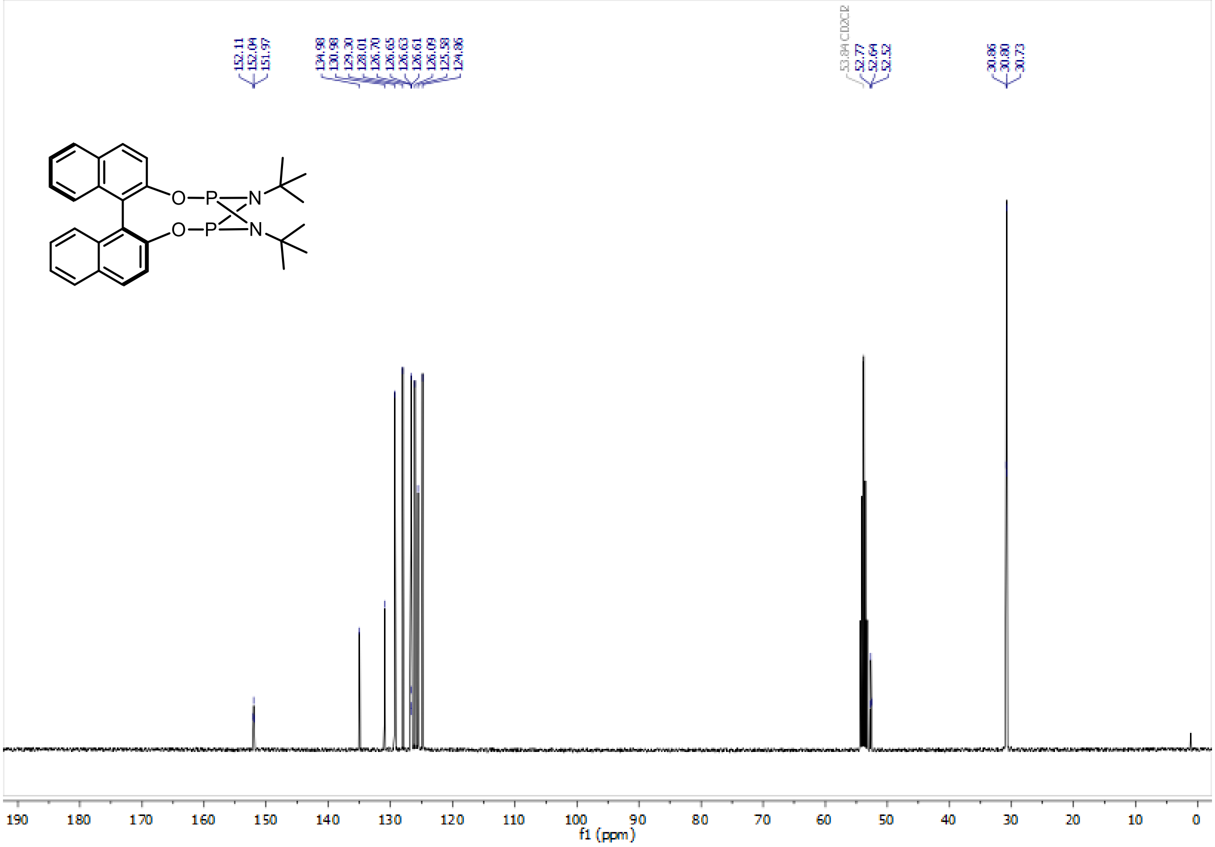
152.11
152.04
151.97
134.98
130.98
129.30
128.01
126.70
126.65
126.63
126.61
126.09
125.58
124.86
53.84 CD2Cl2
52.77
52.64
52.52
30.86
30.80
30.73
O
P
N
O
P
N
190
180
170
160
150
140
130
120
110
100
90
80
70
60
50
40
30
20
10
0
f1 (ppm)

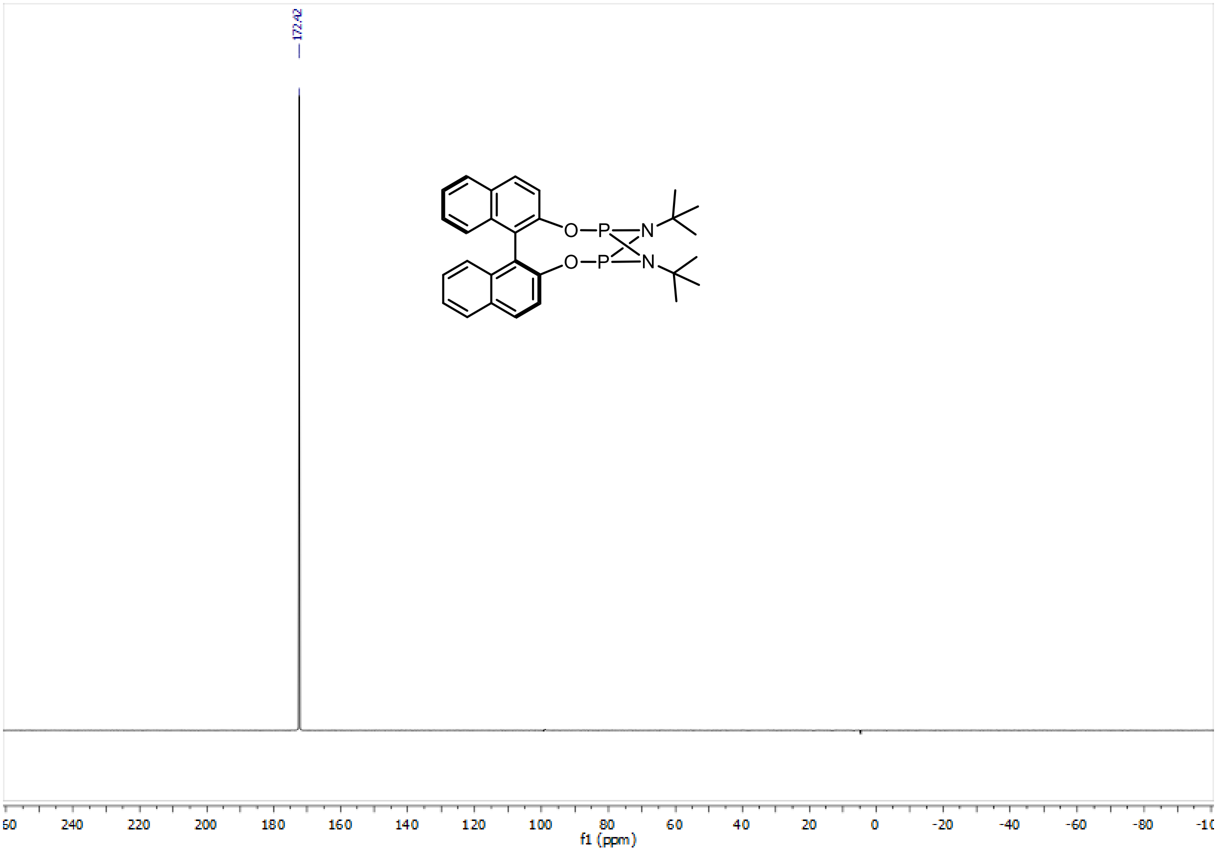

172.42
O
P
N
O
P
N
f1 (ppm)

SI-L10

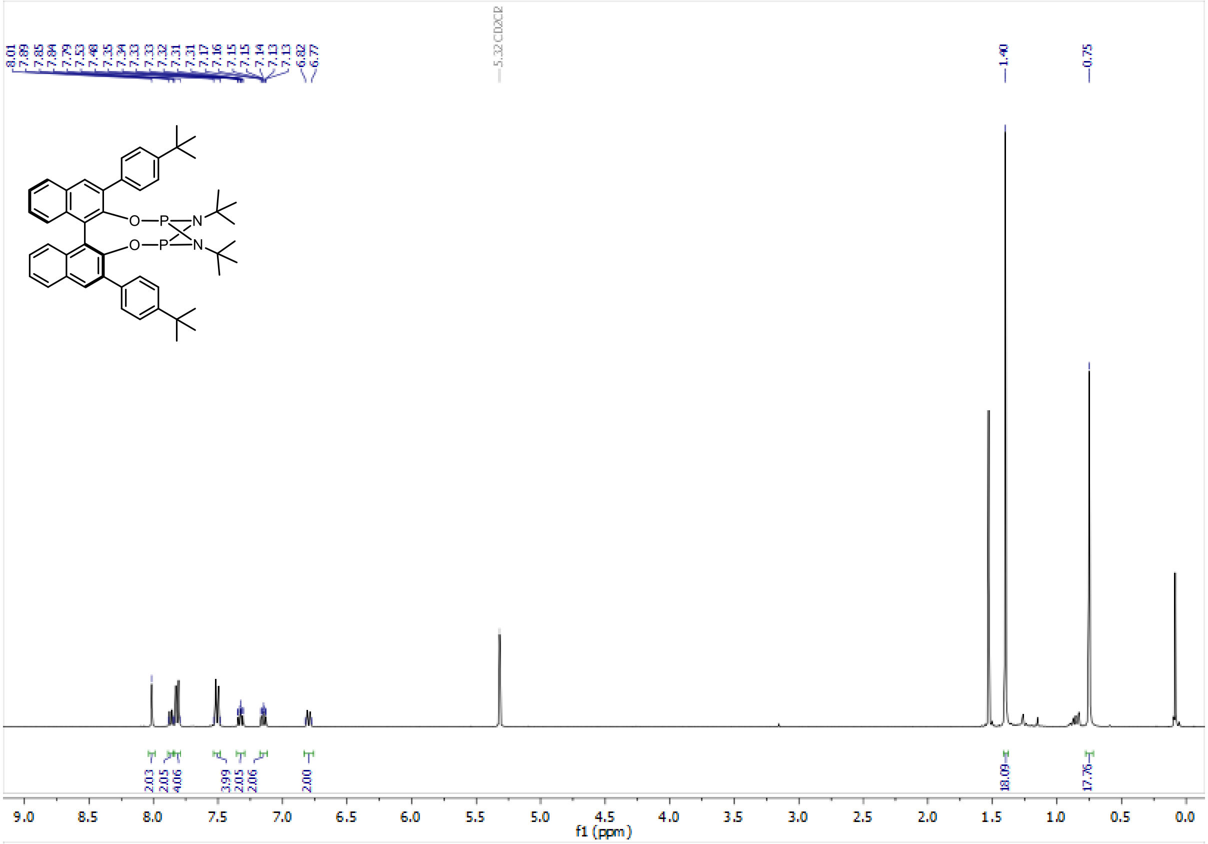
8.01
7.89
7.85
7.84
7.79
7.53
7.48
7.35
7.34
7.33
7.33
7.32
7.31
7.31
7.17
7.16
7.15
7.15
7.14
7.13
7.13
6.82
6.77
5.32 CD2Cl2
1.40
0.75
2.03
2.05
4.06
3.99
2.05
2.06
2.00
18.09
17.76
f1 (ppm)

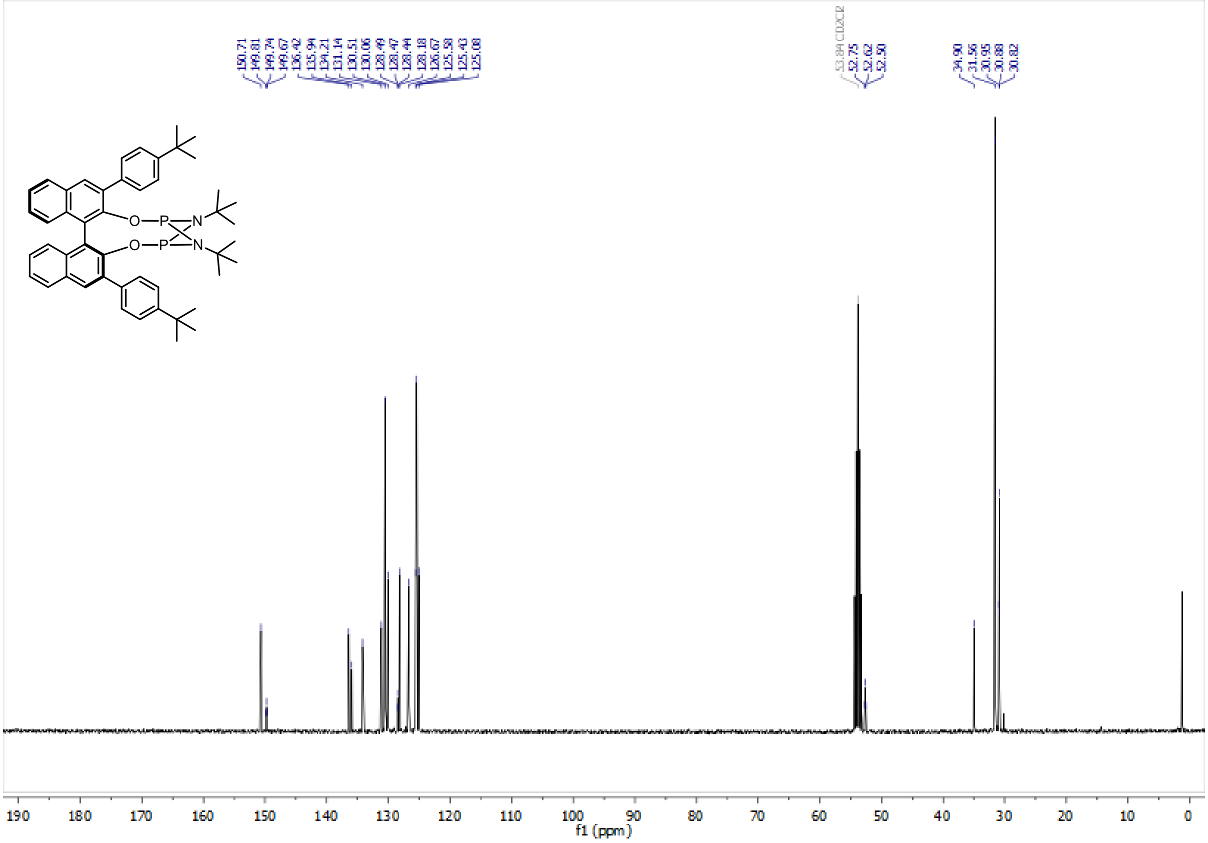
150.71
149.81
149.74
149.67
136.42
135.94
134.21
131.14
130.51
130.06
128.49
128.47
128.44
128.18
126.67
125.58
125.43
125.08
53.84 CD2Cl2
52.75
52.62
52.50
34.90
31.56
30.95
30.88
30.82
f1 (ppm)

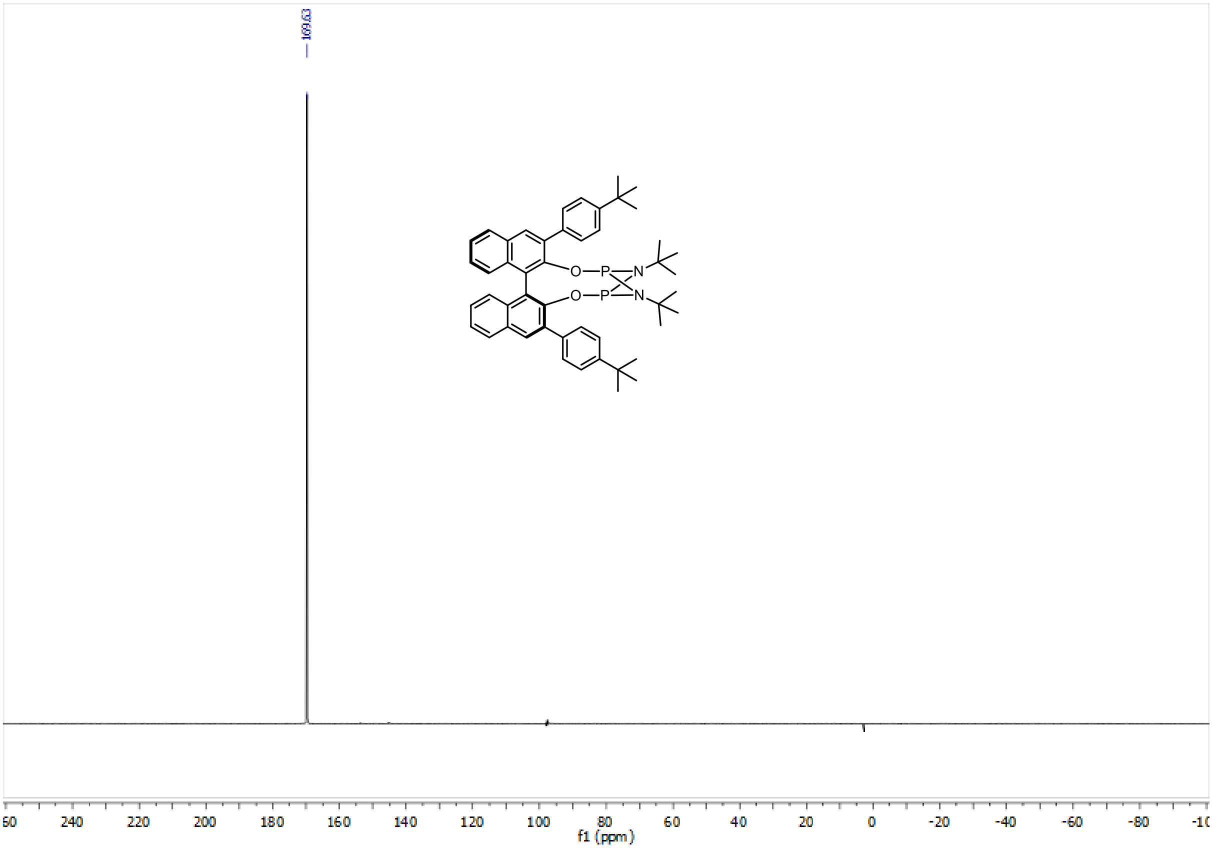
169.63
O
P
N
O
P
N
60
240
220
200
180
160
140
120
100
80
60
40
20
0
-20
-40
-60
-80
-10
f1 (ppm)

SI-L12

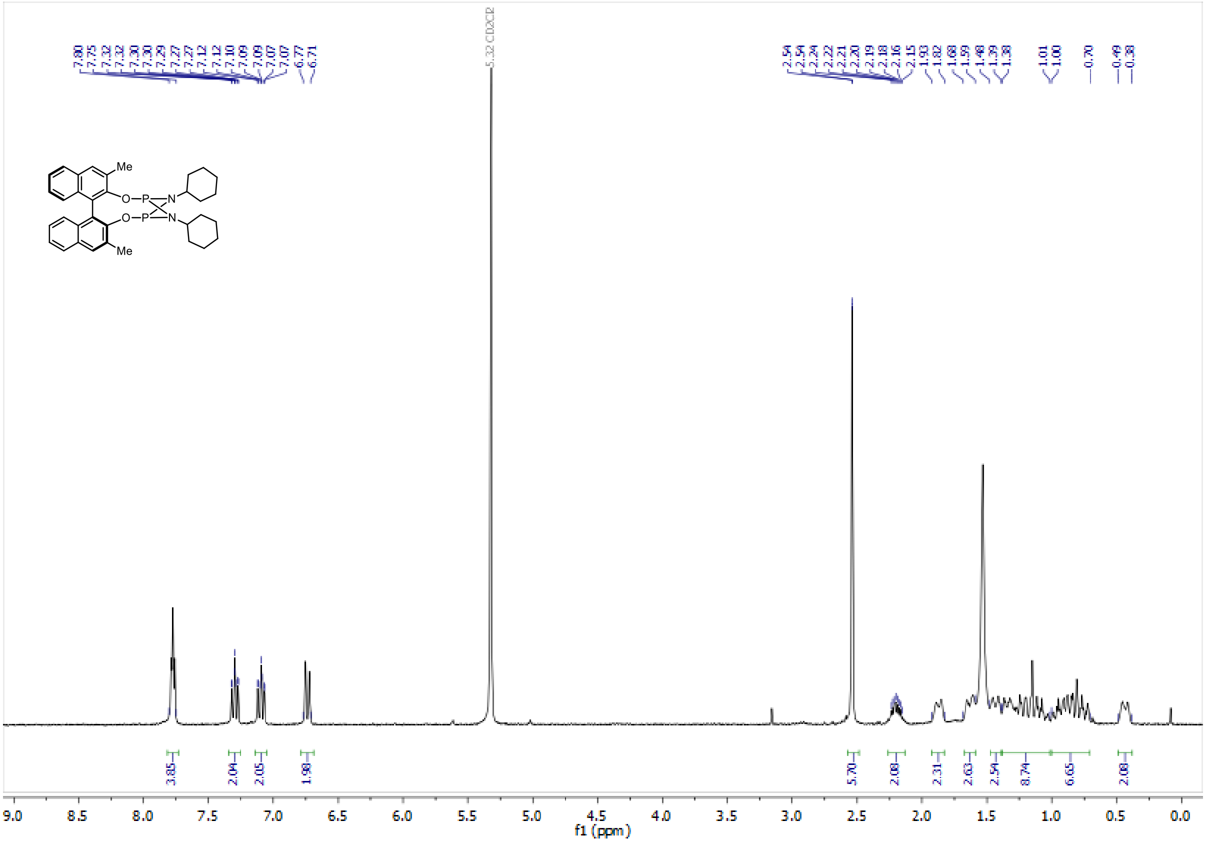

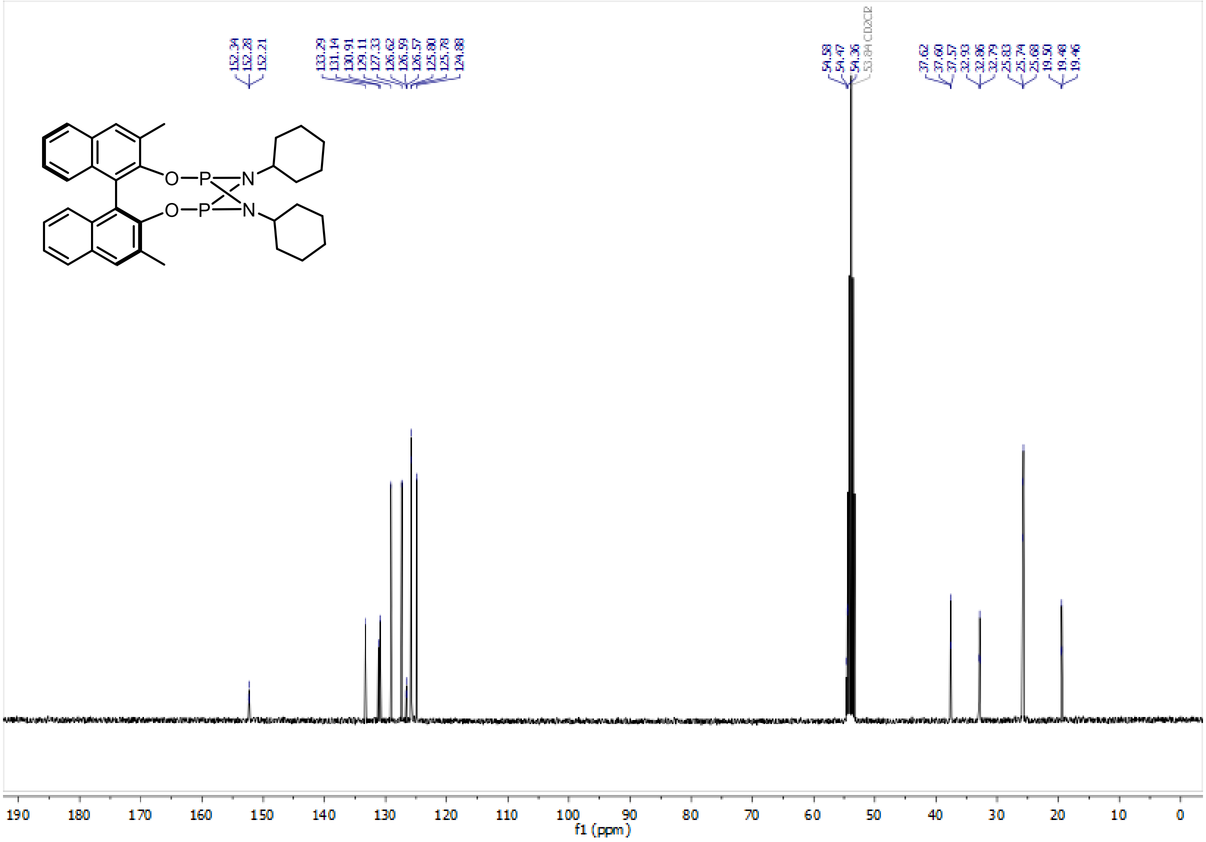

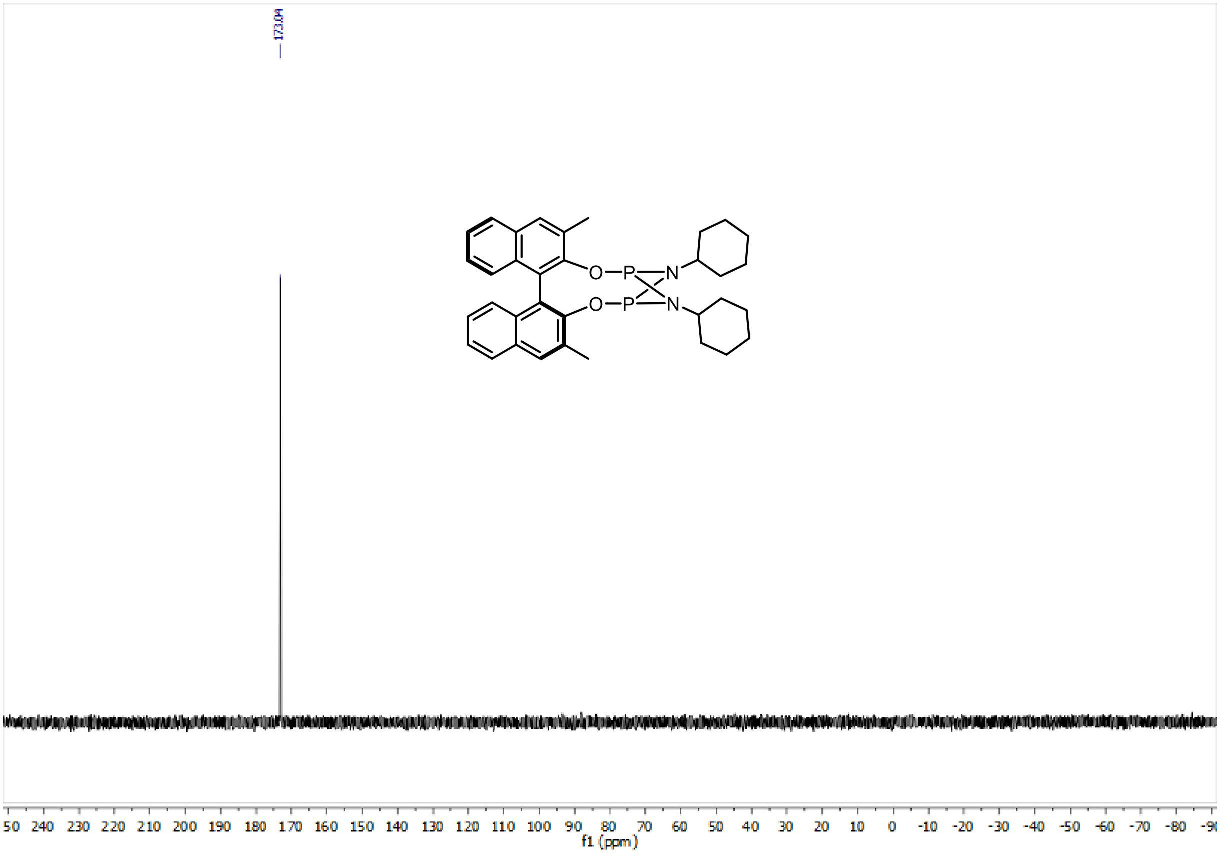
173.04
O
P
N
O
P
N
50 240 230 220 210 200 190 180 170 160 150 140 130 120 110 100 90 80 70 60 50 40 30 20 10 0 -10 -20 -30 -40 -50 -60 -70 -80
f1 (ppm)

SI-L13

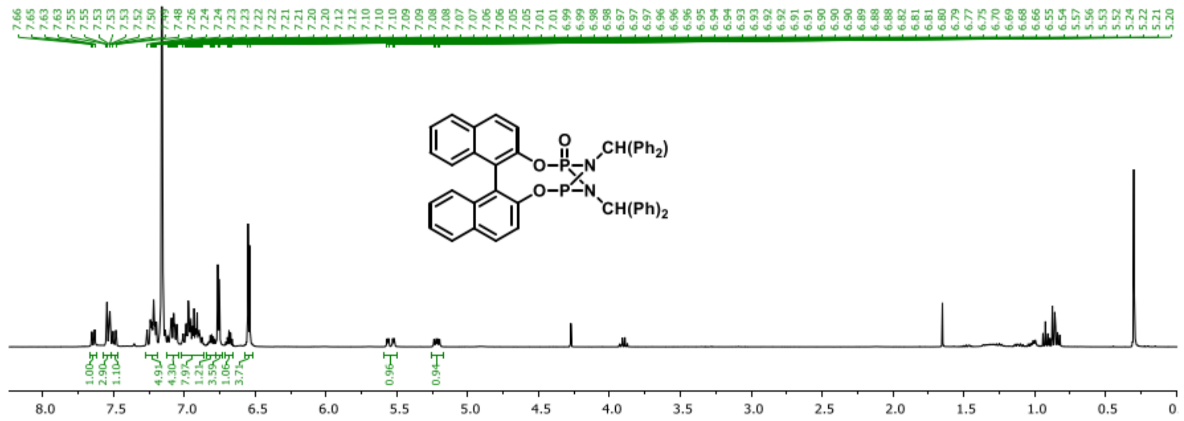

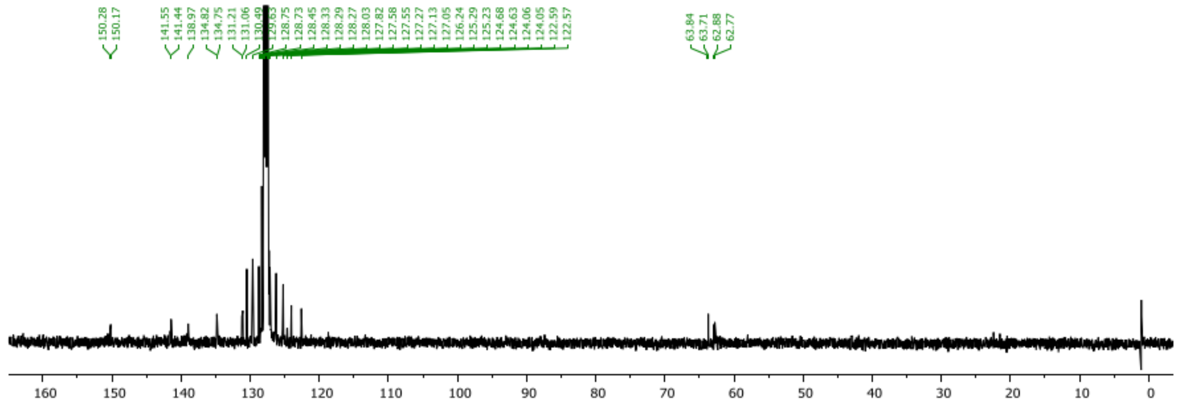

SI-L19

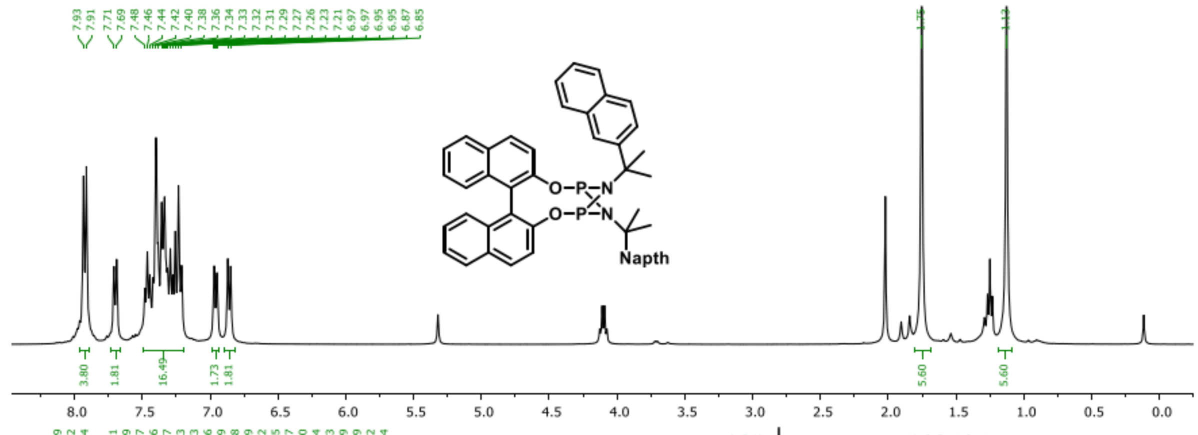

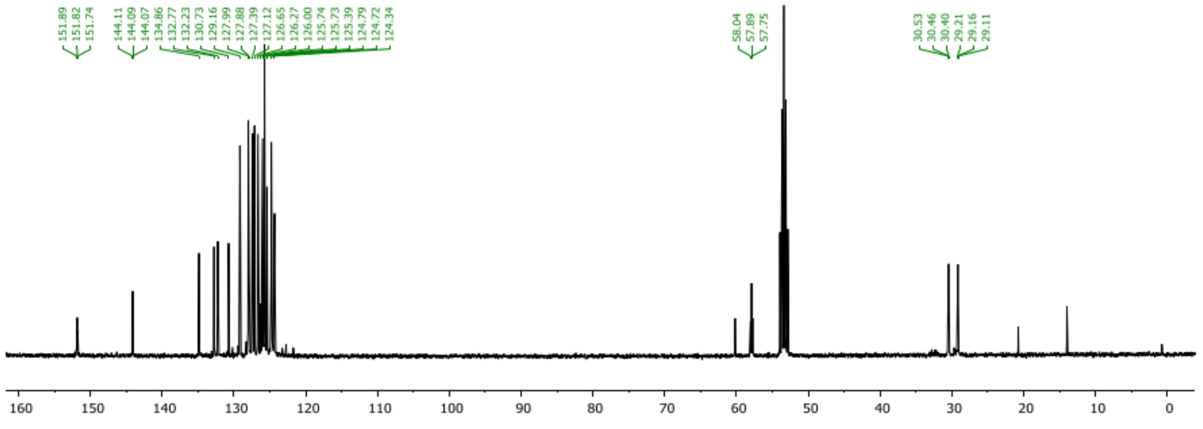

SI-L20

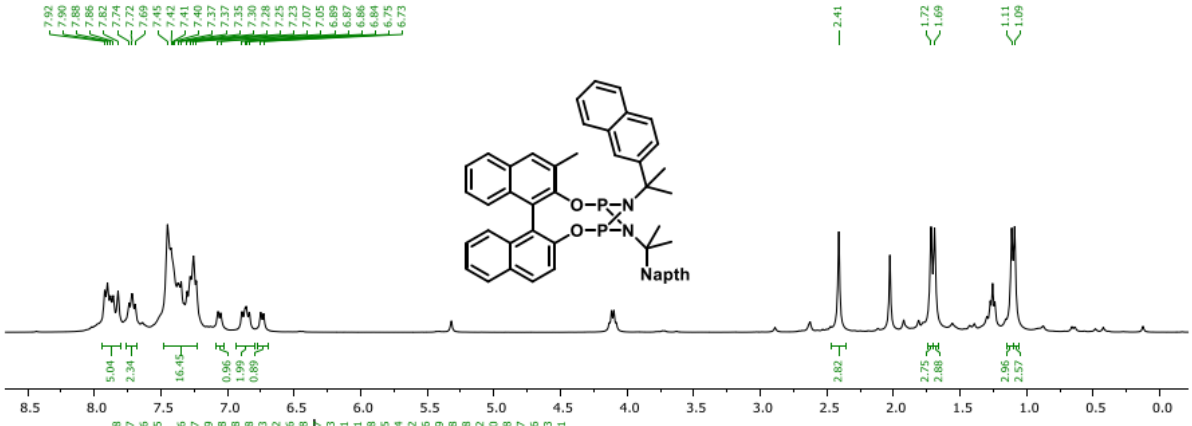

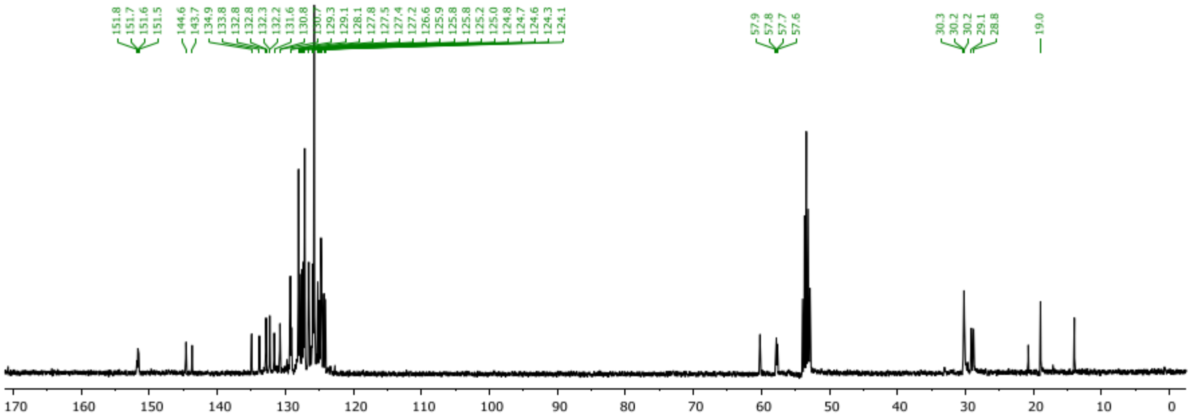

SI-L21

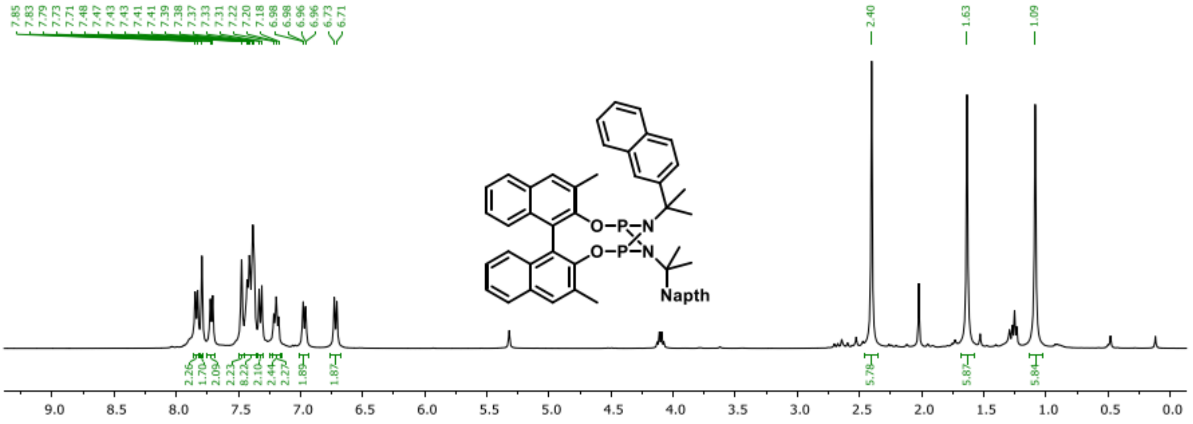

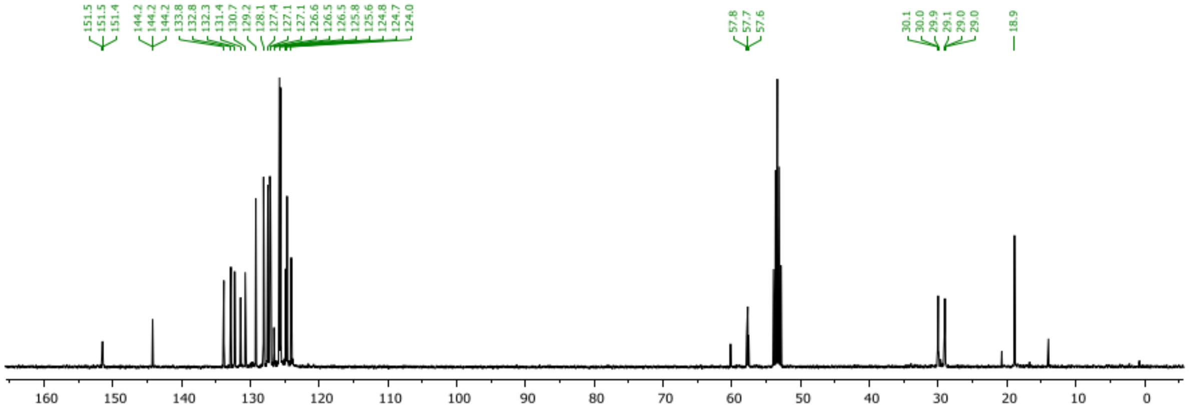

SI-L22

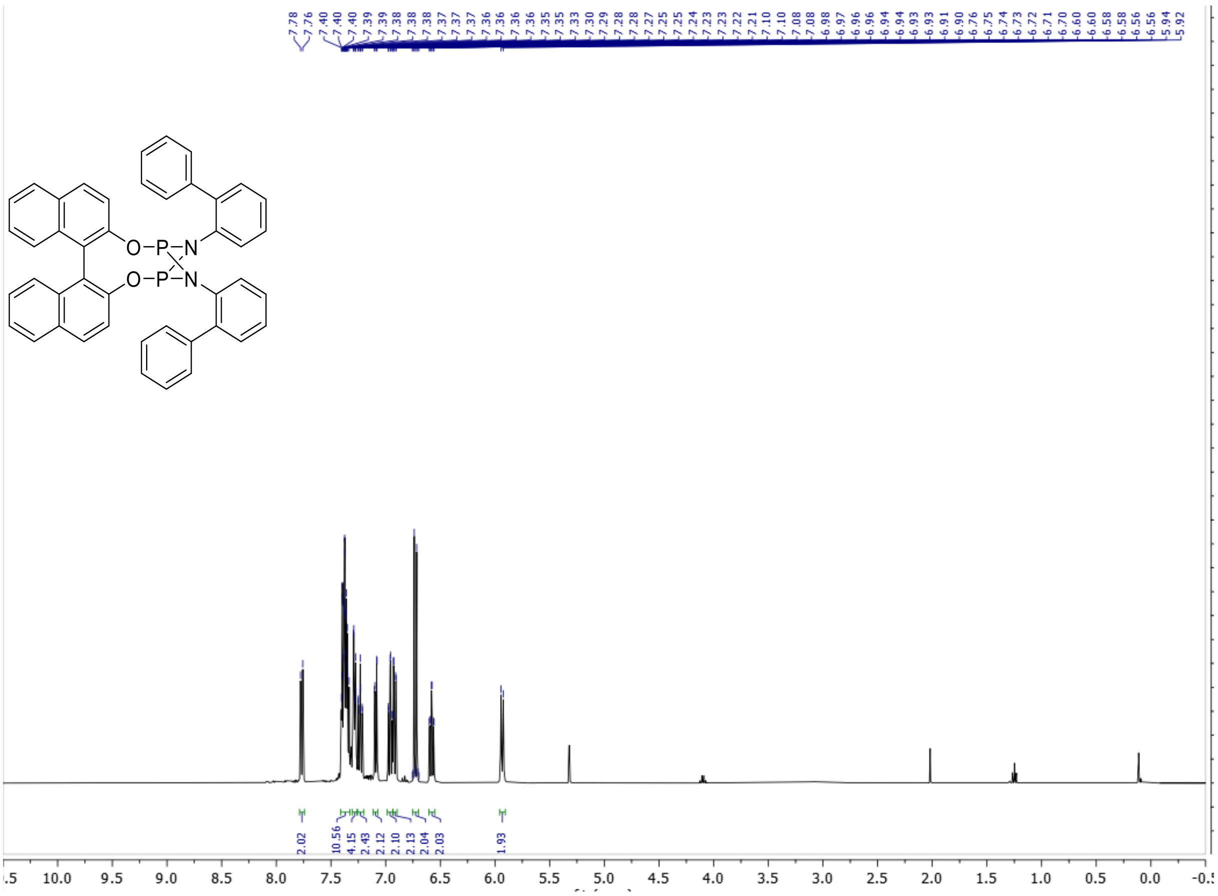
7.78
7.76
7.40
7.40
7.39
7.39
7.38
7.38
7.37
7.37
7.36
7.36
7.36
7.36
7.35
7.35
7.33
7.30
7.29
7.28
7.28
7.27
7.25
7.25
7.24
7.23
7.23
7.22
7.21
7.10
7.10
7.08
7.08
6.98
6.97
6.96
6.96
6.94
6.94
6.93
6.93
6.91
6.90
6.76
6.75
6.74
6.73
6.72
6.71
6.70
6.60
6.60
6.58
6.58
6.56
6.56
5.94
5.92
O–P–N
O–P–N
2.02
10.56
4.15
2.43
2.12
2.10
2.13
2.04
2.03
1.93
10.0 9.5 9.0 8.5 8.0 7.5 7.0 6.5 6.0 5.5 5.0 4.5 4.0 3.5 3.0 2.5 2.0 1.5 1.0 0.5 0.0

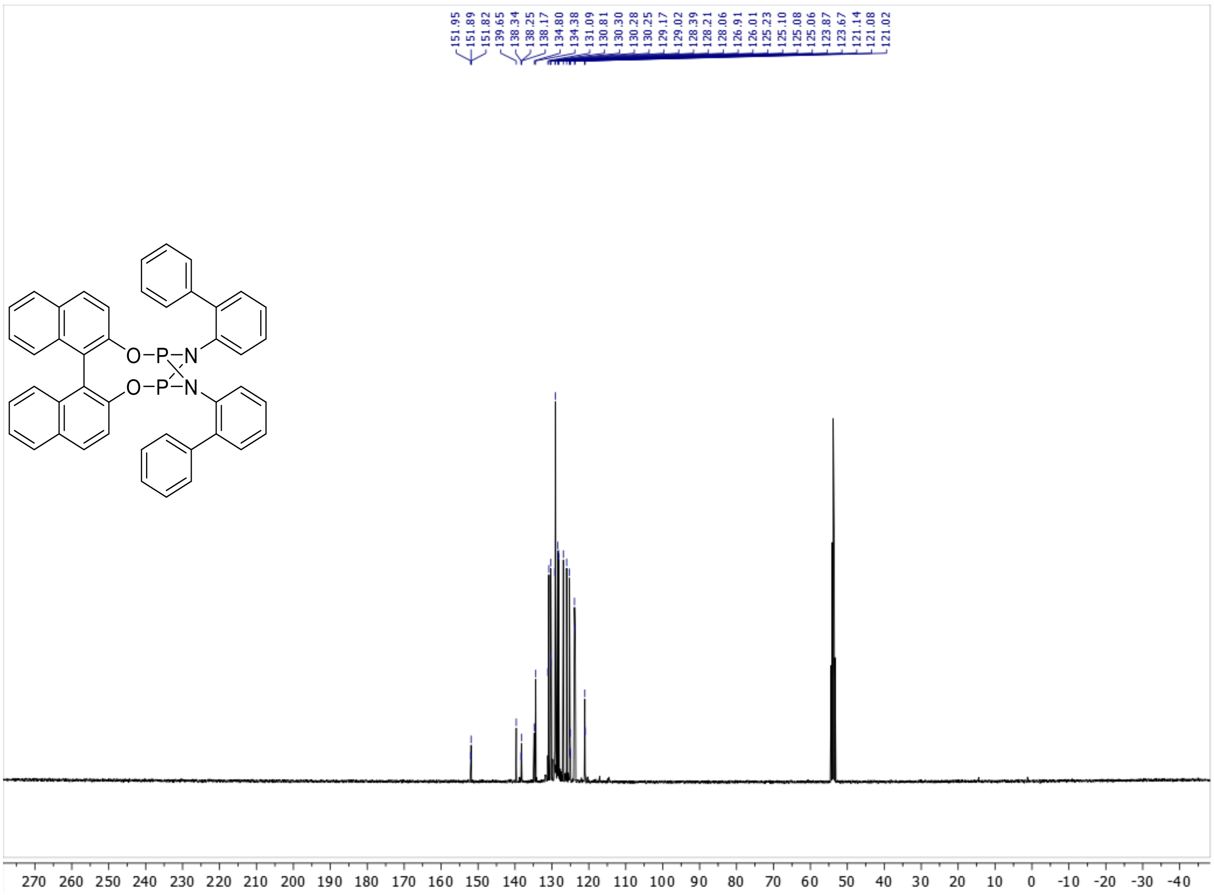
151.95
151.89
151.82
139.65
138.34
138.25
138.17
134.80
134.38
131.09
130.81
130.30
130.28
130.25
129.17
129.02
128.39
128.21
128.06
126.91
126.01
125.23
125.10
125.08
125.06
123.87
123.67
121.14
121.08
121.02
O–P–N
O–P–N
270 260 250 240 230 220 210 200 190 180 170 160 150 140 130 120 110 100 90 80 70 60 50 40 30 20 10 0 -10 -20 -30 -40

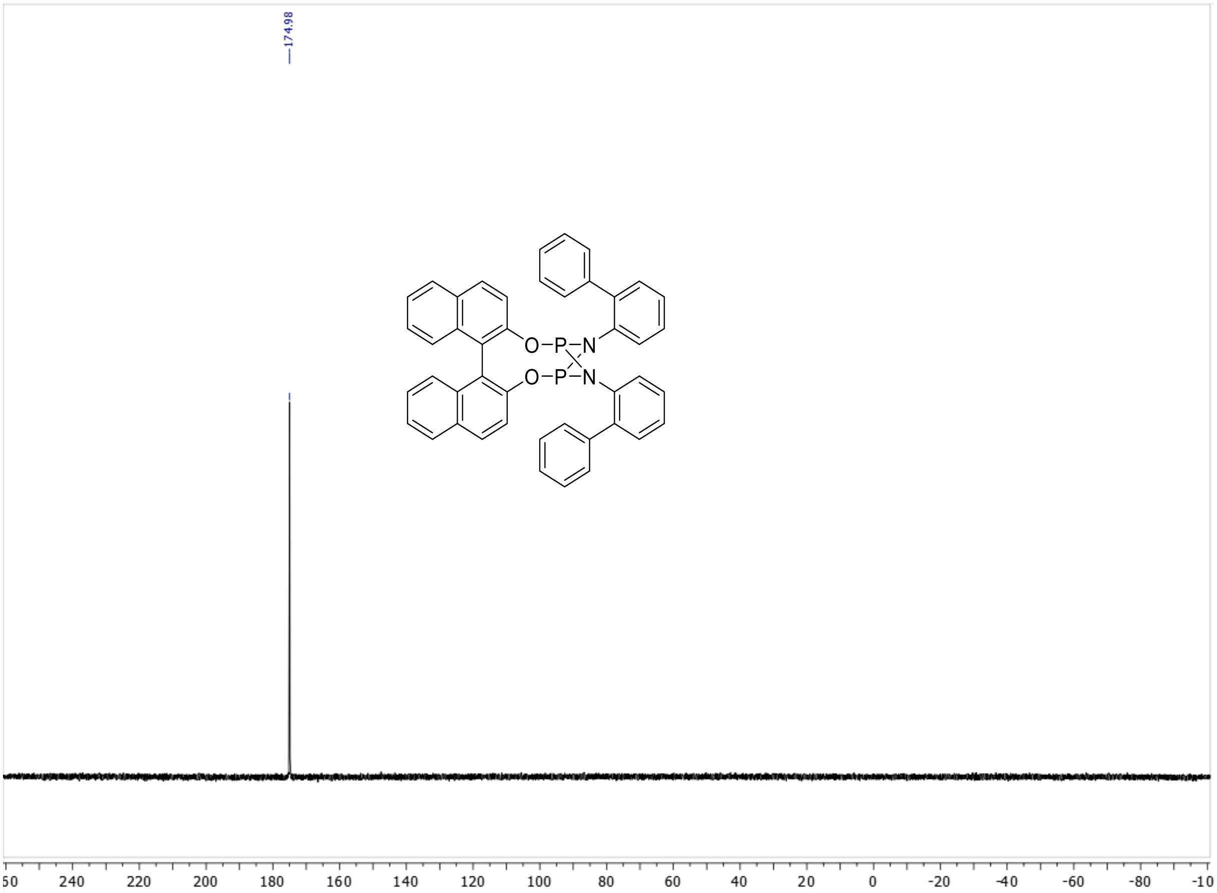

174.98
O
P
N
O
P
N
50
240
220
200
180
160
140
120
100
80
60
40
20
0
-20
-40
-60
-80
-10

SI-L23

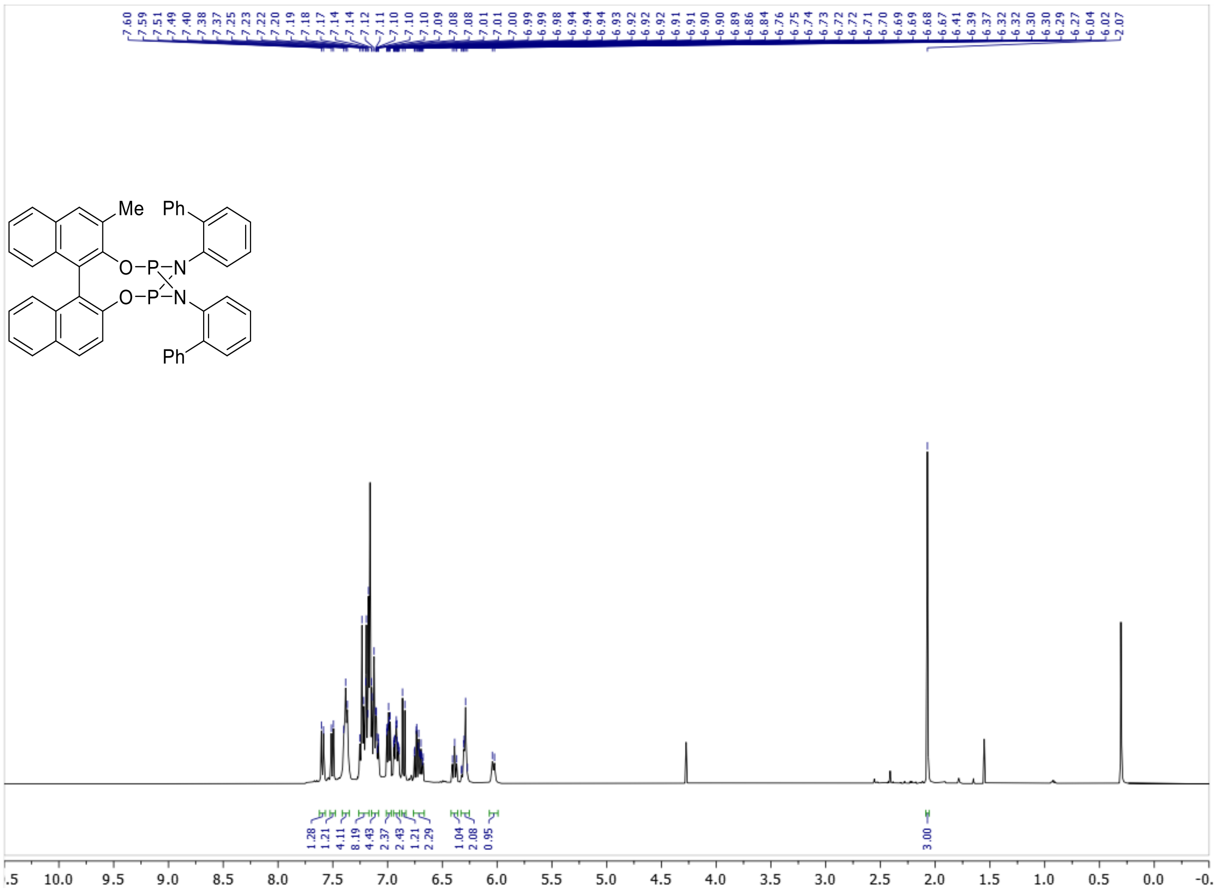

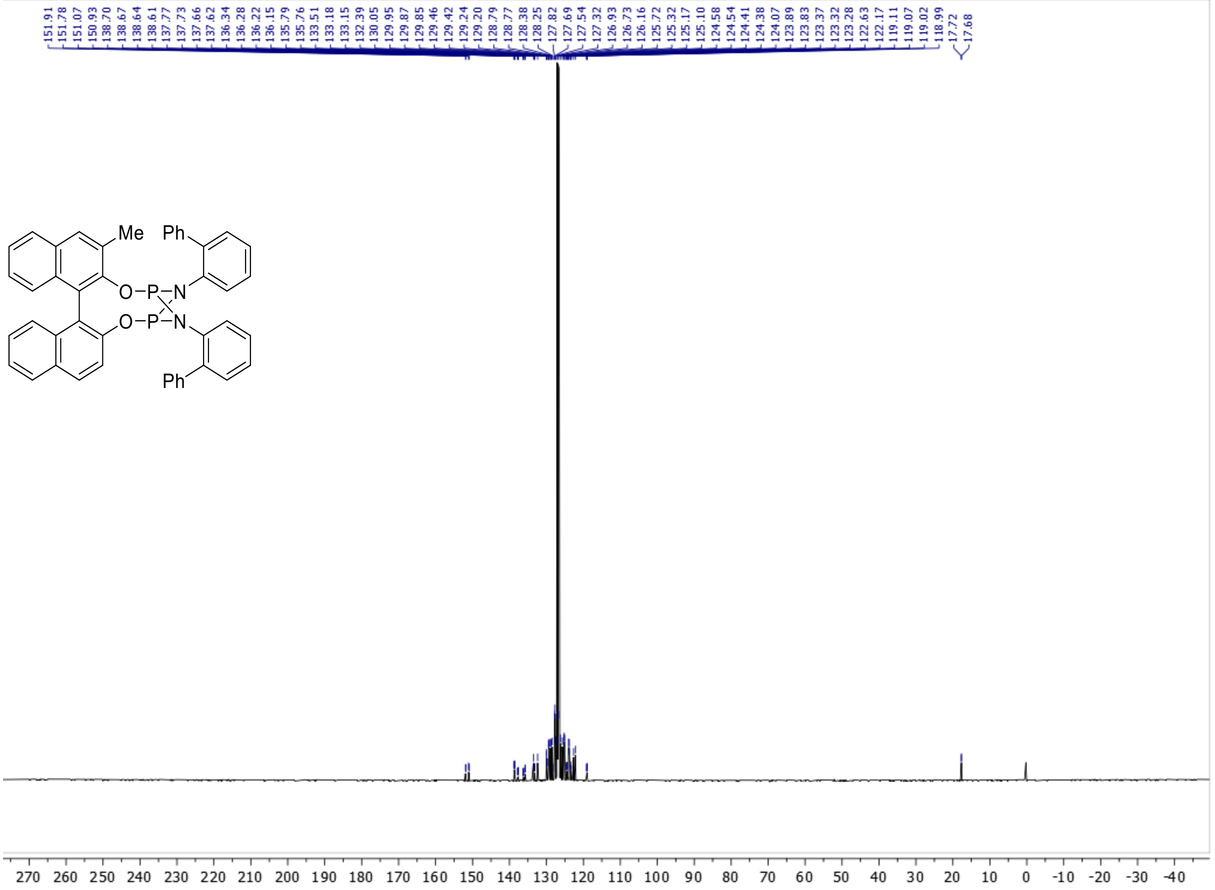

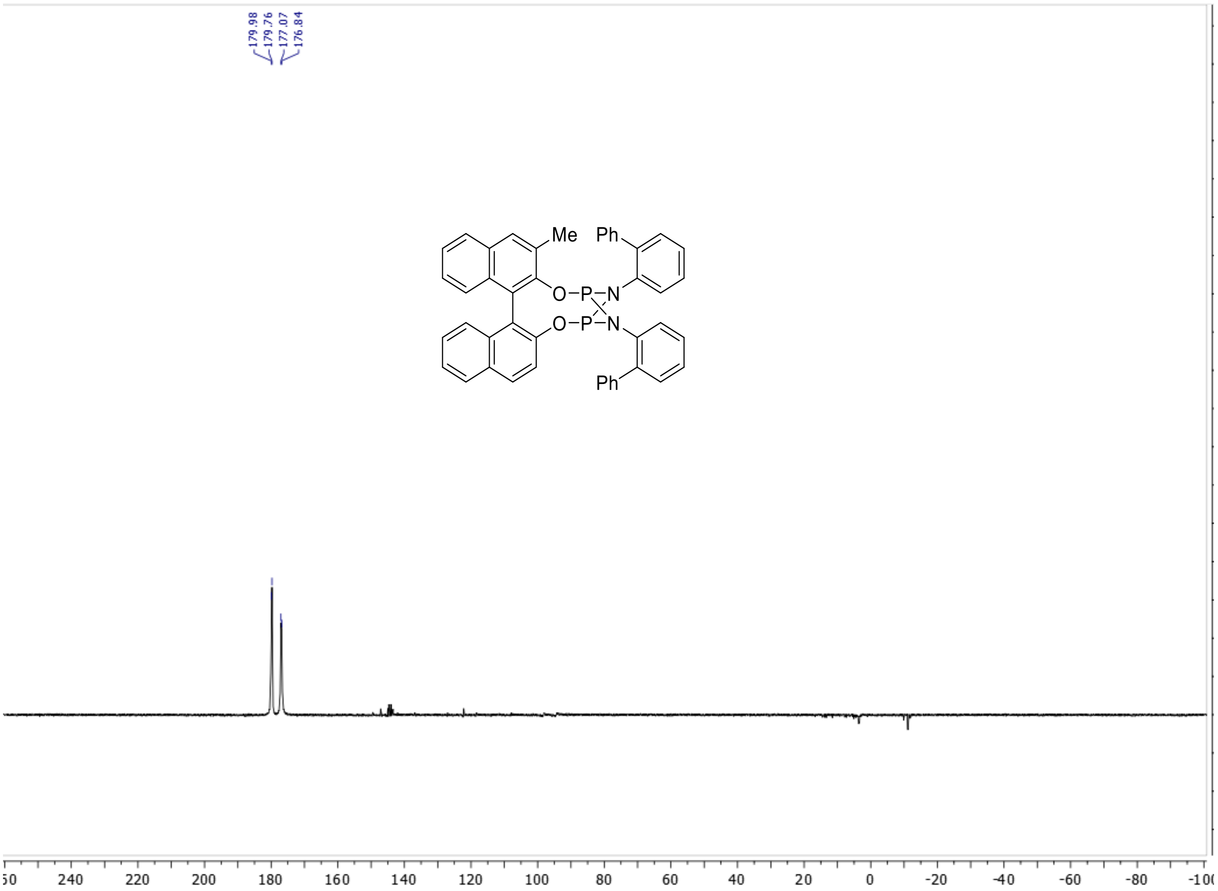
179.98
179.76
177.07
176.84
Me
Ph
O
P
N
O
P
N
Ph
240
220
200
180
160
140
120
100
80
60
40
20
0
-20
-40
-60
-80

SI-L24

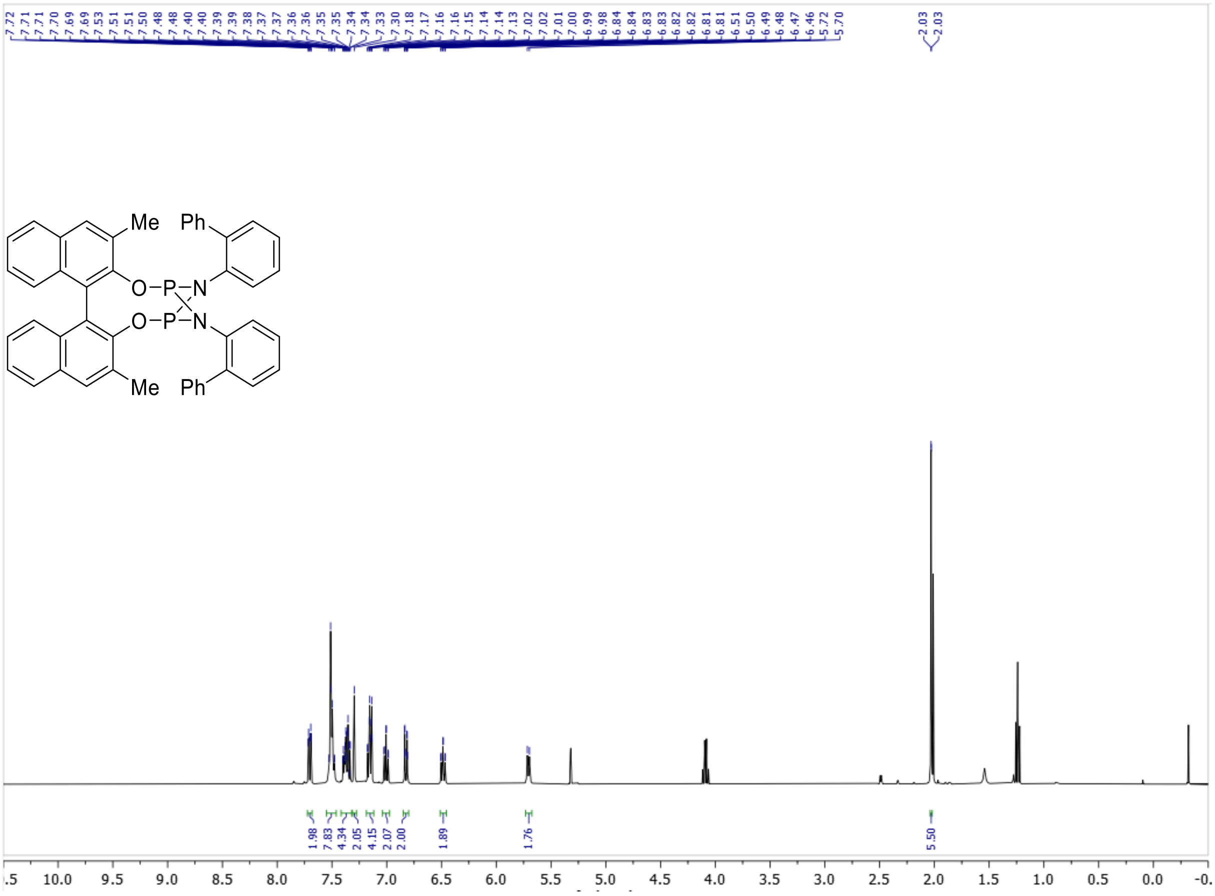

7.72
7.71
7.71
7.70
7.69
7.69
7.53
7.51
7.51
7.50
7.48
7.48
7.40
7.40
7.39
7.39
7.38
7.37
7.37
7.36
7.36
7.35
7.35
7.34
7.34
7.33
7.30
7.18
7.17
7.16
7.16
7.15
7.14
7.14
7.13
7.02
7.02
7.01
7.01
7.00
6.99
6.98
6.84
6.84
6.83
6.83
6.82
6.82
6.81
6.81
6.51
6.50
6.49
6.48
6.47
6.46
5.72
5.70
2.03
2.03
Me
Ph
O
P
N
O
P
N
Me
Ph
1.98
7.83
4.34
2.05
4.15
2.07
2.00
1.89
1.76
5.50
10.0 9.5 9.0 8.5 8.0 7.5 7.0 6.5 6.0 5.5 5.0 4.5 4.0 3.5 3.0 2.5 2.0 1.5 1.0 0.5 0.0

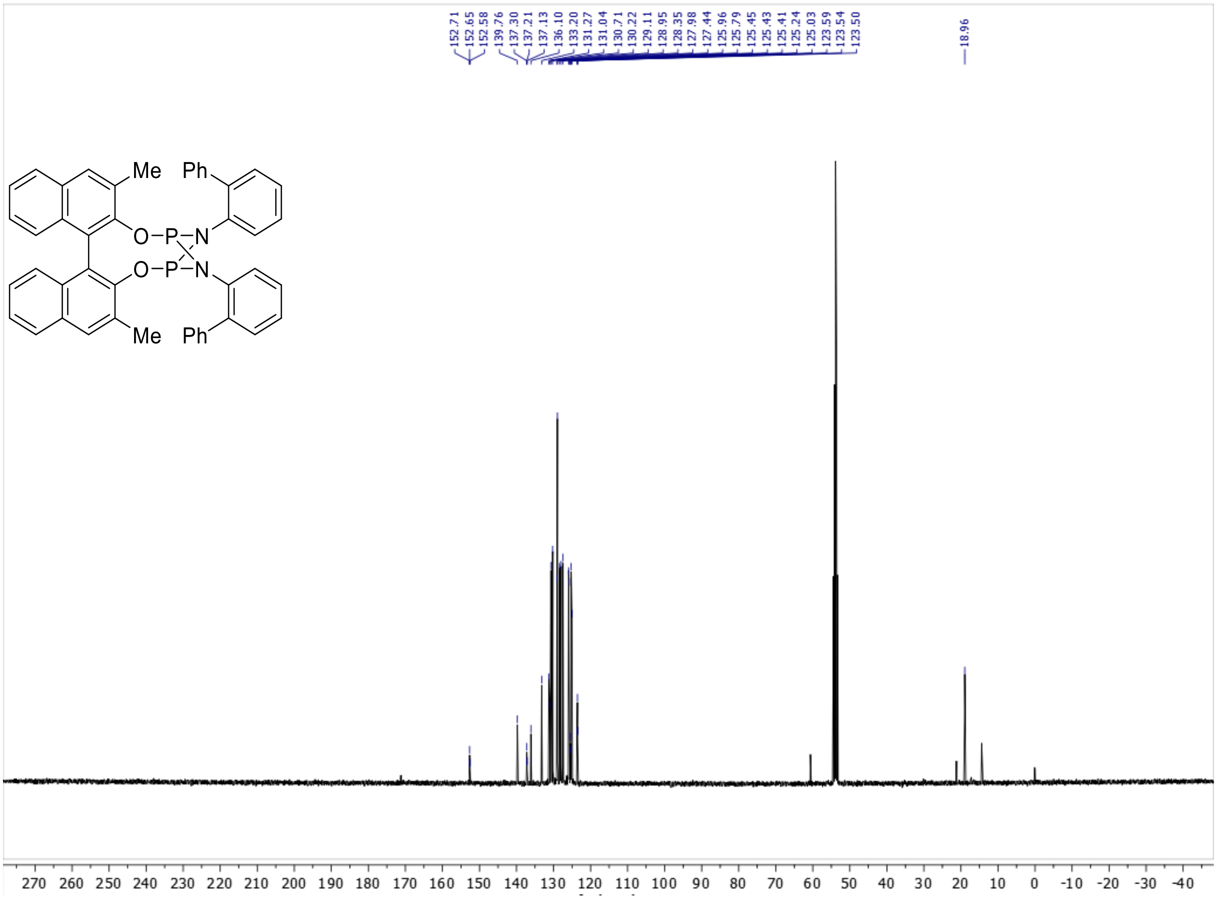

152.71
152.65
152.58
139.76
137.30
137.21
137.13
136.10
133.20
131.27
131.04
130.71
130.22
129.11
128.95
128.35
127.98
127.44
125.96
125.79
125.45
125.43
125.41
125.24
125.03
123.59
123.54
123.50
18.96
Me
Ph
O
P
N
O
P
N
Me
Ph
270 260 250 240 230 220 210 200 190 180 170 160 150 140 130 120 110 100 90 80 70 60 50 40 30 20 10 0 -10 -20 -30 -40

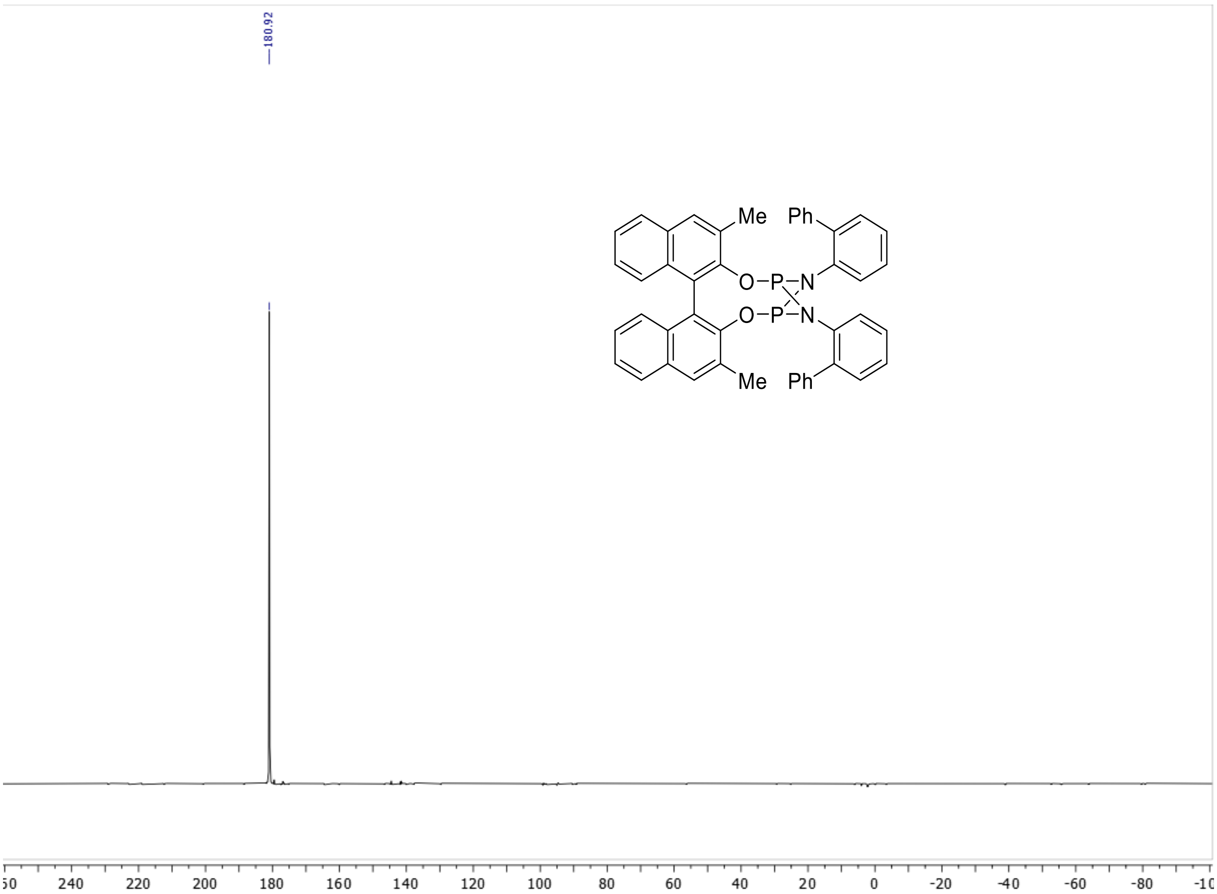

180.92
Me
Ph
O
P
N
O
P
N
Me
Ph
50
240
220
200
180
160
140
120
100
80
60
40
20
0
-20
-40
-60
-80
-10

SI-L25

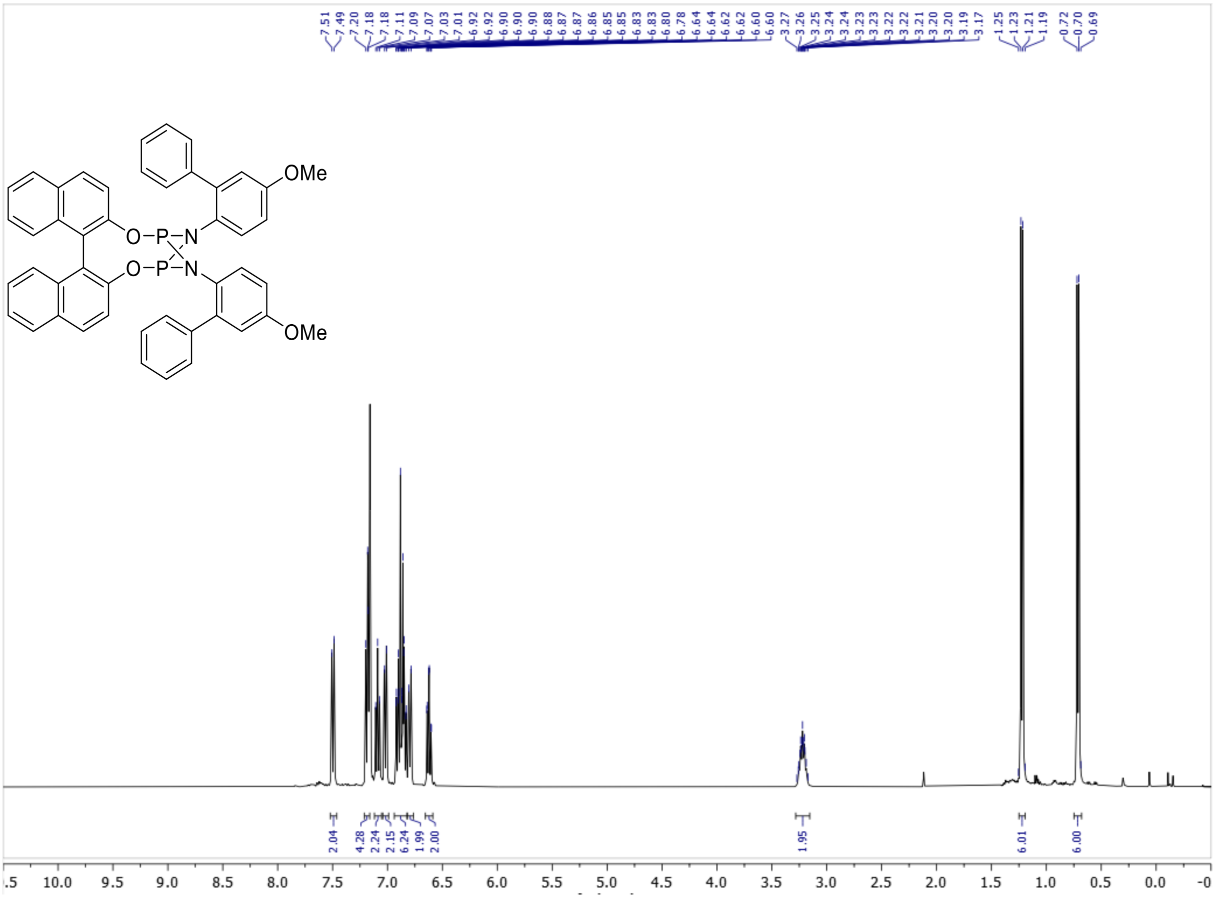

7.51 7.49 7.20 7.18 7.18 7.11 7.09 7.07 7.03 7.01 6.92 6.92 6.90 6.90 6.90 6.88 6.87 6.87 6.86 6.85 6.85 6.83 6.83 6.80 6.78 6.64 6.62 6.62 6.60 6.60 3.27 3.26 3.25 3.24 3.24 3.23 3.23 3.22 3.22 3.21 3.20 3.20 3.19 3.17 1.25 1.23 1.21 1.19 0.72 0.70 0.69
OMe
O
P
N
OMe
2.04 4.28 2.24 2.15 6.24 1.99 2.00 1.95 6.01 6.00
10.0 9.5 9.0 8.5 8.0 7.5 7.0 6.5 6.0 5.5 5.0 4.5 4.0 3.5 3.0 2.5 2.0 1.5 1.0 0.5 0.0

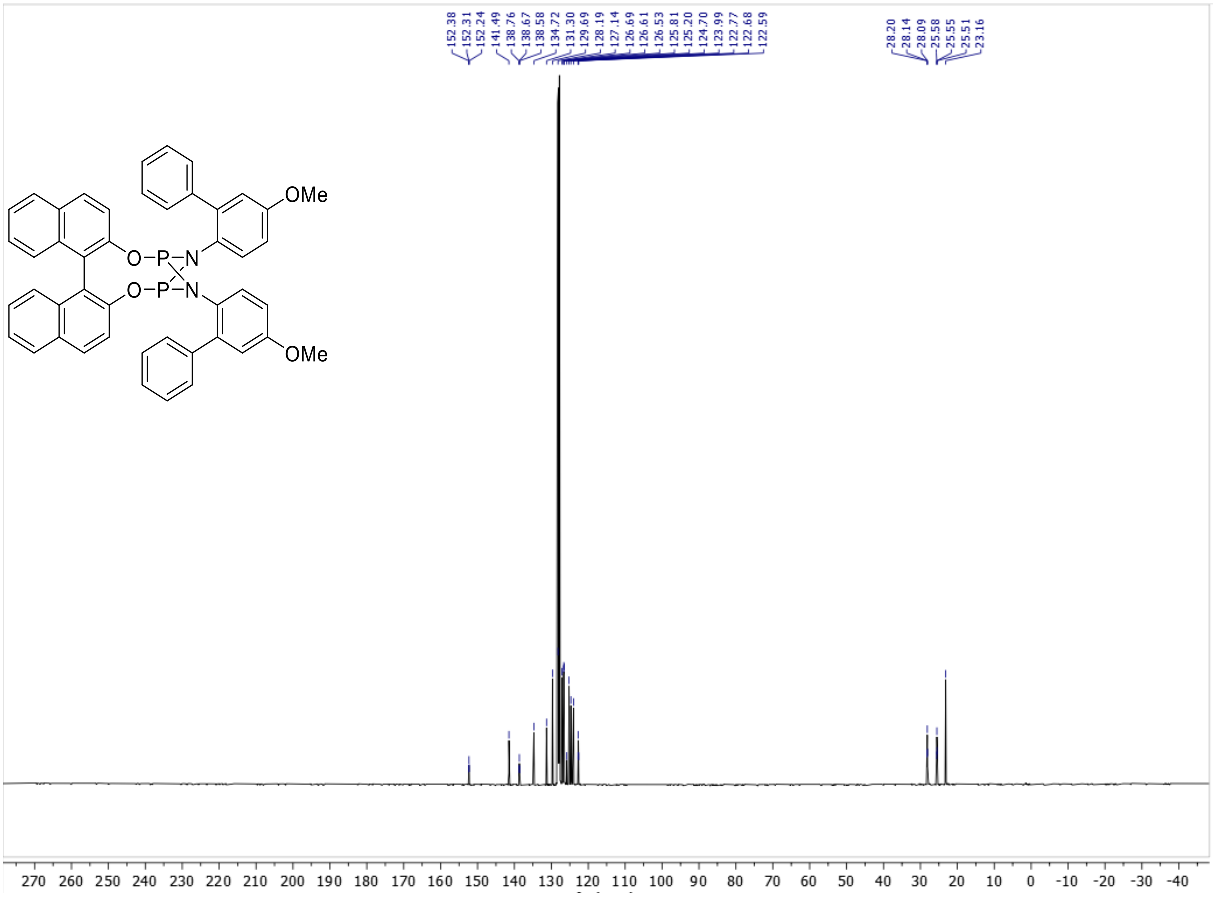

152.38 152.31 152.24 141.49 138.76 138.67 138.58 134.72 131.30 129.69 128.19 127.14 126.69 126.61 126.53 125.81 125.20 124.70 123.99 122.77 122.68 122.59 28.20 28.14 28.09 25.58 25.55 25.51 23.16
OMe
O
P
N
OMe
270 260 250 240 230 220 210 200 190 180 170 160 150 140 130 120 110 100 90 80 70 60 50 40 30 20 10 0 -10 -20 -30 -40

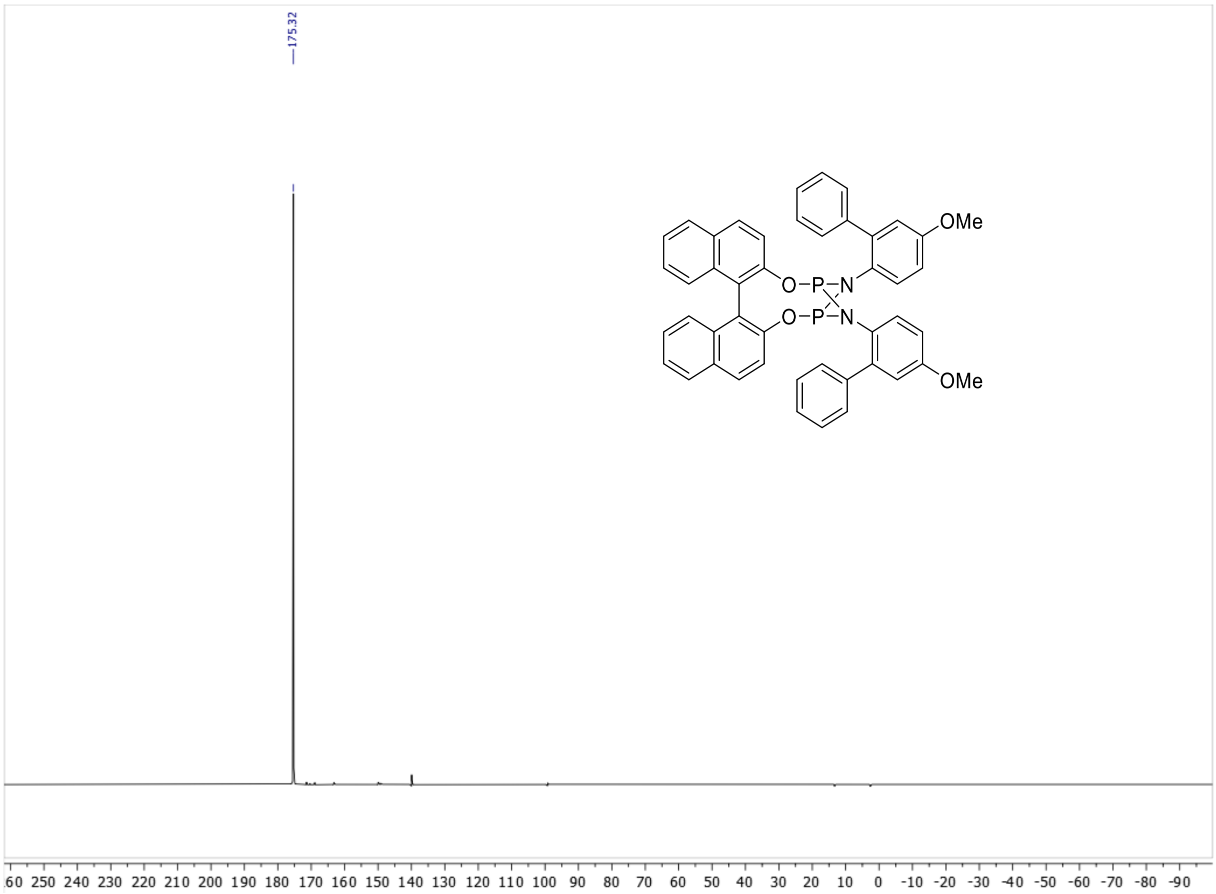
175.32
OMe
O
P
N
O
P
N
OMe
60 250 240 230 220 210 200 190 180 170 160 150 140 130 120 110 100 90 80 70 60 50 40 30 20 10 0 -10 -20 -30 -40 -50 -60 -70 -80 -90

SI-L26

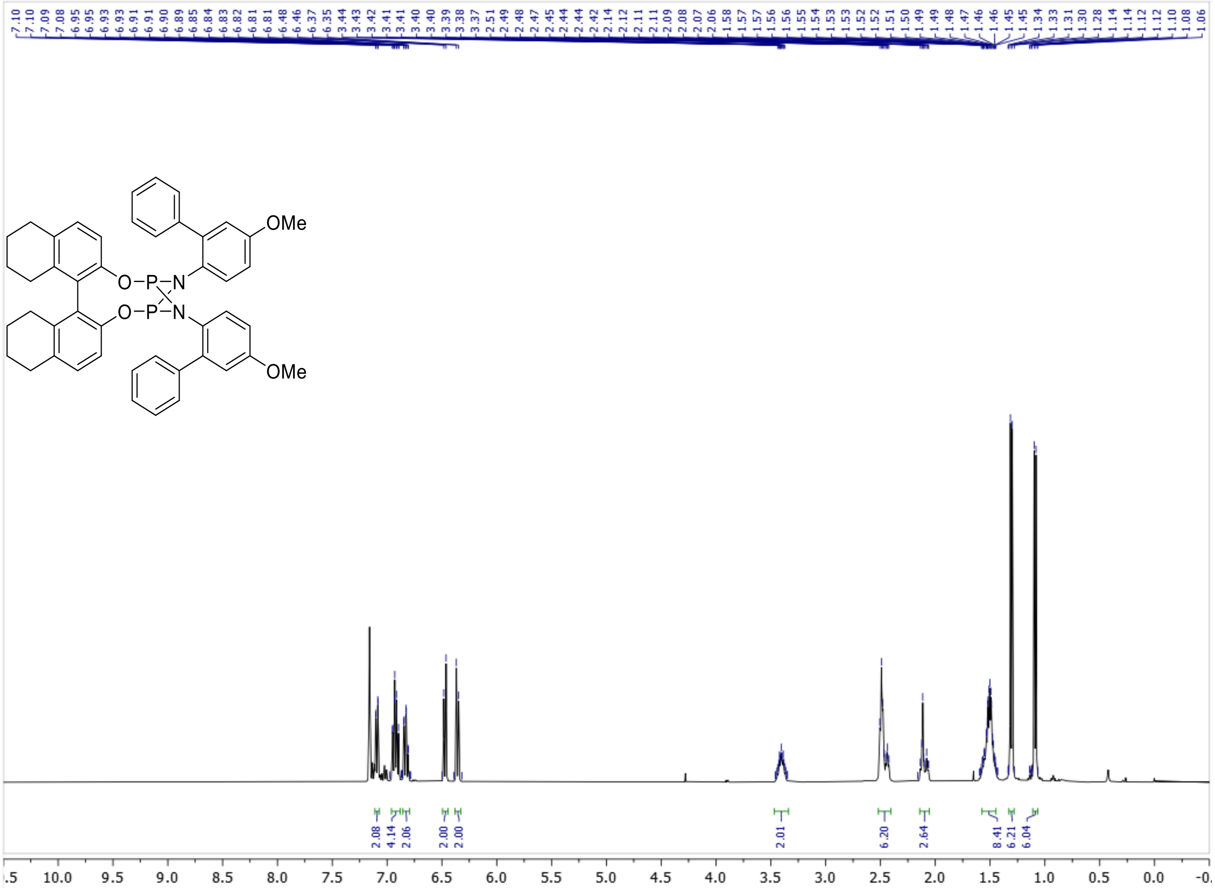

7.10 7.10 7.09 7.08 6.95 6.95 6.93 6.93 6.91 6.91 6.90 6.89 6.85 6.84 6.83 6.82 6.81 6.81 6.48 6.46 6.37 6.35 3.44 3.43 3.42 3.41 3.41 3.40 3.39 3.38 3.37 2.51 2.49 2.48 2.47 2.45 2.44 2.44 2.42 2.14 2.12 2.11 2.11 2.09 2.08 2.07 2.06 1.58 1.57 1.57 1.56 1.56 1.55 1.54 1.53 1.53 1.52 1.52 1.51 1.50 1.49 1.49 1.48 1.47 1.46 1.46 1.45 1.45 1.34 1.33 1.31 1.30 1.28 1.14 1.14 1.12 1.12 1.10 1.08 1.06
OMe
O–P–N
O–P–N
OMe
2.08 4.14 2.06 2.00 2.00 2.01 6.20 2.64 8.41 6.21 6.04
10.0 9.5 9.0 8.5 8.0 7.5 7.0 6.5 6.0 5.5 5.0 4.5 4.0 3.5 3.0 2.5 2.0 1.5 1.0 0.5 0.0

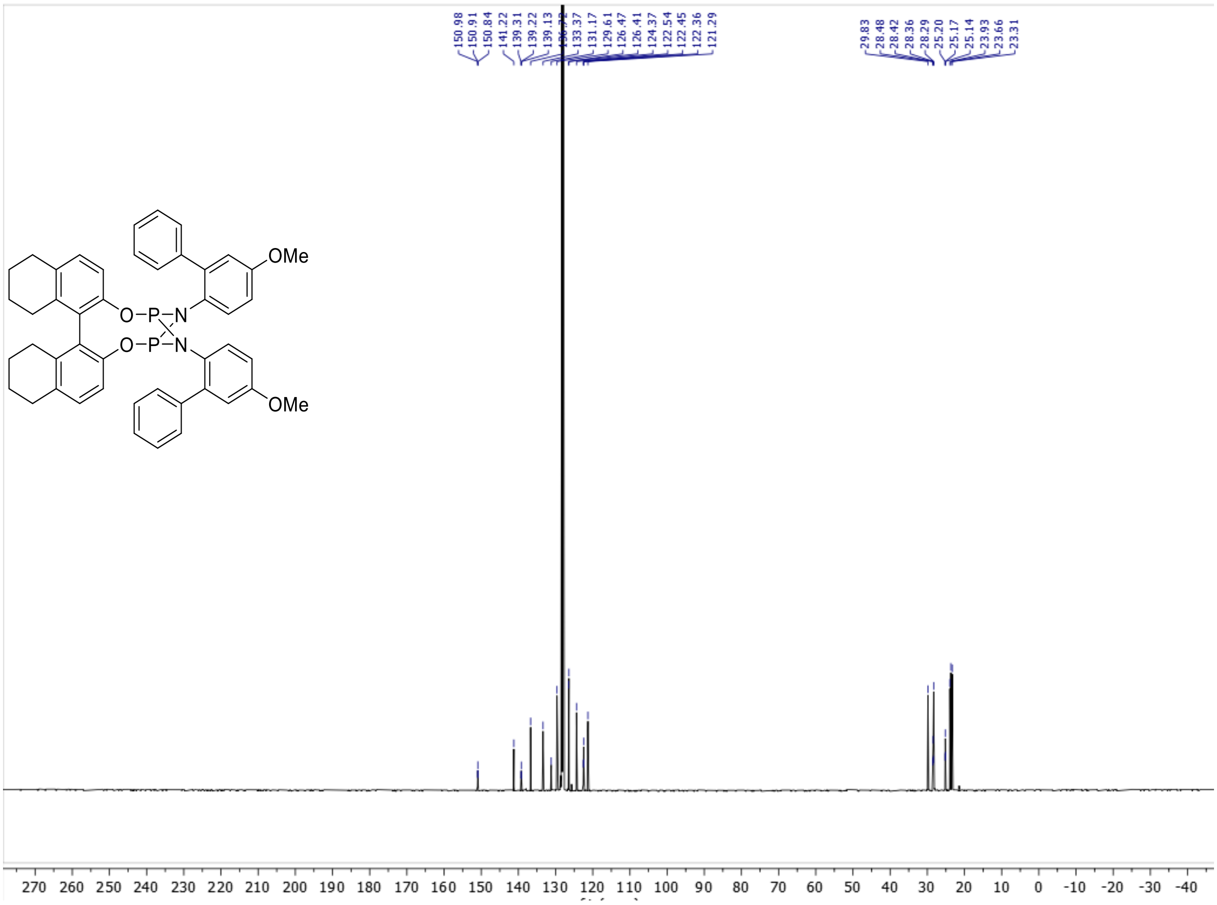

150.98 150.91 150.84 141.22 139.31 139.22 139.13 133.37 131.17 129.61 126.47 126.41 124.37 122.54 122.45 122.36 121.29
29.83 28.48 28.42 28.36 28.29 25.20 25.17 25.14 23.93 23.66 23.31
OMe
O–P–N
O–P–N
OMe
270 260 250 240 230 220 210 200 190 180 170 160 150 140 130 120 110 100 90 80 70 60 50 40 30 20 10 0 -10 -20 -30 -40

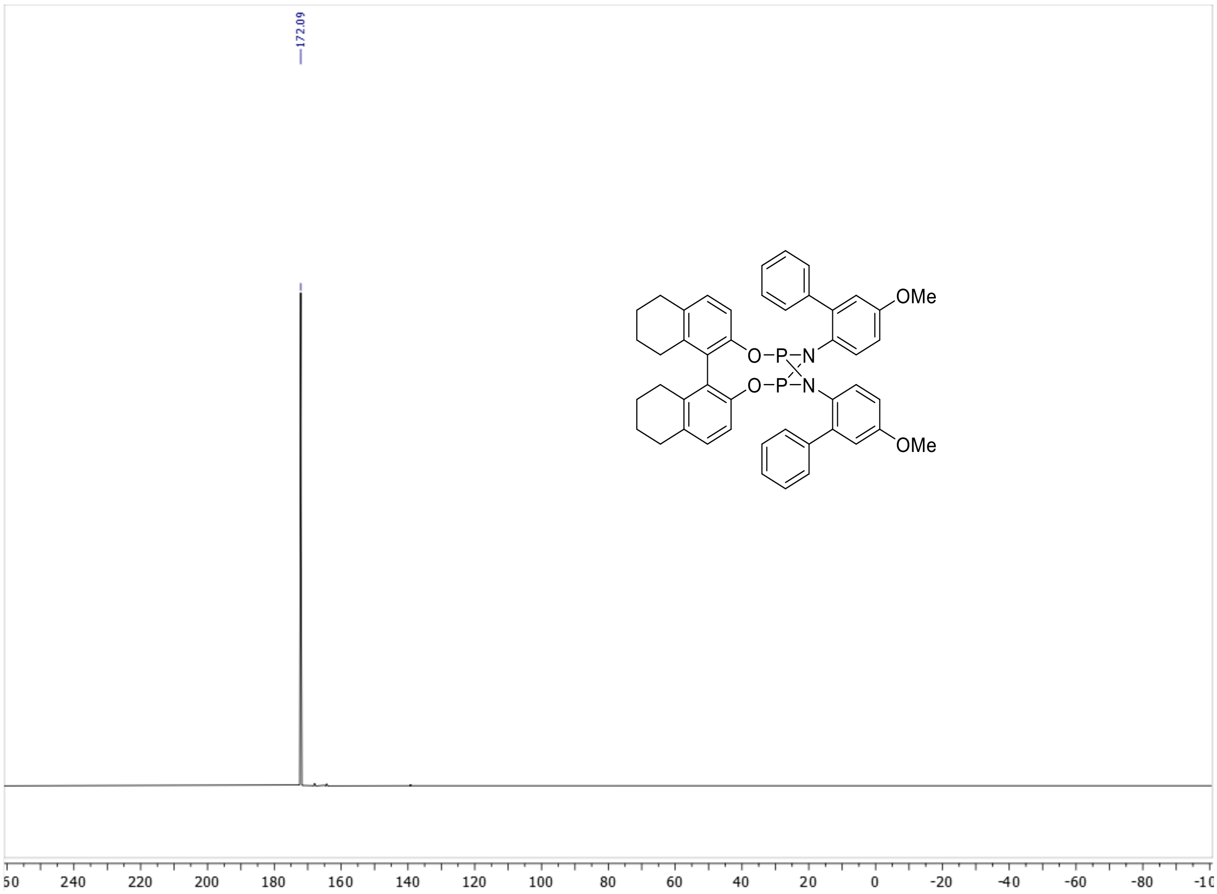

172.09
OMe
O
P
N
O
P
N
OMe
50
240
220
200
180
160
140
120
100
80
60
40
20
0
-20
-40
-60
-80
-10

SI-L27

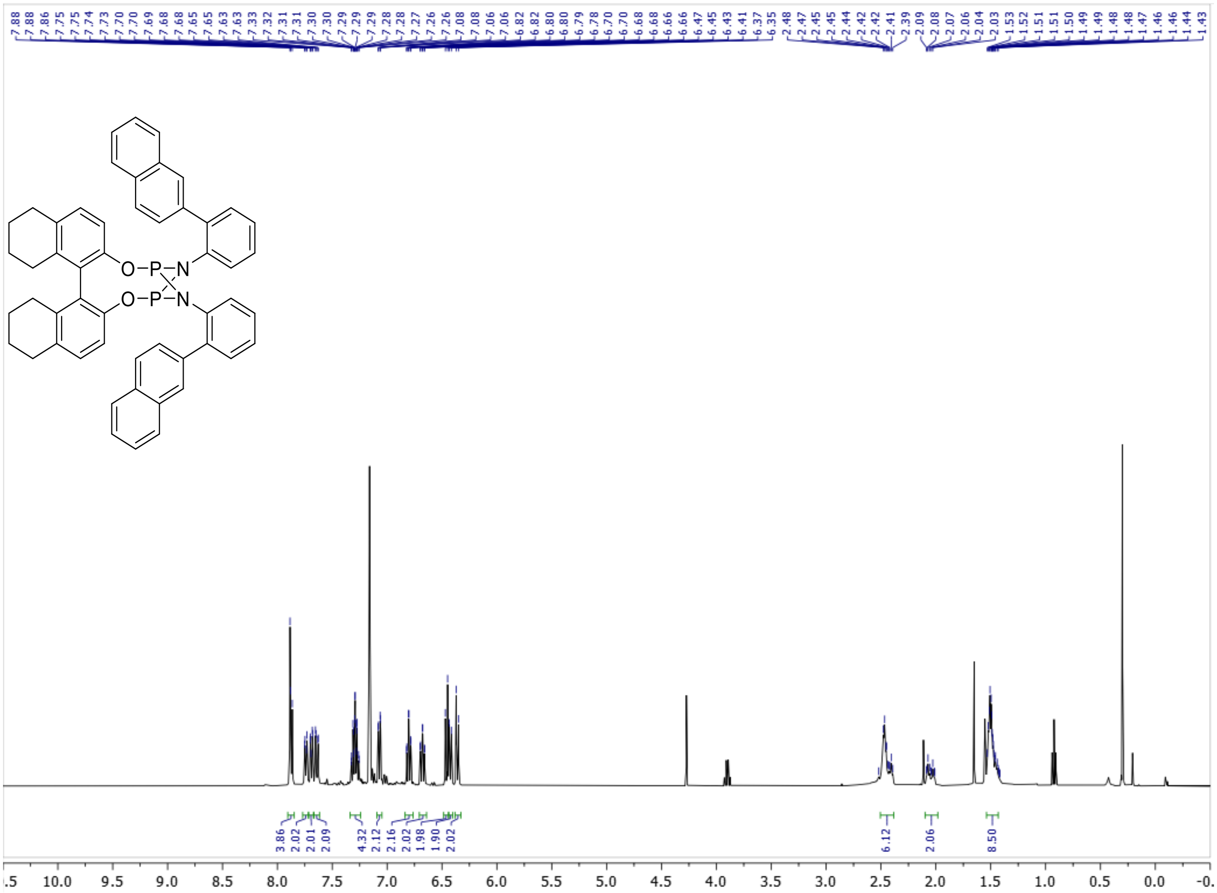
7.88 7.86 7.75 7.75 7.74 7.73 7.70 7.70 7.69 7.68 7.68 7.65 7.65 7.63 7.63 7.33 7.32 7.31 7.31 7.30 7.30 7.29 7.29 7.29 7.28 7.27 7.26 7.26 7.08 7.08 7.06 7.06 6.82 6.82 6.80 6.80 6.79 6.78 6.70 6.70 6.68 6.68 6.66 6.66 6.47 6.45 6.43 6.41 6.37 6.35 2.48 2.47 2.45 2.45 2.44 2.42 2.42 2.41 2.39 2.09 2.08 2.07 2.06 2.04 2.03 1.53 1.52 1.51 1.50 1.49 1.48 1.47 1.46 1.44 1.43
O
P
N
3.86 2.02 2.01 2.09 4.32 2.12 2.16 2.02 1.98 1.90 2.02 6.12 2.06 8.50
.5 10.0 9.5 9.0 8.5 8.0 7.5 7.0 6.5 6.0 5.5 5.0 4.5 4.0 3.5 3.0 2.5 2.0 1.5 1.0 0.5 0.0 -0.

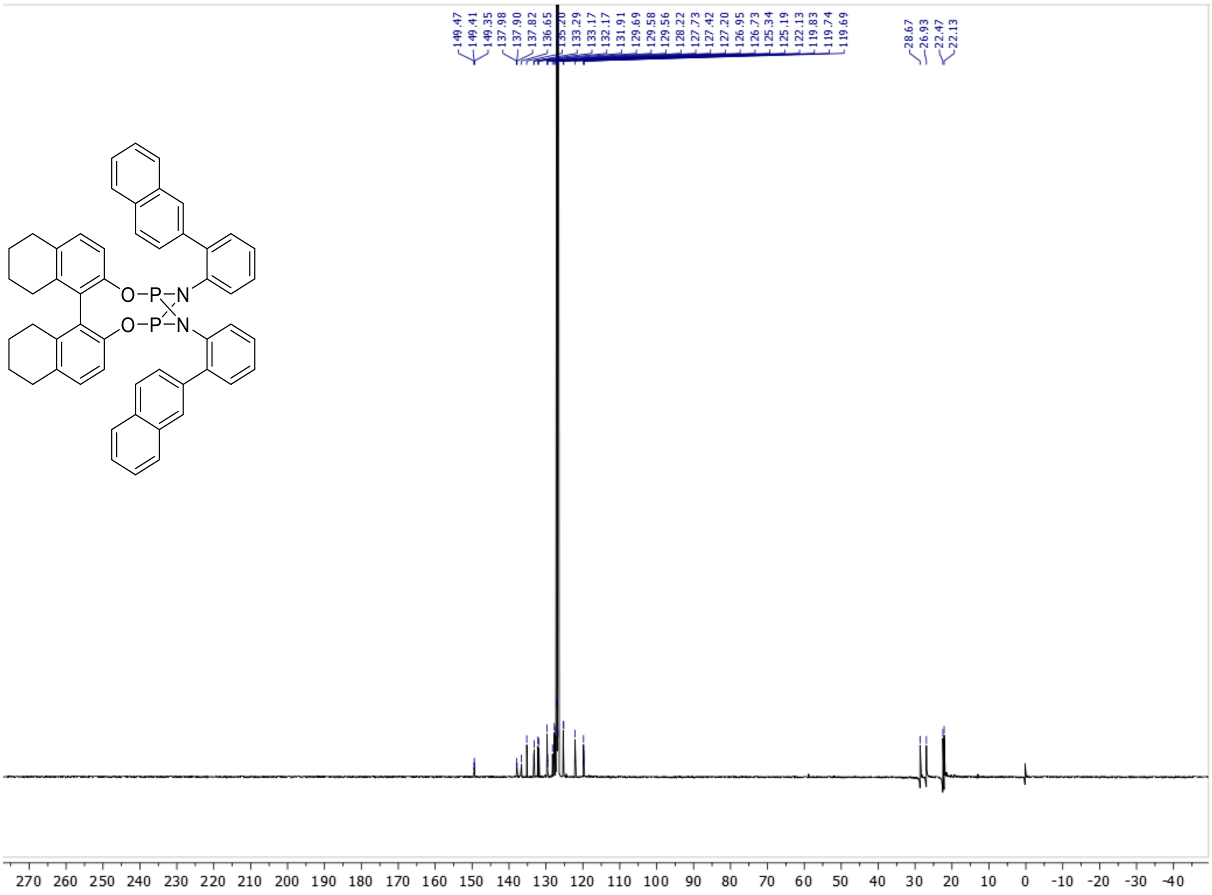
149.47 149.41 149.35 137.98 137.90 137.82 136.65 135.20 133.29 133.17 131.91 129.69 129.58 129.56 128.22 127.73 127.42 127.20 126.95 126.73 125.34 125.19 122.13 119.83 119.74 119.69
28.67 26.93 22.47 22.13
O
P
N
270 260 250 240 230 220 210 200 190 180 170 160 150 140 130 120 110 100 90 80 70 60 50 40 30 20 10 0 -10 -20 -30 -40

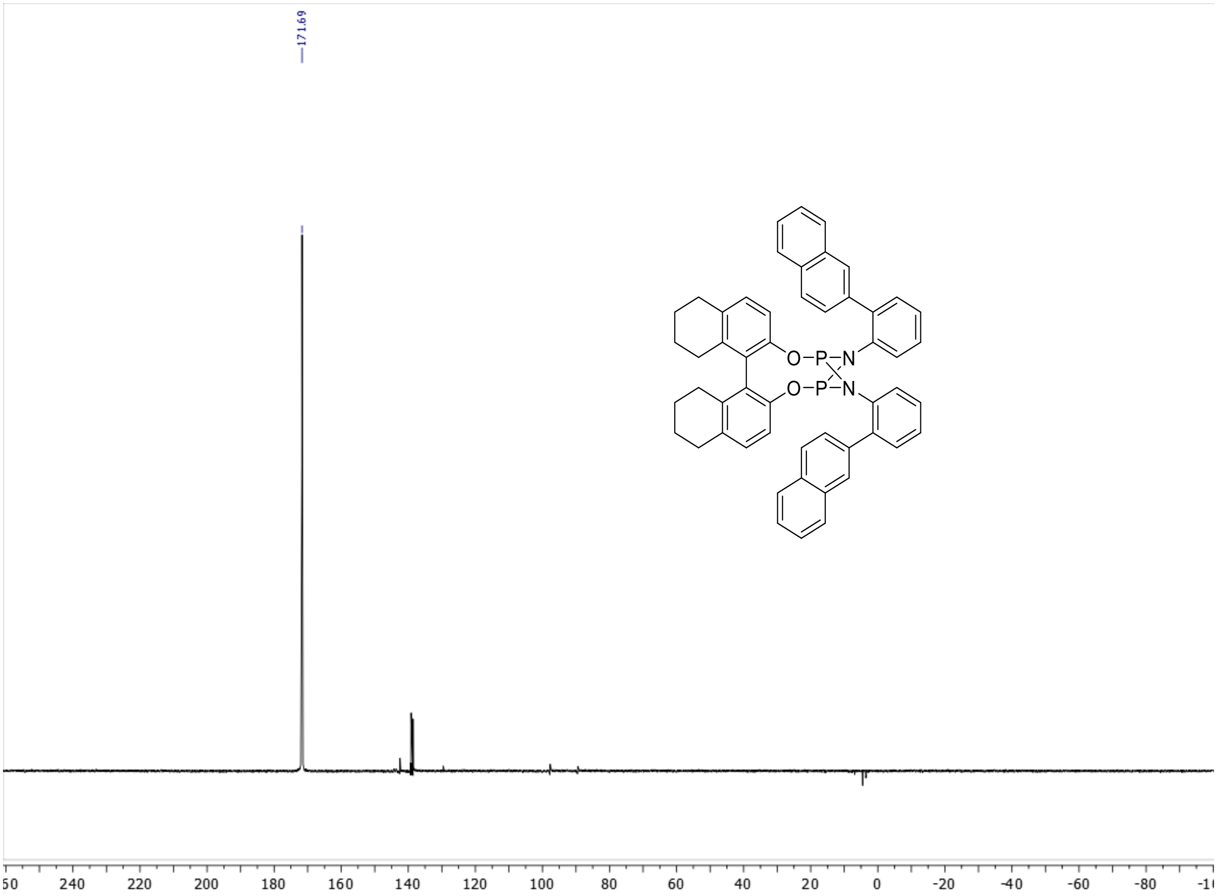

171.69
O
P
N
O
P
N
250
240
220
200
180
160
140
120
100
80
60
40
20
0
-20
-40
-60
-80
-100

SI-L28

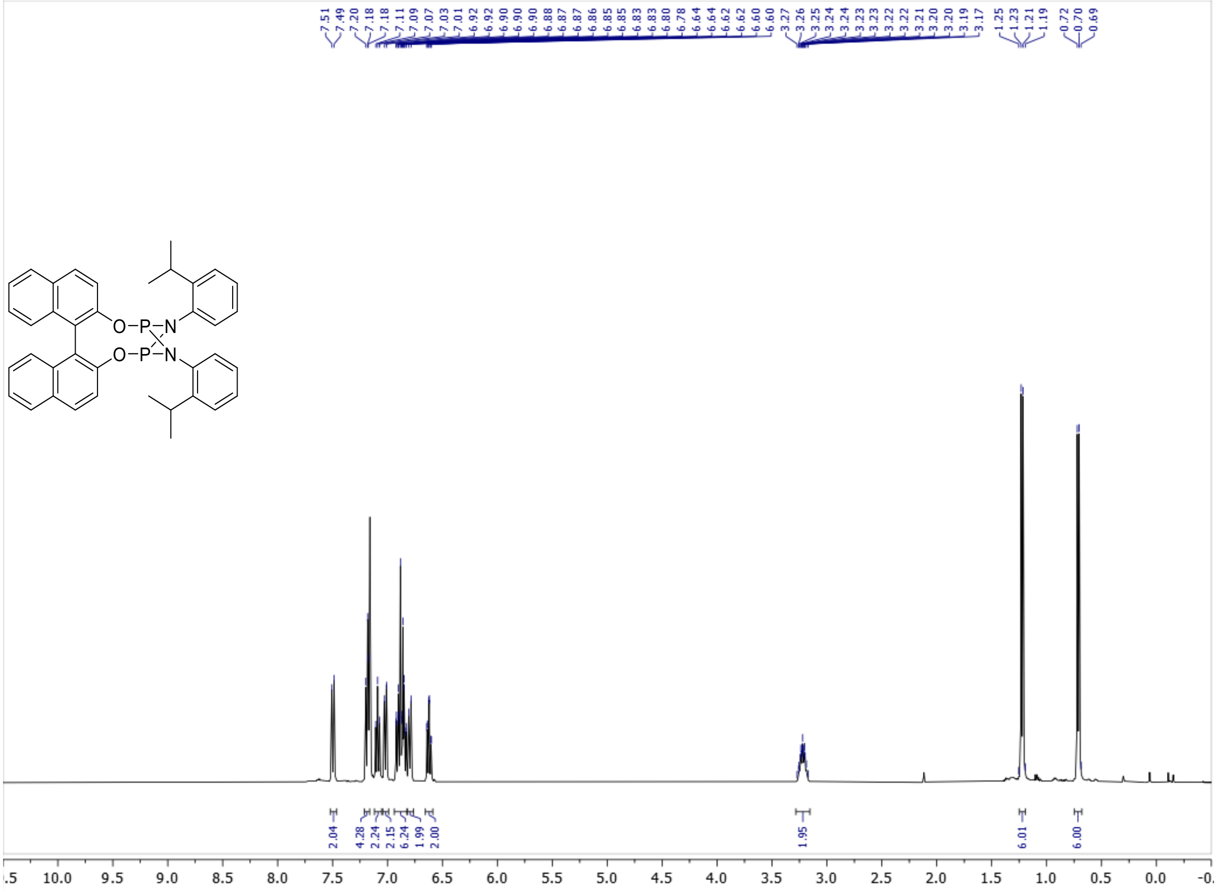
7.51
7.49
7.20
7.18
7.18
7.11
7.09
7.07
7.03
7.01
6.92
6.92
6.90
6.90
6.90
6.88
6.87
6.87
6.86
6.85
6.85
6.83
6.83
6.80
6.78
6.64
6.62
6.62
6.60
6.60
3.27
3.26
3.25
3.24
3.24
3.23
3.23
3.22
3.22
3.21
3.20
3.20
3.19
3.17
1.25
1.23
1.21
1.19
0.72
0.70
0.69
O
P
N
O
P
N
2.04
4.28
2.24
2.15
6.24
1.99
2.00
1.95
6.01
6.00
10.0
9.5
9.0
8.5
8.0
7.5
7.0
6.5
6.0
5.5
5.0
4.5
4.0
3.5
3.0
2.5
2.0
1.5
1.0
0.5
0.0

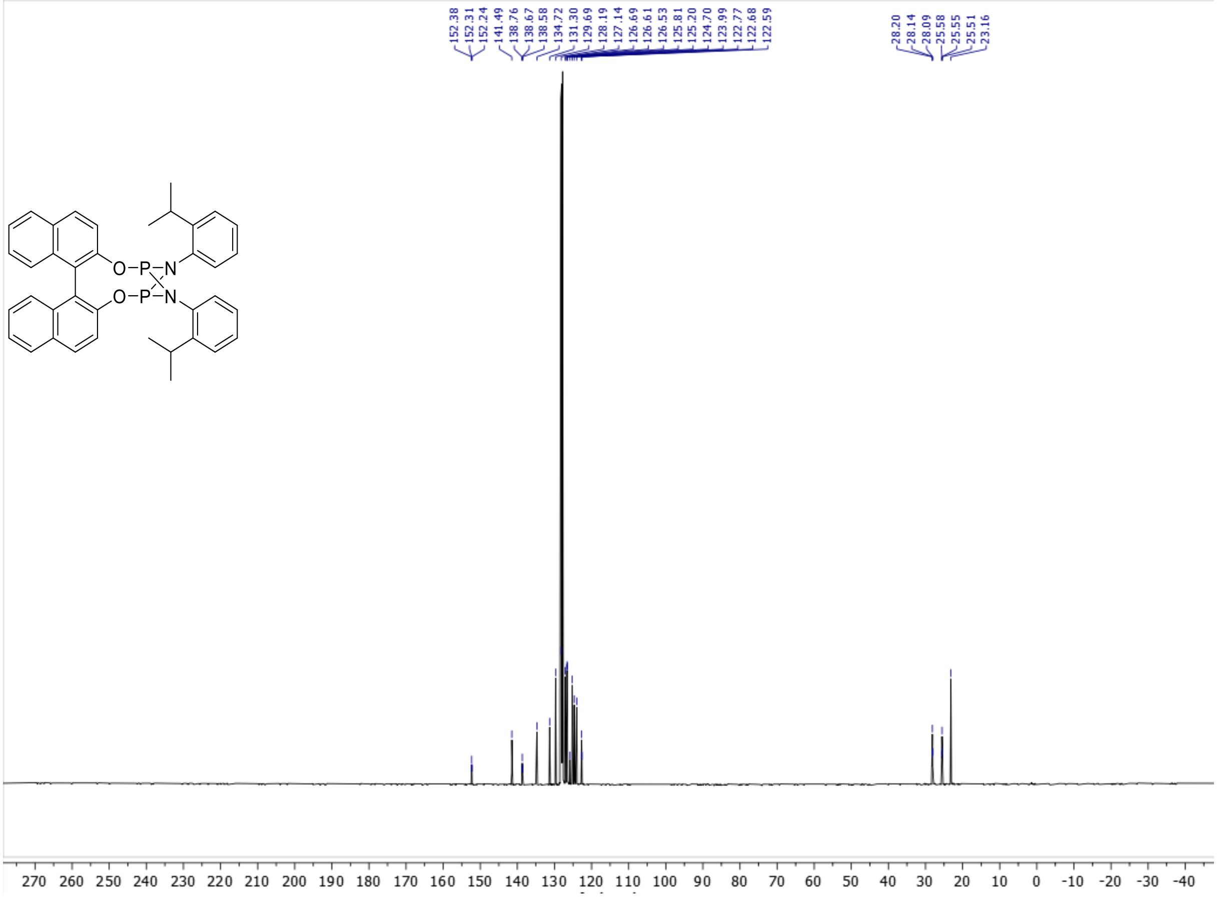
152.38
152.31
152.24
141.49
138.76
138.67
138.58
134.72
131.30
129.69
128.19
127.14
126.69
126.61
126.53
125.81
125.20
124.70
123.99
122.77
122.68
122.59
28.20
28.14
28.09
25.58
25.55
25.51
23.16
O
P
N
O
P
N
270
260
250
240
230
220
210
200
190
180
170
160
150
140
130
120
110
100
90
80
70
60
50
40
30
20
10
0
-10
-20
-30
-40

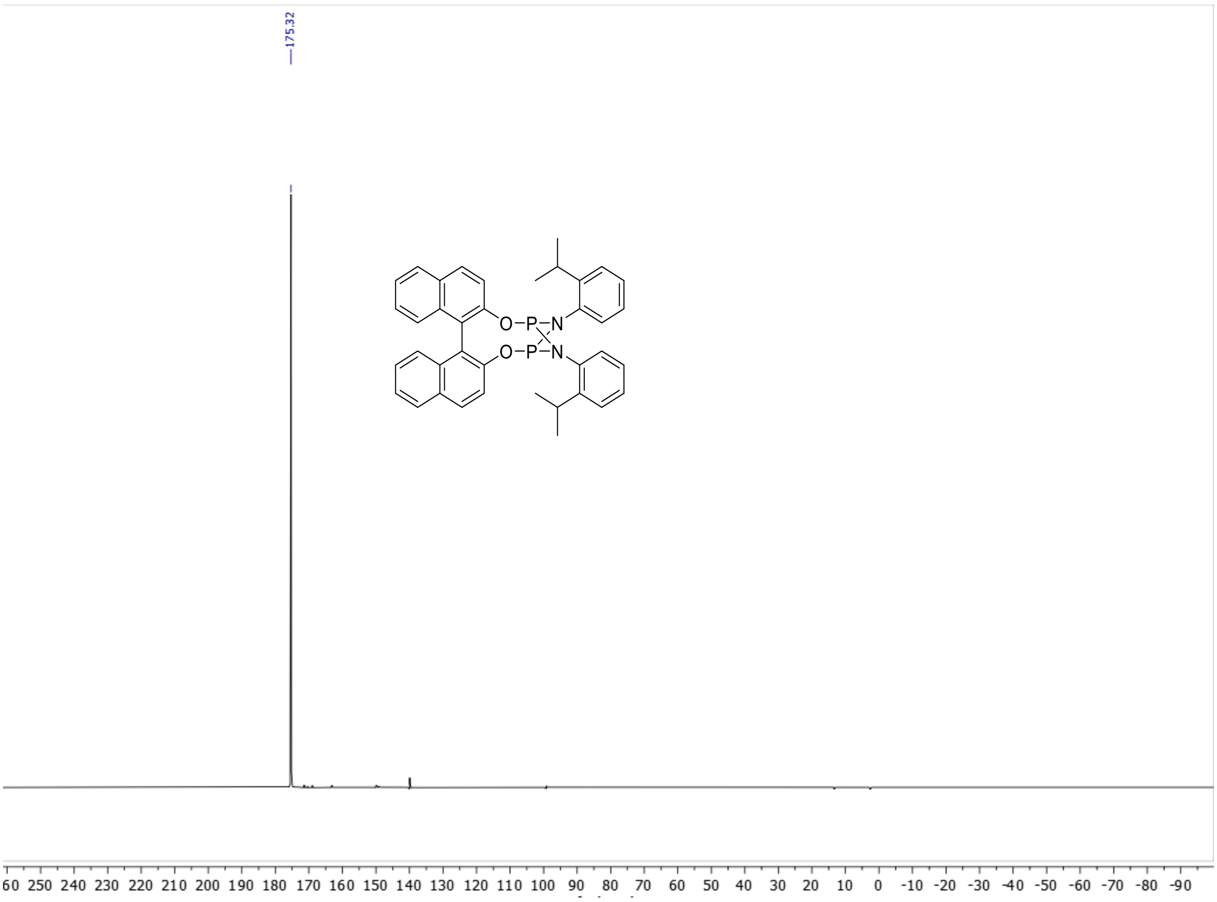
175.32
O
P
N
O
P
N
60 250 240 230 220 210 200 190 180 170 160 150 140 130 120 110 100 90 80 70 60 50 40 30 20 10 0 -10 -20 -30 -40 -50 -60 -70 -80 -90

SI-L29

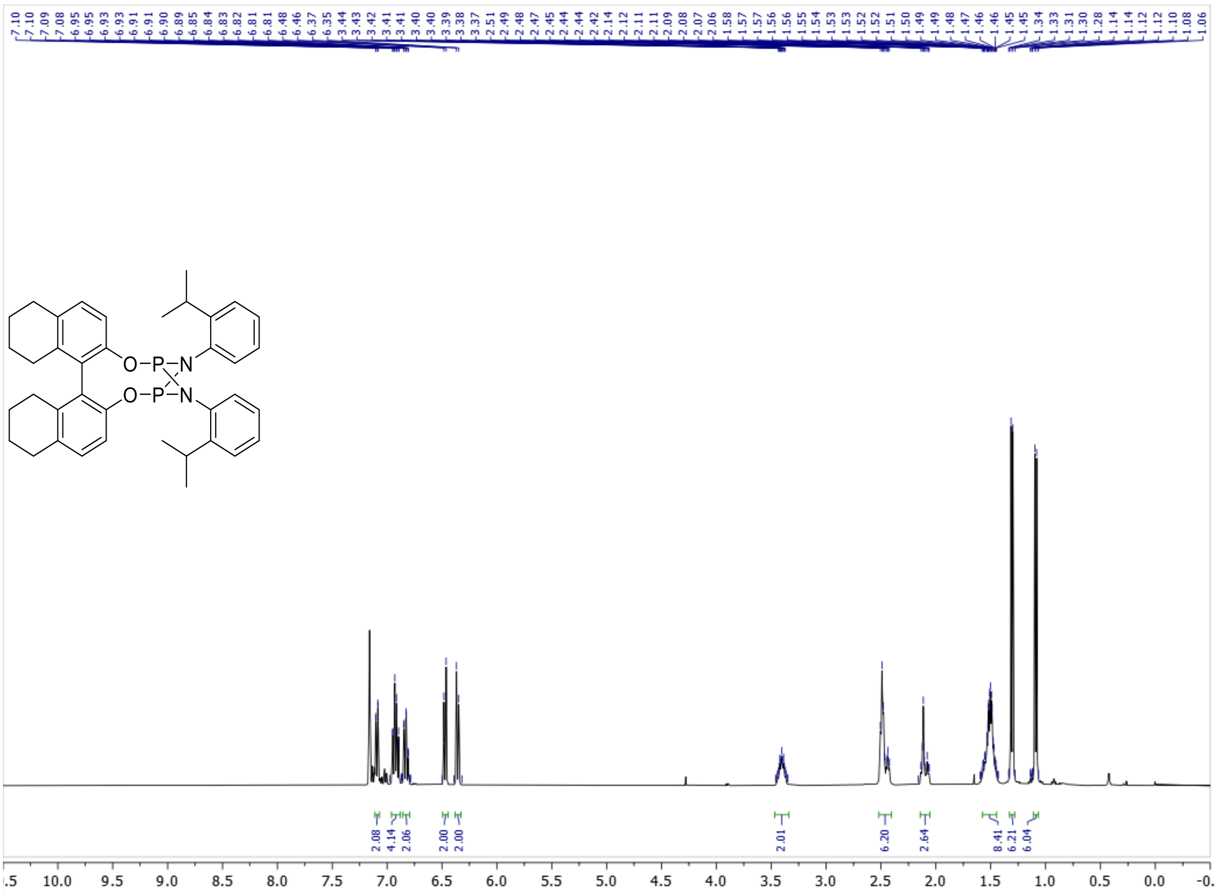

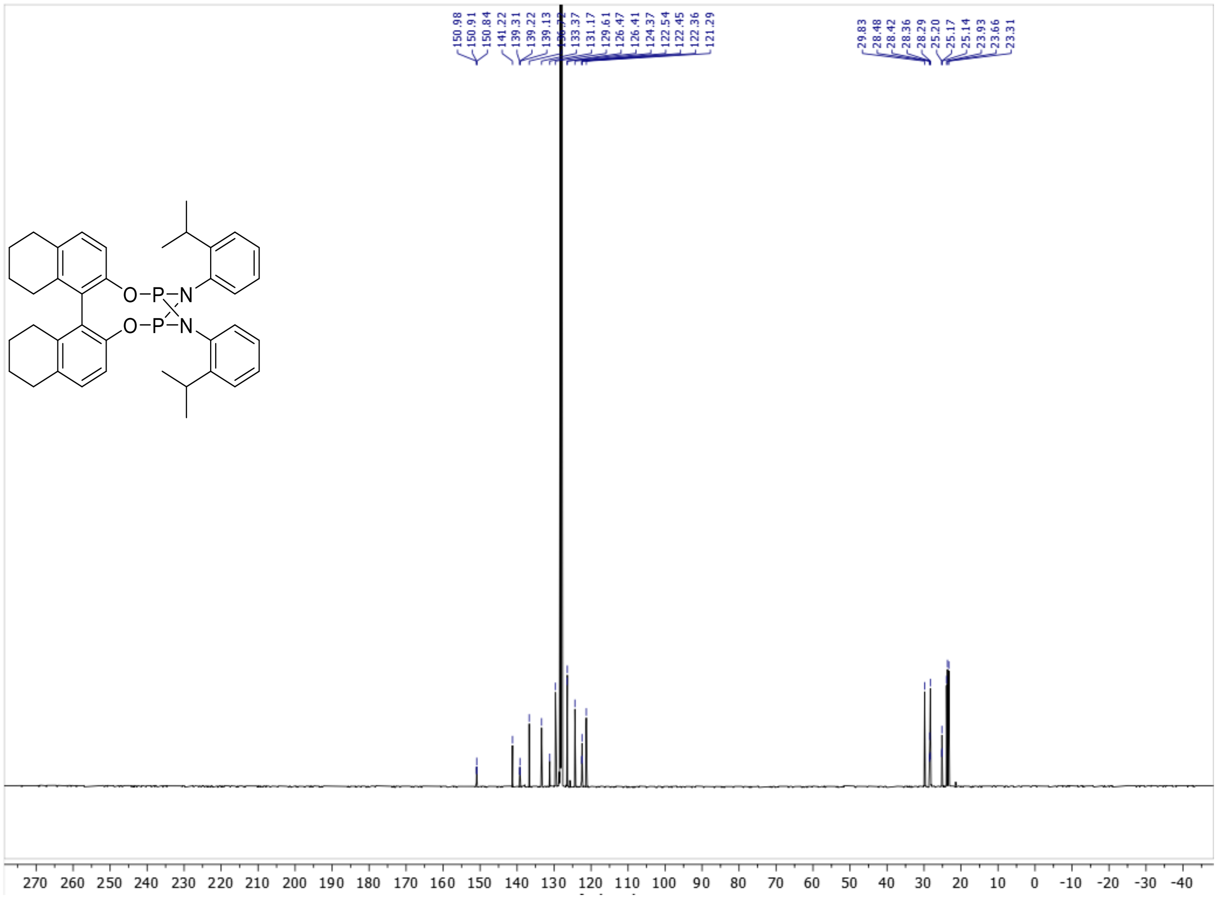

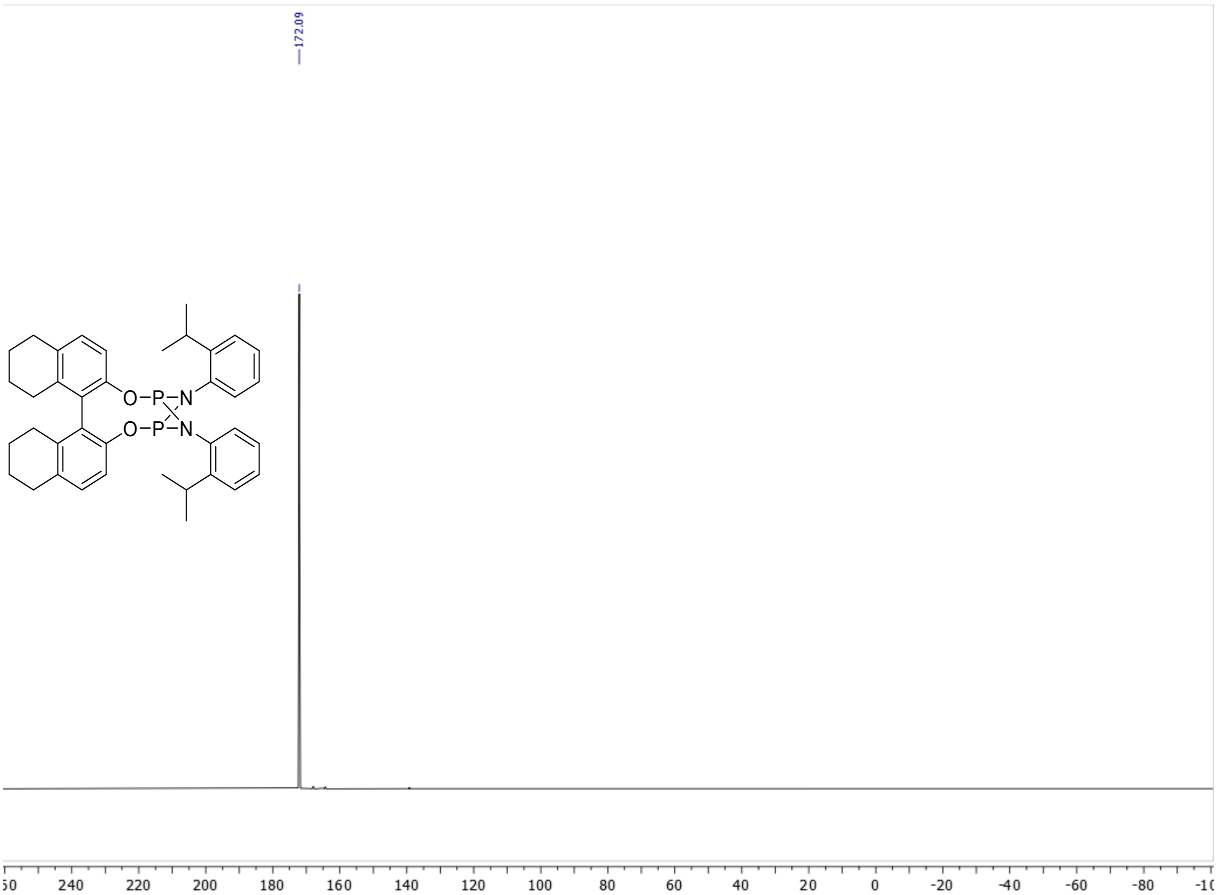
172.09
O
P
N
O
P
N
240
220
200
180
160
140
120
100
80
60
40
20
0
-20
-40
-60
-80

SI-L30

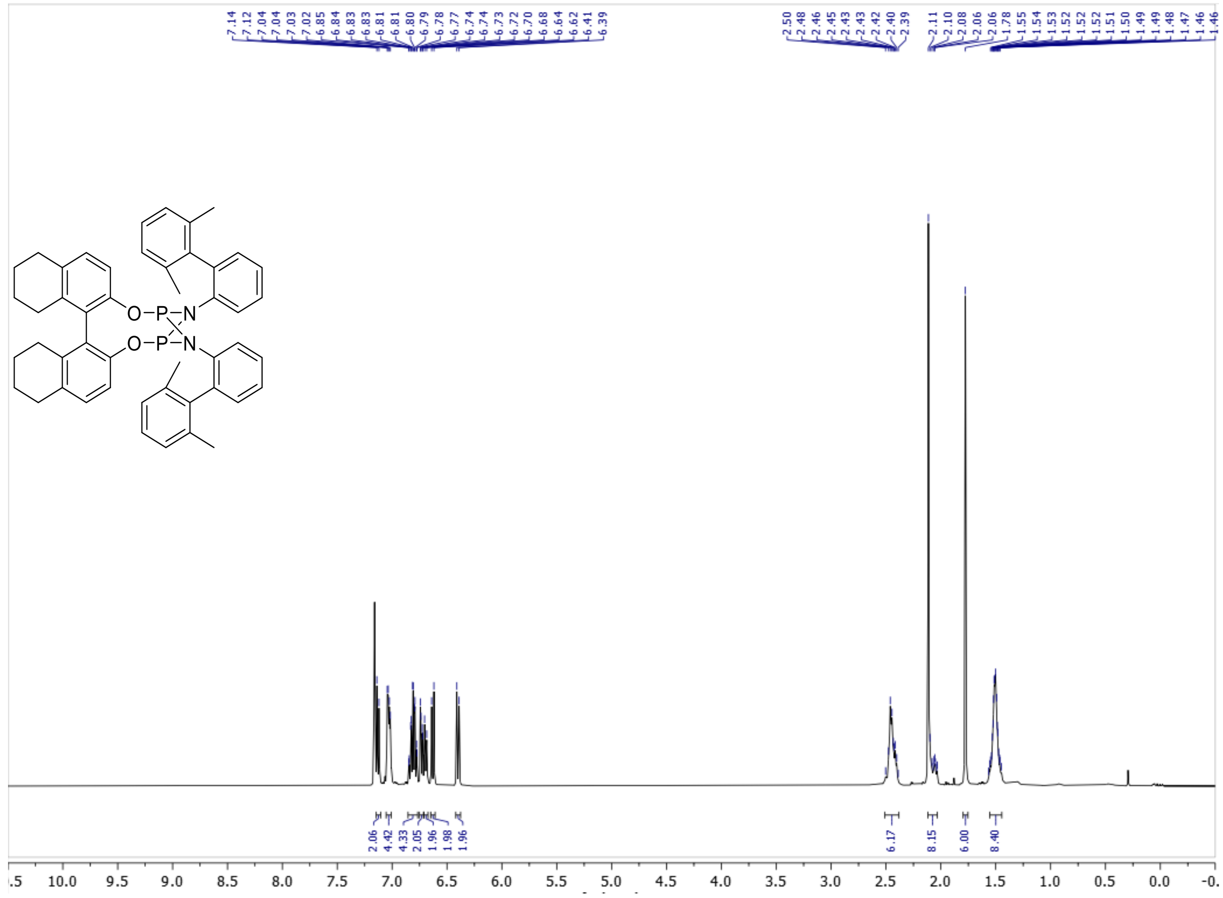

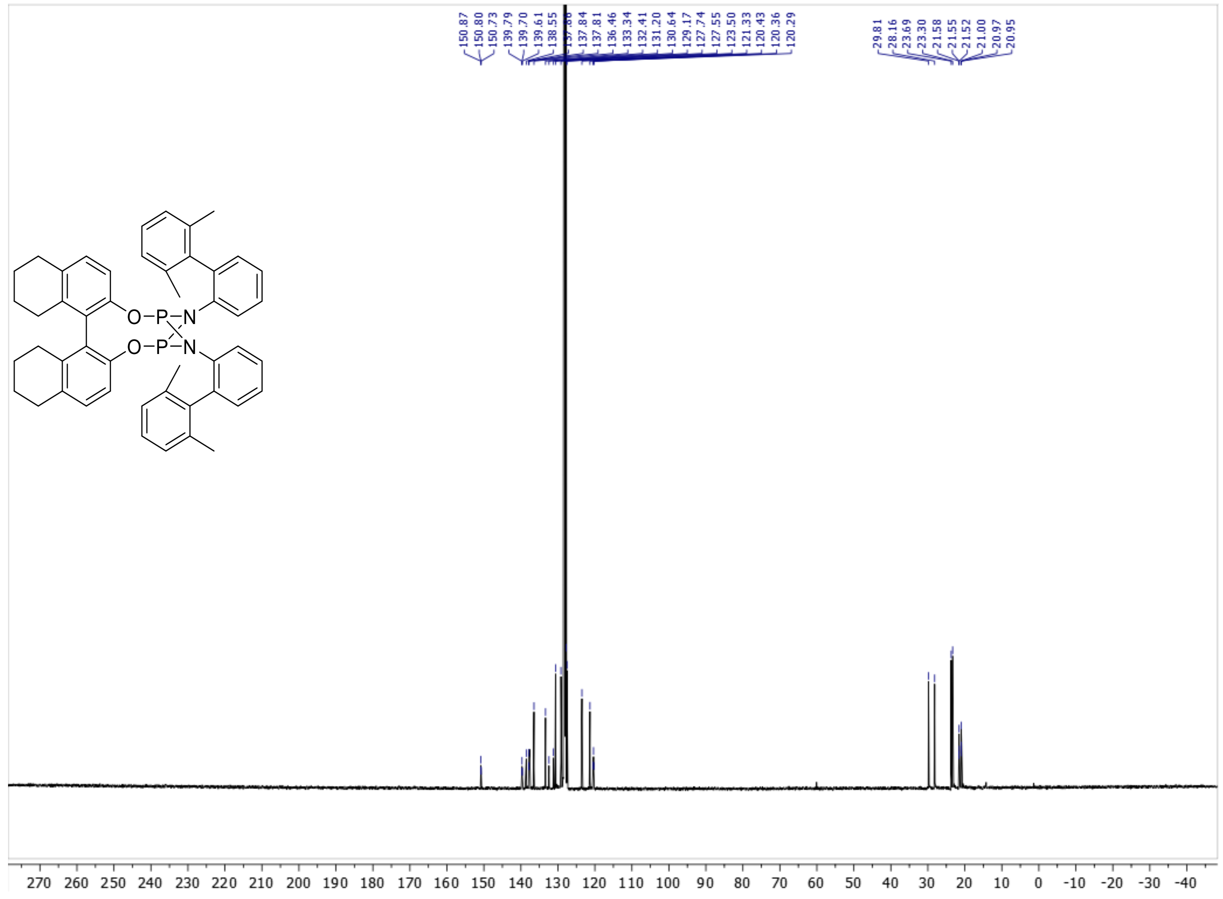

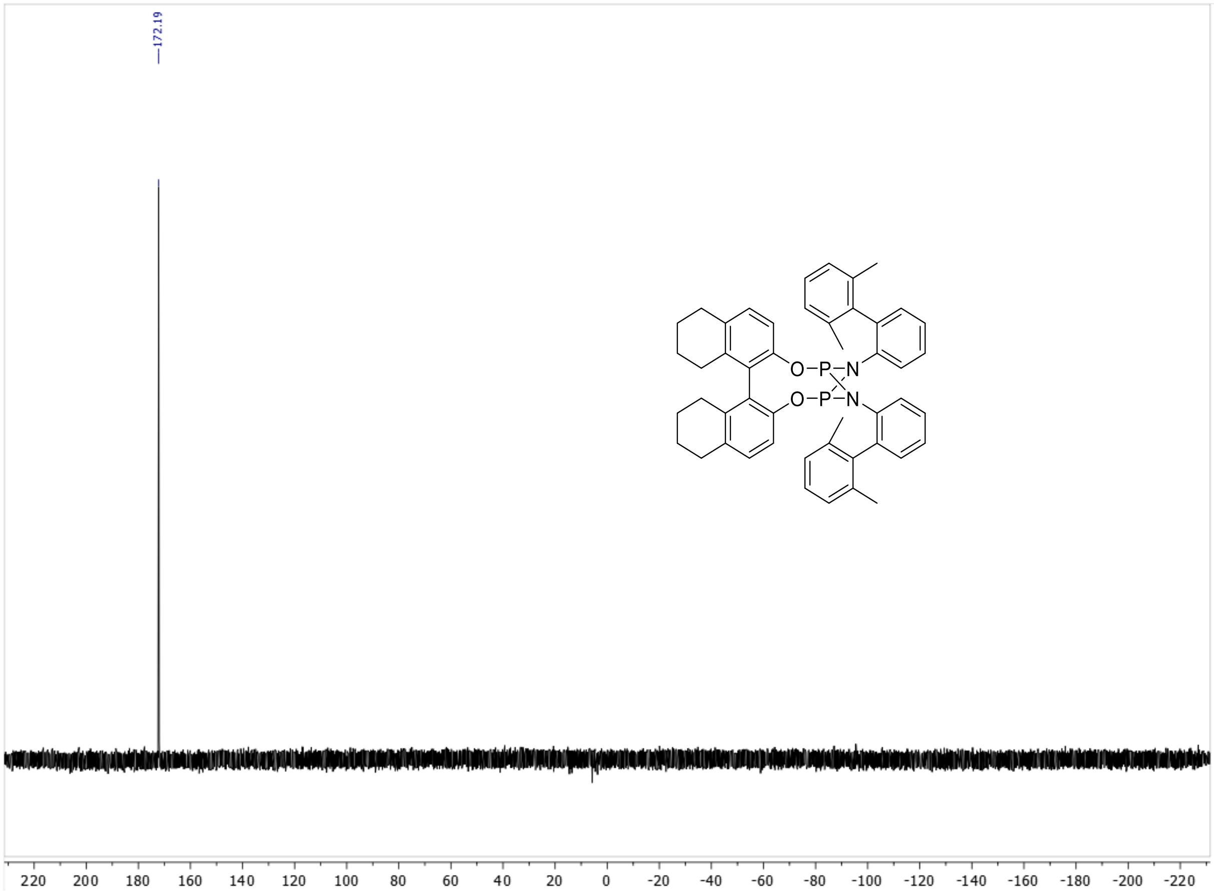
172.19
O P N
O P N
220 200 180 160 140 120 100 80 60 40 20 0 -20 -40 -60 -80 -100 -120 -140 -160 -180 -200 -220

SI-L31

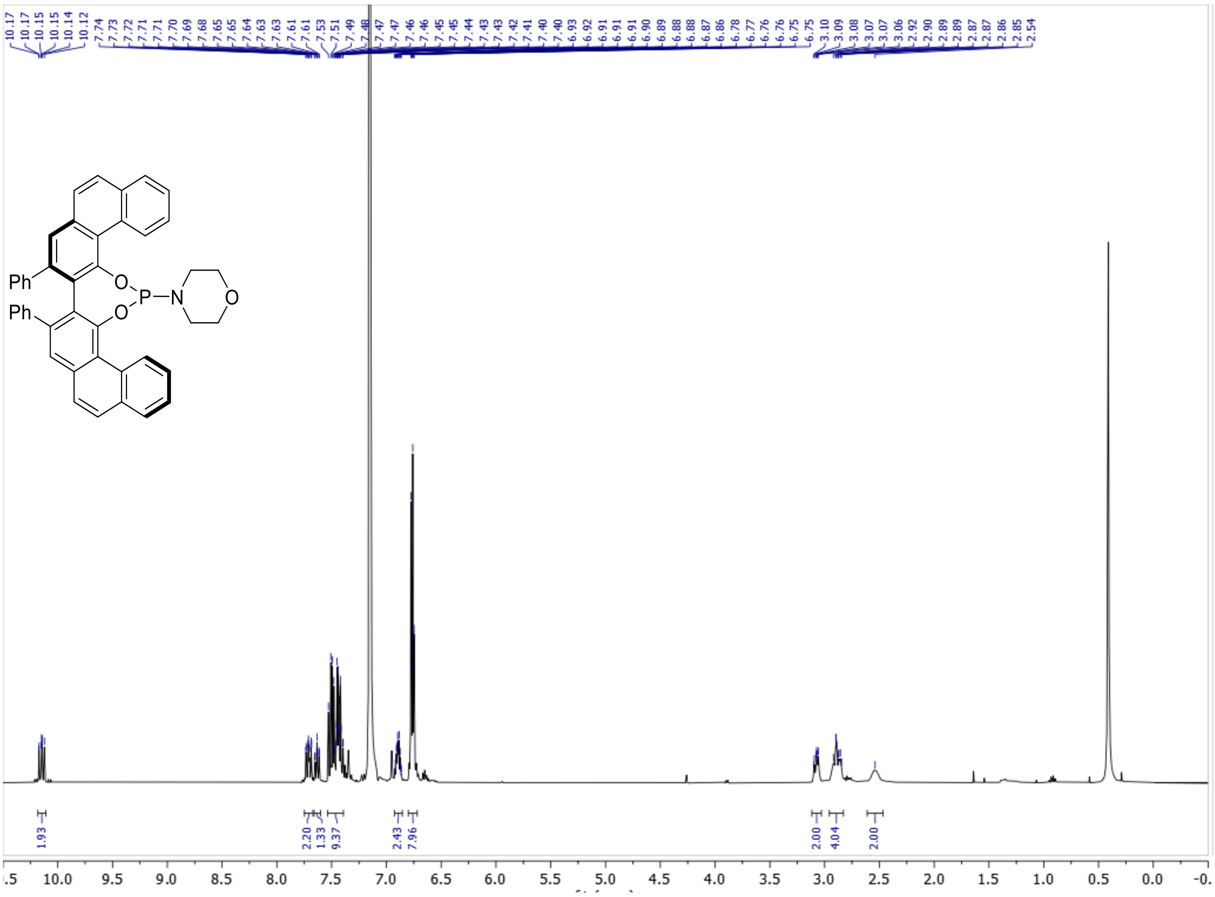

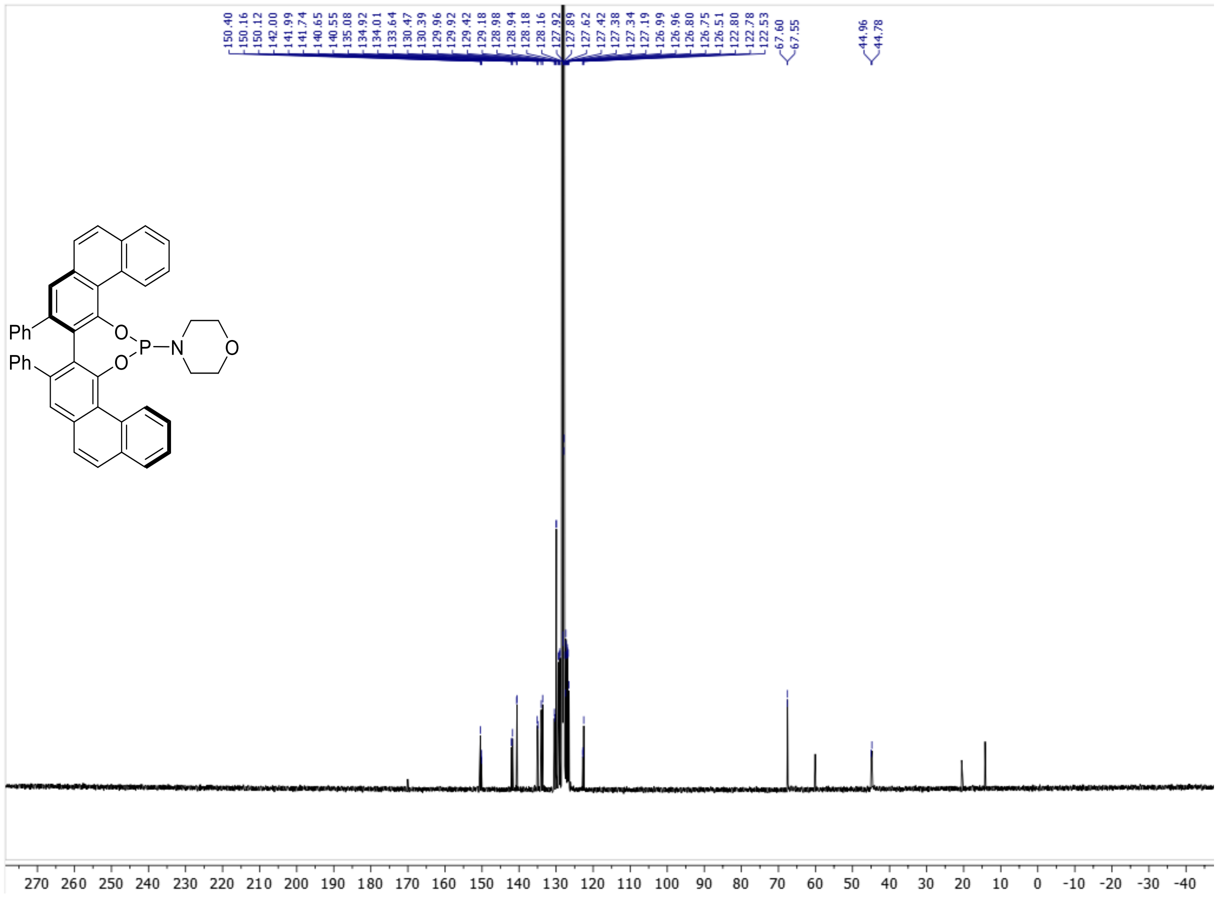

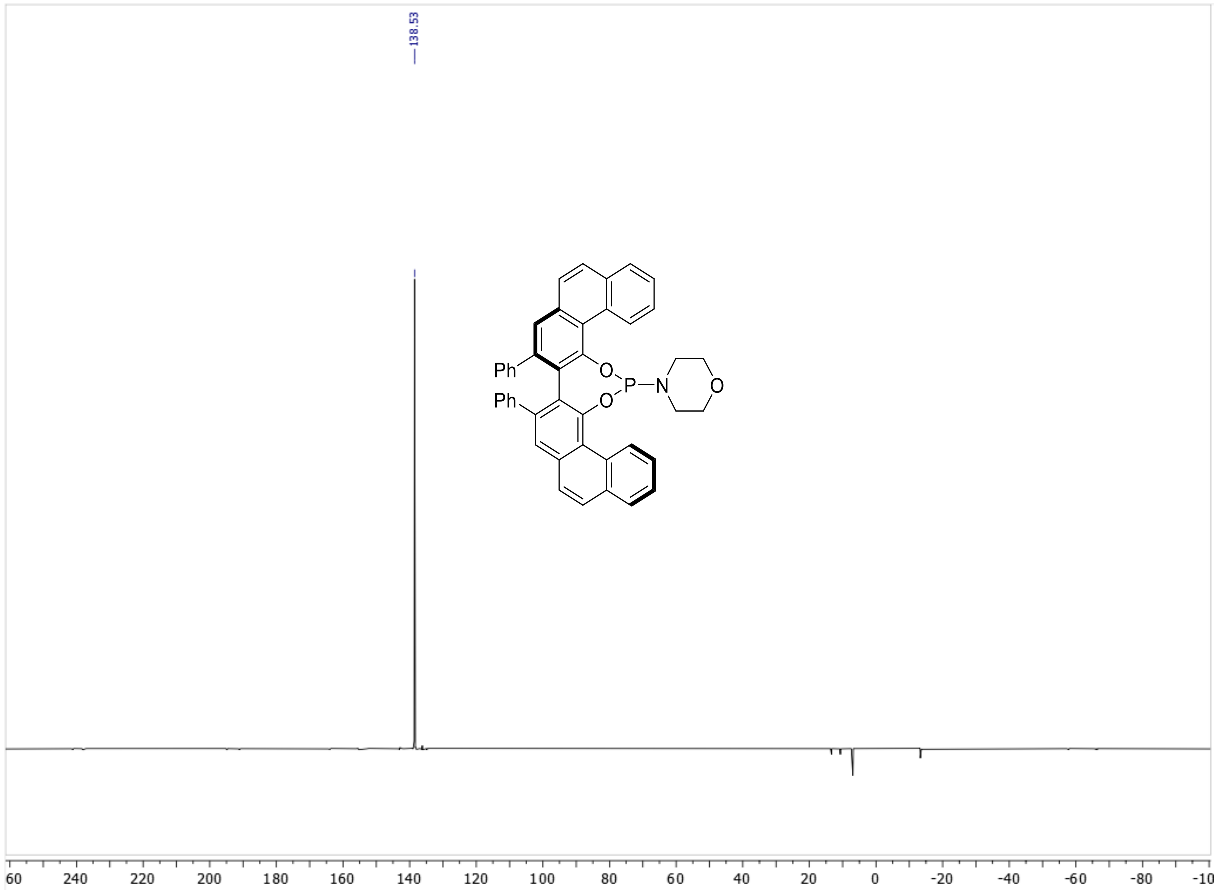
138.53
Ph
Ph
O
P
N
O
O
60
240
220
200
180
160
140
120
100
80
60
40
20
0
-20
-40
-60
-80
-10

SI-L32

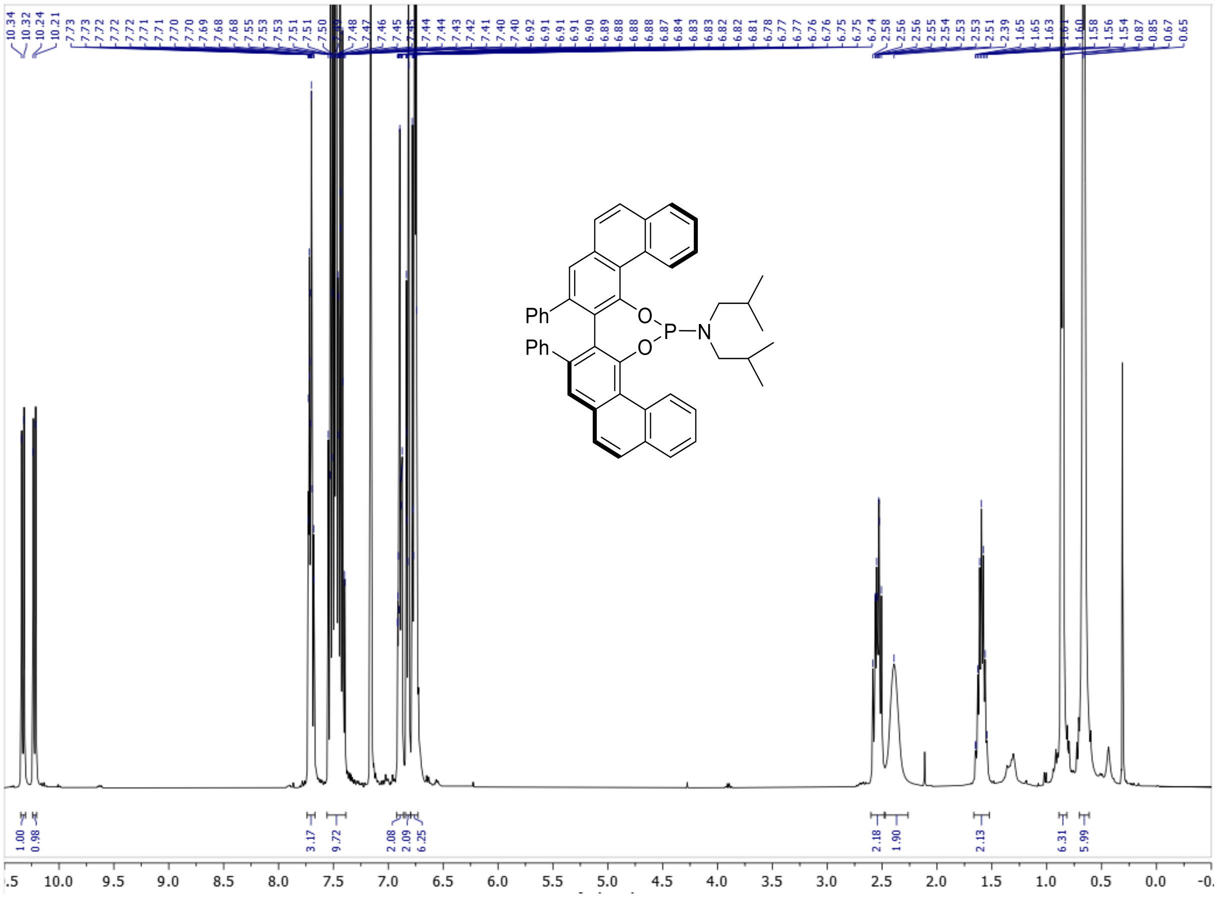

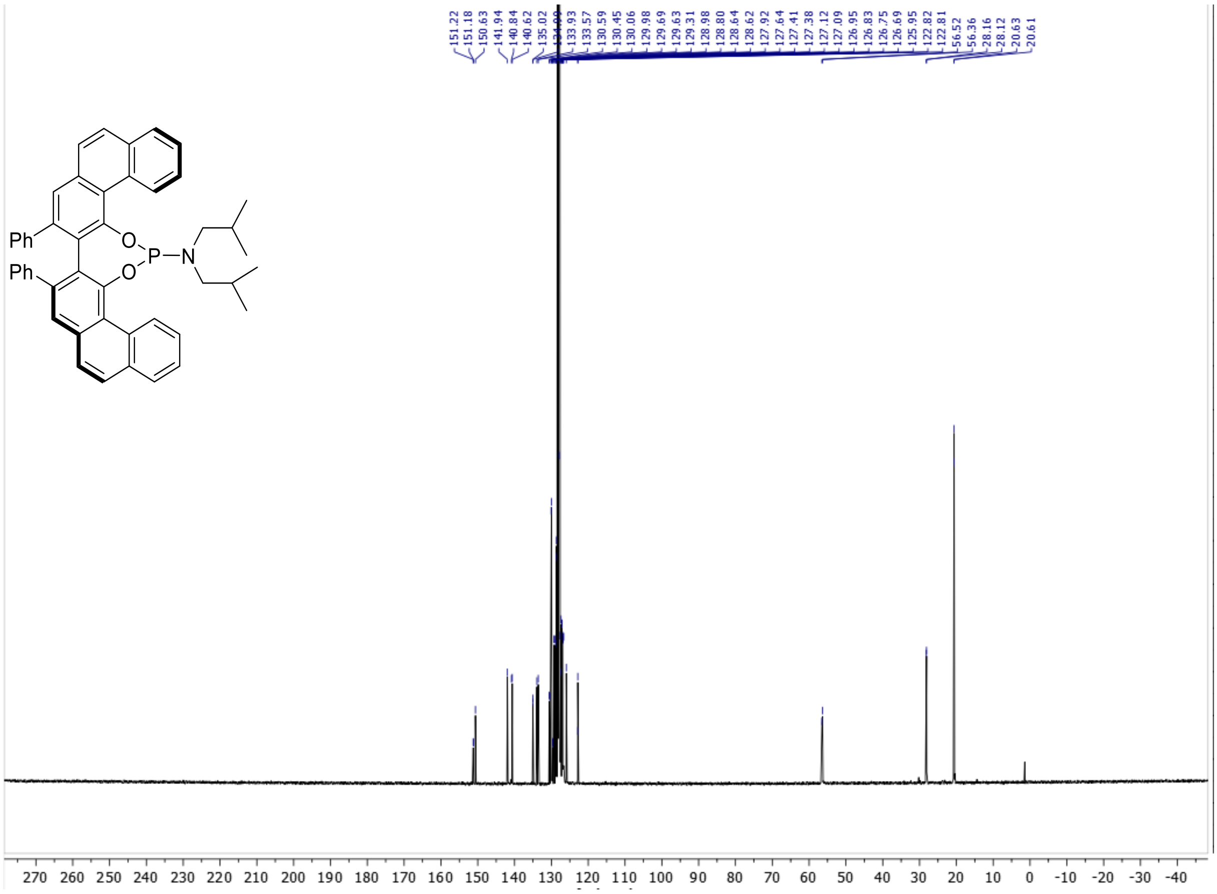

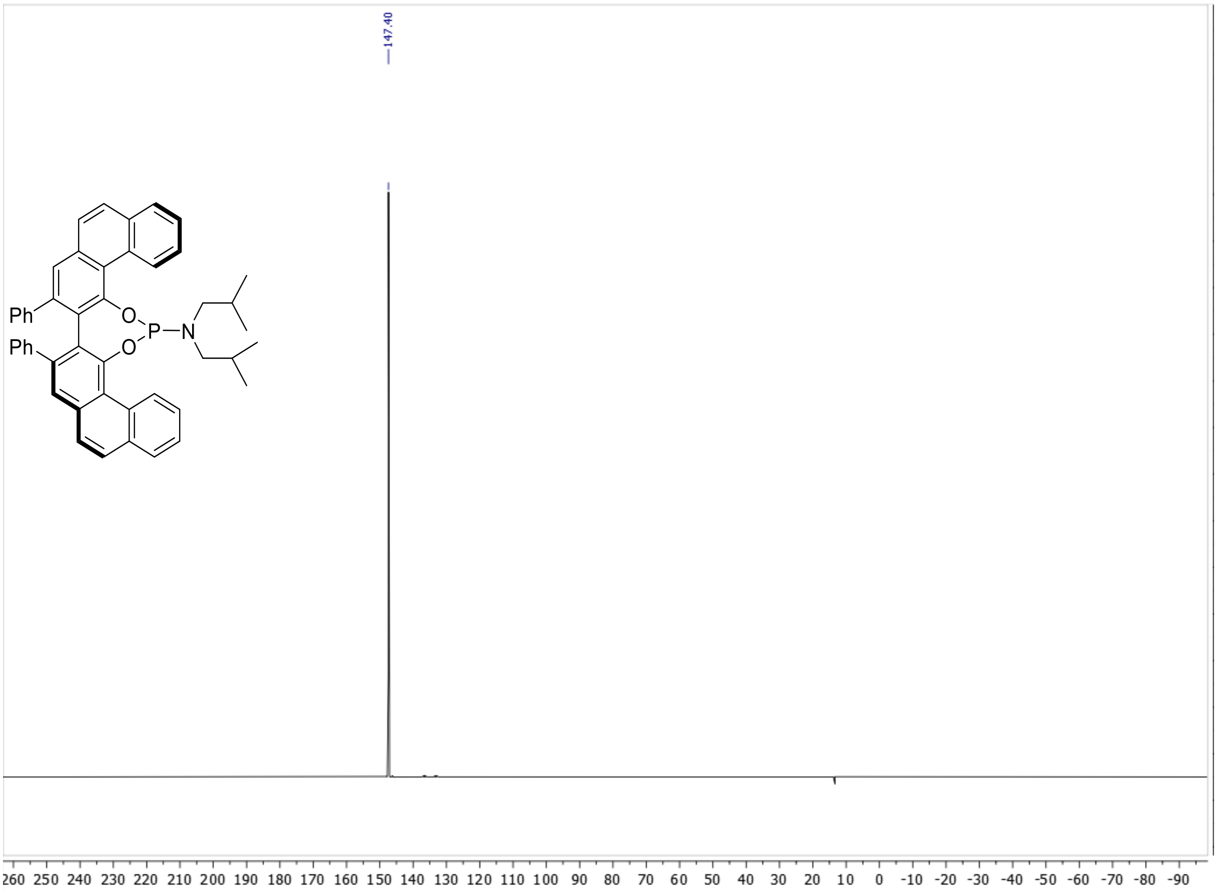
147.40
Ph
Ph
O
O
P
N
260 250 240 230 220 210 200 190 180 170 160 150 140 130 120 110 100 90 80 70 60 50 40 30 20 10 0 -10 -20 -30 -40 -50 -60 -70 -80 -90

SI-L33

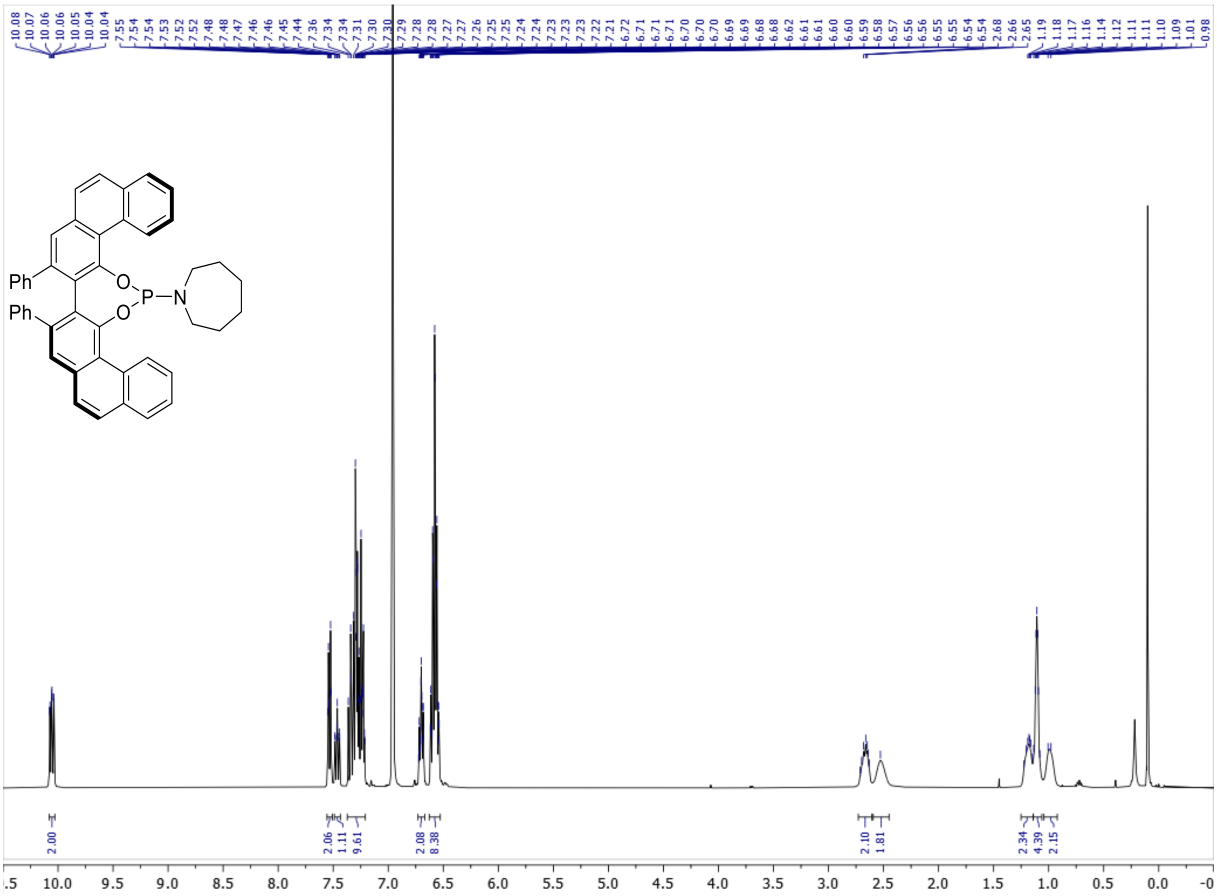
10.08 10.07 10.06 10.06 10.05 10.04 10.04 7.55 7.54 7.54 7.53 7.52 7.52 7.48 7.47 7.46 7.46 7.45 7.44 7.36 7.34 7.34 7.31 7.30 7.30 7.29 7.28 7.28 7.27 7.27 7.26 7.25 7.25 7.24 7.24 7.23 7.23 7.23 7.22 7.21 6.72 6.71 6.71 6.71 6.70 6.70 6.69 6.69 6.68 6.68 6.62 6.61 6.61 6.60 6.59 6.58 6.57 6.56 6.56 6.55 6.55 6.54 6.54 2.68 2.66 2.65 1.19 1.18 1.17 1.16 1.14 1.12 1.11 1.11 1.10 1.09 1.01 0.98
Ph
Ph
O
O
P
N
2.00
2.06
1.11
9.61
2.08
8.38
2.10
1.81
2.34
4.39
2.15
.5 10.0 9.5 9.0 8.5 8.0 7.5 7.0 6.5 6.0 5.5 5.0 4.5 4.0 3.5 3.0 2.5 2.0 1.5 1.0 0.5 0.0 -0

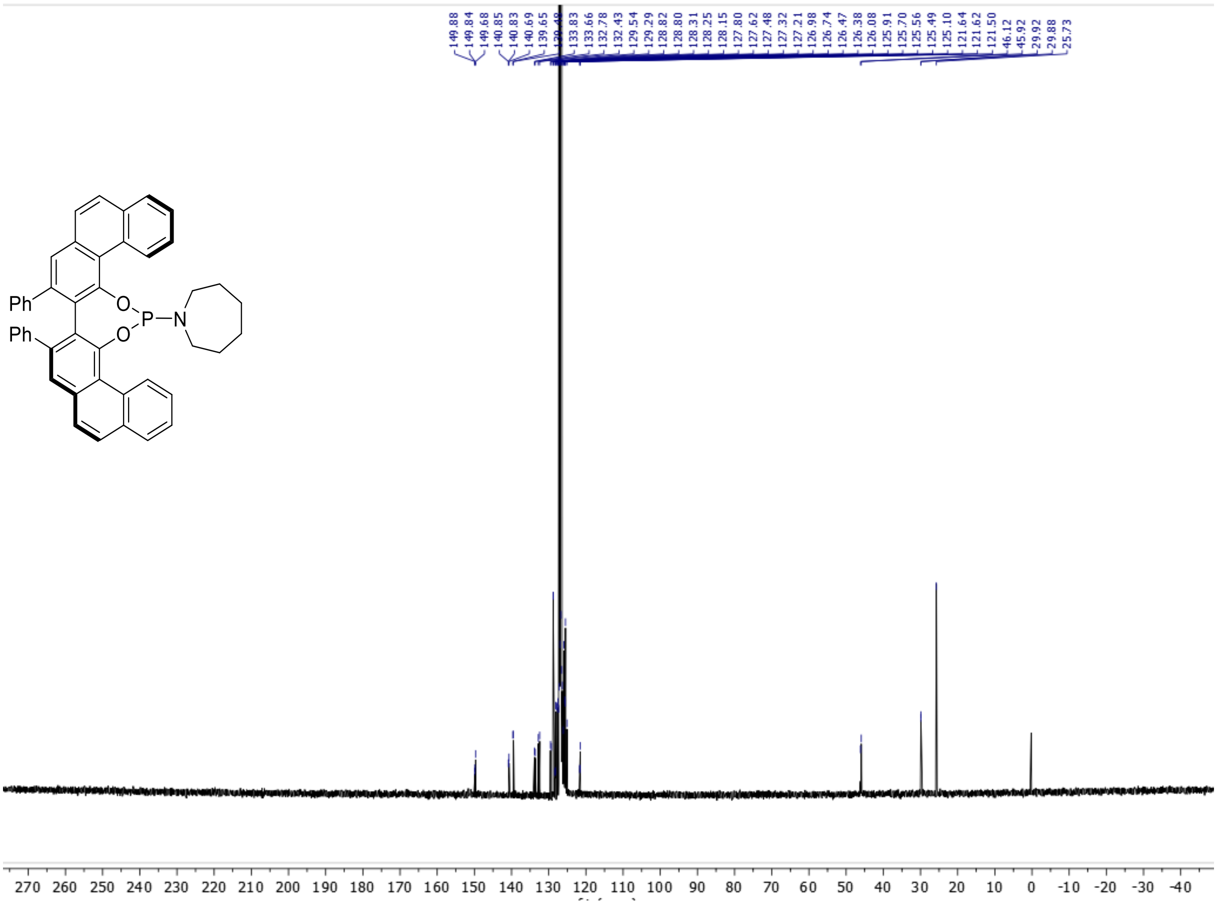
149.88 149.84 149.68 140.85 140.83 140.69 139.65 133.83 133.66 132.78 132.43 129.54 129.29 128.82 128.80 128.31 128.25 128.15 127.80 127.62 127.48 127.32 127.21 126.98 126.74 126.47 126.38 126.08 125.91 125.70 125.56 125.49 125.10 121.64 121.62 121.50 46.12 45.92 29.92 29.88 25.73
Ph
Ph
O
O
P
N
270 260 250 240 230 220 210 200 190 180 170 160 150 140 130 120 110 100 90 80 70 60 50 40 30 20 10 0 -10 -20 -30 -40

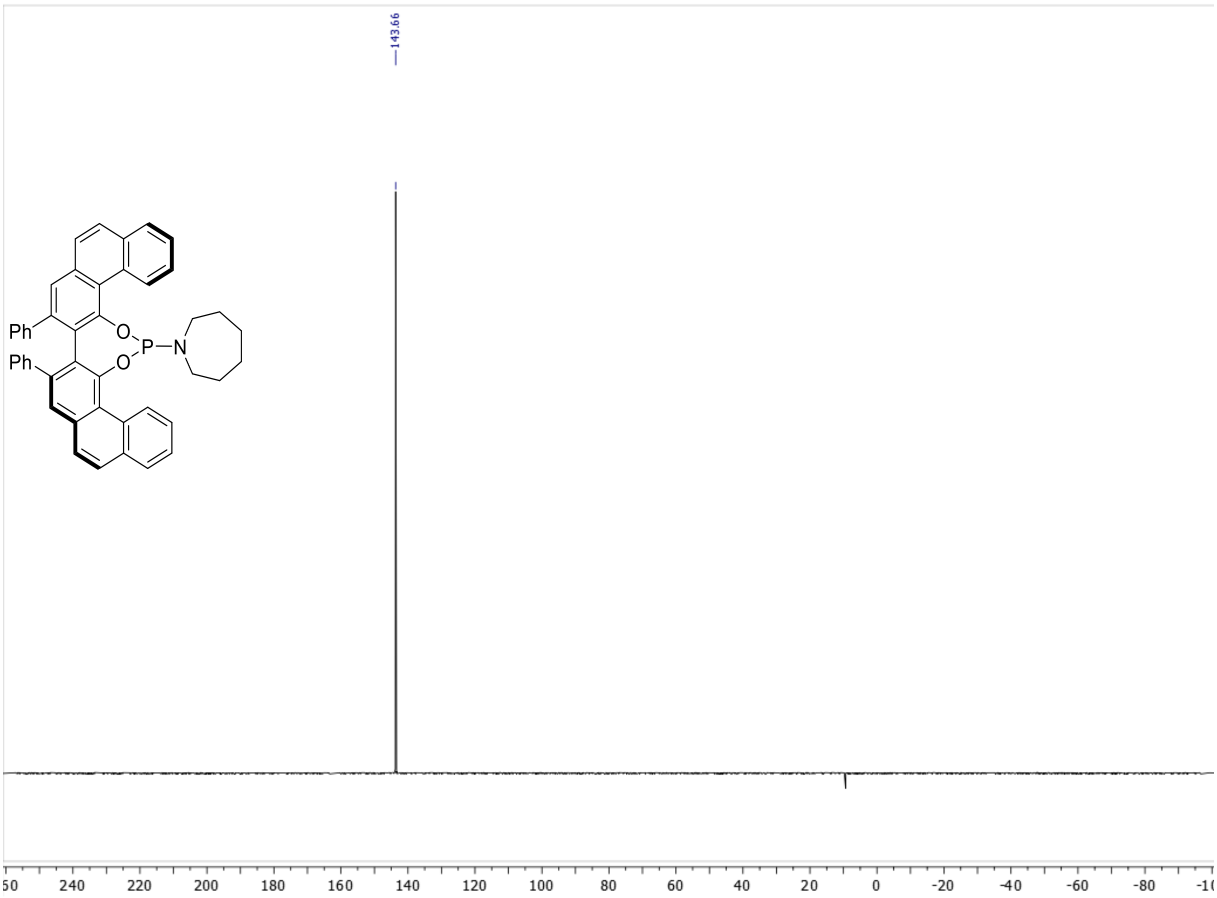

143.66
Ph
Ph
O
O
P
N
50
240
220
200
180
160
140
120
100
80
60
40
20
0
-20
-40
-60
-80
-1(

SI-L34

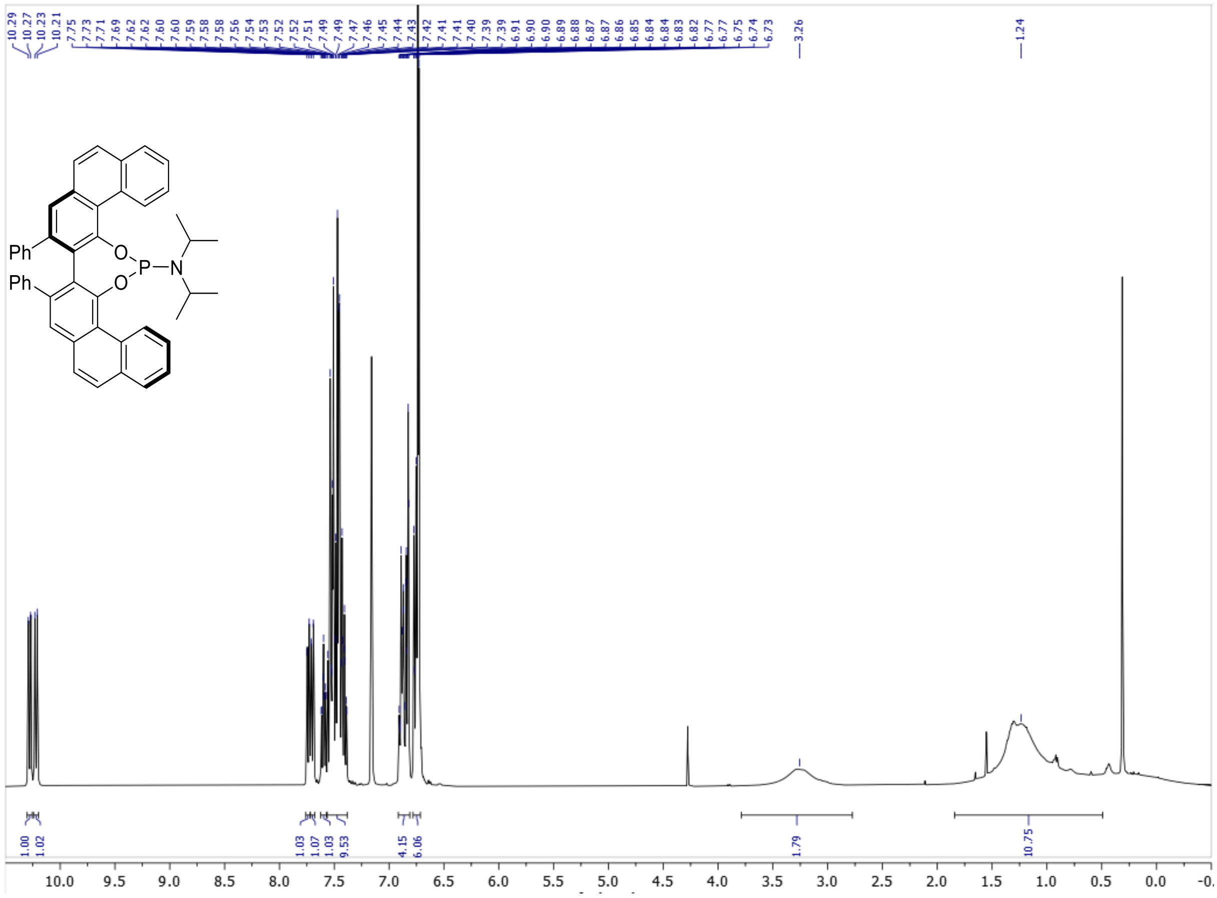

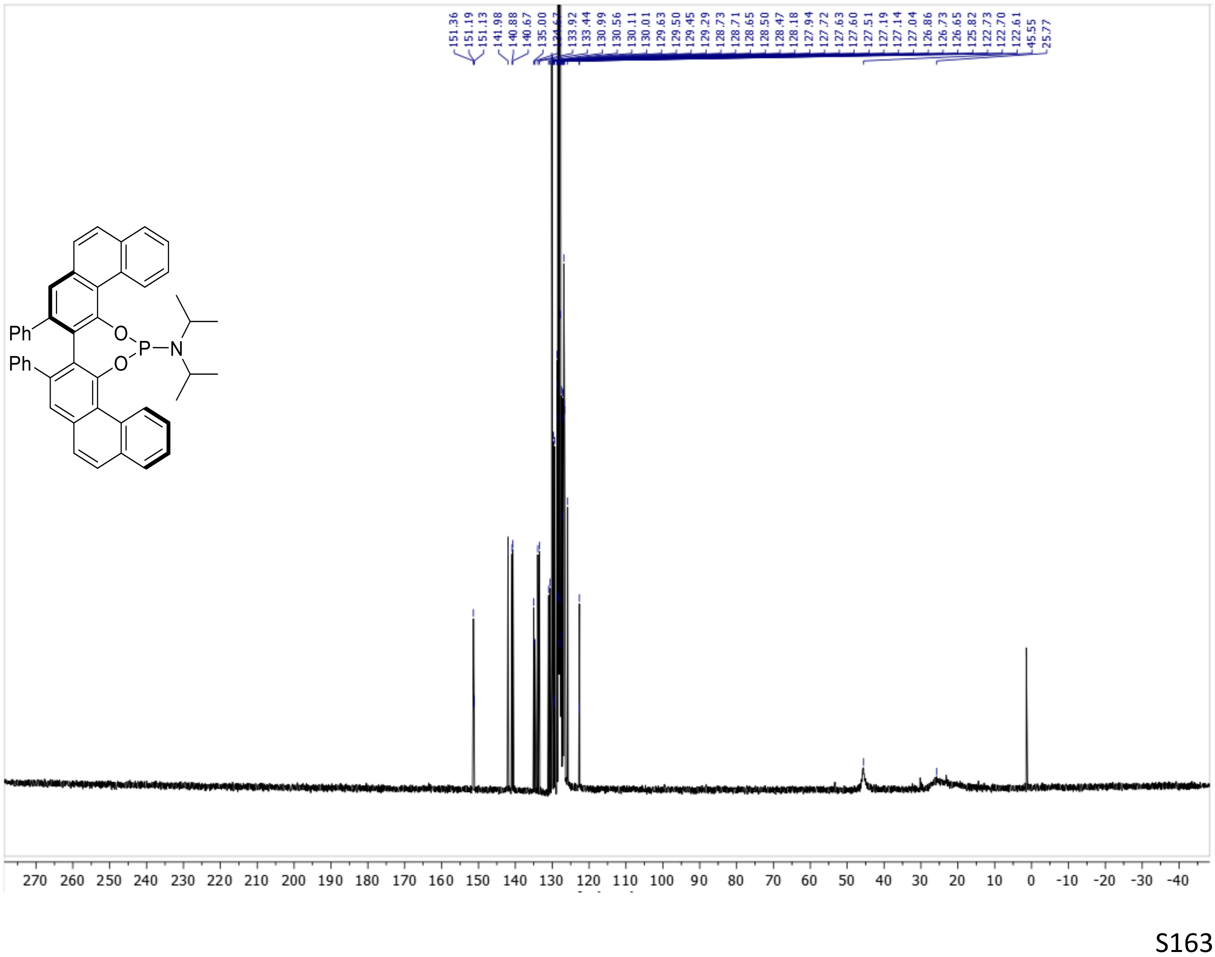

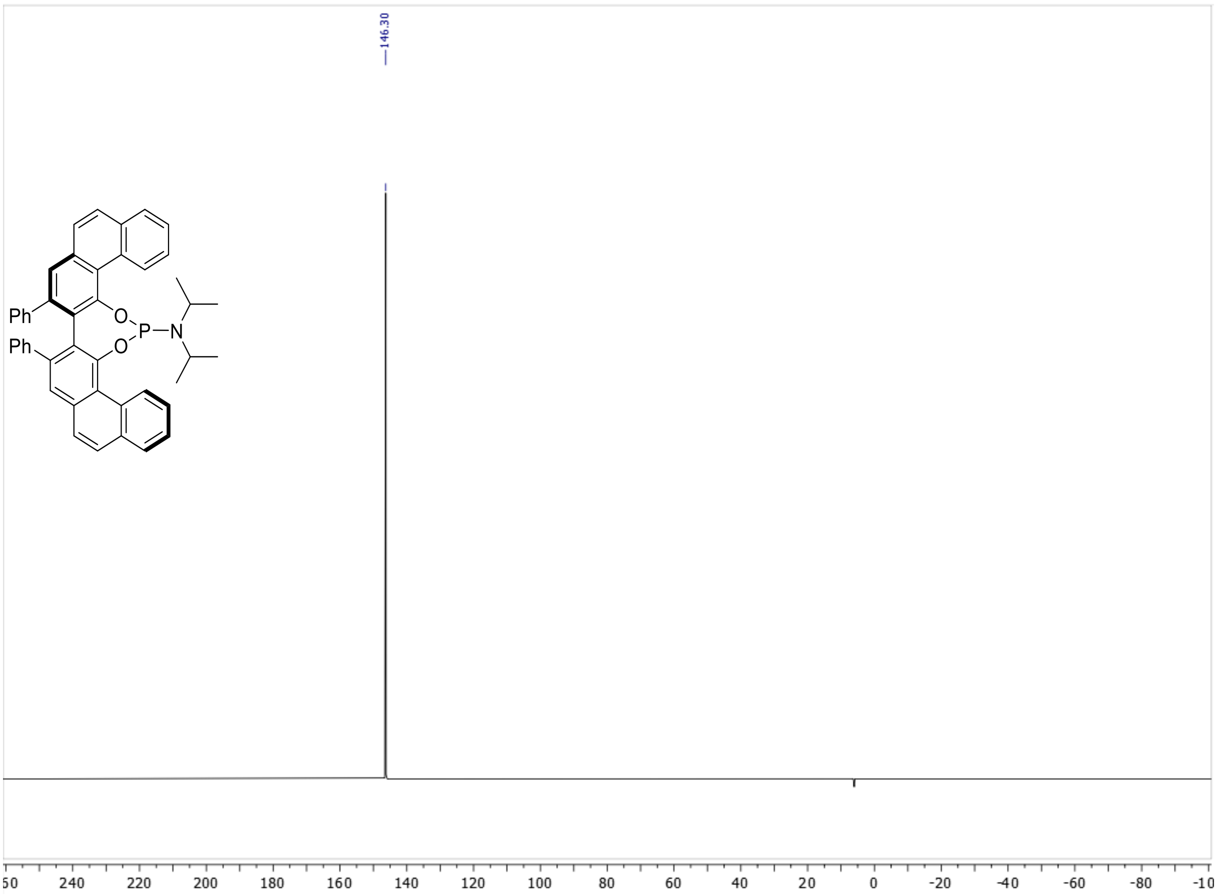
146.30
Ph
Ph
O
O
P
N
50
240
220
200
180
160
140
120
100
80
60
40
20
0
-20
-40
-60
-80
-10

SI-L35

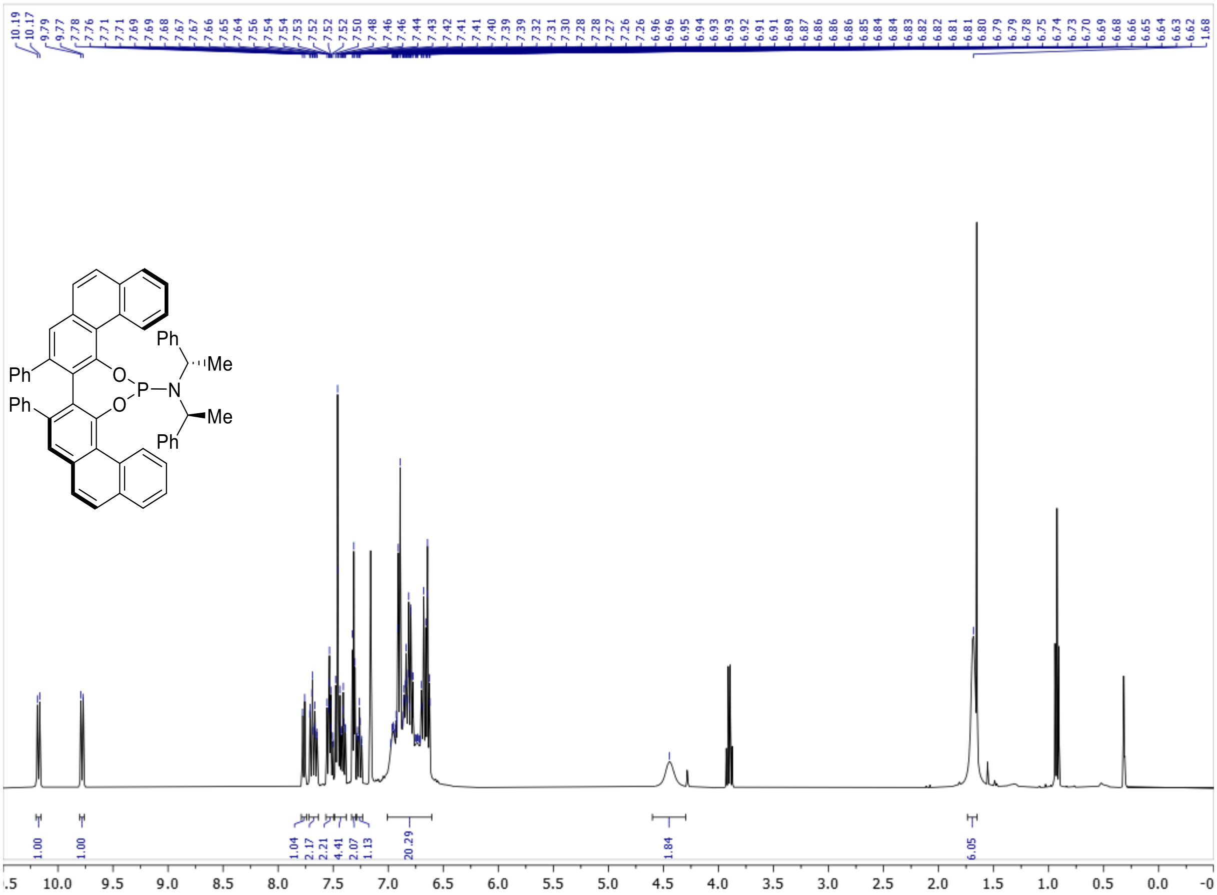

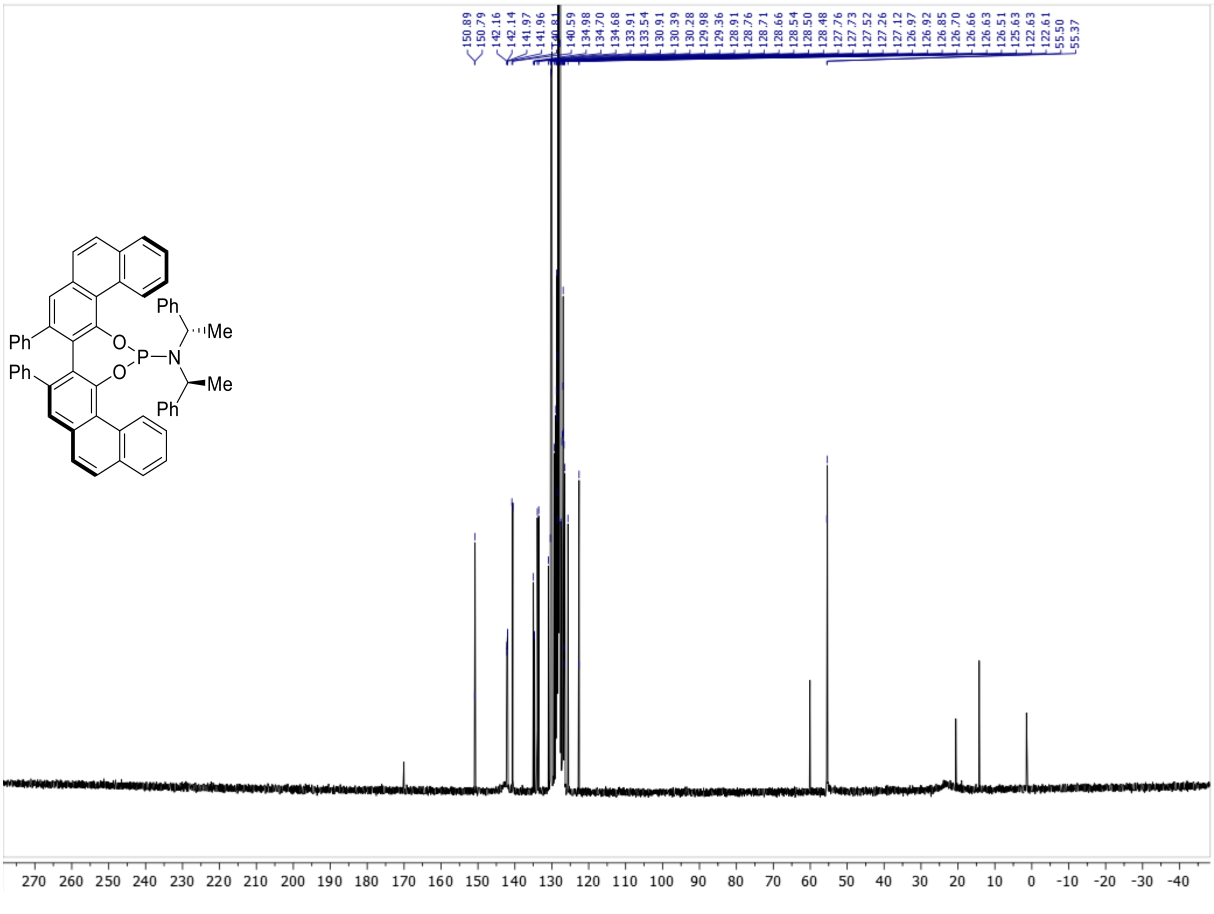

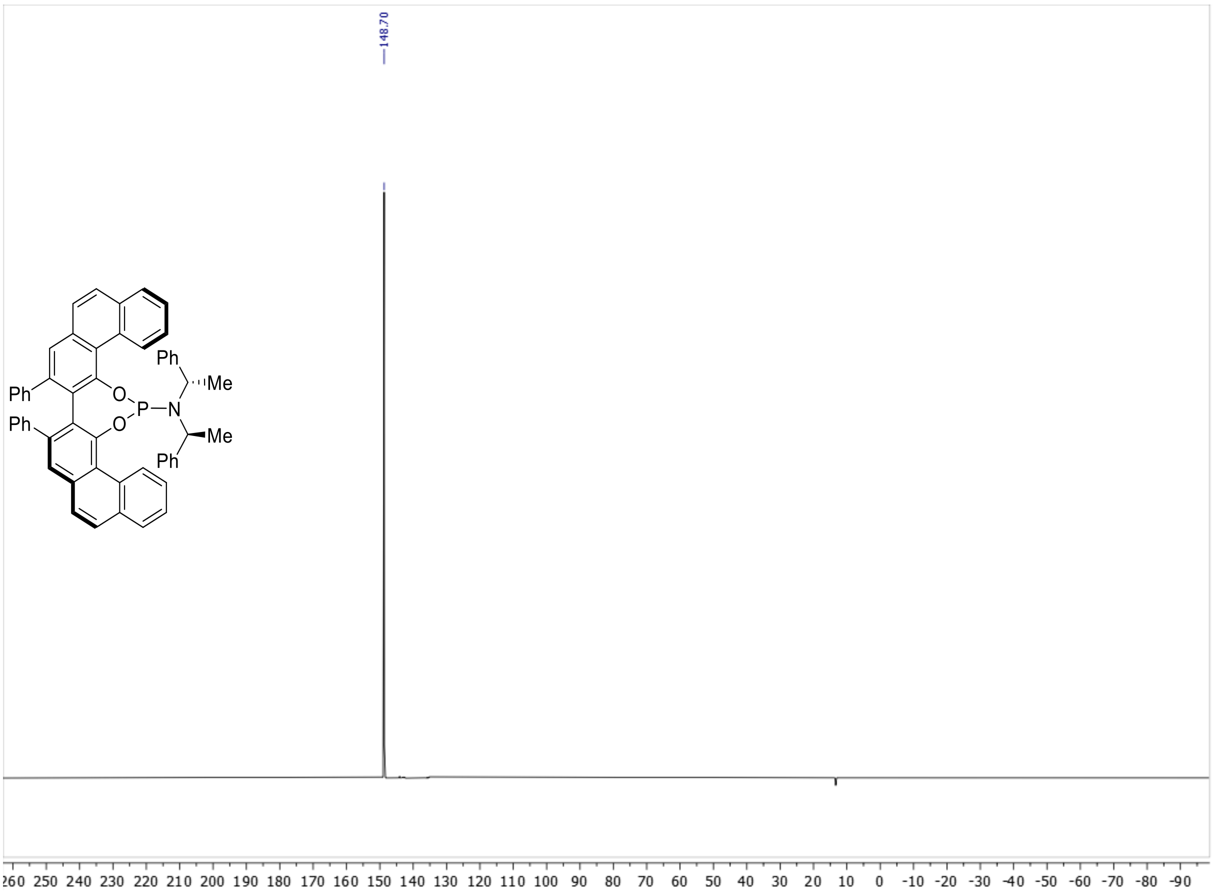

148.70
Ph
Me
Ph
O
P
N
Ph
O
Me
Ph
260 250 240 230 220 210 200 190 180 170 160 150 140 130 120 110 100 90 80 70 60 50 40 30 20 10 0 -10 -20 -30 -40 -50 -60 -70 -80 -90

SI-L36

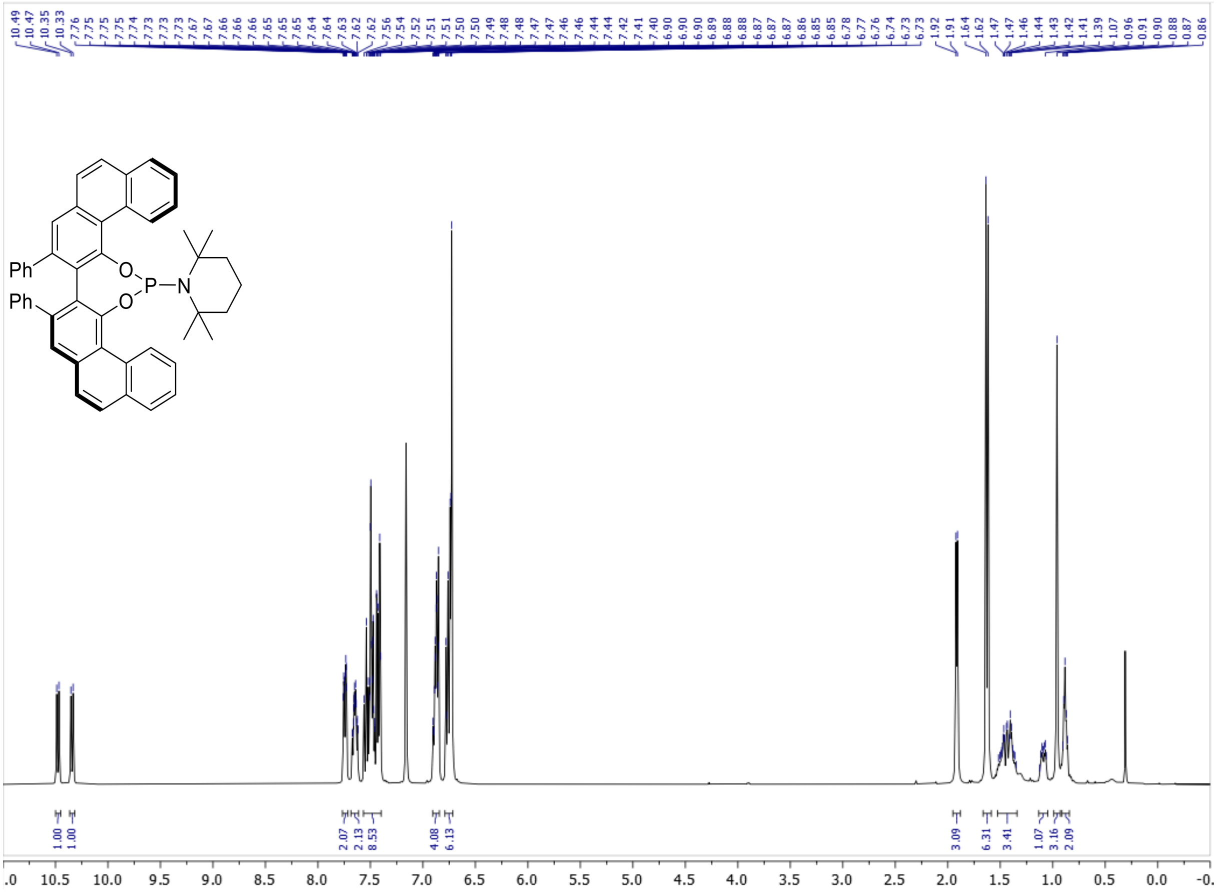

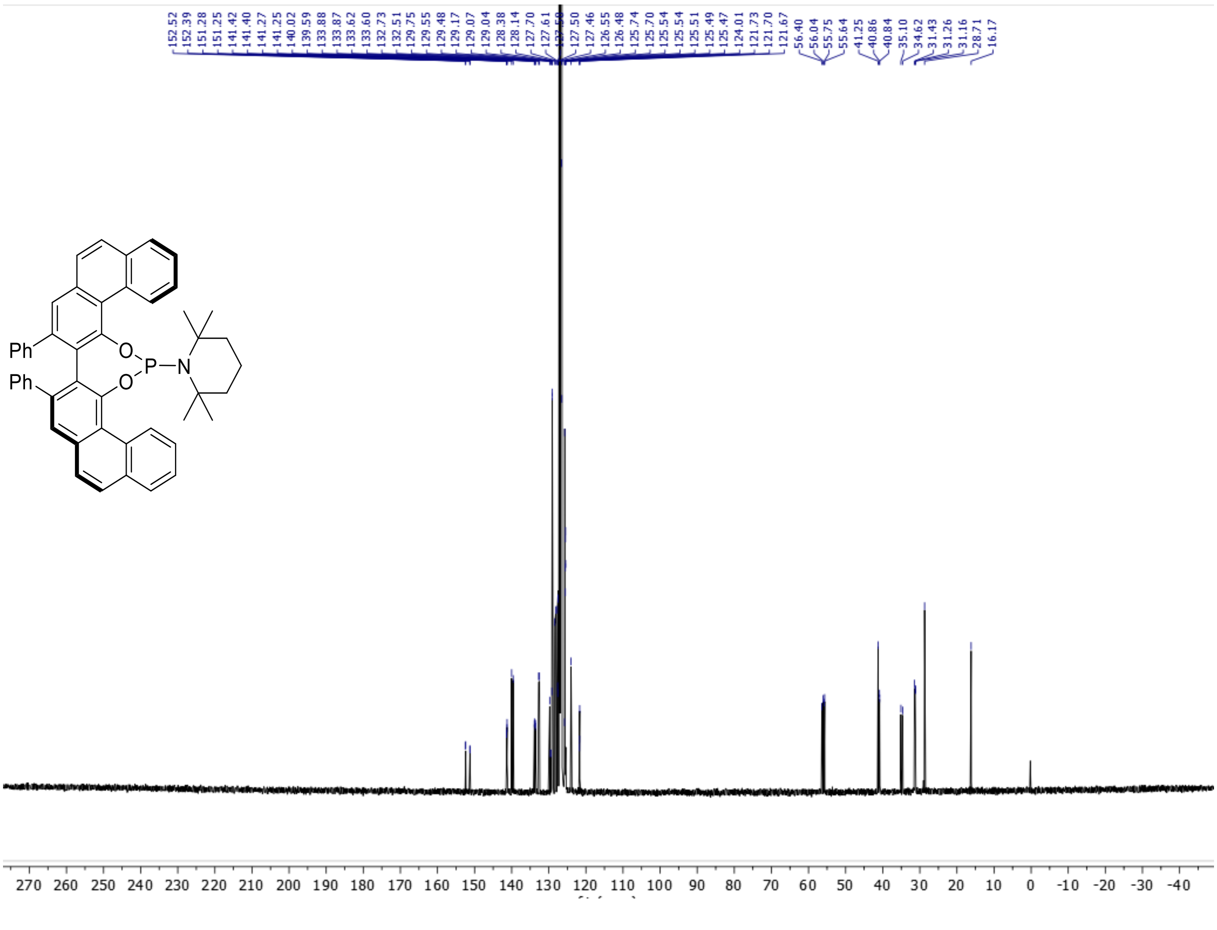

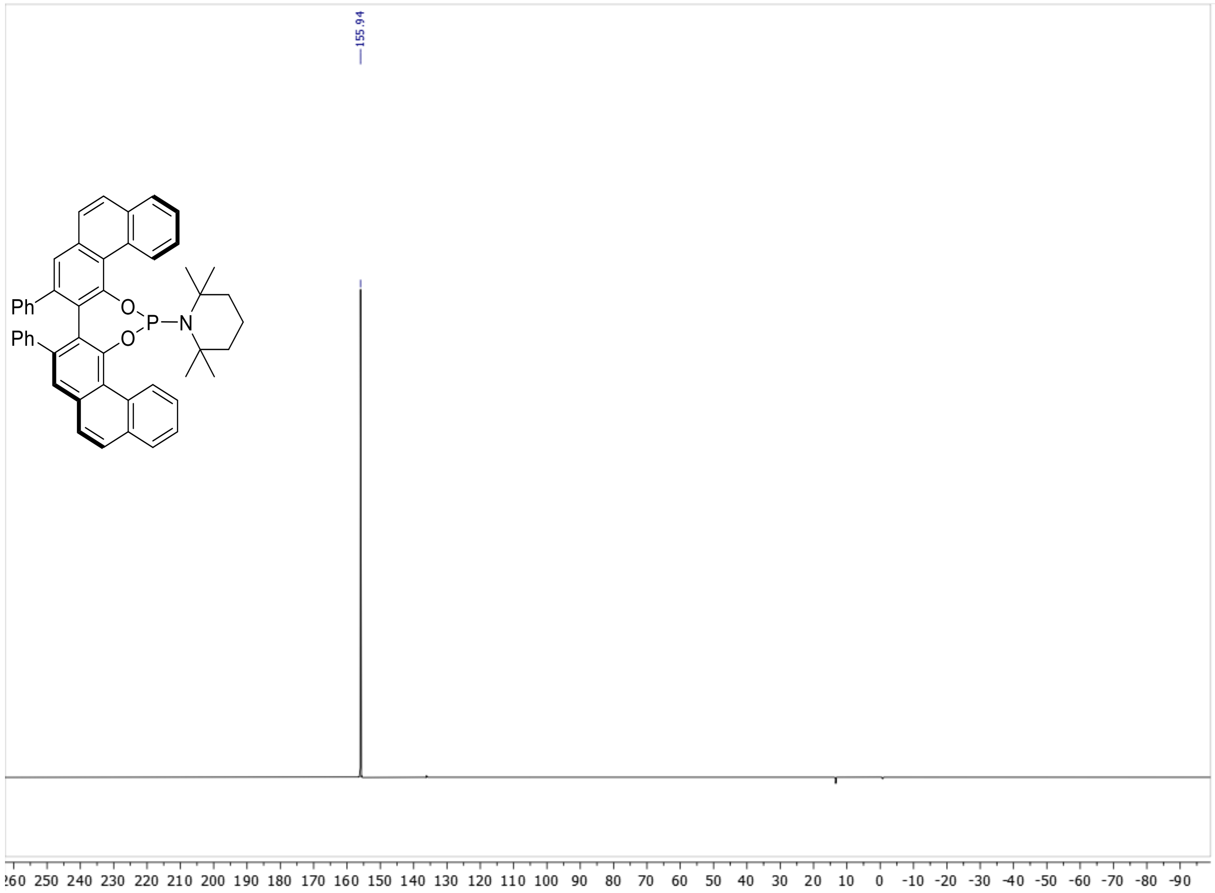

155.94
Ph
Ph
O
O
P
N
260 250 240 230 220 210 200 190 180 170 160 150 140 130 120 110 100 90 80 70 60 50 40 30 20 10 0 -10 -20 -30 -40 -50 -60 -70 -80 -90

SI-L37

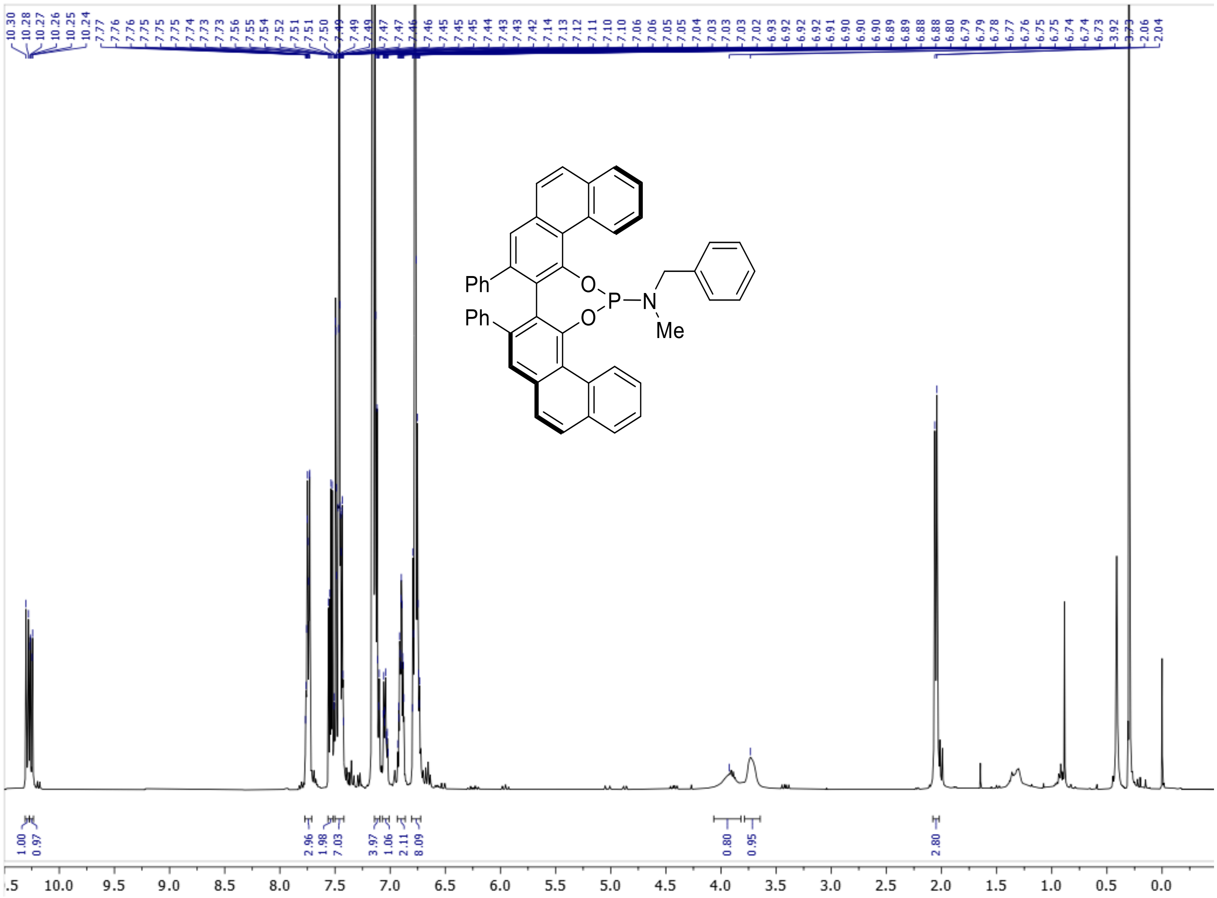

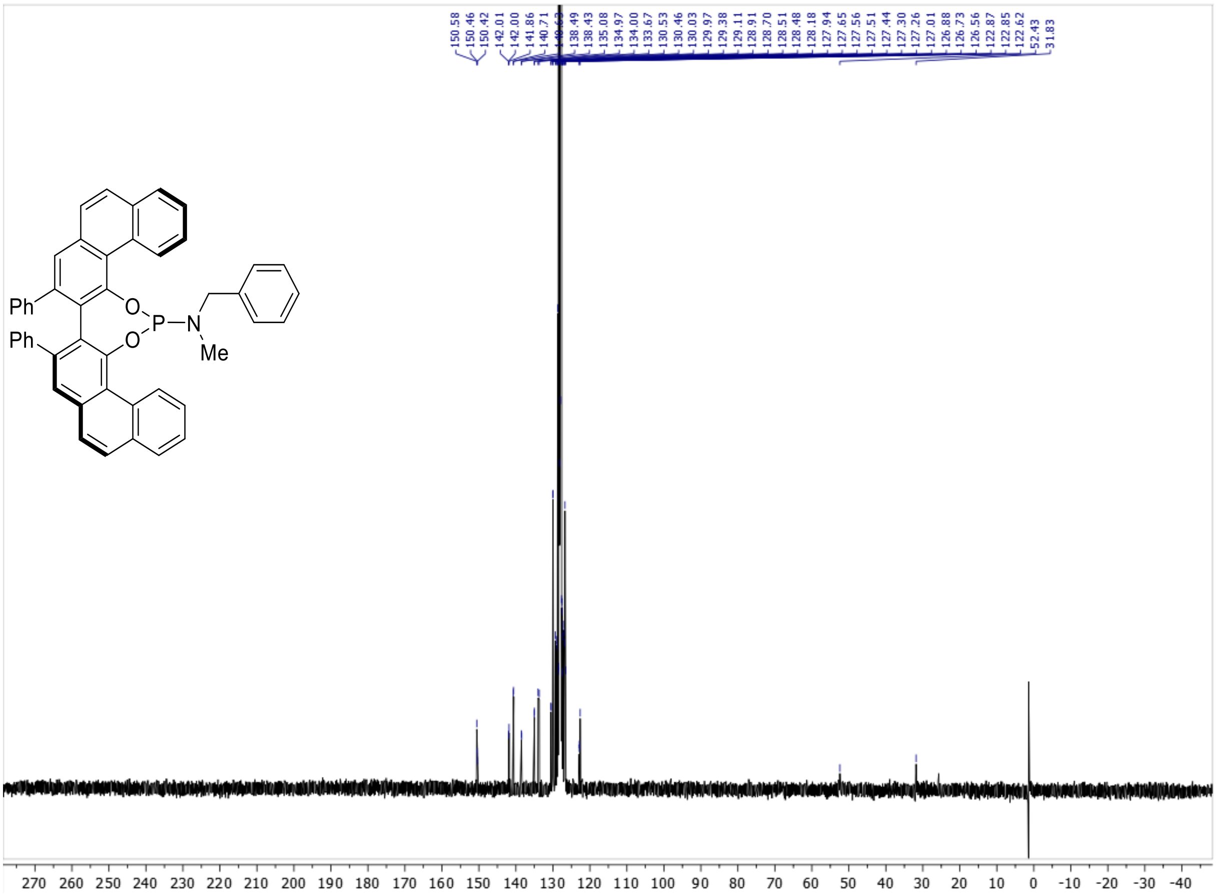

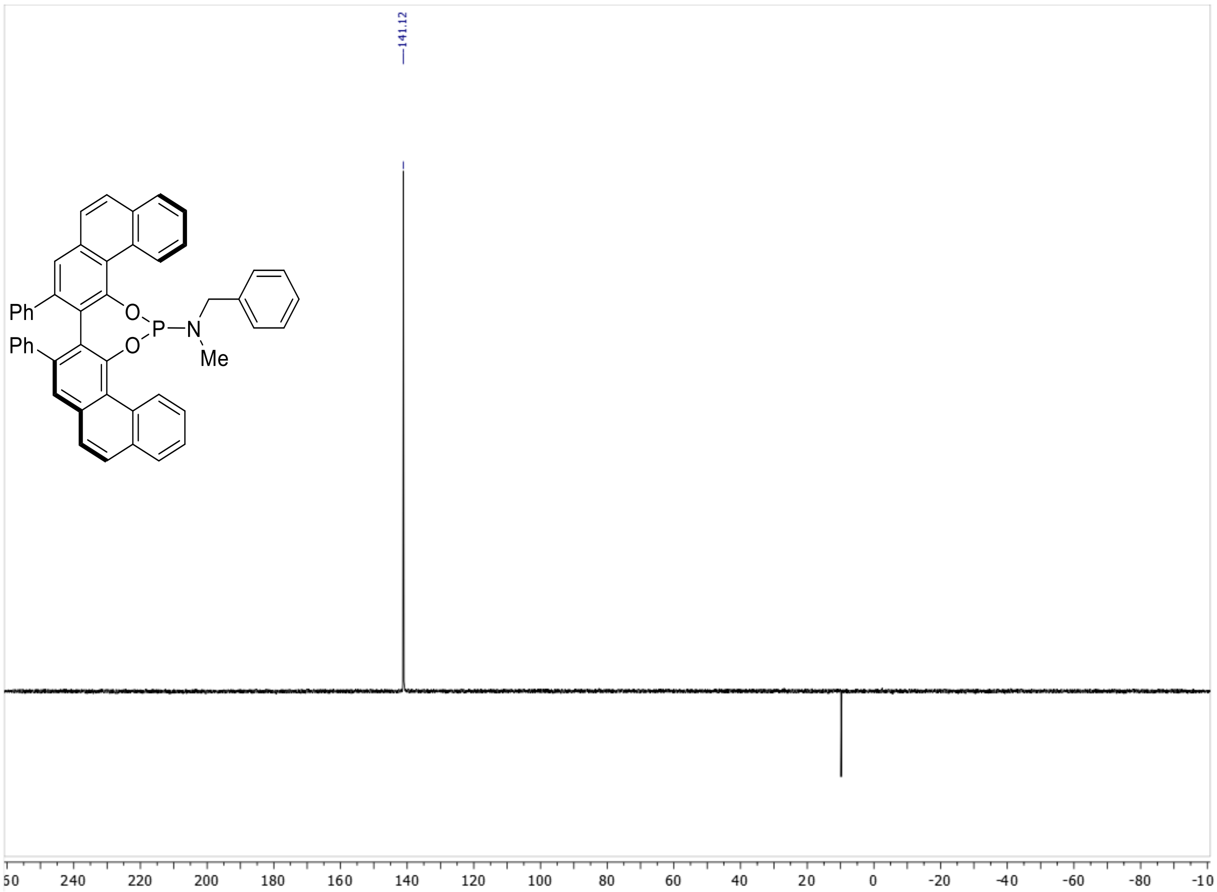
141.12
Ph
Ph
O
P
N
O
Me
60
240
220
200
180
160
140
120
100
80
60
40
20
0
-20
-40
-60
-80
-10

SI-L38

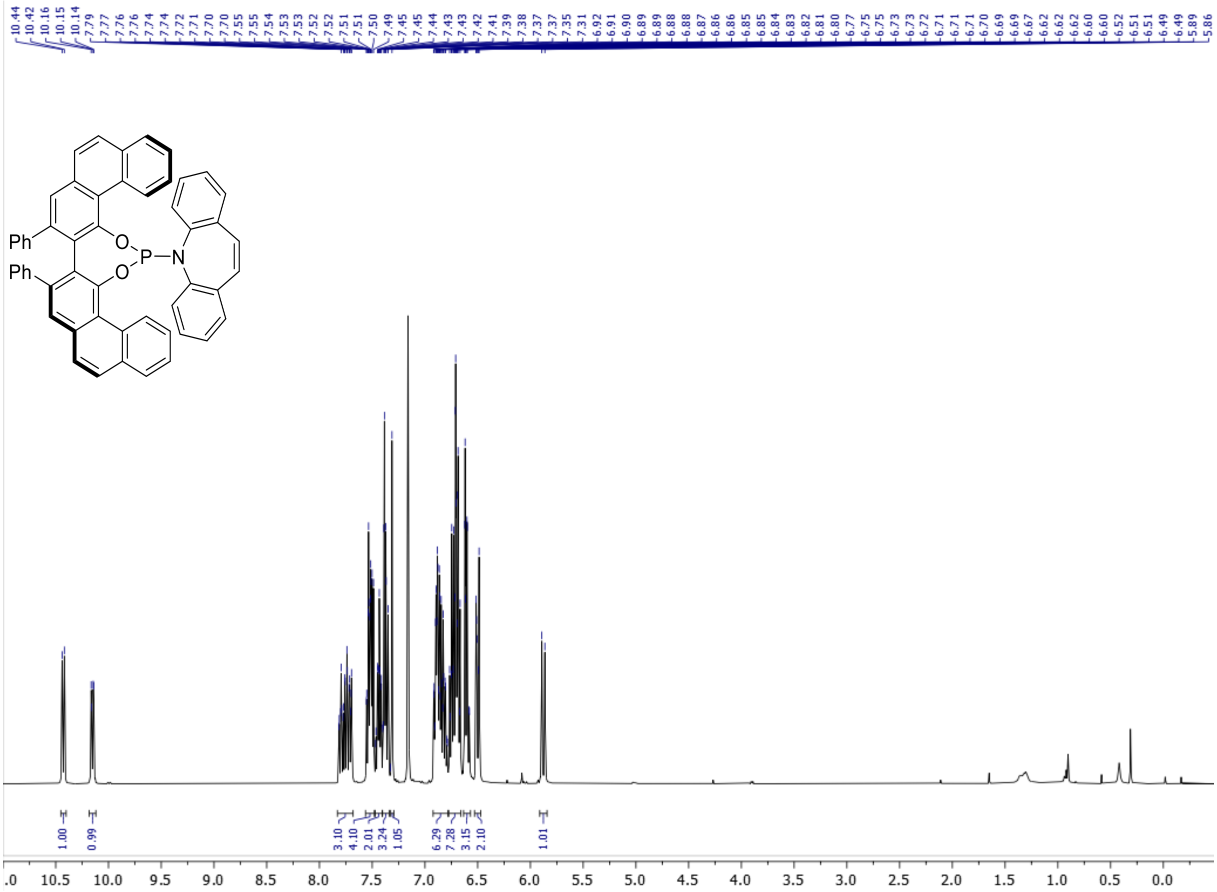
10.44 10.42 10.16 10.15 10.14 7.79 7.77 7.76 7.76 7.74 7.74 7.72 7.71 7.70 7.55 7.55 7.54 7.53 7.53 7.52 7.52 7.51 7.51 7.50 7.49 7.45 7.45 7.44 7.43 7.43 7.43 7.42 7.41 7.39 7.38 7.37 7.37 7.35 7.31 6.92 6.91 6.90 6.89 6.89 6.88 6.88 6.87 6.86 6.86 6.85 6.85 6.84 6.83 6.82 6.81 6.80 6.77 6.75 6.75 6.73 6.73 6.72 6.71 6.71 6.70 6.69 6.69 6.67 6.62 6.62 6.62 6.60 6.60 6.52 6.51 6.51 6.49 6.49 5.89 5.86
Ph
O
P
N
O
Ph
1.00 0.99 3.10 4.10 2.01 3.24 1.05 6.29 7.28 3.15 2.10 1.01
.0 10.5 10.0 9.5 9.0 8.5 8.0 7.5 7.0 6.5 6.0 5.5 5.0 4.5 4.0 3.5 3.0 2.5 2.0 1.5 1.0 0.5 0.0

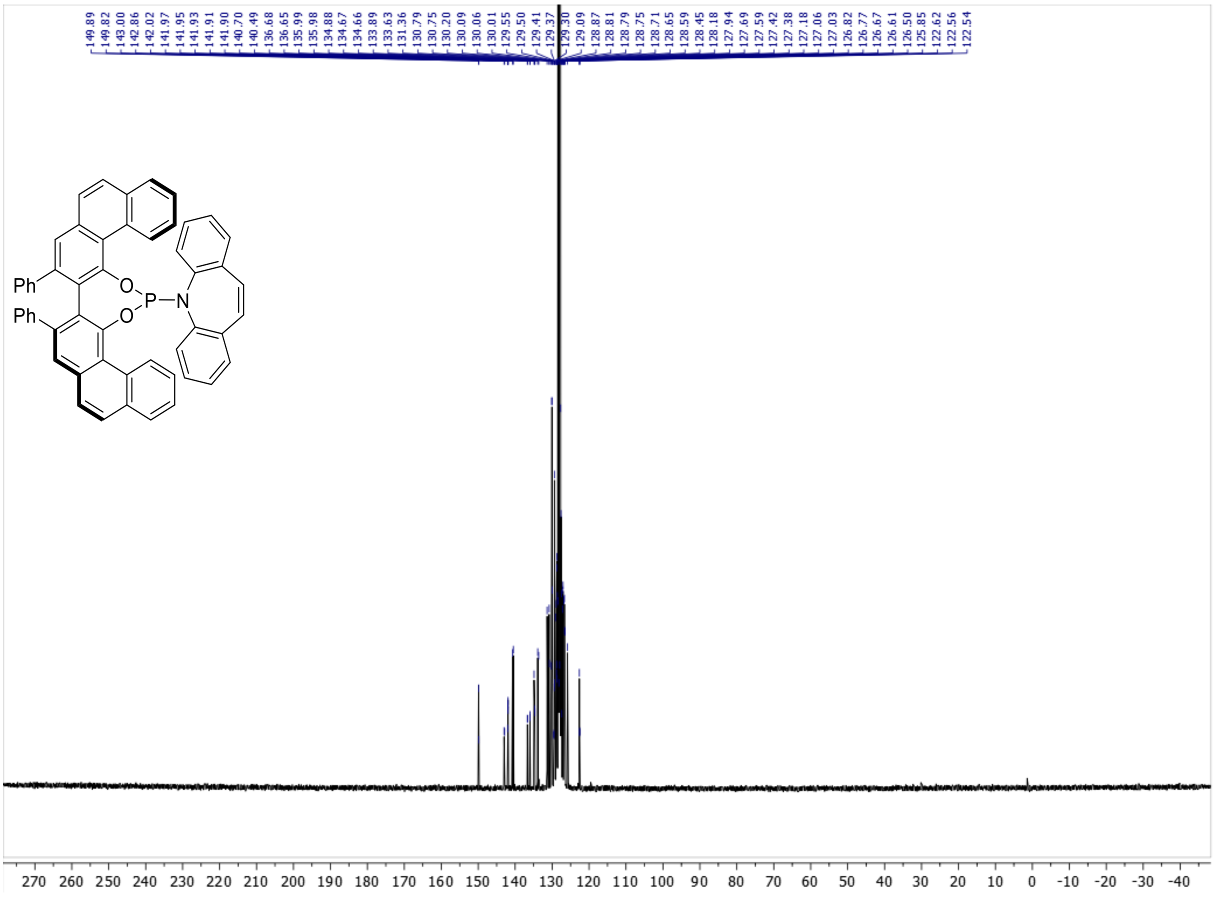
149.89 149.82 143.00 142.86 142.02 141.97 141.95 141.93 141.91 141.90 140.70 140.49 136.68 136.65 135.99 135.98 134.88 134.67 134.66 133.89 133.63 131.36 130.79 130.75 130.20 130.09 130.06 130.01 129.55 129.50 129.41 129.37 129.30 129.09 128.87 128.81 128.79 128.75 128.71 128.65 128.59 128.45 128.18 127.94 127.69 127.59 127.42 127.38 127.18 127.06 127.03 126.82 126.77 126.67 126.61 126.50 125.85 122.62 122.56 122.54
Ph
O
P
N
O
Ph
270 260 250 240 230 220 210 200 190 180 170 160 150 140 130 120 110 100 90 80 70 60 50 40 30 20 10 0 -10 -20 -30 -40

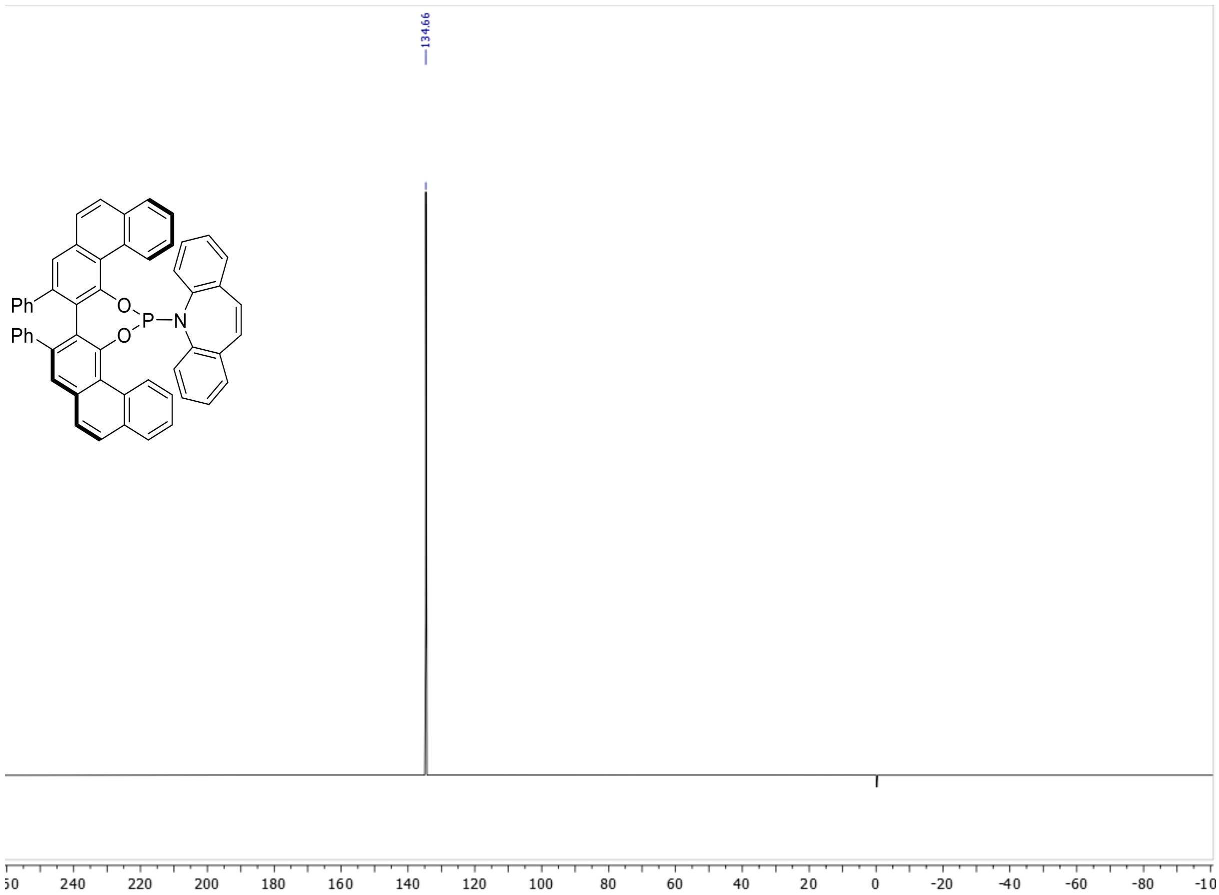
134.66
Ph
Ph
O
O
P
N
50 240 220 200 180 160 140 120 100 80 60 40 20 0 -20 -40 -60 -80 -10

***anti*-6-methyl-1-phenyl-4-((triisopropylsilyl)oxy)hept-6-en-3-ol (9a).**

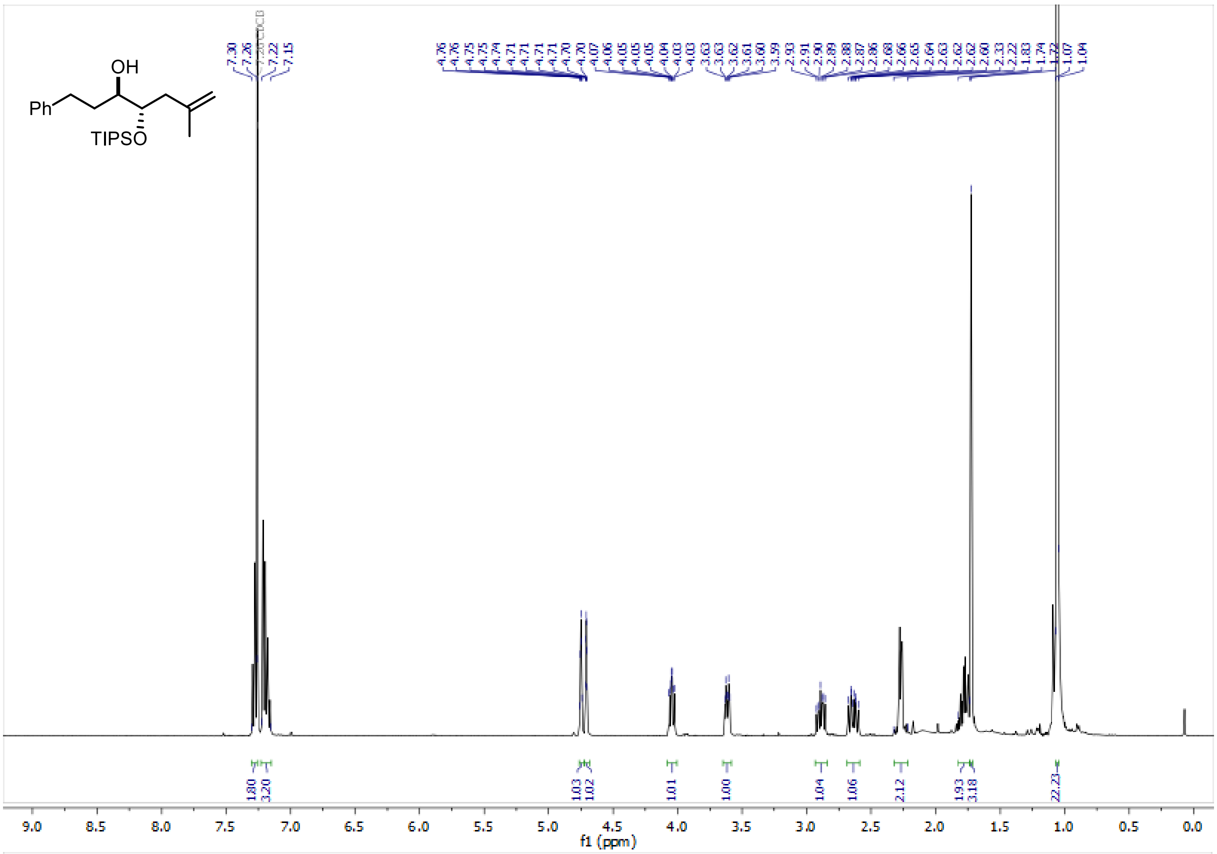

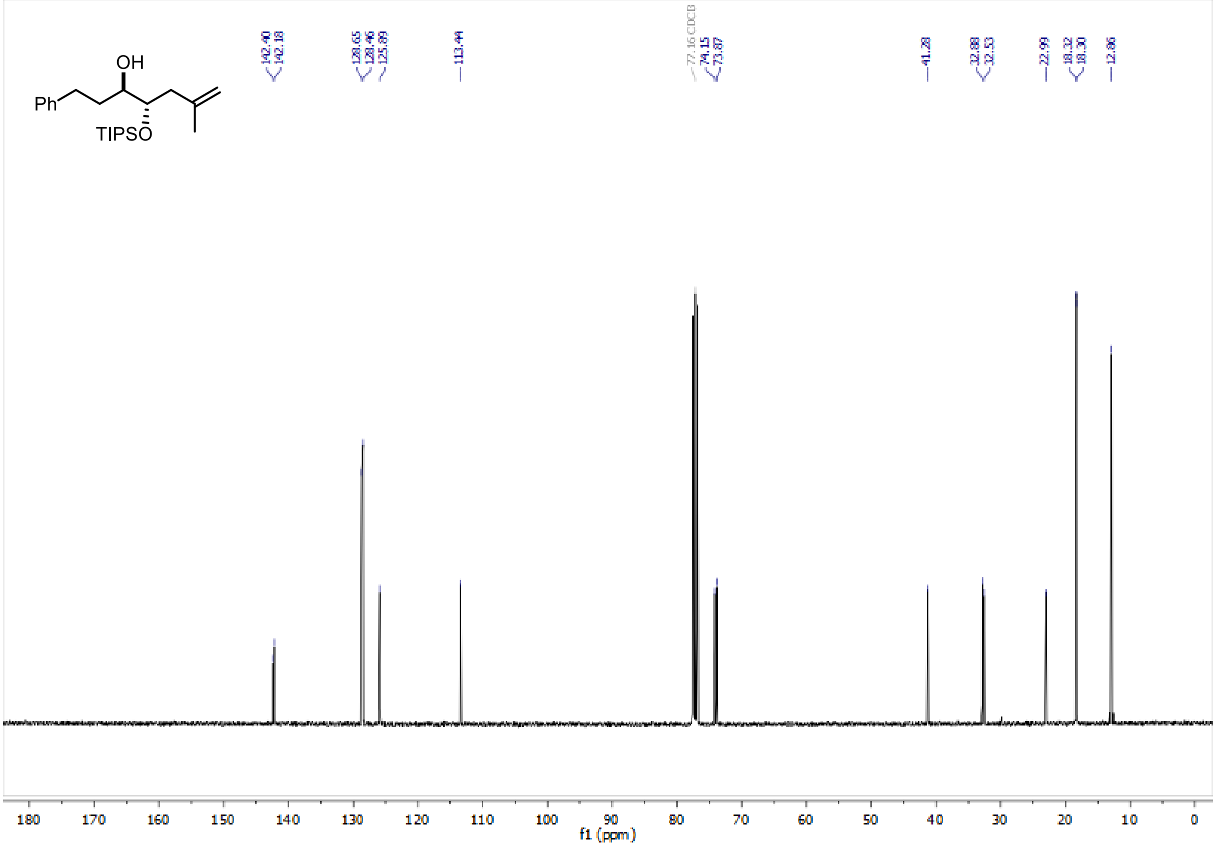

***anti*-4-((tert-butyldimethylsilyl)oxy)-6-methyl-1-phenylhept-6-en-3-ol (9b).**

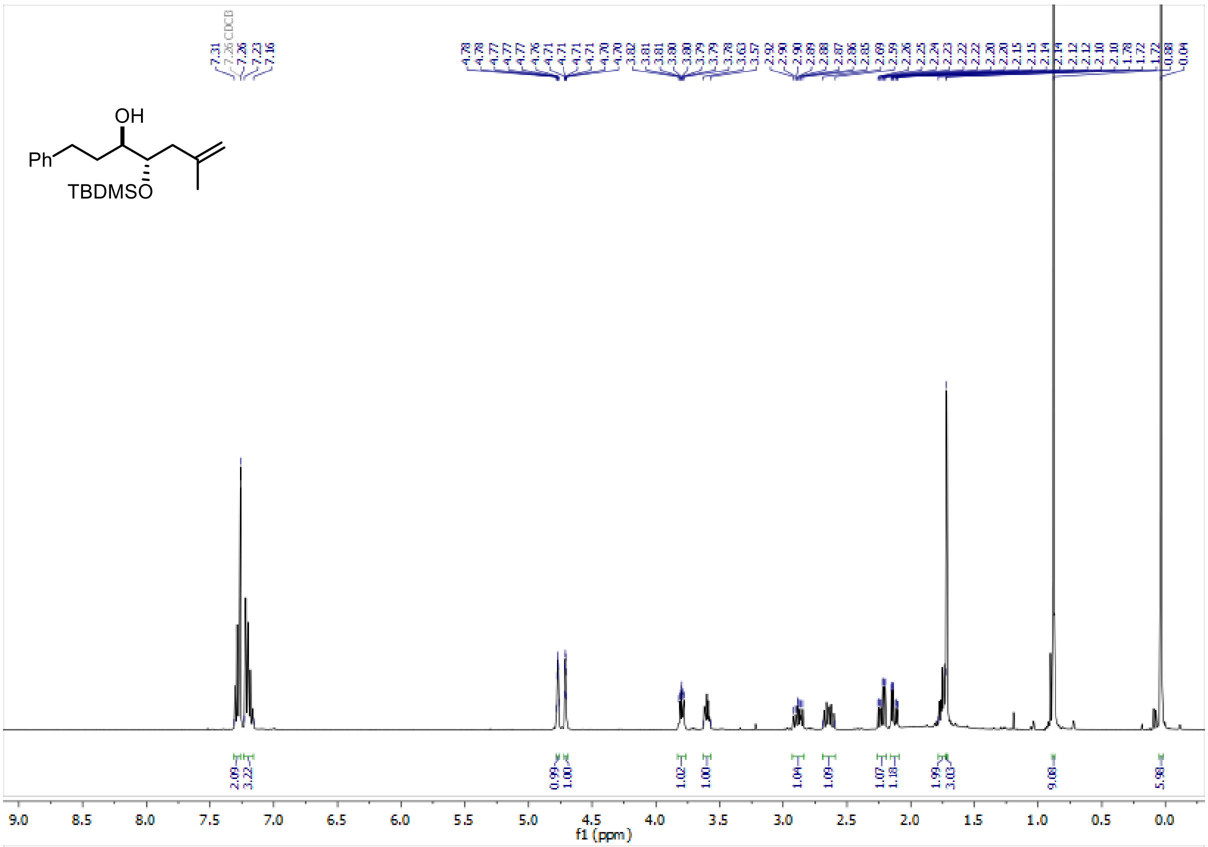

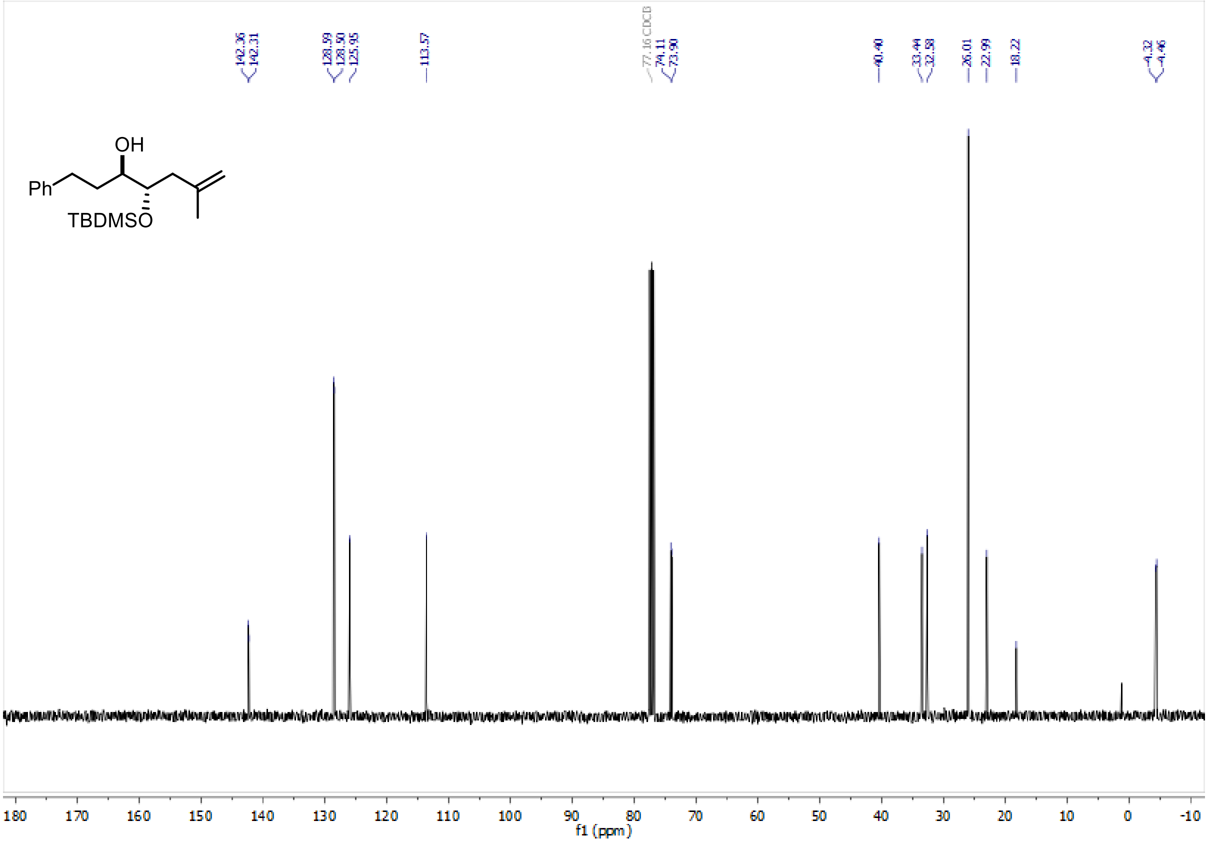

***anti*-6-methyl-1-phenyl-4-((triethylsilyl)oxy)hept-6-en-3-ol (9c).**

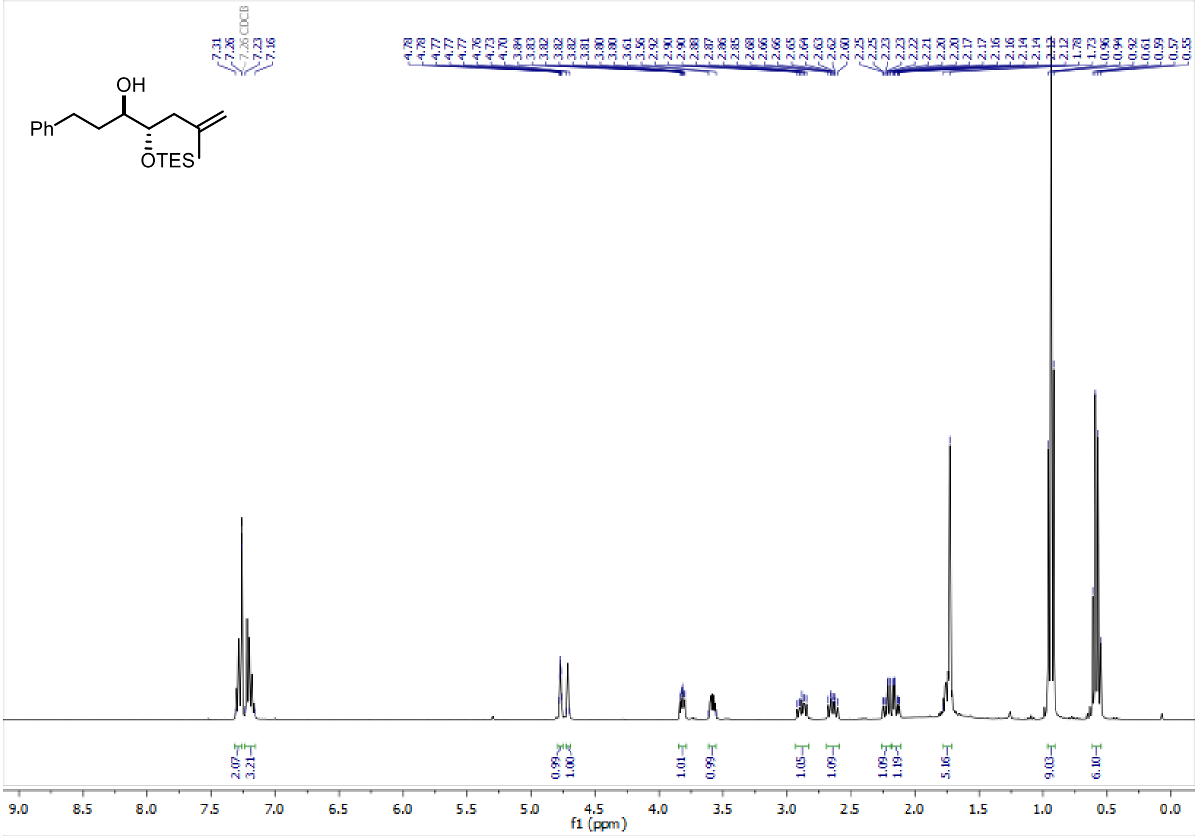

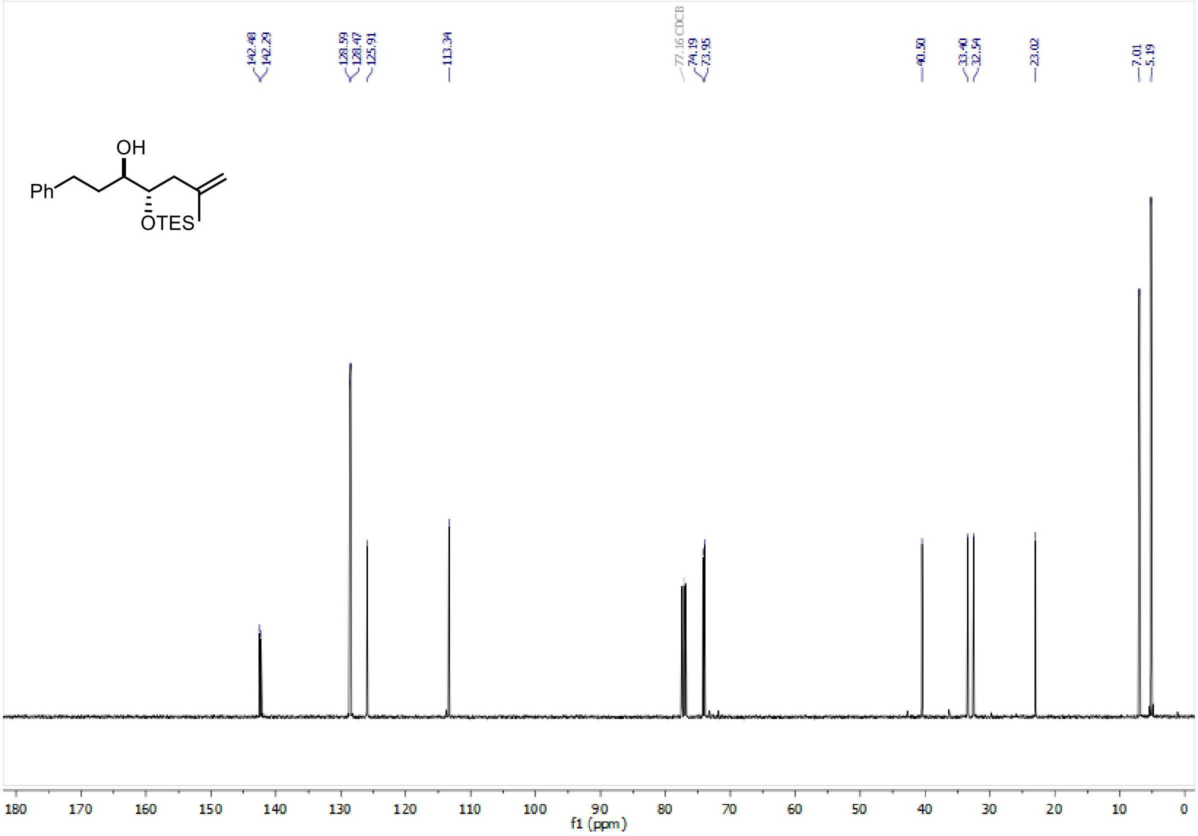

***anti*-2-methyl-4-((triisopropylsilyl)oxy)non-1-en-5-ol (9d).**

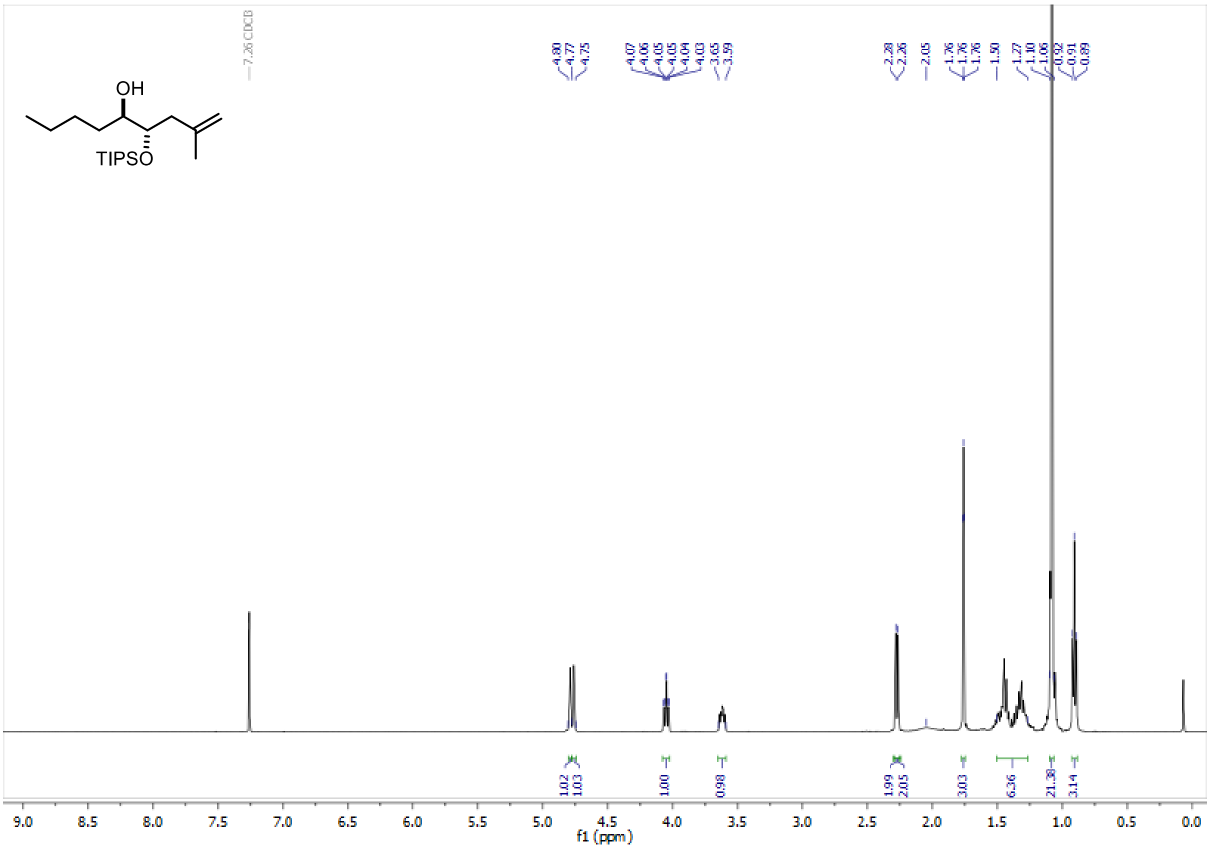

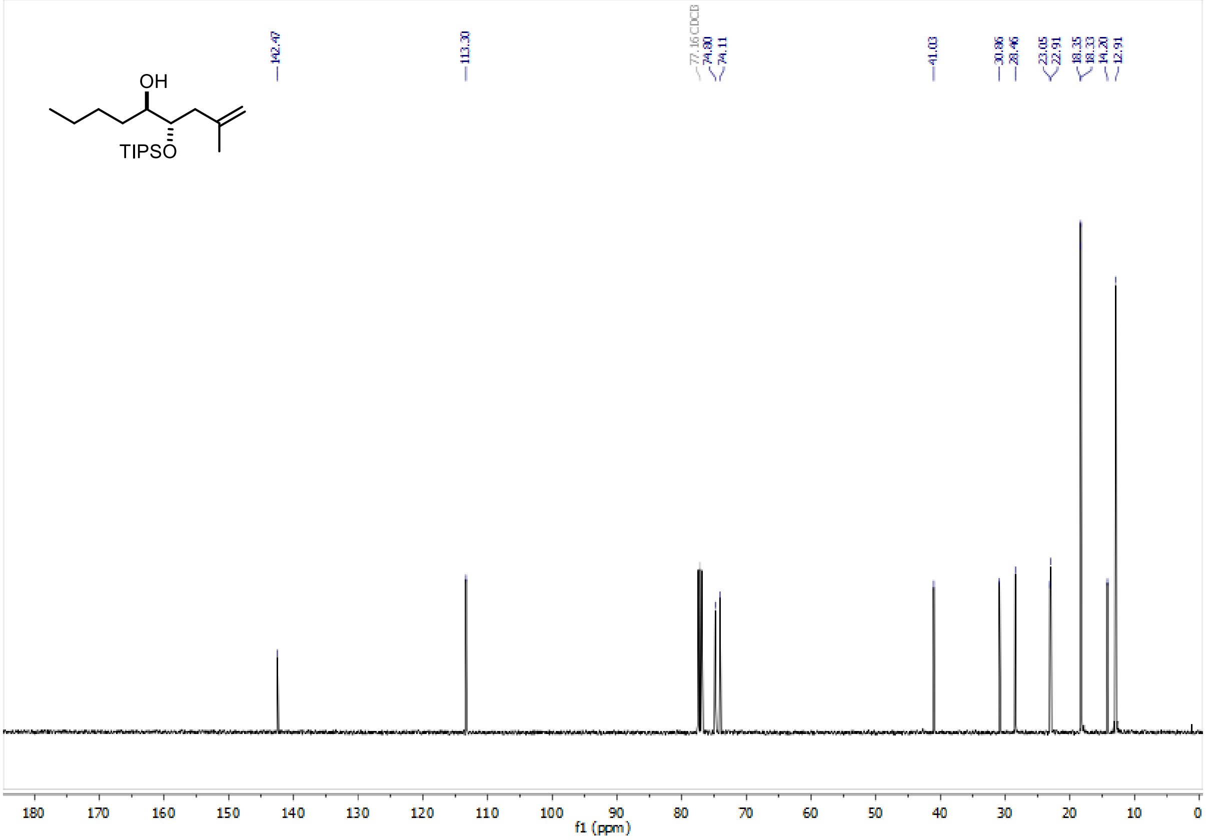

***anti*-2,6-dimethyl-4-((triisopropylsilyl)oxy)hept-6-en-3-ol (9e).**

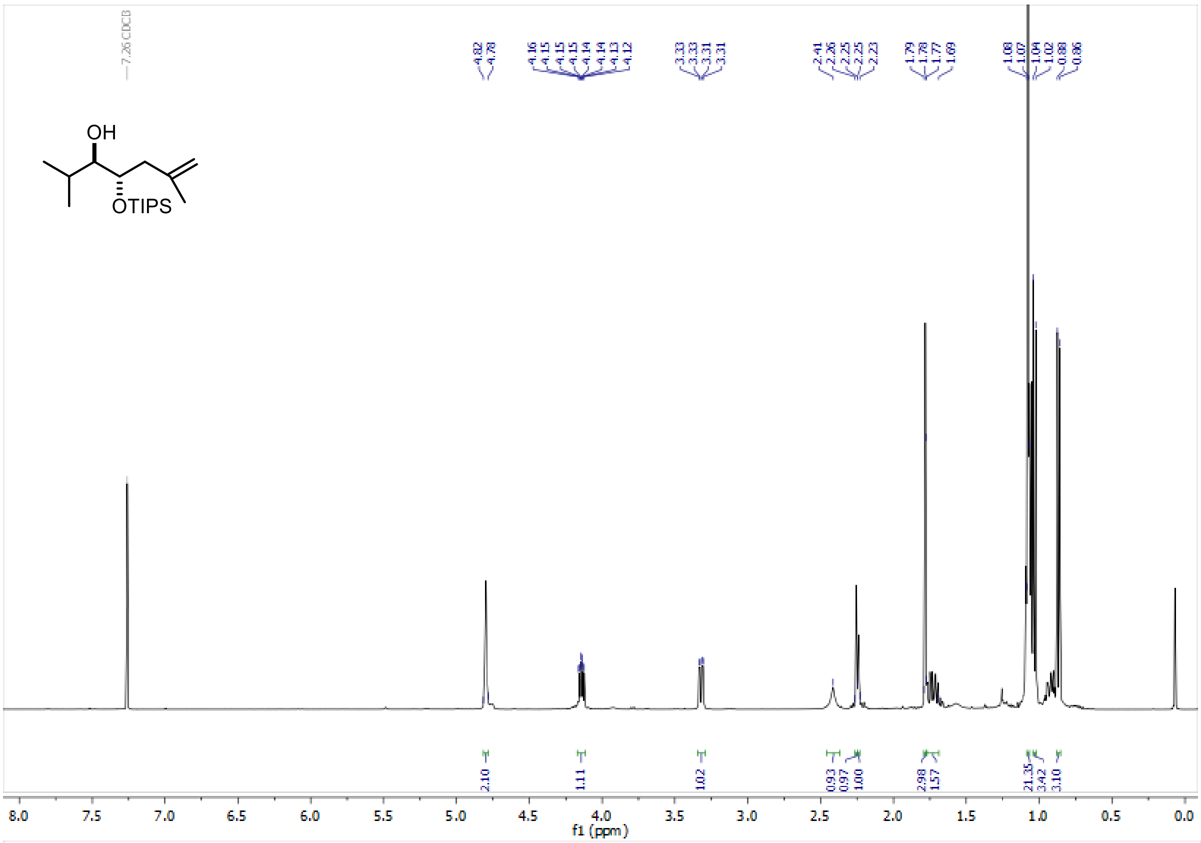

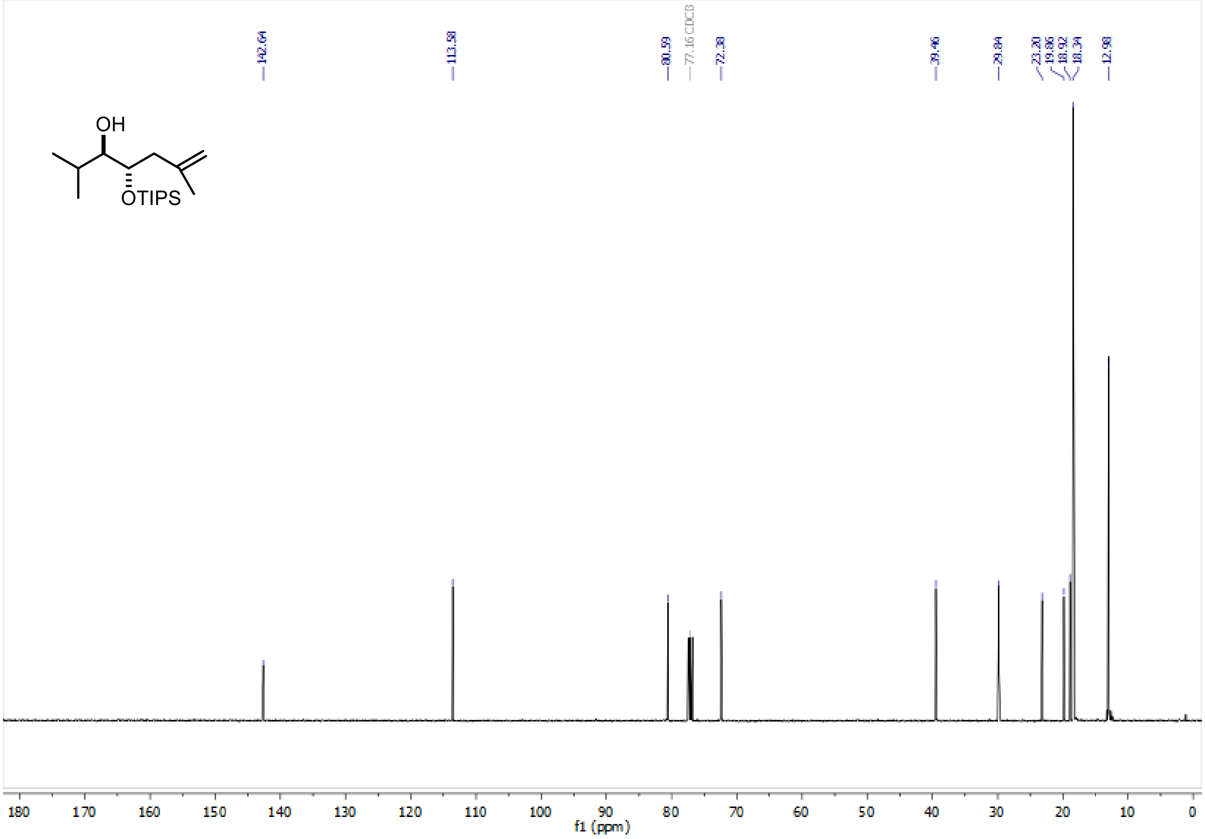

***anti*-1-cyclopropyl-4-methyl-2-((triisopropylsilyl)oxy)pent-4-en-1-ol (9f).**

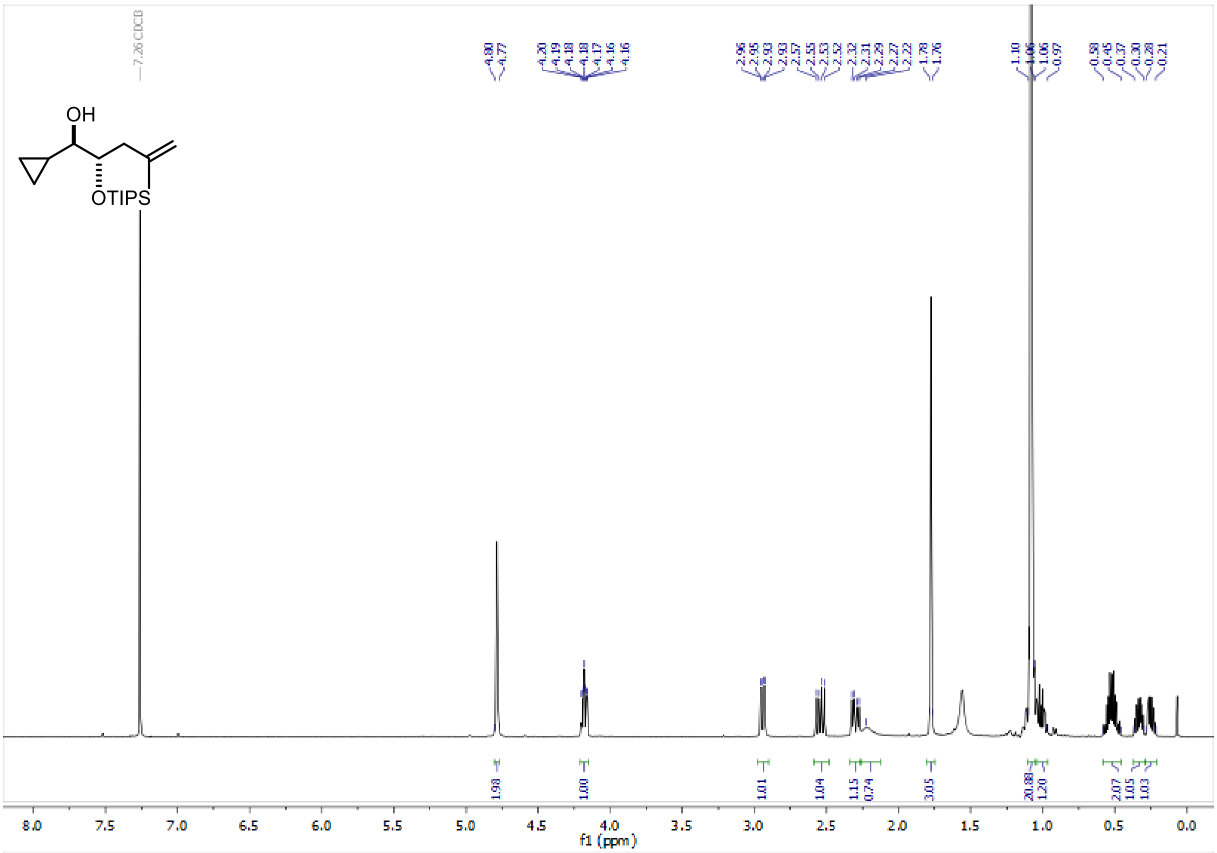

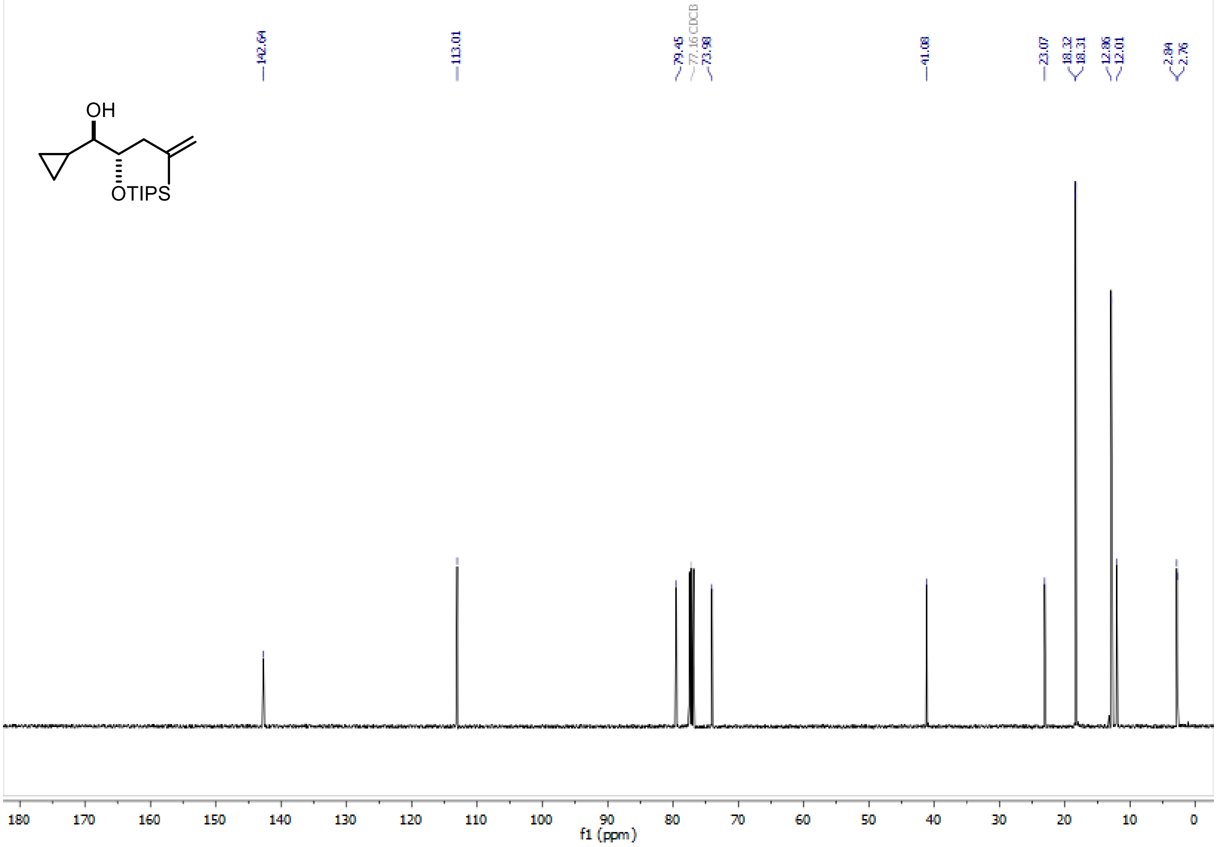

***anti*-2,2,6-trimethyl-4-((triisopropylsilyl)oxy)hept-6-en-3-ol (9g).**

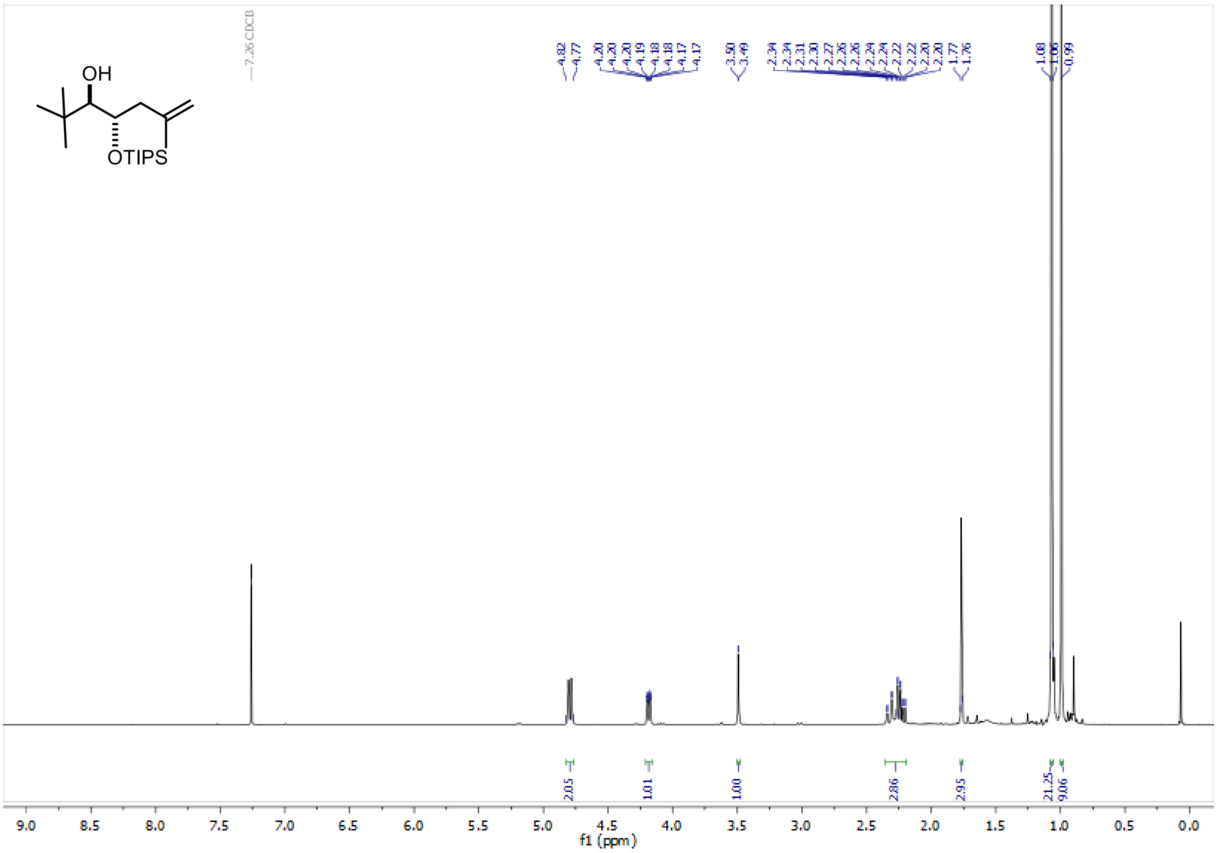

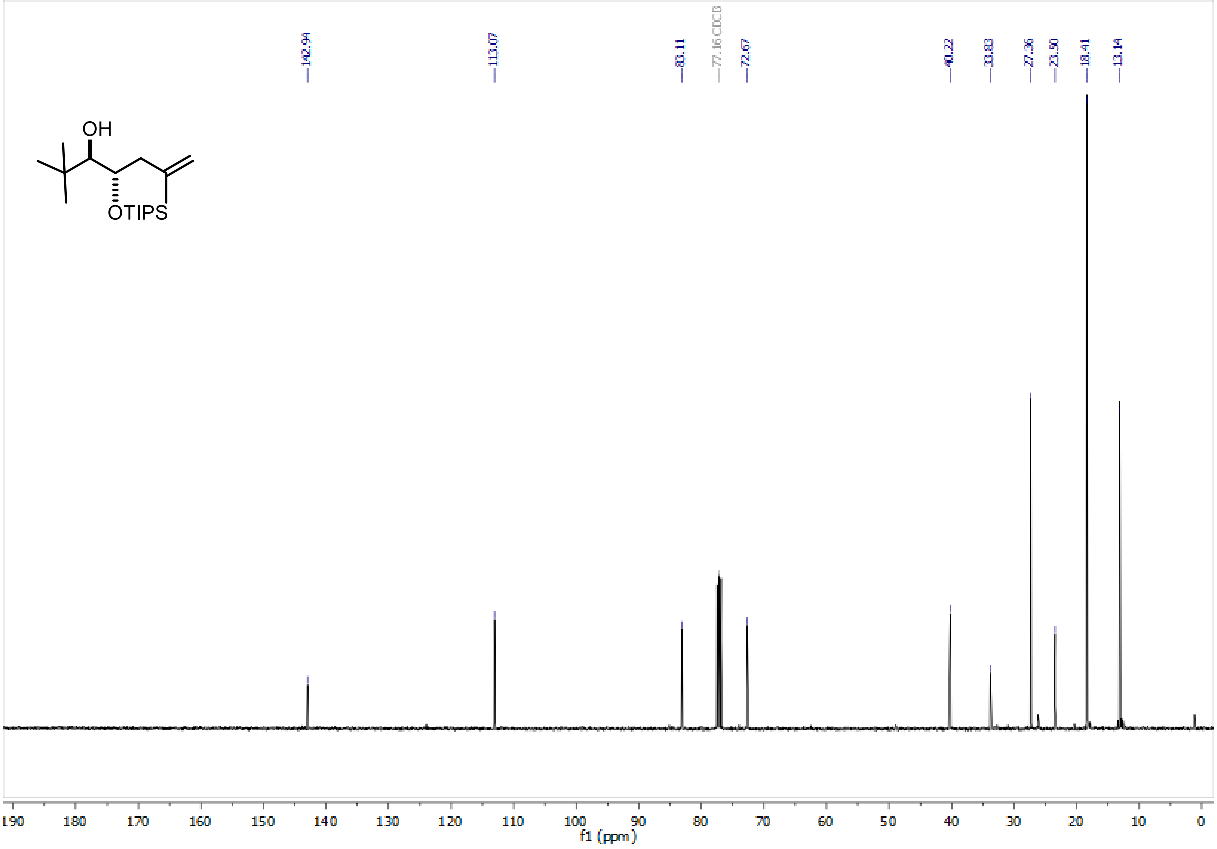

***anti*-2,7,11-trimethyl-4-((triisopropylsilyl)oxy)dodeca-1,10-dien-5-ol (9h).**

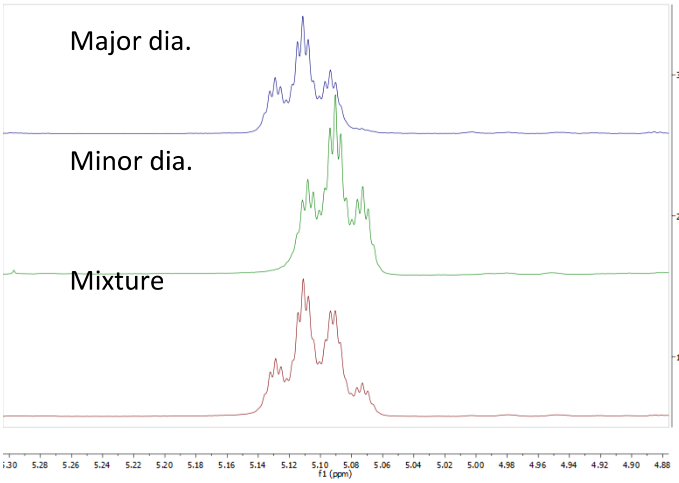

Figure 1: ^{1}H NMR

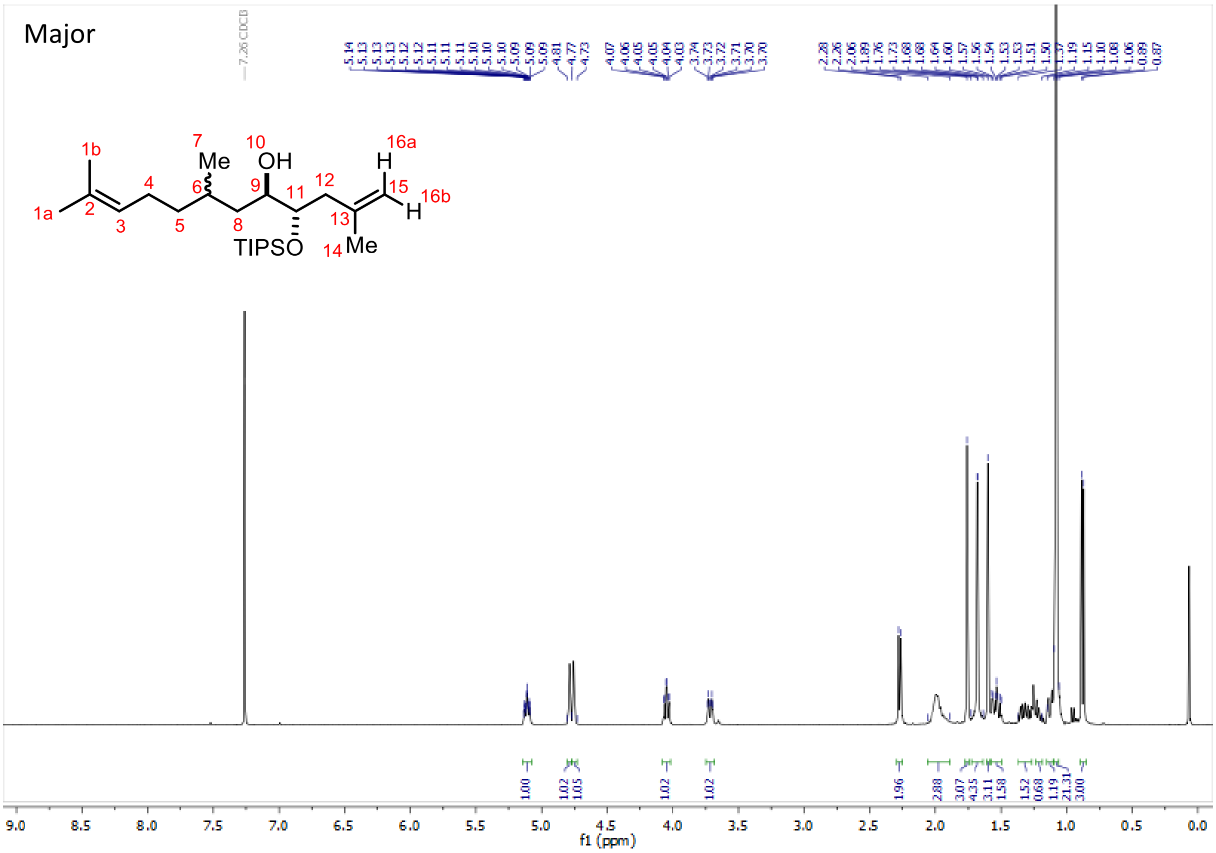

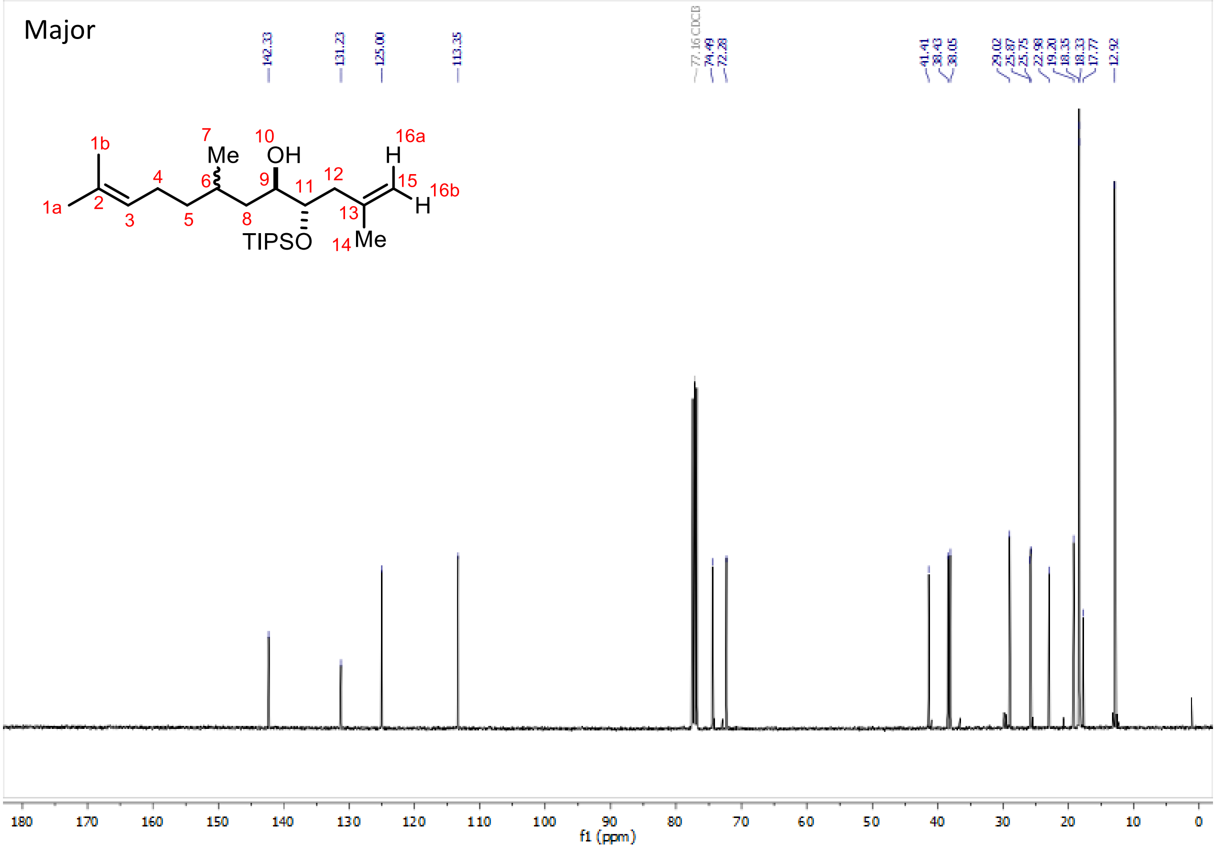
Major
142.33
131.23
125.00
113.35
77.16 CDCl3
74.49
72.28
41.41
38.43
38.05
29.02
25.87
25.75
22.98
19.20
18.35
18.33
17.77
12.92
1b
7
10
16a
Me
OH
H
1a
2
3
4
5
6
8
9
11
12
13
15
16b
TIPSO
14
Me
f1 (ppm)

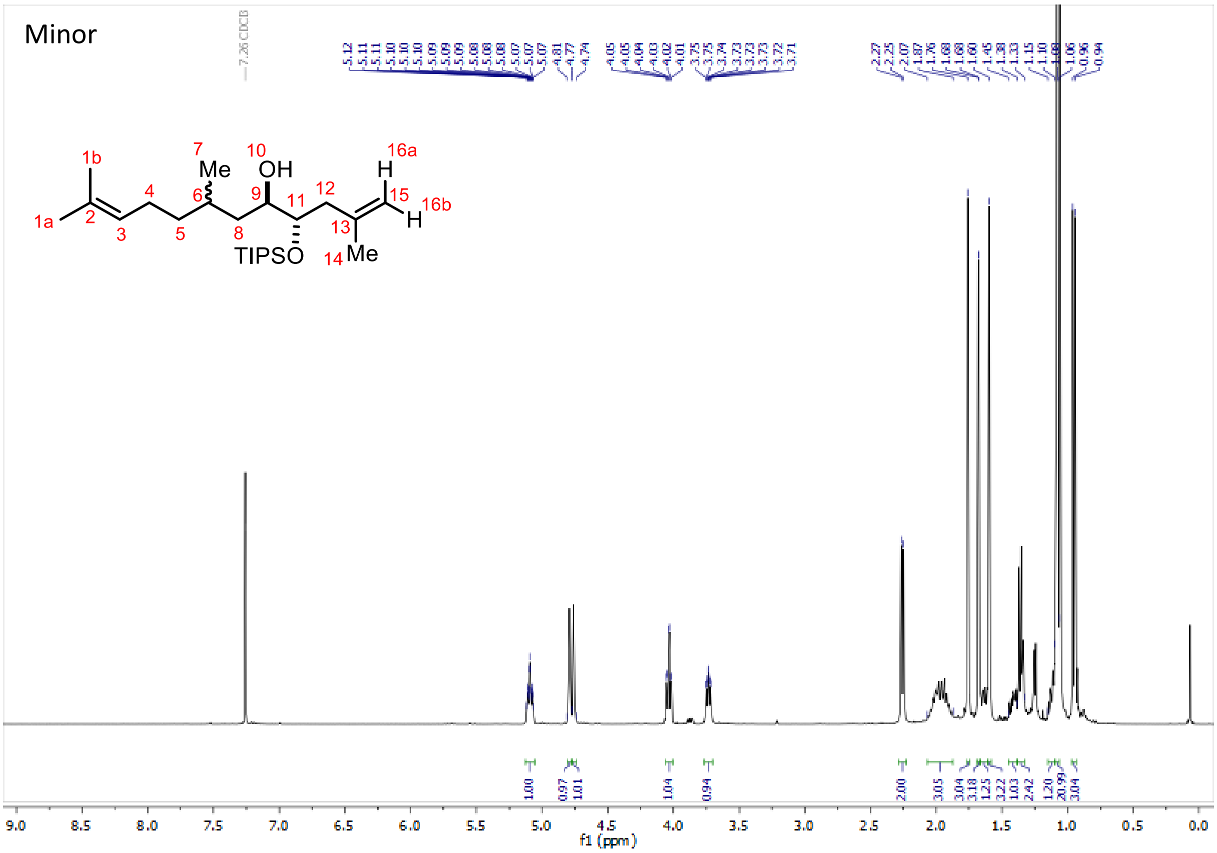
Minor
7.26 CDCl3
f1 (ppm)

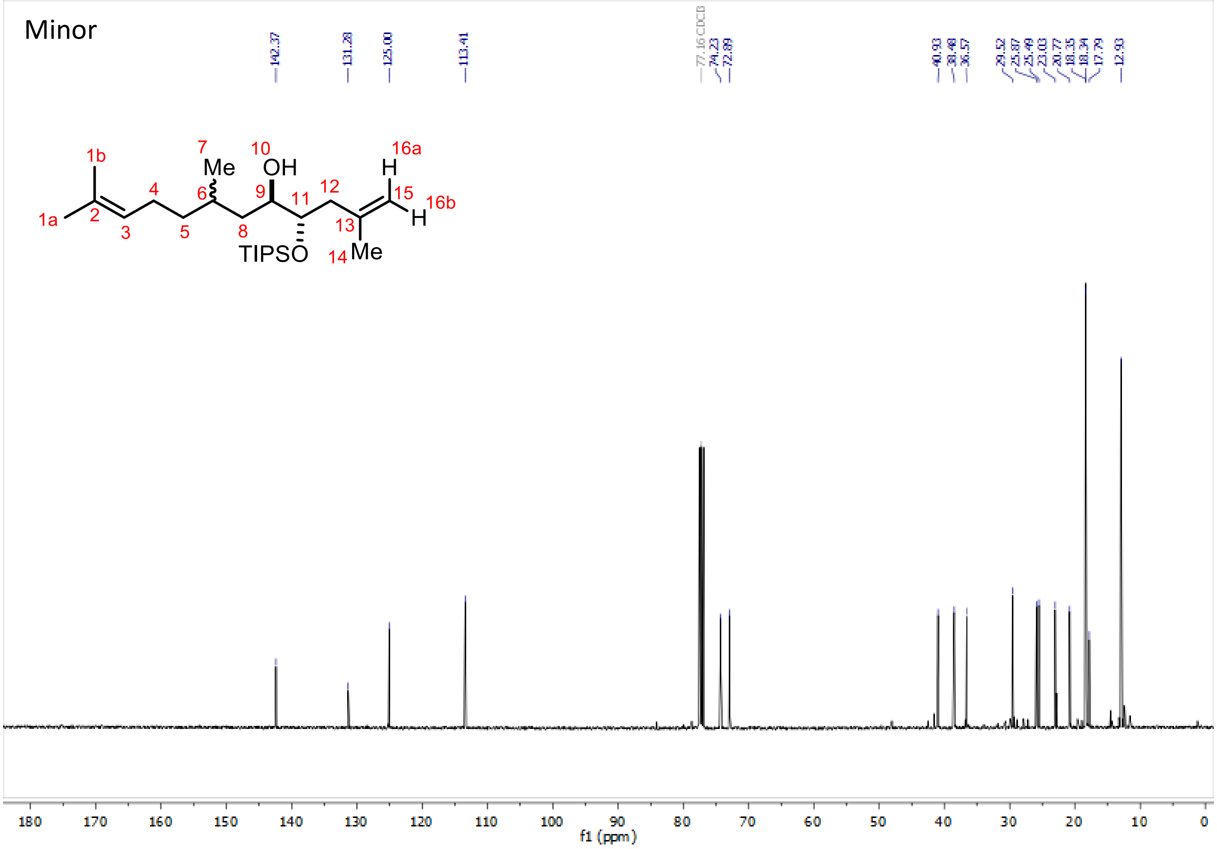
Minor
142.37
131.28
125.00
113.41
77.16 CDCl3
74.23
72.89
40.93
38.48
36.57
29.52
25.87
25.49
23.03
20.77
18.35
18.34
17.79
12.93
1b
1a
2
3
4
5
6
7
Me
8
9
10
OH
11
12
13
14
Me
15
16a
16b
H
H
TIPSO
f1 (ppm)

***anti*-1-phenyl-4-((triisopropylsilyl)oxy)hept-6-en-3-ol (9i).**

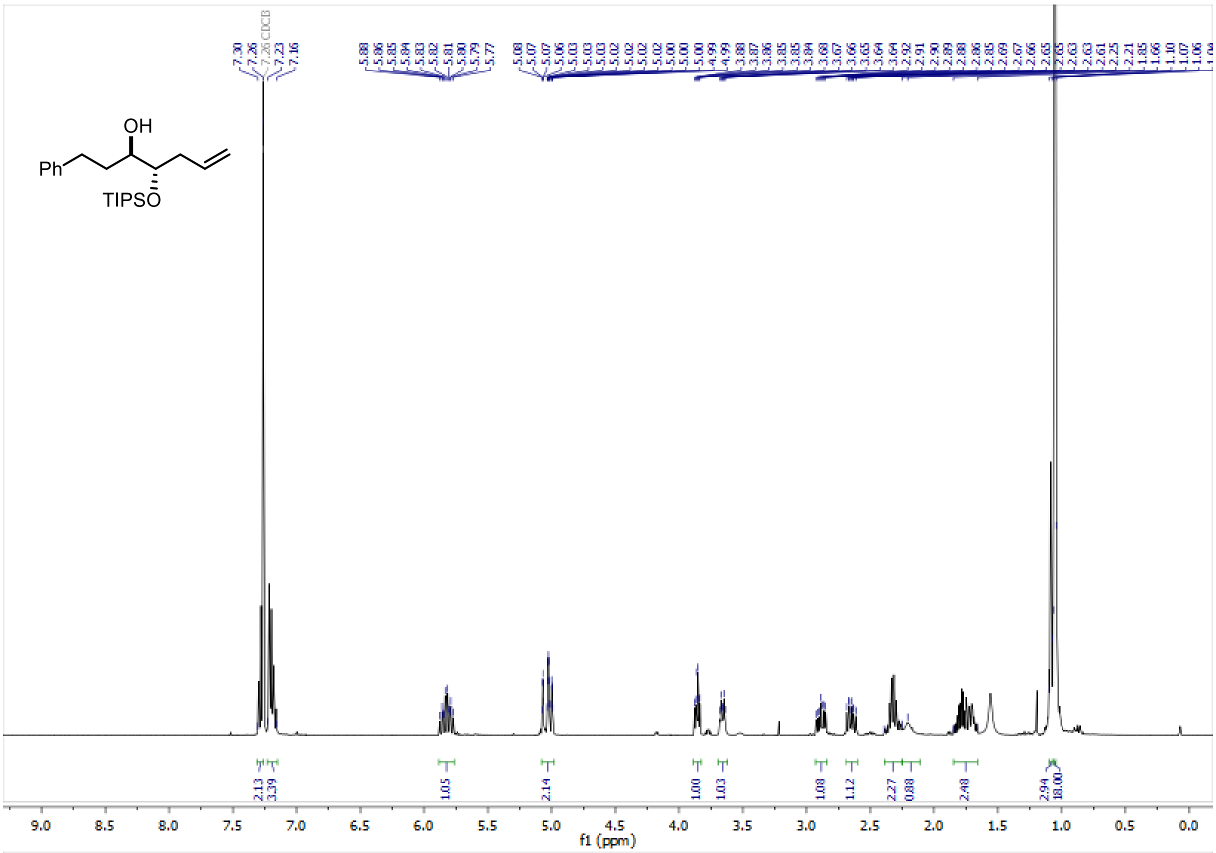

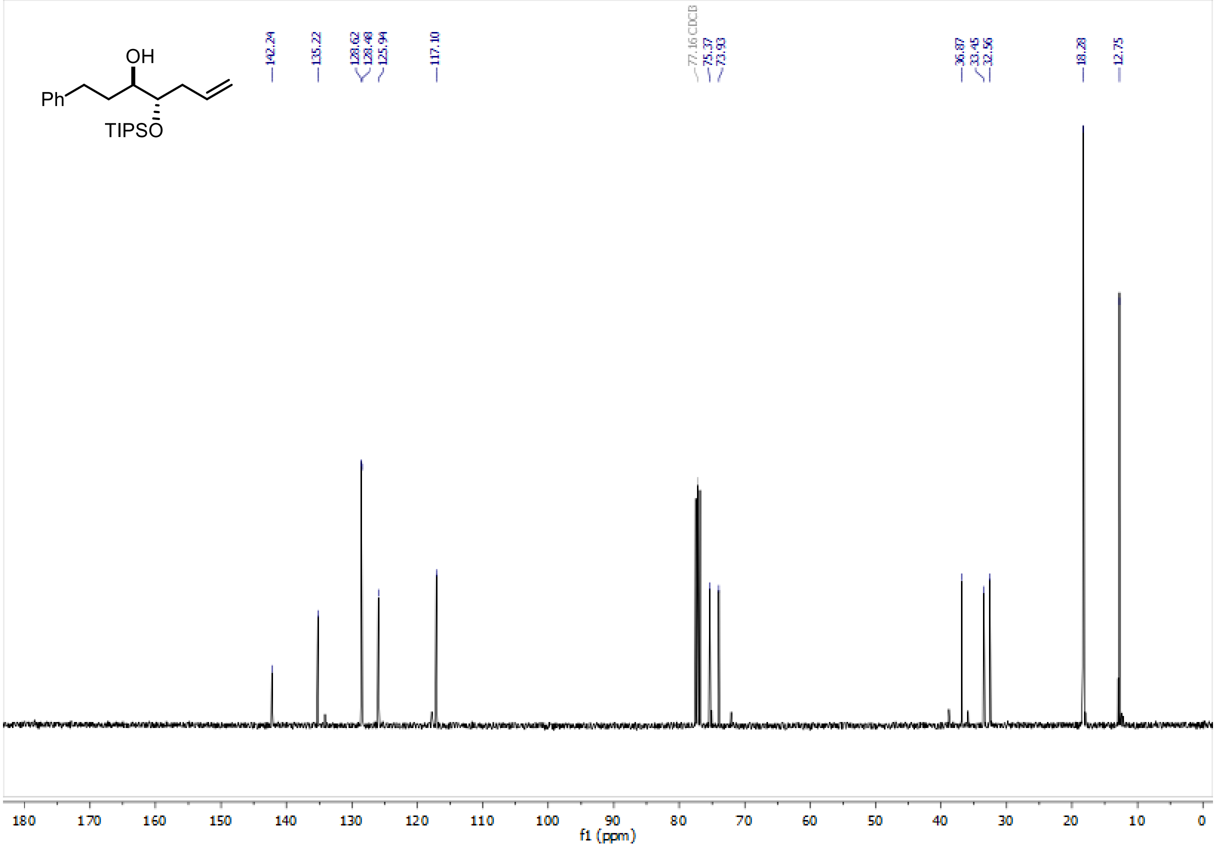

9k

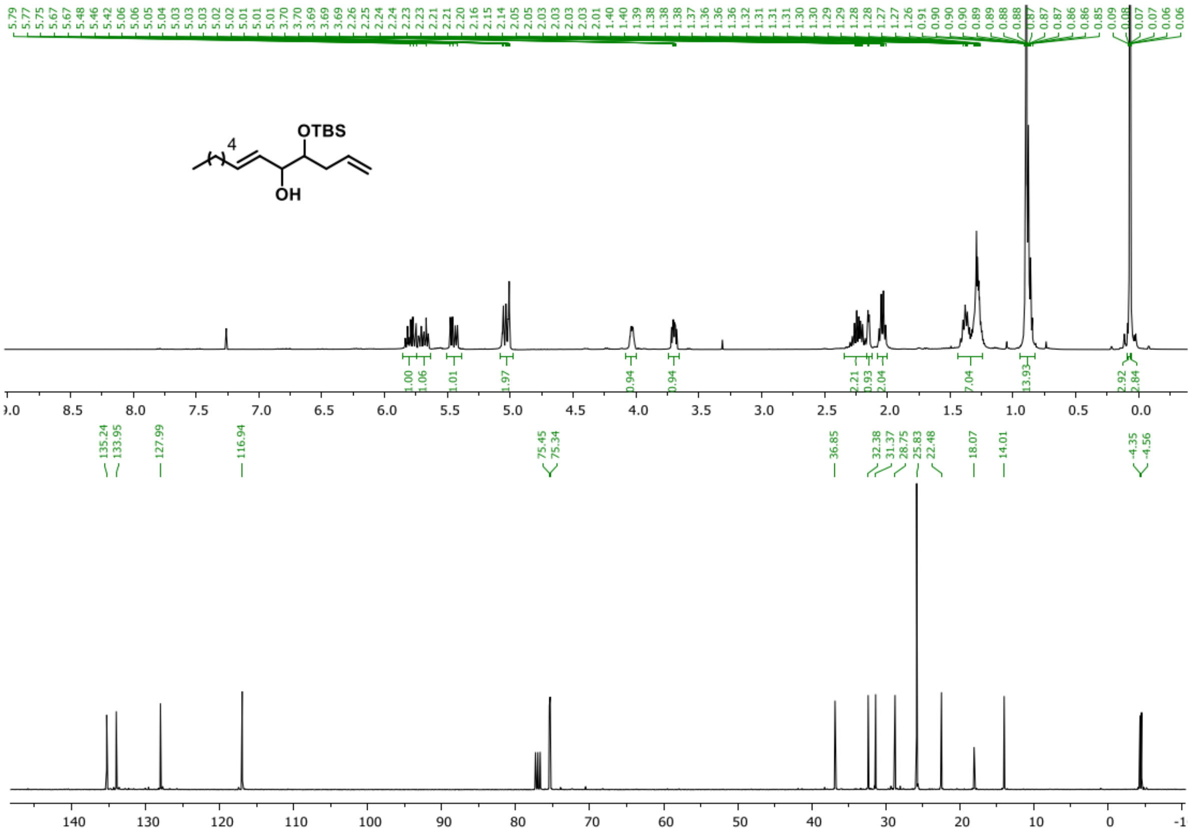
OTBS
4
OH
135.24
133.95
127.99
116.94
75.45
75.34
36.85
32.38
31.37
28.75
25.83
22.48
18.07
14.01
-4.35
-4.56

iso-**9k**

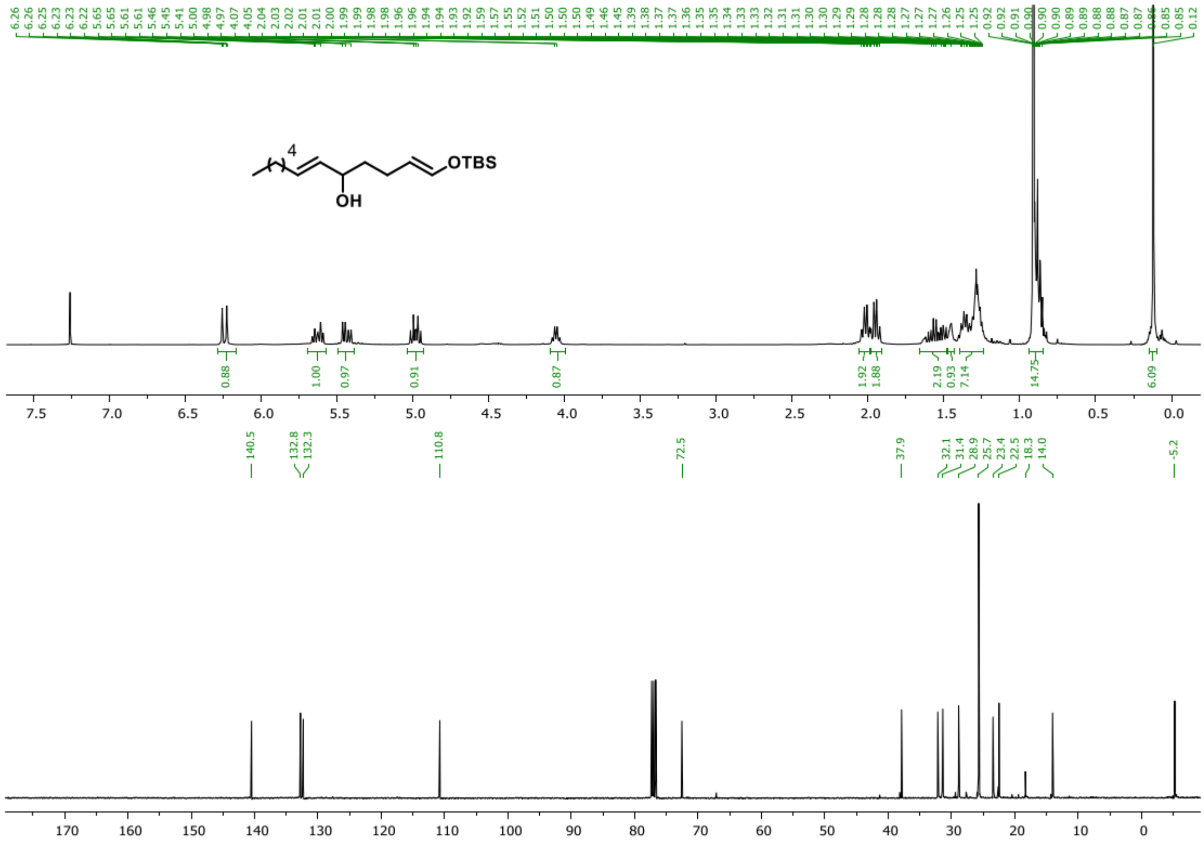
4
OTBS
OH
140.5
132.8
132.3
110.8
72.5
37.9
32.1
31.4
28.9
25.7
23.4
22.5
18.3
14.0
-5.2

***anti*-4-methyl-1-phenyl-2-((triisopropylsilyl)oxy)pent-4-en-1-ol (9l).**

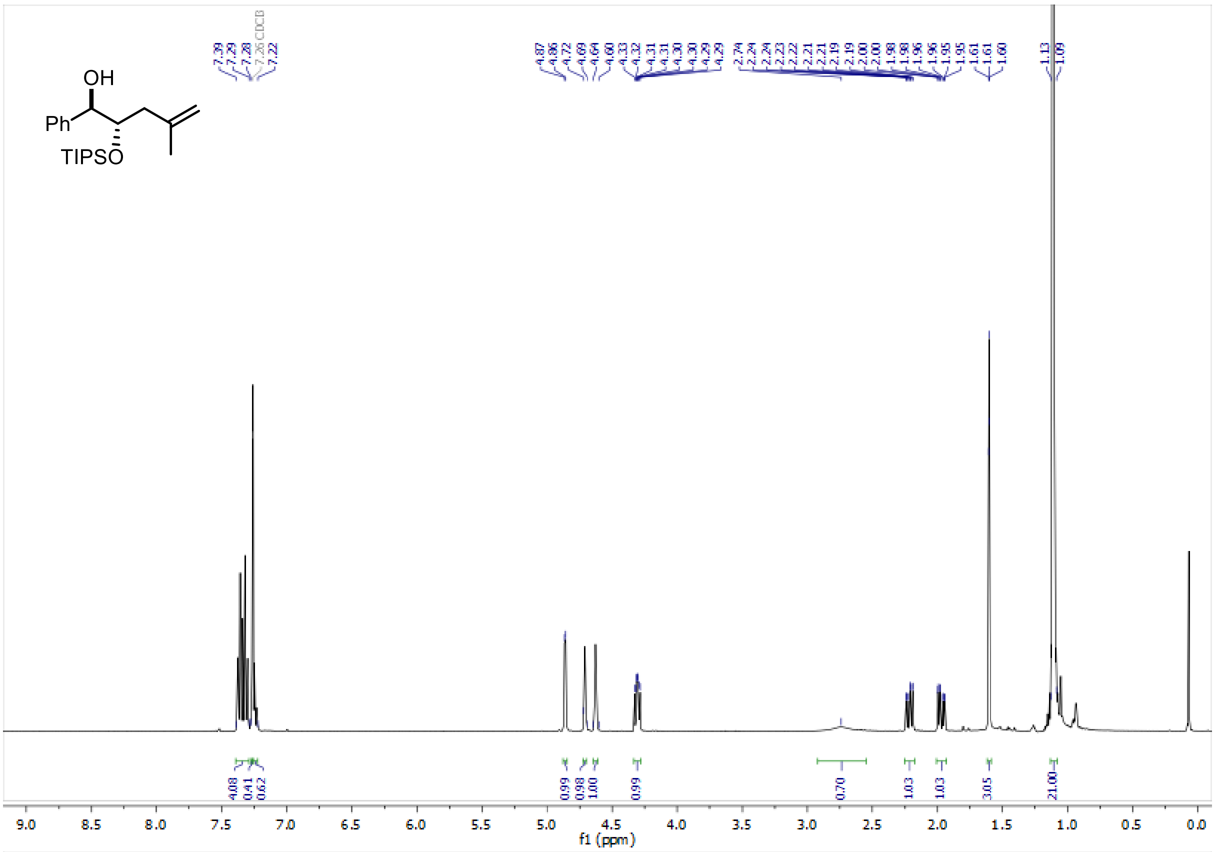

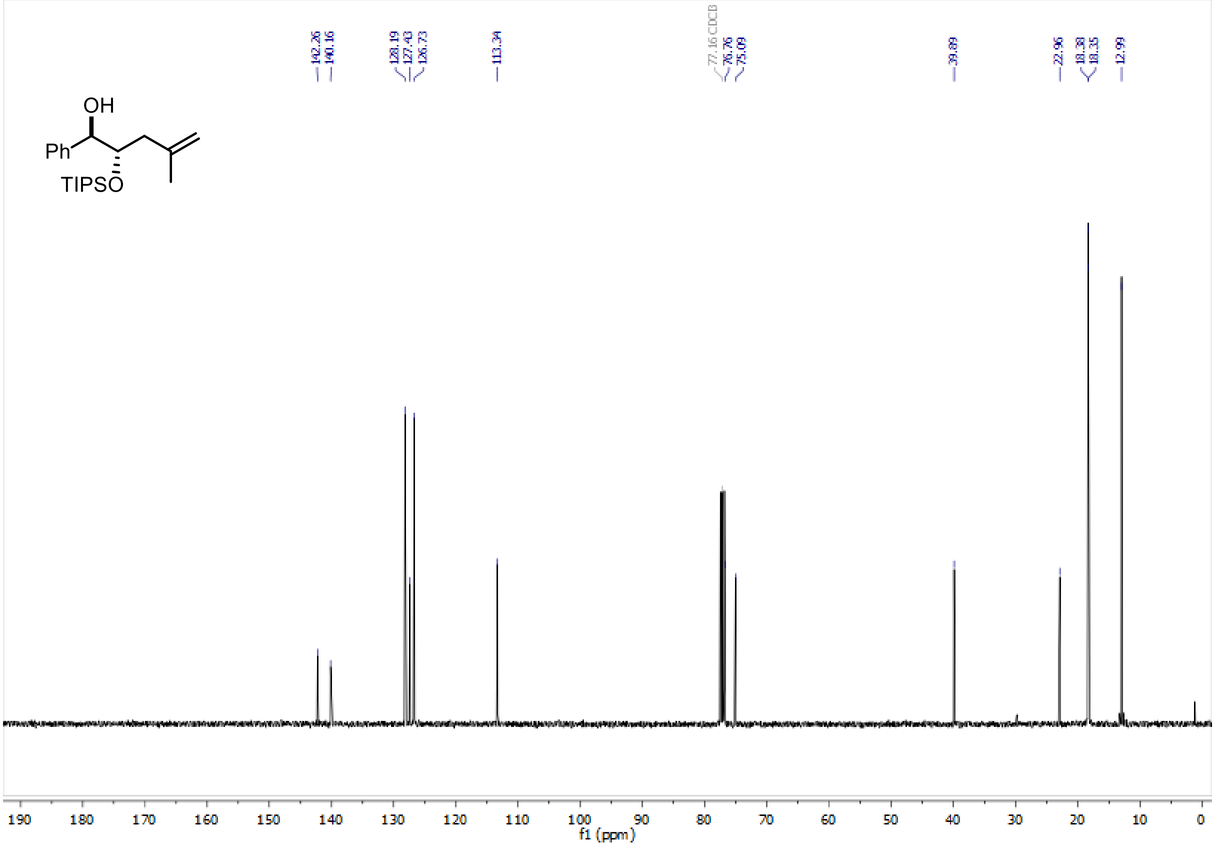

***anti*-2-((tert-butyldimethylsilyl)oxy)-4-methyl-1-phenylpent-4-en-1-ol (9m).**

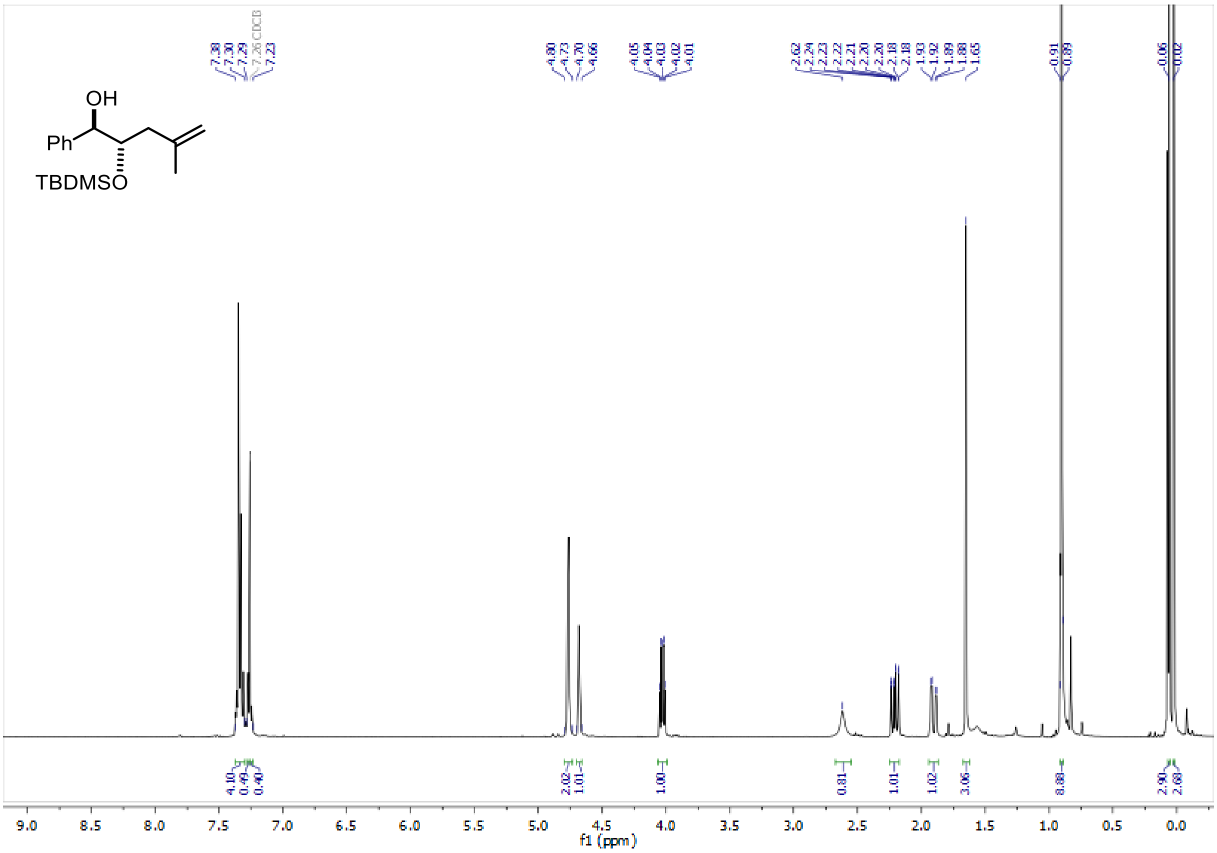

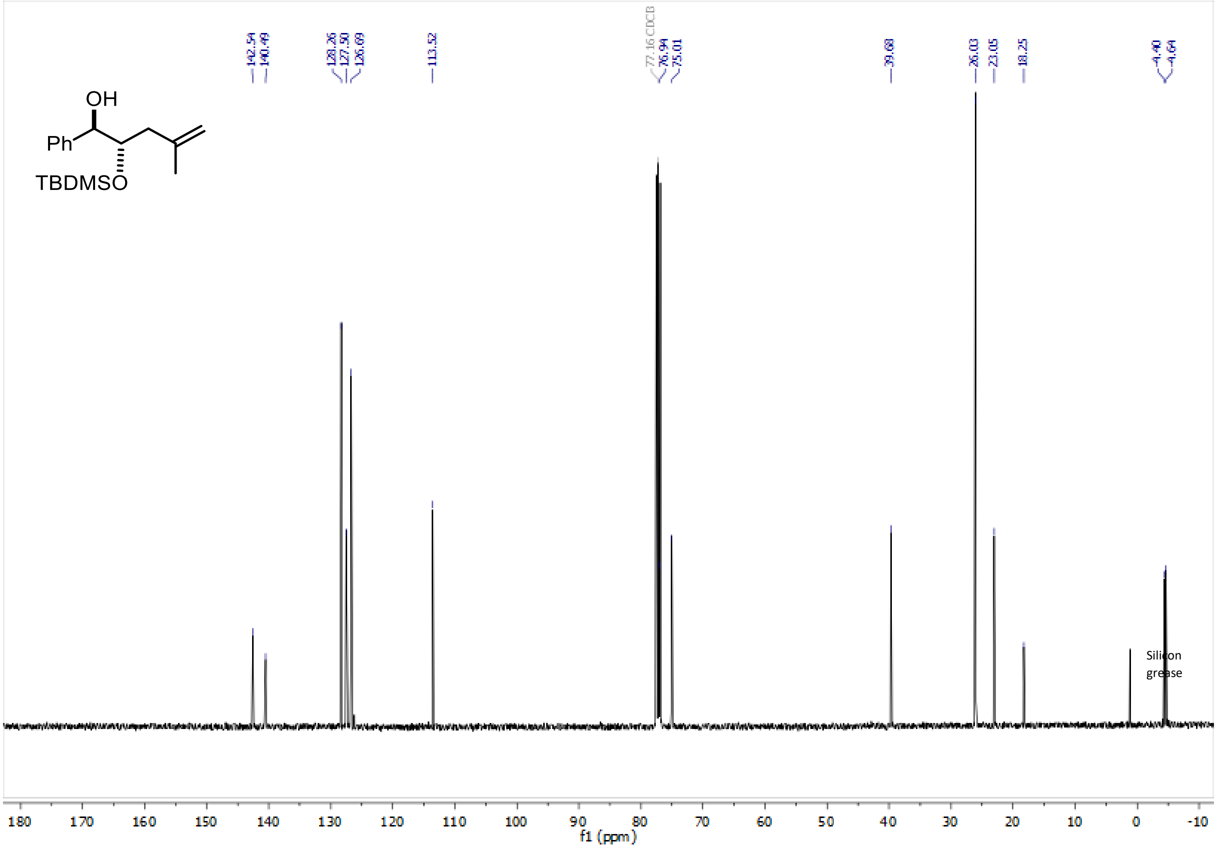

Methyl 4-(*anti*-2-((*tert*-butyldimethylsilyl)oxy)-1-hydroxypent-4-en-1-yl)benzoate (9n)

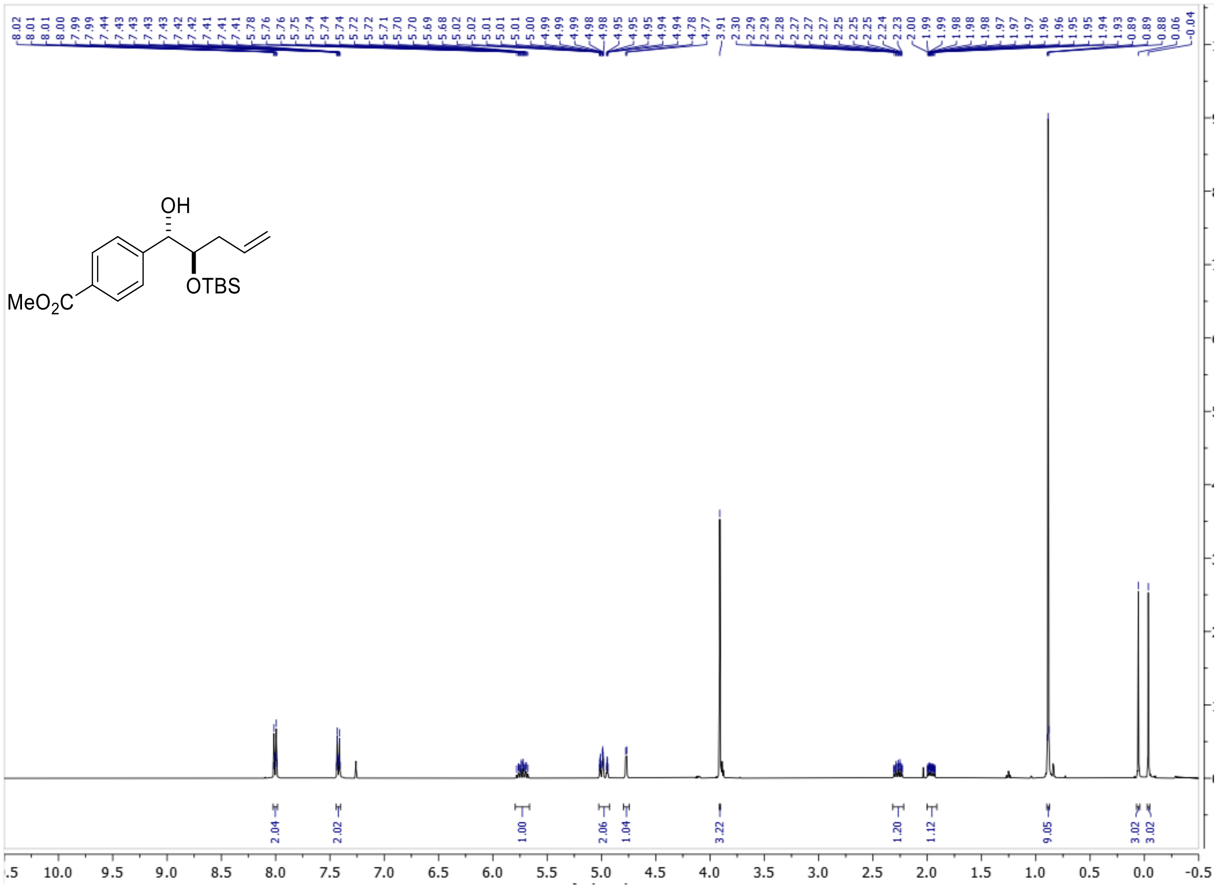

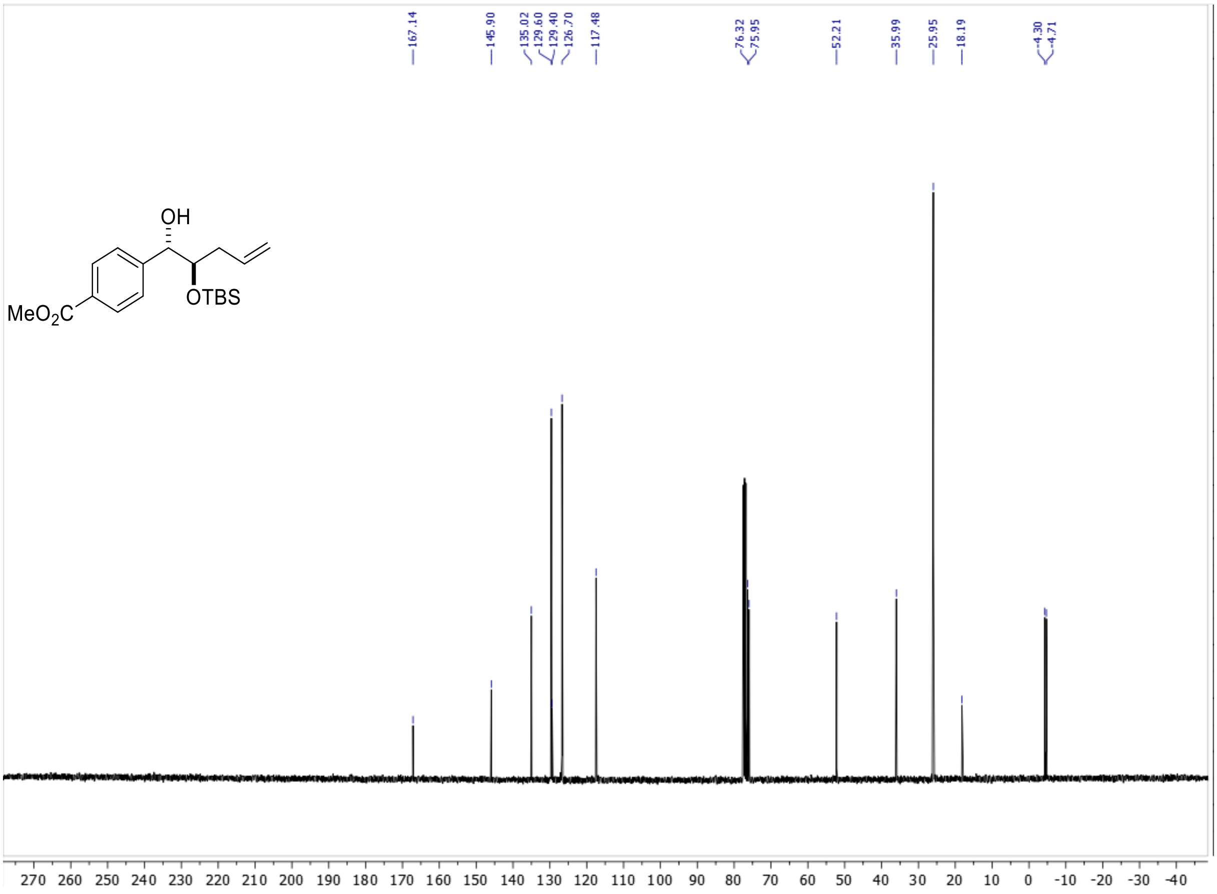

4-(*anti*-2-((*tert*-butyldimethylsilyl)oxy)-1-hydroxypent-4-en-1-yl)benzonitrile (9o)

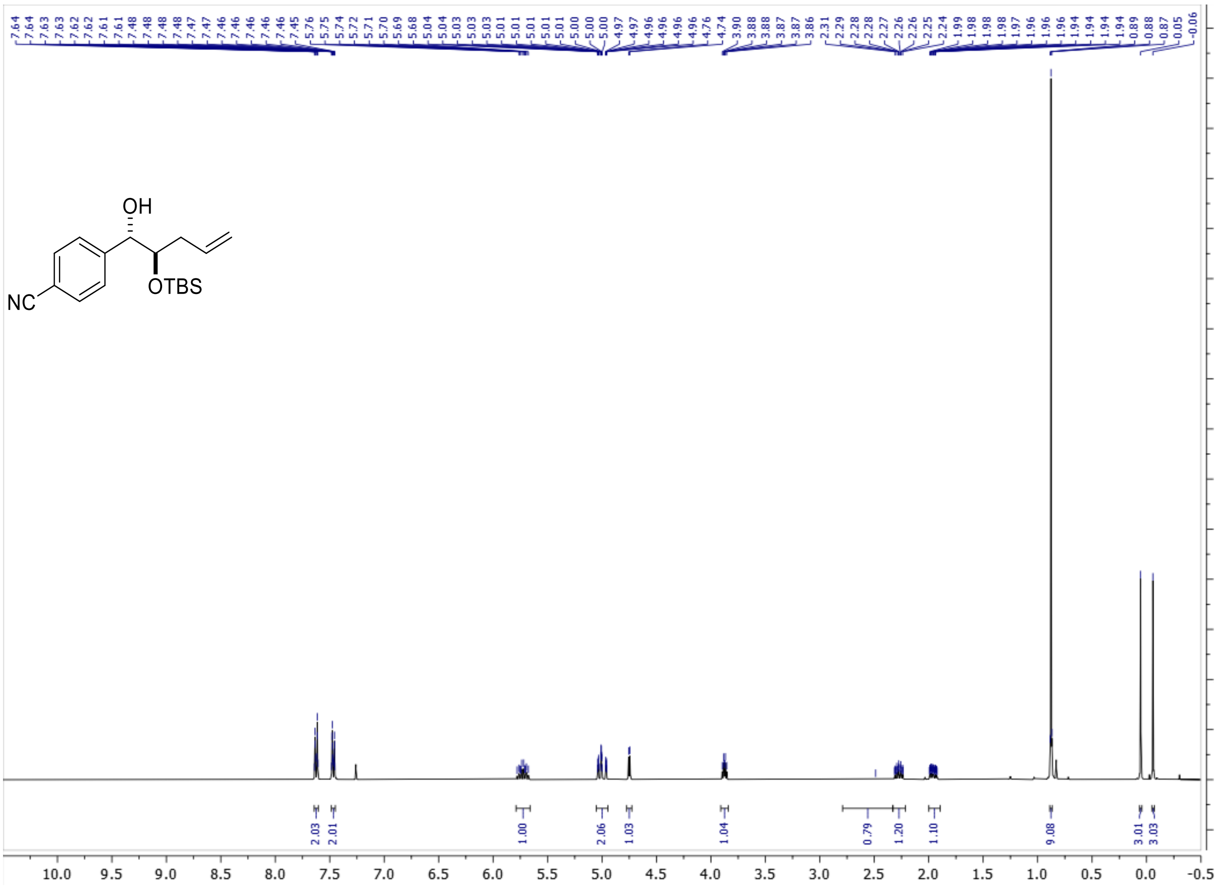

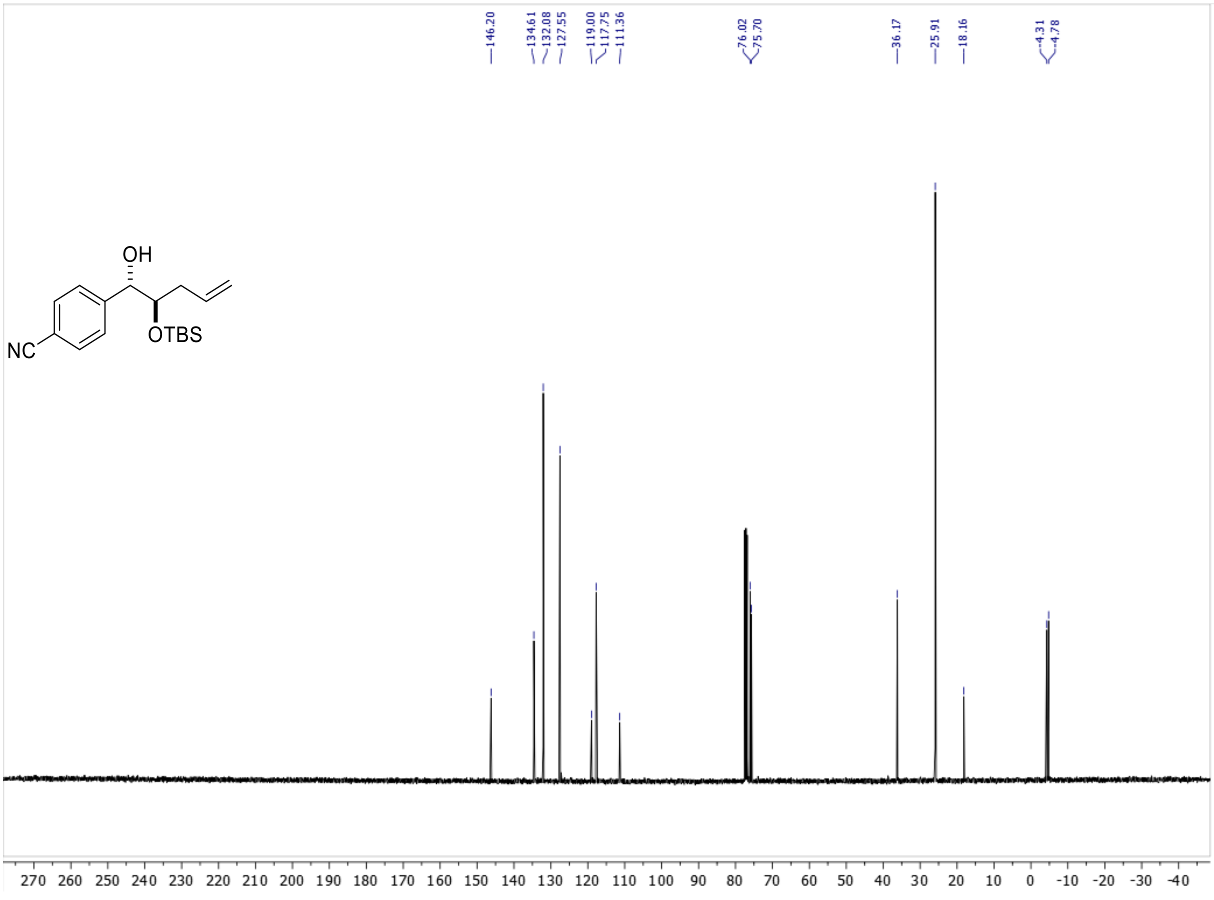

1-(3-(*anti*-2-((*tert*-butyldimethylsilyl)oxy)-1-hydroxypent-4-en-1-yl)phenyl)ethan-1-one (9p)

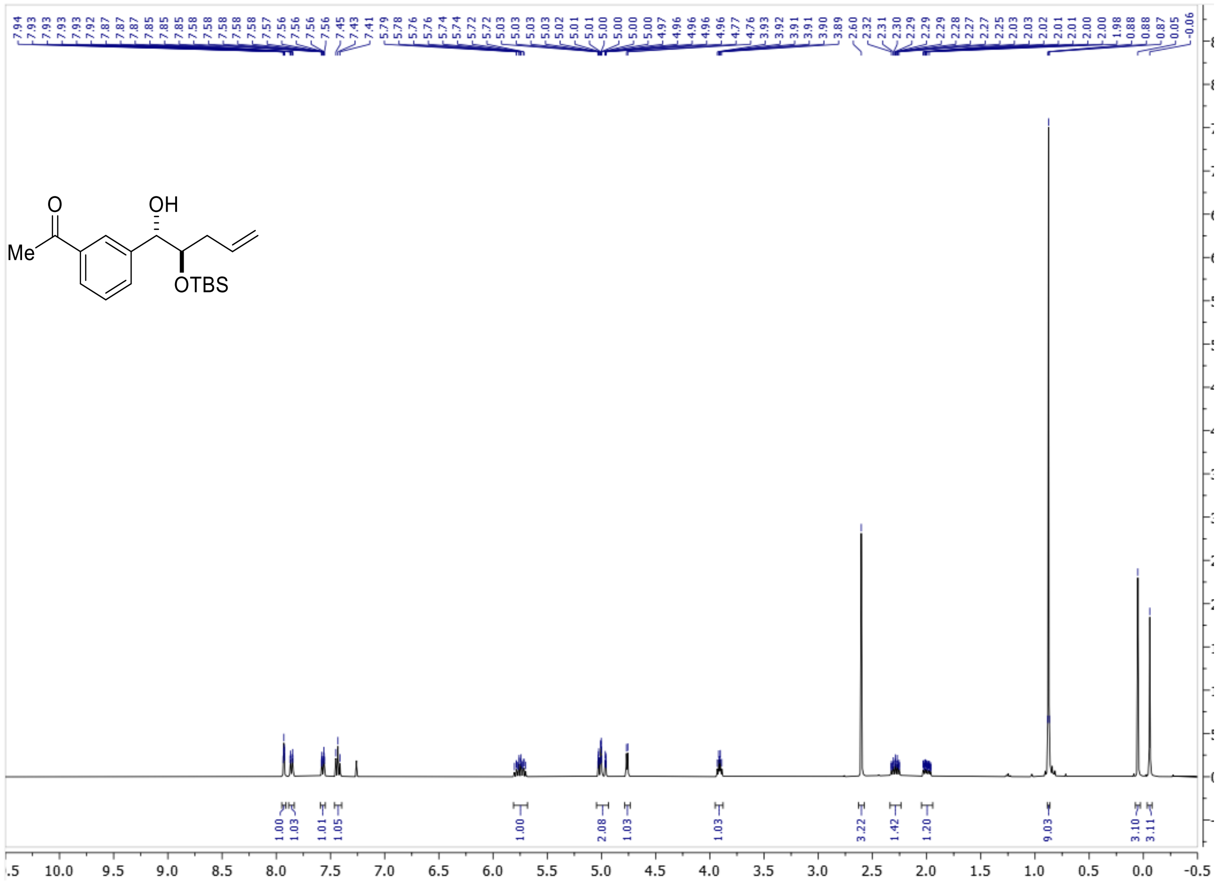

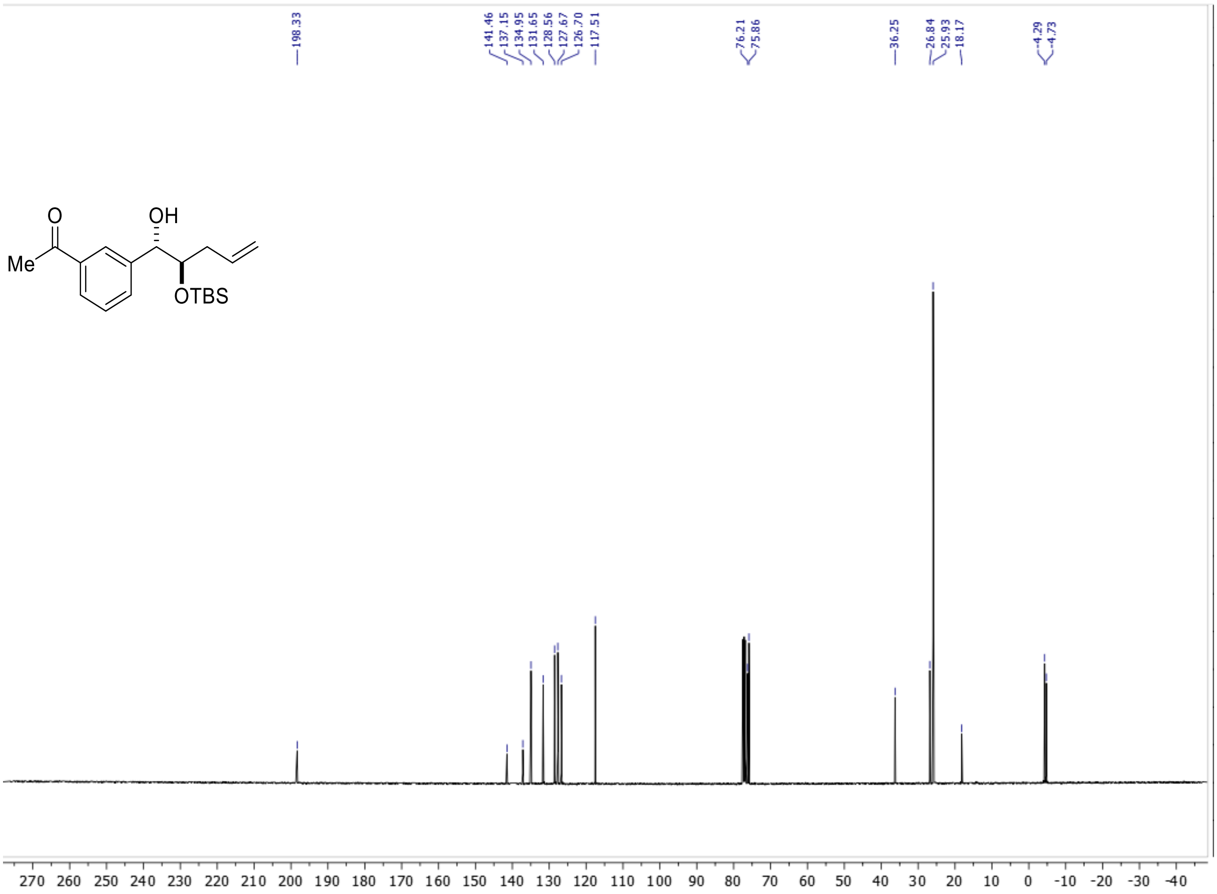

***anti*-1-(furan-2-yl)-4-methyl-2-((triisopropylsilyl)oxy)pent-4-en-1-ol (9q).**

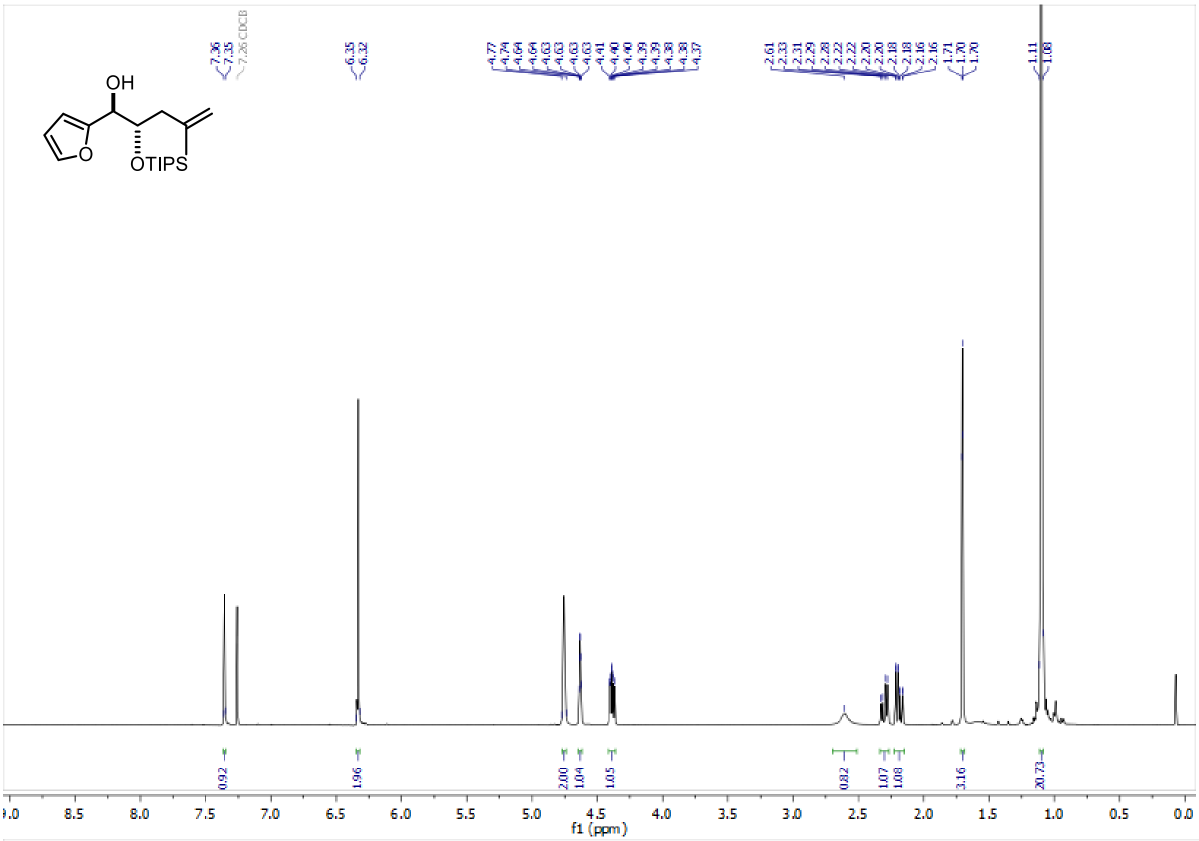

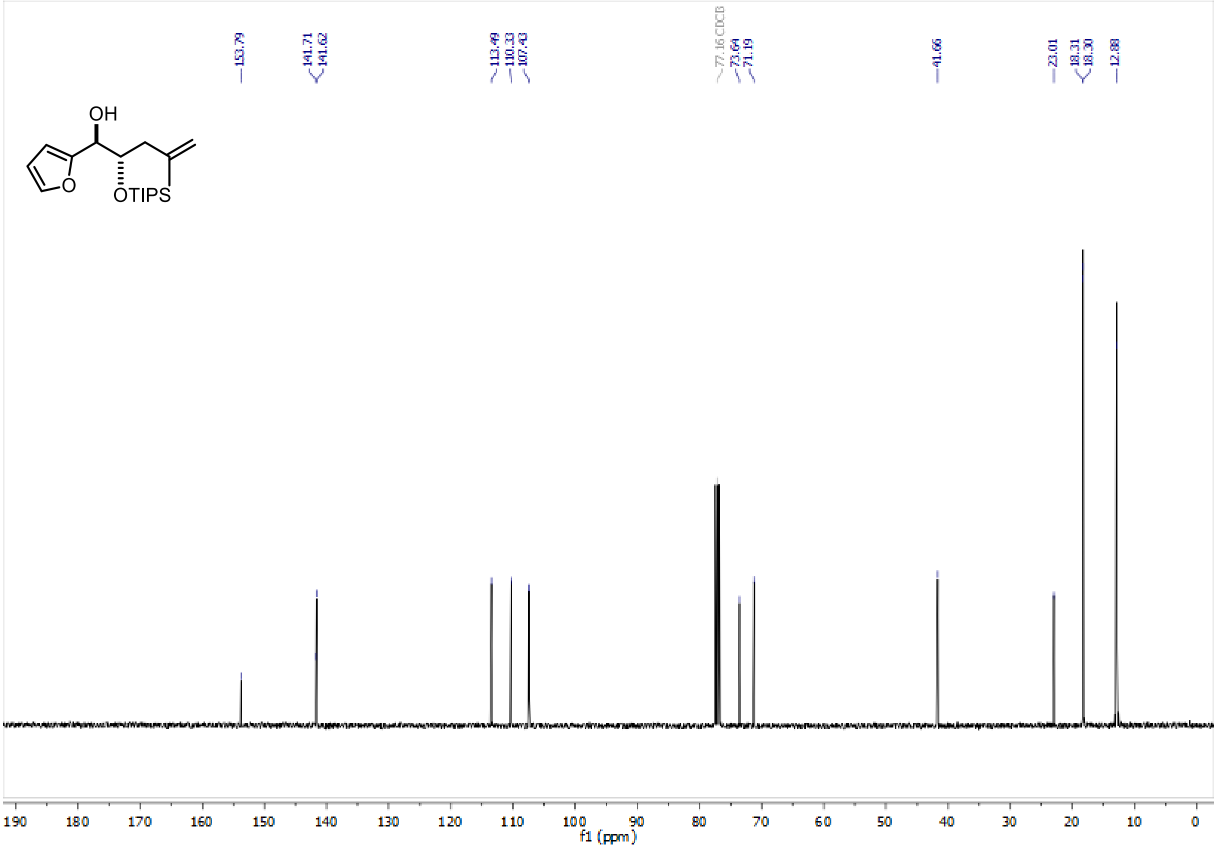

***anti*-4-methyl-1-(thiophen-2-yl)-2-((triisopropylsilyl)oxy)pent-4-en-1-ol (9r).**

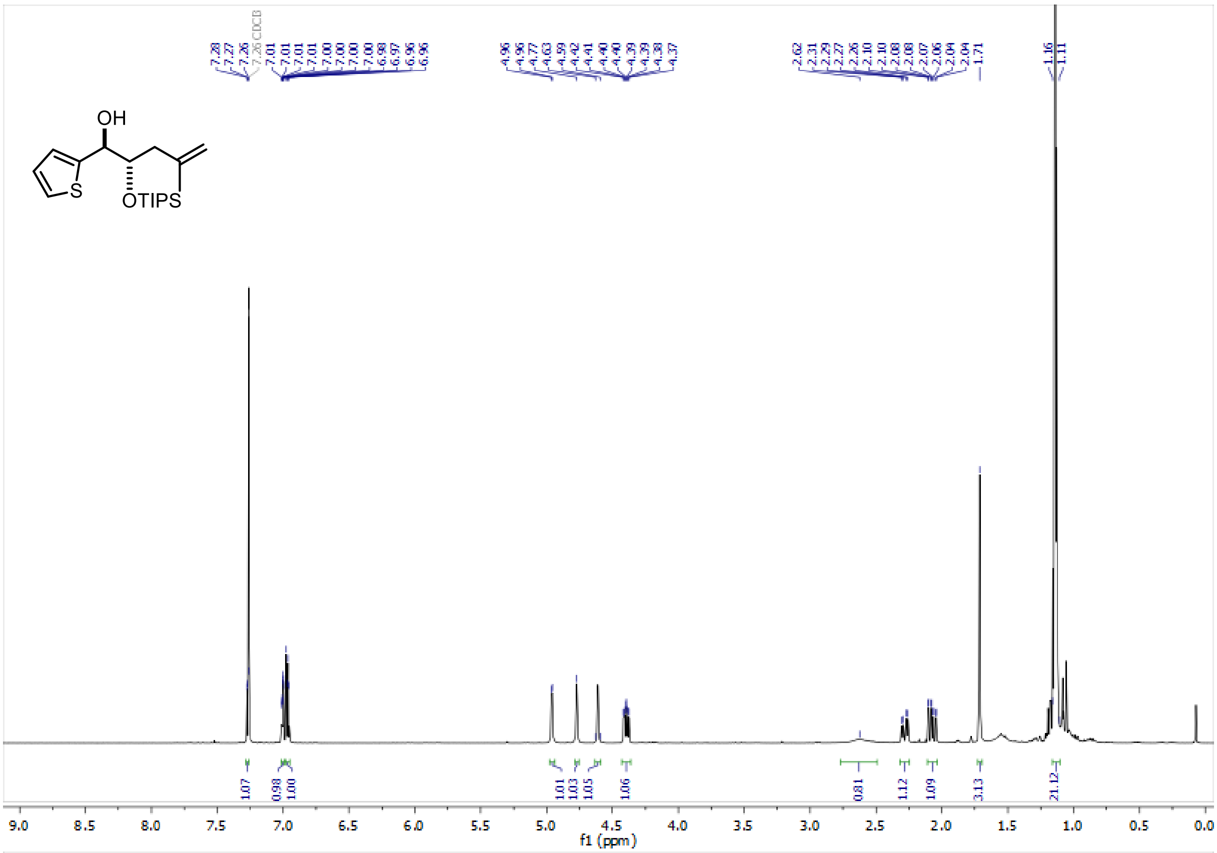

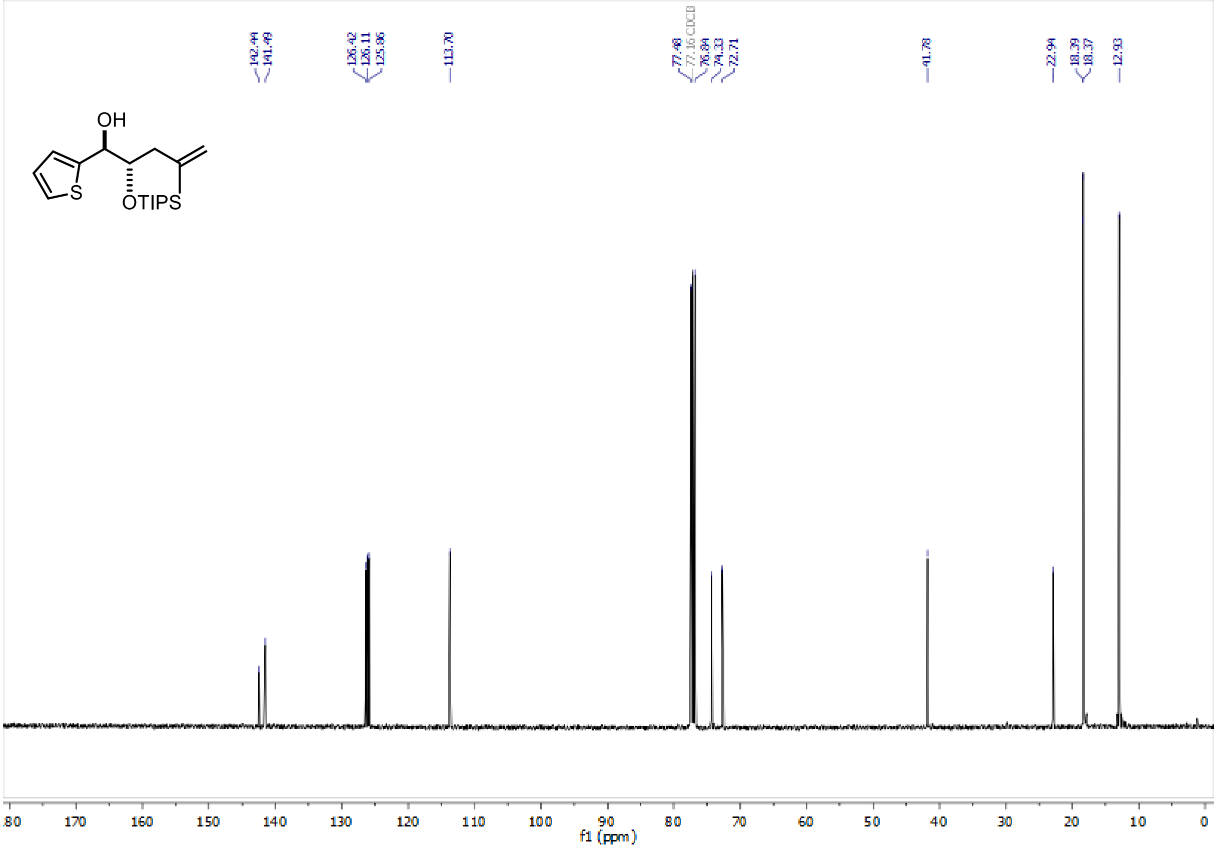

syn-4-((tert-butyldimethylsilyl)oxy)-1-phenylhept-6-en-3-ol **11a**

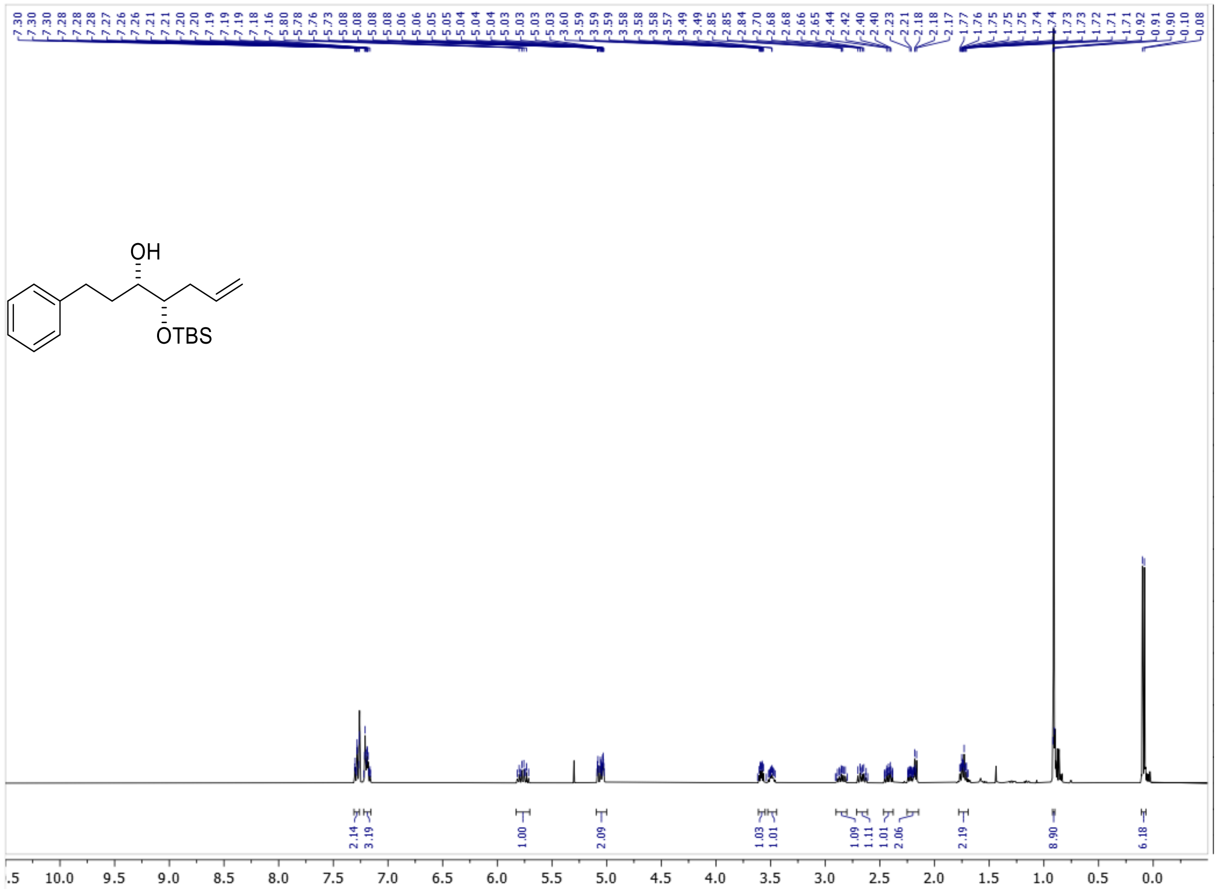

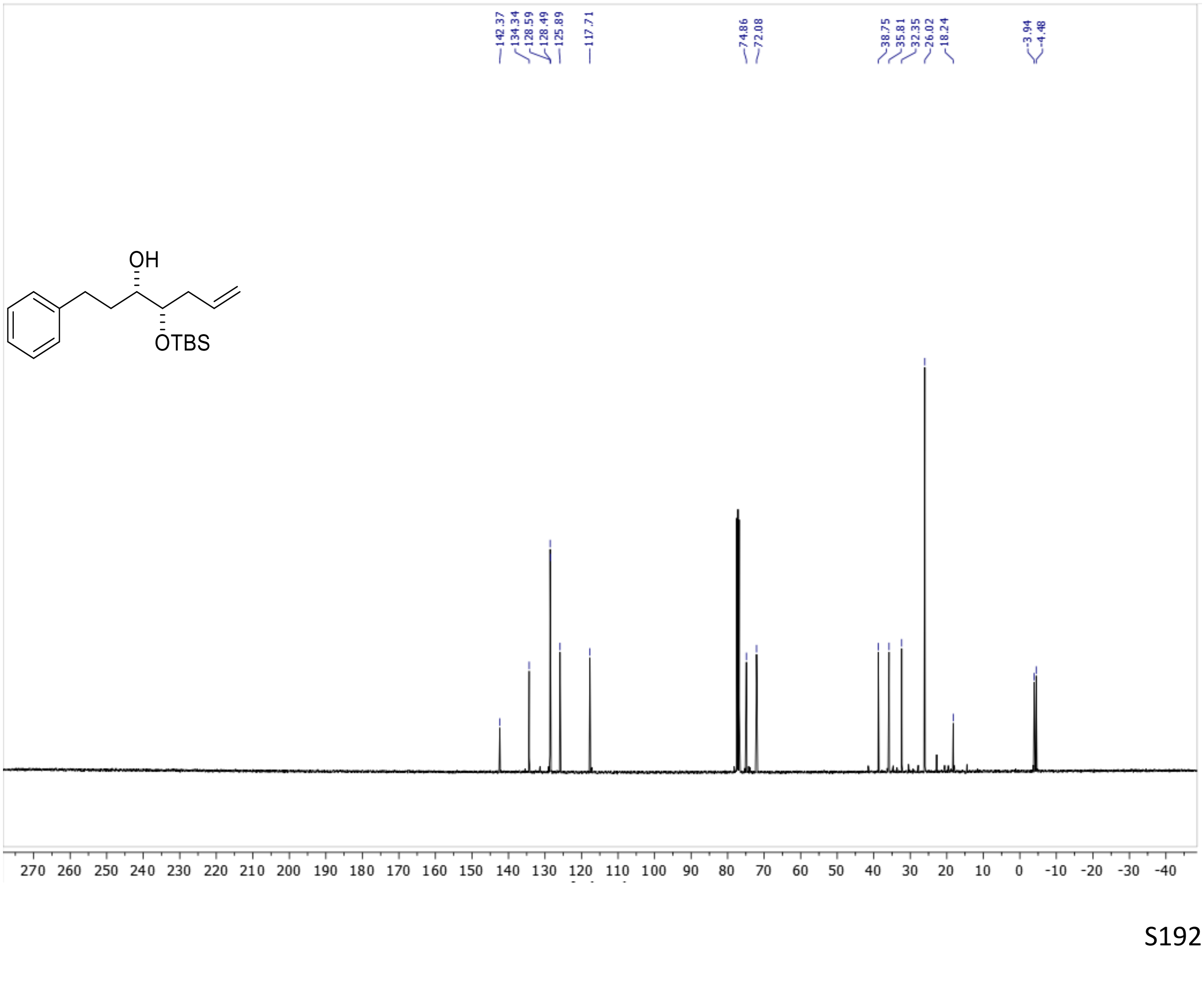

syn-2-((tert-butyldimethylsilyl)oxy)-1-phenylpent-4-en-1-ol 11b

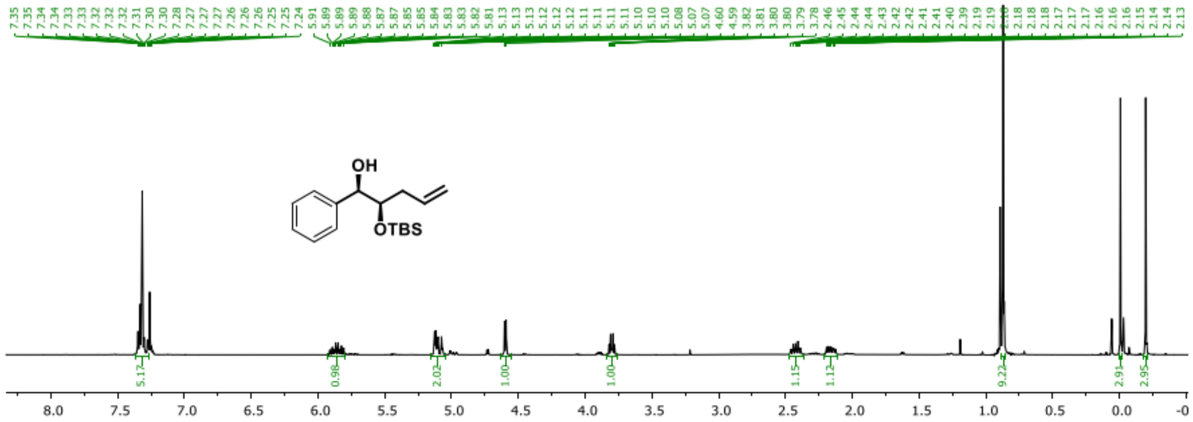

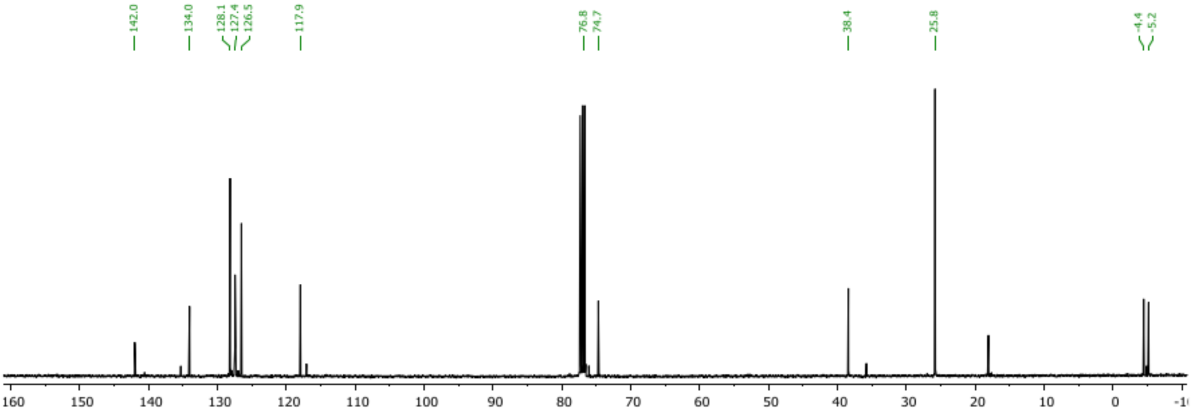

***syn*-5-methyl-1-phenyl-4-((triisopropylsilyl)oxy)hept-6-en-3-ol (13).**

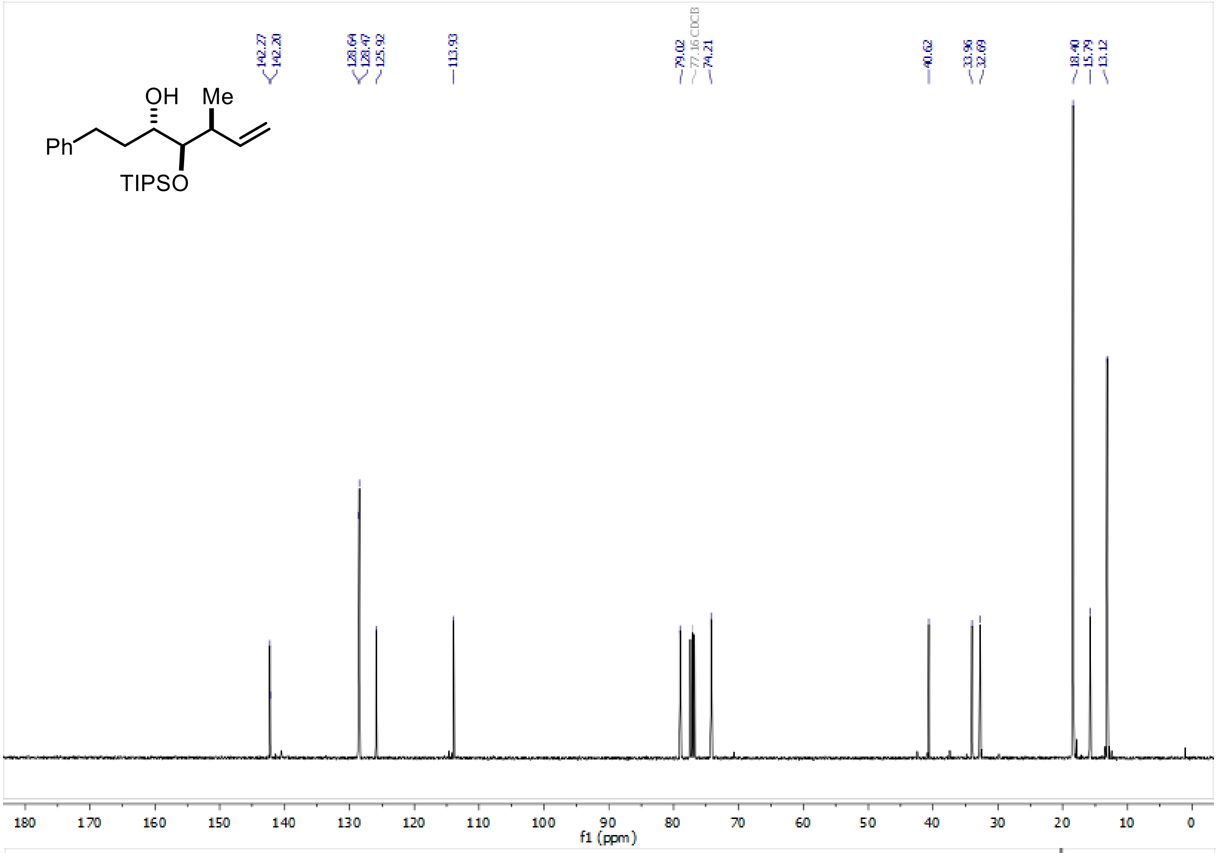

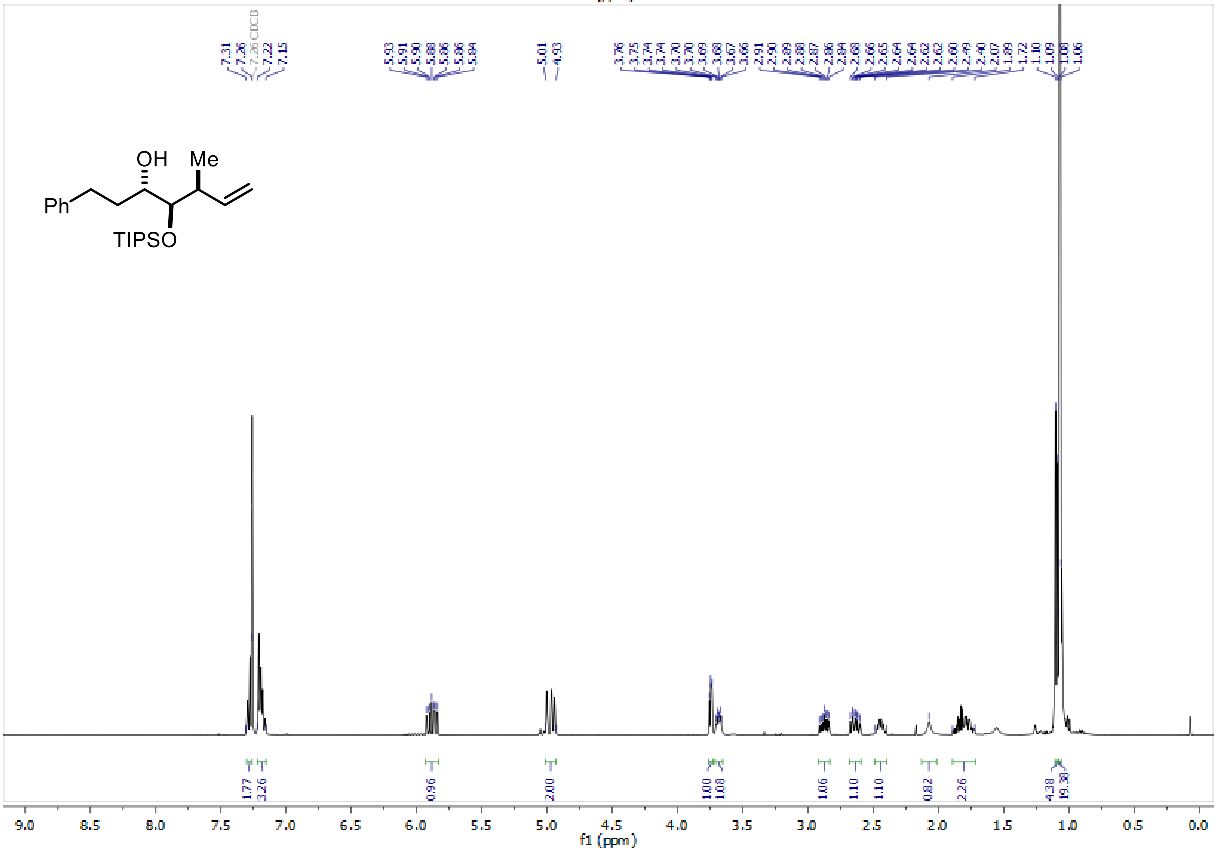

anti-4-((tert-butyldimethylsilyl)oxy)-1-phenylhept-6-en-3-ol **18a**

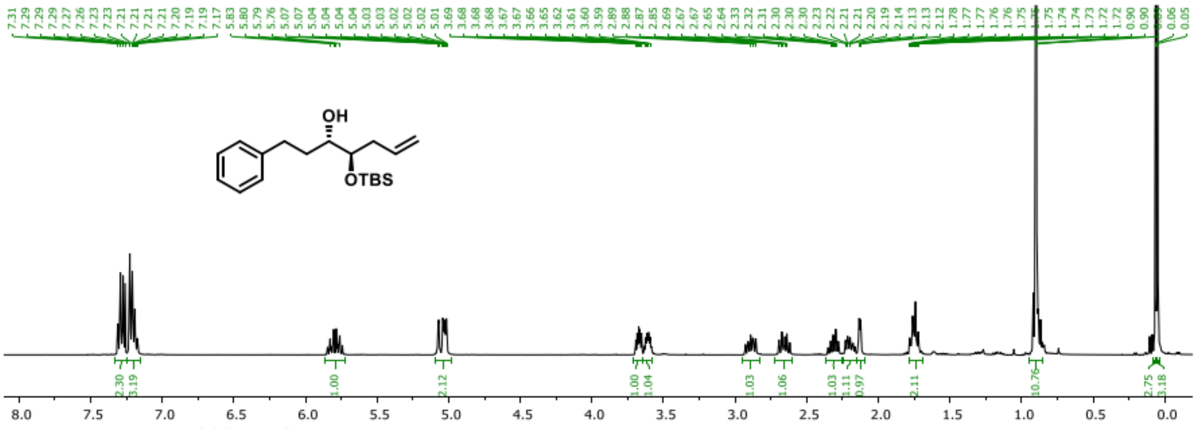

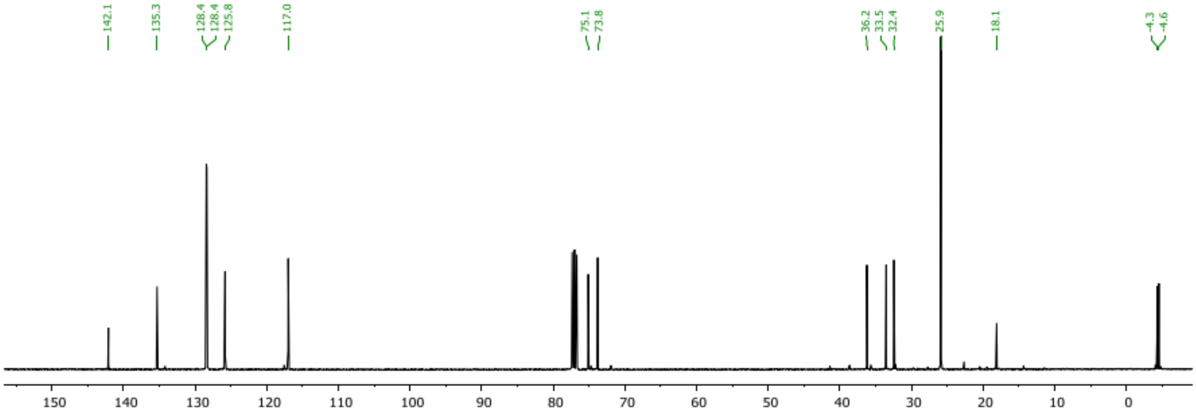

anti-1-phenyl-4-((triethylsilyl)oxy)hept-6-en-3-ol **18b**

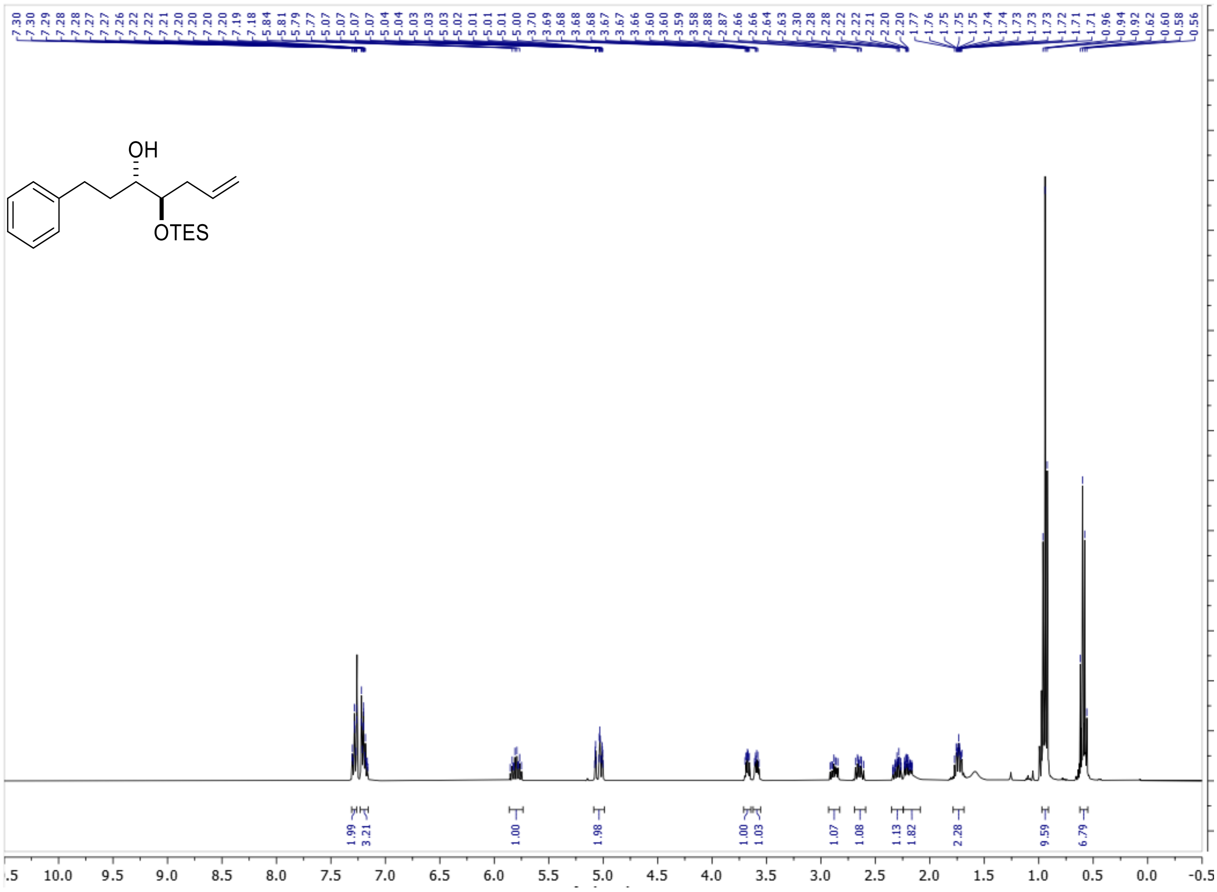

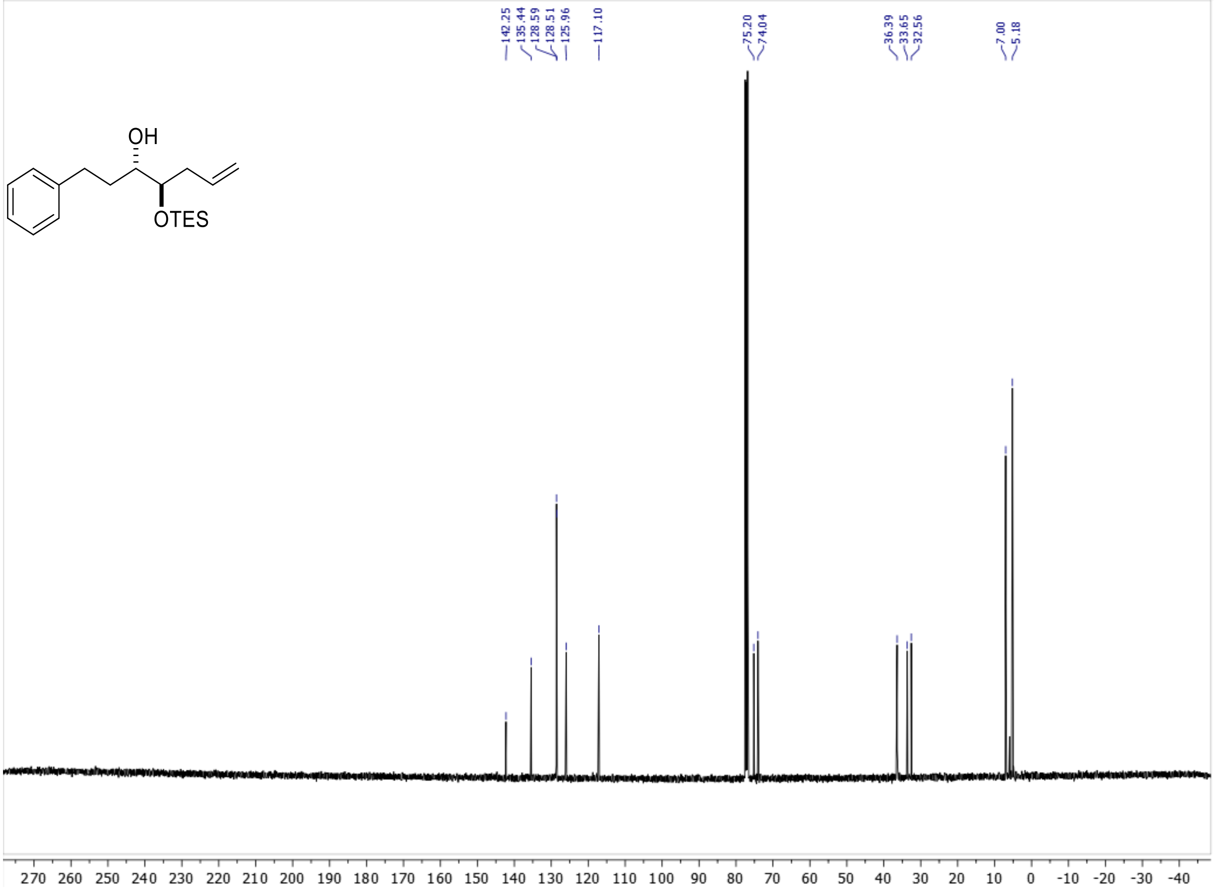

anti-2-((tert-butyldimethylsilyl)oxy)-1-cyclohexylpent-4-en-1-ol **18c**

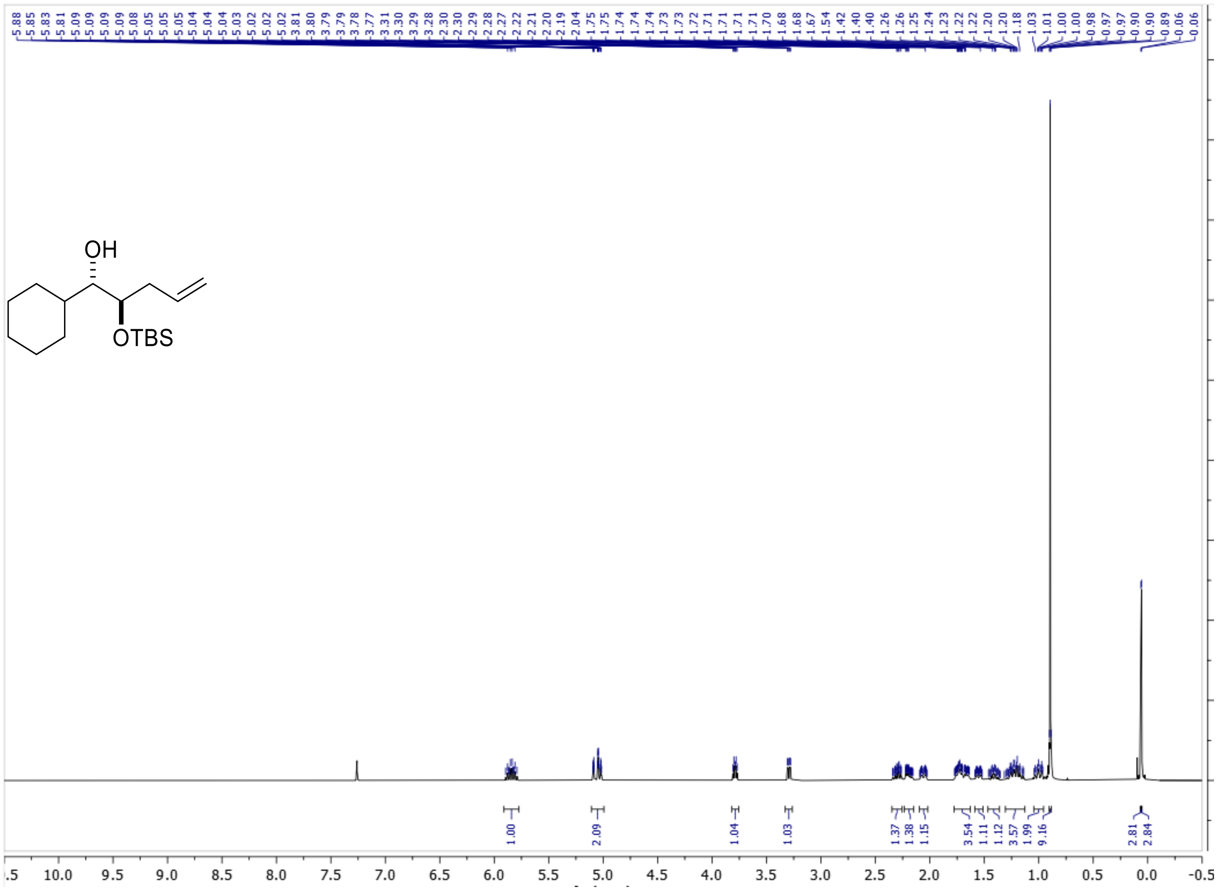

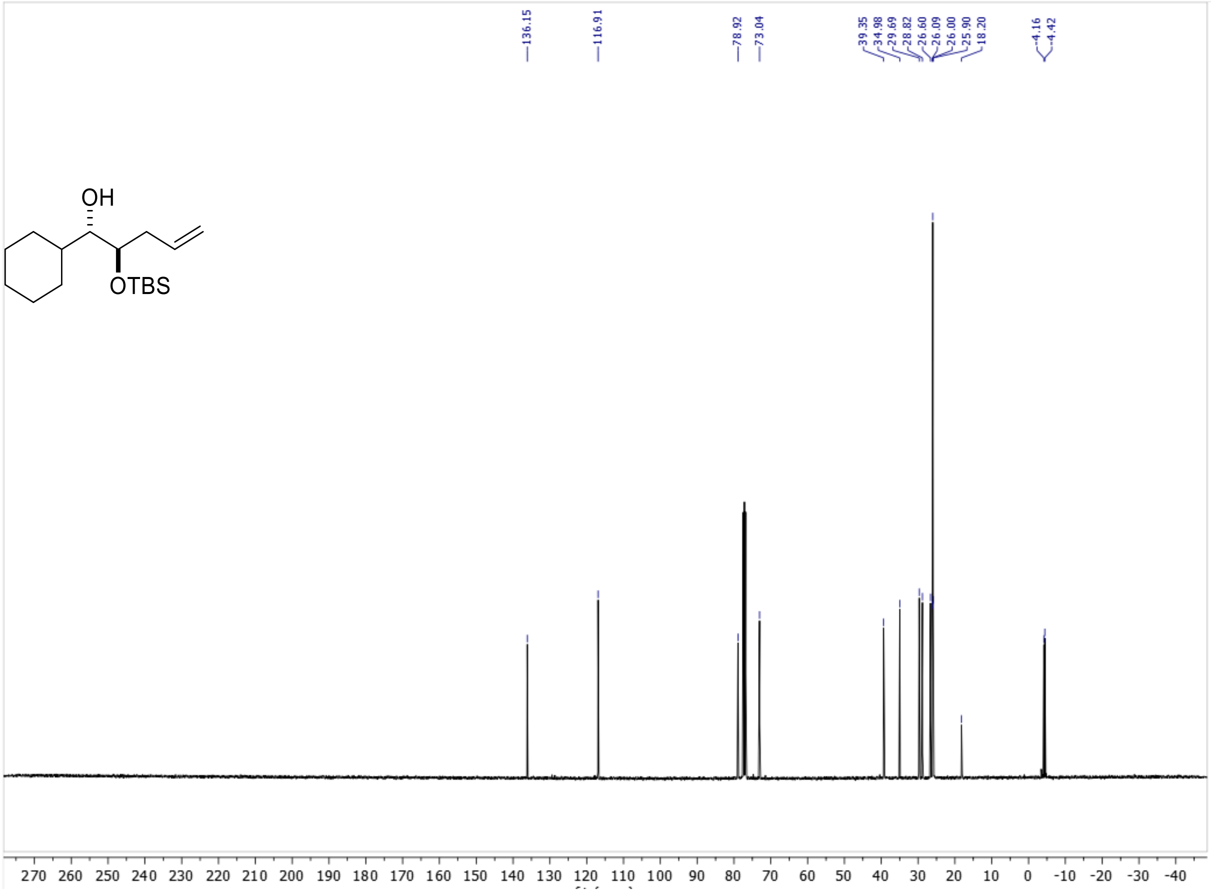

anti-1-cyclohexyl-2-((triisopropylsilyl)oxy)pent-4-en-1-ol **18d**

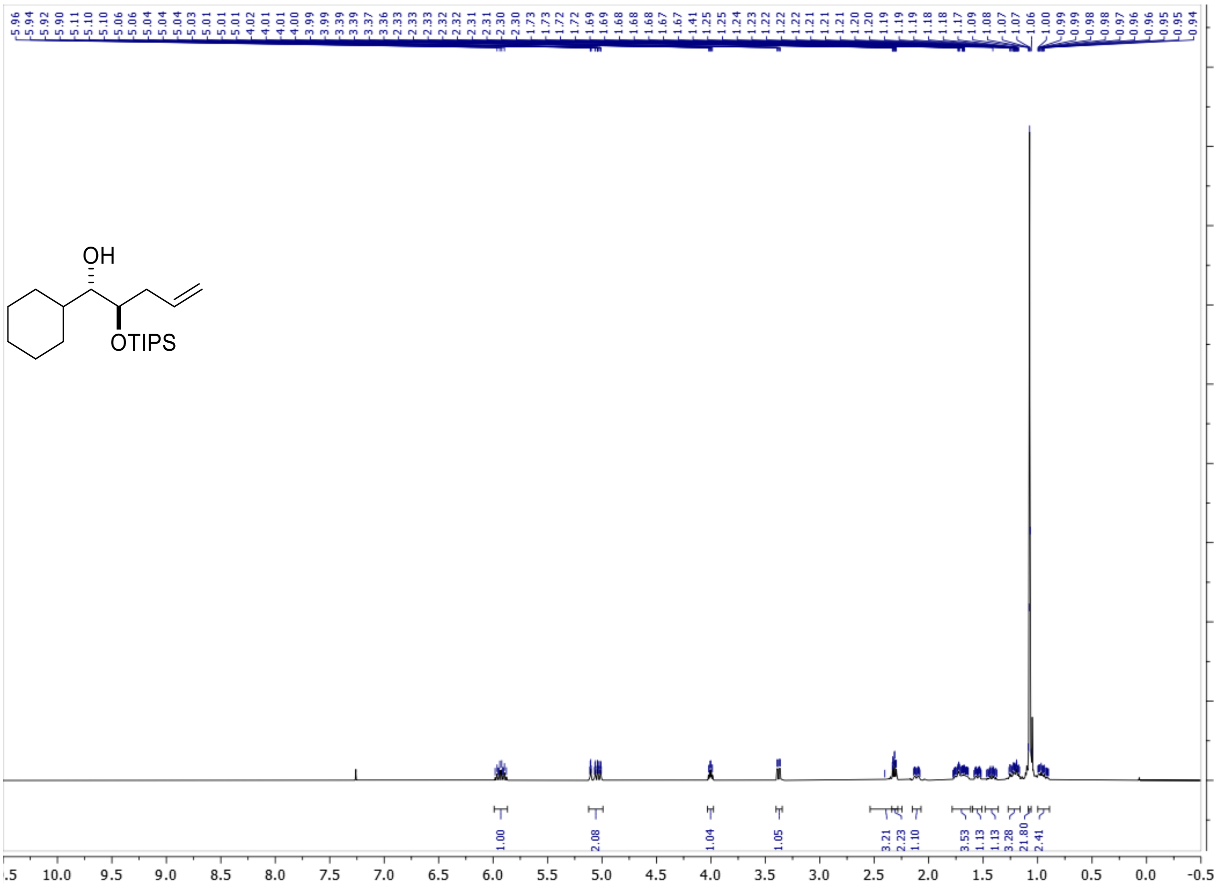

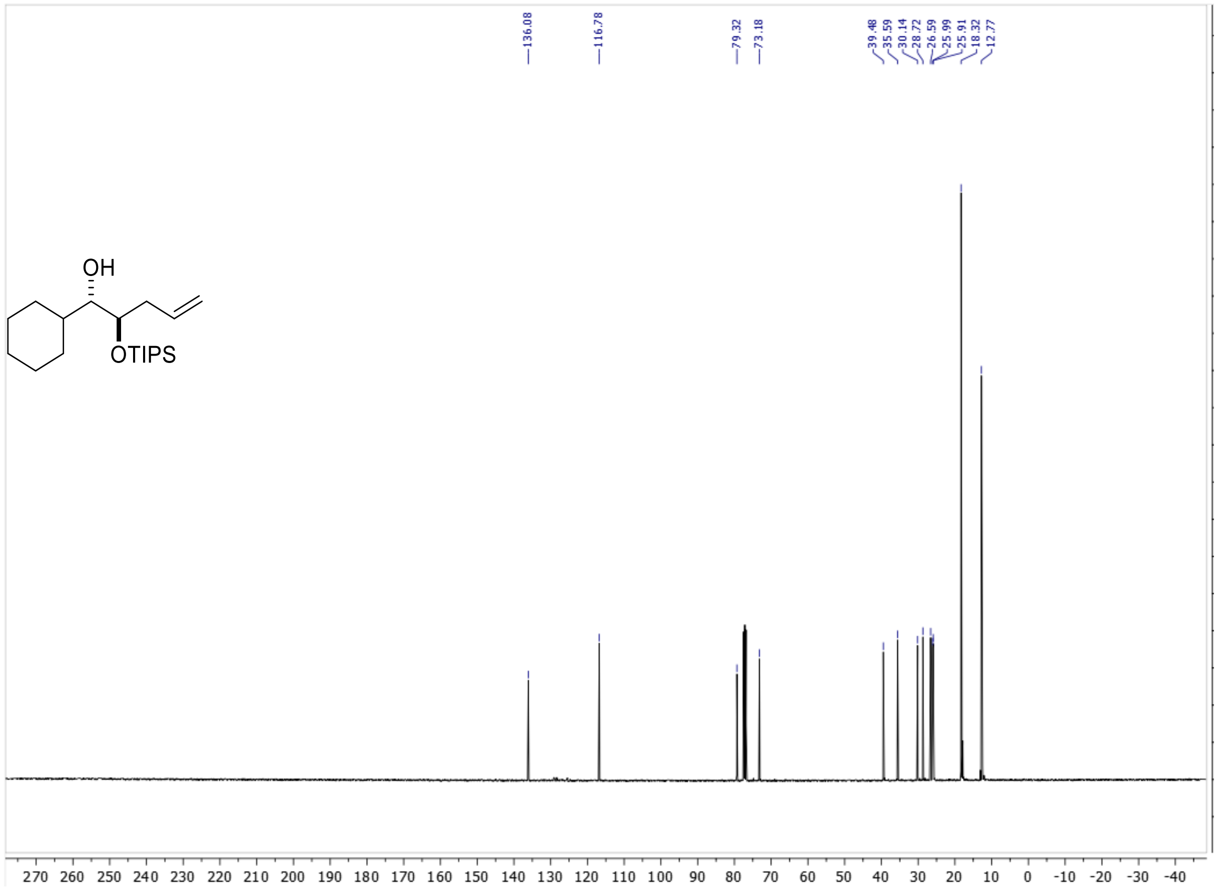

anti-1-([1,1'-biphenyl]-4-yl)-2-((triisopropylsilyl)oxy)pent-4-en-1-ol **18e**

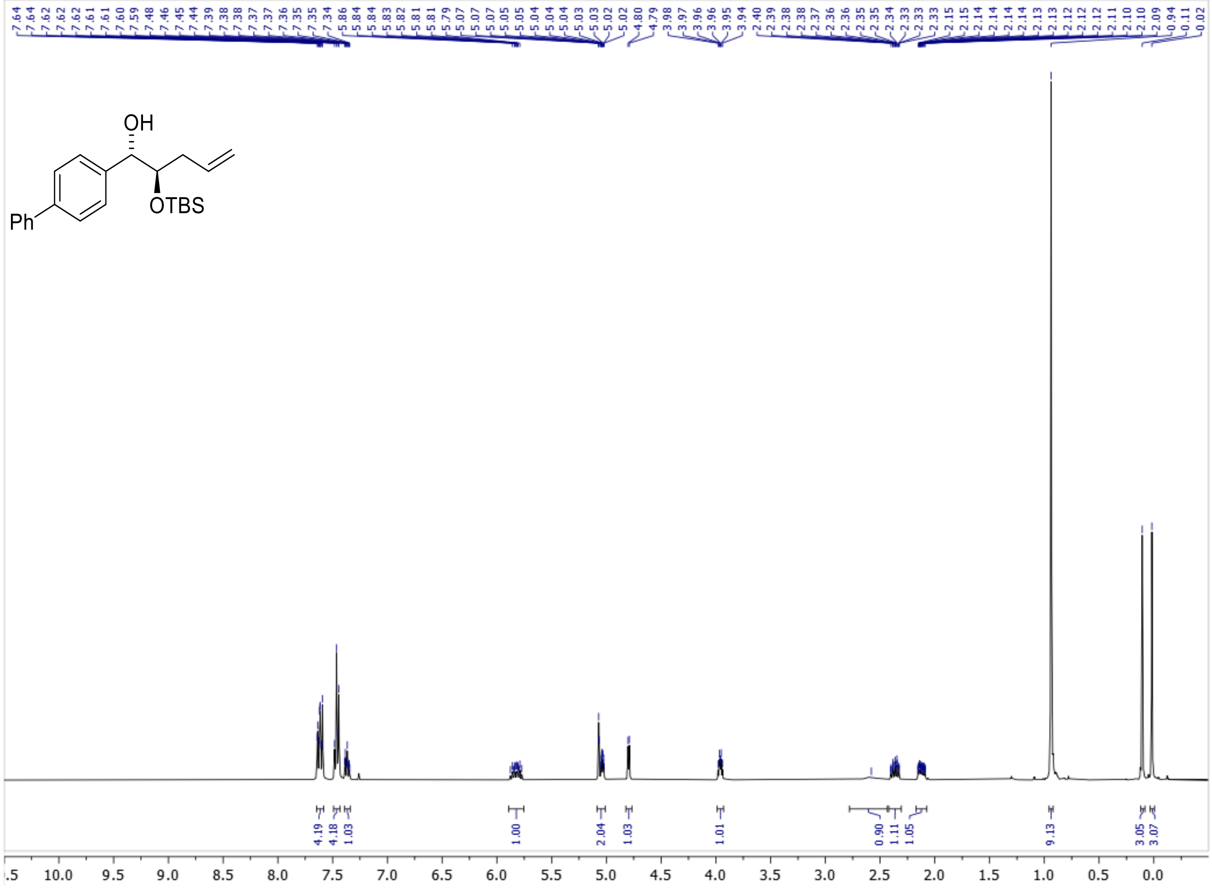

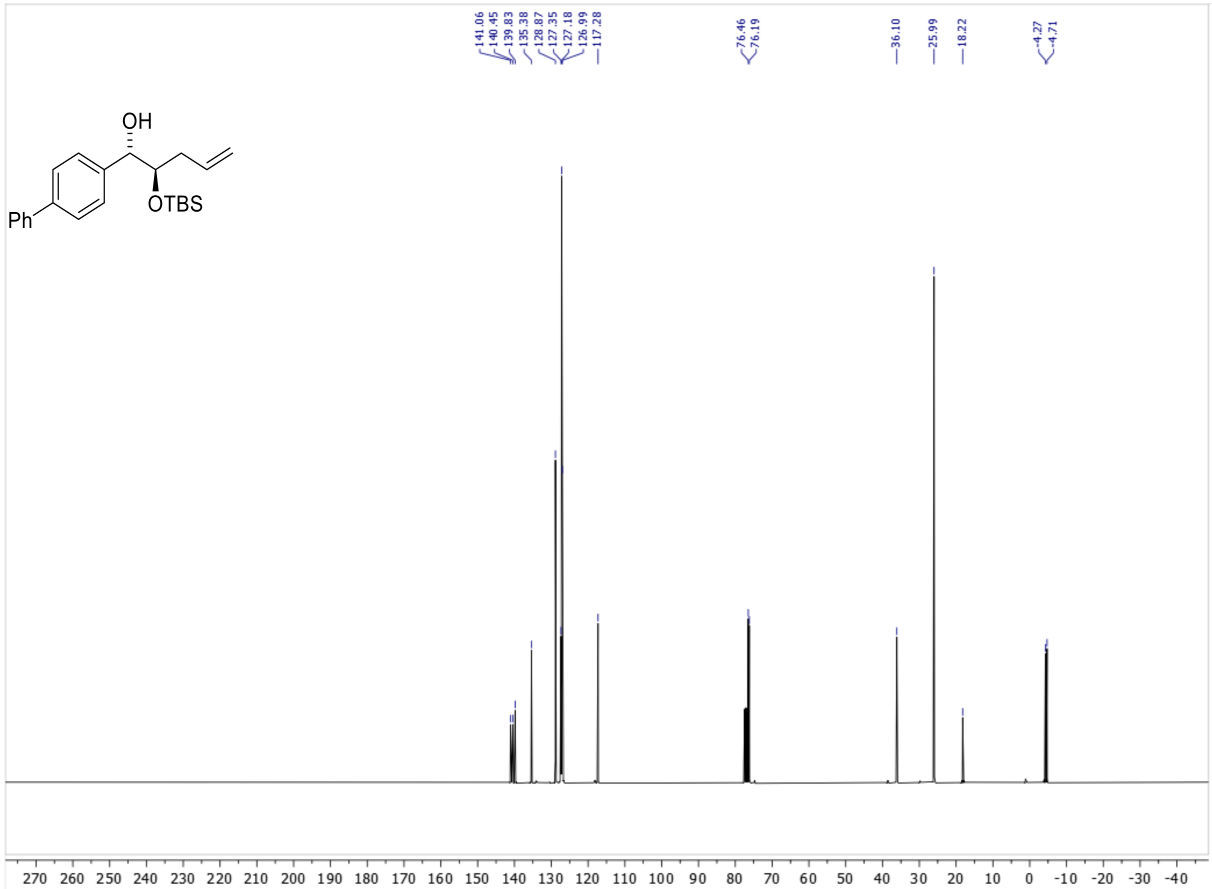

anti-1-(4-methoxyphenyl)-2-((triisopropylsilyl)oxy)pent-4-en-1-ol **18f**

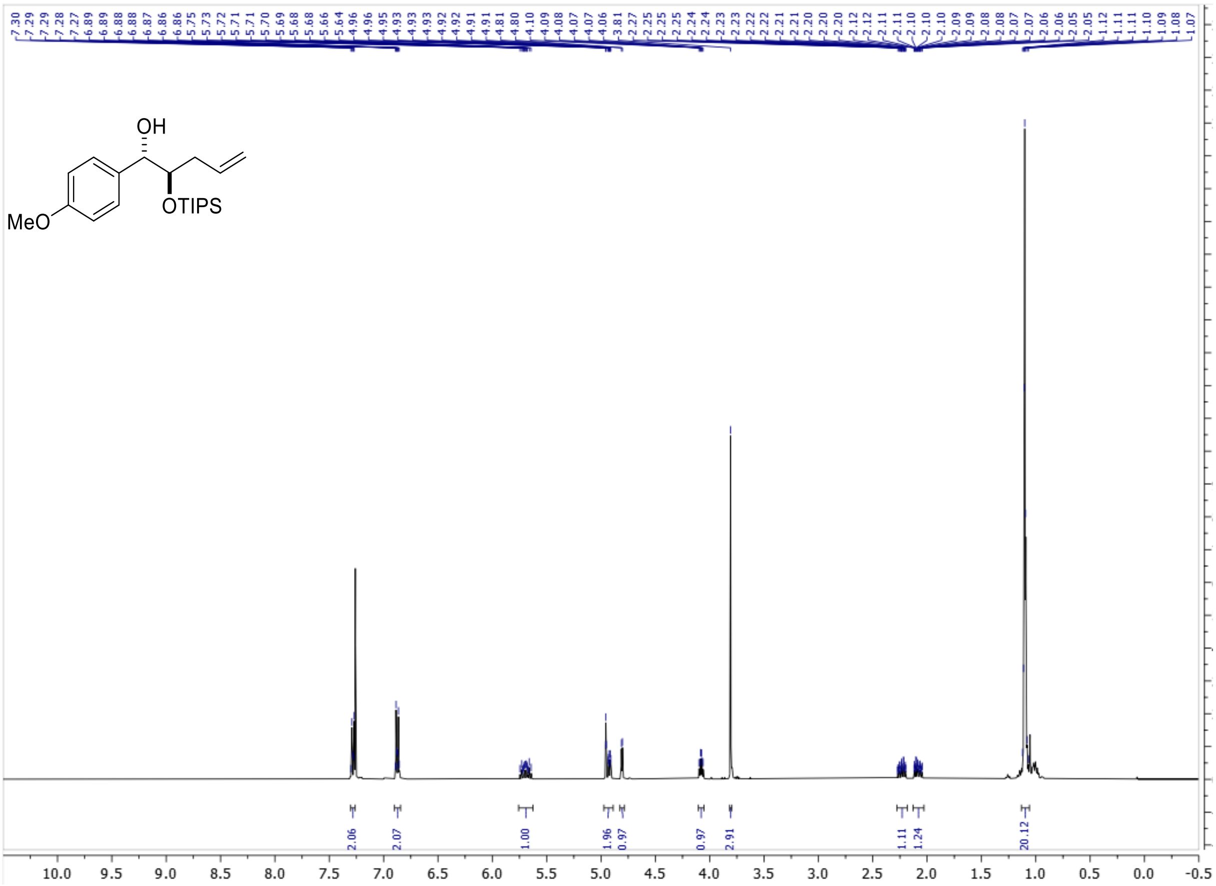

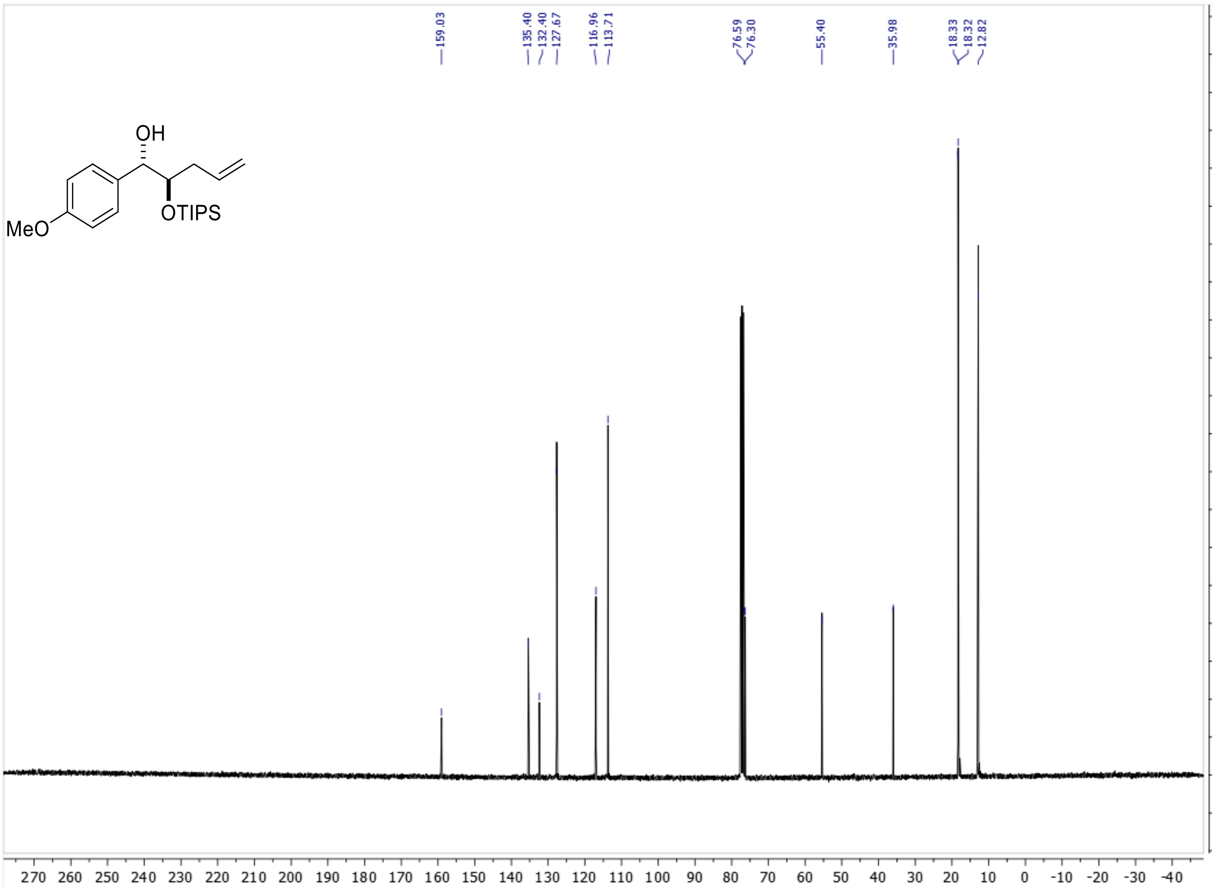

anti-1-(*o*-tolyl)-2-((triisopropylsilyl)oxy)pent-4-en-1-ol **18g**

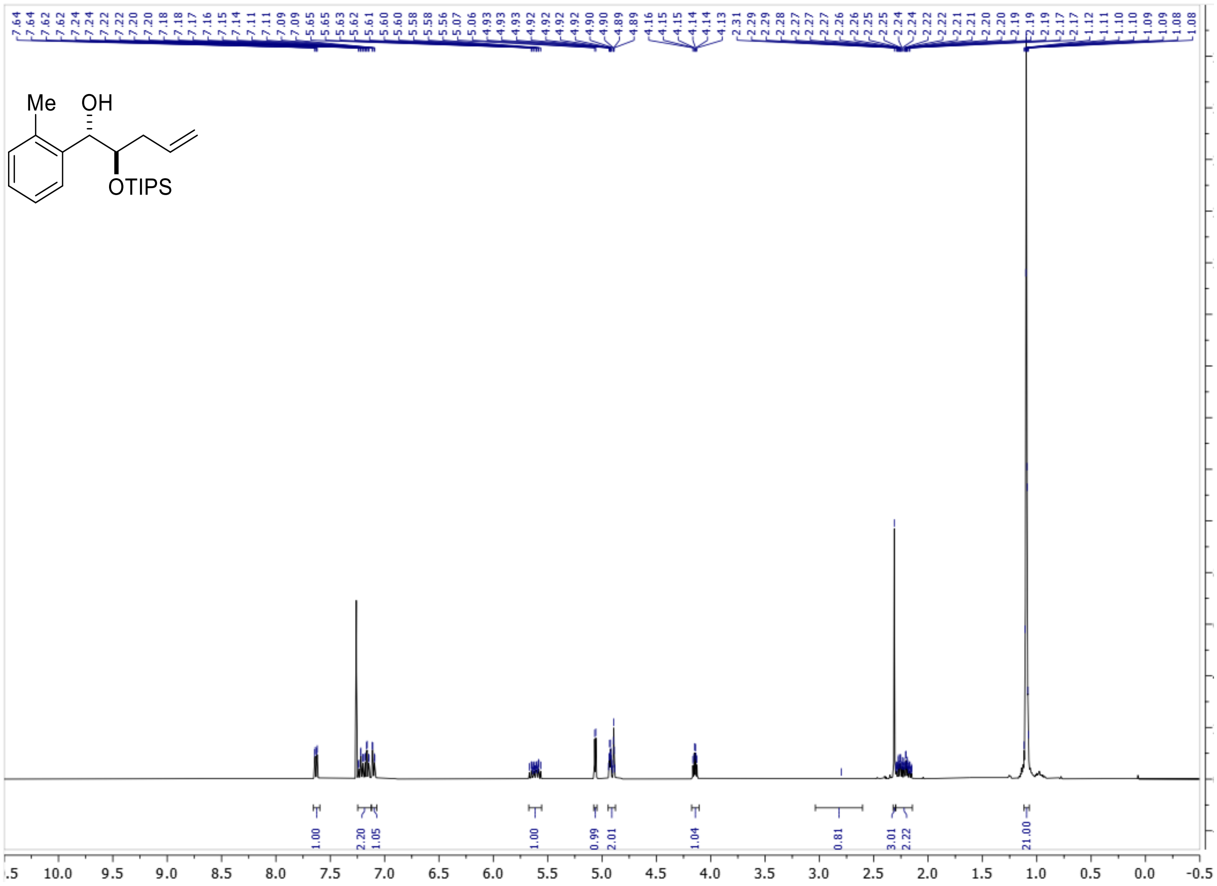

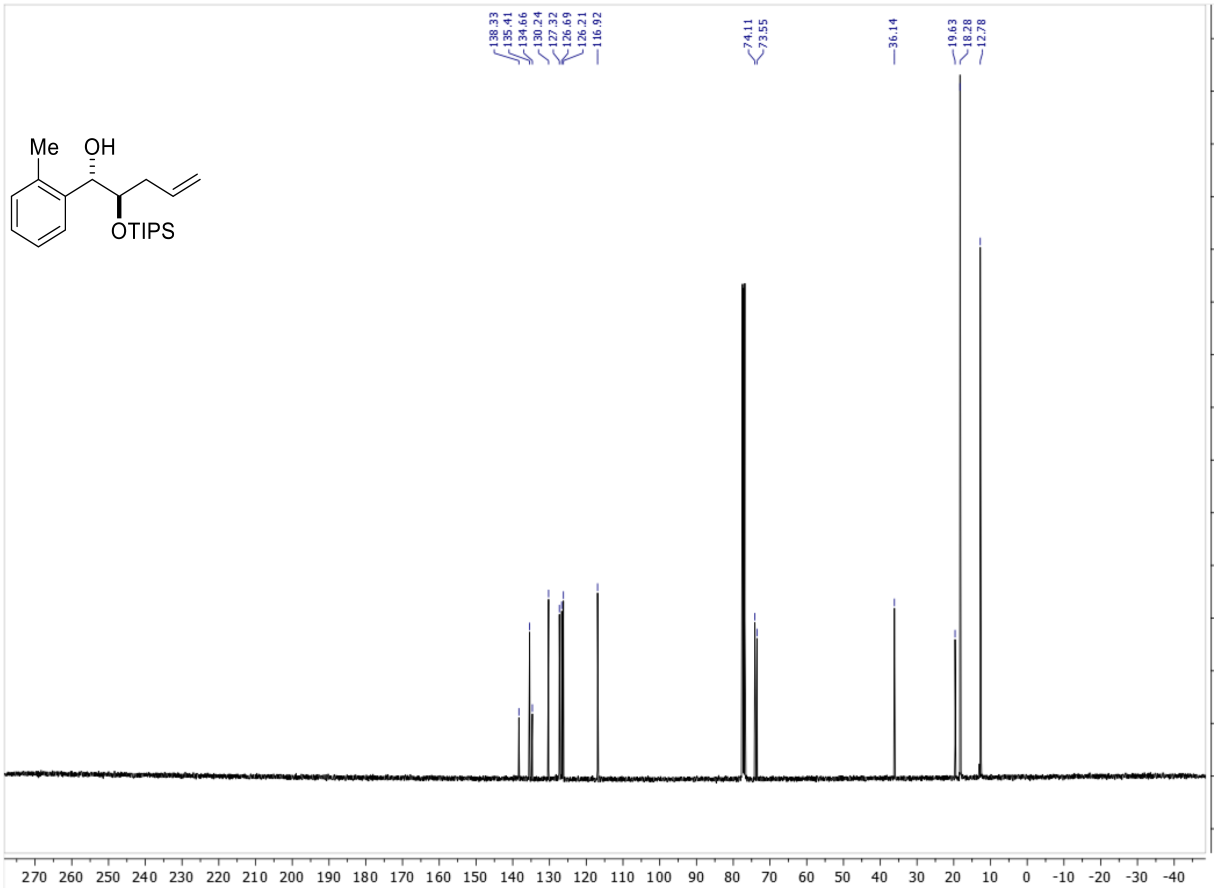

anti-1-(naphthalen-2-yl)-2-((triisopropylsilyl)oxy)pent-4-en-1-ol **18h**

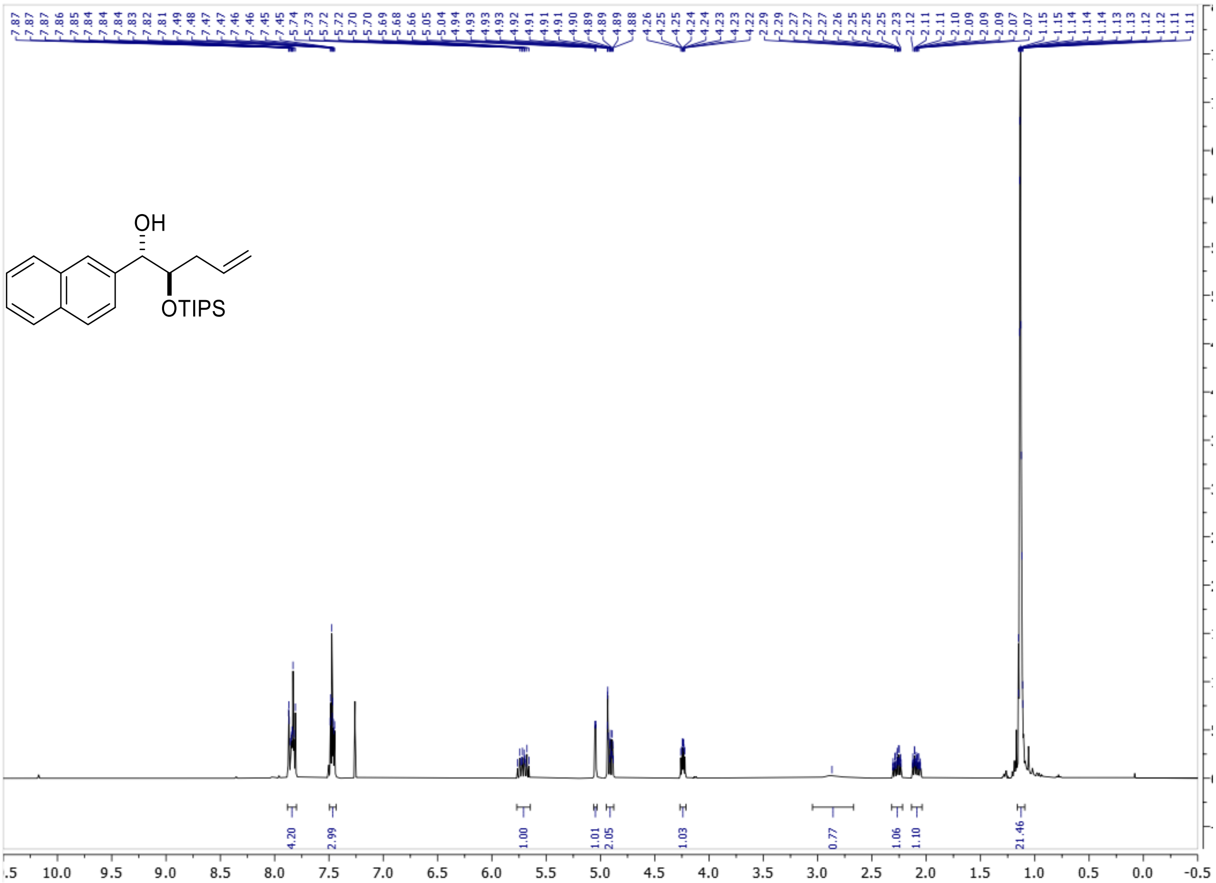

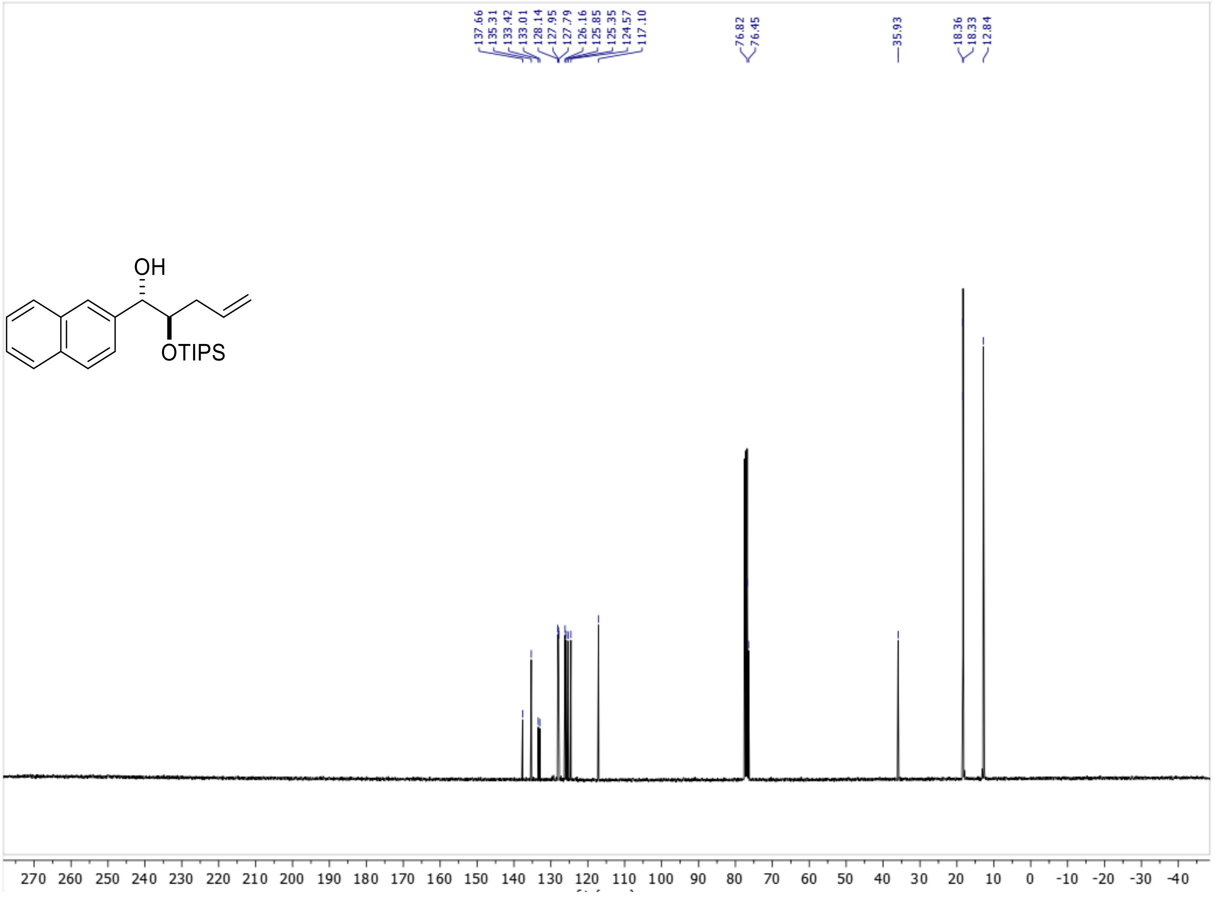

anti-1-(4-fluorophenyl)-2-((triisopropylsilyl)oxy)pent-4-en-1-ol **18i**

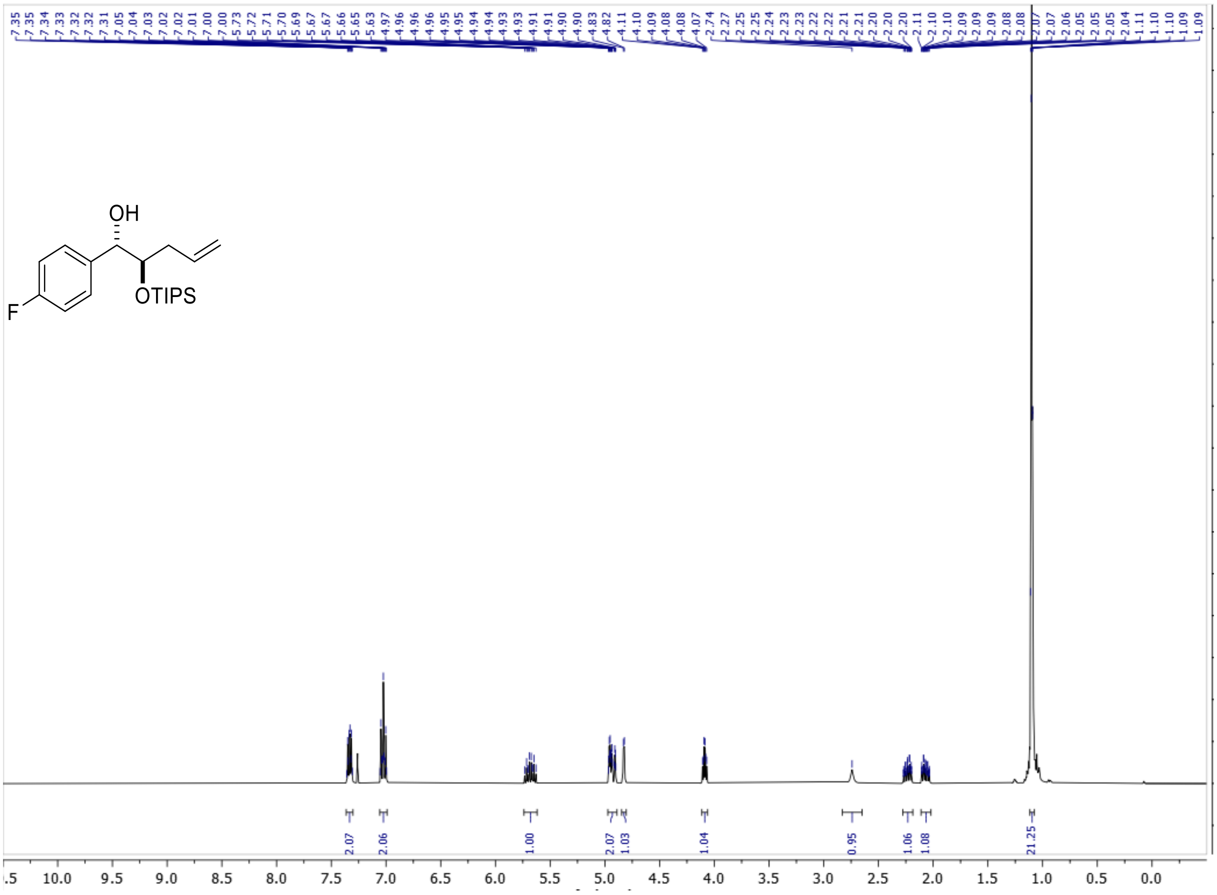

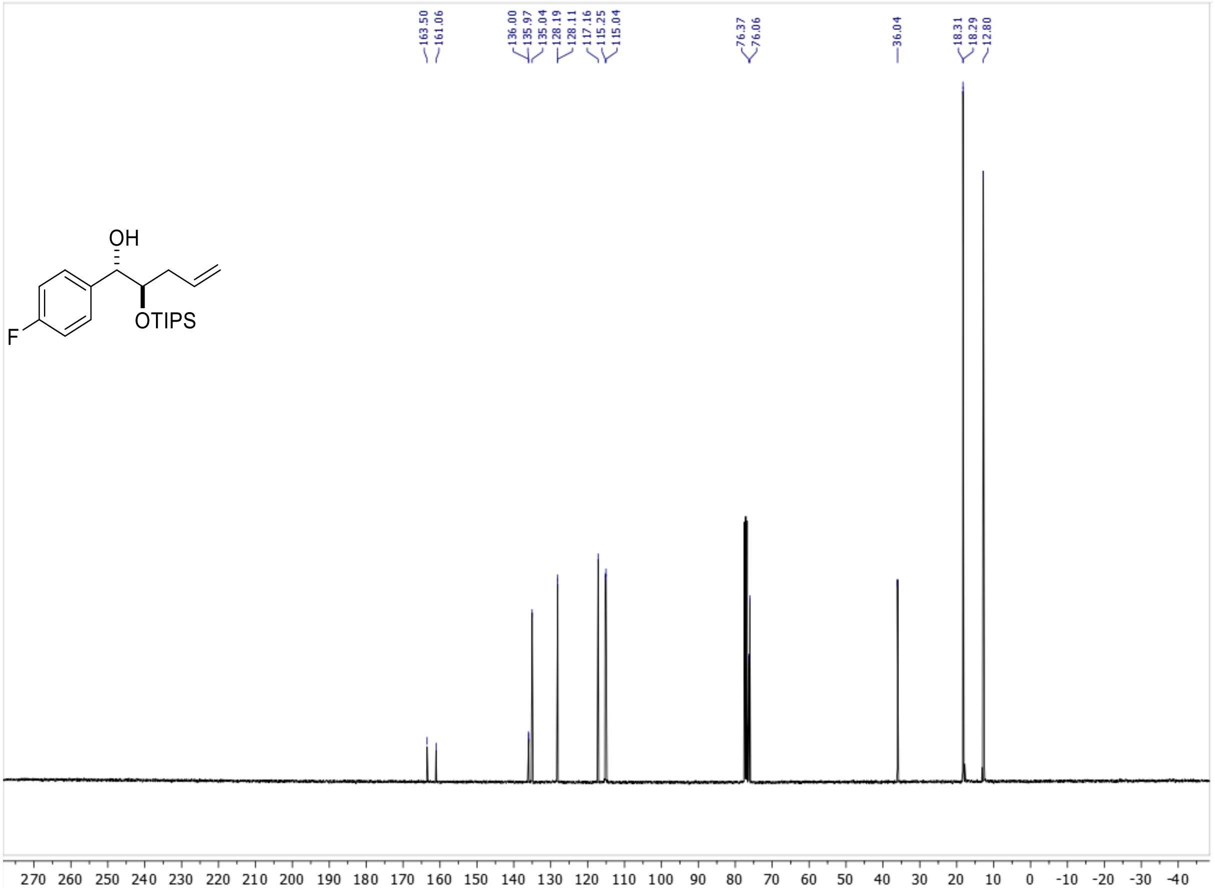

anti-1-(3-chlorophenyl)-2-((triisopropylsilyl)oxy)pent-4-en-1-ol **18j**

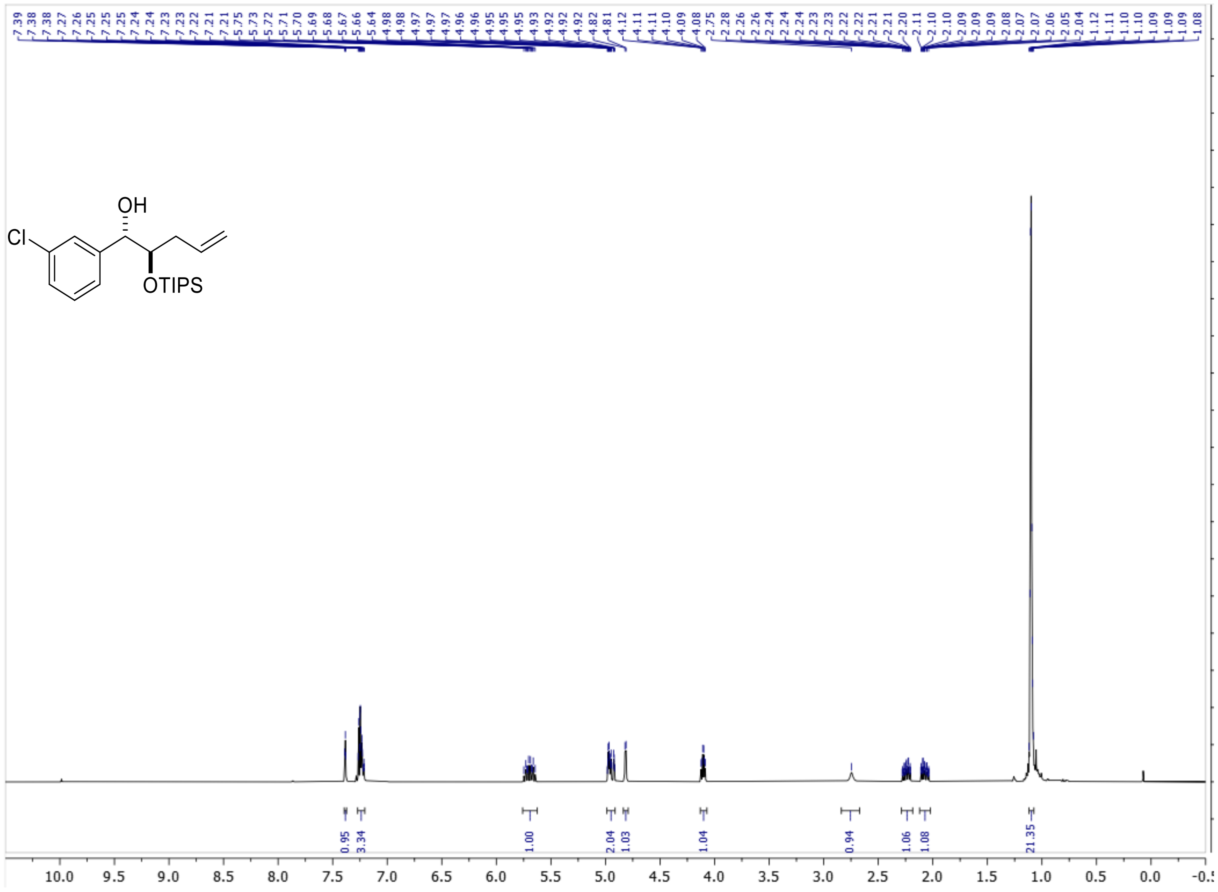

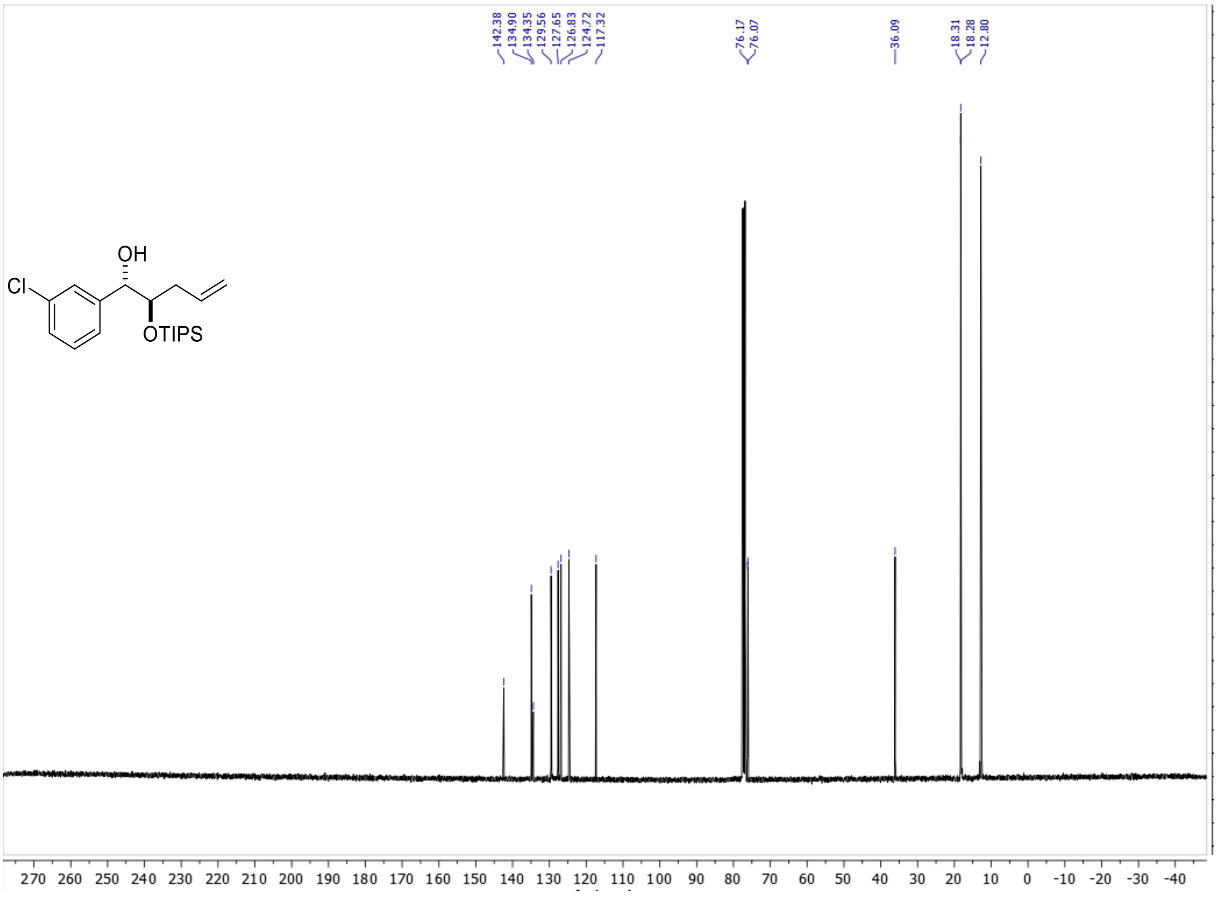

anti-1-(4-(trifluoromethyl)phenyl)-2-((triisopropylsilyl)oxy)pent-4-en-1-ol **18k**

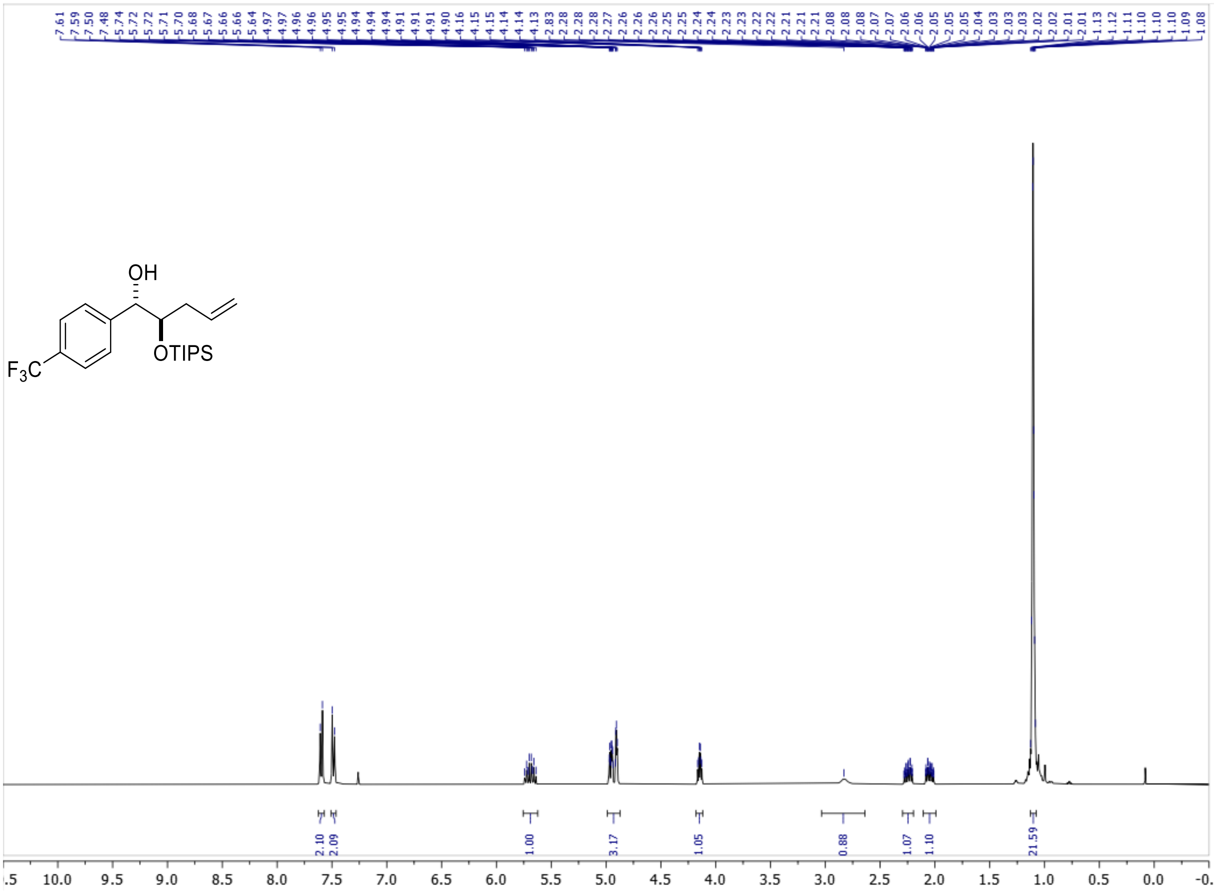

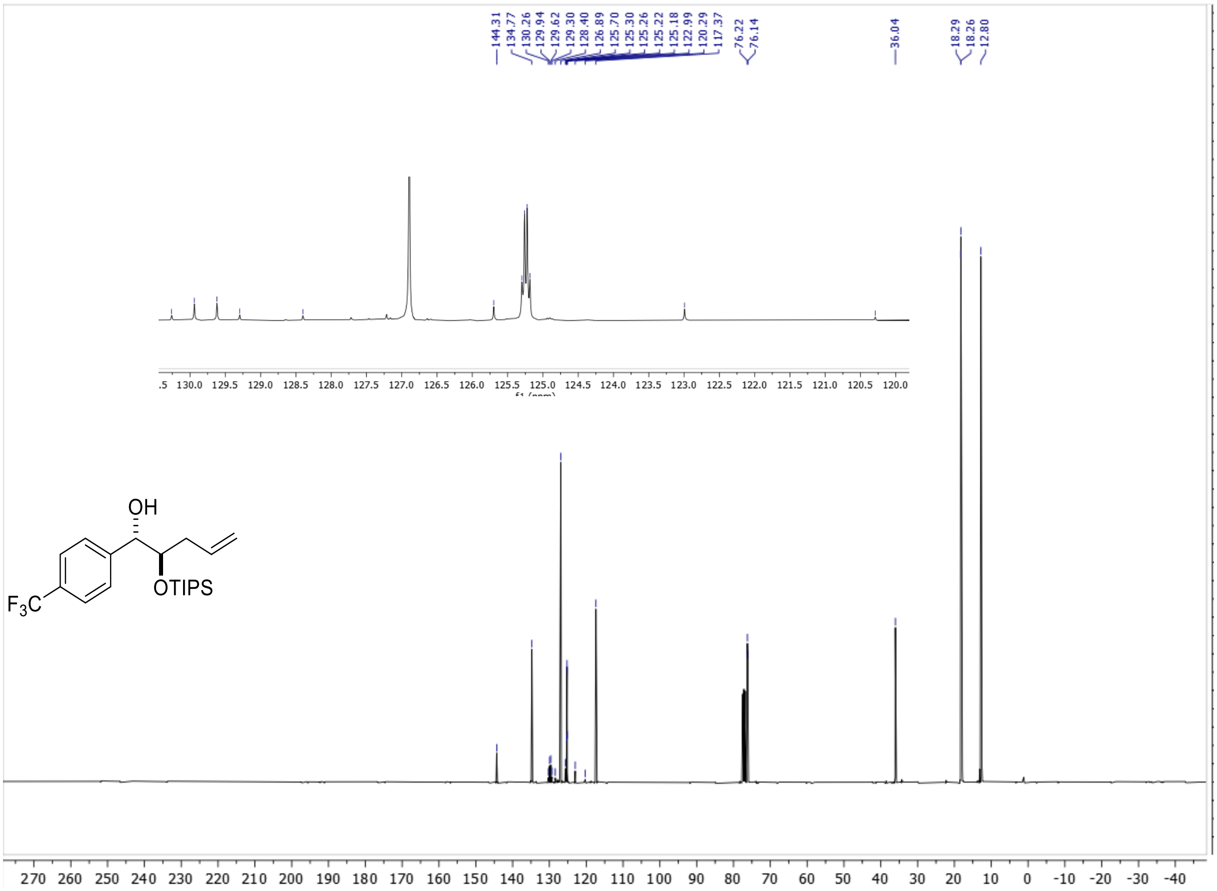

anti-1-(4-(4,4,5,5-tetramethyl-1,3,2-dioxaborolan-2-yl)phenyl)-2-((triisopropylsilyl)oxy)pent-4-en-1-ol **18l**

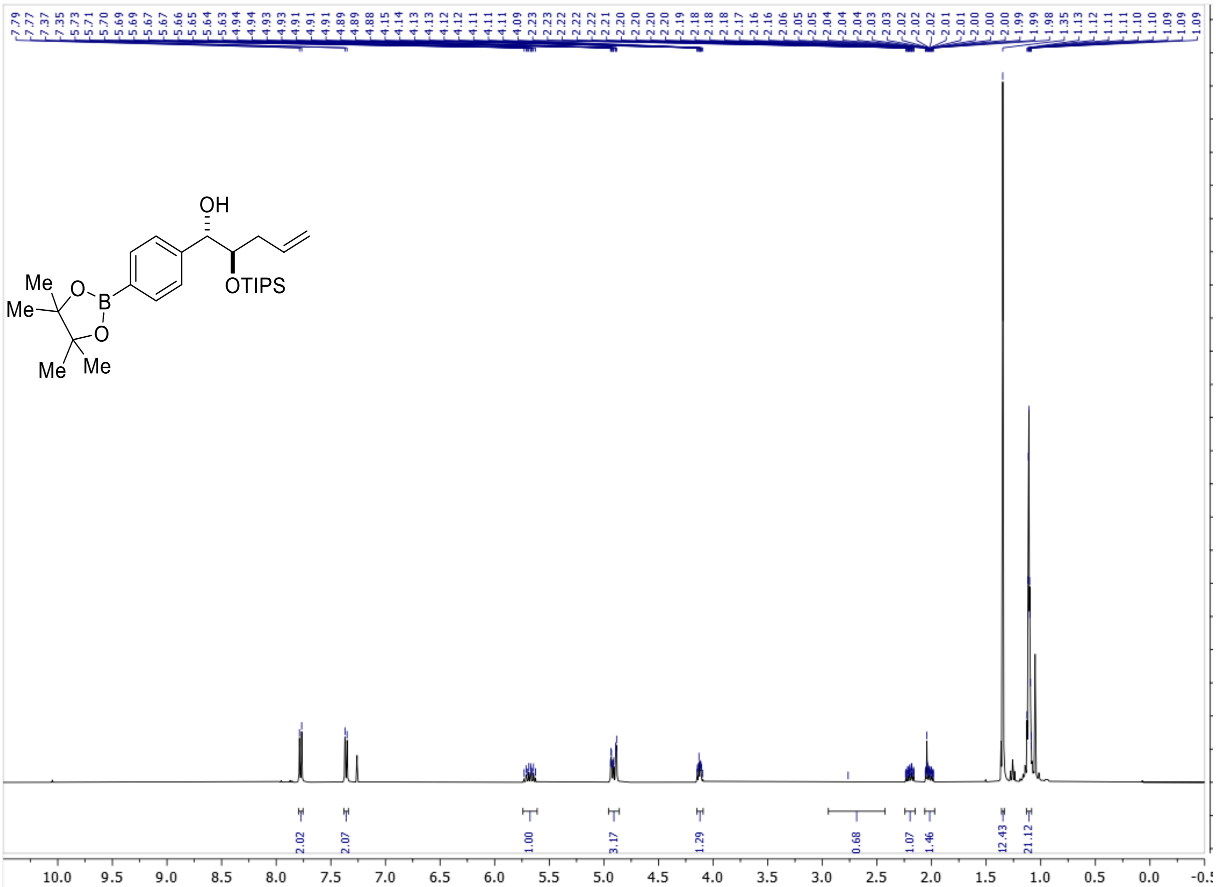

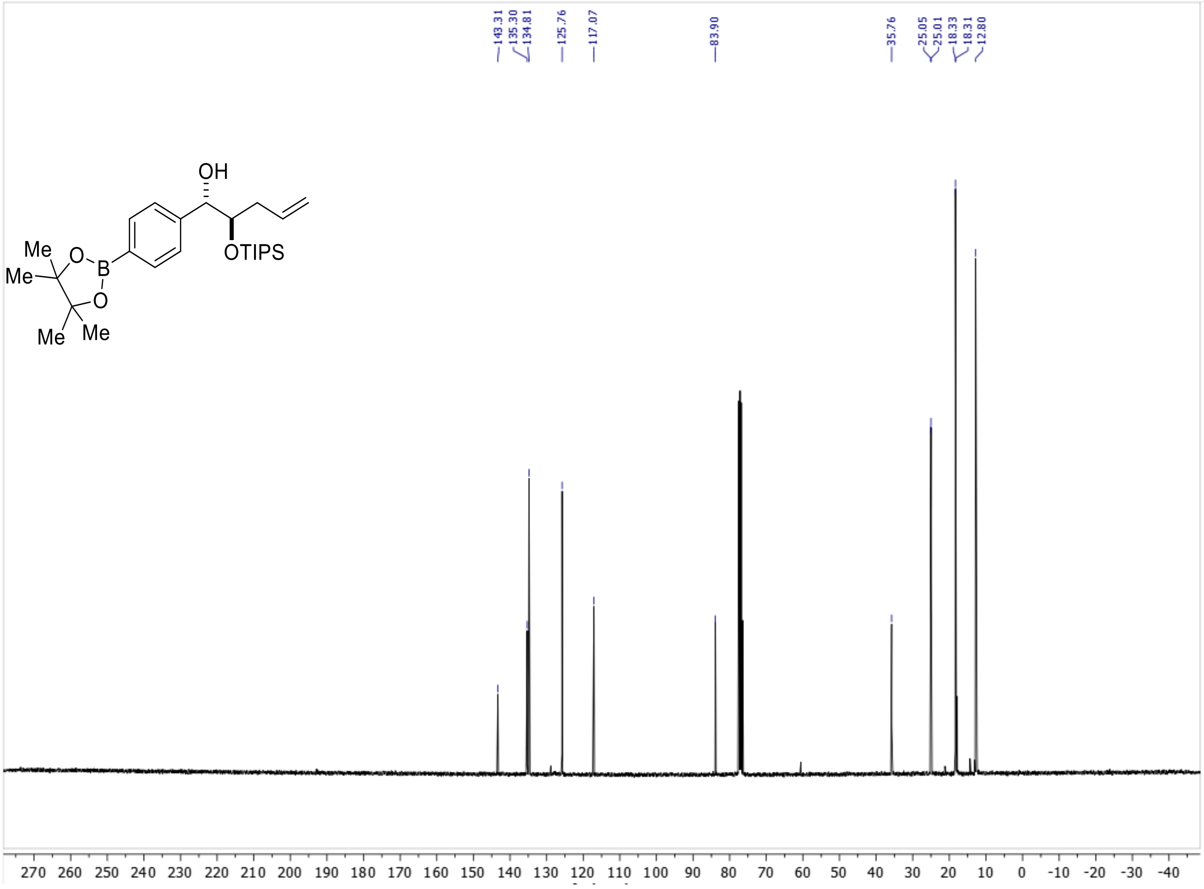

anti-1-(furan-2-yl)-2-((triisopropylsilyl)oxy)pent-4-en-1-ol **18m**

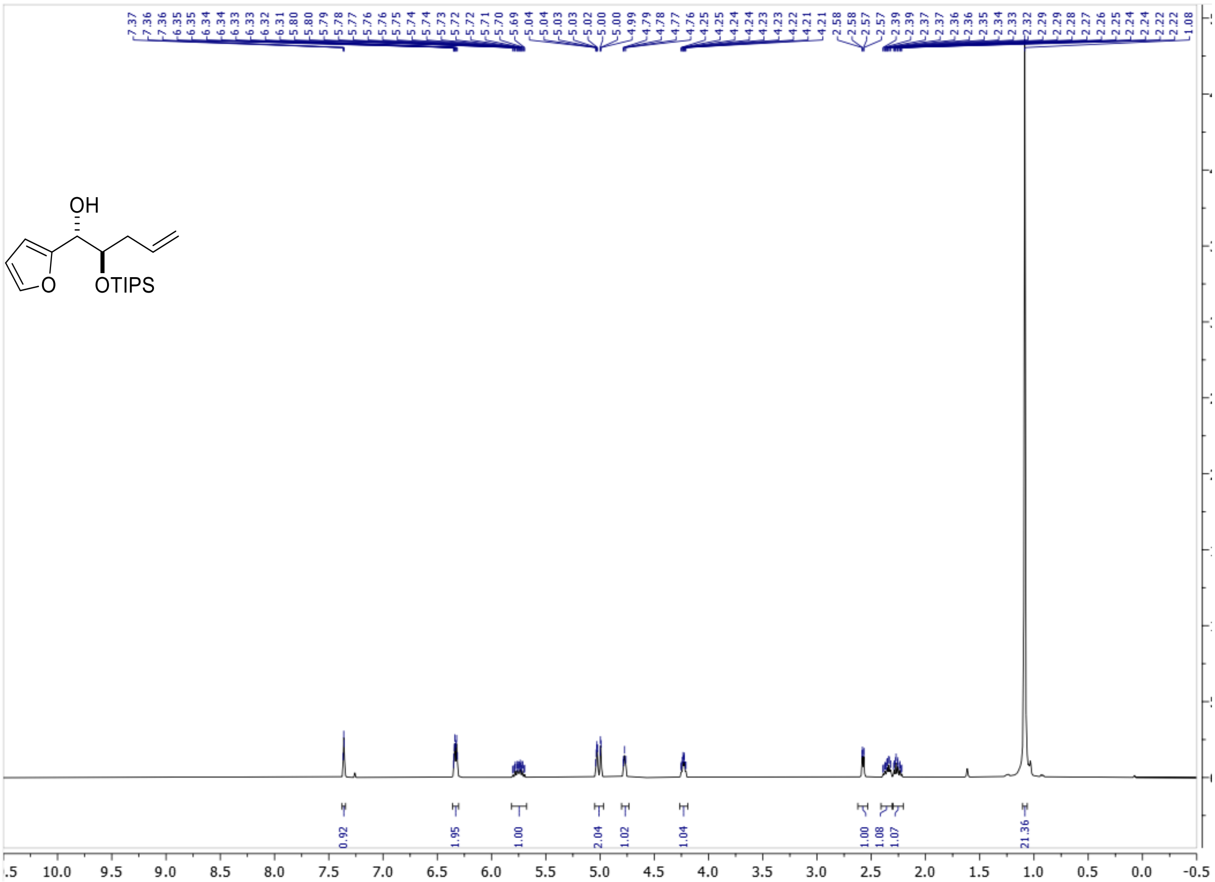

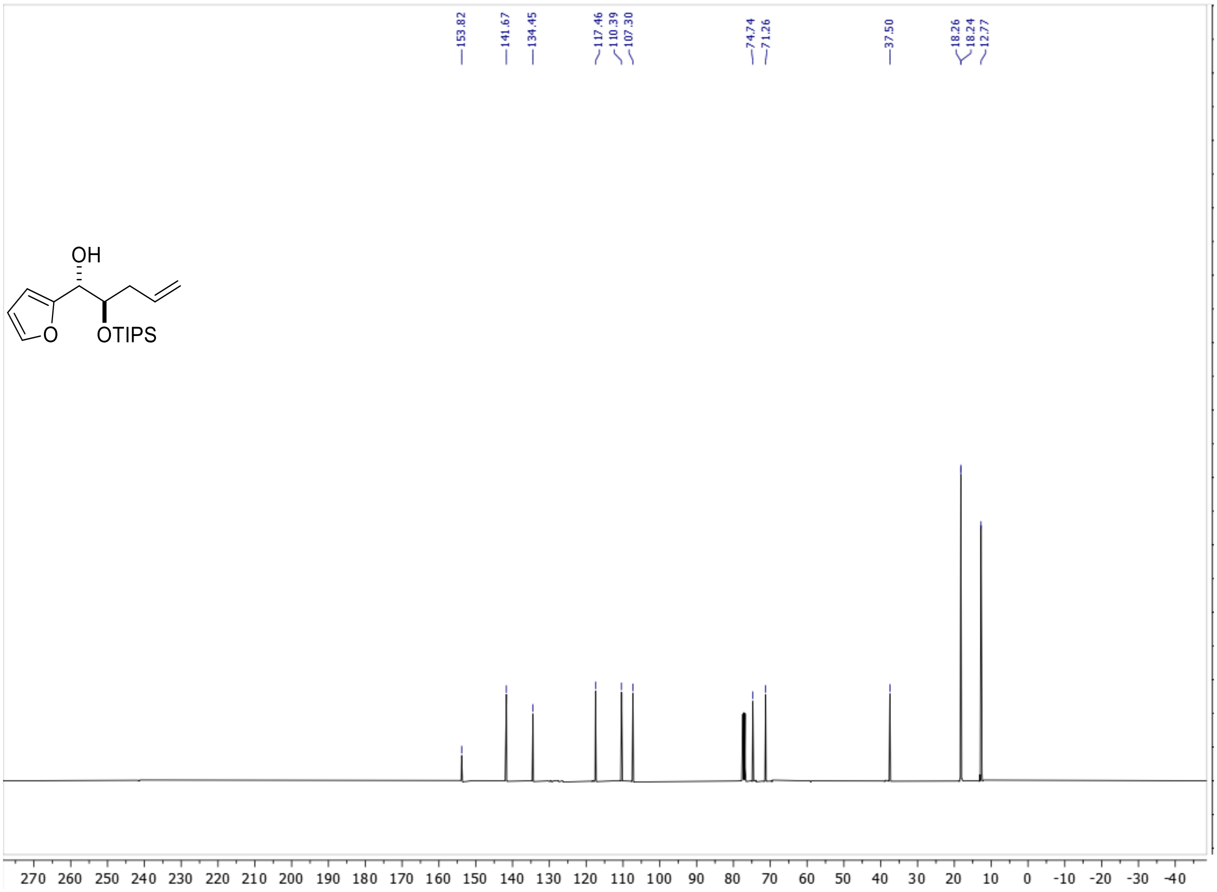

syn-2-((tert-butyldimethylsilyl)oxy)-1-cyclohexylpent-4-en-1-ol **19a**

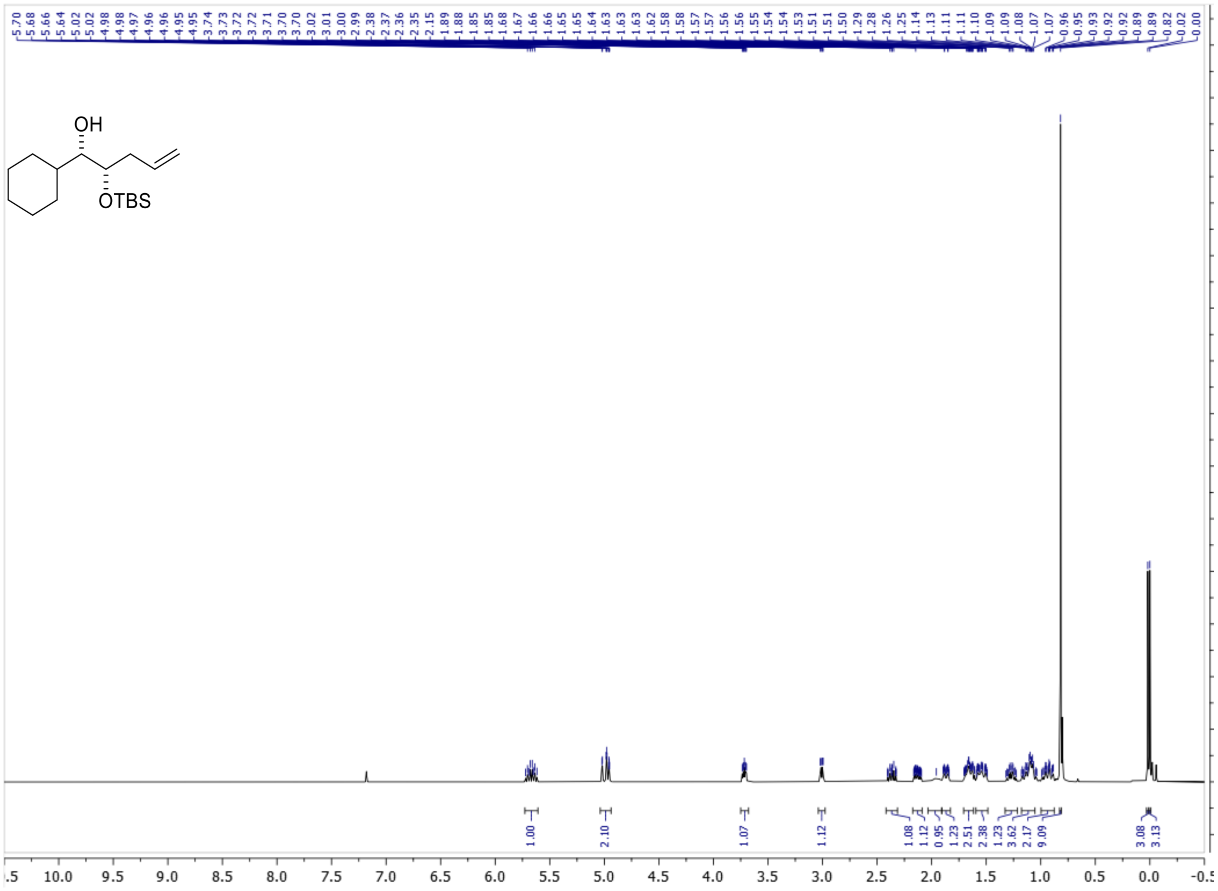

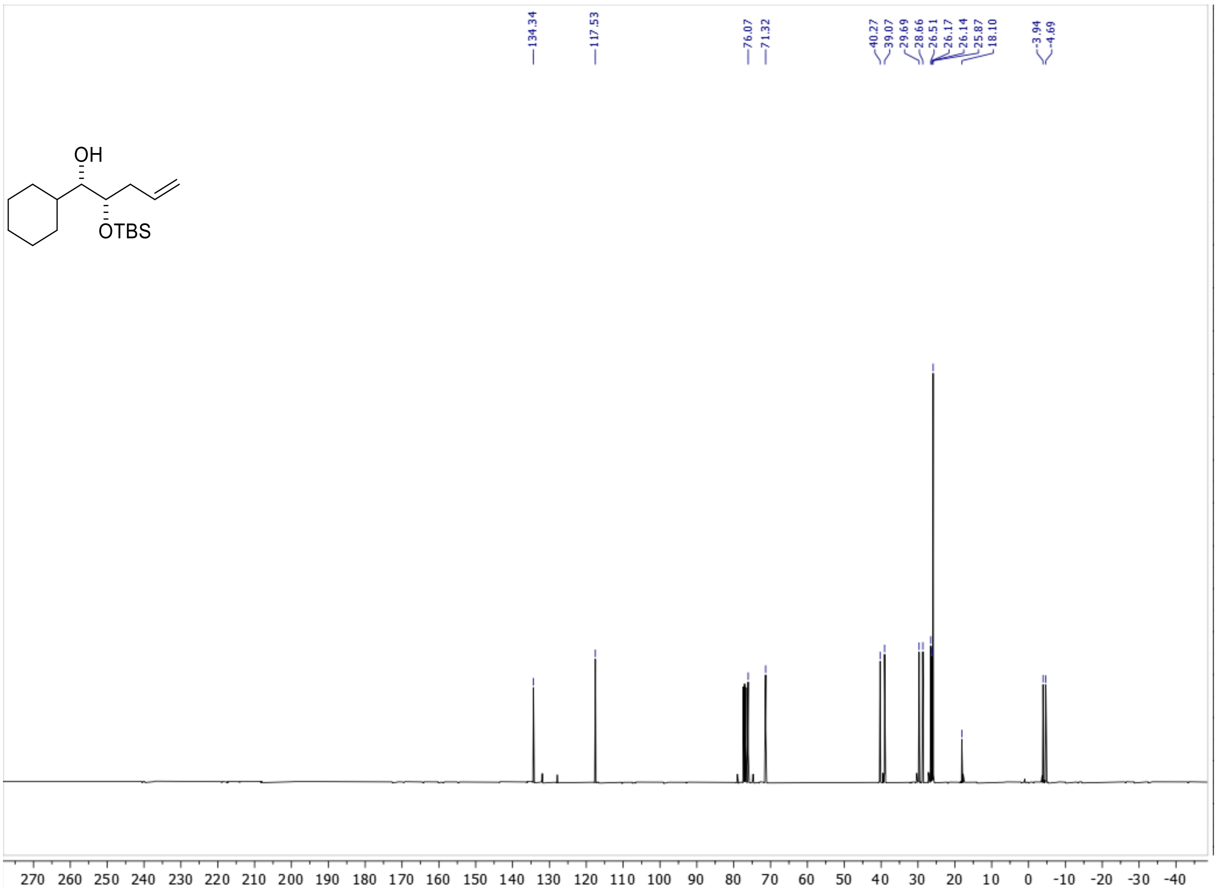

syn-1-cyclohexyl-2-((triisopropylsilyl)oxy)pent-4-en-1-ol **19b**

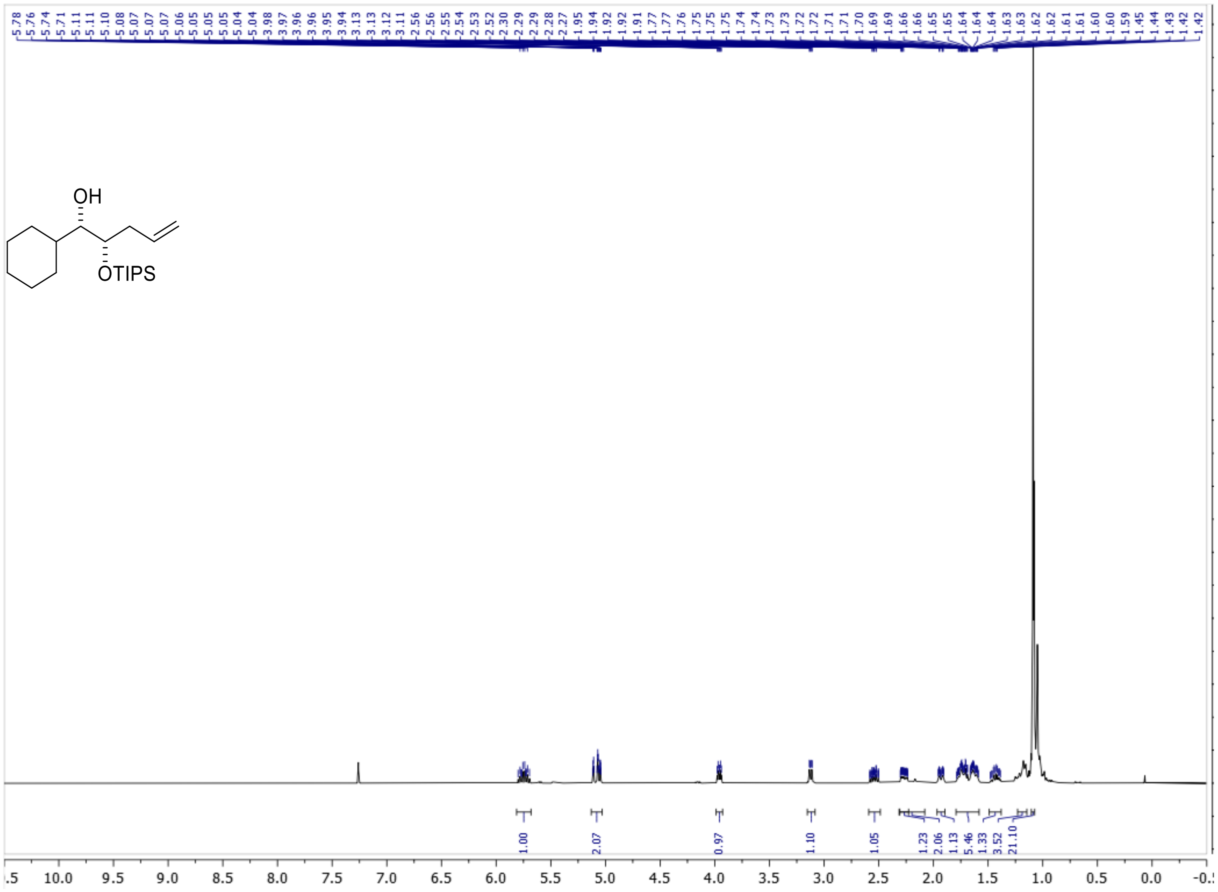

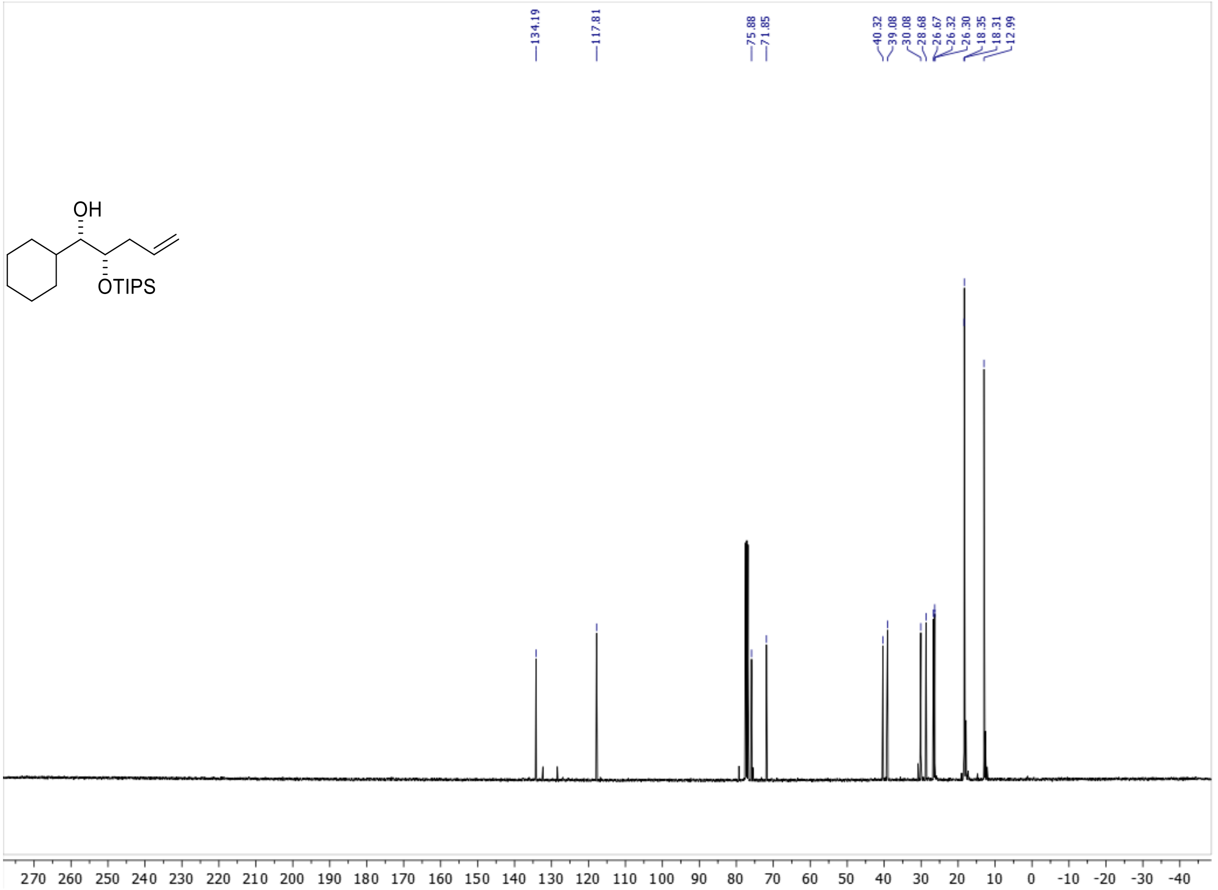

syn-4-((tert-butyldimethylsilyl)oxy)-2-methylhept-6-en-3-ol **19c**

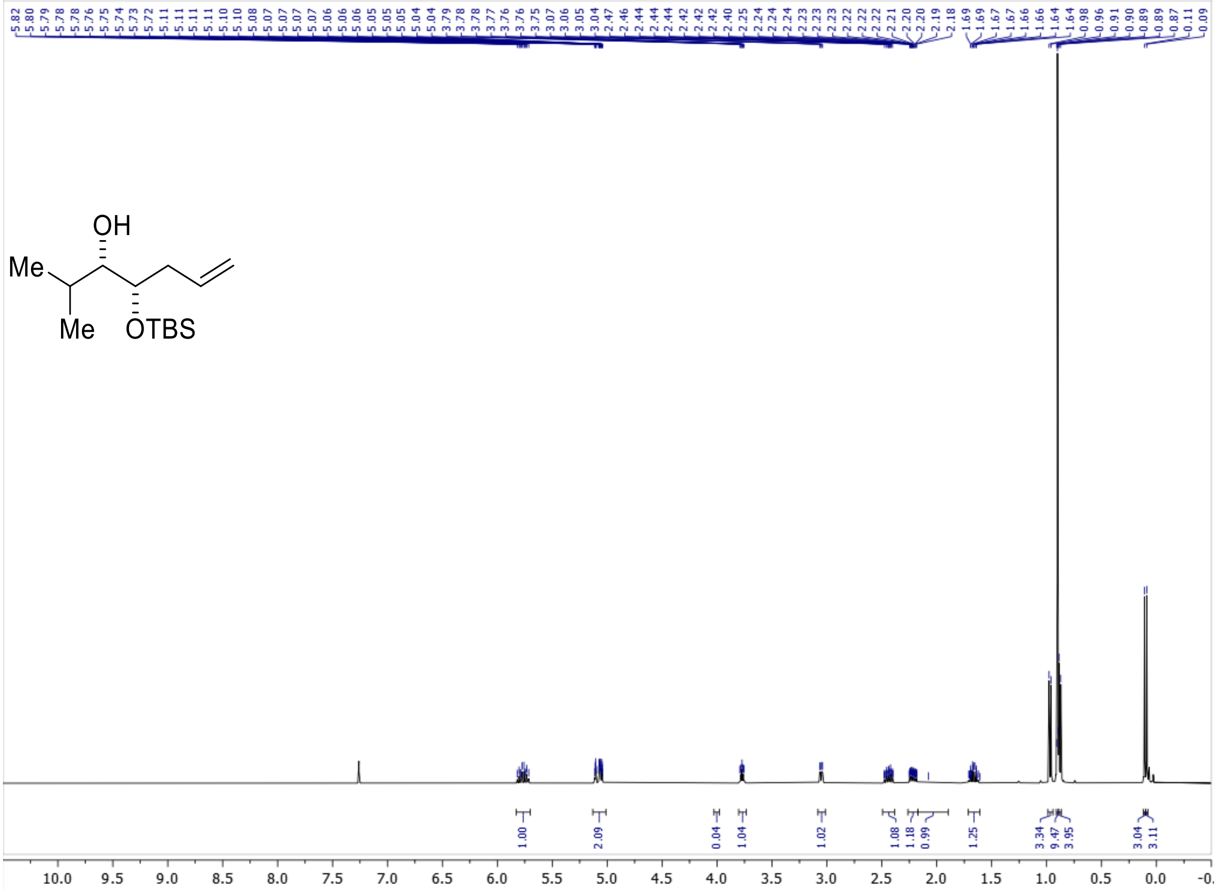

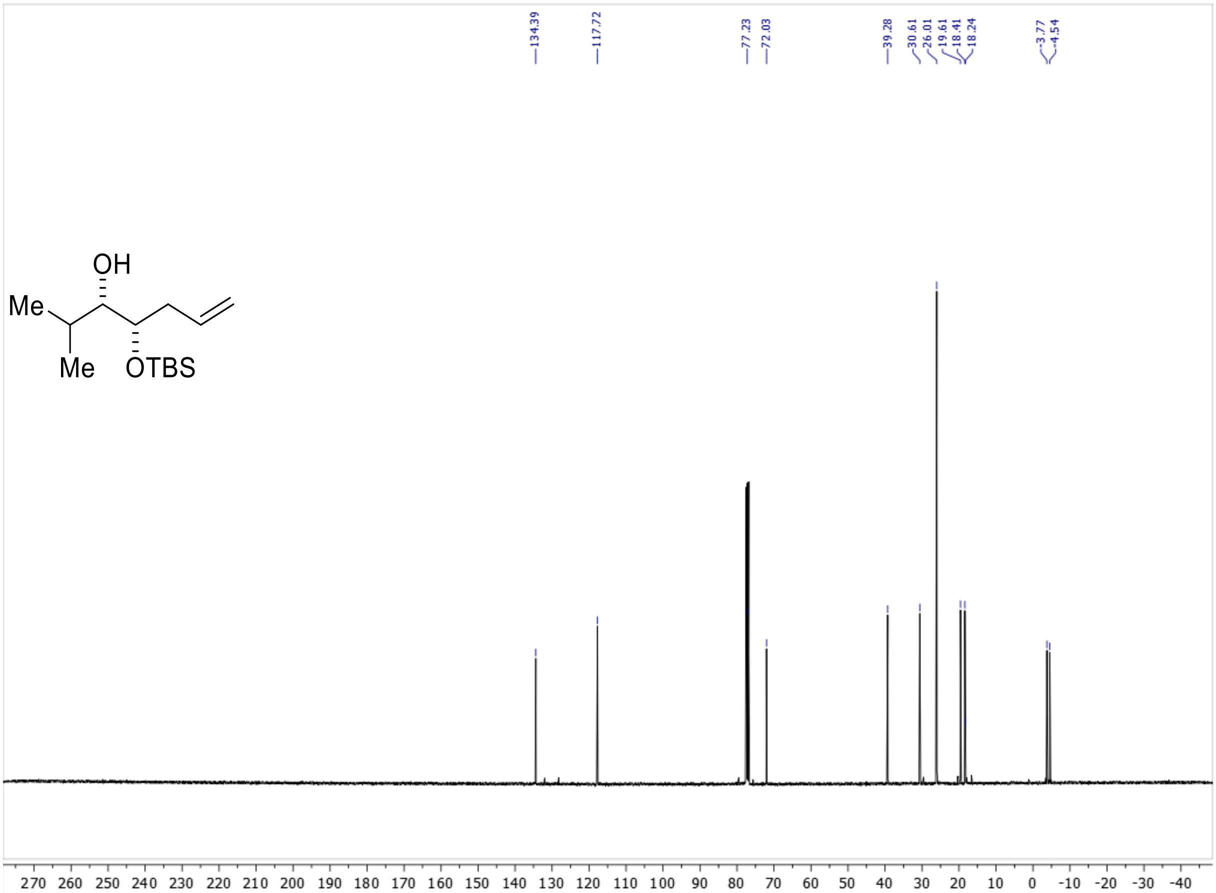

syn-3-((tert-butyldimethylsilyl)oxy)-1-phenylhex-5-en-2-ol **19d**

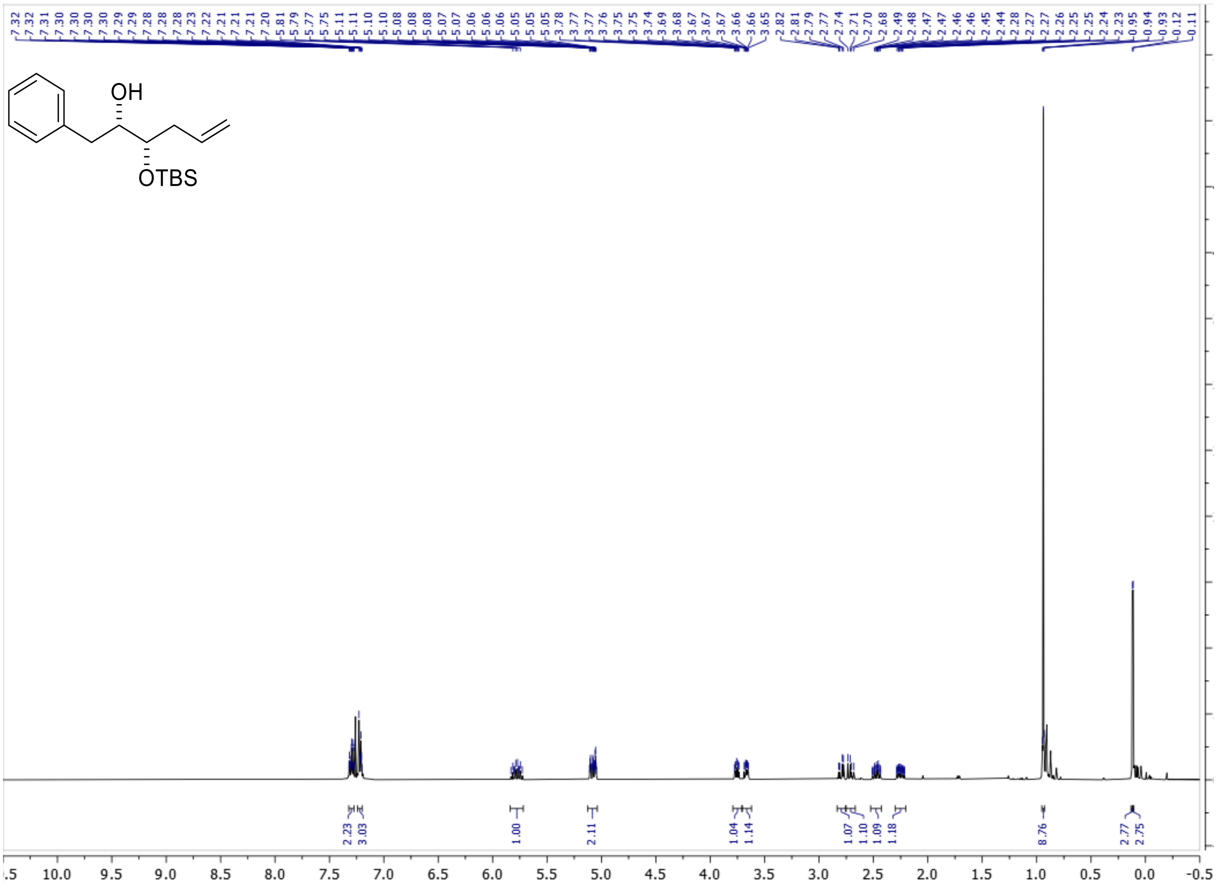

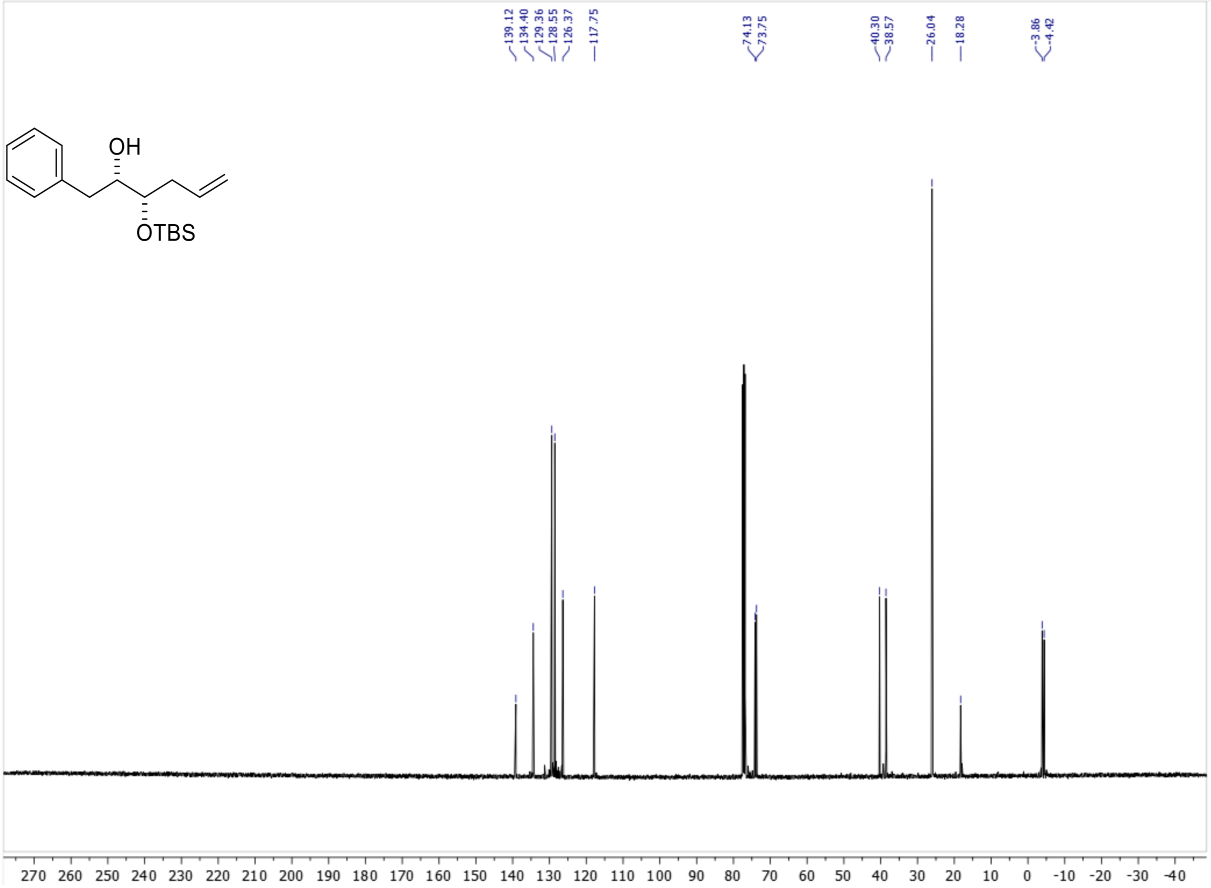

syn-1-([1,1'-biphenyl]-4-yl)-2-((triisopropylsilyl)oxy)pent-4-en-1-ol **19e**

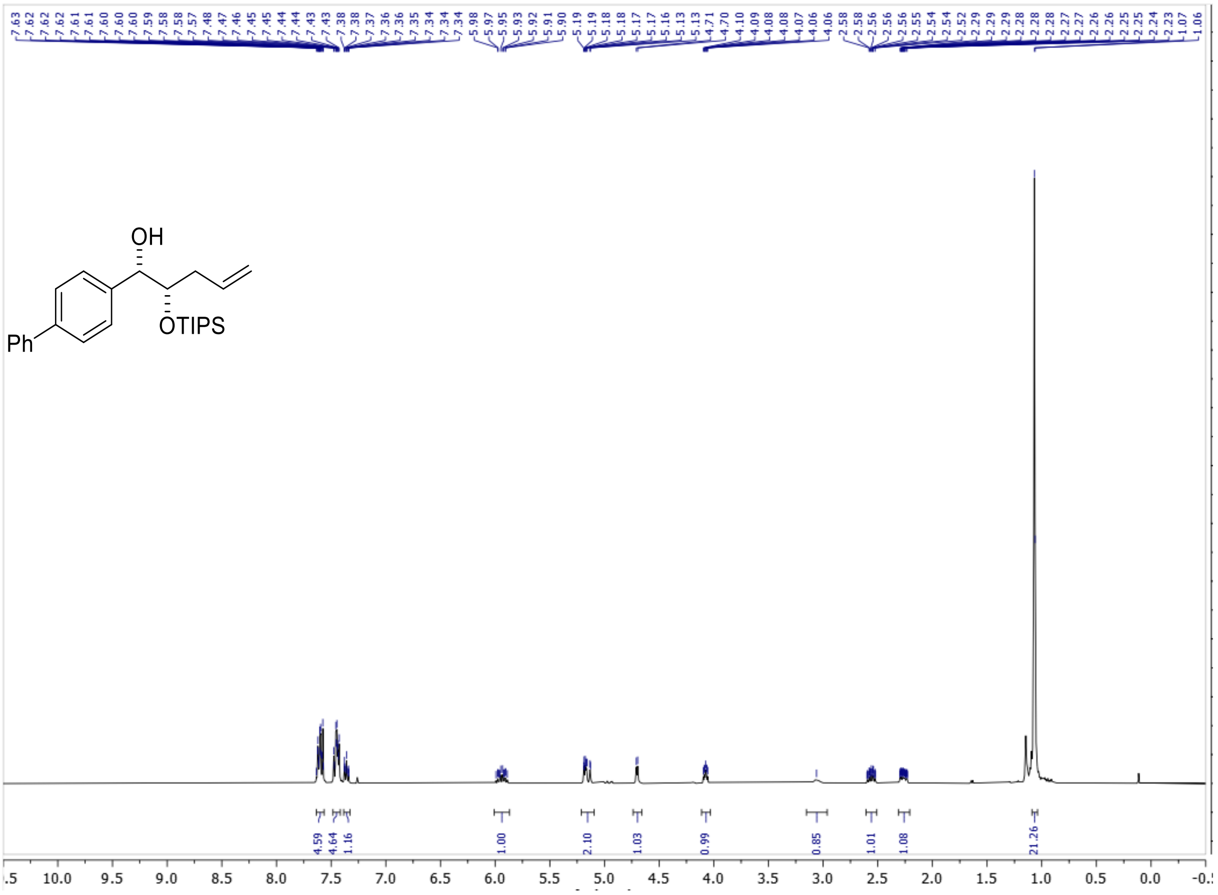

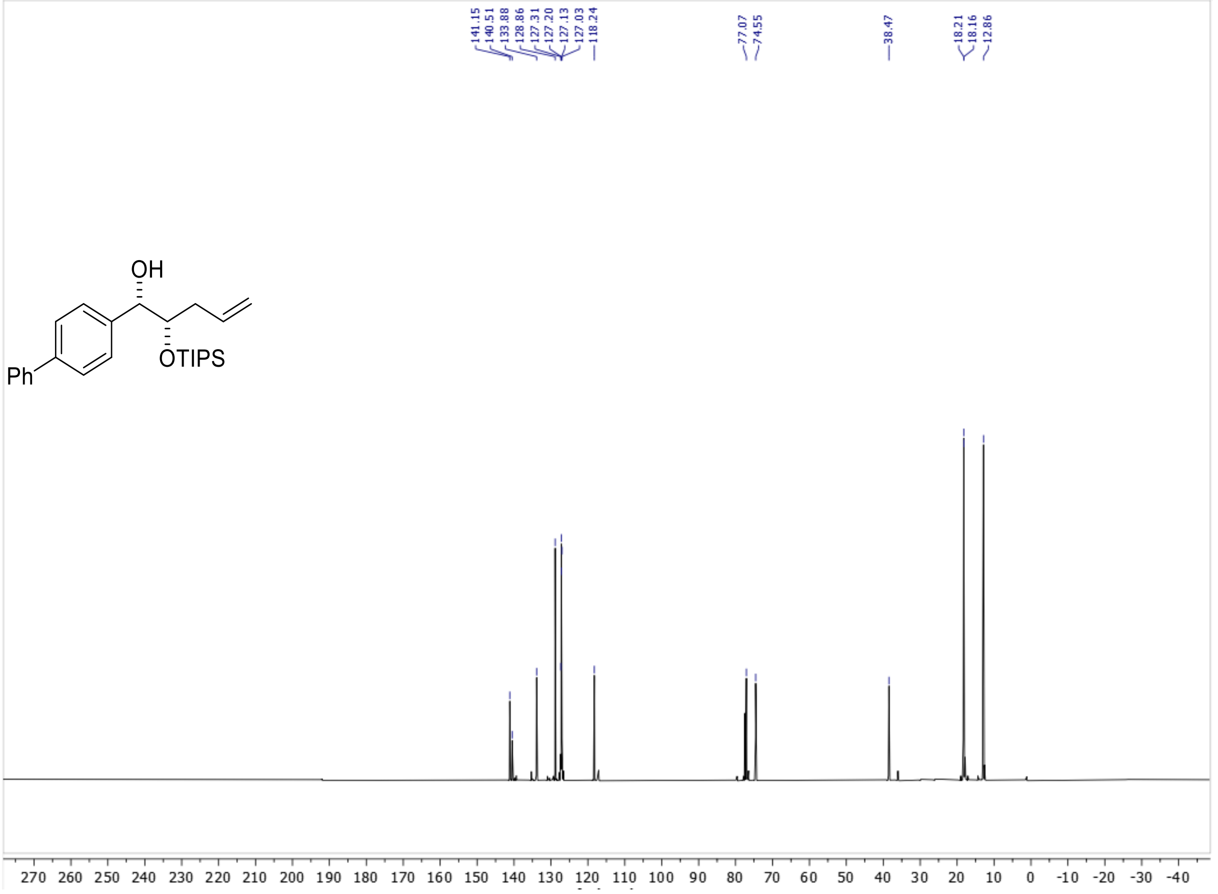

syn-1-([1,1'-biphenyl]-4-yl)-2-(benzyloxy)pent-4-en-1-ol **19f**

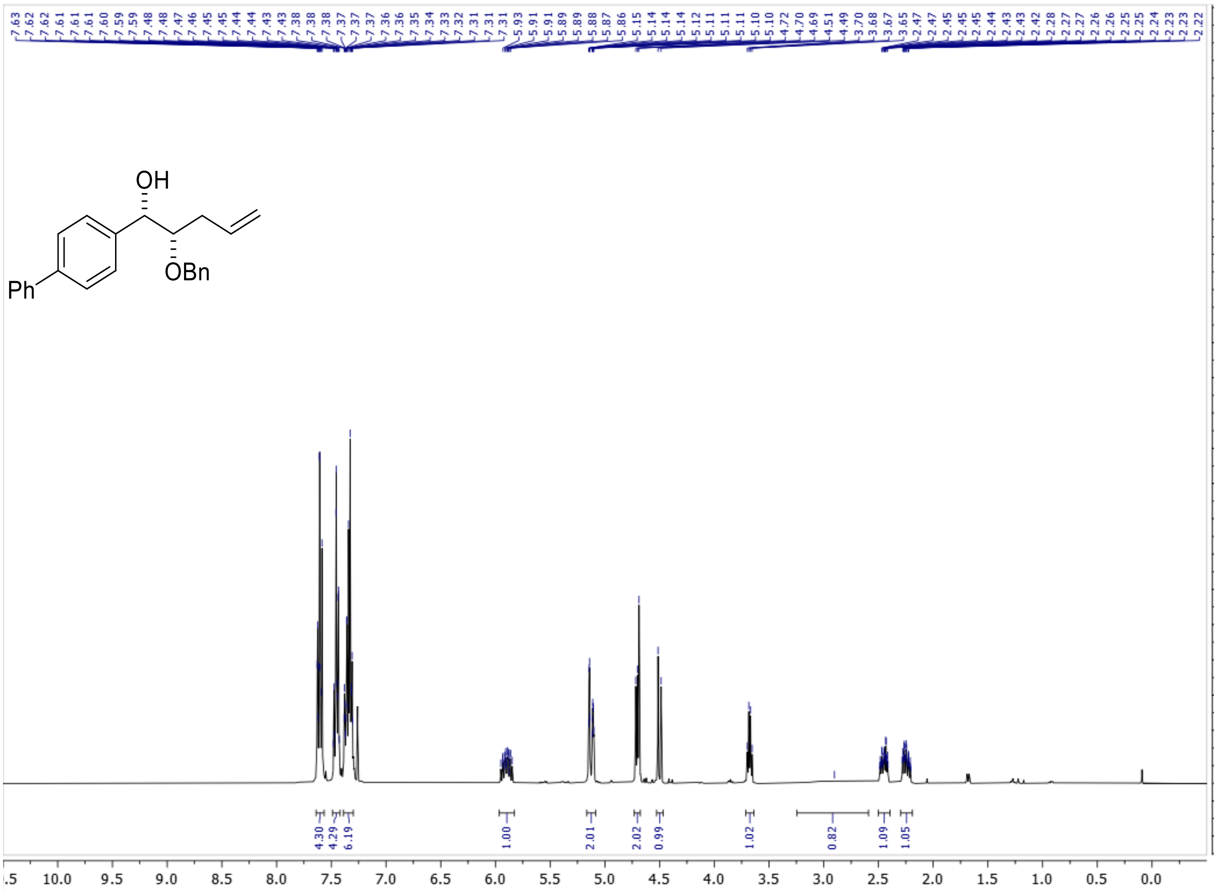

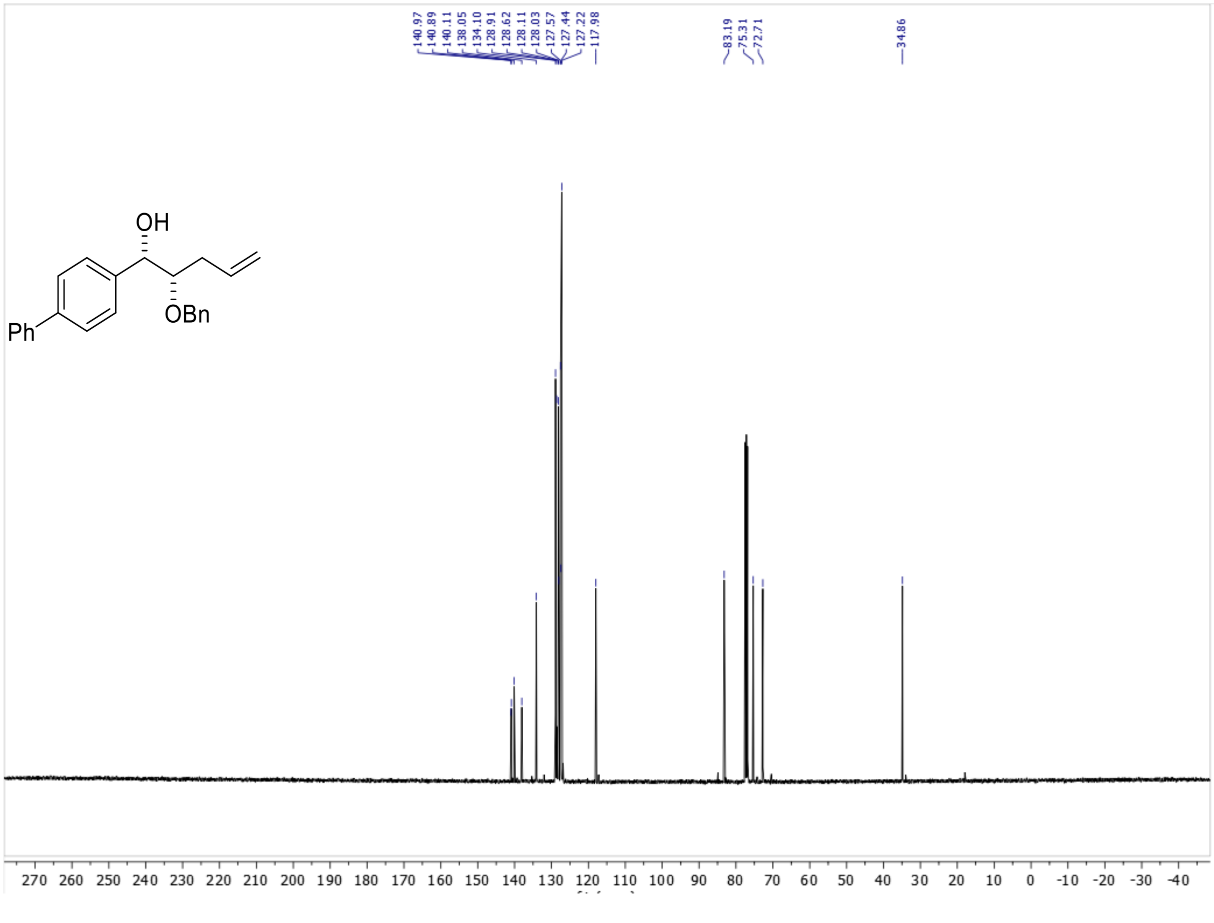

3. References

1. Malik, H. A.; Sormunen, G. J.; Montgomery, J., A General Strategy for Regiocontrol in Nickel-Catalyzed Reductive Couplings of Aldehydes and Alkynes. *J. Am. Chem. Soc.* **2010,** *132* (18), 6304-6305.
2. Teichert, J. F.; Feringa, B. L., Phosphoramidites: Privileged Ligands in Asymmetric Catalysis. *Angew. Chem. Int. Ed.* **2010,** *49* (14), 2486-2528.
3. Coombs, J. R.; Haeffner, F.; Kliman, L. T.; Morken, J. P., Scope and Mechanism of the Pt-Catalyzed Enantioselective Diboration of Monosubstituted Alkenes. *J. Am. Chem. Soc.* **2013,** *135* (30), 11222-11231.
4. Roth, T.; Wadepohl, H.; Wright, D. S.; Gade, L. H., Chiral Ditopic Cyclophosphazane (CycloP) Ligands: Synthesis, Coordination Chemistry, and Application in Asymmetric Catalysis. *Chem. Eur. J.* **2013,** *19* (41), 13823-13837.
5. Zhou, H.; Wang, W.-H.; Fu, Y.; Xie, J.-H.; Shi, W.-J.; Wang, L.-X.; Zhou, Q.-L., Highly Enantioselective Copper-Catalyzed Conjugate Addition of Diethylzinc to Enones Using Chiral Spiro Phosphoramidites as Ligands. *J. Org. Chem.* **2003,** *68* (4), 1582-1584.
6. Yang, Y.; Zhu, S.-F.; Duan, H.-F.; Zhou, C.-Y.; Wang, L.-X.; Zhou, Q.-L., Asymmetric Reductive Coupling of Dienes and Aldehydes Catalyzed by Nickel Complexes of Spiro Phosphoramidites: Highly Enantioselective Synthesis of Chiral Bishomoallylic Alcohols. *J. Am. Chem. Soc.* **2007,** *129* (8), 2248-2249.
7. Hulst, R.; de Vries, N. K.; Feringa, B. L., α-Phenylethylamine based chiral phospholidines; new agents for the determination of the enantiomeric excess of chiral alcohols, amines and thiols by means of 31P NMR. *Tetrahedron: Asymmetry* **1994,** *5* (4), 699-708.
8. van den Berg, M.; Minnaard, A. J.; Haak, R. M.; Leeman, M.; Schudde, E. P.; Meetsma, A.; Feringa, B. L.; de Vries, A. H. M.; Maljaars, C. E. P.; Willans, C. E.; Hyett, D.; Boogers, J. A. F.; Henderickx, H. J. W.; de Vries, J. G., Monodentate Phosphoramidites: A Breakthrough in Rhodium-Catalysed Asymmetric Hydrogenation of Olefins. *Adv. Syn. Cat.* **2003,** *345* (1-2), 308-323.
9. Erre, G.; Junge, K.; Enthaler, S.; Addis, D.; Michalik, D.; Spannenberg, A.; Beller, M., Synthesis of Novel Monodentate Phosphoramidites and Their Application in Iridium-Catalyzed Asymmetric Hydrogenations. *Chem. Asian J.* **2008,** *3* (5), 887-894.
10. Fürstner, A.; Nagano, T.; Müller, C.; Seidel, G.; Müller, O., Total Synthesis and Evaluation of the Actin-Binding Properties of Microcarpalide and a Focused Library of Analogues. *Chemistry – A European Journal* **2007,** *13* (5), 1452-1462.
11. Schulz, A.; Villinger, A.; Westenkirchner, A., Synthesis of 1,3-Dichloro-cyclo-1,3-diphosphadiazanes from Silylated Amino(dichloro)phosphanes. *Inorganic Chemistry* **2013,** *52* (19), 11457-11468.
12. Nocquet, P. A.; Mace, A.; Legros, F.; Lebreton, J.; Dujardin, G.; Collet, S.; Martel, A.; Carboni, B.; Carreaux, F., Stereodivergent approach in the protected glycal synthesis of L-vancosamine, L-saccharosamine, L-daunosamine and L-ristosamine involving a ring-closing metathesis step. *Beilstein J Org Chem* **2018,** *14*, 2949-2955.
13. Sawano, T.; Yamamoto, H., Enantioselective Epoxidation of β,γ-Unsaturated Carboxylic Acids by a Cooperative Binuclear Titanium Complex. *ACS Catal.* **2019,** *9* (4), 3384-3388.
14. Jiao, P.; Kawasaki, M.; Yamamoto, H., A Sequential O-Nitrosoaldol and Grignard Addition Process: An Enantio- and Diastereoselective Entry to Chiral 1,2-Diols. *Angew. Chem. Int. Ed.* **2009,** *48* (18), 3333-3336.
15. Kimura, M.; Ezoe, A.; Mori, M.; Iwata, K.; Tamaru, Y., Regio- and stereoselective nickel-catalyzed homoallylation of aldehydes with 1,3-dienes. *J Am Chem Soc* **2006,** *128* (26), 8559-8568.
16. Mechsner, B.; Henssen, B.; Pietruszka, J., First enantioselective total synthesis of altersolanol A. *Org Biomol Chem* **2018,** *16* (41), 7674-7681.

17. Bose, D.; Frey, W.; Pietruszka, J., The 'Mikami'-Catalyst in Enantioselective Diels-Alder Reactions of Juglone-Based Dienophiles with Different 1-Oxygenated Dienes: An Investigation on the Substitution Pattern Dependent Regioselectivity. *Synthesis-Stuttgart* **2014,** *46* (18), 2524-2532.
18. LeBideau, F.; Gilloir, F.; Nilsson, Y.; Aubert, C.; Malacria, M., Stereocontrolled synthesis of alpha-trialkylsilyl-beta,gamma-unsaturated aldehydes via palladium(0) catalysis. Synthetic usefulness. *Tetrahedron* **1996,** *52* (21), 7487-7510.
19. Emery, K. J.; Tuttle, T.; Murphy, J. A., Evidence of single electron transfer from the enolate anion of an N,N'-dialkyldiketopiperazine additive in BHAS coupling reactions. *Org Biomol Chem* **2017,** *15* (41), 8810-8819.
20. Tayama, E.; Saito, S., Copper-Catalyzed Regiospecific and 1,2-Regioselective Cyclopropanation of (1*Z*)-1-Amino- and (1*Z*)-1-Oxy-1,3-butadienyl Derivatives. *Synlett* **2015,** *26* (13), 1880-1884.
